# Single-Cell RNA-Seq Reveals a Crosstalk between Hyaluronan Receptor LYVE-1-Expressing Macrophages and Vascular Smooth Muscle Cells

**DOI:** 10.3390/cells11030411

**Published:** 2022-01-25

**Authors:** Fabienne Burger, Daniela Baptista, Aline Roth, Karim J. Brandt, Rafaela Fernandes da Silva, Fabrizio Montecucco, François Mach, Kapka Miteva

**Affiliations:** 1 Division of Cardiology, Foundation for Medical Research, Department of Medicine Specialized Medicine, Faculty of Medicine, University of Geneva, Av. de la Roseraie 64, 1206 Geneva, Switzerland; fabienne.burger@unige.ch (F.B.); daniela.baptista@unige.ch (D.B.); aline.roth@unige.ch (A.R.); Karim.Brandt@hcuge.ch (K.J.B.); rfdasilva.ufmg@gmail.com (R.F.d.S.); Francois.mach@hcuge.ch (F.M.); 2 Department of Physiology and Biophysics, Institute of Biological Sciences, Federal University of Minas Gerais, Belo Horizonte 6627, Brazil; 3 Swiss Institute for Translational and Entrepreneurial Medicine, Freiburgstrasse 3, 3010 Bern, Switzerland; 4 Ospedale Policlinico San Martino Genoa—Italian Cardiovascular Network, 10 Largo Benzi, 16132 Genoa, Italy; fabrizio.montecucco@unige.it; 5 First Clinic of Internal Medicine, Department of Internal Medicine and Centre of Excellence for Biomedical Research (CEBR), University of Genoa, 6 Viale Benedetto XV, 16132 Genoa, Italy

**Keywords:** resident-like macrophages, LYVE-1, CCL24, VSMC transdifferentiation, osteogenic-like cells, vascular calcification

## Abstract

**Background**: Atherosclerosis is a chronic inflammatory disease where macrophages participate in the progression of the disease. However, the role of resident-like macrophages (res-like) in the atherosclerotic aorta is not completely understood. **Methods:** A single-cell RNA sequencing analysis of CD45^+^ leukocytes in the atherosclerotic aorta of apolipoprotein E–deficient (*Apoe*^−/−^) mice on a normal cholesterol diet (NCD) or a high cholesterol diet (HCD), respecting the side-to-specific predisposition to atherosclerosis, was performed. A population of res-like macrophages expressing hyaluronan receptor LYVE-1 was investigated via flow cytometry, co-culture experiments, and immunofluorescence in human atherosclerotic plaques from carotid artery disease patients (CAD). **Results:** We identified 12 principal leukocyte clusters with distinct atherosclerosis disease-relevant gene expression signatures. LYVE-1^+^ res-like macrophages, expressing a high level of CC motif chemokine ligand 24 (CCL24, eotaxin-2), expanded under hypercholesteremia in *Apoe*^−/−^ mice and promoted VSMC phenotypic modulation to osteoblast/chondrocyte-like cells, ex vivo, in a CCL24-dependent manner. Moreover, the abundance of LYVE-1^+^CCL24^+^ macrophages and elevated systemic levels of CCL24 were associated with vascular calcification and CAD events. **Conclusions:** LYVE-1 res-like macrophages, via the secretion of CCL24, promote the transdifferentiation of VSMC to osteogenic-like cells with a possible role in vascular calcification and likely a detrimental role in atherosclerotic plaque destabilization.

## 1. Introduction

Macrophages represent an immune cell population of major interest in the progression of chronic inflammation-driven diseases, such as atherosclerosis [1]. However, recent studies have demonstrated that the bipolar M1/M2 classification does not accurately describe the diversity of macrophages [2]. One of the key features of macrophages is their high degree of plasticity that allows them to produce a fine-tuned response to microenvironmental triggers, such as the enrichment of activated immune cells, modified lipoproteins, and proinflammatory factors, as well as dying and apoptotic cells found in atherosclerosis. Moreover, aortic intima resident macrophages are sustained by local proliferation and express a subset of microenvironment-driven genes in combination with the original resident macrophage gene profile [3]. An important tool is the recently developed transcriptome analysis, single-cell RNA sequencing (scRNAseq) [4], which allows a greater understanding of the complexity, abundance, and functional state of atherosclerosis-associated macrophages and their activation variations [2]. Resident macrophages expressing the hyaluronan (HA) receptor LYVE-1 have been shown to degrade collagen on VSMC [5]. The depletion of LYVE1^+^ macrophages resulted in increased arterial stiffness and an increased collagen deposition, suggesting a key role in the maintenance of arterial tone [5]. Three macrophage subsets have been shown to express *Lyve1* in atherosclerotic plaques of *Ldlr*^−/−^ mice using scRNAseq [6] It has been suggested that atherosclerotic aortas contain resident macrophages originating from an embryonic pool, which, upon atherosclerosis development, are replaced by, or accompanied by, recruited monocyte-derived macrophages that adopt a resident-like macrophage phenotype and who play a role in endocytosis [7]. However, how the lesional microenvironment orchestrates macrophage phenotypes and their role during the progression of atherosclerosis is still not completely clear.

Vascular calcification results in hydroxyapatite deposition in the arterial wall, which is linked to an increased risk of heart disease, stroke, atherosclerotic plaque ruptures, vessel stiffness, and systolic hypertension, as well as increased diastolic dysfunction and heart failure [8]. The calcification of both the intimal and medial layers is an active and tightly regulated process, principally driven by VSMCs. The VSMC phenotypic switch results in the transition toward a synthetic phenotype of a dedifferentiated state, characterized by the decrease or loss of VSMC-specific cytoskeletal proteins and the acquisition of the markers of macrophages, mesenchymal stem cells, and myofibroblasts [9], as well as osteoblasts and chondrocytes, contributing to the destabilization of the lesions in late-stage atherosclerosis [10]. Macrophages are known to be involved in almost all stages of vascular calcification, as well as directly promoting the differentiation of VSMCs into osteoblastic phenotypes [11]. However, the specific mechanism by which macrophages influence the progression of vascular calcification has not been fully elucidated. Moreover, the present therapeutic options only target the factors associated with the development of vascular calcification with attempts at regulating the impaired calcium–phosphate metabolism, and no therapy has, so far, been designed to directly target the mediators of vascular calcification. Progress in this area is dependent on targeting cells and soluble mediators, promoting plaque destabilization.

The present study aims to reveal leukocyte clusters with distinct atherosclerosis disease-relevant gene expression signatures and to unveil their role in atherosclerosis pathology. We identified an atherosclerosis-associated LYVE-1^+^ res-like macrophage population via RNAseq analysis, which was found to trigger VSMC transdifferentiation to osteoblast/chondrocyte-like cells, in a CCL24-dependent manner. Accumulation of LYVE-1^+^ res-like macrophage in human atherosclerotic plaques and increased level of CCL24 has been associated with the occurrence of CAD events.

## 2. Materials and Methods

### 2.1. Animals

Eleven-week-old male *Apoe*^−/−^ C57Bl/6 mice, or *Apoe*^−/−^ Myh11-CreERT2, ROSA26 STOP-flox eYFP^+/+^ mice, were fed an NCD (4.6% fat, 21.1% protein, 4.5% fiber, 6.4% ash, Special Diets Services, Essex, UK) for 16 weeks (early atherogenesis) [12] or an HCD for 11 weeks (20.1% fat, 1.25% cholesterol, Research Diets, Inc., New Brunswick, NJ, USA) to promote advanced atherogenesis [13]. To facilitate VSMC lineage tracing, an injection of tamoxifen was used to induce Cre recombinase activation in male *Apoe*^−/−^ Myh11-CreERT2, ROSA26 STOP-flox eYFP^+/+^ mice. A series of ten intraperitoneal 1 mg tamoxifen (Sigma, St. Louis, MO, USA) injections were given from 9 to 11 weeks of age, for a total of 10 mg of tamoxifen per mouse. An average bodyweight of 25 g for the 2 weeks running up to the start of the high cholesterol diet was performed [14]. Whole blood was collected, and the serum triglycerides, total cholesterol, and low-density lipoprotein cholesterol (LDL-C) were measured. Animals were sacrificed by exsanguination after anesthesia with 4% isoflurane. Experimental protocols and procedures were reviewed and approved by the Institutional Animal Care and Use Committee of the Geneva University School of Medicine. Animal care and experimental procedures were carried out in accordance with the guidelines of the Institutional Animal Care and Use Committee of the Geneva University School of Medicine. All procedures comply with the guidelines of directive 2010/63/EU of the European Parliament on the protection of animals used for scientific purposes, as well as the NIH Guide for the Care and Use of Laboratory Animals.

### 2.2. Tissue Processing, Cell Staining and Flow Cytometry

**Figure 1:**
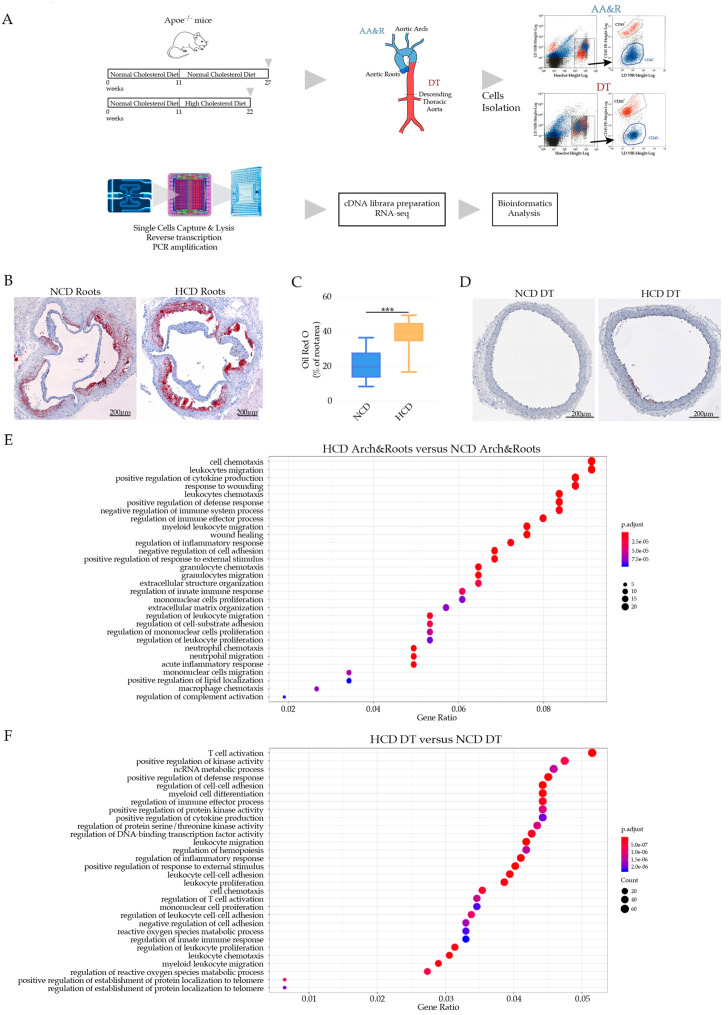
(**A**) Experimental setting of scRNAseq of CD45^+^ cells of *Apoe^−/−^* on NCD versus HCD. (**B**) Oil Red O-stained atherosclerotic lesions of *Apoe^−/−^* mice on NCD and HCD. (**C**) Bar graphs represent the mean ± SEM of atherosclerotic lesion quantification in aortic roots with 6–8 mice /group and *** *p* < 0.001. (**D**) Oil Red O-stained DT aorta of *Apoe^−/−^* on NCD and HCD. (**E**) Bubble plot GO term enrichment analysis of cells derived from AA&R of *Apoe^−/−^* on NCD versus HCD. Dot size is proportional to the number of genes overlapping with each GO term, while the adjusted *p*-value is color-coded from red to blue. (**F)** Bubble plot GO term enrichment analysis of cells derived from DT aorta of *Apoe^−/−^* mice on NCD versus HCD. Dot size is proportional to the number of genes overlapping with each GO term, while the adjusted *p*-value is color-coded from red to blue.

After the intracardial perfusion of *Apoe*^−/−^ C57Bl/6 mice on NCD or HCD, the aorta was surgically excised. The aorta adventitia was carefully excised by a sharp surgical dissection in a clearly defined plane, to leave a naked media over the length of the AA&R (the aortal segment from just left of the branchpoint for the brachiocephalic artery to just right of the branchpoint for the left subclavian artery) and the DT aorta (the straight aortal segment after the arch to the renal aortas) were separated (Figure 1A). The segments obtained from the AA&R and the DT aorta were digested separately at 37 °C in DMEM containing collagenase P, dispase, and DNaseI. The cell suspensions individually obtained from the AA&R and DT aortas were passed through a 70µm cell strainer and were stained with anti-mouse CD45-PE (Biolegend, clone 30-F11), LIVE/DEAD Fixable Near-IR Dead Cell Dye (Thermo Fisher, Waltham, MA, USA), and Hoechst 33342 (Thermo Fisher, Waltham, MA, USA) fluorescent dyes to exclude cell debris. CD45^+^ cells were then selected from the total viable AA&R and DT aorta cells using Beckman Coulter’s MoFlo Astrios EQ for scRNA-sequencing. The aortic roots of male *Apoe*^−/−^ Myh11-CreERT2, ROSA26 STOP-flox eYFP^+/+^ mice that were fed a NCD or HCD were embedded in OCT and were serially cut into 5 μm sections.

### 2.3. Single-Cell RNA-Sequencing

The total viable CD45^+^ cells from the AA&R and DT aortas were loaded separately on a C1 Single-Cell mRNA Seq HT IFC chip (10–17 μm) to automatically isolate individual cells to separate reaction chambers. After labelling each cell and using the microscopy imaging of the C1 chips to confirm the cell count of about a 70% capture efficiency, each cell was lysed for RNA amplification and cDNA synthesis using the C1™ Single-Cell mRNA Seq HT Reagent Kit (Fluidigm, South San Francisco (HQ), CA, USA). The Illumina Nextera XT DNA Sample Preparation Kit (Illumina San Diego, CA, USA) was used for library preparation. Ten million reads from individual cells were acquired for the relative quantitation of mRNA expressions on an Illumina sequencer.

### 2.4. scRNAseq Data Analysis

Demultiplexed FASTQ pairs were generated, UMI-tools were used to assign cell and UMI barcodes to each read, and reads were trimmed using Cutadapt [15]. Quality scores were assessed using FastQC [16]. Reads were aligned to the Mus musculus genome build mm10 using STAR [17]. Individual sample reads were quantified using HTseq [18]. Alignments were deduplicated with UMI-tools on the gene level, and such reads were grouped together if they shared the same UMI and gene. Cells with more than 1.5% of reads that aligned to mitochondrial-encoded genes were discarded. Resulting counts were analyzed using Seurat 3.1.1. Counts were normalized using ‘SCTransform’ and the analysis used 10 and 7 dimensions, respectively, for the ‘FindNeighbors’ functions. Clusters were visualized using t-SNE. The ROSALIND™ RNA-seq assay was used (https://rosalind.onramp.bio/ accessed on 20 January 2020), with HyperScale architecture developed by OnRamp BioInformatics, Inc. (San Diego, CA, USA). Reads were trimmed using Cutadapt [15]. Quality scores were assessed using FastQC [16]. Individual sample reads were quantified using HTseq [18] and were normalized via relative log expression (RLE) using the DESeq2 R library. Read distribution percentages, violin plots, identity heatmaps, and sample MDS plots were generated as part of the QC step using RSeQC [19]. DEseq2 was also used to calculate fold changes and *p*-values. The clustering of genes for the final heatmap of the differentially expressed genes was done using the PAM (partitioning around medoids) method using the fpc R library [20]. The functional enrichment analysis of the pathways, gene ontology, domain structure, and other ontologies was performed using HOMER [21]. Several database sources were referenced for the enrichment analysis, including Interpro [22], NCBI [23], KEGG [24,25,26], MSigDB [27,28], REACTOME [29], and WikiPathways [30]. The enrichment was calculated relative to a set of background genes relevant for the experiment. The additional gene enrichment is available from the following partner institution: Advaita (http://www.advaitabio.com/ipathwayguide, accessed on 15 November 2019) [31,32].

### 2.5. Human Samples

The specimens of the internal carotid plaques of a previously published cohort study [33] of symptomatic patients with CAD and their first episode of an ipsilateral ischemic stroke (an ipsilateral focal neurological deficit of an acute onset lasting >24 h), as well as specimens of asymptomatic patients (no history of ischemic symptoms) undergoing an endarterectomy for severe carotid stenosis were used for immunofluorescent analyses. A carotid endarterectomy (CEA) was performed due to an extra cranial high-grade internal carotid stenosis (>70% luminal narrowing) in symptomatic and asymptomatic patients. US Doppler echography and angiographic confirmation, using the criteria of the North American Symptomatic Carotid Endarterectomy Trial (NASCET) [34], was applied to determine the degree of luminal narrowing. The indication of the CEA for asymptomatic patients was based on the recommendations of the Asymptomatic Carotid Surgery Trial (ACST) [35], while for symptomatic patients, the CEA indication followed the recommendations of the European Carotid Surgery Trial (ECST) [36] and the North American Symptomatic Carotid Endarterectomy Trial (NASCET) [36]. After surgical excision, the internal carotid plaque specimens were cut perpendicular to the long axis through the point of maximum stenosis to obtain the atherosclerotic plaque upstream to the blood flow. The upstream internal carotid plaque specimens from symptomatic and asymptomatic patients were embedded in an optimal cutting temperature (OCT) compound. The study was approved by the Medical Ethics Committee of San Martino Hospital in Genoa (Italy) and were conducted in compliance with the Declaration of Helsinki after participants provided written informed consent.

### 2.6. The Multiplex Immunoassay

Mouse serum samples of *Apoe*^−/−^ C57Bl/6 mice on NCD or HCD were centrifuged, and the Bio-Plex Pro Mouse Chemokine Eotaxin-2/CCL24 Set #12002236, including coupled magnetic beads and detection antibodies for detecting mouse Eotaxin-2/CCL24, was used in combination with the Bio-Plex Pro Reagent Kit V (12002798) and the Bio-Plex Pro Mouse Chemokine Standards (12002796) (Bio-Rad, Hercules, CA, USA). The assay procedure was performed according to the manual instructions of the Bio-Plex Pro Mouse Chemokine Eotaxin-2/CCL24 assay. Results were obtained by a calibrated Luminex Instrument (Luminex Corporation). The absolute quantification was performed with xPONENT^®^ 4.2 for MAGPIX^®^ (Luminex Corporation, Austin, TX, USA).

### 2.7. CCL24 Human ELISA

The human serum of patients undergoing an endarterectomy for severe carotid stenosis was used for the quantification of systemic levels of CCL24. The human serum was obtained from patients of a previously published cohort study [33] containing symptomatic patients with CAD and their first episode of an ipsilateral ischemic stroke (an ipsilateral focal neurological deficit of acute onset lasting >24 h), as well as asymptomatic patients (no history of ischemic symptoms) undergoing an endarterectomy for severe carotid stenosis. Human CCL24 serum levels were quantified (diluted 1:4, total volume 100 µL) using Human CCL24 ELISA (R&D). The assay procedure was performed according to the manual instructions.

### 2.8. Oil Red O Staining for Lipid Content

Five sections of the aortic roots and abdominal aorta, per mouse, were stained with Oil Red O, as previously described [37,38]. Sections and aortas were counter-stained with Mayer’s hemalum solution and were rinsed in distilled water. The quantification was performed using the MetaMorph software. Data were calculated as the percentage of the stained area from the total lesion area.

### 2.9. Immunohistochemistry

The mouse aortic sinus was serially cut in 5 μm transversal sections, as previously described [37,38]. Sections from mouse specimens were fixed in acetone and immunostained with specific anti-mouse CD68 (Serotec, Puchheim, Germany) staining in the atherosclerotic roots. The quantification was performed using the MetaMorph or Definiens software. Results were expressed as a percentage of the stained area from the total lesion area. Sections of the aortas of *Apoe*^−/−^ Myh11-CreERT2, ROSA26, STOP-flox eYFP^+/+^ mice on NCD and HCD were embedded in OCT and serially cut into 7 μm sections and stained with Oil Red O for lipid content quantification, as previously described [39]. Alternatively, the sections were fixed in acetone and immunostained with a specific anti-mouse CD68 (Serotec, Puchheim, Germany). The sections were counter-stained with Mayer’s hemalum solution and rinsed in distilled water. The quantification was performed using the Definiens Tissue Studio software (Definiens Inc., Munich, Germany). Data were calculated as the percentage of the stained area from the total lesion area. To detect calcium, the sections of the aortas of *Apoe*^−/−^ Myh11-CreERT2, ROSA26, STOP-flox eYFP^+/+^ mice on NCD and HCD were embedded in OCT and were serially cut into 7 μm sections, rehydrated in water for 2 min, stained in 40 nM of alizarin red staining solution (Sigma Aldrich, St. Louis, MO, USA) with a pH of 4.2 for 6 min, rinsed in distilled water followed by 3 changes of phosphate-buffered saline with a pH of 7.4, and rinsed in Neoclear (VWR International, Radnor, PA, USA) 2 times. The slides were then air-dried and mounted in Neo-Mount (VWR International). The quantification was performed using the Definiens Tissue Studio software (Definiens Inc.), or QuPath open-source software, for a whole slide image analysis.

### 2.10. Immunofluorescent Staining and Quantification

Internal carotid plaque specimens from symptomatic and asymptomatic patients, as well as the aortic roots of male *Apoe*^−/−^ Myh11-CreERT2, ROSA26 STOP-flox eYFP^+/+^ mice on NCD and HCD, were embedded in OCT and were serially cut into 5 μm sections. Cryosections were fixed in 1% paraformaldehyde, washed with 1xPBS, incubated with a blocking solution consisting of 5% BSA in PBS for 30 min, then permeabilized with Triton X-100 at 0.1%. Endarterectomy specimens were stained with a primary rat anti-LYVE1 (R&D Systems, Minneapolis, MN, USA), an anti-Myh11 (Thermo Fischer, Waltham, MA, USA), and a goat anti-CCL24 in a blocking solution. Microcalcification was detected by using a bisphosphonate-conjugated imaging agent that binds to hydroxyapatite (OsteoSense 680, PerkinElmer, Waltham, MA, USA), elaborating fluorescence that was evident though the near-infrared window (ex/em 650/680 nm). Briefly, frozen sections were treated with OsteoSense 680 (1:100) overnight at 4 °C. After washing, the samples were incubated with the following secondary antibodies: PE anti-rat (Thermo Fischer), Alexa 350 anti-mouse (Thermo Fischer), or anti-goat AF647, and were mounted with a ProLong Glass Antifade Mountant (Thermo Fischer). Immunofluorescent images were acquired with Axioscan Z1 microscopy and were analyzed and quantified with the QuPath software platform for the whole slide image analysis. The aortic root cryosections of *Apoe*^−/−^ Myh11-CreERT2, ROSA26 STOP-flox eYFP^+/+^ mice were stained with a primary rabbit anti-GDF10 (Thermo Fischer), RUNX2 (Novusbio, Littleton, CO, USA), and a cell-permeant SYTO orange fluorescent nucleic acid stain (Thermo Fischer) in a blocking solution. After washing, the samples were incubated with the following secondary antibodies: the Alexa 647 anti-rabbit (Thermo Fischer) or the DyLight 405 and were mounted with the ProLong Glass Antifade Mountant (Thermo Fischer). Immunofluorescent images were acquired with Axioscan Z1 microscopy and were analyzed and quantified with the QuPath software platform for the whole slide image analysis.

### 2.11. Tissue Processing, Flow Cytometry, and Cell Sorting

*Apoe*^−/−^ Myh11-CreERT2, ROSA26 STOP-flox eYFP^+/+^ mice were subject to NCD and HCD, as described previously. The aorta adventitia was carefully excised by a sharp surgical dissection in a clearly defined plane to leave a naked media over the length of the AA&R (the aortal segment from just left of the branchpoint for the brachiocephalic artery to just right of the branchpoint for the left subclavian artery) and abdominal aorta (the straight aortal segment after the renal aortas). The segments obtained from the AA&R and the abdominal aorta were digested separately at 37 °C in DMEM containing collagenase P, dispase, and DNaseI. For aorta samples, two aortas were pooled into one sample to get sufficient cell numbers. Cells were stained with fluorochrome-conjugated antibodies specific to mouse CD45, CD11b, LYVE-1, F4/80, CD64, and CD163 while Hoechts 33342 and Draq7 were used to exclude the dead cells. Cell sorting was performed using Beckman Coulter’s MoFlo Astrios EQ. LYVE-1^+^ macrophages were identified and sorted as CD45^+^CD11b^+^CD64^+^CD163^+^F4/80^+^ with >98% purity. The aorta adventitia was carefully excised by a sharp surgical dissection in a clearly defined plane, to leave naked media over the length of AA&R derived from male C57Bl/6 mice and *Apoe*^−/−^ C57Bl/6 mice. The VSMCs were isolated from the AA&R via digestion at 37 °C in DMEM containing collagenase P, dispase, and DNaseI. The VSMC phenotype was confirmed by a flowcytometry analysis for a smooth muscle α-actin, a Myh11 positive expression, and CD31 (endothelial cell marker) and CD90 (fibroblast cell marker) negative expressions. These cells were cultured at a density of 3 × 10^4^ cells/cm^2^ using a SmBMTM Basal Medium (CC-3181, Lonza, Basel, Switzerland), and SmGMTM-2 SingleQuotsTM supplements (CC-4149, Lonza) were required for the growth of the VSMCs for 3 weeks. The medium was renewed every 3 days.

### 2.12. The Quantification of VSMC Osteoblast-like Cell Transdifferentiation

The quantification of VSMC osteoblast-like cell transdifferentiation was performed using VSMC derived from the AA&R of male C57Bl/6 mice. In vitro VSMCs were stimulated with either 40ng/mL oxLDL or were co-cultured with LYVE1 macrophages derived from *Apoe*^−/−^ Myh11-CreERT2, ROSA26 STOP-flox eYFP^+/+^ mice on HCD using Transwell cell culture inserts for 7 days. The quantification of VSMC osteoblast-like cell transdifferentiation was performed via a flow cytometry analysis of RUNX2 DyLight 405 (NOVUS), alkaline phosphatase APC (NOVUS), osteopontin PE (R&D), collagen 8 and Mac-2 PE/Cyanine7 (Biolegend, San Diego, CA, USA), and Brilliant Violet 650™ anti-mouse F4/80 after excluding dead cells via LIVE/DEAD Fixable Near-IR Dead Cell Dye staining (Thermo Fisher). Samples were acquired in CytoFLEX (Beckman Coulter) and were analyzed with FlowJo software (TreeStar, Version 10.5.3, Woodburn, OR, USA).

### 2.13. Statistical Analyses

Statistical analyses were performed in GraphPad Prism 8 for Mac OS X (GraphPad Software, Inc., La Jolla, CA, USA). All data sets were tested for a normal distribution with normality tests before proceeding with parametric or non-parametric analyses. Grubb’s test was performed in order to exclude spurious outliers. Statistical significance was tested using an unpaired *t*-test, a one-way analysis of variance (ANOVA) with a Tukey post-hoc test, and a two-way ANOVA with a Bonferroni post-hoc test for data sets with normal distributions. Statistical significance was tested with the Mann–Whitney U test and a one-way ANOVA with the Dunn’s post-hoc test for data sets without a normal distribution, with a significance threshold of *p* ≤ 0.05. A linear regression analysis was performed to compare the Alizarin red positive staining to the percentage of LYVE1^+^ CCL24^+^ cells, or CCL24 levels, in the serum.

## 3. Results

### 3.1. The Site-Specific Development of Atherosclerosis Defines the Hypercholesteremia-Associated Transcriptional Signature

The site-specific development of atherosclerotic lesions are observed in both murine models of atherosclerosis and in humans [40]. *Apoe*^−/−^ mice exhibit lesion formation predominantly in the aortic arch and root (AA&R) and the abdominal aorta [40]. Regarding the side-specific predisposition to atherosclerosis, we undertook a precise approach to reveal disease-associated cell populations, genes, and molecular determinants by performing the scRNAseq of the atherosclerosis-prone sites in AA&R separately from the more atherosclerosis-resistant descending thoracic aorta (DT). The adventitia was carefully excised by a sharp surgical dissection in a clearly defined plane, to leave a naked media. The Fluidigm C1 platform for RNA sequencing of single cells was used to reveal the transcriptional profiles of viable individual CD45^+^ aorta cells isolated from the AA&R and DT aortas of *Apoe*^−/−^ mice, fed either an NCD for 16 weeks or an HCD for 11 weeks (Figure 1A). CD45^+^ cells were selected after the exclusion of positive cells for LIVE/DEAD Fixable Near-IR Dead Cell Dye and the selection of Hoechst 33342 positive cells to exclude dead cells and cell debris, as shown in Figure 1A. Cells with more than 1.5% of their reads aligning to mitochondrial-encoded genes were discarded. As a result, the scRNAseq profiles of 1059 cells passed the quality control (NCD (AA&R) 285 cells; NCD (DT) 279 cells; HCD (AA&R) 210 cells; HCD (DT) 285 cells) with their respective median genes per cell and with reads mapped to genes, as illustrated on Figure S1A,B. HCD-fed *Apoe*^−/−^ mice showed significantly elevated levels of cholesterol and LDL-C (Figure S2A,B), as well as larger atherosclerotic lesions in the aortic roots and abdominal aortas in comparison with NCD-fed *Apoe*^−/−^ mice (Figure 1B,C and Figure S2C). In contrast, atherosclerotic plaques were not observed in the DT aortas, even when the *Apoe*^−/−^ mice were fed HCD (Figure 1D). To better explain the broader mechanisms that control the initiation and progression of atherosclerosis, we investigated how hypercholesteremia impacts the gene expression profile, specifically in atheroprone AA&R. A GO term enrichment analysis comparing HCD with NCD, specifically with the AA&R-derived cells of *Apoe*^−/−^ mice (Figure 1E), showed an induction of gene sets that were related to leukocyte migration and chemotaxis, cytokine production, the positive regulation of the inflammatory response, the regulation of leukocyte proliferation, the acute inflammatory response, the positive regulation of lipid localization, macrophage chemotaxis, and the regulation of complement activations all in line with hypercholesteremia immune cell activation. However, atheroprotective mechanisms, such as the negative regulation of the immune system process and the regulation of the immune effector process were also triggered in the AA&R-derived cells of *Apoe*^−/−^ mice that were fed HCD (Figure 1E). Interestingly, among the gene sets revealed by the GO term enrichment analysis of the cells derived from the DT aortas of *Apoe*^−/−^ mice on HCD versus NCD, there were genes involved in T-cell activation, the positive regulation of defense response, the regulation of the immune effector process, the positive regulation of cytokine production, and the regulation of protein serine/threonine kinase activity, which could have an important implications for the regulation of the T-cell response [41] (Figure 1F). Furthermore, triggered gene sets were involved in the regulation of the inflammatory response, the positive regulation of the response to an external stimulus, leukocyte cell-to-cell adhesion and proliferation, cell chemotaxis, the regulation of T-cell activation, and the regulation of the innate immune response (Figure 1F). The mechanisms of regulation that were triggered in the DT aorta-derived cells in response to HCD showed the potential mechanisms of atheroprotection that potentially contribute to the atheroresistance of the DT aorta to hypercholesteremia-mediated atherogenic stress.

### 3.2. Atherosclerosis-Associated Immune Cell Populations Revealed by scRNAseq

**Figure 2:**
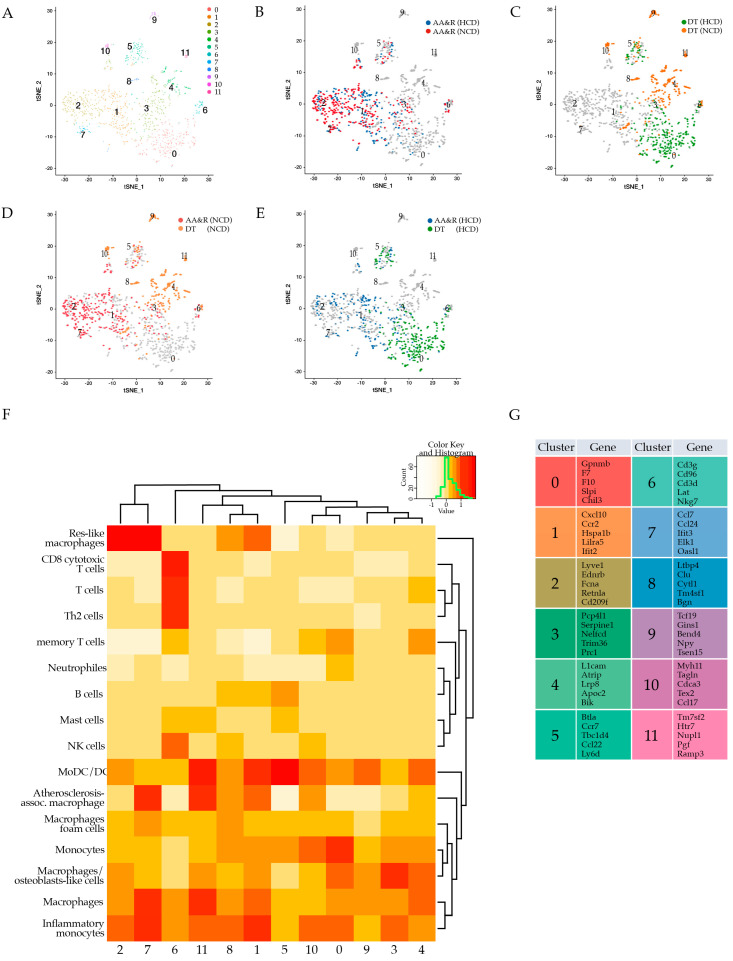
Clusters of AA&R and DT aorta CD45^+^ cells of *Apoe*^−/−^ on NCD and HCD; *t*-distributed stochastic neighbor embedding (tSNE) plot showing (**A**) all twelve identified clusters; (**B**) AA&R aorta-derived clusters of *Apoe*^−/−^ mice on NCD versus HCD; (**C**) DT aorta-derived clusters of *Apoe*^−/−^ mice on NCD versus HCD; (**D**) clusters of AA&R and DT aorta-derived cells of *Apoe*^−/−^ mice on NCD; (**E**) clusters of AA&R and DT aorta-derived cells of *Apoe*^−/−^ mice on HCD; (**F**) Heat map of the gene expression profile of 12 immune cell populations showing their immune cell population identity; (**G**) top 5 differentially expressed genes detected in each cluster.

To characterize, in an unbiased manner, the gene expression profile of the total viable CD45^+^ leukocytes specifically derived from the atherosclerosis-prone AA&R, and separately, from the more atheroresistant DT aortas of *Apoe*^−/−^ mice fed either NCD for 16 weeks or HCD for 11 weeks, representing more advanced stages of lesion development, we used a screening approach of single cell transcriptomics. An unsupervised clustering algorithm was applied to investigate the aortic cell phenotypic diversity which revealed 12 distinct cell clusters (Figure 2A), typical for either the athero-prone AA&R or the more athero-resistant DT aortas of *Apoe*^−/−^ mice (Figure 2B–E and Figure S2D). The identified clusters of CD45^+^ cells showed a gene expression pattern of established canonical markers of various lymphocyte lineages (*Cd3d*, *Nkg7*, and *Cd8b1*) and myeloid cells (*Itgax* encoding CD11c, *Cd14*, *Csf1r*, *Lgals3*, *Ccr2*, *Cd68*, *Ly6c2*, and *Cd209a,* Supplementary Table S1). Clusters 1, 2, and 7 were composed of CD45^+^ cells derived almost exclusively from the AA&R cells of *Apoe*^−/−^ mice on NCD or HCD (Figure S2D) which expressed gene profiles of inflammatory monocytes/macrophages and, particularly, of resident and inflammatory macrophages (Figure S3). Clusters 0, 4, 9, 10, and 11 were predominantly or exclusively composed of cells derived from the DT aortas (Figure S2D).

Collectively, this result provides the first step towards the ambitious goal of building a comprehensive single-cell atlas illustrating the side-specific predisposition to atherosclerosis. Single-cell transcriptional analyses enables the exploration of the immune system in the diseased vasculature, providing an overview of the complex side-specific cellular and molecular alterations.

### 3.3. Atherosclerosis Cluster Gene Expression Signatures

Cluster 0 showed a gene expression profile typical for monocytes with a differential expression of *Gpnmb* [42] and Slpi, shown to attenuate inflammatory cytokine production [43] (Figure 2F,G). The GO term enrichment analysis of cluster 0 showed the involvement of the identified cluster of monocytes/macrophages in the negative regulation of cell adhesion, wound healing, leukocyte chemotaxis, the negative regulation of cytokine production, and the regulation of cytokine secretion and blood coagulation (Figure S4A). Cluster 1, consisting of cells mainly derived from the AA&R, expressed a set of genes corresponding to a population of monocyte-derived dendritic cells (MoDC/DC) with a pro-inflammatory phenotype (Figure 2F,G) as shown by the high expression of Cxcl10, Ccr2, Hspa1b, Lilra5, and Ifit2 (Figure 2G and Figure S3). In line with the observed gene expression profile of cluster 1, the GO term enrichment analysis revealed signaling pathways linked to T-cell activation, the positive regulation of cell adhesion, cytokine production, cell-to-cell adhesion, and leukocyte migration, as well as antigen processing and presentation via MHC class II (Figure S4B). This indicates that cluster 1 is an important population involved in antigen presentation and T-cell activation in the atherosclerotic plaques [44]. Cluster 2 exhibited a gene expression profile of macrophages as shown by the expression of Ednrb, Fcna, Retnl, and Cd209f, as well as genes that are typical for resident macrophages (Lyve1 and FCGR1A (CD64)) [5] (Figure 2G and Figure S3). Cluster 3 emerges as a population of macrophages characterized by the expression of Pcp4l1, Serpine1, Nelfcd, Trim36, Prc1, and Lgals3 (Galectin-3, also known as Mac-2) (Figure 2G and Figure S3). The GO term enrichment analysis of cluster 3 showed a positive regulation of protein kinase activity and a negative regulation of cell migration, motility, and locomotion, as well as a positive regulation of MAP kinase activity and a negative regulation of blood coagulation and platelet activation, probably linked to an important role in coagulation (Figure S4C). Cluster 4 was present only in the DT aortas of *Apoe*^−/−^ mice on NCD and showed macrophages with a Mo/DC gene expression profile (Figure 2F,G). Interestingly, one of the differentially expressed genes of cluster 4 was Apoc2, which has an important role in atheroprotection since it activates lipoprotein lipase, the main enzyme that hydrolyses plasma triglycerides [45] (Figure 2G and Figure S3). Cluster 5 showed a gene expression profile that was characterized by the expression of Btla, Ccr7, Tbc1d4, Ccl22, and Ly6d (Figure 2G and Figure S3) revealing a population of MoDC/DC cells (Figure 2F,G) present in the aortas that were independent of the atherosclerosis predisposition or the diet (Figure S2D). The GO term analysis of cluster 5 confirmed the involvement of this cluster in antigen processing and presentation, with a role in the negative regulation of immune system processes, T-cell activation, and co-stimulation, as well as the negative regulation of B-cell activation (Figure S4D). The gene expression profile of cluster 6 clearly characterized a T-cell population as shown by the expression of Cd3g, Cd96, and Cd3d, as well as Lat, which is critical for T-cell activation and development [46], and NKG7, which is essential for the cytotoxic degranulation of CD8^+^ T-cell and CD4^+^ T-cell activation and inflammation [47] (Figure 2F,G and Figure S3). The GO term enrichment analysis, in line with the T-cell identity of cluster 6, showed T-cell receptor signaling pathway activation, lymphocyte-mediated immunity, and the positive regulation of cytokine production, particularly of IFN-γ (Figure S4E). Cluster 7 predominantly composed of cells derived from the AA&R exhibited a gene expression profile of macrophages as evident by a high level of Ccl7 and Ifit3, found on M1 polarized macrophages, in addition to Elk1, a gene linked to severe inflammation [48] (Figure 2F,G, Figures S2D and S3). The GO term enrichment analysis of cluster 7 showed a positive regulation of cytokine production and a positive response to pro-inflammatory cytokines, in addition to ERK1/2 signaling cascade activation, implicated in many pathological conditions associated with chronic inflammation (Figure S4F). Cluster 8, predominantly derived from cells in the DT aorta in NCD (Figure S2D), emerged as a population showing a gene expression profile characterized by the expression of Ltbp4 an essential regulator of TGFβ signaling, which is related to development, immunity, injury repair, and diseases, as well as playing a central role in regulating inflammation and fibrosis [49] (Figure 2G). Furthermore, cluster 8 also expressed genes like Clu (clusterin) with a role in complement inhibition, inflammation regulation, lipid transport, apoptosis, and cell differentiation [50]. In addition, Oasl1 gene shown to negatively regulate the production of type I interferon via inhibition of the master transcription factor IRF7 [51] was upregulated in cluster 8 (Figure 2G). Dendritic cells (DCs) and macrophages are thought to perform overlapping functions in atherosclerosis, and the distinction of bona fide DCs compared to macrophages in lesions is an unresolved issue in atherosclerosis research [52]. Clusters 9, 10, and 11, composed from cells in the DT aorta, showed a gene expression profile of MoDC/DC (Figure 2F, Figures S3 and S4G–I). The identified atherosclerosis-associated immune cell cluster, particularly plaque macrophages, was comprised of highly heterogeneous subsets with a role in the exacerbation of inflammation, lipid transport, antigen presentation, and T-cell activation, as well as the negative regulation of inflammation.

### 3.4. Res-like Macrophages Populated the Aortic Arch and Roots in Atherosclerosis

**Figure 3:**
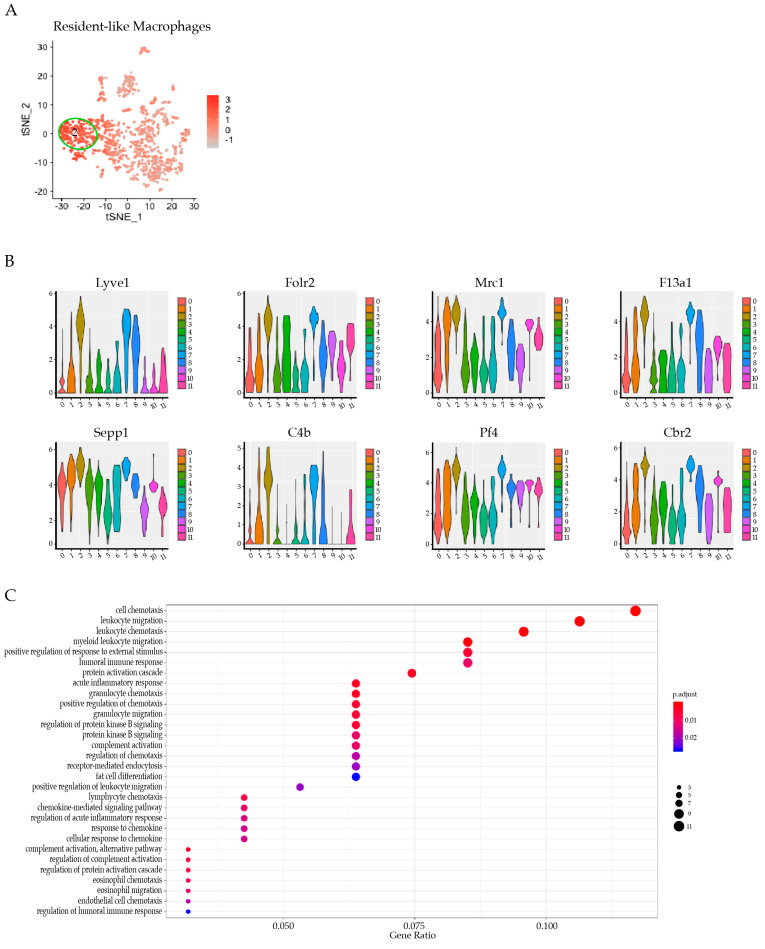
Cluster 2 gene expression signature. Gene sets were overlaid on single cells on a tSNE plot to identify the cell identity of cluster 2 with an enrichment of indicated gene sets: (**A**) Resident-like Macrophages; and (**B**) top eight expressed genes of cluster 2. (**C**) Bubble plot GO term enrichment analysis of cells of cluster 2. Dot size is proportional to the number of genes overlapping with each GO term, while the adjusted *p*-value is color-coded from red to blue.

As expected, the percentage of CD68 positive cells increased significantly in the aortic roots in response to HCD, in comparison to *Apoe*^−/−^ mice on NCD (Figure S5A,B). Cluster 2 emerged as a population of particular interest since it was derived from cells specifically from the atherosclerosis-prone AA&R of *Apoe*^−/−^ mice on either NCD or HCD (Figure S2D). Notably, the gene expression analysis of cluster 2 revealed a resident-like macrophage gene expression profile (Figure 3A). In accordance with this, cluster 2 showed a high expression of *Lyve1* and *F13a1,* known as resident macrophage markers [53,54]. Interestingly, cluster 2 expressed *CD163,* which is typical for a subtype of alternatively activated macrophages called Mhem/M (Hb) [55] and were first described in areas with intraplaque hemorrhages [56]. Furthermore, CD163 positive macrophages have been found in vulnerable human carotid plaques, which support the notion that *CD163* expression could contribute to clinical events [55]. Cluster 2 also expressed *Rcn3* (Figure 2G), which is emerging as a new potential negative regulator of collagen production [57] and genes typically expressed in M2-like macrophages, such as *Folr2*, *Cbr2,* and *Mrc1,* also known as *Cd206* [2], as well as *F13A1* expressed by alternatively activated macrophages [58], *Selenoprotein 1* (sepp1) [59], *Pf4* encoding CXCL4, and the inflammatory gene *Ednrb* [60] (Figure 3B). The GO term enrichment analysis of cluster 2 revealed an upregulation of gene sets involved in leukocyte and lymphocyte migration/chemotaxis and the regulation of the humoral immune response, the acute inflammatory response, and complement activation (Figure 3C).

### 3.5. LYVE-1 Res-like Macrophages Expanded in Murine Atherosclerotic Lesions

**Figure 4:**
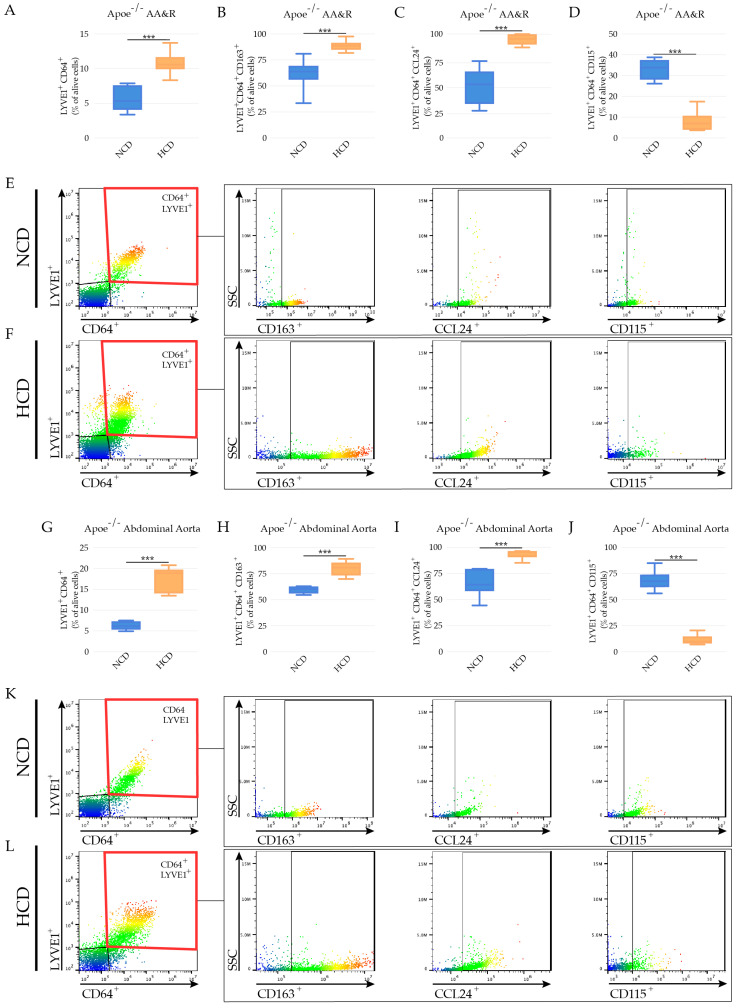
LYVE-1 res-like macrophages in mouse atherosclerotic plaques. Bar graphs represent the mean ± SEM of the percentage of LYVE1 res-like macrophages in AA&R of *Apoe*^−/−^ mice on NCD versus HCD, expressed as percentage of the total live plaque cells, as defined by (**A**) LYVE1^+^CD64^+^; (**B**) LYVE1^+^CD64^+^CD163^+^; (**C**) LYVE1^+^CD64^+^CCL24^+^; and (**D**) LYVE1^+^CD64^+^CD115^+^, with an expression of *n* = 8 mice per group and *** *p* < 0.001. Representative dot plots show the gating strategy illustrating LYVE1^+^ res-like macrophage marker expression in AA&R of *Apoe*^−/−^ mice on (**E**) NCD and (**F**) HCD. Bar graphs represent the mean ± SEM of the percentage of LYVE1 res-like macrophages in abdominal aortas of *Apoe*^−/−^ mice on NCD versus HCD, expressed as percentage of the total live plaque cells, as defined by (**G**) LYVE1^+^CD64^+^; (**H**) LYVE1^+^CD64^+^CD163^+^; (**I**) LYVE1^+^CD64^+^CCL24^+^; and (**J**) LYVE1^+^CD64^+^CD115^+^, with an expression of *n* = 8 mice per group and *** *p* < 0.001. Representative dot plots illustrating the gating strategy show the LYVE1^+^ res-like macrophage marker expression in abdominal aortas of *Apoe*^−/−^ mice on (**K**) NCD and (**L**) HCD.

Flow cytometric analyses, after the exclusion of doublets, LIVE/DEAD Fixable Near-IR Dead Cell Dye positive cells, and a selection of Hoechst 33342 positive cells (Figure S5D) revealed a raise in LYVE-1^+^CD64^+^ aortic macrophages in the atherosclerosis-prone aortic arch (Figure 4A,E,F) and abdominal aortas of *Apoe*^−/−^ mice (Figure 4G,K,L) in response to hypercholesteremia. As revealed by scRNAseq, LYVE-1^+^CD64^+^ macrophages expressed CD163 and CCL24 in the aortic arch (Figure 4B,C,E,F) and abdominal aortas of *Apoe*^−/−^ mice, with a higher expression in *Apoe*^−/−^ mice on HCD (Figure 4H,I,K,L). Interestingly, the hypercholesteremia in *Apoe*^−/−^ mice promoted the downregulation of CD115 expressions (colony-stimulating factor-1 receptor (CSF-1R) in LYVE-1^+^CD64^+^ macrophages (Figure 4D,J)). CD115 activation has been shown to be critical for the survival, proliferation, and differentiation of tissue macrophages through the activation of the CSF-1 receptor (CSF-1R) [61].

### 3.6. LYVE-1 Res-like Macrophages Promoted a VSMC Phenotypic Switch

**Figure 5:**
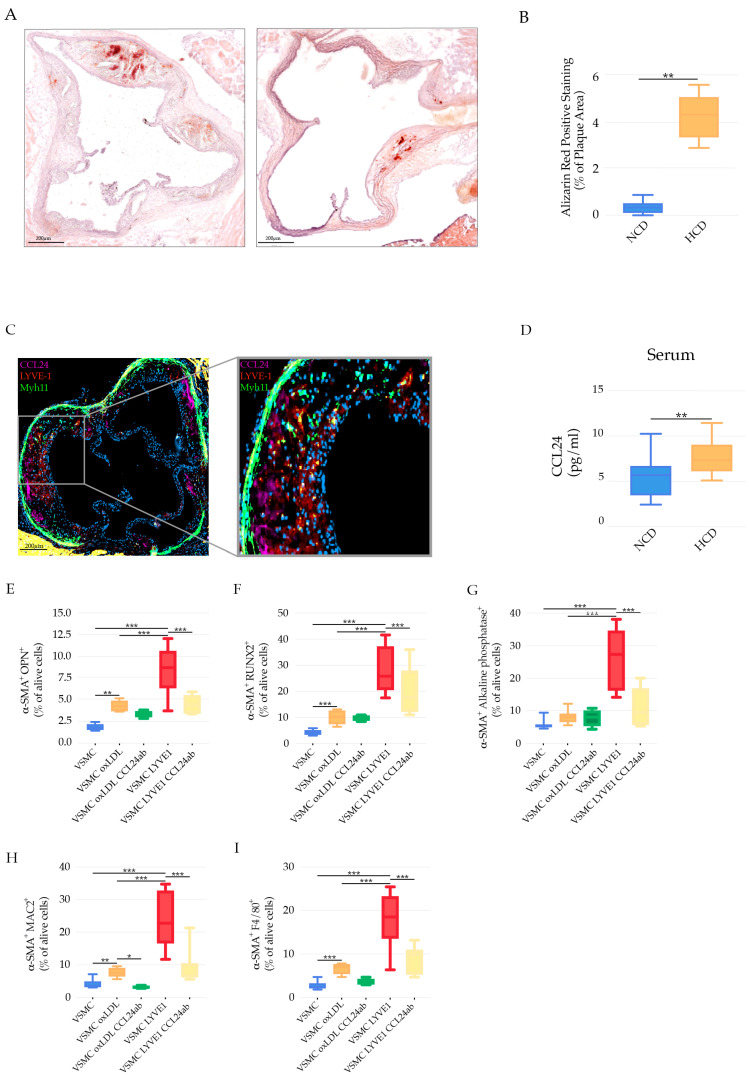
The role of LYVE-1 res-like macrophages in vascular calcification. (**A**) Representative images of vascular calcification illustrated that Alizarin red positively stained areas in aortic root cryosections of *Apoe*^−/−^ Myh11-CreERT2, ROSA26STOP-flox eYFP^+/+^ mice fed NCD or HCD. Scale bars: 200 μm. (**B**) Bar graphs represent the mean ± SEM of the percentage of Alizarin red positively stained areas in the total atherosclerotic plaque in aortic root cryosections of *Apoe*^−/−^ Myh11-CreERT2, ROSA26STOP-flox eYFP^+/+^ mice fed NCD or HCD, *n* = 8 and ** *p* < 0.01. (**C**) Representative immunofluorescence staining showing LYVE1^+^CCL24^+^ cells in close proximity to VSMC (Myh11 positive cells) in atherosclerotic lesions of *Apoe*^−/−^ mice on HCD; Myh11 (green), LYVE1 (red), and CCL24 (purple). (**D**) Bar graphs represent the mean ± SEM of systemic levels of CCL24 in *Apoe*^−/−^ mice on NCD and HCD, *n* = 8 mice per group and ** *p* < 0.01. Bar graph representing the mean ± SEM of (**E**) α-SMA^+^OPN^+^; (**F**) α-SMA^+^RUNX2^+^; (**G**) α-SMA^+^ alkaline phosphatase^+^; (**H**) α-SMA^+^MAC2^+^; (**I**) α-SMA^+^F4/80^+^ expression in WT VSMC post-stimulation with oxLDL or co-cultured with LYVE-1 res-like macrophages in the presence or absence of CCL24 neutralizing antibodies, *n* = 6 and *** *p* < 0.001.

Atherosclerosis and vascular calcification remain the leading cause of death worldwide and there is a huge need to investigate mediators of the osteogenic differentiation of VSMC as a major pathological process. The progress in this area is dependent on targeting cellular and soluble mediators promoting vascular calcification. LYVE-1 macrophages have been shown to regulate arterial stiffness by controlling collagen expression in VSMCs [5] and they express a high level of CCL24 (eotaxin-2) (Figure 2G), a chemokine shown to promote atherosclerosis damage [62] and VSMC calcification [63]. We hypothesized that LYVE-1 macrophages, via CCL24, could have a causal role in VSMC osteogenic differentiation. Calcification was observed in the aortic root cryosections of *Apoe*^−/−^ Myh11-CreERT2, ROSA26STOP-flox eYFP^+/+^ mice fed NCD or HCD (Figure 5A,B), with an increase in calcium deposits in response to hypercholesteremia (Figure 5B).

Using these mice, we demonstrated the spatial distribution of LYVE-1^+^CCL24^+^ macrophages within the arterial layers and their proximity to VSMCs. Using immunofluorescence, LYVE-1^+^CCL24^+^ macrophages were shown to accumulate in the vicinity of VSMCs (yellow fluorescent protein (eYFP) positive cells) (Figure 5C). In line with the rise in the number of LYVE-1^+^CCL24^+^ macrophages in response to HCD in *Apoe*^−/−^ mice (Figure 4), the circulating level of CCL24 was significantly elevated in *Apoe*^−/−^ mice on HCD (Figure 5B). To reveal whether LYVE-1 macrophages could triggering VSMC transdifferentiation to osteoblast-chondrocyte and macrophage-like cells, we co-cultured LYVE-1 res-like macrophages with VSMCs derived from of male C57Bl/6 mice. Because of the limited number of LYVE-1 res-like macrophages derived from the AA&R after cell sorting, we additionally used macrophages sorted from the abdominal aortas of *Apoe*^−/−^ mice on HCD to investigate, ex vivo, the effect of LYVE-1 res-like macrophages on the VSMC phenotypic switch. LYVE-1 res-like macrophages were sorted for the positive expressions of CD45, CD64 (FcγR1) known as a tissue-resident macrophage marker [64], the macrophage pan markers F4/80 and CD11b, and the alternatively activated macrophage marker CD163 [55] expressed by macrophages of vulnerable plaques [55]. Upon the in vitro co-culture of VSMCs derived from the AA&R of male C57Bl/6 mice with sorted LYVE-1 res-like macrophages, we observed a pronounced increase in the expression of osteoblast/chondrocyte markers, such as OPN (osteopontin) (Figure 5E); RUNX2, an osteogenic transcription factor with an important role in VSMC calcification (Figure 5F) [65]; and alkaline phosphatase, which is an early indicator of VSMC osteogenic differentiation (Figure 5G), in comparison to VSMC stimulated with oxLDL alone. In addition, the expression of the macrophage markers Mac-2 and F4/80 in the VSMC post co-culture with LYVE-1 res-like macrophages was significantly increased, as demonstrated by flow cytometry analyses (Figure 5H,I). Importantly, the CCL24-neutralizing antibody abrogated LYVE-1 res-like macrophages, which mediated the induction of markers of VSMC osteogenic differentiation (Figure 5C–E) and the phenotypic switch to macrophage-like cells (Figure 5F,G). This result clearly showed that LYVE-1 res-like macrophages derived from *Apoe*^−/−^ mice on HCD promoted VSMC transdifferentiation to osteoblast-, chondrocyte-, and macrophage-like cells in a CCL24-dependent manner. In this regard, CCL24 expressed by LYVE-1 res-like macrophages may emerge as a potential inducer of the pro-calcific differentiation of VSMCs.

### 3.7. LYVE-1 Res-like Macrophage Accumulation was Associated with Plaque Ruptures in Human Carotid Artery Disease

**Figure 6:**
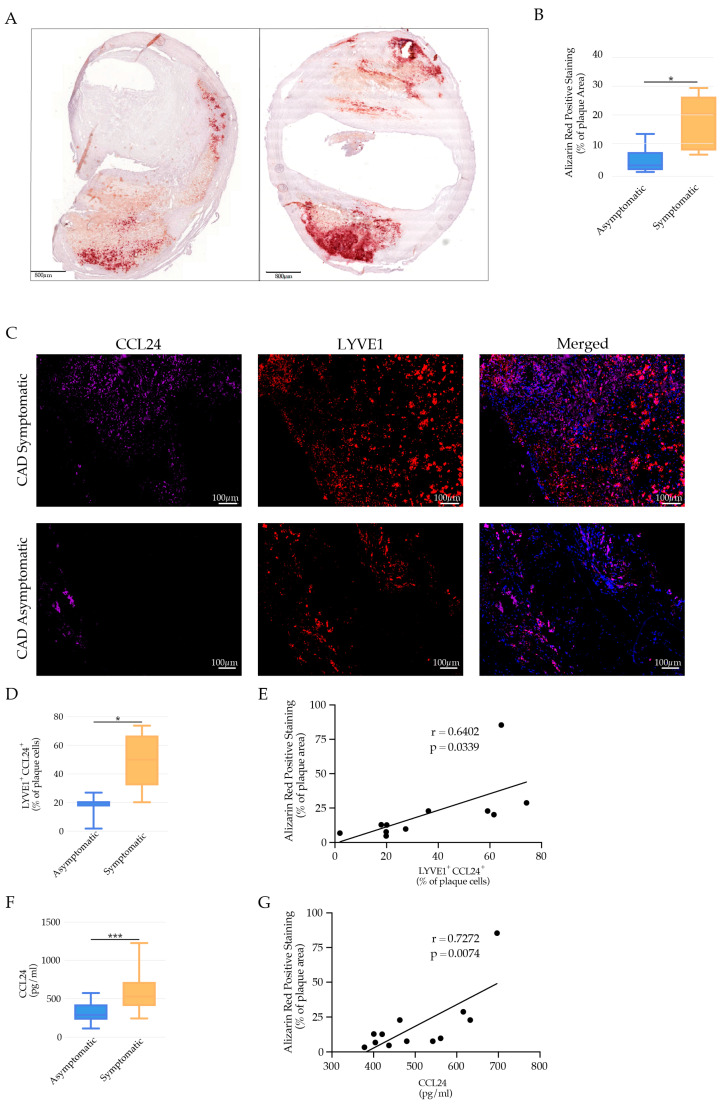
LYVE-1 res-like macrophages in human atherosclerotic plaques. (**A**) Representative images of vascular calcification illustrated by Alizarin red staining of human atherosclerotic plaques of asymptomatic and symptomatic CAD patients, respectively. Scale bars: 800 μm. (**B**) Bar graphs represent the mean ± SEM of the percentage of Alizarin red positively stained areas in the total atherosclerotic plaque area of asymptomatic and symptomatic CAD patients, respectively, *n* = 8 and * *p* < 0.05. (**C**) Representative immunofluorescence staining showing LYVE1^+^ cells (red) expressing CCL24^+^ (violet) in atherosclerotic lesions of asymptomatic and symptomatic CAD patients, respectively. (**D**) Bar graphs represent the mean ± SEM of LYVE1 cells expressing CCL24 in asymptomatic and symptomatic CAD patients, respectively, *n* = 8 and * *p* < 0.05. (**E**) Positive correlation of vascular calcification (percentage of positive Alizarin red stained areas) with percentage of LYVE1 cells expressing CCL24. (**F**) Bar graphs represent the mean ± SEM of systemic levels of CCL24^+^ in asymptomatic and symptomatic CAD patients, respectively, *n* = 8 and *** *p* < 0.001. (**G**) Positive correlation of vascular calcification (percentage of positive Alizarin red stained areas) with systemic levels of CCL24.

To gain insight into the possible role of LYVE-1 res-like macrophages in the destabilization of human atherosclerotic plaques, we used specimens of internal carotid plaques from a previously published cohort study [33] from symptomatic CAD patients with their first episode of an ipsilateral ischemic stroke, as well as specimens from asymptomatic patients (no history of ischemic symptoms) undergoing an endarterectomy for a severe carotid stenosis. We observed the pronounced calcification of human atherosclerotic lesions of CAD asymptomatic and symptomatic patients with an increased calcium deposit in symptomatic patients (Figure 6A,B). Furthermore, we found an accumulation of LYVE-1 res-like macrophages in close proximity to VSMC cells (Myh11, red) in areas of human atherosclerotic plaque micro-calcification (OsteoSense, purple staining) in symptomatic patients (Figure S5C). OsteoSense is indicative of advanced microcalcification that is localized preferentially with osteopontin-positive cells [66]. Furthermore, symptomatic patients, in comparison to asymptomatic patients, had a significant increase in the number of LYVE-1^+^CCL24^+^ macrophages (Figure 6C,D), which correlated positively with the extent of vascular calcification, as indicated by increased levels of Alizarin red positive staining (Figure 6E). Furthermore, symptomatic CAD patients exhibited a higher systemic level of CCL24 in comparison to asymptomatic patients (Figure 6F), which also correlated positively with the extent of vascular calcification (Figure 6G). These findings implied that the increased number of LYVE-1 res-like macrophages expressing CCL24, in addition to the elevated systemic serum levels of CCL24, could have a causal role in human atherosclerotic plaque destabilization. Taken together, our data implies the possible role of CCL24 in vascular calcification, with implications in human atherosclerotic plaque destabilization.

## 4. Discussion

Using scRNAseq, we revealed 12 distinct leukocyte populations specifically preset in the atherosclerosis-prone AA&R and the atherosclerosis-resistant DT aortas of *Apoe*^−/−^ mice in early and advanced stages of atherosclerosis. As previously shown, mouse plaques were mostly populated by macrophages, T-cells, monocytes, and MoDC/DC [67]. We found impressive atherosclerosis-associated diversity of macrophage populations in atherosclerotic aortas, expressing genes of resident-like macrophages, as well as atherosclerosis-associated populations expressing foam cell genes. Genes controlling antigen presentation were involved in the control of atherosclerosis-associated inflammation. Considering that one of the identified populations of macrophages was specifically derived from cells of the atherosclerosis-prone AA&R, we gained further insight into the role of LYVE-1 res-like macrophages in the pathogenesis of atherosclerosis. Importantly, the LYVE-1 res-like macrophage population expanded with the advancement of the lesion formation in *Apoe*^−/−^ mice in response to hypercholesteremia. Moreover, we found that LYVE-1 res-like macrophages exhibited pro-osteogenic action, triggering VSMC transdifferentiation into calcified VSMC osteoblast/chondrocyte-like cells via the release of CCL24 (eutaxin-2). We also showed an increased accumulation of LYVE-1^+^CCL24^+^ macrophages, particularly in human atherosclerotic plaques prone to rupture, as well as an association of elevated plasma levels of CCL24 with vascular calcification and an increased risk of carotid artery disease events in humans.

In response to hypercholesteremia, the gene expression profile was differently impacted depending on the side-specific predisposition to atherosclerosis, which has been observed in both murine and human models of atherosclerosis [40]. The AA&R-derived cells of *Apoe*^−/−^ showed an induction of gene sets related to the activation of an inflammatory response typical for hypercholesteremia-associated immune cell activation. However, atheroprotective mechanisms, such as the negative regulation of immune system processes, were also triggered in an attempt to counteract the inflammation. Cells derived from the DT aortas of *Apoe*^−/−^ mice on HCD, compared to NCD, showed an expression of gene sets that are important in the regulation of the defense response, immune effector process, cytokine production, and protein serine/threonine kinase activity, which could have an important function in the regulation of T-cell activation [41]. The activation of these gene sets in the DT aorta illustrates mechanisms of atheroprotection contributing to the atheroresistance of the DT aorta to the development of atherosclerotic plaques. In line with the observed specificity in the gene expression profile of cells derived from the AA&R and DT aortas, respectively, we observed district-specific gene expression profiles of the identified 12 clusters, mostly based on the side-specific predisposition, and less in response to hypercholesteremia. The clusters predominantly composed of cells derived from the AA&R and showed a gene expression profile of clusters with a pro-inflammatory phenotype promoting T-cell activation, in contrast to DT aorta-derived clusters with a more atheroprotective role, such as the negative regulation of inflammation, blood coagulation, lipoprotein metabolism, and repair. T-cells are activated in an antigen-specific manner in the atherosclerotic plaque by antigen presenting cells, including macrophages in the plaque, B-cells in the adventitia, conventional dendritic cells, and plasmacytoid dendritic cells [68]. Interestingly we revealed a cluster of T-cells derived from the AA&R and DT aorta cells characterized by the expression of gene sets of T-cell receptor signaling pathways, lymphocyte-mediated immunity, and the positive regulation of cytokine production, particularly IFN-γ. As T-cells are highly plastic, the state of the T-cells in the atherosclerotic plaque appears to be driven by the environmental activation signals promoting activation, exhaustion, development, inflammation, and the recognition of autoantigens, resulting in autoimmunity [69].

The identified subsets of pro-atherogenic or pro-resolving macrophages found during atherosclerosis progression likely corresponded to distinct functional states as a reflection of the specific environmental signals, rather than predetermined cell subsets [70,71]. Hyaluronan receptor LYVE-1 macrophage subpopulations appeared to have distinct functions within the vessel wall, since the specific depletion of LYVE-1 macrophages in Lyve1^wt/cre^ Csf1r^flox/flox^ mice resulted in increased arterial stiffness and collagen deposition [5]. LYVE-1 res-like macrophages are present in both healthy and atherosclerotic aortas [2,67]. Furthermore, Kim et al. distinguished three macrophage subsets expressing *Lyve1* in mouse atherosclerotic plaques using scRNAseq [6]. Interestingly, those identified in the present study population of LYVE-1 res-like macrophages expressing CCL24 were not only present in human atherosclerotic plaques prone to rupture, but the elevated plasma levels of CCL24 correlated with vascular calcification and carotid artery disease events in human atherosclerosis. CCL24 has been shown to play a role in the initiation and progression of atherosclerosis [62] and VSMC calcification [63]. Vascular calcification, principally driven by VSMCs [8], is linked to an increased risk of heart disease, stroke, atherosclerotic plaque rupture, vessel stiffness, systolic hypertension, increased diastolic dysfunction, and heart failure [8]. The VSMC phenotypic switch was characterized by the acquisition of markers of macrophages, mesenchymal stem cells, myofibroblasts [9], osteoblasts, and chondrocytes contributing to the destabilization of the lesions in late-stage atherosclerosis [10]. Importantly, eotaxin has been shown to induce the migration of VSMCs [72] and promotes VSMC proliferation, as well as increasing osteoblast/chondrocyte markers, alkaline phosphatase activity, and RUNX2 expression. The present study further demonstrated the pro-osteogenic effect of LYVE-1^+^CCL24^+^ on VSMCs.

Current anti-atherosclerosis therapies only modulate the factors associated with the development of the disease, and no therapy has, so far, been designed to directly target the mediators of vascular calcification. Progress in the area is dependent on targeting the cellular and soluble mediators that promote plaque destabilization. In the past few decades, accumulating evidence has increased our knowledge of the pathogenesis of vascular calcification; however, despite numerous studies, the complexity and diversity of vascular calcification pathophysiology has obstructed the discovery of the optimal drug targets, as well as drug development. Therapeutic strategies targeting cellular and soluble mediators are anticipated to reduce unmet clinical needs in vascular calcification. Extending mechanistic investigations on CCL24 may raise enticing therapeutic opportunities for abrogating the transdifferentiation of VSMCs into osteoblast/chondrocyte-like cells to prevent vascular calcification in atherosclerosis. The present study demonstrated an increased accumulation of LYVE-1^+^CCL24^+^ macrophages, particularly in human atherosclerotic plaques prone to rupture, as well as an association of the elevated plasma levels of CCL24 with vascular calcification and an increased risk of carotid artery disease events in humans. This is in line with previous findings showing that CCL24 is involved in the initiation and progression of atherosclerosis [62] and VSMC calcification [63]. Current anti-atherosclerosis therapies only modulate the factors associated with the development of the disease, and no therapy has, so far, been designed to directly target the mediators of vascular calcification or fibrosis. CM-101, which is a fully humanized, first-class monoclonal antibody that targets CCL24 has been shown to substantially attenuate fibrosis, reduce inflammatory injury, and significantly improve organ damage [73]. CM-101 is being developed as a treatment for patients with fibrosis-related diseases, such as primary sclerosing cholangitis (CM-101 in PSC patients, the SPRING study), systemic sclerosis, and non-alcoholic steatohepatitis. Targeting circulating CCL24 in atherosclerosis with CM-101 may prove to be a novel approach to not only reduce inflammation and vascular calcification, but to also have beneficial effects on plaque stability, reducing cardiovascular events.

## Data Availability

Data is contained within the article or supplementary material. Additional data that support the findings of this study are available from the corresponding author upon reasonable request.

## Supplementary Materials

The following are available online at https://www.mdpi.com/article/10.3390/cells11030411/s1, Figure S1: Quality control of the profiled scRNAseq transcriptomes of CD45^+^ cells derived from atherosclerosis-prone AA&R, and atherosclerosis-resistant DT aorta of Apoe^−/−^mice on NCD and HCD. (**A**) Violin plots showing the quality control metrics of (**A**) number of genes detected; (**B**) number of Unique molecular identifiers (UMI) *n* = 6 mice.; Figure S2: Bar graphs represent the mean ± SEM (**A**) cholesterol; (**B**) LDL-C, *n* = 6 mice/group, and *** *p* < 0.001. (**C**) Representative images of Oil Red O stained abdominal aorta of Apoe^−/−^ on NCD and HCD. (**D**) Relative frequency of cells derived from AA&R and DT aorta of Apoe^−/−^mice on NCD and HCD composing the 12 clusters; Figure S3: Heat map of top differentially expressed genes among all detected 12 clusters; Figure S4: GO terms enrichment analysis of AA&R and DT aorta derived clusters of Apoe^−/−^ on NCD and HCD. Bubble plot of selected GO terms enrichment analysis of: (**A**) cluster 0; (**B**) cluster 1; (**C**) cluster 3, (**D**) cluster 5, (**E**) cluster 6; (**F**) cluster 7; (**G**) cluster 8, (**H**) cluster 9 and (**I**) cluster 10. Dot size is proportional to the number of genes overlapping with each GO term, and the adjusted p-value is colour-coded from red to blue; Figure S5: (**A**) Representative images of CD68 positive cells-stained aortic roots of Apoe^−/−^ on NCD and HCD. (**B**) Bar graphs represent the mean ± SEM of total CD68 positive cells in aortic roots of Apoe^−/−^ on NCD and HCD, *n* = 6 mice per group and * *p* < 0.05. (**C**) Representative immunofluorescence staining showing LYVE1 (green) expression in close proximity to VSMC (Myh11 positive staining in red) in atherosclerotic lesions in areas of microcalcification as indicated by Osteosense positive staining (purple) in symptomatic CAD patients. (**D**) Dotplots showing exclusion of doublets and LIVE/DEAD Fixable Near-IR Dead Cell Dye positive cells and selection of Hoechst cells in the aortic arch and root and abdominal aorta, before gating for markers of interest. Table S1.
