# Supplementary material for: Single-Cell RNA-Seq Reveals a Crosstalk between Hyaluronan Receptor LYVE-1-Expressing Macrophages and Vascular Smooth Muscle Cells
Source: Cells. 2022 Jan 25;11(3):411. doi: 10.3390/cells11030411 (PMC8834524; doi:10.3390/cells11030411)
Supplement: Supplementary file 1 [file cells-11-00411-s001.zip › ! cells-1548159-supplementary.pdf]

Manuscript Supplementary
Figure S1.

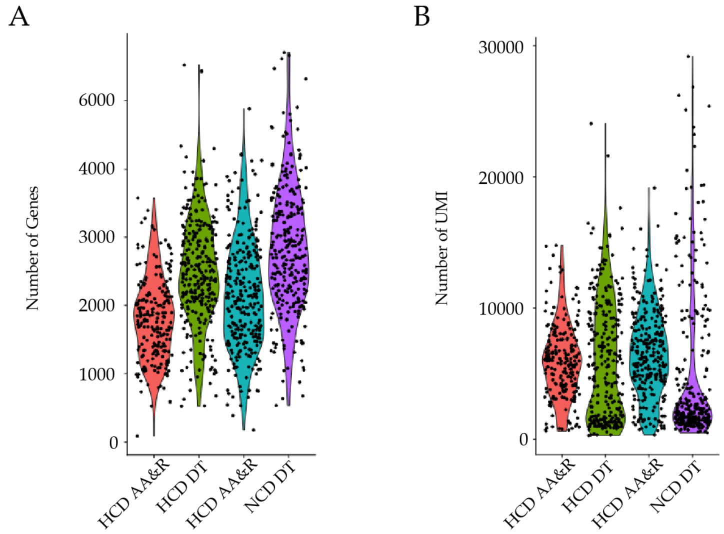

Figure S1. Quality control of the profiled scRNAseq transcriptomes of CD45$^+$ cells derived from atherosclerosis-prone AA&R, and atherosclerosis-resistant DT aorta of *Apoe*$^{-/-}$ mice on NCD and HCD. (A) Violin plots showing the quality control metrics of (A) number of genes detected; (B) number of Unique molecular identifiers (UMI) n=6 mice.

Figure S2.

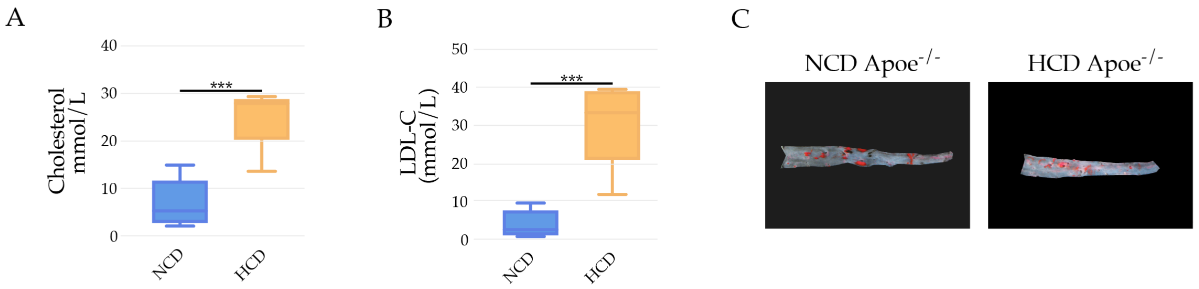

D

	AA&R		DT	
	NCD	HCD	NCD	HCD
0	2.2	7.9	3.9	86
1	42.5	45.6	8.8	3.1
2	74.4	25.6	0	0
3	17.4	17.4	43.1	22.2
4	0	0	100	0
5	15.1	17.8	21.9	45.2
6	31.2	12.5	18.8	37.5
7	65.5	31	3.4	0
8	3.7	18.5	74.1	3.7
9	0	0	100	0
10	0	0	100	0
11	0	0	100	0

Figure S2. Bar graphs represent the mean ± SEM (A) cholesterol; (B) LDL-C, n=6 mice/group, and ***p < 0.001. (C) Representative images of Oil Red O stained abdominal aorta of *Apoe*$^{-/-}$ on NCD and HCD. (D) Relative frequency of cells derived from AA&R and DT aorta of *Apoe*$^{-/-}$ mice on NCD and HCD composing the 12 clusters.

Figure S3

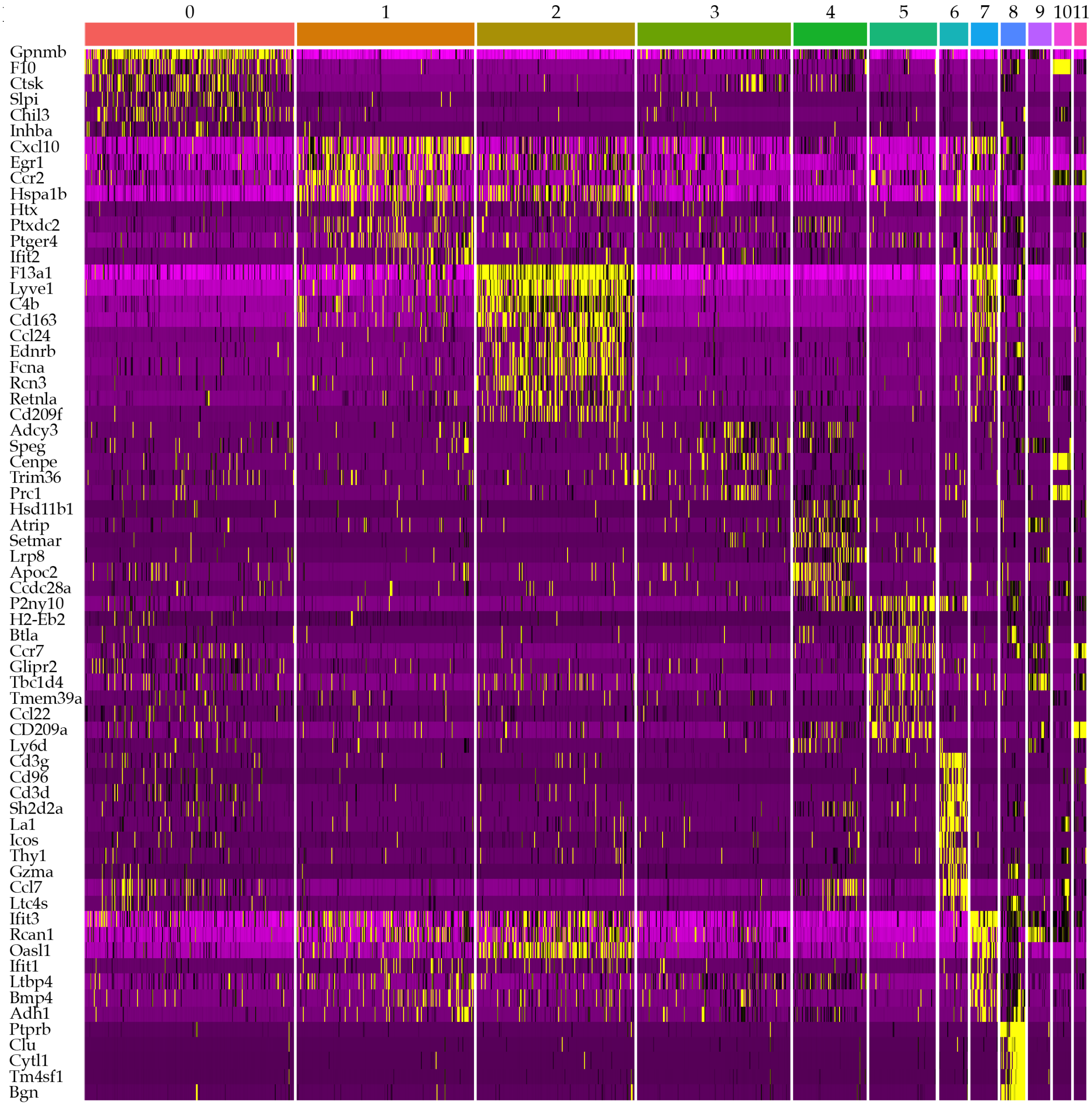

Figure S3. Heat map of top differentially expressed genes among all detected 12 clusters.

Figure S4

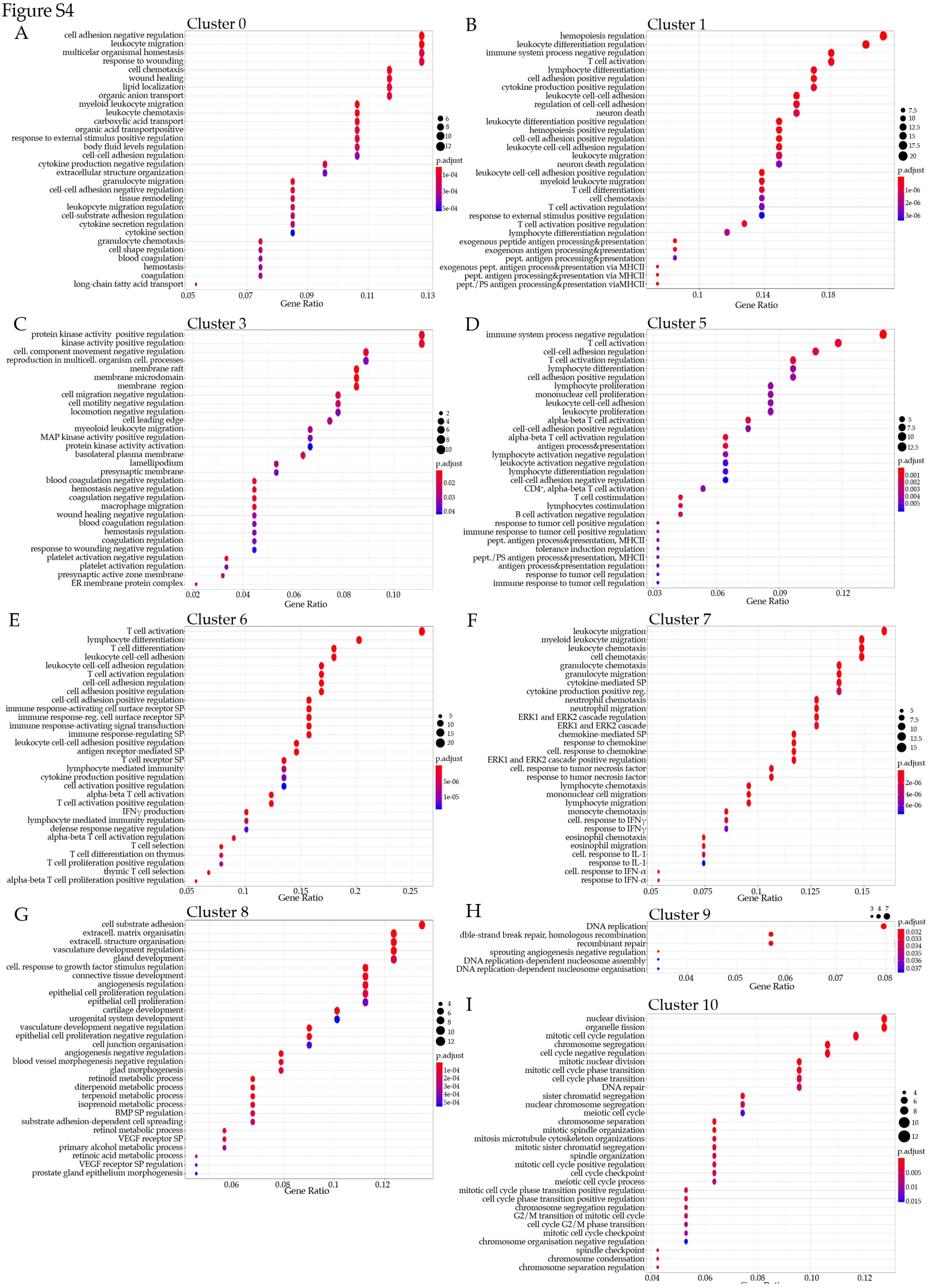

Figure S4. GO terms enrichment analysis of AA&R and DT aorta derived clusters of *Apoe*$^{-/-}$ on NCD and HCD. Bubble plot of selected GO terms enrichment analysis of: (A) cluster 0; (B) cluster 1; (C) cluster 3, (D) cluster 5, (E) cluster 6; (F) cluster 7; (G) cluster 8, (H) cluster 9 and (I) cluster 10. Dot

size is proportional to the number of genes overlapping with each GO term, and the adjusted *p*-value is colour-coded from red to blue.

Figure S5.

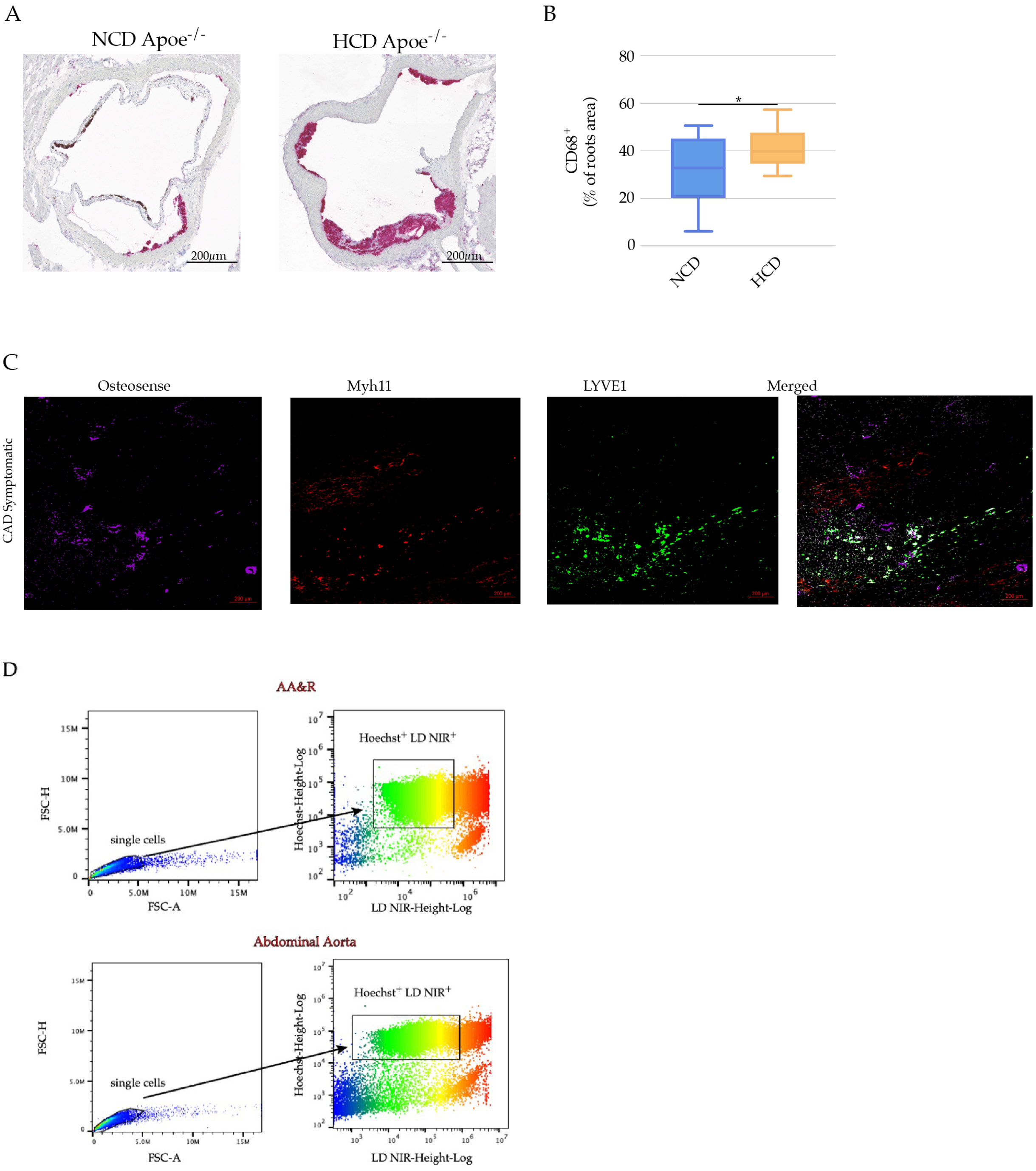

Figure S5. (A) Representative images of CD68 positive cells-stained aortic roots of *Apoe*$^{-/-}$ on NCD and HCD. (B) Bar graphs represent the mean ± SEM of total CD68 positive cells in aortic roots of *Apoe*$^{-/-}$ on NCD and HCD, n=6mice per group and *$p < 0.05$. (C) Representative immunofluorescence staining showing LYVE1 (green) expression in close proximity to VSMC (Myh11 positive staining in red) in

atherosclerotic lesions in areas of microcalcification as indicated by Osteosense positive staining (purple) in symptomatic CAD patients. (D) Dotplots showing exclusion of doublets and LIVE/DEAD Fixable Near-IR Dead Cell Dye positive cells and selection of Hoechst cells in the aortic arch and root and abdominal aorta, before gating for markers of interest.

Supplementary Table S1

log fold-chage of the average expression	Adjusted p-value	p-value	CLUSTER	GENE
1.716791367	1.18E-75	8.86E-80	0	Gpnmb
1.227479102	7.01E-61	5.25E-65	0	Spp1
0.848934922	2.86E-58	2.15E-62	0	Cstb
1.655057308	7.01E-58	5.25E-62	0	Fabp5
1.974309986	1.85E-56	1.38E-60	0	F7
1.708285942	6.50E-50	4.86E-54	0	Atp6v0d2
0.638836422	2.61E-48	1.96E-52	0	Ctsd
-0.420116227	9.94E-48	7.44E-52	0	Rn45s
1.312684434	3.89E-47	2.92E-51	0	Syngr1
1.118417206	1.29E-46	9.66E-51	0	Ctsl
1.230221854	2.33E-46	1.74E-50	0	Lhfpl2
-1.550659201	3.35E-46	2.51E-50	0	Tmem176b
0.731842988	3.34E-44	2.51E-48	0	Plin2
0.611555392	4.96E-43	3.72E-47	0	Lgals3
0.912953025	1.61E-41	1.20E-45	0	Gngt2
1.816545861	8.20E-39	6.14E-43	0	F10
1.506373525	3.01E-38	2.26E-42	0	Ctsk
1.187008737	4.71E-38	3.53E-42	0	Msrb1
0.696648955	5.78E-38	4.33E-42	0	Cd63
-1.397175818	3.40E-37	2.54E-41	0	Cd81
0.491612993	7.71E-37	5.77E-41	0	Cyba
0.923495847	4.91E-35	3.67E-39	0	Emp1
0.981596766	2.37E-34	1.77E-38	0	Mmp12
1.073697228	9.38E-34	7.02E-38	0	Anpep
1.606386303	4.07E-33	3.05E-37	0	Sgms2
0.971512387	4.40E-33	3.30E-37	0	Wsb2
1.150208642	5.33E-33	3.99E-37	0	Rnf128
0.982698976	7.72E-33	5.78E-37	0	Il7r
0.872505967	2.24E-32	1.68E-36	0	Anxa1
-1.934105549	5.85E-32	4.38E-36	0	Cbr2
0.710985989	6.35E-32	4.76E-36	0	Bhlhe40
0.86034885	6.73E-32	5.04E-36	0	Lpl
1.15803012	2.93E-31	2.20E-35	0	Hilpda
0.567229305	4.94E-31	3.70E-35	0	Psap
1.001173567	7.58E-31	5.68E-35	0	Fn1
0.708604862	1.07E-30	8.01E-35	0	Abcg1
0.799876016	1.23E-30	9.19E-35	0	Pld3
1.239078294	1.81E-30	1.36E-34	0	Mmp19
0.449582744	2.10E-30	1.58E-34	0	Cd68
0.98813351	8.29E-29	6.21E-33	0	Acp5
1.19346815	8.31E-29	6.22E-33	0	Mgst1
0.693657722	8.54E-29	6.40E-33	0	Psmb6
1.11686649	8.60E-29	6.44E-33	0	Pdpn
1.336876089	2.40E-27	1.80E-31	0	Thbs1
1.175171478	2.52E-27	1.88E-31	0	Fabp4
0.858060305	3.62E-27	2.71E-31	0	Cd9
0.774714826	4.18E-27	3.13E-31	0	Sgk1
1.283242241	4.40E-27	3.30E-31	0	Sdc1
0.367553048	7.25E-27	5.43E-31	0	Ctss
0.663265774	4.70E-26	3.52E-30	0	Rnh1
0.557200858	5.95E-26	4.46E-30	0	Abca1
2.035346051	1.62E-25	1.21E-29	0	Slpi
0.898622011	1.71E-25	1.28E-29	0	Mmp14
1.228942117	4.48E-25	3.35E-29	0	Vegfa
0.743480068	5.25E-25	3.93E-29	0	Adam8

0.782955887	1.42E-24	1.06E-28	0	Atp6v1c1
1.832793499	1.55E-24	1.16E-28	0	Chil3
0.805755736	2.13E-24	1.60E-28	0	Clec7a
0.398768594	3.50E-24	2.63E-28	0	Vim
0.803455591	5.63E-24	4.22E-28	0	Galnt6
0.717417829	1.43E-23	1.07E-27	0	Anxa4
0.99403417	1.75E-23	1.31E-27	0	Rgcc
1.02995677	2.30E-23	1.72E-27	0	Scd2
1.347767361	3.45E-23	2.59E-27	0	Arg2
1.183415397	4.53E-23	3.40E-27	0	Fam63a
0.858475148	5.26E-23	3.94E-27	0	Il1rn
1.182612336	6.63E-23	4.96E-27	0	1300002K09Rik
-1.57291794	1.23E-22	9.20E-27	0	Hspa1a
0.513434452	1.24E-22	9.30E-27	0	Anxa2
0.588844792	1.77E-22	1.32E-26	0	Atp6v1a
0.755920757	2.21E-22	1.65E-26	0	Esd
0.41740763	2.27E-22	1.70E-26	0	Akr1a1
0.960045562	3.56E-22	2.66E-26	0	Tmem65
0.64590084	4.80E-22	3.60E-26	0	Creg1
0.737484662	6.32E-22	4.74E-26	0	Emb
0.749001999	1.11E-21	8.28E-26	0	Soat1
0.809652227	1.14E-21	8.52E-26	0	Gla
0.299367298	1.24E-21	9.32E-26	0	Ctsb
0.589663432	1.33E-21	9.99E-26	0	Trem2
1.088938319	1.43E-21	1.07E-25	0	Cd300lf
-1.791830253	1.90E-21	1.42E-25	0	Slco2b1
-0.665071232	3.50E-21	2.62E-25	0	Serinc3
0.61895248	4.27E-21	3.20E-25	0	Pla2g7
0.344390153	6.09E-21	4.56E-25	0	Ftl1
0.687809458	8.35E-21	6.25E-25	0	Ctnnb1
0.356310714	1.10E-20	8.20E-25	0	Lamp1
-1.058993804	2.10E-20	1.57E-24	0	Ccnd1
0.619561547	2.20E-20	1.65E-24	0	Txn1
0.578109787	2.44E-20	1.83E-24	0	Aprt
0.581895398	3.91E-20	2.93E-24	0	Rnf149
0.950411022	6.01E-20	4.50E-24	0	Slc27a1
-1.430062701	1.30E-19	9.72E-24	0	Lifr
0.529184438	2.88E-19	2.16E-23	0	Prdx1
0.770162869	5.37E-19	4.02E-23	0	Gstm1
0.963550738	6.60E-19	4.95E-23	0	Fam20c
0.599155149	6.93E-19	5.19E-23	0	Prr13
0.557834606	7.36E-19	5.52E-23	0	Osbpl8
0.51898831	7.60E-19	5.70E-23	0	Itgb2
-1.86719763	8.35E-19	6.25E-23	0	Mgl2
0.824518264	8.87E-19	6.65E-23	0	Clec4d
0.969478343	1.28E-18	9.57E-23	0	Papss2
-1.302622594	1.56E-18	1.17E-22	0	Ssh2
-0.829568696	2.74E-18	2.05E-22	0	Trf
1.018445472	7.37E-18	5.52E-22	0	Gsr
0.583947543	1.11E-17	8.33E-22	0	Atp6v1b2
0.651625296	1.29E-17	9.67E-22	0	Hvcn1
0.804237283	1.50E-17	1.12E-21	0	Clec4n
-0.93232217	1.58E-17	1.18E-21	0	Slamf9
0.456852458	1.78E-17	1.33E-21	0	Ndufa3
-1.369508537	1.97E-17	1.48E-21	0	Fcgrt
0.306037385	2.03E-17	1.52E-21	0	Ctsz
1.326530856	2.19E-17	1.64E-21	0	Slc7a11
0.629922197	2.29E-17	1.72E-21	0	Vat1
0.929703073	2.33E-17	1.75E-21	0	Flrt3
0.651907994	3.63E-17	2.72E-21	0	Commd6
0.547624696	3.88E-17	2.90E-21	0	Atp6v1e1

0.563226517	4.26E-17	3.19E-21	0	Ndufa1
0.852582252	4.32E-17	3.24E-21	0	Mocs2
1.668846714	6.77E-17	5.07E-21	0	Inhba
0.513800858	6.83E-17	5.12E-21	0	Tnfaip2
-2.071280488	8.12E-17	6.08E-21	0	Lyve1
0.346858055	2.00E-16	1.50E-20	0	Gabarap
0.590364229	2.09E-16	1.57E-20	0	Fam129b
-1.717281011	4.17E-16	3.12E-20	0	Igfbp4
0.743073796	4.62E-16	3.46E-20	0	Fblim1
0.482707904	6.02E-16	4.51E-20	0	Capg
0.996410807	6.61E-16	4.95E-20	0	Clec4e
-1.542274291	8.35E-16	6.26E-20	0	Tmem176a
-0.739953442	8.72E-16	6.53E-20	0	Cd83
-1.863043046	1.64E-15	1.23E-19	0	Retnla
0.772988429	1.92E-15	1.44E-19	0	Gzf1
0.441237408	2.92E-15	2.19E-19	0	Atp6v0e
0.655457163	3.22E-15	2.41E-19	0	Sod2
0.894330476	5.59E-15	4.18E-19	0	Slc6a8
0.511363022	6.09E-15	4.56E-19	0	Nceh1
0.735833037	6.43E-15	4.82E-19	0	Eepd1
0.658369398	7.69E-15	5.76E-19	0	Gde1
-1.269739027	7.74E-15	5.80E-19	0	Cxcl10
0.525722916	1.00E-14	7.49E-19	0	Degs1
0.436037489	1.24E-14	9.26E-19	0	Pkm
0.613195027	1.25E-14	9.37E-19	0	Gdf15
0.55900008	1.55E-14	1.16E-18	0	Plaur
0.767755058	1.78E-14	1.33E-18	0	Znhit1
0.615265462	2.55E-14	1.91E-18	0	Malt1
0.556524477	2.82E-14	2.11E-18	0	Csf2rb2
0.800151629	2.86E-14	2.14E-18	0	Atg12
-0.712910828	2.99E-14	2.24E-18	0	H2-Eb1
0.686473188	3.00E-14	2.25E-18	0	Cd300a
0.318124279	3.07E-14	2.30E-18	0	Lgals1
0.523806465	3.55E-14	2.66E-18	0	Cd84
0.528170633	3.61E-14	2.70E-18	0	Tgm2
0.618859419	4.12E-14	3.09E-18	0	Cox17
0.412818825	4.15E-14	3.11E-18	0	Aldoa
0.522625925	4.25E-14	3.18E-18	0	Prdx5
-1.447943336	5.90E-14	4.42E-18	0	Folr2
0.567229707	5.96E-14	4.46E-18	0	Ckap4
0.651570296	6.92E-14	5.18E-18	0	Msr1
-1.025996767	1.10E-13	8.20E-18	0	Fcrls
0.599182635	1.25E-13	9.39E-18	0	Lyz1
0.406508582	1.28E-13	9.61E-18	0	Mpeg1
0.775665309	1.68E-13	1.26E-17	0	1110008P14Rik
0.650477794	1.74E-13	1.31E-17	0	AI413582
0.600301798	1.95E-13	1.46E-17	0	Fnip2
0.524535564	2.37E-13	1.78E-17	0	Smpdl3a
-0.841290272	3.34E-13	2.50E-17	0	Stab1
0.350038312	3.58E-13	2.68E-17	0	Sdcbp
-1.954856214	4.06E-13	3.04E-17	0	Cd163
-0.772433137	4.56E-13	3.41E-17	0	Tgfbr2
0.518641423	5.63E-13	4.21E-17	0	Gsto1
0.900848558	9.45E-13	7.08E-17	0	Cipc
0.804555189	1.18E-12	8.85E-17	0	Igf2r
0.450054532	1.27E-12	9.50E-17	0	2010107E04Rik
0.868064139	1.83E-12	1.37E-16	0	Hsd3b7
0.529746933	2.19E-12	1.64E-16	0	Clcn7
-0.549362296	2.72E-12	2.04E-16	0	Csf1r
0.524221109	2.82E-12	2.11E-16	0	Csf2rb
0.466816274	3.55E-12	2.66E-16	0	Trim25

0.306120779	4.27E-12	3.20E-16	0	Anxa5
0.977191092	4.41E-12	3.31E-16	0	Gng11
-1.163109857	4.58E-12	3.43E-16	0	Mrc1
0.633277923	4.65E-12	3.49E-16	0	Hist1h2bc
0.778181376	5.65E-12	4.23E-16	0	Echs1
0.549771778	6.46E-12	4.84E-16	0	Uap1l1
-1.694557527	6.53E-12	4.89E-16	0	Cp
0.268409176	7.06E-12	5.29E-16	0	Npc2
-0.926968596	8.78E-12	6.57E-16	0	Tcf4
0.364339748	9.88E-12	7.40E-16	0	Mir692-1
-1.363618064	1.18E-11	8.84E-16	0	Cx3cr1
0.830930505	1.34E-11	1.00E-15	0	Hk3
0.625756821	1.60E-11	1.19E-15	0	P2rx4
0.423993315	1.68E-11	1.26E-15	0	AF251705
0.765171574	1.87E-11	1.40E-15	0	Sar1b
0.488616634	2.00E-11	1.50E-15	0	Tpp1
0.588992827	2.42E-11	1.81E-15	0	Lrp12
0.664390821	2.71E-11	2.03E-15	0	Mfsd7b
0.550054161	2.86E-11	2.15E-15	0	Cebpb
-0.643276182	3.66E-11	2.74E-15	0	Ccl4
0.724275596	4.25E-11	3.18E-15	0	Cd200
0.471914801	4.69E-11	3.51E-15	0	Myof
0.754728213	5.65E-11	4.23E-15	0	Ap3s1
0.393667103	6.20E-11	4.64E-15	0	5730416F02Rik
0.977104633	6.63E-11	4.97E-15	0	Gyg
0.540994985	7.17E-11	5.37E-15	0	Cib1
-0.870411238	7.61E-11	5.70E-15	0	Cd72
0.716408425	7.86E-11	5.89E-15	0	Fgr
0.586445003	8.16E-11	6.11E-15	0	Nkap
-1.371593863	9.60E-11	7.19E-15	0	Cdr2
-1.283823148	9.94E-11	7.44E-15	0	Maf
0.451174553	1.01E-10	7.59E-15	0	Ndufc1
0.493489504	1.11E-10	8.34E-15	0	Atp6v1d
0.824611825	1.13E-10	8.49E-15	0	Fbxo32
0.736915006	1.19E-10	8.89E-15	0	Slc37a2
0.429154856	1.24E-10	9.26E-15	0	Lipa
0.330195741	1.28E-10	9.57E-15	0	Emp3
-1.323746637	1.47E-10	1.10E-14	0	Hpgd
-1.429404343	1.97E-10	1.48E-14	0	Abca9
0.648575901	2.14E-10	1.60E-14	0	Setd8
0.818534599	2.24E-10	1.68E-14	0	Ddhd1
0.552204488	2.81E-10	2.10E-14	0	Ndufb6
-1.641111207	3.02E-10	2.26E-14	0	Hspa1b
0.624426453	3.41E-10	2.56E-14	0	Tmem165
0.328988873	3.50E-10	2.62E-14	0	Shfm1
0.515080884	3.74E-10	2.80E-14	0	Npc1
0.534894382	3.90E-10	2.92E-14	0	Abr
0.356221174	4.08E-10	3.05E-14	0	Pnpla7
-0.579415543	4.14E-10	3.10E-14	0	H2-Ab1
0.62416641	5.17E-10	3.88E-14	0	Lonrf3
0.410863925	6.15E-10	4.61E-14	0	S100a6
-1.271835738	6.39E-10	4.79E-14	0	Col14a1
-0.879359569	6.58E-10	4.93E-14	0	Pf4
-0.651971961	6.85E-10	5.13E-14	0	Emr1
-1.096487933	7.44E-10	5.57E-14	0	Alox5
0.460771279	7.81E-10	5.85E-14	0	Tomm6
0.460561539	8.25E-10	6.18E-14	0	Nagk
-1.506035125	8.64E-10	6.47E-14	0	Ccl12
-1.166818947	8.69E-10	6.51E-14	0	Dst
0.661031817	9.61E-10	7.20E-14	0	Iqsec1
-0.342531298	1.01E-09	7.56E-14	0	Rps20

0.439002802	1.01E-09	7.57E-14	0	Mrpl20
-0.98311544	1.10E-09	8.25E-14	0	Mef2c
0.606421609	1.20E-09	8.99E-14	0	Cav2
-0.591894823	1.27E-09	9.49E-14	0	Dab2
0.511747248	1.65E-09	1.23E-13	0	Fbxo33
-1.423774277	1.70E-09	1.27E-13	0	Gas6
-1.542817962	1.76E-09	1.32E-13	0	Ptgs1
0.47716265	1.88E-09	1.41E-13	0	Lrpap1
0.614890421	1.97E-09	1.48E-13	0	Gng12
0.344649391	2.07E-09	1.55E-13	0	Mcfd2
-0.603227731	2.13E-09	1.59E-13	0	Pld4
0.349891076	2.15E-09	1.61E-13	0	Litaf
0.690466338	2.34E-09	1.76E-13	0	Npy
-0.254141561	2.51E-09	1.88E-13	0	Malat1
0.290658924	2.51E-09	1.88E-13	0	Chchd2
0.517725009	2.61E-09	1.95E-13	0	Nus1
0.386267071	2.74E-09	2.05E-13	0	Tnfsf12
0.801102114	3.04E-09	2.28E-13	0	Plxna1
0.45054292	3.12E-09	2.33E-13	0	Bnip3l
0.461477917	3.29E-09	2.46E-13	0	Gpr137b-ps
0.472943819	3.64E-09	2.73E-13	0	Mrpl36
0.465725698	4.31E-09	3.23E-13	0	Esyt1
1.137870062	4.45E-09	3.33E-13	0	Csf1
0.673220979	4.62E-09	3.46E-13	0	Large
0.514402265	4.91E-09	3.68E-13	0	Atp6v0d1
0.38034075	5.39E-09	4.04E-13	0	Ccl6
0.838850561	5.60E-09	4.19E-13	0	Atp1a3
-1.112669536	5.73E-09	4.29E-13	0	Cfh
0.522814648	9.81E-09	7.35E-13	0	S100a1
0.463809692	9.82E-09	7.36E-13	0	Lgals9
0.565929292	1.24E-08	9.26E-13	0	1810058I24Rik
0.412290285	1.24E-08	9.32E-13	0	Dpm2
0.400761208	1.61E-08	1.21E-12	0	Mdfic
-0.747013789	1.71E-08	1.28E-12	0	Ifi27l2a
0.345410715	1.87E-08	1.40E-12	0	Tmem256
0.357925367	1.98E-08	1.48E-12	0	Cox5a
0.413379207	2.18E-08	1.63E-12	0	Fam214b
0.606018603	2.23E-08	1.67E-12	0	Cd274
0.268579915	2.35E-08	1.76E-12	0	Atp6v0b
-0.293370478	2.43E-08	1.82E-12	0	Hspa8
0.715922018	2.50E-08	1.87E-12	0	Sowahc
0.366689698	2.55E-08	1.91E-12	0	Pdia6
0.474768903	2.76E-08	2.07E-12	0	Mtpn
-1.438207083	2.93E-08	2.19E-12	0	F13a1
0.657888391	3.02E-08	2.26E-12	0	Mfge8
0.393818153	3.45E-08	2.58E-12	0	Gpr137b
0.35591425	3.57E-08	2.68E-12	0	Vdac2
-0.55733396	3.59E-08	2.69E-12	0	Cd74
0.389802129	3.79E-08	2.84E-12	0	Taldo1
-1.741221494	3.94E-08	2.95E-12	0	C4b
-1.363089004	4.40E-08	3.29E-12	0	Fxyd2
0.348822557	4.97E-08	3.72E-12	0	Surf4
0.455427437	5.00E-08	3.75E-12	0	Blvra
0.397701657	5.08E-08	3.80E-12	0	Nme1
0.390834803	5.59E-08	4.18E-12	0	Cox5b
0.552280964	6.10E-08	4.57E-12	0	Vwf
0.82108316	7.18E-08	5.37E-12	0	Htra1
0.377322436	9.73E-08	7.29E-12	0	Spcs1
-1.376502792	1.02E-07	7.62E-12	0	Lilra5
0.426605325	1.08E-07	8.10E-12	0	Ssu72
0.518664912	1.14E-07	8.56E-12	0	Cln3

-1.151013701	1.19E-07	8.88E-12	0	Txnip
0.555602437	1.22E-07	9.16E-12	0	Tmem9b
0.39222466	1.26E-07	9.43E-12	0	Tgif1
0.984436236	1.29E-07	9.69E-12	0	Alas1
0.348498249	1.46E-07	1.09E-11	0	Cd44
0.263009033	1.64E-07	1.23E-11	0	Cox6b1
0.523817912	1.78E-07	1.33E-11	0	Grpel1
-1.476510478	1.87E-07	1.40E-11	0	Clec4b1
0.468570295	2.03E-07	1.52E-11	0	0610012G03Rik
0.337455011	2.10E-07	1.57E-11	0	Lgmn
0.355842696	2.41E-07	1.81E-11	0	Ms4a6d
0.479255094	2.68E-07	2.01E-11	0	Cmas
0.282533233	2.70E-07	2.02E-11	0	Gga2
0.411675622	2.79E-07	2.09E-11	0	Psmc4
0.435844134	2.98E-07	2.23E-11	0	Psmd14
0.729661695	3.53E-07	2.64E-11	0	Pqlc3
-0.719930085	3.56E-07	2.67E-11	0	Stk17b
0.857432448	3.90E-07	2.92E-11	0	Fam198b
0.279295961	4.10E-07	3.07E-11	0	Tcirg1
0.777626928	4.32E-07	3.24E-11	0	Htatip2
0.426048973	4.52E-07	3.39E-11	0	BC031181
0.314912966	4.60E-07	3.45E-11	0	Cox6c
0.361230993	5.24E-07	3.92E-11	0	C3
0.551189675	5.40E-07	4.05E-11	0	Rnf181
0.348039901	5.59E-07	4.18E-11	0	Arhgap9
0.524490788	5.91E-07	4.43E-11	0	Ipo5
0.591369806	6.29E-07	4.71E-11	0	Pbdc1
-1.266079328	6.74E-07	5.05E-11	0	Gpr34
0.47801251	7.00E-07	5.24E-11	0	Ube2a
0.305513071	7.84E-07	5.87E-11	0	Rnasek
0.303621688	8.32E-07	6.23E-11	0	Lrp1
0.400847642	9.01E-07	6.75E-11	0	Snx10
0.454523962	9.38E-07	7.02E-11	0	Snx1
0.499240273	9.66E-07	7.23E-11	0	Aurkaip1
0.387132027	9.70E-07	7.27E-11	0	Atp6v1f
0.404755351	9.70E-07	7.27E-11	0	Ccl9
0.442171327	9.91E-07	7.42E-11	0	Lasp1
-0.586240057	1.06E-06	7.91E-11	0	Adrbk1
0.552638762	1.07E-06	8.03E-11	0	Igsf8
0.333186159	1.19E-06	8.89E-11	0	Sgpl1
0.290772259	1.25E-06	9.36E-11	0	Snrnp27
0.449631474	1.35E-06	1.01E-10	0	Ndufa9
0.362614789	1.47E-06	1.10E-10	0	Scamp3
0.660761607	1.47E-06	1.10E-10	0	Agtrap
0.436460494	1.67E-06	1.25E-10	0	Gaa
-0.580800663	1.77E-06	1.32E-10	0	Ptpn18
0.320046317	1.79E-06	1.34E-10	0	Psen2
-1.466577037	1.83E-06	1.37E-10	0	Ltc4s
0.343268802	1.87E-06	1.40E-10	0	Vps35
0.615671703	1.89E-06	1.42E-10	0	Evl
0.432755556	1.93E-06	1.45E-10	0	Ugp2
-0.931999898	2.08E-06	1.56E-10	0	Ptger4
-0.50572358	2.11E-06	1.58E-10	0	H2-Aa
0.397893986	2.18E-06	1.63E-10	0	Sep.15
-1.4530955	2.41E-06	1.80E-10	0	Ednrb
0.557280026	2.55E-06	1.91E-10	0	Sdhd
0.356073272	2.62E-06	1.96E-10	0	Cd93
0.25256608	3.04E-06	2.28E-10	0	Gm2a
-0.641642815	3.07E-06	2.30E-10	0	Ccl3
-0.404488178	3.33E-06	2.49E-10	0	Nfkbiz
0.345157783	3.35E-06	2.51E-10	0	Irf2bpl

0.290472465	3.44E-06	2.57E-10	0	Uqcr11
0.465057205	3.47E-06	2.60E-10	0	Dtnbp1
0.319488475	3.52E-06	2.64E-10	0	S100a11
0.396921189	3.56E-06	2.67E-10	0	0610031J06Rik
-1.200176856	3.66E-06	2.74E-10	0	Adam19
-0.728879879	3.69E-06	2.76E-10	0	Zfp36l1
0.425869809	3.77E-06	2.83E-10	0	AB124611
0.333946208	4.02E-06	3.01E-10	0	Rab1
0.515829879	4.23E-06	3.16E-10	0	Ddt
0.383634655	4.34E-06	3.25E-10	0	Cox7a2
0.326399269	4.35E-06	3.26E-10	0	Atad1
-0.916460373	4.41E-06	3.31E-10	0	Syne1
-0.62936283	4.70E-06	3.52E-10	0	Eid1
0.373761205	5.67E-06	4.25E-10	0	Gusb
-1.229644209	6.25E-06	4.68E-10	0	Ifit1
0.686291097	6.71E-06	5.03E-10	0	Gpalpp1
0.30976861	7.49E-06	5.61E-10	0	Cd48
0.326429946	7.84E-06	5.87E-10	0	1110007C09Rik
0.939554732	7.97E-06	5.97E-10	0	Sh2b2
-1.084573848	8.40E-06	6.29E-10	0	Smagp
0.300728653	8.47E-06	6.34E-10	0	Atp6ap1
-1.177040945	9.44E-06	7.07E-10	0	Nes
0.418029127	9.58E-06	7.18E-10	0	Acaa2
-1.20290243	9.87E-06	7.39E-10	0	Fgd2
0.607766812	9.94E-06	7.44E-10	0	Mrpl38
0.541741516	9.94E-06	7.45E-10	0	Osm
1.454768337	1.13E-05	8.44E-10	0	S100a8
0.338112327	1.18E-05	8.86E-10	0	Pdcd6
0.530877056	1.19E-05	8.88E-10	0	Snhg3
-0.645419107	1.20E-05	8.98E-10	0	Ppp1r15a
0.376959334	1.21E-05	9.04E-10	0	Timm13
0.294677076	1.21E-05	9.07E-10	0	Zfand2a
0.488712677	1.22E-05	9.14E-10	0	Sgsh
0.336966888	1.25E-05	9.33E-10	0	Sirpa
-0.881358152	1.29E-05	9.64E-10	0	Peak1
0.326619945	1.32E-05	9.87E-10	0	Slc43a2
0.600804023	1.34E-05	1.00E-09	0	Pgd
-0.471201983	1.41E-05	1.06E-09	0	Wnk1
0.417429254	1.48E-05	1.11E-09	0	Atraid
0.296588888	1.59E-05	1.19E-09	0	Dusp3
0.334960481	1.64E-05	1.22E-09	0	Dap
0.307887893	1.64E-05	1.23E-09	0	Cox7b
-1.232794271	1.65E-05	1.24E-09	0	Psd3
0.461213693	1.84E-05	1.38E-09	0	Ostm1
-0.878044208	1.87E-05	1.40E-09	0	Trex1
0.355895973	1.88E-05	1.41E-09	0	Pygl
0.383158404	1.91E-05	1.43E-09	0	Ndufb8
-0.574822795	1.94E-05	1.45E-09	0	Marcksl1
0.454207901	2.12E-05	1.59E-09	0	Tmem189
-0.298865863	2.38E-05	1.78E-09	0	C1qc
0.486335011	2.41E-05	1.81E-09	0	E430025E21Rik
-0.941887721	2.43E-05	1.82E-09	0	Fnbp1l
-0.918489008	2.62E-05	1.96E-09	0	Pxdc1
-0.821660591	2.81E-05	2.10E-09	0	Pou2f2
0.503568152	2.91E-05	2.18E-09	0	Tmem203
-1.017437668	2.98E-05	2.23E-09	0	Ptchd1
-0.397750208	3.02E-05	2.26E-09	0	Rgs10
0.470725912	3.13E-05	2.35E-09	0	Tex264
0.422828659	3.14E-05	2.35E-09	0	Bax
0.291313547	3.17E-05	2.38E-09	0	Cpeb4
0.53288016	3.23E-05	2.42E-09	0	Tmem206

0.268036731	3.40E-05	2.54E-09	0	Pet100
-1.409351611	3.45E-05	2.59E-09	0	Rcn3
0.497341741	3.49E-05	2.61E-09	0	1700017B05Rik
0.42787176	3.62E-05	2.71E-09	0	Cers2
-1.115718027	3.70E-05	2.77E-09	0	Ch25h
-1.206021087	3.70E-05	2.77E-09	0	St3gal6
0.602722079	3.74E-05	2.80E-09	0	Cd24a
0.273978688	4.06E-05	3.04E-09	0	Fis1
-0.669398624	4.06E-05	3.04E-09	0	Cd86
0.380498898	4.15E-05	3.11E-09	0	Edem2
0.456930327	4.20E-05	3.15E-09	0	Atp6v1h
0.78576394	4.23E-05	3.17E-09	0	Nr1h3
0.381683211	4.62E-05	3.46E-09	0	Mkrn1
-0.831540382	4.67E-05	3.50E-09	0	Tnfsf13b
-0.749770551	5.01E-05	3.75E-09	0	Herpud1
0.4335402	5.08E-05	3.80E-09	0	Cd300lb
0.431246287	5.09E-05	3.81E-09	0	Samd8
0.343554188	5.19E-05	3.89E-09	0	Ndufa7
0.661800352	5.34E-05	4.00E-09	0	Orc3
0.672001547	5.41E-05	4.05E-09	0	Epas1
-1.553686747	5.49E-05	4.11E-09	0	Ccl24
0.252942585	5.55E-05	4.16E-09	0	Pomp
0.343228389	5.70E-05	4.27E-09	0	Mob4
-0.961882772	5.77E-05	4.32E-09	0	Fez2
0.322808503	5.99E-05	4.48E-09	0	Napsa
0.31071953	6.04E-05	4.52E-09	0	Hsbp1
0.45347025	6.30E-05	4.72E-09	0	Ly6c2
0.396881681	6.46E-05	4.84E-09	0	Fam96a
-0.410344036	6.57E-05	4.92E-09	0	Arap3
-0.835050162	6.61E-05	4.95E-09	0	Sh3pxd2a
0.36792593	6.67E-05	4.99E-09	0	Tceb1
-0.597606039	7.17E-05	5.37E-09	0	Prkcb
-0.763928115	7.59E-05	5.69E-09	0	Traf1
0.283027066	7.73E-05	5.79E-09	0	Sec11c
0.90755886	8.37E-05	6.27E-09	0	Kif3a
0.276589	8.37E-05	6.27E-09	0	Arl8b
-0.58446721	8.74E-05	6.55E-09	0	C5ar1
0.332739153	8.80E-05	6.59E-09	0	Atxn10
0.415239013	9.73E-05	7.29E-09	0	Zfp593
0.307083593	9.83E-05	7.36E-09	0	Atp6v1g1
0.298225099	0.000100864	7.55E-09	0	Vps29
0.261956133	0.000101158	7.58E-09	0	Rala
0.299846408	0.000101162	7.58E-09	0	Ubl3
0.526852262	0.000101385	7.59E-09	0	Impdh1
0.382204292	0.00010577	7.92E-09	0	Trappc2l
0.316928089	0.000107773	8.07E-09	0	Dnase2a
0.32903397	0.00010797	8.09E-09	0	Pfdn1
0.278740317	0.000108843	8.15E-09	0	Cox8a
0.494340156	0.000110594	8.28E-09	0	Timm17a
0.303022999	0.000114685	8.59E-09	0	Hpcal1
-0.869969754	0.000120003	8.99E-09	0	Gatm
-0.843098752	0.000121057	9.07E-09	0	Ptpro
0.722588128	0.000127044	9.52E-09	0	Mgat4b
0.429616489	0.000140651	1.05E-08	0	Cript
0.496637245	0.00014505	1.09E-08	0	Bst1
-1.183238349	0.000145329	1.09E-08	0	Fcna
0.274203813	0.00014541	1.09E-08	0	Psmd8
0.382468455	0.000151569	1.14E-08	0	Hectd3
0.411538149	0.000171254	1.28E-08	0	Cd22
-0.855886765	0.000190595	1.43E-08	0	Ctla2b
-0.787898886	0.000197525	1.48E-08	0	Ppp1r12c

0.490828577	0.000210573	1.58E-08	0	Ppt2
0.414725716	0.000223422	1.67E-08	0	Cnih4
-0.86907031	0.000232547	1.74E-08	0	Sipa1
0.293194228	0.000239088	1.79E-08	0	Slc25a5
0.326135017	0.000240634	1.80E-08	0	Usmg5
0.584720859	0.000242396	1.82E-08	0	Cyp4f18
0.858488237	0.000244431	1.83E-08	0	Dmxl2
0.565159853	0.000253241	1.90E-08	0	Rfwd2
0.364768343	0.000253337	1.90E-08	0	Mitf
-0.797177304	0.00025813	1.93E-08	0	Utrn
-0.960448261	0.000265066	1.99E-08	0	Rsad2
0.679953023	0.000265457	1.99E-08	0	Arhgap24
0.561928256	0.000270168	2.02E-08	0	Creld2
-0.763122787	0.000288613	2.16E-08	0	Ppp2r4
0.481942003	0.000339611	2.54E-08	0	Nudt8
0.5505704	0.00036579	2.74E-08	0	Sema4d
-0.49086598	0.000367029	2.75E-08	0	Neurl3
0.330804052	0.000383148	2.87E-08	0	Atp13a2
0.378698158	0.000395252	2.96E-08	0	Cat
0.535942328	0.00039599	2.97E-08	0	Slc16a10
0.521528007	0.000415723	3.11E-08	0	Psmc6
0.412334737	0.000429569	3.22E-08	0	Mrpl41
0.284891316	0.000431053	3.23E-08	0	Nampt
0.34844598	0.00044913	3.36E-08	0	Atp5j
0.651848333	0.000509388	3.82E-08	0	Mmadhc
0.466835844	0.000515218	3.86E-08	0	Bola2
-0.579591548	0.000525558	3.94E-08	0	Filip1l
-0.614931489	0.000539172	4.04E-08	0	Ehd4
0.267530177	0.000541732	4.06E-08	0	Ythdf1
0.47578374	0.000546017	4.09E-08	0	Hmox2
0.260151736	0.000550553	4.12E-08	0	Ak2
0.379456256	0.000557575	4.18E-08	0	Plxnd1
0.313886883	0.000593462	4.44E-08	0	Hif1a
0.301732041	0.000598143	4.48E-08	0	Tax1bp1
0.435759391	0.000641962	4.81E-08	0	Eif4e
0.266408737	0.000700962	5.25E-08	0	Ppp2r1a
0.590567534	0.000706436	5.29E-08	0	Elp2
0.38321239	0.000734545	5.50E-08	0	Gtf2h5
0.382652856	0.00075	5.62E-08	0	Slc11a1
0.32522444	0.00081145	6.08E-08	0	Akirin1
0.367718928	0.000855455	6.41E-08	0	Yif1b
0.268843477	0.000868437	6.50E-08	0	Sars
0.348776739	0.000888551	6.65E-08	0	Kcnk6
0.357942701	0.000895328	6.71E-08	0	Bbip1
0.262054874	0.000899324	6.74E-08	0	Dpp3
0.372080151	0.000921689	6.90E-08	0	Tmem208
0.251742667	0.000951418	7.13E-08	0	Mdm2
0.294427722	0.001006275	7.54E-08	0	Uqcrb
0.312562677	0.001050736	7.87E-08	0	Ssna1
-0.64578185	0.001079688	8.09E-08	0	Rel
0.495766482	0.001091625	8.18E-08	0	Rrs1
0.34203763	0.001094999	8.20E-08	0	B4galnt1
0.280630429	0.001117437	8.37E-08	0	Spcs2
0.576891876	0.001138577	8.53E-08	0	Mrto4
0.51998455	0.001160746	8.69E-08	0	Ly9
0.257264907	0.001161525	8.70E-08	0	Capns1
0.277946188	0.001170734	8.77E-08	0	Dbi
0.520660049	0.001182769	8.86E-08	0	Tmem38b
-0.28400402	0.001190613	8.92E-08	0	Ptma
-0.25707889	0.001229591	9.21E-08	0	Gnai2
0.280419645	0.001263021	9.46E-08	0	Atp1b3

0.55514274	0.001275143	9.55E-08	0	Tpra1
0.369840938	0.001281667	9.60E-08	0	D17Wsu104e
0.301546559	0.001304575	9.77E-08	0	Commd4
0.426541087	0.001304872	9.77E-08	0	Ezr
0.264696046	0.001324089	9.92E-08	0	Cd53
0.310842418	0.001350372	1.01E-07	0	Furin
0.316571673	0.001362191	1.02E-07	0	Atg3
0.32558831	0.001367246	1.02E-07	0	Matk
0.518870916	0.001485525	1.11E-07	0	Vti1b
0.523750806	0.001515364	1.13E-07	0	Gba
0.327892894	0.001523728	1.14E-07	0	Thoc3
0.259042824	0.001527331	1.14E-07	0	1810013L24Rik
0.299203549	0.001530505	1.15E-07	0	Vapa
0.707007988	0.001550901	1.16E-07	0	Ccdc167
0.677098026	0.001551513	1.16E-07	0	Bhlhe41
0.271656928	0.001562252	1.17E-07	0	Fkbp2
0.262744957	0.001567464	1.17E-07	0	Tbcb
0.254994174	0.001577147	1.18E-07	0	Mrpl30
-0.658156827	0.001711797	1.28E-07	0	Pmp22
0.367850419	0.001714872	1.28E-07	0	Chmp5
0.2724889	0.00174019	1.30E-07	0	Ecm1
-0.866099657	0.001872705	1.40E-07	0	Clec10a
-0.286210688	0.001885725	1.41E-07	0	C1qa
0.290872926	0.001889281	1.41E-07	0	Orc4
-0.786645062	0.001896017	1.42E-07	0	Ifi203
0.574088346	0.001943253	1.46E-07	0	Adssl1
-0.957331402	0.002260463	1.69E-07	0	Slc9a9
0.279517128	0.002267113	1.70E-07	0	Tmsb10
0.413915619	0.002359861	1.77E-07	0	Slc30a7
0.290204304	0.002384067	1.79E-07	0	Dctn3
0.418187997	0.002464007	1.85E-07	0	Slc31a2
0.368553831	0.002468595	1.85E-07	0	3110043O21Rik
0.557661187	0.002528181	1.89E-07	0	Ak6
0.589906717	0.00259751	1.95E-07	0	Tnfrsf21
-1.22808054	0.002810711	2.11E-07	0	Fam71a
0.286969502	0.002902361	2.17E-07	0	Sf3b5
0.335348733	0.002941862	2.20E-07	0	Hnrnpll
0.27250029	0.003010523	2.25E-07	0	Prelid1
0.35634299	0.003078817	2.31E-07	0	Fam104a
0.442242632	0.003191121	2.39E-07	0	Ndufs4
-0.626689668	0.003258757	2.44E-07	0	Jun
0.304476706	0.003285017	2.46E-07	0	Ethe1
0.333441627	0.003303711	2.47E-07	0	Igsf6
0.371663081	0.003336752	2.50E-07	0	Slc35f6
-0.874596893	0.00343849	2.58E-07	0	Rcsd1
0.457485265	0.003788032	2.84E-07	0	Tmem242
0.460784795	0.003820793	2.86E-07	0	Ap1s1
0.407985187	0.003825044	2.86E-07	0	Arl5a
0.294579561	0.003918603	2.93E-07	0	Dnajc15
0.292447621	0.003993886	2.99E-07	0	Actn1
0.278754994	0.004119069	3.08E-07	0	Serp1
0.375330462	0.004192061	3.14E-07	0	Slc2a1
0.410627225	0.004298671	3.22E-07	0	Cd36
0.321467274	0.004417537	3.31E-07	0	Renbp
0.332937007	0.004433889	3.32E-07	0	Arfgef2
-0.938610186	0.004437437	3.32E-07	0	Arhgap4
0.417815429	0.004711729	3.53E-07	0	Pgap1
0.489364494	0.004757286	3.56E-07	0	2310022A10Rik
0.309041073	0.004976836	3.73E-07	0	Glrx3
0.703937106	0.004992951	3.74E-07	0	Gnptab
0.530474627	0.005057033	3.79E-07	0	Dym

-0.499224118	0.005147585	3.86E-07	0	Sepp1
0.25691877	0.005391461	4.04E-07	0	Wfdc17
0.286319331	0.005396835	4.04E-07	0	Srek1ip1
0.379640105	0.005584888	4.18E-07	0	Arl8a
-0.708823564	0.005836359	4.37E-07	0	Pmepa1
0.269250438	0.005857832	4.39E-07	0	Tomm22
0.258013873	0.006110777	4.58E-07	0	Rps27l
0.418728254	0.006448859	4.83E-07	0	Alg1
0.571124153	0.006460151	4.84E-07	0	Sep.11
0.336898977	0.006537398	4.90E-07	0	Azin1
0.373751756	0.00677473	5.07E-07	0	Ndufb2
0.326653989	0.007141296	5.35E-07	0	Txnrd1
-0.657947427	0.007153026	5.36E-07	0	Sdc4
0.522480655	0.007219659	5.41E-07	0	Socs6
0.52126513	0.007423328	5.56E-07	0	Chchd4
0.285055939	0.007552936	5.66E-07	0	Zfp511
-1.094979731	0.007879154	5.90E-07	0	Mki67
0.380151569	0.008047464	6.03E-07	0	Slc48a1
0.297602829	0.008200935	6.14E-07	0	Tceal8
0.304677631	0.00823555	6.17E-07	0	Atp6v0a1
-1.206626516	0.008309637	6.22E-07	0	Apobec3
-0.369863614	0.008596261	6.44E-07	0	Rock2
0.298429858	0.008672136	6.50E-07	0	Mdh2
0.27961927	0.00886925	6.64E-07	0	Hnrnpab
-0.482762551	0.009035921	6.77E-07	0	Clic4
0.532552895	0.00909509	6.81E-07	0	Fuca2
-0.736099517	0.009254044	6.93E-07	0	Ap1b1
0.343532218	0.009732575	7.29E-07	0	Txndc17
0.605616451	0.01015747	7.61E-07	0	Smim12
0.518643951	0.010641294	7.97E-07	0	Csf3r
0.415201048	0.010951921	8.20E-07	0	Eno1b
0.253167124	0.011095776	8.31E-07	0	Coro1c
0.252787551	0.011249768	8.43E-07	0	Lgals3bp
0.498475843	0.011419871	8.55E-07	0	Gclm
-0.456163658	0.011470508	8.59E-07	0	Jmjd1c
0.419098302	0.012028175	9.01E-07	0	Grina
-0.862131579	0.012155718	9.10E-07	0	Ccnd2
0.259484454	0.01223711	9.17E-07	0	Myl12a
-0.744978757	0.012321805	9.23E-07	0	Cebpz
0.486976778	0.012730617	9.53E-07	0	Abcb7
0.515542319	0.013097755	9.81E-07	0	Golga2
0.346356465	0.013110866	9.82E-07	0	Atp5g1
0.288743573	0.013368902	1.00E-06	0	Tmem167
-0.535926489	0.013408892	1.00E-06	0	Parp14
0.577022428	0.013523551	1.01E-06	0	Slc39a11
-1.144799358	0.013792648	1.03E-06	0	Cmah
0.345577238	0.014031181	1.05E-06	0	Psmb5
0.30906475	0.014565255	1.09E-06	0	Ap2s1
0.375878123	0.014629079	1.10E-06	0	Mrps28
-0.688748115	0.01476135	1.11E-06	0	Cd40
-0.996145584	0.014944988	1.12E-06	0	Klf2
-0.551574564	0.014976813	1.12E-06	0	Ckb
-0.59724756	0.015196914	1.14E-06	0	Hnrnpul1
0.362316568	0.015812846	1.18E-06	0	1500011K16Rik
0.301549679	0.015862143	1.19E-06	0	Pnkd
0.285013337	0.015894658	1.19E-06	0	Ccdc86
0.404223056	0.01594248	1.19E-06	0	Rexo2
0.285297596	0.016115273	1.21E-06	0	Lamtor5
-0.829094778	0.01689215	1.27E-06	0	Ifih1
0.286228225	0.017060473	1.28E-06	0	Mrps18c
-0.87392955	0.017082001	1.28E-06	0	Arhgef3

0.34162629	0.017233807	1.29E-06	0	Tmem87b
0.367028522	0.017436943	1.31E-06	0	Nipsnap3b
0.459722149	0.018101625	1.36E-06	0	Bola1
0.353491298	0.020394934	1.53E-06	0	Emilin2
0.345063154	0.020489119	1.53E-06	0	Zbtb8os
0.278667662	0.021379376	1.60E-06	0	Pycard
-0.947519248	0.021508538	1.61E-06	0	Tagap
0.258320079	0.021709601	1.63E-06	0	Gyk
-0.414306103	0.022138389	1.66E-06	0	Pea15a
0.281658744	0.02223857	1.67E-06	0	Eif1a
-0.702131967	0.022707356	1.70E-06	0	Nfatc2
0.354754212	0.022976252	1.72E-06	0	Romo1
0.410988151	0.023041582	1.73E-06	0	Trappc4
-0.504843559	0.023312646	1.75E-06	0	Rcc2
0.456530816	0.023475832	1.76E-06	0	Timm8b
-0.521557323	0.02365916	1.77E-06	0	Oasl1
0.452216855	0.025103052	1.88E-06	0	Bri3
-0.814221845	0.026491621	1.98E-06	0	Egr1
-0.705883417	0.026563619	1.99E-06	0	Exoc3
-0.945535283	0.027149475	2.03E-06	0	Spn
0.280888116	0.028549462	2.14E-06	0	Naa38
0.274311765	0.028559715	2.14E-06	0	Mboat7
0.353090913	0.030087282	2.25E-06	0	Emc7
0.52556829	0.030952724	2.32E-06	0	Dlst
-0.548169738	0.031804956	2.38E-06	0	Fam110a
-0.818630004	0.031925572	2.39E-06	0	Il21r
-0.550281526	0.033335391	2.50E-06	0	Sertad2
0.66702471	0.033384914	2.50E-06	0	Fbxw8
0.306175676	0.033421189	2.50E-06	0	Mrpl14
-0.701010605	0.035411115	2.65E-06	0	Psmb9
-0.857924174	0.035449035	2.65E-06	0	Lyl1
0.363963934	0.035693047	2.67E-06	0	Mien1
0.414617287	0.035834966	2.68E-06	0	Map2k4
-0.835573574	0.036622902	2.74E-06	0	Pid1
0.363330584	0.036800902	2.76E-06	0	Sidt2
0.283824959	0.037257267	2.79E-06	0	Mrpl33
0.331051153	0.037637726	2.82E-06	0	Acot10
-0.707083992	0.037864678	2.84E-06	0	Btg2
0.284607233	0.038066682	2.85E-06	0	Ndufv3
0.426239765	0.038553748	2.89E-06	0	Toporsos
-0.714551687	0.0386348	2.89E-06	0	Map3k3
0.256466461	0.039200529	2.94E-06	0	Atp5h
0.276092945	0.040084192	3.00E-06	0	Ccr1
0.349468761	0.040629297	3.04E-06	0	Ralgds
0.263032837	0.04114391	3.08E-06	0	Amz1
0.571045652	0.041868098	3.14E-06	0	B430306N03Rik
0.374140365	0.04187399	3.14E-06	0	Scfd1
-0.391338756	0.042024574	3.15E-06	0	Bcl2
0.354929943	0.043657589	3.27E-06	0	Psma4
-0.327760841	0.044792789	3.35E-06	0	Skil
0.579374994	0.04545413	3.40E-06	0	Pphln1
0.34085206	0.046586178	3.49E-06	0	N6amt2
0.292503083	0.046763946	3.50E-06	0	Cyth1
0.481911374	0.047629304	3.57E-06	0	Tmem43
-0.336718434	0.04842943	3.63E-06	0	Klf6
-0.549337284	0.048582505	3.64E-06	0	Sbf2
-0.730201815	0.050010431	3.75E-06	0	Mapk14
-0.650057928	0.050581155	3.79E-06	0	Itpripl1
-0.753508786	0.052420535	3.93E-06	0	Nfkbid
0.338383408	0.053315695	3.99E-06	0	Cds2
0.33512923	0.053647472	4.02E-06	0	Tm9sf2

0.331755264	0.054241196	4.06E-06	0	Rap2a
0.971662606	0.054623229	4.09E-06	0	Met
0.524664198	0.054934888	4.11E-06	0	Gdf3
-0.440798752	0.05539434	4.15E-06	0	Ctsc
-0.646070537	0.055950488	4.19E-06	0	Gamt
-0.946930596	0.056590464	4.24E-06	0	Smc2
0.641058939	0.058372465	4.37E-06	0	Tecpr1
0.485554624	0.059132628	4.43E-06	0	Usp14
0.27338259	0.059397246	4.45E-06	0	Tnfrsf1b
0.613996482	0.059884024	4.49E-06	0	Ubqln2
0.564820313	0.061569818	4.61E-06	0	Vcl
-0.868166929	0.063137355	4.73E-06	0	Rnasel
0.277750246	0.065644519	4.92E-06	0	Psenen
0.340084598	0.065812765	4.93E-06	0	Mbp
0.308017916	0.067663397	5.07E-06	0	Ten1
0.651820701	0.068477581	5.13E-06	0	Nelfe
-0.633787383	0.068722975	5.15E-06	0	Cd2ap
0.29442228	0.06989467	5.23E-06	0	Sec61g
0.474393515	0.070012858	5.24E-06	0	Ptpmt1
0.29306529	0.070152904	5.25E-06	0	Ech1
-0.751979414	0.07037467	5.27E-06	0	Hes1
0.5830779	0.070837509	5.31E-06	0	Ltbp3
0.260486879	0.071073541	5.32E-06	0	Copa
0.364819481	0.071771999	5.38E-06	0	Abi1
0.663392034	0.072393463	5.42E-06	0	Aggf1
-0.769167501	0.079669476	5.97E-06	0	Phlda1
-1.014304397	0.083054875	6.22E-06	0	Stard8
0.388069035	0.083641533	6.26E-06	0	2310039H08Rik
-0.596649411	0.084029801	6.29E-06	0	Rnd3
0.272963518	0.085444847	6.40E-06	0	Plin3
0.292109598	0.085454808	6.40E-06	0	Utp6
0.540349067	0.0858357	6.43E-06	0	Alkbh3
-0.531796711	0.086463982	6.48E-06	0	Ehd1
0.579067733	0.086714774	6.49E-06	0	Tmem60
-0.336798074	0.087132573	6.53E-06	0	Marcks
0.361205873	0.091388662	6.84E-06	0	Polr2g
0.364025254	0.092185341	6.90E-06	0	Ttc13
-0.588545384	0.095038075	7.12E-06	0	Trim33
0.341086726	0.097925889	7.33E-06	0	Tm2d1
-0.320548721	0.100468469	7.52E-06	0	Fos
-0.501551799	0.10203518	7.64E-06	0	Ppt1
-0.651391567	0.102431776	7.67E-06	0	Phf11b
-0.98950835	0.103647044	7.76E-06	0	Mtus1
0.315387982	0.103931145	7.78E-06	0	Uckl1
0.274547263	0.106287386	7.96E-06	0	Pgs1
0.281812579	0.108700978	8.14E-06	0	Ufsp2
-0.853700862	0.109439238	8.20E-06	0	Arhgef6
0.522694394	0.110797506	8.30E-06	0	Snx12
0.318966734	0.112225482	8.41E-06	0	Plekhf2
-0.508028133	0.116298192	8.71E-06	0	Rbm33
-0.759335667	0.116457468	8.72E-06	0	Tanc2
-0.918175527	0.116505164	8.73E-06	0	Rrm1
0.268332885	0.116513694	8.73E-06	0	Wdr1
0.310686247	0.118712826	8.89E-06	0	Vps26a
0.256240541	0.120024902	8.99E-06	0	Ndufb3
-0.290950952	0.121640558	9.11E-06	0	Fyb
0.511398571	0.124087111	9.29E-06	0	Wdr81
0.431782991	0.124624852	9.33E-06	0	Cyp4v3
-0.951266034	0.124625969	9.33E-06	0	Elmsan1
0.295480926	0.126442249	9.47E-06	0	Taf12
0.264163792	0.126930798	9.51E-06	0	Cxcr4

0.359447355	0.127039633	9.51E-06	0	Snapc5
0.303040693	0.131376236	9.84E-06	0	Itgav
0.329818779	0.131818686	9.87E-06	0	Mcoln2
-0.541249119	0.13211014	9.89E-06	0	Frmd4b
-0.648696705	0.136223171	1.02E-05	0	Pla2g16
0.489522439	0.13788941	1.03E-05	0	Ccdc53
0.375632581	0.140427798	1.05E-05	0	Cln5
-0.475333775	0.140780281	1.05E-05	0	Ptprj
0.365268825	0.146913534	1.10E-05	0	Ift22
-0.440111586	0.147198616	1.10E-05	0	Dapp1
0.361948967	0.147419775	1.10E-05	0	Ppm1h
0.303310801	0.149918421	1.12E-05	0	Rbm34
-0.574368367	0.152404033	1.14E-05	0	Pik3r1
0.70747854	0.153398181	1.15E-05	0	Il1a
0.360206001	0.153480143	1.15E-05	0	Cdk17
0.263728726	0.1565715	1.17E-05	0	Dctn4
0.380808977	0.157002196	1.18E-05	0	Ptrhd1
0.48772309	0.159249168	1.19E-05	0	Ap5s1
-0.652625983	0.15952194	1.19E-05	0	Mdp1
0.473631778	0.164144556	1.23E-05	0	Sec11a
0.258335385	0.164287006	1.23E-05	0	1110008F13Rik
0.399502423	0.167565315	1.25E-05	0	Ptgr1
-0.587596894	0.168708392	1.26E-05	0	Cd33
-0.556216298	0.169081479	1.27E-05	0	Rapgef2
0.339601362	0.174977157	1.31E-05	0	Hsd17b10
-0.654104317	0.176394367	1.32E-05	0	Cttnbp2nl
0.252716423	0.176429279	1.32E-05	0	Chmp1b
-0.402954131	0.178595754	1.34E-05	0	Fam129a
0.432822808	0.1808427	1.35E-05	0	Tmem184b
0.283542716	0.181494522	1.36E-05	0	Sar1a
0.36772478	0.183602265	1.38E-05	0	Elof1
0.331049209	0.188881203	1.41E-05	0	Sptlc2
-0.679028073	0.19044664	1.43E-05	0	Ap2a2
0.398308449	0.19183452	1.44E-05	0	Rfc2
0.259611943	0.195481875	1.46E-05	0	Edf1
0.312304412	0.206200536	1.54E-05	0	Tmed7
0.410958963	0.21199992	1.59E-05	0	Mcee
-0.669239194	0.221201913	1.66E-05	0	Adam15
0.32494518	0.229348527	1.72E-05	0	Psmd6
-0.606864235	0.229651725	1.72E-05	0	Apc
0.362152481	0.242493787	1.82E-05	0	Naglu
-0.444202692	0.246936236	1.85E-05	0	2410006H16Rik
0.68307329	0.256565332	1.92E-05	0	Chst11
0.367482493	0.262665892	1.97E-05	0	Mgat2
0.376486377	0.265786273	1.99E-05	0	Edem1
0.477919353	0.267610724	2.00E-05	0	Dctn6
0.459298362	0.27034981	2.02E-05	0	Pgls
0.333682736	0.270583243	2.03E-05	0	Prep
-0.30315736	0.27137535	2.03E-05	0	Lsp1
-0.651284813	0.272704788	2.04E-05	0	Trib1
-0.571871434	0.275024711	2.06E-05	0	Fosb
0.324768173	0.279653417	2.09E-05	0	Rpl19
-0.713641604	0.29378715	2.20E-05	0	Samd9l
-0.303924578	0.297973711	2.23E-05	0	Rpl13a
0.305279239	0.302269115	2.26E-05	0	Mapk3
0.360645383	0.309810093	2.32E-05	0	Micu2
0.269308752	0.318967192	2.39E-05	0	Etfb
0.279579467	0.323708514	2.42E-05	0	Acaa1a
0.3796786	0.323818904	2.43E-05	0	Nubp1
-0.827428708	0.324524148	2.43E-05	0	Foxn3
0.273444327	0.327987201	2.46E-05	0	Ndufa2

0.335397126	0.330026127	2.47E-05	0	Leprotl1
0.411483602	0.334539158	2.51E-05	0	Ubac1
-0.624369256	0.338167274	2.53E-05	0	Rasa1
0.359277353	0.341788807	2.56E-05	0	Tor2a
-0.400217968	0.341889523	2.56E-05	0	Fmnl1
0.30424546	0.342707558	2.57E-05	0	Ythdf3
0.523328024	0.342800257	2.57E-05	0	Enoph1
0.402298968	0.363847114	2.73E-05	0	Tmem86a
0.31796721	0.368558964	2.76E-05	0	Tatdn2
-0.332118917	0.374665217	2.81E-05	0	Atf3
0.43900791	0.390636163	2.93E-05	0	Il11ra1
-0.268258704	0.390935847	2.93E-05	0	Mir22hg
0.394105596	0.395205703	2.96E-05	0	Senp5
0.389884044	0.433872328	3.25E-05	0	Gm12942
0.421217237	0.435966347	3.27E-05	0	Cnih1
0.302565564	0.439788329	3.29E-05	0	Galc
0.260606972	0.442129437	3.31E-05	0	Elp5
0.376886887	0.448803934	3.36E-05	0	Cnot4
0.286205633	0.455531107	3.41E-05	0	Stx3
0.40451005	0.455975088	3.42E-05	0	Rce1
0.406018873	0.458441355	3.43E-05	0	Rps6ka4
-0.26769686	0.46433907	3.48E-05	0	Mex3c
0.306526296	0.472477553	3.54E-05	0	Imp4
0.26755383	0.479890871	3.59E-05	0	Plekhj1
-0.556272083	0.48289234	3.62E-05	0	Creb5
0.637840189	0.483522796	3.62E-05	0	Slc30a1
0.30184786	0.486786973	3.65E-05	0	Fundc2
-0.926443015	0.504831699	3.78E-05	0	Nfxl1
-0.738133687	0.507007434	3.80E-05	0	Mt2
0.256294241	0.523519435	3.92E-05	0	Tmem141
0.458458722	0.537117891	4.02E-05	0	Lypla1
0.355151692	0.538251805	4.03E-05	0	Hopx
-0.62169939	0.54574966	4.09E-05	0	Gas7
0.276550651	0.546640717	4.09E-05	0	Mettl7a1
0.336034659	0.551072075	4.13E-05	0	Ccng1
0.251388107	0.571406083	4.28E-05	0	Mrpl50
0.327193573	0.578470236	4.33E-05	0	Map4k3
0.347656994	0.588223511	4.41E-05	0	Arpc3
-0.704277019	0.601633622	4.51E-05	0	Zfhx3
-0.99125043	0.609460991	4.56E-05	0	Nfia
-0.773550401	0.614837784	4.60E-05	0	Arsb
-0.333395553	0.615973693	4.61E-05	0	Rpl29
-0.423102404	0.61967028	4.64E-05	0	Cep170
-0.724702198	0.620685395	4.65E-05	0	Bmp2k
0.259273784	0.632597526	4.74E-05	0	Arl11
-0.479694395	0.64712854	4.85E-05	0	Rnase4
0.285643655	0.661174048	4.95E-05	0	Frrs1
0.258901153	0.662029201	4.96E-05	0	Cyb5
0.292011384	0.670980324	5.03E-05	0	Glrx5
0.413259612	0.67143584	5.03E-05	0	Zfp260
0.251192176	0.68742006	5.15E-05	0	Tmem179b
-0.822206092	0.704235525	5.27E-05	0	Bmyc
-0.5652978	0.709862107	5.32E-05	0	Cep250
0.514721964	0.718508545	5.38E-05	0	Lmf1
-0.63478285	0.752234508	5.63E-05	0	Mtmr12
-0.942285445	0.778303825	5.83E-05	0	Gins4
0.426612643	0.780146047	5.84E-05	0	Cyb5r1
0.276276712	0.784613153	5.88E-05	0	Prkaa1
0.379204229	0.787778385	5.90E-05	0	Mrpl35
0.276391303	0.789902558	5.92E-05	0	Glrx
0.273724657	0.804771633	6.03E-05	0	Psmd10

-0.556417664	0.807039409	6.04E-05	0	Epsti1
-0.513468088	0.808678157	6.06E-05	0	Clip1
0.290854125	0.809762313	6.06E-05	0	Ndufa12
0.320350238	0.821641864	6.15E-05	0	Gab1
-0.47169482	0.825668089	6.18E-05	0	Adap2
-0.740047042	0.829508681	6.21E-05	0	P2ry12
-0.465069574	0.834080268	6.25E-05	0	Gadd45g
0.307059472	0.843491755	6.32E-05	0	Bcl2l13
0.542500994	0.844230559	6.32E-05	0	Dgkz
0.469978146	0.862601463	6.46E-05	0	Amdhd2
-0.698631841	0.874657535	6.55E-05	0	2610507B11Rik
-0.624437588	0.877049269	6.57E-05	0	1700025G04Rik
-0.380833821	0.898746941	6.73E-05	0	Kctd12
-0.532372878	0.938592709	7.03E-05	0	Jdp2
0.411846341	0.951447242	7.13E-05	0	Rufy3
0.450054834	0.96195572	7.20E-05	0	Mrpl55
-0.630418694	0.962785631	7.21E-05	0	Luc7l
0.575190136	0.963154506	7.21E-05	0	Sestd1
0.263190901	0.970333422	7.27E-05	0	Pstpip1
0.342003345	0.989511963	7.41E-05	0	Glb1
-0.550003897	1	7.66E-05	0	Il10ra
-0.572877248	1	7.70E-05	0	Ivns1abp
0.482139058	1	7.78E-05	0	Slc35b1
-0.418685462	1	7.87E-05	0	Ccl7
0.328032607	1	7.90E-05	0	Plekhm2
-0.511969283	1	7.90E-05	0	Acap2
0.571634436	1	8.03E-05	0	Brix1
0.44812846	1	8.05E-05	0	Snx27
-0.644436795	1	8.12E-05	0	Prune2
0.322094515	1	8.13E-05	0	Kxd1
0.335330007	1	8.20E-05	0	Rnf5
-0.303115562	1	8.32E-05	0	Hexb
0.257962354	1	8.37E-05	0	Vps28
-0.714278787	1	8.47E-05	0	Trak1
0.346868286	1	8.68E-05	0	Dock5
-0.433704062	1	8.82E-05	0	Lbr
-0.663341902	1	8.85E-05	0	Akap8l
0.391462245	1	8.94E-05	0	Tusc2
-0.775686076	1	9.13E-05	0	Abcb1b
0.50418292	1	9.14E-05	0	Trnt1
-0.736325002	1	9.17E-05	0	Cyp27a1
-1.066459341	1	9.52E-05	0	Fgfr1
0.327187154	1	9.53E-05	0	Plekhm1
0.347801628	1	9.53E-05	0	Dpp7
0.298270278	1	9.56E-05	0	Pdxk
-0.541999311	1	9.76E-05	0	Noc2l
0.368905362	1	9.96E-05	0	Gatad2a
-0.825181837	1	0.0001006	0	Bbx
0.427603435	1	0.0001029	0	Bloc1s6
-0.384639308	1	0.0001032	0	Rgs2
-0.531488469	1	0.0001037	0	Brd4
0.282557762	1	0.000105	0	Mrpl51
-0.570731423	1	0.0001059	0	Baz1b
0.633940995	1	0.000106	0	Mefv
0.368171103	1	0.0001071	0	Pik3cb
-0.428376218	1	0.0001074	0	Golgb1
0.40506117	1	0.0001077	0	Qpct
0.519774083	1	0.0001081	0	Rnf7
0.283396587	1	0.0001102	0	Pno1
0.266746915	1	0.0001139	0	Mrpl10
0.396354411	1	0.0001164	0	Ufm1

-0.640289378	1	0.0001206	0	Serpinb8
-0.700276581	1	0.0001233	0	Kansl1l
0.309798869	1	0.0001249	0	Gm12060
0.393186834	1	0.0001259	0	Egln1
-0.530508792	1	0.0001273	0	Smarca2
-0.270272483	1	0.0001306	0	Rps21
-0.663799284	1	0.000138	0	Tmcc3
0.294815442	1	0.0001402	0	Tmx1
-0.336676972	1	0.0001426	0	Ifitm3
0.318613326	1	0.0001432	0	Nubp2
0.263924523	1	0.0001461	0	Isca2
0.279101497	1	0.0001503	0	Npepps
-0.910555399	1	0.0001509	0	Mndal
0.258841515	1	0.0001524	0	Yipf6
-0.56706949	1	0.0001532	0	Casc3
-0.456184037	1	0.0001547	0	Pros1
0.288216175	1	0.0001581	0	Cars
-0.553593103	1	0.0001593	0	Smc1a
-0.328140628	1	0.0001596	0	Spred1
-0.633568559	1	0.0001603	0	Ogfrl1
-0.264286767	1	0.0001611	0	Nlrp3
0.328305976	1	0.0001632	0	Hcst
0.379486165	1	0.0001642	0	Casp1
-0.330471053	1	0.0001667	0	Irf2bp2
0.296985932	1	0.0001689	0	Gas2l3
0.35148147	1	0.0001717	0	Mapkapk5
-0.661572518	1	0.0001723	0	Tmem71
-1.000053278	1	0.0001745	0	Gbp7
-1.201903664	1	0.0001768	0	Bank1
0.360212885	1	0.0001793	0	Timm10b
0.263880011	1	0.0001809	0	Hnrnpd
0.25013722	1	0.0001875	0	Man2b2
0.264981201	1	0.0001879	0	Idua
0.417365384	1	0.0001894	0	Emc4
0.417559837	1	0.0001914	0	Atp11b
-0.672109869	1	0.0001922	0	Ccdc22
0.314444172	1	0.0001963	0	Comtd1
0.362923314	1	0.0001965	0	Golph3l
-0.639385196	1	0.0002016	0	Tbc1d9b
-0.343545043	1	0.000204	0	Hcls1
-0.635230152	1	0.0002049	0	Zfp513
0.376772464	1	0.0002053	0	Snrpa1
-0.651997382	1	0.0002103	0	Agpat6
0.399903448	1	0.000215	0	Inf2
0.389150364	1	0.0002152	0	Akr1b8
0.261389435	1	0.0002162	0	Tor1aip2
0.360792055	1	0.0002216	0	Vps54
-0.828949693	1	0.0002239	0	Ppfia4
0.256365446	1	0.0002254	0	Rcbtb2
0.405500158	1	0.0002334	0	Srxn1
0.332037938	1	0.0002335	0	Aip
0.49986473	1	0.0002357	0	Plgrkt
-0.289745673	1	0.000239	0	Ly86
0.255883208	1	0.0002395	0	Pon3
0.304044434	1	0.0002408	0	Ppp6c
0.376491048	1	0.0002472	0	Psmb7
0.254670871	1	0.0002497	0	Slc38a6
0.303620933	1	0.000261	0	Wdr41
0.300065083	1	0.0002626	0	Pgk1
-0.744670723	1	0.0002634	0	Adrb2
-0.361899768	1	0.0002634	0	P2rx7

0.598960284	1	0.0002706	0	Gipc1
-0.52183751	1	0.0002784	0	Slc2a6
-0.565926531	1	0.0002811	0	Wwp1
-0.576174809	1	0.0002817	0	Xrn1
0.361515911	1	0.0002818	0	Ict1
0.316221396	1	0.0002847	0	Triap1
0.426960761	1	0.0002868	0	Tspan5
0.266088396	1	0.0003016	0	Tmem14c
-0.463599974	1	0.0003024	0	Aes
-1.03700168	1	0.0003066	0	Batf3
0.344415731	1	0.000307	0	Rad23a
0.259725484	1	0.0003071	0	Agpat2
-0.702761796	1	0.0003203	0	Elf1
-0.569973451	1	0.0003213	0	Ythdc1
0.292336565	1	0.0003272	0	Tmem33
-1.063421043	1	0.0003308	0	Top2a
-0.300675247	1	0.000334	0	Macf1
0.615951759	1	0.0003351	0	Cstf3
-0.620540437	1	0.0003364	0	Aak1
0.406448862	1	0.0003372	0	Nfil3
-0.830165384	1	0.0003404	0	Pcnt
0.301216314	1	0.0003435	0	Srp19
0.255560247	1	0.0003478	0	Prorsd1
-0.420106566	1	0.0003547	0	Dek
-0.607138629	1	0.0003624	0	Smc6
-0.604823595	1	0.0003684	0	Eps15
0.345738131	1	0.000369	0	Lgals8
-0.445261932	1	0.0003719	0	Nfkb1
0.269502393	1	0.0003763	0	Txndc9
0.305067016	1	0.0003785	0	Mrps7
-0.720422324	1	0.0003825	0	Zfp68
0.263580981	1	0.0003914	0	Vps25
0.273563031	1	0.0004056	0	Babam1
0.293695411	1	0.0004245	0	Mif
0.288498881	1	0.0004256	0	Mrpl24
-0.482371122	1	0.0004325	0	Vrk1
-0.349848312	1	0.000436	0	Dock8
-0.493815463	1	0.0004364	0	Lrmp
-0.627909198	1	0.000437	0	Ophn1
-0.344874848	1	0.0004468	0	Trmt1
-0.485386664	1	0.0004533	0	Hivep1
-0.528304412	1	0.0004635	0	P2ry6
0.474559678	1	0.0004674	0	Sik3
-0.493190278	1	0.0004769	0	Dclre1c
0.261638189	1	0.0004778	0	Ttc7
-0.504963088	1	0.0004939	0	Glg1
-0.600040748	1	0.0004959	0	Slc4a7
-0.544783387	1	0.0005021	0	Exoc6b
-0.698783126	1	0.0005063	0	Rnf216
-0.557221883	1	0.0005121	0	Peli1
-0.479109865	1	0.0005232	0	Sema4a
-0.514052275	1	0.0005232	0	Csk
-0.674147039	1	0.0005309	0	Swap70
-0.424467902	1	0.0005313	0	Cxcl1
0.315923525	1	0.0005337	0	Dpcd
0.26984628	1	0.0005343	0	Sfxn1
-0.379141804	1	0.0005351	0	Brd2
0.26869076	1	0.0005398	0	Timm17b
0.305565231	1	0.0005405	0	Ndufs1
0.559742563	1	0.0005501	0	Sdhaf2
0.297069994	1	0.0005543	0	Cebpzos

-0.686126972	1	0.0005562	0	Acvrl1
-0.582596082	1	0.0005777	0	Tnfaip8
0.291752331	1	0.0005886	0	Nol12
0.347546233	1	0.0005914	0	Rai14
0.398905774	1	0.0005956	0	Eaf1
-0.765923933	1	0.0006016	0	Per1
0.675345831	1	0.0006071	0	Pop7
-0.286570652	1	0.0006086	0	Aim2
-0.716349116	1	0.0006118	0	Pygo2
-0.594027448	1	0.000614	0	Man1a
-0.400559867	1	0.0006219	0	Pcp4l1
0.725894129	1	0.0006318	0	Arrdc4
-0.691874353	1	0.0006393	0	Ebi3
-0.572401128	1	0.0006433	0	Uvrag
-0.499509965	1	0.0006756	0	Plrg1
-0.516973462	1	0.0006878	0	Pdlim1
0.279946883	1	0.0006917	0	Psme3
-0.467931057	1	0.0006986	0	Sp110
0.308725463	1	0.0007018	0	Dnpep
0.465681339	1	0.0007122	0	1110057K04Rik
0.490839967	1	0.0007219	0	Med10
-0.806490335	1	0.0007527	0	Cwf19l2
-0.432157422	1	0.0007537	0	Nsmce1
0.457716645	1	0.0007552	0	Exosc9
-0.417194841	1	0.000772	0	Fnbp1
0.333311336	1	0.0007941	0	Pafah1b2
0.312998539	1	0.0007941	0	Dnttip1
0.335431547	1	0.0007945	0	Zfp148
0.289890318	1	0.0008003	0	Lamtor3
0.29288158	1	0.0008138	0	Ncbp1
-0.514679362	1	0.0008307	0	Ccdc88b
0.372911357	1	0.0008609	0	Emc6
-0.610250842	1	0.0008798	0	Arrb1
-0.282783466	1	0.0008807	0	Atrx
-0.528611032	1	0.0008869	0	Phactr4
0.273764466	1	0.0008959	0	Ggta1
0.438169949	1	0.0009092	0	Samm50
-0.825602501	1	0.0009303	0	Pla2g4a
-0.545521084	1	0.0009381	0	Plbd1
-0.615752394	1	0.0009594	0	Tnrc18
0.252699204	1	0.0009635	0	Haghl
0.329030742	1	0.000996	0	Ift46
0.339236535	1	0.0009961	0	Hs2st1
0.317695489	1	0.0009992	0	Psmc5
-0.586850273	1	0.0010032	0	Coro7
-0.577637944	1	0.0010052	0	Tbc1d5
0.25032954	1	0.0010137	0	Tmem126a
0.343166477	1	0.0010188	0	Nkiras2
0.266772061	1	0.0010294	0	Cdc34
-0.631861909	1	0.0010415	0	Dpysl2
0.346530988	1	0.0010444	0	Srprb
0.53539457	1	0.0010624	0	Mrpl32
-0.263978645	1	0.0010707	0	Ddx3x
0.268124065	1	0.001089	0	Rnf41
-0.272612629	1	0.0011245	0	Atp2b1
-0.591128126	1	0.0011297	0	Def6
0.265709537	1	0.0011553	0	Ccdc127
-0.621266904	1	0.0011768	0	Ccdc50
-0.379065384	1	0.001179	0	Sp3
-0.684857959	1	0.0011867	0	Batf
0.322192634	1	0.0012328	0	Uso1

0.555009404	1	0.001237	0	Galk2
-1.274827607	1	0.0012733	0	Mgp
-0.290175247	1	0.0012757	0	Lcp2
-0.546708156	1	0.0012782	0	Camk1d
-0.52040718	1	0.0012879	0	Dennd5a
0.576343001	1	0.0012901	0	0610010F05Rik
-0.462672471	1	0.001302	0	Snx20
0.265381953	1	0.0013163	0	Rtfdc1
0.279547696	1	0.0013285	0	Deb1
0.257485532	1	0.0013328	0	Unc50
-0.437850682	1	0.0013358	0	Sec24b
0.332369154	1	0.0013394	0	Vapb
0.327250514	1	0.0013617	0	Mrpl27
-0.292513816	1	0.0013622	0	Ly6e
0.253803976	1	0.001369	0	Mroh1
0.347225113	1	0.0013693	0	Atp11a
-0.815316688	1	0.0013838	0	Ip6k1
-0.671140519	1	0.001392	0	Gtpbp1
-0.469772724	1	0.0013987	0	Errfi1
0.350204934	1	0.0014424	0	Ergic1
-0.451369114	1	0.0014527	0	Smad1
-0.435841011	1	0.0014625	0	Fam168a
-0.847926579	1	0.0014725	0	P2ry10
-0.262844707	1	0.001477	0	Pfdn5
-0.443940515	1	0.0014918	0	Zbtb2
-0.667709225	1	0.001519	0	Enox2
-0.307138875	1	0.0015242	0	Inpp5b
-0.621484625	1	0.0015668	0	Lacc1
0.308072637	1	0.0015999	0	Ttc7b
0.308459883	1	0.0016226	0	Golph3
-0.601200771	1	0.0016241	0	Pcna
-0.28797882	1	0.001627	0	Lpcat2
-0.403149311	1	0.0016302	0	Slc15a4
-0.541228836	1	0.0016403	0	Hfe
-0.642501644	1	0.0016606	0	Mcm6
-0.325284196	1	0.0016614	0	Tmem63a
0.324655909	1	0.0017211	0	Klhdc4
-0.544155519	1	0.0017396	0	Zcchc11
-0.481596262	1	0.0017776	0	Grk6
0.262938173	1	0.0017857	0	Llph
0.258861634	1	0.0018006	0	Csrp2bp
-0.548596583	1	0.0018267	0	Tmem109
-0.516714907	1	0.0018368	0	Fhl3
-0.827858105	1	0.0018529	0	Glcci1
0.306608194	1	0.0018635	0	Rars
-0.593422674	1	0.0018895	0	Pak1
0.253366408	1	0.0018951	0	Chmp4b
0.298219462	1	0.0019185	0	Rbms1
-0.690801795	1	0.0019211	0	Sgpp1
-0.584958763	1	0.0019444	0	Trip10
-0.462155696	1	0.0019581	0	Mknk1
-0.266308597	1	0.002036	0	Cyth4
0.312454913	1	0.0021126	0	Yrdc
-0.418887865	1	0.002147	0	Ctbp2
-0.494468972	1	0.0021813	0	Dctpp1
-0.507587699	1	0.0021941	0	Sorbs3
0.267568462	1	0.0022056	0	Rpl35
-0.445742191	1	0.0022138	0	Prkacb
0.268330108	1	0.0022354	0	Ndufb4
0.358547107	1	0.0022496	0	Adi1
-0.461129664	1	0.002253	0	Uba7

0.26026916	1	0.0022757	0	Znrf1
-0.776176223	1	0.0023094	0	Zmynd15
0.358430414	1	0.0023159	0	Slc31a1
0.326879923	1	0.00232	0	Akr7a5
0.416649701	1	0.0023604	0	Nqo2
-0.517764439	1	0.0023716	0	Oxsr1
-0.496168497	1	0.0024193	0	Snx2
-0.439590781	1	0.0024236	0	Kmt2e
-0.523074462	1	0.0024285	0	Hivep2
-0.506909985	1	0.0024319	0	Phf20
0.559999293	1	0.0024394	0	Ubxn8
0.285668911	1	0.0024488	0	Arfip1
-0.441936825	1	0.0024652	0	Zcchc6
0.296791238	1	0.002468	0	Mrps26
-0.402297337	1	0.0024834	0	Stap1
0.474141568	1	0.0025141	0	B4galt3
0.338540851	1	0.0025299	0	Bola3
0.330850315	1	0.0025701	0	1700123O20Rik
0.278393459	1	0.0025712	0	Tmem11
0.259314733	1	0.002574	0	Galnt7
-0.362261204	1	0.0026119	0	Prps2
-0.433815783	1	0.0026327	0	2810025M15Rik
-0.46968504	1	0.002645	0	Itsn2
0.360119585	1	0.0027599	0	Nlrc5
-0.637031125	1	0.0027728	0	Fam53b
-0.45654561	1	0.0027759	0	Acsl1
-0.538890435	1	0.0028244	0	Frmd4a
-0.274115135	1	0.0028538	0	Ddx6
-0.407527734	1	0.0028996	0	Rbpj
0.375537678	1	0.002921	0	Dock7
-0.514379615	1	0.0030008	0	Vps16
0.38973889	1	0.0030808	0	Mrpl40
0.269534282	1	0.003084	0	Btf3
0.571204448	1	0.0030848	0	Phf8
0.375555619	1	0.0031303	0	Mnat1
-0.400227381	1	0.003198	0	Aif1
0.280150099	1	0.0032208	0	Nt5dc3
0.634401579	1	0.0032685	0	Sdad1
-0.419566842	1	0.0033668	0	Bin1
-0.45347116	1	0.0034355	0	Mat2a
-0.305411784	1	0.0034708	0	Cnot1
0.531102133	1	0.0034811	0	Nek6
-0.514314367	1	0.0035034	0	Yars
-0.635777643	1	0.0035519	0	Dnajb1
-0.38374467	1	0.0035576	0	Ubtf
-0.509484211	1	0.0036104	0	Rhob
0.486867628	1	0.0036846	0	Abhd5
0.399075959	1	0.0036999	0	Uri1
0.265205344	1	0.0037913	0	Fbxo30
0.354003916	1	0.0038006	0	Odc1
-0.405233377	1	0.0038327	0	Tmx4
0.267249476	1	0.0038414	0	Cox19
-0.573177379	1	0.0038562	0	Ier2
-0.632761242	1	0.0038641	0	Sin3a
-0.739789648	1	0.0038667	0	Taf15
-0.305970359	1	0.0039109	0	Smndc1
-0.402484105	1	0.0039263	0	Rnf145
-0.65461366	1	0.0039543	0	Vps13a
-0.29031555	1	0.0040221	0	Ascc1
-0.471320803	1	0.004029	0	Cr1l
0.513125608	1	0.0040481	0	Tsr1

0.272430049	1	0.0040585	0	Scarb1
-0.301053225	1	0.0040804	0	Acss1
-0.373733807	1	0.0041792	0	Osgep
-0.496709039	1	0.0041793	0	Nav1
0.350560177	1	0.0042019	0	Lamtor2
-0.339523846	1	0.0042415	0	Arhgap22
0.334506523	1	0.0043017	0	Znrf2
-0.533449726	1	0.0043162	0	Eva1b
0.300358285	1	0.0043824	0	Fam213b
0.407233319	1	0.0044227	0	Uba3
0.271108862	1	0.0044455	0	Sf3b4
0.26800315	1	0.0044655	0	Ctnnbip1
0.363974362	1	0.004466	0	Dram1
-0.563268778	1	0.004502	0	Smarca4
-0.441981341	1	0.0046958	0	Sec14l1
0.297362565	1	0.004808	0	Prosc
-0.350423342	1	0.0048121	0	Mef2a
-0.356480949	1	0.0048128	0	Cpsf2
-0.3385592	1	0.0048337	0	Phactr2
0.25455191	1	0.0049097	0	Mrpl57
-0.467690046	1	0.0049641	0	Fam134b
-0.276043333	1	0.0050268	0	Tpr
-1.22701916	1	0.0050986	0	Plxdc2
0.289527932	1	0.0051082	0	Rspry1
-0.53739072	1	0.0051336	0	Arhgap12
-0.56232899	1	0.0052111	0	Eftud2
-0.502397329	1	0.0052753	0	Jade2
-0.321713177	1	0.0054158	0	Cct5
-0.507088621	1	0.0054237	0	E030024N20Rik
-0.46722276	1	0.0055019	0	Uap1
-0.258917418	1	0.0056551	0	Hbs1l
-0.996290521	1	0.0056719	0	Ly6a
-0.449303163	1	0.0058548	0	Kmt2c
-0.541468151	1	0.0058613	0	Nr3c1
0.342329542	1	0.0059852	0	Pdzd11
-0.378965409	1	0.0060279	0	Psmb8
0.375984861	1	0.0060453	0	Rptor
-0.628366587	1	0.0060574	0	Cbfa2t3
-0.33191762	1	0.0060634	0	Rbbp8
-0.494016981	1	0.0060802	0	Kdm3b
0.266219029	1	0.0060914	0	Tufm
-0.64651655	1	0.0061667	0	Cspp1
-0.485159527	1	0.0063909	0	Fam178a
-0.46716159	1	0.0064376	0	Nbeal1
0.385101729	1	0.0065849	0	Zc3h12a
0.256105629	1	0.0066325	0	Mvp
-0.260659512	1	0.0070161	0	Nars
-0.511770369	1	0.0070478	0	Cebpd
-0.324734065	1	0.0070493	0	Sltm
-0.388657829	1	0.0070665	0	Bcl6
-0.445482773	1	0.0070765	0	Ep300
-0.255174018	1	0.0071074	0	Rraga
-0.4895127	1	0.007166	0	Ddx41
-0.640737989	1	0.0071681	0	Arhgap18
-0.482474962	1	0.0074961	0	Relb
-0.395446779	1	0.0076329	0	Notch2
-0.554671465	1	0.0077255	0	Rere
-0.40506747	1	0.0077319	0	Pde4b
-0.273876555	1	0.0077473	0	Top1
0.47098096	1	0.0077501	0	Msmo1
-0.466139923	1	0.0080445	0	Ralbp1

-0.797839281	1	0.0080549	0	Apoe
0.322881986	1	0.0080628	0	Ddrgk1
-0.282028981	1	0.0081072	0	Mir703
-0.466149557	1	0.0081214	0	Zfml
0.359048417	1	0.0081589	0	Polr2b
-0.480229183	1	0.0081709	0	Brd3
0.392497621	1	0.0082546	0	Abhd17c
-0.51756482	1	0.0084362	0	Ash1l
-0.427935907	1	0.0084838	0	Brd9
0.382609673	1	0.0085133	0	Glrx2
-0.40288444	1	0.0085776	0	Ptpra
0.319258666	1	0.0086215	0	Gramd1b
0.421351841	1	0.0088134	0	Tmem251
-0.356512646	1	0.0089323	0	Wdr48
0.274807866	1	0.0089744	0	Rasa3
-0.759477559	1	0.009119	0	D16Ertd472e
-0.503384754	1	0.0091649	0	Pabpn1
-0.314230141	1	0.0091788	0	Pik3c2a
-0.25076218	1	0.0091835	0	Gm6251
-0.434167812	1	0.0091942	0	Tmod3
-0.400554535	1	0.0092694	0	Vaultrc5
-0.275202143	1	0.009293	0	Mtmr10
0.25140719	1	0.0093062	0	Fbxo22
-0.554654749	1	0.0093271	0	Rcan1
-0.60487966	1	0.0095042	0	Soga1
-0.502927929	1	0.0095917	0	Cep83
-0.394210421	1	0.0096332	0	Tcof1
-0.794360424	1	0.0096935	0	Dennd4a
-0.488372166	1	0.0097958	0	Arhgap15
0.325168366	1	0.0098141	0	Lemd2
-0.294561274	1	0.0099147	0	Rac2
-0.528722551	1	0.0099499	0	Ankrd44
-0.463610082	1	0.0099552	0	Arid5a
0.80130862	1.53E-38	1.14E-42	1	Cd74
0.846427568	1.34E-34	1.00E-38	1	H2-Eb1
0.710850428	1.80E-30	1.35E-34	1	H2-Ab1
0.972729251	1.41E-26	1.06E-30	1	Ccl4
0.686657317	8.47E-26	6.35E-30	1	H2-Aa
-1.293094635	1.98E-22	1.48E-26	1	Spp1
-1.730985387	4.00E-20	3.00E-24	1	Gpnmb
-0.580040233	3.18E-19	2.38E-23	1	Ctsd
1.003755776	5.53E-19	4.14E-23	1	Ccl3
-1.099077077	7.53E-19	5.64E-23	1	Pld3
-1.561728094	1.38E-18	1.03E-22	1	Adam8
-1.650421099	4.28E-18	3.21E-22	1	Syngr1
-0.712613211	4.51E-18	3.38E-22	1	Cstb
-0.849074954	1.52E-17	1.14E-21	1	Psmb6
-1.362032921	2.30E-17	1.72E-21	1	Il7r
-0.757885954	9.28E-17	6.95E-21	1	Rnh1
1.328646899	1.82E-16	1.36E-20	1	Cxcl10
0.295880356	2.07E-16	1.55E-20	1	Malat1
-0.838518598	2.23E-16	1.67E-20	1	Emp1
-0.761336341	3.12E-16	2.33E-20	1	Ndufc1
-0.65269054	1.00E-15	7.50E-20	1	1110007C09Rik
0.320161076	3.20E-15	2.40E-19	1	C1qb
0.659043156	4.36E-15	3.26E-19	1	Tmem176b
-0.727492069	4.61E-15	3.45E-19	1	0610031J06Rik
-0.488646752	8.18E-15	6.13E-19	1	Lgals1
-0.625802067	8.57E-15	6.42E-19	1	Cox6a1
-0.766925679	8.63E-15	6.47E-19	1	Cox7b
-0.476086309	9.35E-15	7.00E-19	1	Aprt

-0.671827966	9.59E-15	7.18E-19	1	Bhlhe40
-0.710746708	1.38E-14	1.04E-18	1	Ndufa1
-0.729623367	4.49E-14	3.36E-18	1	Osbpl8
-0.965342486	5.46E-14	4.09E-18	1	Clec4d
-0.83306057	1.43E-13	1.07E-17	1	Ndufb6
-1.038515257	2.31E-13	1.73E-17	1	Csf2rb2
0.455670566	4.54E-13	3.40E-17	1	Serinc3
-0.729852636	6.49E-13	4.86E-17	1	Nme1
-0.693949431	7.24E-13	5.42E-17	1	Esd
-0.488463705	8.54E-13	6.40E-17	1	Anxa2
0.588050728	8.99E-13	6.73E-17	1	Zfp36
1.009179971	1.03E-12	7.69E-17	1	Egr1
-0.690356783	1.10E-12	8.26E-17	1	Anxa4
-0.516374064	1.12E-12	8.40E-17	1	Hint1
-0.544514433	1.63E-12	1.22E-16	1	Mpc2
-0.798091855	2.27E-12	1.70E-16	1	Gpr137b
-0.62009282	2.85E-12	2.13E-16	1	S100a4
-0.4874448	5.44E-12	4.07E-16	1	Ndufs6
-0.674438447	6.95E-12	5.20E-16	1	Dnajc15
-0.605676306	8.25E-12	6.18E-16	1	S100a6
0.531293309	1.33E-11	9.97E-16	1	Klf6
-0.76910471	1.37E-11	1.02E-15	1	Tspan4
-0.783559938	2.23E-11	1.67E-15	1	Gstm1
-0.706166695	4.49E-11	3.37E-15	1	Sec11c
-0.650188069	4.50E-11	3.37E-15	1	Ecm1
-0.586294906	5.03E-11	3.77E-15	1	Atp6v1c1
-1.71484523	7.83E-11	5.87E-15	1	Ctsk
-0.506836728	8.62E-11	6.46E-15	1	Tnfaip2
-0.979630292	9.10E-11	6.81E-15	1	Galnt6
-0.709129817	9.15E-11	6.85E-15	1	Aurkaip1
-0.919500688	1.01E-10	7.60E-15	1	Yif1b
0.432617144	1.35E-10	1.01E-14	1	Junb
-0.527073017	1.45E-10	1.09E-14	1	Txndc17
-0.780856357	1.75E-10	1.31E-14	1	Tgm2
-0.417292226	1.83E-10	1.37E-14	1	Lgals3
-0.663420643	2.67E-10	2.00E-14	1	Anxa1
-1.014752674	3.12E-10	2.34E-14	1	Scd2
-0.522683223	3.15E-10	2.36E-14	1	Cox7a2
-1.020155559	3.42E-10	2.56E-14	1	Grpel1
-0.700436815	3.56E-10	2.66E-14	1	Fam129b
-0.497528732	3.58E-10	2.68E-14	1	Creg1
-0.759675614	5.50E-10	4.12E-14	1	Lpl
-0.552429521	5.69E-10	4.26E-14	1	Atp5j
-0.651865776	5.96E-10	4.46E-14	1	1810058I24Rik
-1.15079086	6.12E-10	4.58E-14	1	Acot13
-0.53128111	6.47E-10	4.85E-14	1	Dusp3
-0.530609744	7.00E-10	5.24E-14	1	Abcg1
-0.898460807	8.78E-10	6.58E-14	1	Slc48a1
-0.934861668	1.03E-09	7.72E-14	1	Timm17a
-0.527975761	1.19E-09	8.93E-14	1	Capg
-0.579594834	1.20E-09	8.96E-14	1	Mrpl54
-1.21285893	1.69E-09	1.27E-13	1	Slc6a8
-0.522287216	1.74E-09	1.30E-13	1	Cox5a
0.762265069	1.84E-09	1.38E-13	1	H2-DMa
-0.522317684	2.03E-09	1.52E-13	1	Tmem256
-0.462156304	2.50E-09	1.87E-13	1	Aldoa
-0.775769971	4.47E-09	3.35E-13	1	Prdx6
-0.531691748	4.59E-09	3.43E-13	1	Timm13
-0.715348223	5.48E-09	4.11E-13	1	Lhfpl2
-0.562976591	7.67E-09	5.75E-13	1	Lipa
-0.679896758	8.54E-09	6.40E-13	1	Polr2e

-0.493032391	9.11E-09	6.82E-13	1	Gm12338
-0.355442887	9.52E-09	7.13E-13	1	Mrps24
-0.438129886	1.24E-08	9.30E-13	1	Ndufa3
-0.309544564	1.38E-08	1.04E-12	1	Nedd8
-0.570730724	1.67E-08	1.25E-12	1	Nrp2
-0.405902703	2.20E-08	1.65E-12	1	Capns1
-0.423638162	2.22E-08	1.66E-12	1	Uqcr11
-0.513228224	2.39E-08	1.79E-12	1	Tmem258
-0.569513962	2.63E-08	1.97E-12	1	Dtnbp1
-0.644498129	2.90E-08	2.17E-12	1	Ndufa9
-0.463918002	2.98E-08	2.23E-12	1	Ssr4
-0.579484794	3.11E-08	2.33E-12	1	Nagk
-0.490574439	3.17E-08	2.37E-12	1	S100a10
-0.566704476	3.62E-08	2.71E-12	1	Elp5
-0.570241527	3.76E-08	2.82E-12	1	Gdf15
-0.625993288	3.89E-08	2.91E-12	1	Dap
-1.346720204	4.76E-08	3.57E-12	1	Fabp5
-0.399170565	4.90E-08	3.67E-12	1	Ldha
-0.663983311	5.00E-08	3.74E-12	1	Cytip
-0.497710617	5.43E-08	4.07E-12	1	Atp6v0d1
-0.398121401	5.45E-08	4.08E-12	1	Bola2
-0.495187082	5.72E-08	4.28E-12	1	Atp6v1d
-0.40348996	6.35E-08	4.76E-12	1	Uqcr10
-0.493484201	6.62E-08	4.96E-12	1	Ap2s1
-0.878436488	6.74E-08	5.05E-12	1	Mob4
-0.361720369	7.85E-08	5.88E-12	1	Surf4
-0.408691116	8.05E-08	6.03E-12	1	Gpr137b-ps
-0.611005247	8.06E-08	6.04E-12	1	Pgls
0.441610343	8.80E-08	6.59E-12	1	Mrc1
-0.507420075	9.16E-08	6.86E-12	1	Trem2
-0.373273956	9.41E-08	7.05E-12	1	Ywhag
-0.993400051	9.54E-08	7.14E-12	1	Fbxo32
0.466354727	1.02E-07	7.66E-12	1	Fos
-0.381556998	1.08E-07	8.06E-12	1	Ctbp1
-0.652620351	1.08E-07	8.07E-12	1	Ech1
-0.513247153	1.09E-07	8.20E-12	1	Hsbp1
-0.394106133	1.20E-07	8.98E-12	1	Rnasek
-0.5607455	1.22E-07	9.14E-12	1	Mrpl20
-0.518523236	1.32E-07	9.90E-12	1	Ndufb7
-0.669087633	1.39E-07	1.04E-11	1	Rnf128
-1.331130328	1.39E-07	1.04E-11	1	Igf2r
-0.728026814	1.55E-07	1.16E-11	1	Fundc2
-0.522924104	1.62E-07	1.21E-11	1	Cox17
-1.172504333	1.64E-07	1.23E-11	1	Pdpn
0.442828988	1.91E-07	1.43E-11	1	Dusp1
-0.513729911	2.03E-07	1.52E-11	1	Gusb
-1.418859854	2.09E-07	1.56E-11	1	Atp6v0d2
-0.397148643	2.32E-07	1.74E-11	1	Pkm
-0.475028013	2.37E-07	1.77E-11	1	Polr2f
-0.461288435	2.45E-07	1.83E-11	1	Itgax
-0.481778564	2.75E-07	2.06E-11	1	Ddx46
-0.253049079	2.81E-07	2.11E-11	1	Spcs1
-0.517210931	2.97E-07	2.22E-11	1	Cox5b
-0.606579743	3.00E-07	2.25E-11	1	Sars
-0.463915041	3.09E-07	2.31E-11	1	Tmem208
1.195396292	3.36E-07	2.52E-11	1	Ccr2
1.072210889	3.46E-07	2.59E-11	1	Hspa1b
-0.623367257	3.53E-07	2.64E-11	1	Ndufb9
-0.565190257	3.61E-07	2.71E-11	1	Ssu72
-0.515802081	3.62E-07	2.71E-11	1	Plec
0.366242345	3.83E-07	2.87E-11	1	Cybb

-0.487318773	3.84E-07	2.87E-11	1	Krtcap2
-0.620327395	3.96E-07	2.97E-11	1	Csf2rb
-1.244696292	4.07E-07	3.05E-11	1	Rgcc
-0.557787555	4.09E-07	3.06E-11	1	Mmadhc
0.559851387	4.09E-07	3.07E-11	1	Atf3
-0.747380424	4.31E-07	3.23E-11	1	Malt1
-0.384411423	4.46E-07	3.34E-11	1	Fam20c
-0.768533208	4.63E-07	3.46E-11	1	Lonrf3
-0.77661566	4.84E-07	3.62E-11	1	Msrb1
-0.639896368	5.48E-07	4.11E-11	1	Iqsec1
0.508604069	5.51E-07	4.13E-11	1	Unc93b1
-0.32395746	6.27E-07	4.70E-11	1	Fkbp2
-0.739459416	6.37E-07	4.77E-11	1	Mrpl43
-0.454456755	6.38E-07	4.78E-11	1	Cope
-0.56706595	6.67E-07	4.99E-11	1	Slc37a2
-0.28809256	6.77E-07	5.07E-11	1	Degs1
-0.518079741	7.92E-07	5.93E-11	1	Serpinb6a
-0.654482002	8.30E-07	6.22E-11	1	Mrps7
-0.566955079	8.47E-07	6.34E-11	1	Vps25
-0.440434178	8.65E-07	6.48E-11	1	Taldo1
-0.462341393	1.04E-06	7.79E-11	1	Smpdl3a
1.127948332	1.18E-06	8.85E-11	1	Lilra5
0.565535193	1.19E-06	8.92E-11	1	Cxcl2
-1.738228057	1.20E-06	8.97E-11	1	Npy
-0.462319685	1.24E-06	9.28E-11	1	Ctsl
-0.386064207	1.25E-06	9.35E-11	1	Atp5g3
-0.475586463	1.36E-06	1.02E-10	1	Vps29
-0.421629409	1.65E-06	1.24E-10	1	Psma1
-0.427808144	1.66E-06	1.24E-10	1	Tmem160
-0.408922299	1.83E-06	1.37E-10	1	Trappc2l
-0.746709991	1.83E-06	1.37E-10	1	Acot7
0.778659812	1.85E-06	1.38E-10	1	Hspa1a
-0.450937542	1.97E-06	1.48E-10	1	Ninj1
-0.278039449	2.08E-06	1.56E-10	1	Park7
-0.512763568	2.21E-06	1.66E-10	1	Lasp1
-0.686096199	2.23E-06	1.67E-10	1	Trappc1
-0.325577741	2.23E-06	1.67E-10	1	Mrpl12
-0.377883923	2.38E-06	1.78E-10	1	Psmb4
-0.294757628	2.51E-06	1.88E-10	1	Mdh2
-0.773536906	2.59E-06	1.94E-10	1	Actn1
-0.381404165	2.66E-06	1.99E-10	1	Cd63
-0.519004823	2.93E-06	2.20E-10	1	Naa38
-0.511665884	2.93E-06	2.20E-10	1	Psmc4
-0.309550784	3.02E-06	2.26E-10	1	Psmc5
-0.301348553	3.04E-06	2.27E-10	1	Emilin2
-0.537802257	3.08E-06	2.30E-10	1	S100a1
-0.3741362	3.15E-06	2.36E-10	1	Ndufb8
-0.353568444	3.19E-06	2.39E-10	1	Chchd1
0.481920936	3.25E-06	2.43E-10	1	Hexb
-0.400426598	3.44E-06	2.58E-10	1	Cyb5r3
-0.582027545	3.56E-06	2.66E-10	1	Tmem167
-0.468012466	3.74E-06	2.80E-10	1	Cib1
-0.414909499	3.85E-06	2.89E-10	1	Txn1
-0.629470347	4.06E-06	3.04E-10	1	Gabarapl1
-0.355838295	4.27E-06	3.20E-10	1	Mrps18c
-0.38917107	4.32E-06	3.24E-10	1	Slc9a3r1
-0.307293142	4.32E-06	3.24E-10	1	Vat1
-0.523528347	4.38E-06	3.28E-10	1	Ndufs8
-0.323354572	4.71E-06	3.53E-10	1	Uqcrc2
-0.440986537	5.06E-06	3.79E-10	1	Soat1
-0.450787764	5.20E-06	3.89E-10	1	Ugp2

-0.528345258	5.32E-06	3.98E-10	1	Gsto1
-0.388476441	5.32E-06	3.99E-10	1	Ndufb2
-0.27773752	5.46E-06	4.09E-10	1	Minos1
-0.620795187	5.47E-06	4.10E-10	1	Ndufs4
-0.423478973	5.80E-06	4.34E-10	1	Lpp
-1.156558501	5.86E-06	4.39E-10	1	P2ry10
-0.424062072	6.23E-06	4.66E-10	1	Lrpap1
-0.907615534	6.27E-06	4.70E-10	1	Hilpda
0.269538313	6.60E-06	4.94E-10	1	Hspa8
-0.371972645	6.77E-06	5.07E-10	1	Ptplad2
-0.321526975	7.25E-06	5.43E-10	1	Mtss1
-0.496033102	8.55E-06	6.40E-10	1	Actr1a
-0.385126024	8.69E-06	6.51E-10	1	Myof
-0.292249053	8.74E-06	6.55E-10	1	Prdx2
-0.565257216	8.87E-06	6.64E-10	1	Cuta
-0.503663512	9.08E-06	6.80E-10	1	Yif1a
-0.349832698	9.25E-06	6.93E-10	1	Gtf2h5
-0.383559864	9.28E-06	6.95E-10	1	Hspa9
-0.389228685	9.35E-06	7.00E-10	1	Ckap4
-0.349003531	9.71E-06	7.27E-10	1	Cct2
-0.331322537	9.73E-06	7.29E-10	1	Akr1a1
0.91249635	1.05E-05	7.87E-10	1	Tmem176a
-0.45167528	1.08E-05	8.06E-10	1	Vimp
-0.368585362	1.08E-05	8.07E-10	1	Atp6v0e
-0.900127296	1.13E-05	8.47E-10	1	Fam134a
-0.458356304	1.15E-05	8.62E-10	1	Pcyt1a
-0.604100201	1.16E-05	8.66E-10	1	Aip
-0.544062908	1.27E-05	9.51E-10	1	Cct6a
-0.29194692	1.29E-05	9.63E-10	1	Coa3
-0.717959468	1.30E-05	9.72E-10	1	Mrpl36
-0.400045622	1.31E-05	9.84E-10	1	Aldh2
-0.349707171	1.33E-05	9.94E-10	1	Tax1bp1
-0.287061352	1.41E-05	1.05E-09	1	2010107E04Rik
-0.483727469	1.46E-05	1.10E-09	1	Galns
-0.499711664	1.48E-05	1.11E-09	1	Fnip2
-0.382445356	1.66E-05	1.24E-09	1	Eif3i
0.485795433	1.70E-05	1.28E-09	1	Cd81
-0.381588863	1.79E-05	1.34E-09	1	Atp6v1e1
-0.461288487	1.84E-05	1.38E-09	1	Zfand2a
-0.604445034	1.90E-05	1.42E-09	1	Prpsap1
0.283610174	1.96E-05	1.47E-09	1	Sepp1
-0.700193335	2.20E-05	1.65E-09	1	Ccdc107
0.64081162	2.23E-05	1.67E-09	1	Rhob
-0.343875756	2.24E-05	1.68E-09	1	Mrpl42
-0.435820722	2.29E-05	1.71E-09	1	Nceh1
-0.530904724	2.29E-05	1.71E-09	1	Abhd5
-0.830621474	2.33E-05	1.74E-09	1	Tmem65
-1.201831992	2.51E-05	1.88E-09	1	Mgst1
-0.357200418	2.60E-05	1.95E-09	1	Sgpl1
-0.323656086	2.65E-05	1.98E-09	1	Prdx1
-0.344388558	2.68E-05	2.00E-09	1	Npepps
0.301170073	2.69E-05	2.01E-09	1	H2-K1
-0.353722363	2.86E-05	2.14E-09	1	Ghitm
0.549243807	2.87E-05	2.15E-09	1	Nfkbiz
-0.434145748	2.87E-05	2.15E-09	1	Atg3
-0.489950334	2.93E-05	2.19E-09	1	Flii
0.262647648	3.05E-05	2.29E-09	1	C1qc
-0.520914975	3.13E-05	2.34E-09	1	Lsm4
-0.705158464	3.15E-05	2.36E-09	1	Stam2
0.361789152	3.18E-05	2.38E-09	1	Csf1r
0.461350976	3.33E-05	2.49E-09	1	Skil

-0.514113898	3.44E-05	2.58E-09	1	Pgk1
-0.398492005	3.54E-05	2.65E-09	1	Atp5d
-0.434931086	3.56E-05	2.67E-09	1	Dph3
-0.280017475	3.84E-05	2.88E-09	1	Bag1
-0.395073839	4.20E-05	3.15E-09	1	Snx1
-0.333214914	4.49E-05	3.36E-09	1	Myeov2
-0.358423914	4.64E-05	3.47E-09	1	Fam195b
-0.376663952	4.74E-05	3.55E-09	1	Plaur
-0.309633204	4.74E-05	3.55E-09	1	Ifi30
-0.665198115	4.84E-05	3.63E-09	1	Pgd
-0.324506745	4.87E-05	3.65E-09	1	Ndufb11
-0.59306432	4.89E-05	3.66E-09	1	Lsm3
-0.376854798	4.94E-05	3.70E-09	1	Igf1
-0.401352127	5.11E-05	3.82E-09	1	Fkbp1a
-0.410765917	5.18E-05	3.88E-09	1	Ctnnb1
-0.644851977	5.22E-05	3.91E-09	1	Ndrg1
-0.437485449	5.58E-05	4.18E-09	1	Tmem147
-0.568838747	5.59E-05	4.19E-09	1	Uba1
-0.423605146	5.62E-05	4.21E-09	1	Rftn1
-0.434243176	5.65E-05	4.23E-09	1	AI413582
0.468204292	5.75E-05	4.31E-09	1	Jun
-0.405466001	5.84E-05	4.38E-09	1	Cd93
-0.855283591	5.87E-05	4.40E-09	1	Gcsh
-0.80903494	5.89E-05	4.41E-09	1	Unc50
-0.316107823	6.10E-05	4.57E-09	1	Ankrd13a
-0.299899707	6.29E-05	4.71E-09	1	Cops6
-0.349160632	6.47E-05	4.85E-09	1	Ndufa11
-0.340591097	6.63E-05	4.97E-09	1	Rps27l
-0.304862312	6.68E-05	5.00E-09	1	Atp5f1
-0.585700273	6.74E-05	5.05E-09	1	Hsd17b10
-0.53005339	6.75E-05	5.05E-09	1	Ralgds
-0.394987493	6.89E-05	5.16E-09	1	Bcap31
-0.291039471	7.41E-05	5.55E-09	1	Uqcrq
-0.559417333	7.45E-05	5.58E-09	1	BC004004
-0.825449905	7.53E-05	5.64E-09	1	Htatip2
0.256466972	7.88E-05	5.90E-09	1	C1qa
-0.417881621	7.90E-05	5.92E-09	1	Por
-0.640745859	8.17E-05	6.12E-09	1	Mtf2
-0.345090329	8.56E-05	6.41E-09	1	Ndufv2
-0.503466807	8.65E-05	6.48E-09	1	Snrpe
-0.306104631	9.81E-05	7.35E-09	1	BC031181
-0.5022359	9.85E-05	7.38E-09	1	Wsb2
-0.38812104	9.93E-05	7.44E-09	1	Mien1
-0.426173031	0.000100325	7.51E-09	1	Tuba4a
-0.378059536	0.000100568	7.53E-09	1	Sod2
-0.523003249	0.000100766	7.55E-09	1	S100a11
-0.666766116	0.000103462	7.75E-09	1	Gyg
-0.511441583	0.000105728	7.92E-09	1	Hbp1
-0.408617377	0.000105753	7.92E-09	1	Tecr
-0.36753239	0.000107549	8.05E-09	1	Plin2
-0.495401153	0.000107912	8.08E-09	1	Crem
-0.478918931	0.000110454	8.27E-09	1	Pdcd6
-0.568414919	0.000119192	8.93E-09	1	Sdf2
-0.290129494	0.000121256	9.08E-09	1	Arl8b
-0.697782707	0.000122509	9.18E-09	1	Thoc7
-0.27532548	0.00012545	9.40E-09	1	Tpm1
-0.304217752	0.000127355	9.54E-09	1	Vcp
-0.664003439	0.00013024	9.75E-09	1	Commd3
-0.906497401	0.000134208	1.01E-08	1	Med8
-0.375818948	0.000142667	1.07E-08	1	Psmd1
-0.444223828	0.000144559	1.08E-08	1	Elovl1

-0.965304555	0.000147247	1.10E-08	1	Alg3
-0.387035696	0.000147977	1.11E-08	1	Ssr2
-0.27395227	0.000159521	1.19E-08	1	Ssb
-0.67070423	0.000161263	1.21E-08	1	D17Wsu104e
-0.389197705	0.000171737	1.29E-08	1	Rexo2
-0.401603389	0.000174266	1.31E-08	1	Bud31
-0.464215187	0.00017749	1.33E-08	1	Vdac3
-0.659511589	0.000184129	1.38E-08	1	Il1rn
-0.424033162	0.000185716	1.39E-08	1	Pik3r5
-0.259778788	0.000190068	1.42E-08	1	Gabarap
-0.626790863	0.000192122	1.44E-08	1	Dda1
-0.271238199	0.000193412	1.45E-08	1	Atp6v0b
0.591077609	0.000193839	1.45E-08	1	Ppp1r15a
-0.404455755	0.000195903	1.47E-08	1	Ndufa5
-0.353241732	0.000201139	1.51E-08	1	Cript
-0.414411867	0.000211798	1.59E-08	1	Mlf2
0.413411903	0.000214723	1.61E-08	1	Ctsc
-0.431868458	0.000217908	1.63E-08	1	Tmed3
-0.384485547	0.000218568	1.64E-08	1	Lgals3bp
-1.334701029	0.000226913	1.70E-08	1	F7
-0.593751009	0.000230264	1.72E-08	1	Gfpt1
-0.314380046	0.000236934	1.77E-08	1	Cyb5
-0.272500451	0.000239795	1.80E-08	1	Dad1
-0.271369009	0.000247796	1.86E-08	1	Eif2s2
-0.493983508	0.000261331	1.96E-08	1	Uchl5
0.503563885	0.00026544	1.99E-08	1	Irf2bp2
-0.529321814	0.00027191	2.04E-08	1	Nhp2
-0.403273139	0.000273933	2.05E-08	1	Cd36
-0.566294488	0.000275809	2.07E-08	1	St6galnac4
-0.544332973	0.00028763	2.15E-08	1	Sar1b
-1.558696064	0.000288294	2.16E-08	1	F10
-0.497121277	0.00029007	2.17E-08	1	Atg12
-0.467558327	0.000298554	2.24E-08	1	Sptlc2
-0.257503887	0.000301943	2.26E-08	1	Psmc2
-0.37000158	0.0003146	2.36E-08	1	Snx12
-0.621723713	0.000315263	2.36E-08	1	Ift20
0.701921093	0.000321692	2.41E-08	1	Axl
-0.562124284	0.000322703	2.42E-08	1	Kctd10
-0.337525087	0.00033587	2.52E-08	1	Etf1
-0.416387554	0.000341198	2.56E-08	1	Tmem9b
0.644702311	0.000354694	2.66E-08	1	Ccrl2
-0.524658102	0.00036436	2.73E-08	1	Clcn7
-0.580202403	0.000373431	2.80E-08	1	Pak1ip1
-0.806490979	0.000377067	2.82E-08	1	Ercc6
-0.386792782	0.000379798	2.84E-08	1	Chmp4b
-0.267442906	0.000391477	2.93E-08	1	Tm2d2
-0.392974487	0.000391779	2.93E-08	1	Commd4
-0.264781331	0.000404808	3.03E-08	1	Copz1
0.525284175	0.000404897	3.03E-08	1	Mafb
-0.385227754	0.000406098	3.04E-08	1	Tes
-0.82773079	0.000410222	3.07E-08	1	Aldh3b1
-0.329788584	0.000421357	3.16E-08	1	Vps28
-0.379050819	0.000422189	3.16E-08	1	Rnf7
-0.276460143	0.000429209	3.21E-08	1	Mrps21
-0.452941489	0.000450732	3.38E-08	1	Dyrk1a
-0.291828493	0.000456133	3.42E-08	1	Fbxo33
-0.314153849	0.000462586	3.46E-08	1	Oxct1
-0.513526631	0.000471299	3.53E-08	1	Echs1
-0.569405031	0.000479595	3.59E-08	1	Mrpl41
-0.795030132	0.000483473	3.62E-08	1	Anpep
-0.451911478	0.000489299	3.66E-08	1	Uap1l1

-0.282667741	0.000509499	3.82E-08	1	Smdt1
-0.29191405	0.000509907	3.82E-08	1	Atp6v1a
0.986007039	0.000518405	3.88E-08	1	Serpinf1
-0.520010241	0.000523562	3.92E-08	1	Tbrg1
-0.289251983	0.000525314	3.93E-08	1	Mrpl33
-0.398424546	0.000526012	3.94E-08	1	Stom
-0.387832764	0.000535795	4.01E-08	1	Tcp1
-0.593473997	0.000537381	4.02E-08	1	Nubp2
-0.433808033	0.000537918	4.03E-08	1	Kcnn4
-0.307979438	0.000559083	4.19E-08	1	Eif3k
-0.355479382	0.000578527	4.33E-08	1	Hvcn1
-0.336194628	0.000605417	4.53E-08	1	Ppp2ca
-0.327662759	0.000609768	4.57E-08	1	Blvra
-0.733385644	0.00061791	4.63E-08	1	Mrpl28
-0.282679644	0.000623483	4.67E-08	1	Ubxn1
0.519594665	0.000633776	4.75E-08	1	Emr1
0.253313779	0.000645654	4.84E-08	1	Jund
-0.60240127	0.000646566	4.84E-08	1	Rps6kb1
-0.392328952	0.000651133	4.88E-08	1	Ndufb10
-0.665284977	0.000664313	4.98E-08	1	Prpf38a
-0.663028053	0.000675294	5.06E-08	1	Inf2
0.582038532	0.000686608	5.14E-08	1	C5ar1
0.951201655	0.000691832	5.18E-08	1	Phlda1
-0.30426969	0.000710618	5.32E-08	1	Eprs
-0.394568106	0.00072987	5.47E-08	1	Cct3
-0.996813163	0.000753035	5.64E-08	1	Angptl2
-0.322861297	0.000769081	5.76E-08	1	Sdhd
-0.582681419	0.000800194	5.99E-08	1	Scoc
-0.473882961	0.000813294	6.09E-08	1	Uggt1
-0.445816751	0.000834974	6.25E-08	1	Dctn3
-0.264701427	0.000841297	6.30E-08	1	Pmp22
-0.423385663	0.000845805	6.33E-08	1	Gng5
-0.55581791	0.000852758	6.39E-08	1	St3gal1
-0.668270463	0.000935442	7.01E-08	1	Vps37b
-0.495217044	0.000951714	7.13E-08	1	Ankrd40
-0.361396382	0.001005554	7.53E-08	1	Leprotl1
-0.343236784	0.001019573	7.64E-08	1	Ube2a
-0.514350002	0.00102114	7.65E-08	1	Napsa
-0.321445015	0.00103147	7.73E-08	1	Srf
-0.331952864	0.00103505	7.75E-08	1	C1qbp
-0.631685348	0.001058414	7.93E-08	1	Ict1
-0.376556958	0.001106833	8.29E-08	1	Romo1
0.411456517	0.001217157	9.12E-08	1	AI607873
-0.862585934	0.001221178	9.15E-08	1	Pts
-0.422733426	0.00122723	9.19E-08	1	Alg5
-0.457975777	0.00125709	9.41E-08	1	Gda
-0.325179791	0.001271411	9.52E-08	1	Prr13
-0.511193063	0.001297587	9.72E-08	1	Hist1h2bc
-0.683834585	0.001356911	1.02E-07	1	Uso1
-1.08165635	0.001361917	1.02E-07	1	Gdpd1
-0.309852183	0.001369128	1.03E-07	1	Ndufs7
-0.285960626	0.001460909	1.09E-07	1	Fis1
-0.285016885	0.001477446	1.11E-07	1	Emc4
-0.257992635	0.001545661	1.16E-07	1	Txn2
-0.301705233	0.001586788	1.19E-07	1	Itgb2
-0.656403222	0.001724858	1.29E-07	1	Ptgr1
-0.267906158	0.00174634	1.31E-07	1	Cd68
-0.559929393	0.001754544	1.31E-07	1	Zmat5
-0.702891354	0.001803462	1.35E-07	1	Pqlc3
-1.686108766	0.001821332	1.36E-07	1	Ccr7
-0.738561185	0.00186417	1.40E-07	1	Stk16

-0.318553133	0.001873322	1.40E-07	1	Dynlrb1
-0.455710474	0.00195099	1.46E-07	1	Slc31a2
-0.296670791	0.001981305	1.48E-07	1	Psma2
0.275278228	0.001987403	1.49E-07	1	Nfkbia
-0.401977004	0.002005412	1.50E-07	1	Gba
-0.589411806	0.002030649	1.52E-07	1	Mphosph10
-0.283229363	0.002054892	1.54E-07	1	Vps35
-0.402016541	0.00206135	1.54E-07	1	Smu1
-0.265466907	0.002099348	1.57E-07	1	Cox8a
-0.517244714	0.002188533	1.64E-07	1	Cd151
-0.569685031	0.002190397	1.64E-07	1	Hcst
-0.874966148	0.00227128	1.70E-07	1	Slc7a11
-0.45818551	0.002306537	1.73E-07	1	Sdhc
-0.745055899	0.002345381	1.76E-07	1	Smim12
-0.538560884	0.002430987	1.82E-07	1	Srp68
-0.394305556	0.002437085	1.83E-07	1	Lypla1
-0.422888689	0.002527082	1.89E-07	1	Pdlim7
-0.486771836	0.002538851	1.90E-07	1	Pdgfa
-0.400268417	0.002571444	1.93E-07	1	Snx17
-0.3133936	0.002585393	1.94E-07	1	Cnih1
-0.310356565	0.002585528	1.94E-07	1	Npc1
-0.294444208	0.00269192	2.02E-07	1	Rrp1
-0.527874668	0.002791531	2.09E-07	1	Ammecr1l
-0.343288162	0.002933203	2.20E-07	1	Myo1e
-0.475045255	0.003039829	2.28E-07	1	Atp2b4
-0.369056033	0.003150532	2.36E-07	1	Reep3
-0.331831539	0.00320952	2.40E-07	1	Edf1
-0.331327832	0.003283997	2.46E-07	1	Ier3ip1
-0.266782268	0.003467237	2.60E-07	1	Atp6v1b2
0.415133728	0.003467618	2.60E-07	1	Tgif1
-0.374706051	0.003606799	2.70E-07	1	Dctn6
1.049075323	0.003623768	2.71E-07	1	Hlx
-0.260980598	0.00368159	2.76E-07	1	Mpp1
-0.499460509	0.003823219	2.86E-07	1	Ostm1
-0.690458813	0.003836023	2.87E-07	1	Clns1a
-0.552787341	0.003901584	2.92E-07	1	Arpc5l
-0.285512034	0.003931615	2.94E-07	1	Tor1aip2
-0.470885906	0.004001523	3.00E-07	1	Ufm1
-0.582204497	0.004179011	3.13E-07	1	Fgr
-0.315446416	0.00421882	3.16E-07	1	Limd1
-0.314681049	0.004249261	3.18E-07	1	Cuedc2
0.580639668	0.004260828	3.19E-07	1	Tnf
0.691448076	0.004303015	3.22E-07	1	H2-DMb1
0.653867729	0.004324169	3.24E-07	1	Mgl2
-0.287157591	0.004338679	3.25E-07	1	Impdh1
-0.257089874	0.004391062	3.29E-07	1	Nme2
0.772097205	0.004407522	3.30E-07	1	Btg2
-0.674181918	0.004413644	3.31E-07	1	Nek6
0.490346591	0.004436234	3.32E-07	1	Zfp36l1
1.063102497	0.004449241	3.33E-07	1	Plxdc2
-0.80032974	0.004462464	3.34E-07	1	Rai14
-0.453924595	0.004479508	3.35E-07	1	Akip1
-0.453721119	0.004539422	3.40E-07	1	Nadk
0.511756077	0.004543683	3.40E-07	1	Aif1
-0.29056475	0.004676233	3.50E-07	1	Eif1a
-0.423825308	0.004677133	3.50E-07	1	Pqlc1
-0.489875534	0.004946378	3.70E-07	1	Slc43a2
-0.266892259	0.004993634	3.74E-07	1	Emc7
-0.561371174	0.005109512	3.83E-07	1	Mrpl14
-0.259112466	0.005182	3.88E-07	1	Emp3
-0.780600533	0.005201857	3.90E-07	1	Gramd3

-0.363100085	0.005230792	3.92E-07	1	Sec61a1
-0.667132078	0.005349627	4.01E-07	1	Gla
-0.341776632	0.005361516	4.02E-07	1	U2af1l4
-0.279769992	0.005410708	4.05E-07	1	Dpp7
-0.341776784	0.005480047	4.10E-07	1	Rps19bp1
-0.310236743	0.005494889	4.12E-07	1	Psmd14
-0.391318743	0.005580287	4.18E-07	1	Tcn2
-0.314138756	0.005944027	4.45E-07	1	Nsf
-1.239767025	0.005977343	4.48E-07	1	Pcp4l1
-0.304605164	0.005988417	4.49E-07	1	Copa
-0.386424315	0.006123233	4.59E-07	1	Atraid
-0.683847693	0.006285314	4.71E-07	1	1500011K16Rik
-0.407942484	0.006348966	4.76E-07	1	Apoa1bp
-0.310277291	0.006503389	4.87E-07	1	Slc25a24
0.796719929	0.00667304	5.00E-07	1	Lacc1
-0.27616992	0.006801235	5.09E-07	1	Psma4
-0.323717884	0.007041094	5.27E-07	1	2310036O22Rik
-0.475829612	0.007055971	5.28E-07	1	Prosc
-0.307455075	0.00713139	5.34E-07	1	Fam96a
-0.323823671	0.007374026	5.52E-07	1	Hipk1
-0.368441652	0.007708627	5.77E-07	1	Hras
0.518026856	0.007781208	5.83E-07	1	Ier5
-0.853096318	0.007855303	5.88E-07	1	Yars
-0.435236685	0.008075699	6.05E-07	1	Cenpb
-0.460080277	0.008101965	6.07E-07	1	Bola3
-0.608778224	0.008424215	6.31E-07	1	Idh1
-0.282009741	0.008440358	6.32E-07	1	Eif1b
-0.407537373	0.008465024	6.34E-07	1	Trappc4
-0.505861503	0.008470756	6.34E-07	1	Tmem43
-0.270486855	0.008625127	6.46E-07	1	Tomm22
-0.572141264	0.009054281	6.78E-07	1	Tfrc
-0.588188734	0.00909126	6.81E-07	1	Fam63a
-0.496610583	0.009293853	6.96E-07	1	Iscu
-0.612648249	0.009392771	7.03E-07	1	Bcap29
-0.645031375	0.009848476	7.38E-07	1	Slc30a1
-0.257116816	0.009995577	7.49E-07	1	Scpep1
-0.459187462	0.01001523	7.50E-07	1	Hk3
-0.629372692	0.010018334	7.50E-07	1	Cyb561d2
-0.745163752	0.010620922	7.95E-07	1	Galc
-0.426896306	0.010752478	8.05E-07	1	AI462493
-0.598403137	0.010774579	8.07E-07	1	Mrpl55
-0.472418032	0.010889202	8.16E-07	1	Pitpnc1
-0.305771499	0.011348789	8.50E-07	1	Itgb1bp1
0.708970379	0.01162764	8.71E-07	1	Il10ra
-0.271164879	0.011754714	8.80E-07	1	Psma7
-0.366094475	0.012016267	9.00E-07	1	Ppt2
-0.396920245	0.012274932	9.19E-07	1	Ten1
-0.332218576	0.012402688	9.29E-07	1	Sptlc1
1.004125091	0.012423436	9.30E-07	1	Tagap
-0.395007413	0.012762666	9.56E-07	1	Slc38a1
0.722690892	0.012883697	9.65E-07	1	Zfhx3
-1.686952924	0.013063378	9.78E-07	1	Nkg7
0.493259528	0.013136057	9.84E-07	1	Slamf9
-0.39846111	0.013235976	9.91E-07	1	Cox19
-0.271662232	0.013362016	1.00E-06	1	Hn1
-0.398739483	0.013869118	1.04E-06	1	Phf5a
0.690796813	0.014019059	1.05E-06	1	Cx3cr1
-0.48789537	0.014204641	1.06E-06	1	Mocs2
0.53598265	0.014272419	1.07E-06	1	Kctd12
-0.526528989	0.014311436	1.07E-06	1	Cyb5r4
-0.365865669	0.014463333	1.08E-06	1	Rnf166

-0.272724247	0.015098931	1.13E-06	1	Atpif1
-0.548937989	0.015219249	1.14E-06	1	Mrpl38
-0.265735082	0.015264122	1.14E-06	1	Clec4e
-0.255987489	0.015770853	1.18E-06	1	S100a13
-0.250639759	0.016119151	1.21E-06	1	Hcfc1r1
0.925809756	0.016864226	1.26E-06	1	Pmaip1
-0.374875285	0.017174684	1.29E-06	1	Psmc6
-0.690458813	0.017257213	1.29E-06	1	Ercc1
-0.496939967	0.017316049	1.30E-06	1	Rangap1
-0.289457452	0.017536702	1.31E-06	1	Usp5
-0.412981549	0.017621282	1.32E-06	1	Scp2
-0.368716935	0.01791848	1.34E-06	1	Nudcd3
-0.455841139	0.018265782	1.37E-06	1	Gngt2
-0.897714649	0.018290649	1.37E-06	1	Ttyh2
-0.411148606	0.018383449	1.38E-06	1	Tmem60
-0.273088362	0.019206525	1.44E-06	1	Rab22a
-0.384245684	0.019379495	1.45E-06	1	Mmp14
-0.679311474	0.019827014	1.48E-06	1	Apeh
-0.256027694	0.019853428	1.49E-06	1	Zranb2
-0.333993869	0.02030168	1.52E-06	1	Stx8
-0.370475956	0.020644757	1.55E-06	1	Arhgap1
-0.278449263	0.02126066	1.59E-06	1	Ngdn
-0.40862961	0.022426189	1.68E-06	1	Emb
-0.439926148	0.023088555	1.73E-06	1	Rnf5
-0.647327718	0.023483186	1.76E-06	1	Trp53inp2
-0.413738244	0.023497424	1.76E-06	1	Rit1
-0.571613299	0.023699049	1.77E-06	1	1600002K03Rik
-0.468779069	0.023699552	1.77E-06	1	Polr2i
-0.424533632	0.024467693	1.83E-06	1	Eif3b
-0.337875524	0.024595297	1.84E-06	1	Puf60
-0.274785084	0.024759324	1.85E-06	1	Ufc1
-0.627963567	0.02510729	1.88E-06	1	L1cam
-0.435305778	0.025250866	1.89E-06	1	Acp5
-0.438407281	0.025825701	1.93E-06	1	Prdx4
-0.510061158	0.025937324	1.94E-06	1	Timm50
-0.310997316	0.026089523	1.95E-06	1	Asap1
-0.284701506	0.028928262	2.17E-06	1	Tbcb
-0.388392444	0.029152469	2.18E-06	1	Mrpl53
-0.31342911	0.029482889	2.21E-06	1	Mrpl17
-0.757098274	0.029527651	2.21E-06	1	Plrg1
-0.367216633	0.030377845	2.28E-06	1	Sulf2
-0.358756499	0.03044541	2.28E-06	1	Tmem189
-0.53842656	0.030664466	2.30E-06	1	Mrps17
-0.607340736	0.032635895	2.44E-06	1	Slc27a1
0.758844687	0.032792366	2.46E-06	1	Cited2
-0.315892683	0.032871292	2.46E-06	1	Slc25a5
-0.502391308	0.033035802	2.47E-06	1	Gnptab
-0.265477915	0.033118534	2.48E-06	1	Timm22
1.047245637	0.033732619	2.53E-06	1	Ptger4
-0.335150447	0.034141659	2.56E-06	1	Rwdd1
-0.371022424	0.034497815	2.58E-06	1	Clk4
0.268694976	0.034581233	2.59E-06	1	Mir6236
-0.283154389	0.035459742	2.66E-06	1	Stx12
-0.519616738	0.036659478	2.75E-06	1	Gmps
-0.25930469	0.03720326	2.79E-06	1	Slmo2
-0.637485314	0.037228824	2.79E-06	1	Gpd2
0.745348945	0.037235556	2.79E-06	1	Rnd3
-0.421500393	0.037575273	2.81E-06	1	Bhlhe41
-0.371403812	0.03802396	2.85E-06	1	Emc6
0.562512009	0.038240154	2.86E-06	1	Arrb2
-0.250602243	0.038773036	2.90E-06	1	Lrp1

-0.425739528	0.038817517	2.91E-06	1	Bcas2
-0.339627313	0.03945484	2.95E-06	1	Tceb1
-0.503770729	0.039461909	2.96E-06	1	Dhrs7b
-0.509178282	0.039517007	2.96E-06	1	Rad23a
-0.475734845	0.039858512	2.99E-06	1	Cul3
-0.572610814	0.040759168	3.05E-06	1	Sumf1
-0.499567888	0.040858394	3.06E-06	1	Msra
-0.337431686	0.040954935	3.07E-06	1	Arl11
-1.136834332	0.041290955	3.09E-06	1	Il1r2
-0.290697649	0.04129883	3.09E-06	1	Dnajb11
-0.332297721	0.041551272	3.11E-06	1	Poldip3
-0.411359613	0.042923788	3.21E-06	1	Ltbp3
-0.3326367	0.043120336	3.23E-06	1	Blvrb
-0.288638894	0.044401844	3.33E-06	1	Keap1
-0.434233153	0.045116594	3.38E-06	1	Lxn
0.568692385	0.045290882	3.39E-06	1	2900060B14Rik
-0.629690543	0.04572971	3.42E-06	1	Lrpprc
-0.323151488	0.045957219	3.44E-06	1	Uhrf1bp1l
-0.417242483	0.04617068	3.46E-06	1	Mfsd6
0.354457215	0.046492352	3.48E-06	1	Ccnl1
-0.267081459	0.046598568	3.49E-06	1	Esyt1
-0.278373133	0.047364388	3.55E-06	1	Scd1
-0.333809871	0.047639271	3.57E-06	1	Fn1
-0.895511492	0.04790799	3.59E-06	1	Tgfbrap1
-0.687396711	0.048213049	3.61E-06	1	Enoph1
-0.488908011	0.049990145	3.74E-06	1	Dirc2
-0.279316037	0.05207797	3.90E-06	1	Rala
-0.50915794	0.053937581	4.04E-06	1	Dpm1
0.309220213	0.054719544	4.10E-06	1	Lifr
-0.512969339	0.055631362	4.17E-06	1	Srp19
-0.543508149	0.055973876	4.19E-06	1	Anapc15
-0.26625416	0.058351075	4.37E-06	1	Emc8
-0.326155695	0.058604975	4.39E-06	1	Cisd2
-0.425342668	0.059277977	4.44E-06	1	Gstp1
-0.264681649	0.059375352	4.45E-06	1	Lrrc59
-0.354068728	0.059520528	4.46E-06	1	Naglu
-0.286050069	0.059989829	4.49E-06	1	Frrs1
-0.571456905	0.060018124	4.50E-06	1	Pcbd2
-0.352628459	0.060039218	4.50E-06	1	Ccng1
-0.279444281	0.060442509	4.53E-06	1	Cers2
-0.697686859	0.061264084	4.59E-06	1	Mphosph8
-0.352342109	0.063354773	4.74E-06	1	Selm
-0.308982626	0.064944957	4.86E-06	1	Tusc2
-0.331599111	0.065209522	4.88E-06	1	Taf9
-0.505116928	0.065924944	4.94E-06	1	Ccser2
-0.453218581	0.067330964	5.04E-06	1	Itgb7
-0.403769184	0.068032475	5.10E-06	1	Phpt1
-0.609126315	0.069911959	5.24E-06	1	Hsd3b7
-0.741443902	0.07058675	5.29E-06	1	Arl2
-0.433593906	0.072206712	5.41E-06	1	Vac14
-0.454650551	0.073806858	5.53E-06	1	Atg5
0.365633532	0.074035368	5.54E-06	1	Cd83
-0.522704373	0.077666757	5.82E-06	1	Nat6
-0.421507637	0.078315781	5.87E-06	1	Aco2
-0.555588798	0.079363012	5.94E-06	1	Mrpl34
-0.570950767	0.080705583	6.04E-06	1	Mfge8
-0.261292709	0.081848583	6.13E-06	1	Wfdc17
-0.267139966	0.082513937	6.18E-06	1	Ppm1g
-0.338317592	0.083778881	6.27E-06	1	Mkl1
-0.499978108	0.085482436	6.40E-06	1	Mrps34
-0.258236515	0.086218424	6.46E-06	1	Gatad2a

-0.424431807	0.086984423	6.51E-06	1	Dpm2
-0.375344558	0.090156044	6.75E-06	1	Ndfip1
-0.285981573	0.090204941	6.76E-06	1	Coa5
-0.62832316	0.090533969	6.78E-06	1	Plxna1
-0.431221725	0.090739276	6.80E-06	1	Sep.11
-0.252612197	0.091144371	6.83E-06	1	Napa
-0.500243891	0.092180575	6.90E-06	1	Trnau1ap
-0.470275339	0.093988828	7.04E-06	1	Dpy30
-0.446975956	0.094612448	7.09E-06	1	Lsm1
-0.567679824	0.09612949	7.20E-06	1	Snhg3
-0.252732152	0.098194557	7.35E-06	1	Mrpl24
-0.547808568	0.099767118	7.47E-06	1	Thoc1
-0.836387215	0.100716617	7.54E-06	1	Ece2
-0.325803676	0.101244425	7.58E-06	1	Cd84
-0.339286787	0.102712402	7.69E-06	1	Tmem134
-0.576999161	0.103380886	7.74E-06	1	Chst11
-0.394712409	0.104302275	7.81E-06	1	Mrps6
-0.543906157	0.104487647	7.83E-06	1	Wdr74
-0.285745463	0.104701557	7.84E-06	1	Dhx15
-0.309062908	0.106562742	7.98E-06	1	Pdlim1
-0.364836285	0.106901264	8.01E-06	1	1700123O20Rik
-0.275158559	0.108108168	8.10E-06	1	Tpp1
-0.441804871	0.109950026	8.23E-06	1	Tm2d3
-0.448609852	0.110077132	8.24E-06	1	Ubap1
0.281192824	0.113128586	8.47E-06	1	Ifi27l2a
-0.296954548	0.117937182	8.83E-06	1	Syt11
-0.624476703	0.117998338	8.84E-06	1	Ykt6
-0.437455869	0.119423457	8.94E-06	1	Fopnl
-0.552525666	0.120054562	8.99E-06	1	Srxn1
-0.456847683	0.121048047	9.07E-06	1	Cipc
-0.521791486	0.123784991	9.27E-06	1	Alas1
-0.576201134	0.123854948	9.28E-06	1	Tars2
-0.58920944	0.126844395	9.50E-06	1	Polr3k
-0.480378123	0.127494409	9.55E-06	1	Tex264
-0.417177781	0.129094038	9.67E-06	1	Acbd3
-0.403967375	0.132187798	9.90E-06	1	Mtmr10
-0.379365868	0.133731555	1.00E-05	1	Jtb
-0.34620183	0.134732739	1.01E-05	1	Mea1
-0.37400919	0.135656906	1.02E-05	1	Psen1
-0.252267648	0.145400784	1.09E-05	1	Map7d1
-0.373282788	0.149289724	1.12E-05	1	Atmin
-0.409777378	0.150236047	1.13E-05	1	Bre
-0.668082753	0.150323193	1.13E-05	1	Rnf14
-0.895080682	0.152540996	1.14E-05	1	Sdhaf2
-0.43924346	0.153539453	1.15E-05	1	Triap1
-0.571169306	0.154694105	1.16E-05	1	Trim35
-0.33817508	0.157381764	1.18E-05	1	Tmem205
-0.250342681	0.159113833	1.19E-05	1	Tmed10
-0.370684301	0.16018153	1.20E-05	1	Ndufb3
-0.309413475	0.161796491	1.21E-05	1	Babam1
-0.337968322	0.162283575	1.22E-05	1	Lsm14a
-0.267114517	0.162623471	1.22E-05	1	Slc39a7
-0.311339136	0.163122214	1.22E-05	1	Commd1
-0.398055045	0.165427201	1.24E-05	1	Fam104a
-0.440778726	0.169797217	1.27E-05	1	Cox16
-0.251535406	0.17075407	1.28E-05	1	Suclg1
0.28805468	0.170942107	1.28E-05	1	Rpl18a
-0.354526464	0.171068692	1.28E-05	1	Mmp12
-0.365172347	0.1723632	1.29E-05	1	Vti1b
-0.364123683	0.172877604	1.29E-05	1	Mrpl35
-0.383028942	0.177892541	1.33E-05	1	Mapkap1

-0.254456526	0.179427092	1.34E-05	1	Cetn3
-0.271044685	0.184784343	1.38E-05	1	Psme2b
-0.430011985	0.187440284	1.40E-05	1	Actr10
-0.780707359	0.188252338	1.41E-05	1	Dmxl2
0.715329768	0.189805151	1.42E-05	1	Ier2
0.323240449	0.194321645	1.46E-05	1	Sat1
0.455568039	0.200391317	1.50E-05	1	Hpgd
-0.259243266	0.201045431	1.51E-05	1	Chchd2
-0.461491219	0.202731074	1.52E-05	1	Agap1
0.546470809	0.20419692	1.53E-05	1	Icosl
0.384099415	0.205097055	1.54E-05	1	Isg15
-0.384477879	0.207796533	1.56E-05	1	Glrx2
-0.515112467	0.219828364	1.65E-05	1	Ndrg3
-0.325492631	0.22040876	1.65E-05	1	Ccdc174
-0.371008018	0.220928848	1.65E-05	1	Csrp1
-0.361781275	0.223390342	1.67E-05	1	St13
-0.43052796	0.2268539	1.70E-05	1	Otud4
-0.425342324	0.229481228	1.72E-05	1	Fmn1
-0.412795532	0.238083725	1.78E-05	1	Tmem120a
-0.295598431	0.239513005	1.79E-05	1	Ostc
-0.396794453	0.241204048	1.81E-05	1	Mrps23
-0.280412378	0.241455106	1.81E-05	1	Nifk
-0.562113924	0.250369548	1.88E-05	1	Nkiras2
-0.55740744	0.256863243	1.92E-05	1	Dnajc4
-0.262824207	0.257924301	1.93E-05	1	Rhoc
-0.499912853	0.263515369	1.97E-05	1	Adss
-0.384332091	0.265464902	1.99E-05	1	Uqcrb
-0.461713006	0.266871637	2.00E-05	1	Galnt7
-0.410379368	0.27990082	2.10E-05	1	Clcc1
-0.410729306	0.28168683	2.11E-05	1	Cd276
0.834692099	0.284297865	2.13E-05	1	Bbc3
-0.414133305	0.285439959	2.14E-05	1	Nop14
-0.377392637	0.289723061	2.17E-05	1	Rsbn1
-0.36182777	0.297207813	2.23E-05	1	Metap1d
-0.251409394	0.298982992	2.24E-05	1	Nol7
-0.411740445	0.301204641	2.26E-05	1	Eif2b4
-0.297983505	0.302523512	2.27E-05	1	Polr2g
-0.318594317	0.303055926	2.27E-05	1	Vps11
-0.410279579	0.306489071	2.30E-05	1	2010012O05Rik
0.39895828	0.311334875	2.33E-05	1	Pld4
-0.300558081	0.312381466	2.34E-05	1	Fyn
-0.396910695	0.32628789	2.44E-05	1	Timm17b
-0.274699898	0.328588466	2.46E-05	1	Ccdc124
-0.301108051	0.333031427	2.49E-05	1	Prelid1
-0.600777547	0.337245781	2.53E-05	1	Tmem11
-0.451852652	0.347556743	2.60E-05	1	Adam9
-0.38658917	0.375465046	2.81E-05	1	Pbdc1
-0.546731287	0.381304001	2.86E-05	1	Dhx8
0.285445779	0.389696555	2.92E-05	1	Ubc
-0.290827596	0.389913916	2.92E-05	1	Snrnp25
-0.306882955	0.402876168	3.02E-05	1	Wapal
-0.456796466	0.4031795	3.02E-05	1	Plcl2
-0.478766527	0.405109105	3.03E-05	1	Ethe1
-0.328054627	0.405539381	3.04E-05	1	Msmo1
-0.72137031	0.409535061	3.07E-05	1	Flrt3
-0.51131227	0.420296851	3.15E-05	1	Farsb
-0.739849101	0.424939725	3.18E-05	1	Ddhd1
0.35243085	0.429971087	3.22E-05	1	4632428N05Rik
-0.38761747	0.433487288	3.25E-05	1	Hmgn5
-0.29882529	0.436006678	3.27E-05	1	Trappc5
-0.460031846	0.438181913	3.28E-05	1	Mkl2

-0.398265382	0.448056972	3.36E-05	1	Slc35b1
0.939522587	0.449578891	3.37E-05	1	Tmem119
-0.572788404	0.451511898	3.38E-05	1	Kcnab2
-0.325485066	0.454151396	3.40E-05	1	Gnptg
0.881285325	0.456440741	3.42E-05	1	Col14a1
-0.312085886	0.461975931	3.46E-05	1	Arid2
-0.286331043	0.466110952	3.49E-05	1	Smad4
-0.284244706	0.466763185	3.50E-05	1	Brcc3
-0.49741197	0.467858243	3.50E-05	1	Ilf3
-0.270482802	0.482013725	3.61E-05	1	Rac2
-0.28286927	0.49097736	3.68E-05	1	Eif4ebp1
-0.429167853	0.495293332	3.71E-05	1	Heatr3
0.576347973	0.501086945	3.75E-05	1	Plbd1
-0.346371191	0.509666505	3.82E-05	1	Rad23b
-0.250672594	0.512648293	3.84E-05	1	Mrps14
0.712025362	0.516865601	3.87E-05	1	Cyp27a1
-0.484779822	0.520020332	3.89E-05	1	Tubb4b
-0.51311182	0.573574005	4.30E-05	1	Slc16a3
-0.306313107	0.580789633	4.35E-05	1	Ppp1r14b
0.367670603	0.596099432	4.46E-05	1	Cd72
-0.356638976	0.60170785	4.51E-05	1	Tssc1
-0.340711637	0.609413606	4.56E-05	1	Zc3h15
-0.449804499	0.61042651	4.57E-05	1	Gipc1
-0.500404994	0.628784594	4.71E-05	1	Dcun1d5
-0.514450416	0.641324935	4.80E-05	1	Ndufaf3
-0.285484298	0.643032721	4.82E-05	1	Ddx1
-0.469162403	0.644307882	4.83E-05	1	Mrps25
-0.269489337	0.654499326	4.90E-05	1	Rc3h1
-0.253847832	0.666626335	4.99E-05	1	Acot9
-0.954588218	0.677741483	5.08E-05	1	Plac8
-0.251344036	0.712234728	5.33E-05	1	Ebna1bp2
-0.281906687	0.714195901	5.35E-05	1	Nagpa
-0.646843647	0.721307675	5.40E-05	1	Slc39a11
-0.470178112	0.723908845	5.42E-05	1	Agfg1
-0.291541789	0.733568299	5.49E-05	1	Cpd
-0.563342418	0.74575257	5.59E-05	1	1110008P14Rik
-0.391939364	0.745916564	5.59E-05	1	Mkln1
-0.42361732	0.750484971	5.62E-05	1	Xpo1
-0.499703885	0.759408561	5.69E-05	1	Tmem223
-0.611847035	0.767599332	5.75E-05	1	Hnrnpll
-0.408401852	0.769472142	5.76E-05	1	Ergic1
-0.275204706	0.80111525	6.00E-05	1	Mitf
-0.360615263	0.805691548	6.03E-05	1	Jak2
-0.403180436	0.821874175	6.16E-05	1	Cd9
-0.382978015	0.84083571	6.30E-05	1	Slc31a1
0.31637298	0.844580849	6.33E-05	1	Fyb
-0.34286552	0.847702762	6.35E-05	1	Preb
-0.316422687	0.855193807	6.40E-05	1	Vapb
-0.470832173	0.855682876	6.41E-05	1	Clec4n
-0.403651901	0.888695315	6.66E-05	1	Zfand2b
-0.285663871	0.88912617	6.66E-05	1	Fam3c
-0.281470598	0.93538022	7.01E-05	1	Mmd
0.461342126	0.938730331	7.03E-05	1	Fosb
-0.298869818	0.941543923	7.05E-05	1	0610012G03Rik
-0.362119388	0.944108717	7.07E-05	1	Tbc1d22a
-0.499312321	0.95816869	7.18E-05	1	Epb4.1
-0.724736977	0.97626592	7.31E-05	1	Slc25a19
-0.476543702	0.987682061	7.40E-05	1	Sh3bp5
-0.610722394	1	7.56E-05	1	Adssl1
-0.274294608	1	7.58E-05	1	Arhgap10
0.470584713	1	7.82E-05	1	Hmga2-ps1

0.306961866	1	7.84E-05	1	Scamp4
-0.36587349	1	7.87E-05	1	Mfsd5
-0.457618834	1	7.87E-05	1	Tmx2
0.403581532	1	7.97E-05	1	Sdc4
-0.728644213	1	8.14E-05	1	Srprb
-0.36180316	1	8.38E-05	1	G6pdx
-0.355015362	1	8.40E-05	1	2310009A05Rik
0.313457233	1	8.69E-05	1	Cd14
-0.602739221	1	9.01E-05	1	Tspan3
-0.507475652	1	9.32E-05	1	Ttc13
-0.289887013	1	9.45E-05	1	1110038B12Rik
0.630952639	1	9.64E-05	1	Fgd2
-0.277011783	1	9.80E-05	1	Vegfb
-0.33643726	1	9.93E-05	1	Vegfa
-0.446025187	1	0.0001026	1	Ptplb
-0.300879135	1	0.0001029	1	Htra2
-0.708632764	1	0.0001039	1	Dohh
-0.564853069	1	0.0001056	1	Cops7a
0.290024614	1	0.0001073	1	2810004N23Rik
-0.515548228	1	0.0001102	1	Gpr108
-0.268145243	1	0.0001121	1	Lars
-0.37962613	1	0.0001129	1	Leprot
-0.539095914	1	0.0001146	1	Slc35e4
-0.364774343	1	0.0001149	1	Yme1l1
-0.288554699	1	0.0001155	1	Snx6
-0.455232831	1	0.0001175	1	Nelfe
-0.685507705	1	0.0001181	1	Srm
-0.25574553	1	0.0001196	1	Ttc1
-0.29853038	1	0.0001213	1	Mbd1
-0.512972195	1	0.0001216	1	Adi1
-0.314058795	1	0.0001239	1	Psmd11
-0.268348666	1	0.000125	1	Atp6v1h
-0.34995171	1	0.0001255	1	Cnot1
-0.373427866	1	0.0001258	1	Elof1
-0.394632183	1	0.0001263	1	Mrpl3
-0.280940036	1	0.0001279	1	Gtpbp4
-0.785584295	1	0.0001285	1	Lmf2
-0.348917193	1	0.0001292	1	Pmpca
-0.271790364	1	0.0001295	1	Pnpla2
-0.306709756	1	0.000133	1	Rusc2
-0.288402587	1	0.0001371	1	Ap3m1
-0.354274824	1	0.0001377	1	Trim41
-0.651578095	1	0.000138	1	Vhl
-0.368837545	1	0.0001381	1	Trappc6a
-0.288940808	1	0.0001413	1	Tmem87b
-0.321727844	1	0.0001416	1	Gnai3
-0.33072646	1	0.0001424	1	Prpf6
-0.268982762	1	0.0001425	1	E430025E21Rik
-0.566928509	1	0.000143	1	Mcm6
-0.272695225	1	0.000144	1	Pi4k2a
-0.379600569	1	0.0001448	1	Plekhm1
-0.447527652	1	0.0001504	1	Ranbp10
-0.305657098	1	0.0001508	1	Emc2
-0.31222355	1	0.0001514	1	Dnajc7
-0.313582119	1	0.0001515	1	Snx4
-0.312768907	1	0.0001518	1	Ndufs1
-0.261292212	1	0.0001519	1	Capn2
-0.458878429	1	0.0001551	1	Trmt1
-0.3110333	1	0.000159	1	Sfxn1
-0.482841653	1	0.0001613	1	Eepd1
-0.265477915	1	0.0001634	1	Pkp4

-0.452690288	1	0.000164	1	Mmp19
-0.284242985	1	0.0001648	1	Rpp21
0.317271613	1	0.0001675	1	Mir703
-0.297698749	1	0.0001686	1	Fblim1
-0.380280049	1	0.0001736	1	Nxt1
-0.331952241	1	0.0001737	1	Tmem86a
-0.257867111	1	0.0001767	1	Rnpepl1
-0.340464587	1	0.0001776	1	Pitpnb
-0.301467565	1	0.000179	1	Lrp12
-0.388794639	1	0.0001808	1	Parp4
0.516895448	1	0.0001811	1	Ccnd1
0.560503425	1	0.0001823	1	Dpysl2
-0.252584281	1	0.0001831	1	Csnk1g2
-0.55817756	1	0.000191	1	Egln1
-0.328551222	1	0.0001911	1	Dgkz
-0.404718607	1	0.0001945	1	Nsmce1
-0.39444488	1	0.0001977	1	Eif2ak1
-0.305971437	1	0.0002001	1	Fbxo22
-0.587942617	1	0.0002006	1	Jmjd6
-0.272675185	1	0.0002034	1	Cmc1
0.327289625	1	0.0002071	1	Maf
-0.372083076	1	0.0002116	1	Ube2w
-0.406744389	1	0.0002142	1	Pdrg1
-0.459255073	1	0.0002223	1	Cops8
-0.621117998	1	0.0002225	1	Nqo2
-0.320842008	1	0.0002254	1	Fibp
-0.440865569	1	0.0002289	1	Gch1
-0.924057882	1	0.0002297	1	Cdadc1
-0.427487125	1	0.00024	1	Esyt2
-0.698542422	1	0.000242	1	Cd209a
-0.56359797	1	0.000243	1	9130401M01Rik
-0.335211755	1	0.0002445	1	Rab12
0.418466743	1	0.0002513	1	Cwc27
-0.484720764	1	0.0002519	1	Cenpa
-0.264506375	1	0.0002547	1	Mcm3
0.417134596	1	0.0002591	1	Idnk
-0.487130608	1	0.00026	1	Lyst
-0.655567699	1	0.0002716	1	Isy1
0.314802572	1	0.0002757	1	Marcks
-0.485028693	1	0.0002761	1	Lin7c
-0.313389347	1	0.0002769	1	Tmem126a
-0.283242836	1	0.0002803	1	Cmtr1
-0.295912928	1	0.0002808	1	Napg
-0.492470776	1	0.0002811	1	Iah1
-0.56834435	1	0.0002855	1	Dpcd
0.797965538	1	0.0002946	1	St3gal6
-0.253872483	1	0.0002956	1	Tns3
-0.296557685	1	0.0002968	1	Gtf3c6
-0.440719655	1	0.0002974	1	Pdcd4
-0.348174281	1	0.0003002	1	P2rx4
-0.725809545	1	0.0003075	1	Hgsnat
-0.498034171	1	0.0003104	1	Kif1c
-0.560808419	1	0.0003129	1	Ddt
-0.305580774	1	0.0003322	1	Mkks
-0.328464022	1	0.0003326	1	Trappc13
-0.450991096	1	0.0003436	1	Ginm1
-0.410612792	1	0.0003456	1	Slc38a7
-0.254540778	1	0.0003474	1	Map2k4
-0.444283744	1	0.0003561	1	Amdhd2
0.26110191	1	0.0003669	1	Siglece
-0.257558453	1	0.0003718	1	Riok1

0.28476551	1	0.0003727	1	Synj2bp
0.394856494	1	0.0003907	1	Tlr2
-0.348065066	1	0.000405	1	H2afv
0.591903516	1	0.0004062	1	Ppp1r9b
0.724860881	1	0.00041	1	Mndal
-0.276511115	1	0.0004116	1	Ubxn6
-0.321664546	1	0.0004162	1	Ubap2
-0.386002245	1	0.000417	1	Tspan5
-0.383732454	1	0.000429	1	Sdccag8
0.399678618	1	0.0004359	1	Klf2
-0.396024862	1	0.0004365	1	Syvn1
-0.310857975	1	0.0004366	1	Srpr
-0.460534348	1	0.0004378	1	AW112010
-0.319329326	1	0.0004379	1	Agpat4
-0.267684408	1	0.0004405	1	Gpatch8
-0.453350384	1	0.0004439	1	Mbp
-0.42516465	1	0.0004478	1	Abcc1
-0.482530486	1	0.0004497	1	Brap
0.647866933	1	0.0004525	1	Fam46a
-0.345869595	1	0.0004545	1	Trak2
-0.479272818	1	0.0004579	1	Yipf5
-0.600358276	1	0.000458	1	Nr1h3
-0.497834004	1	0.0004766	1	Kif3a
-0.278534529	1	0.0004781	1	Smc3
-0.597628485	1	0.0004806	1	Prnp
0.737374605	1	0.000508	1	Dst
-0.277997677	1	0.0005346	1	Fkbp15
-0.325094496	1	0.0005427	1	Zpr1
-0.325772166	1	0.000544	1	Polr1c
-0.353925183	1	0.0005471	1	Ftsj3
-0.791456134	1	0.0005503	1	Trem1
-0.375791502	1	0.000551	1	Mgat4b
-0.494667864	1	0.0005524	1	Dctpp1
-0.383907133	1	0.0005566	1	Traf7
-0.334012402	1	0.0005566	1	Sucla2
-0.491235445	1	0.0005622	1	Cdipt
-0.415464918	1	0.0005629	1	Vps36
-0.340044807	1	0.0005726	1	Zmpste24
-0.499027419	1	0.0005733	1	Mrps12
-0.325578664	1	0.0005762	1	Snapin
-0.371609677	1	0.0005807	1	Rtca
-0.325548949	1	0.0005864	1	Rsl1d1
0.601376953	1	0.0006051	1	H2-M3
0.262463379	1	0.0006141	1	Gp49a
-0.296149742	1	0.000616	1	Gnl1
-0.268091894	1	0.0006166	1	Akr1b10
-0.333532777	1	0.0006223	1	Znrf1
-0.289659526	1	0.0006426	1	2610001J05Rik
-0.538275275	1	0.0006519	1	Mrps26
-0.375689588	1	0.0006573	1	Cyhr1
1.126459549	1	0.0006622	1	Ifit2
-0.417771838	1	0.0006646	1	Mad2l2
0.317997272	1	0.0006895	1	Cdkn1a
-0.392788602	1	0.0007048	1	Bap1
-0.331170504	1	0.0007071	1	Uvrag
0.616530965	1	0.0007374	1	Ccl12
-0.334995719	1	0.0007543	1	Exosc8
-0.262393873	1	0.0007676	1	Poldip2
-0.266104452	1	0.0007817	1	Mrpl40
-0.289998338	1	0.0007925	1	Uba5
-0.320399027	1	0.000795	1	Ldlrap1

-0.27467739	1	0.0007986	1	Dnajc14
0.400490818	1	0.0008192	1	Rgs1
0.256714738	1	0.0008289	1	Lrrfip2
-0.476667029	1	0.0008315	1	Farsa
-0.290524914	1	0.0008389	1	Clec7a
0.37094714	1	0.0008392	1	Wdfy4
-0.338200413	1	0.0008682	1	Sik3
-0.429615289	1	0.0008712	1	Ccndbp1
-0.350316688	1	0.000882	1	Tmem70
-0.305062325	1	0.0008898	1	Mrpl37
-0.311967619	1	0.0009021	1	Mpnd
-0.292497651	1	0.0009204	1	Prorsd1
-0.343532012	1	0.0009272	1	Setd5
-0.302658836	1	0.0009323	1	Nup62
-0.317118772	1	0.0009326	1	Mybbp1a
-0.265591829	1	0.0009418	1	Erlin1
-0.294900938	1	0.0009465	1	Akirin1
-0.334469957	1	0.0009554	1	Plgrkt
-0.423669794	1	0.0009586	1	Heatr5a
-0.849005679	1	0.0009652	1	Avpi1
-0.347464388	1	0.0009715	1	Mcee
-0.470190557	1	0.001	1	Gmnn
-0.52130648	1	0.0010005	1	Sh2b2
-0.386567896	1	0.0010129	1	B3gnt2
-0.347900358	1	0.0010138	1	Tor1a
-0.264182997	1	0.00102	1	Cnpy3
-1.307184912	1	0.0010262	1	Ly6c2
-0.540675953	1	0.0010342	1	Arl6ip5
-0.265958939	1	0.0010836	1	Gcc2
-0.260819429	1	0.0010936	1	Mrpl32
-0.345235881	1	0.0010984	1	Eif1ad
-0.271029944	1	0.0011104	1	Myl6
0.399777872	1	0.0011331	1	Usp4
-0.726211915	1	0.0011596	1	Ap4s1
-0.30839824	1	0.0011697	1	Prkch
-0.58700235	1	0.0011761	1	Mki67
-0.315293282	1	0.0012129	1	Lap3
-0.263690016	1	0.0012416	1	Cct7
-0.295566967	1	0.0012429	1	Zfp598
-0.442377159	1	0.0012509	1	Cd274
0.423523272	1	0.0012708	1	Tnfaip3
0.985092039	1	0.001276	1	Ppfia4
0.66255961	1	0.0013019	1	Oasl2
-0.251374477	1	0.0013116	1	Utp3
-0.552796835	1	0.0013208	1	Pptc7
-0.424745704	1	0.0013267	1	2310035C23Rik
-0.376654754	1	0.0013406	1	Gpalpp1
-0.355227271	1	0.0013443	1	4921524J17Rik
-0.438855465	1	0.0013489	1	Psmf1
-0.315739467	1	0.0013621	1	Hint2
-0.401236579	1	0.0013774	1	Rab28
0.684216181	1	0.0013885	1	Abca9
0.294304639	1	0.0014249	1	Nbr1
-0.271168819	1	0.0014494	1	Map2k2
-0.381788179	1	0.0014522	1	Tpgs1
-0.25976184	1	0.0014543	1	Pmpcb
0.344333699	1	0.0014921	1	Ccl2
-0.451055368	1	0.0015093	1	Fgd4
-0.677054868	1	0.0015174	1	Pop7
-0.368206976	1	0.0015255	1	Gsr
0.666780706	1	0.0015455	1	Rqcd1

-0.973081128	1	0.0015464	1	Tbc1d4
-0.378773015	1	0.0015486	1	Fam213b
-0.302677473	1	0.0015515	1	Xpnpep1
-0.479727838	1	0.0016076	1	Cdc16
-0.319631781	1	0.0016205	1	Irf3
-0.600354648	1	0.00163	1	Ing4
0.774052736	1	0.0016385	1	Nlrp3
-0.473101615	1	0.0016691	1	H2-Oa
-0.342483188	1	0.0016767	1	Cgrrf1
-0.523187045	1	0.0016907	1	Cd24a
-0.76503968	1	0.0017765	1	Ube3b
-0.394909521	1	0.0018304	1	Ate1
0.298643555	1	0.0018417	1	Rad50
0.281894949	1	0.0018753	1	Cyth2
-0.468020117	1	0.0018963	1	Fech
-0.296307039	1	0.0019107	1	Snrnp200
-0.28036179	1	0.0019127	1	Fem1c
0.556184564	1	0.0019869	1	Pmepa1
0.659084327	1	0.0020482	1	Id3
-0.305298756	1	0.0020621	1	Tmem248
-0.43430562	1	0.0020695	1	Gclm
-0.268954761	1	0.0020925	1	Cdc123
-0.38654686	1	0.0020999	1	Prkar2a
-0.556712717	1	0.0022249	1	Ap5s1
-0.758661568	1	0.0022451	1	Agtrap
-0.420739215	1	0.0022797	1	Gtpbp1
-0.347601094	1	0.0023067	1	Uckl1
0.262629939	1	0.00232	1	Wnk1
-0.452883042	1	0.0023593	1	Caml
-0.297805234	1	0.0023963	1	Prrc2b
-0.34512931	1	0.0024199	1	Rhoh
-0.291369579	1	0.0024542	1	Tmem57
-0.492417578	1	0.002567	1	Pank2
-0.391851043	1	0.0025908	1	Prpf31
-0.275027335	1	0.0025939	1	Sfmbt1
-0.603022391	1	0.0026394	1	Ifitm1
0.331561256	1	0.0026458	1	Rgs2
0.31248601	1	0.0027195	1	Tpd52
-0.3840293	1	0.0028297	1	Arl5c
0.806647175	1	0.002883	1	Rtp4
-0.251142335	1	0.0029116	1	Aqr
0.631809716	1	0.0029188	1	Fgl2
-0.328132154	1	0.0030286	1	Mrpl11
-0.393640499	1	0.0030525	1	Flot1
-0.291746085	1	0.0030786	1	Vta1
0.782213336	1	0.0031341	1	Trerf1
-0.354912704	1	0.0031722	1	Yeats4
-0.386442404	1	0.003184	1	Lta4h
0.270217962	1	0.0031908	1	Gm1821
0.799676244	1	0.0032037	1	Arhgap4
-0.415727893	1	0.0032342	1	Sep.06
0.597095429	1	0.0033375	1	Oasl1
0.278846331	1	0.0034004	1	Stt3b
-0.300359147	1	0.0034038	1	Dlst
-0.277265321	1	0.003473	1	Osbpl9
-0.46010658	1	0.0035608	1	Pigyl
0.29304488	1	0.0035841	1	Atp2b1
-0.336852353	1	0.0036623	1	Anapc4
0.296236016	1	0.0037098	1	Ly86
0.972071611	1	0.0037367	1	Clec4b1
-0.401507813	1	0.0037767	1	Faf1

-0.361970905	1	0.003791	1	Smarcb1
-0.288958089	1	0.0038228	1	Rsf1
-0.252095448	1	0.0038354	1	Milr1
-0.452969907	1	0.0038426	1	Hspbp1
0.584599001	1	0.0038648	1	Rcsd1
-0.265654762	1	0.003903	1	Ppp6r3
-0.575519134	1	0.0039616	1	Gpr126
-0.26758796	1	0.0039802	1	Mettl6
-0.496506939	1	0.0039839	1	Hmg20b
-0.702326803	1	0.0039882	1	Top2a
-0.327159641	1	0.004109	1	Ppp2r5e
-0.463763624	1	0.0041514	1	Ccnk
-0.25302683	1	0.0043628	1	Lrrc47
-0.348234497	1	0.0043677	1	Twistnb
0.525238953	1	0.0043769	1	Camk1d
-0.370732122	1	0.0043936	1	Imp3
0.631068292	1	0.0044294	1	Setd1b
-0.496933966	1	0.0044956	1	AB041803
-0.369686586	1	0.0045073	1	Zfp120
0.522018027	1	0.0045315	1	Lcp2
-0.314511372	1	0.0046197	1	Otub1
-0.260945646	1	0.0047012	1	Nbeal1
-0.571157873	1	0.0047264	1	Bloc1s6
-0.350492403	1	0.004825	1	Emd
0.323147854	1	0.004826	1	Fam49b
-0.454099829	1	0.004881	1	Cul4b
-0.302323303	1	0.0050643	1	AA467197
-0.477470603	1	0.0051463	1	Abhd17c
-0.524995709	1	0.0051864	1	Foxo1
-0.410276764	1	0.005221	1	Fam114a2
-0.309227811	1	0.0052984	1	Lrrc8a
-0.2950848	1	0.0053207	1	Thoc6
-0.418163588	1	0.0053361	1	Mefv
-0.387655874	1	0.0053511	1	Tpcn1
-0.281960864	1	0.0054864	1	Glg1
-0.315242499	1	0.0055377	1	Eaf1
-0.280554656	1	0.005834	1	Eapp
-0.314764509	1	0.0058579	1	Phf20
-0.36487831	1	0.0059713	1	2310022A10Rik
-0.271035123	1	0.005991	1	Plekha1
-0.338406459	1	0.0060307	1	N6amt2
-1.008917119	1	0.0062463	1	Il6st
-0.368548572	1	0.0065534	1	Hs6st1
0.476744047	1	0.0067008	1	Gm6377
0.554513354	1	0.0067351	1	Rassf2
-0.39955986	1	0.0068007	1	Eif2b5
-0.381342842	1	0.0069699	1	Cdk18
0.311769547	1	0.0071715	1	Cyth4
-1.167361382	1	0.0073803	1	Gpx3
-0.253765345	1	0.0075755	1	Ptplad1
0.373459208	1	0.0076245	1	Rcbtb2
0.27151578	1	0.0083196	1	Srek1ip1
-0.298214596	1	0.0084079	1	Ctnnbl1
-0.582445914	1	0.0084543	1	Arl6ip4
0.596381009	1	0.0084829	1	Uba7
0.273439183	1	0.0084888	1	D17Wsu92e
-0.418398121	1	0.0088045	1	Cnot7
0.570120192	1	0.0089072	1	Trim30d
0.262541333	1	0.0090256	1	Actr3
0.286598928	1	0.00915	1	Ptprj
0.425027189	1	0.0093625	1	Ehd4

-0.261662977	1	0.0093661	1	Tollip
-0.316810247	1	0.009426	1	Ide
0.372487567	1	0.0095933	1	Tspan14
0.493362022	1	0.0096673	1	Parp8
-0.278899895	1	0.0097261	1	Bop1
-0.4509074	1	0.0097445	1	Zbtb17
-0.36361116	1	0.0099371	1	Nolc1
2.035701005	2.87E-78	2.15E-82	2	F13a1
1.918975143	4.61E-74	3.46E-78	2	Cbr2
2.349116348	7.31E-70	5.48E-74	2	Lyve1
1.570407869	9.99E-69	7.48E-73	2	Pf4
1.768802224	5.48E-63	4.11E-67	2	Folr2
1.379209861	9.07E-63	6.79E-67	2	Mrc1
1.052778531	4.69E-61	3.51E-65	2	Sepp1
1.955935035	2.66E-60	1.99E-64	2	C4b
1.713172369	2.63E-55	1.97E-59	2	Gas6
2.068689077	2.52E-54	1.89E-58	2	Cd163
1.512491369	8.26E-53	6.19E-57	2	Maf
1.524941076	4.39E-52	3.29E-56	2	Fcgrt
1.009540859	3.20E-49	2.39E-53	2	Trf
1.493678906	4.25E-46	3.18E-50	2	Cfh
1.909469089	1.12E-45	8.38E-50	2	Ltc4s
1.145736468	2.17E-43	1.62E-47	2	Stab1
0.919213183	2.76E-43	2.07E-47	2	Dab2
1.540449541	4.78E-43	3.58E-47	2	Igfbp4
2.049872414	7.66E-42	5.74E-46	2	Ccl24
-1.682056958	9.30E-40	6.96E-44	2	Gngt2
1.065508168	3.20E-39	2.40E-43	2	Ifi27l2a
-0.968669698	4.61E-39	3.45E-43	2	Lgals3
1.507200671	1.05E-37	7.89E-42	2	Lifr
-1.600782739	1.55E-37	1.16E-41	2	Cd9
1.836800485	3.48E-37	2.61E-41	2	Timd4
1.843923961	6.96E-37	5.21E-41	2	Ccl8
0.670845095	1.30E-36	9.73E-41	2	Serinc3
2.237443258	2.33E-35	1.75E-39	2	Ednrb
1.877583111	2.14E-34	1.60E-38	2	Selenbp1
1.838176249	5.94E-34	4.45E-38	2	Fxyd2
2.154929359	7.09E-34	5.31E-38	2	Fcna
-1.424676267	7.19E-34	5.38E-38	2	Spp1
1.897314937	1.26E-33	9.45E-38	2	Chp2
1.664303747	4.62E-33	3.46E-37	2	Stard8
-1.738237217	8.18E-33	6.13E-37	2	Mmp12
1.833845363	1.13E-32	8.46E-37	2	Fgfr1
0.748981629	1.77E-31	1.32E-35	2	Ly6e
-0.975175897	4.84E-31	3.62E-35	2	Mpeg1
1.30342653	1.05E-30	7.86E-35	2	Txnip
1.588247167	1.43E-30	1.07E-34	2	Sult1a1
1.681955642	1.66E-30	1.24E-34	2	Clec10a
1.639519596	3.85E-29	2.89E-33	2	C6
1.264846073	9.40E-29	7.04E-33	2	Rnasel
-0.983983749	9.73E-29	7.29E-33	2	Cd44
-0.948465765	1.69E-28	1.27E-32	2	Basp1
1.712448599	7.77E-28	5.82E-32	2	Plekhg5
-1.414792253	1.47E-27	1.10E-31	2	Bhlhe40
1.145382466	1.62E-27	1.21E-31	2	Mgl2
2.013302997	1.99E-27	1.49E-31	2	Rcn3
0.801554813	2.43E-27	1.82E-31	2	Ccl6
1.65259218	2.60E-27	1.95E-31	2	Lyl1
0.930081482	6.36E-27	4.76E-31	2	Jun
-1.758216779	1.58E-26	1.18E-30	2	Il7r
-1.805101569	2.38E-26	1.79E-30	2	Adam8

1.812535939	3.53E-26	2.64E-30	2	Colec12
-1.277902736	4.18E-26	3.13E-30	2	Abcg1
0.57655155	4.85E-26	3.63E-30	2	C1qa
1.299972014	1.27E-25	9.48E-30	2	Itsn1
-1.157359988	1.34E-25	1.01E-29	2	Lpl
1.518653571	2.78E-25	2.08E-29	2	Tmod1
1.151916832	3.06E-25	2.29E-29	2	Slco2b1
2.229837983	3.20E-25	2.40E-29	2	Retnla
0.954413657	4.63E-25	3.47E-29	2	Abca9
**0.604254287**	**5.05E-25**	**3.78E-29**	**2**	**Csf1r**
1.4821265	5.12E-25	3.83E-29	2	Cp
-0.805008402	7.48E-25	5.61E-29	2	Cd52
-1.582908088	1.09E-24	8.18E-29	2	Itgax
0.493386735	1.98E-24	1.48E-28	2	C1qc
-0.970466246	4.15E-24	3.11E-28	2	Rgs1
1.536922117	1.79E-23	1.34E-27	2	Pla2g2d
0.66595018	1.94E-23	1.45E-27	2	Ifitm3
1.067589653	2.86E-23	2.14E-27	2	Hspa1a
-1.239168928	7.19E-23	5.38E-27	2	Cytip
-1.867987752	7.91E-23	5.93E-27	2	Gpnmb
1.258262024	8.55E-23	6.40E-27	2	Alox5
1.270250925	2.31E-22	1.73E-26	2	Gpr34
-1.489141029	2.45E-22	1.83E-26	2	Il1rn
2.195785103	3.07E-22	2.30E-26	2	Cd209f
-1.68030532	3.15E-22	2.36E-26	2	Lhfpl2
1.867269427	4.24E-22	3.17E-26	2	Vsig4
-0.891687529	4.76E-22	3.57E-26	2	Lsp1
0.977748778	5.01E-22	3.75E-26	2	Ap2a2
0.715380406	7.68E-22	5.75E-26	2	Cd81
0.404539254	1.48E-21	1.11E-25	2	C1qb
1.00462163	2.72E-21	2.03E-25	2	Eps15
0.824238547	3.53E-21	2.64E-25	2	Glul
-1.25555055	4.10E-21	3.07E-25	2	Glipr1
1.130166271	1.20E-20	9.02E-25	2	Wwp1
0.642680625	2.22E-20	1.67E-24	2	Ctsc
0.66739808	7.49E-20	5.61E-24	2	Tmem176b
-0.504493566	8.80E-20	6.59E-24	2	Cyba
-0.720937101	1.86E-19	1.39E-23	2	Sqstm1
-0.922793429	1.95E-19	1.46E-23	2	S100a11
0.975881476	2.04E-19	1.53E-23	2	Hspa1b
1.178560475	2.08E-19	1.56E-23	2	Bmp2
0.801131792	2.47E-19	1.85E-23	2	Cfp
0.723813801	3.88E-19	2.91E-23	2	Rhob
-1.553062925	3.90E-19	2.92E-23	2	Tgm2
1.053206993	4.09E-19	3.07E-23	2	Klf2
1.25337649	5.13E-19	3.84E-23	2	Hpgd
-0.606402539	6.66E-19	4.99E-23	2	Atox1
1.270338965	1.63E-18	1.22E-22	2	Ddx60
0.866071391	1.68E-18	1.26E-22	2	Rnase4
1.484004435	2.46E-18	1.84E-22	2	Cmah
1.021015483	2.50E-18	1.88E-22	2	Etv1
1.705650931	2.57E-18	1.92E-22	2	Cd209g
1.156097931	3.25E-18	2.44E-22	2	Rin2
-0.880957737	3.93E-18	2.95E-22	2	Fam129b
-0.758155857	6.84E-18	5.12E-22	2	Anxa2
-0.996623293	1.03E-17	7.72E-22	2	Slc15a3
-1.505321915	1.11E-17	8.29E-22	2	Sema4d
0.906814191	1.20E-17	8.97E-22	2	Sema4a
-0.857582103	1.38E-17	1.03E-21	2	S100a6
-1.107101749	1.92E-17	1.44E-21	2	Syngr1

-0.656569571	2.20E-17	1.65E-21	2	Pkm
-0.613048575	2.32E-17	1.74E-21	2	Cxcl16
-1.272497246	2.33E-17	1.74E-21	2	Plaur
-0.925322672	3.05E-17	2.29E-21	2	Sgk1
0.639301001	3.30E-17	2.47E-21	2	Ehd4
1.062400733	4.87E-17	3.65E-21	2	P2ry12
1.29610276	5.07E-17	3.80E-21	2	P2ry13
1.119862971	5.34E-17	4.00E-21	2	Bank1
0.946216969	1.07E-16	7.99E-21	2	Pisd-ps1
0.38660588	1.10E-16	8.22E-21	2	Lyz2
-0.961665552	1.14E-16	8.54E-21	2	S100a4
-0.858937122	1.33E-16	9.98E-21	2	Taldo1
-0.794834155	2.56E-16	1.92E-20	2	Capg
1.221155205	2.91E-16	2.18E-20	2	Reps2
1.165981419	3.75E-16	2.81E-20	2	Gbp7
1.158710915	3.93E-16	2.95E-20	2	Smagp
-1.50955971	4.39E-16	3.29E-20	2	Hilpda
-0.730978826	5.56E-16	4.17E-20	2	Gm2a
-1.061923678	9.37E-16	7.02E-20	2	Gde1
-0.946195153	1.36E-15	1.02E-19	2	Aprt
-0.806920326	1.62E-15	1.21E-19	2	Psmb6
-1.150112344	2.65E-15	1.99E-19	2	Actn1
-0.973698518	3.65E-15	2.74E-19	2	Tnfaip2
0.871742755	3.70E-15	2.77E-19	2	Gas7
-0.836174535	4.62E-15	3.46E-19	2	Rnh1
-0.880154096	6.20E-15	4.64E-19	2	Anxa1
-1.145163623	6.83E-15	5.12E-19	2	Clec4d
-1.114691298	6.86E-15	5.14E-19	2	Rilpl2
-1.299595825	7.59E-15	5.68E-19	2	Mmp14
-0.672127322	8.72E-15	6.53E-19	2	Plin2
-1.255313094	9.09E-15	6.81E-19	2	Cdk18
1.090953593	9.38E-15	7.02E-19	2	Lrp6
-1.61810375	1.30E-14	9.70E-19	2	Ndrg1
-1.678076221	1.34E-14	1.00E-18	2	Clec4e
0.99685428	2.29E-14	1.71E-18	2	Ms4a6b
-1.051943247	2.50E-14	1.87E-18	2	Slamf7
-0.826118748	2.50E-14	1.87E-18	2	Alcam
0.940391184	2.63E-14	1.97E-18	2	Tsc22d3
-1.412493586	2.98E-14	2.23E-18	2	Acp5
1.283134103	3.66E-14	2.74E-18	2	Tns1
-1.101484049	4.95E-14	3.71E-18	2	Dpep2
-1.089223705	7.44E-14	5.57E-18	2	Gpr137b-ps
0.836301722	8.26E-14	6.19E-18	2	Fcgr2b
-1.054853827	1.09E-13	8.17E-18	2	Gpr137b
0.588374872	2.04E-13	1.53E-17	2	Nrp1
1.496448059	2.15E-13	1.61E-17	2	Trpv4
0.54724463	2.77E-13	2.07E-17	2	Fcgr3
0.79377451	2.95E-13	2.21E-17	2	Fcrls
1.29004679	3.18E-13	2.38E-17	2	Sesn1
-0.958754609	3.29E-13	2.47E-17	2	Txnrd1
-0.695729123	3.41E-13	2.55E-17	2	Skil
1.295343366	4.67E-13	3.50E-17	2	Siglec1
-1.253581703	4.78E-13	3.58E-17	2	Smim3
-0.401875691	6.08E-13	4.55E-17	2	Fxyd5
-0.838856756	6.57E-13	4.92E-17	2	Mdm2
-1.134113334	7.43E-13	5.56E-17	2	Rnf128
0.482008801	7.92E-13	5.93E-17	2	Pltp
-1.40190778	9.78E-13	7.32E-17	2	Pdpn
0.281681859	9.96E-13	7.46E-17	2	Cst3
0.752515113	1.06E-12	7.95E-17	2	Snx2
-0.651637688	1.33E-12	9.93E-17	2	Cstb

0.332896045	1.37E-12	1.02E-16	2	Itm2b
-1.170309651	1.66E-12	1.24E-16	2	Ezr
-0.425914282	1.71E-12	1.28E-16	2	Vim
-0.363889244	2.02E-12	1.51E-16	2	Rpl41
0.844599008	2.26E-12	1.69E-16	2	Ap1b1
1.015912729	2.57E-12	1.92E-16	2	Pepd
0.558512205	2.59E-12	1.94E-16	2	Timp2
-1.262295163	3.70E-12	2.77E-16	2	Il1b
-0.605711902	4.23E-12	3.17E-16	2	Id2
-0.689881056	4.39E-12	3.29E-16	2	Atp6v1c1
-1.205826951	6.22E-12	4.66E-16	2	Emb
-1.446750625	6.39E-12	4.79E-16	2	Igf2r
-0.8756646	6.74E-12	5.05E-16	2	Clec7a
1.417705832	8.23E-12	6.16E-16	2	Gm4951
-0.533102868	1.20E-11	8.95E-16	2	Srgn
0.475960359	1.23E-11	9.18E-16	2	Cltc
0.597121001	1.33E-11	9.96E-16	2	Ccl9
-0.613013896	1.34E-11	1.00E-15	2	Slc3a2
-1.323963867	1.38E-11	1.03E-15	2	Galnt6
-0.810197157	1.41E-11	1.05E-15	2	Pld3
0.670149249	1.41E-11	1.06E-15	2	C5ar1
-0.895720943	1.72E-11	1.29E-15	2	Lat2
0.493948643	1.84E-11	1.38E-15	2	Marcks
-1.042712922	1.89E-11	1.42E-15	2	Tspan13
0.72820629	2.30E-11	1.72E-15	2	Tgfbr2
1.152225492	3.18E-11	2.38E-15	2	Npl
-1.375563016	3.37E-11	2.53E-15	2	Slc6a8
-0.540743732	3.40E-11	2.54E-15	2	Atp6v0e
1.03768091	3.51E-11	2.63E-15	2	Slc9a9
-0.958667731	3.65E-11	2.73E-15	2	Pfkp
-0.731299685	3.75E-11	2.81E-15	2	Lrrfip1
-1.136700937	4.62E-11	3.46E-15	2	Pqlc1
-1.275839476	5.18E-11	3.88E-15	2	Gpr132
0.870011043	5.49E-11	4.11E-15	2	Sgpp1
-0.591658668	5.66E-11	4.24E-15	2	Nceh1
-0.816818671	6.15E-11	4.61E-15	2	Stx3
-0.598735074	7.56E-11	5.66E-15	2	Vdac2
-0.981596997	7.96E-11	5.96E-15	2	Fam107b
1.016086649	9.25E-11	6.93E-15	2	Cebpd
0.858182474	9.40E-11	7.04E-15	2	Nfxl1
-0.903043328	1.04E-10	7.82E-15	2	Cd72
1.443050571	1.11E-10	8.34E-15	2	Cfb
0.963622492	1.35E-10	1.01E-14	2	Rab11fip5
-1.713912696	1.44E-10	1.08E-14	2	Acot7
-0.742296769	1.44E-10	1.08E-14	2	Csf2rb
-0.40158738	2.03E-10	1.52E-14	2	Gnas
-0.786104069	2.16E-10	1.62E-14	2	Prdx6
1.01834876	2.39E-10	1.79E-14	2	Serpinb8
-0.8667732	2.55E-10	1.91E-14	2	Zfand2a
-1.195949525	3.33E-10	2.50E-14	2	Napsa
-0.62752888	4.01E-10	3.00E-14	2	Csrnp1
-0.518447416	4.33E-10	3.24E-14	2	AF251705
-0.99447968	4.41E-10	3.30E-14	2	Anpep
-0.869192906	4.44E-10	3.33E-14	2	Vasp
-0.906269498	4.47E-10	3.35E-14	2	Abr
-0.62841234	4.68E-10	3.51E-14	2	Tgfbr1
-0.732942592	4.86E-10	3.64E-14	2	Ccrl2
-0.318395176	6.23E-10	4.67E-14	2	Ctsz
-0.983090434	6.96E-10	5.21E-14	2	Malt1
-0.845403243	7.19E-10	5.39E-14	2	Ybx3
-0.75053487	7.75E-10	5.81E-14	2	Esd

0.644193874	8.01E-10	6.00E-14	2	Ninj1
-1.007953423	8.58E-10	6.42E-14	2	Hvcn1
-0.694223847	1.11E-09	8.29E-14	2	Gdf15
0.541134443	1.19E-09	8.90E-14	2	Pmp22
-0.683807073	1.24E-09	9.27E-14	2	Nme1
-0.55419611	1.41E-09	1.05E-13	2	Litaf
-0.542785763	1.41E-09	1.06E-13	2	Gsto1
-1.445308838	1.58E-09	1.19E-13	2	Scd1
0.922578278	1.75E-09	1.31E-13	2	Ptgs1
-0.713807098	1.83E-09	1.37E-13	2	Fyn
-0.788036912	2.17E-09	1.62E-13	2	Psmc4
0.719332171	2.47E-09	1.85E-13	2	Blvrb
-0.934795249	2.53E-09	1.89E-13	2	Atp8b4
-0.825643632	2.63E-09	1.97E-13	2	2010111I01Rik
0.905391021	2.97E-09	2.22E-13	2	Gda
-0.57064016	3.43E-09	2.57E-13	2	Pde4b
-0.414113516	3.90E-09	2.92E-13	2	Atp5e
-1.159073969	3.98E-09	2.98E-13	2	Scd2
-1.269902623	4.67E-09	3.50E-13	2	Kcnn4
-0.529487725	4.78E-09	3.58E-13	2	Lmna
-0.529758561	5.22E-09	3.91E-13	2	Arl4c
0.57882	5.42E-09	4.06E-13	2	Ccl7
1.044985117	6.10E-09	4.57E-13	2	Adap2
0.633230557	6.67E-09	5.00E-13	2	Hmox1
-0.690299031	8.03E-09	6.01E-13	2	Axl
-0.869333442	9.29E-09	6.95E-13	2	Mrpl20
0.840249691	9.69E-09	7.26E-13	2	Aoah
-1.101832702	1.03E-08	7.72E-13	2	Gpr35
-1.315163456	1.08E-08	8.06E-13	2	Cyfip2
-0.558423835	1.08E-08	8.12E-13	2	Txn1
-0.795323722	1.22E-08	9.17E-13	2	Pik3r5
0.822194097	1.24E-08	9.30E-13	2	Fam46a
-0.823891076	1.34E-08	1.01E-12	2	Sod2
-0.686033145	1.66E-08	1.24E-12	2	Rnf149
-0.622050959	1.69E-08	1.26E-12	2	Atp6v1a
1.225632192	1.78E-08	1.33E-12	2	Il16
0.62546652	1.93E-08	1.44E-12	2	Zfp36l1
0.877625829	2.04E-08	1.53E-12	2	Fgd2
-1.842083111	2.14E-08	1.60E-12	2	F10
-1.459154067	2.24E-08	1.67E-12	2	Cd300lf
-0.565691764	2.28E-08	1.71E-12	2	Pim1
0.934790151	2.44E-08	1.82E-12	2	Arsb
-0.750527195	2.77E-08	2.07E-12	2	Il2rg
-0.512611982	2.83E-08	2.12E-12	2	Tmsb10
1.091676053	3.09E-08	2.32E-12	2	Oas2
1.058586709	3.15E-08	2.36E-12	2	Hfe
-0.665027885	3.19E-08	2.39E-12	2	Psmd8
-0.893763932	3.34E-08	2.50E-12	2	Timm17a
-0.581610253	3.37E-08	2.53E-12	2	Ifrd1
-0.884261057	3.52E-08	2.64E-12	2	Got1
0.903436314	3.70E-08	2.77E-12	2	Bin1
1.120768861	3.89E-08	2.91E-12	2	Dse
1.259431471	4.11E-08	3.07E-12	2	Nudt16
0.801682688	4.67E-08	3.50E-12	2	Dclre1c
0.681694502	4.95E-08	3.71E-12	2	Rab3il1
-1.313627497	5.06E-08	3.79E-12	2	Slc7a11
0.524843624	5.24E-08	3.92E-12	2	Tmem176a
-0.919705241	5.60E-08	4.20E-12	2	Fn1
-1.089534009	6.16E-08	4.62E-12	2	Ccl5
1.001199324	6.35E-08	4.75E-12	2	Rasgrp3
-0.905971093	6.60E-08	4.94E-12	2	Ptk2b

-1.074501905	7.97E-08	5.97E-12	2	Fam20c
0.741857539	8.55E-08	6.41E-12	2	Tmem8
0.81365777	9.20E-08	6.89E-12	2	Snx6
0.679967461	9.65E-08	7.23E-12	2	Aldh2
0.542618845	1.10E-07	8.26E-12	2	Mafb
-1.285426896	1.19E-07	8.90E-12	2	Agpat4
-1.58422334	1.19E-07	8.94E-12	2	Mefv
-0.443796927	1.28E-07	9.59E-12	2	Fnip2
-0.854161466	1.42E-07	1.07E-11	2	Csf2rb2
-0.312372046	1.46E-07	1.09E-11	2	Arpc2
-0.428181734	1.48E-07	1.11E-11	2	Msn
-0.981716949	1.51E-07	1.13E-11	2	Dennd4a
-0.436870763	1.64E-07	1.23E-11	2	Prdx1
1.000913831	1.72E-07	1.29E-11	2	Cd33
-0.89346911	1.86E-07	1.40E-11	2	Pold4
-0.288711485	1.90E-07	1.42E-11	2	Rpl32
-1.339261542	1.91E-07	1.43E-11	2	L1cam
0.415090589	1.94E-07	1.46E-11	2	Mir6236
-0.769885162	2.12E-07	1.59E-11	2	Myo1e
-1.200086067	2.17E-07	1.63E-11	2	Fabp5
0.903351442	2.56E-07	1.92E-11	2	Sulf2
0.993165461	2.58E-07	1.94E-11	2	Sorbs3
0.392787499	3.02E-07	2.26E-11	2	Jund
-0.596706033	3.20E-07	2.39E-11	2	Skap2
-0.438190487	3.20E-07	2.40E-11	2	Aldoa
-1.070962341	3.50E-07	2.62E-11	2	Fgr
-0.483679209	3.61E-07	2.70E-11	2	Itgb2
0.387058086	3.64E-07	2.72E-11	2	Ifitm2
0.536069299	3.75E-07	2.81E-11	2	Egr1
0.37547452	3.93E-07	2.94E-11	2	Wfdc17
-0.531864753	4.25E-07	3.18E-11	2	Plec
-1.599786366	4.67E-07	3.50E-11	2	Apbb2
-1.119127046	4.88E-07	3.65E-11	2	Slc38a1
1.099789365	4.98E-07	3.73E-11	2	Ophn1
-0.290393215	5.19E-07	3.89E-11	2	Rps14
-1.097397337	6.21E-07	4.65E-11	2	Tnip1
-0.782937333	6.49E-07	4.86E-11	2	Bcl2a1b
-1.019896247	7.34E-07	5.50E-11	2	Ell2
0.797395595	8.23E-07	6.16E-11	2	Lpin1
0.902659808	8.51E-07	6.38E-11	2	Dusp6
-0.564842921	9.33E-07	6.98E-11	2	Tgif1
0.668469044	9.48E-07	7.10E-11	2	Ccl12
-0.578681565	1.02E-06	7.65E-11	2	Cct7
0.824555379	1.07E-06	7.99E-11	2	Zcchc24
-1.172942557	1.09E-06	8.18E-11	2	Atp6v0d2
0.582944753	1.21E-06	9.09E-11	2	Ssh2
0.325934647	1.26E-06	9.41E-11	2	Grn
0.816363724	1.30E-06	9.70E-11	2	Vps13c
-0.742526408	1.39E-06	1.04E-10	2	Tnfsf9
-0.942472232	1.44E-06	1.08E-10	2	Crem
-0.727424737	1.45E-06	1.09E-10	2	Itgav
-0.289529423	1.55E-06	1.16E-10	2	Lilrb4
0.631775315	1.69E-06	1.27E-10	2	Mef2c
-0.88816495	1.70E-06	1.27E-10	2	Ltbp3
0.759190233	1.71E-06	1.28E-10	2	Adam15
1.177879286	1.73E-06	1.30E-10	2	Rgs18
-1.151572868	1.79E-06	1.34E-10	2	Cadm1
-0.616498578	2.03E-06	1.52E-10	2	Adam17
1.042564894	2.18E-06	1.63E-10	2	Hrh1
-0.685966124	2.24E-06	1.67E-10	2	Lrrc59
-1.200093083	2.90E-06	2.18E-10	2	Tnip3

-0.337813414	3.06E-06	2.29E-10	2	Iqgap1
-0.822841269	3.14E-06	2.35E-10	2	Fam96b
-0.437097485	3.45E-06	2.58E-10	2	Uqcrfs1
-0.274092733	3.68E-06	2.76E-10	2	Cox4i1
-0.563176937	3.72E-06	2.78E-10	2	Trappc2l
-0.697446643	3.85E-06	2.88E-10	2	Tes
-0.448441127	3.91E-06	2.93E-10	2	Gna13
-1.210326654	4.12E-06	3.08E-10	2	Chst11
0.821732713	4.25E-06	3.18E-10	2	Pros1
1.020172345	4.45E-06	3.34E-10	2	Psd3
-0.466598113	4.47E-06	3.34E-10	2	Rab8b
-0.609134003	4.47E-06	3.35E-10	2	Surf4
-0.523056095	4.65E-06	3.48E-10	2	Myl12a
-0.85886254	5.53E-06	4.14E-10	2	Nlrp3
0.657476959	5.83E-06	4.37E-10	2	Cmtm6
-0.411689351	5.84E-06	4.37E-10	2	Mtpn
-1.180567175	6.19E-06	4.64E-10	2	Slc2a1
-1.29090047	6.22E-06	4.66E-10	2	Vegfa
0.679963101	6.32E-06	4.74E-10	2	Mpp1
1.31749748	6.39E-06	4.79E-10	2	Grap
-0.277639824	6.41E-06	4.80E-10	2	Rps3
1.059753267	6.92E-06	5.19E-10	2	Slc15a2
-1.183980757	7.20E-06	5.39E-10	2	Mmp19
0.694269225	7.20E-06	5.39E-10	2	Clec4a1
0.474537366	7.64E-06	5.72E-10	2	Mndal
0.83819907	7.90E-06	5.92E-10	2	Ctla2b
-0.4935415	7.99E-06	5.98E-10	2	Rac2
-1.150020806	8.23E-06	6.17E-10	2	AA467197
-0.611752149	8.83E-06	6.61E-10	2	Nab1
0.410935508	9.30E-06	6.96E-10	2	Pld4
1.098026215	9.91E-06	7.42E-10	2	Dok2
-1.322198349	9.96E-06	7.46E-10	2	Ctsk
-1.442483069	1.03E-05	7.73E-10	2	Rab11fip1
-0.575196194	1.04E-05	7.81E-10	2	Clic4
0.68693692	1.06E-05	7.91E-10	2	Rgl1
-0.32508231	1.20E-05	9.02E-10	2	Anxa5
-0.26325667	1.21E-05	9.03E-10	2	Gm13826
-0.725663412	1.22E-05	9.11E-10	2	Psmd1
-0.559120759	1.25E-05	9.39E-10	2	Zcrb1
-0.412377192	1.28E-05	9.56E-10	2	Tnfaip3
-1.70012535	1.29E-05	9.64E-10	2	Npy
-1.069930882	1.40E-05	1.05E-09	2	AI504432
-0.732135438	1.43E-05	1.07E-09	2	Msrb1
-1.231489004	1.50E-05	1.13E-09	2	Arl5c
-0.622603884	1.61E-05	1.21E-09	2	Edem1
0.772302143	1.62E-05	1.22E-09	2	Usp24
-0.857780589	1.63E-05	1.22E-09	2	Il1r2
-0.960045601	1.73E-05	1.29E-09	2	Fbxo32
0.754207448	1.78E-05	1.33E-09	2	Cyr61
0.633711527	1.83E-05	1.37E-09	2	Scamp2
1.07686888	1.87E-05	1.40E-09	2	Cxcl13
-0.553751857	1.90E-05	1.42E-09	2	Furin
0.888589047	2.08E-05	1.56E-09	2	Fam213b
-0.758037823	2.14E-05	1.60E-09	2	Wsb2
-0.588242282	2.31E-05	1.73E-09	2	Ppp2ca
0.790608583	2.32E-05	1.74E-09	2	Sbf2
0.579091829	2.53E-05	1.89E-09	2	Tcn2
-1.35087427	2.58E-05	1.93E-09	2	F7
-0.469889083	2.66E-05	1.99E-09	2	Tspo
-0.775762774	2.90E-05	2.17E-09	2	Tbcb
-0.623608051	3.08E-05	2.30E-09	2	Eif3g

0.632019519	3.10E-05	2.32E-09	2	P2ry6
0.632329299	3.15E-05	2.36E-09	2	Marcksl1
-1.11621926	3.33E-05	2.50E-09	2	Pdlim7
-0.630338674	3.37E-05	2.52E-09	2	Nhp2
-0.473526088	3.58E-05	2.68E-09	2	Cd93
-0.487810231	3.72E-05	2.79E-09	2	Elp5
0.775004883	3.87E-05	2.90E-09	2	Tmem37
-0.474644485	4.05E-05	3.03E-09	2	Cox5a
-0.492858592	4.19E-05	3.14E-09	2	Psmb1
0.688503933	4.27E-05	3.20E-09	2	Usp21
0.919311278	4.54E-05	3.40E-09	2	Ctnnd1
-0.523226888	4.92E-05	3.68E-09	2	Pilra
-0.620503263	5.19E-05	3.89E-09	2	Cox17
-0.605714625	5.28E-05	3.96E-09	2	Myo1g
-0.789622425	5.59E-05	4.19E-09	2	Pdgfa
0.741845413	6.30E-05	4.72E-09	2	Myo7a
-1.070733065	6.34E-05	4.75E-09	2	Lrp12
1.072823193	6.43E-05	4.82E-09	2	B3galnt1
-1.067392429	6.58E-05	4.93E-09	2	Fam46c
-1.095286221	6.60E-05	4.94E-09	2	Stap1
-0.437370951	7.02E-05	5.26E-09	2	Esyt1
-0.471466026	7.16E-05	5.36E-09	2	Ubl3
-0.927932188	8.04E-05	6.02E-09	2	Havcr2
-0.343131661	8.25E-05	6.18E-09	2	Psap
-0.891748338	8.45E-05	6.33E-09	2	Plbd1
-0.354726564	0.000100321	7.51E-09	2	Tatdn2
-0.463112572	0.000102265	7.66E-09	2	Nus1
-0.462137711	0.000105342	7.89E-09	2	Ncf4
-0.553048088	0.000107569	8.06E-09	2	Anxa4
-1.200051147	0.00010774	8.07E-09	2	Gsr
-0.721193547	0.000116939	8.76E-09	2	Snx10
-0.716382313	0.000117592	8.81E-09	2	Gpd2
0.41421571	0.000124068	9.29E-09	2	Unc93b1
0.701877718	0.000131576	9.85E-09	2	Nacc2
-0.679849134	0.00013686	1.03E-08	2	Slc11a2
-0.527699258	0.000137634	1.03E-08	2	B4galt5
-0.819487687	0.000141473	1.06E-08	2	Gla
-0.500218297	0.000145628	1.09E-08	2	Itga5
-0.443704709	0.00015519	1.16E-08	2	Tax1bp1
0.323993285	0.000159735	1.20E-08	2	Kctd12
-0.52604233	0.000160805	1.20E-08	2	Pgd
-0.465111888	0.000165488	1.24E-08	2	Cib1
0.32827417	0.000173324	1.30E-08	2	Rgs10
-0.441959047	0.000200379	1.50E-08	2	Rap2a
0.617800513	0.000205903	1.54E-08	2	Myo5a
-0.52313835	0.000206421	1.55E-08	2	Gusb
0.693875503	0.000213899	1.60E-08	2	Klf4
-0.47364635	0.000223768	1.68E-08	2	Cpd
0.692345798	0.000236576	1.77E-08	2	2610507B11Rik
-1.159395849	0.000250262	1.87E-08	2	Cd274
0.561248459	0.000261171	1.96E-08	2	Slfn5
-0.460082023	0.000261778	1.96E-08	2	Slc25a39
-0.628954378	0.000282764	2.12E-08	2	Cux1
0.638518476	0.000287	2.15E-08	2	Cux2
-0.484368086	0.000298906	2.24E-08	2	Pomp
-0.529814052	0.000309028	2.31E-08	2	Gclm
-0.594347744	0.000333357	2.50E-08	2	Tmem43
-0.457829375	0.000340498	2.55E-08	2	Gadd45b
-0.699242421	0.000353822	2.65E-08	2	Ak2
0.885998758	0.000369448	2.77E-08	2	Dpysl3
-0.519800711	0.000372381	2.79E-08	2	Grpel1

0.782558996	0.000392509	2.94E-08	2	Gab3
-0.440182739	0.000399702	2.99E-08	2	Map2k3
-0.865337817	0.000428087	3.21E-08	2	Ppp1r2
-0.358657817	0.000432776	3.24E-08	2	Spag9
0.923950499	0.000434859	3.26E-08	2	Rasgrp4
0.510180376	0.000468451	3.51E-08	2	Gmfg
-0.703894597	0.000480467	3.60E-08	2	Vps37b
-0.292657461	0.000495675	3.71E-08	2	Cox6b1
-0.596796159	0.000503368	3.77E-08	2	Pmepa1
-0.809461842	0.000504181	3.78E-08	2	Bcl2l11
0.922477256	0.000504379	3.78E-08	2	Arhgef12
0.650632305	0.000515825	3.86E-08	2	Cyp27a1
0.903037914	0.000534256	4.00E-08	2	Idh2
-0.485554161	0.000535908	4.01E-08	2	Lasp1
-0.397498792	0.000560747	4.20E-08	2	Gsn
0.705848237	0.000621033	4.65E-08	2	Ptp4a3
-0.512796912	0.000622458	4.66E-08	2	Prkar1a
-0.615347843	0.000650637	4.87E-08	2	Rassf3
-0.538372506	0.000680307	5.10E-08	2	Nr4a2
-0.764888742	0.000720676	5.40E-08	2	Runx3
-0.313877917	0.000769606	5.76E-08	2	Tpm4
-0.368335193	0.000775514	5.81E-08	2	Sra1
-1.039250034	0.000781649	5.85E-08	2	Cipc
-0.347036002	0.000782401	5.86E-08	2	Atf4
0.829741781	0.00080657	6.04E-08	2	P2rx7
-0.773473044	0.000820692	6.15E-08	2	Pbxip1
-1.065614189	0.000891814	6.68E-08	2	Gfpt1
-0.392482101	0.000895519	6.71E-08	2	Soat1
-0.406298078	0.000916044	6.86E-08	2	Smpdl3a
-0.616685917	0.00093712	7.02E-08	2	Ufm1
-0.482006907	0.000998911	7.48E-08	2	Mbnl2
-0.866214004	0.001050413	7.87E-08	2	Zmynd15
1.065729077	0.001051334	7.87E-08	2	Fez2
0.607273868	0.00109796	8.22E-08	2	Kctd2
-0.493358611	0.001145491	8.58E-08	2	Tnfrsf1b
0.474055878	0.001215137	9.10E-08	2	Zcchc6
0.624721584	0.001222414	9.16E-08	2	Mapk14
-0.91172179	0.001263507	9.46E-08	2	Cdt1
-0.446601532	0.001277736	9.57E-08	2	Tcp1
-0.408539424	0.001287267	9.64E-08	2	1110007C09Rik
-0.531331818	0.001302884	9.76E-08	2	Syt11
-0.535738413	0.001307296	9.79E-08	2	Aurkaip1
-0.405873432	0.001321849	9.90E-08	2	Tnfrsf12a
-0.452180228	0.001389583	1.04E-07	2	Ndufa1
-0.561582869	0.001390945	1.04E-07	2	Banf1
0.537734824	0.001391833	1.04E-07	2	Tcf4
-0.427421758	0.001392086	1.04E-07	2	Cxcr4
0.742816493	0.00148246	1.11E-07	2	Frmd4b
-0.336208435	0.001483775	1.11E-07	2	Ier3ip1
-0.515326216	0.001485107	1.11E-07	2	Mrps7
-0.380234125	0.001490821	1.12E-07	2	Eif5a
-0.850499886	0.001558823	1.17E-07	2	Asph
0.779958477	0.001609516	1.21E-07	2	Slc28a2
-0.87650165	0.001622766	1.22E-07	2	Comtd1
0.842693122	0.001641091	1.23E-07	2	B4galt6
-0.687743765	0.001655437	1.24E-07	2	AB124611
-0.409340167	0.001673897	1.25E-07	2	Epb4.1l2
0.76826776	0.001697315	1.27E-07	2	Prkacb
-0.452746327	0.001778664	1.33E-07	2	Fam50a
-0.55602163	0.001814368	1.36E-07	2	Cnih4
-0.973783416	0.001904068	1.43E-07	2	Parvg

0.983982576	0.001912265	1.43E-07	2	Adrb2
-0.325798379	0.001996873	1.50E-07	2	Ptprc
-0.335860227	0.002067794	1.55E-07	2	Ube2d3
-1.021689976	0.00221182	1.66E-07	2	Angptl2
-0.403766015	0.002254891	1.69E-07	2	Actn4
-0.869164938	0.002306411	1.73E-07	2	Glrx
0.485520774	0.002324755	1.74E-07	2	Rnf130
-0.977961011	0.002377626	1.78E-07	2	Rusc2
-0.634633437	0.002397546	1.80E-07	2	Dtnbp1
-0.446476098	0.002628442	1.97E-07	2	Slc35b1
-0.68461995	0.002703893	2.03E-07	2	Rgcc
-0.58634365	0.002715986	2.03E-07	2	Rab4b
-0.704956122	0.002724263	2.04E-07	2	Tuba4a
-0.442008702	0.002748184	2.06E-07	2	Rasgef1b
0.698658982	0.002791902	2.09E-07	2	Hgsnat
-0.467071913	0.002793495	2.09E-07	2	Tmem251
-0.463083008	0.002796531	2.09E-07	2	Eepd1
-0.423381949	0.002804985	2.10E-07	2	Ckb
-0.370738615	0.002821175	2.11E-07	2	Ubxn4
-0.947730537	0.002843833	2.13E-07	2	Slc16a3
-0.561963789	0.002926633	2.19E-07	2	Lrrfip2
-0.708484745	0.002953706	2.21E-07	2	Tpm1
-0.529451319	0.002996004	2.24E-07	2	Mapkapk3
-0.724262243	0.003073042	2.30E-07	2	Wdr74
-1.416330137	0.003181776	2.38E-07	2	Chil3
-0.428082358	0.003195736	2.39E-07	2	Arpp19
0.308307522	0.003355995	2.51E-07	2	Mt1
-0.640800092	0.003413357	2.56E-07	2	Cd300a
0.421667295	0.003416995	2.56E-07	2	Rassf2
-0.257138249	0.003454347	2.59E-07	2	Gp49a
-0.403834109	0.003486243	2.61E-07	2	Dynlrb1
0.543810756	0.003518156	2.63E-07	2	Nr3c1
0.269577218	0.00367946	2.76E-07	2	Snx5
-0.495363525	0.003717724	2.78E-07	2	Fblim1
-0.523131669	0.003760565	2.82E-07	2	Psma6
-0.498258994	0.003829335	2.87E-07	2	Prkch
-0.453109362	0.004022431	3.01E-07	2	Azin1
-0.52099994	0.004055493	3.04E-07	2	Kdm6b
-0.998465482	0.004090475	3.06E-07	2	Snrnp25
-0.410322348	0.004161243	3.12E-07	2	Hexb
-0.856311108	0.004331812	3.24E-07	2	Ercc6
-0.344861626	0.004344489	3.25E-07	2	Flna
-1.188652306	0.004355668	3.26E-07	2	Flrt3
-0.291860799	0.004414427	3.31E-07	2	Spty2d1
-0.835423661	0.00451337	3.38E-07	2	Bzw2
-0.580994742	0.004521812	3.39E-07	2	Senp2
-0.34611589	0.004556717	3.41E-07	2	Nfe2l2
-1.317937254	0.004579605	3.43E-07	2	Trem1
-0.559223523	0.004666611	3.50E-07	2	Rel
-0.557989521	0.00473968	3.55E-07	2	Pdcd6
0.42642737	0.004975183	3.73E-07	2	Lyz1
-0.286389109	0.005005784	3.75E-07	2	Slc25a3
-0.366663327	0.005212729	3.90E-07	2	Trem2
-0.3576092	0.005227035	3.91E-07	2	Cct6a
-0.740914897	0.00543192	4.07E-07	2	D17Wsu104e
-0.846140034	0.005553044	4.16E-07	2	Lyst
-0.762256201	0.005573499	4.17E-07	2	Sik3
-0.525561958	0.005788983	4.34E-07	2	3110043O21Rik
0.671551166	0.00586918	4.40E-07	2	Eps8
0.487582691	0.005908472	4.43E-07	2	Cysltr1
-0.391199748	0.005950232	4.46E-07	2	Slfn2

1.103098901	0.006040749	4.52E-07	2	Ang
-0.466062684	0.006054736	4.53E-07	2	2010107E04Rik
-1.314520612	0.006439786	4.82E-07	2	P2ry10
-0.373003697	0.006459484	4.84E-07	2	Rabgef1
0.484618224	0.006608828	4.95E-07	2	Prcp
-0.953557565	0.007011324	5.25E-07	2	Spn
-0.30987744	0.007724327	5.79E-07	2	Cd14
-0.389473324	0.007860043	5.89E-07	2	5730416F02Rik
-0.568538012	0.00799521	5.99E-07	2	Pkib
0.558506902	0.008082759	6.05E-07	2	Sh3gl1
-0.472571271	0.008186385	6.13E-07	2	Atp6v1d
0.403557815	0.008194754	6.14E-07	2	St3gal6
-0.536784542	0.008270422	6.19E-07	2	Ptprs
-0.528047903	0.008364311	6.26E-07	2	Lmo4
-0.886780664	0.008684821	6.50E-07	2	Creld2
-0.535009304	0.008950697	6.70E-07	2	Pak1ip1
-0.644452905	0.009024576	6.76E-07	2	Por
0.618658691	0.009195333	6.89E-07	2	Pml
0.615141239	0.009428361	7.06E-07	2	Rcsd1
-0.489055174	0.009499635	7.11E-07	2	Map4k3
-0.804699691	0.009563329	7.16E-07	2	Atp2b4
-0.57147045	0.009838595	7.37E-07	2	Ckap4
-0.424653418	0.009845097	7.37E-07	2	Ndufs2
0.514229827	0.00985887	7.38E-07	2	Ifitm6
-0.435720358	0.009888037	7.41E-07	2	Rftn1
-0.418422899	0.010051393	7.53E-07	2	Smim14
-1.125541084	0.010458546	7.83E-07	2	Cd24a
-0.377975222	0.010698082	8.01E-07	2	Rexo2
-0.417423087	0.010916512	8.18E-07	2	Ifngr2
0.391276217	0.011015808	8.25E-07	2	Hlx
-0.451342667	0.011410205	8.55E-07	2	Irak2
-1.343918135	0.011601721	8.69E-07	2	Plac8
1.018615309	0.01168585	8.75E-07	2	Gpx3
1.084141876	0.011793562	8.83E-07	2	Pbx3
-0.527220615	0.011966159	8.96E-07	2	Hnrnpll
-0.507671431	0.012148909	9.10E-07	2	Glt25d1
-0.792119046	0.012398049	9.29E-07	2	Itga6
-1.425181874	0.013388454	1.00E-06	2	Nkg7
-0.456023374	0.013495627	1.01E-06	2	Mdh2
0.706705759	0.013517762	1.01E-06	2	Nfix
-0.57625709	0.013616779	1.02E-06	2	Tmem147
-0.497154837	0.014365533	1.08E-06	2	Galns
0.597367324	0.014614977	1.09E-06	2	Tbc1d9b
-0.407126645	0.01503836	1.13E-06	2	Emp1
0.581631322	0.01568336	1.17E-06	2	Ecm1
-0.989072838	0.016426601	1.23E-06	2	Slc39a11
-0.714538241	0.016529494	1.24E-06	2	Ptpn22
0.564318718	0.016683053	1.25E-06	2	Apobec3
-0.80221947	0.016717331	1.25E-06	2	Abhd5
-0.442072934	0.017091118	1.28E-06	2	Tank
0.652243957	0.01826154	1.37E-06	2	Gm1966
-0.687363511	0.018389693	1.38E-06	2	Odc1
-0.467027406	0.019212823	1.44E-06	2	Psmd14
0.596044743	0.019458842	1.46E-06	2	Ccr5
-0.495180503	0.019459907	1.46E-06	2	Sh3pxd2b
-0.574881092	0.019707227	1.48E-06	2	Map4k4
0.321004604	0.020098619	1.51E-06	2	Fos
0.780857372	0.021086797	1.58E-06	2	Ctsf
-0.371575215	0.02153086	1.61E-06	2	Chchd1
0.553088784	0.022356218	1.67E-06	2	Ap2m1
-0.724875729	0.022856045	1.71E-06	2	Fnbp4

-0.410184595	0.022985342	1.72E-06	2	Pin1
0.605868539	0.023713363	1.78E-06	2	Zfp90
-0.425342426	0.024008277	1.80E-06	2	Scamp3
0.377639406	0.024378641	1.83E-06	2	Aplp2
-0.398797063	0.024572785	1.84E-06	2	Aup1
-0.644188603	0.024658624	1.85E-06	2	Amz1
-0.323831013	0.025063827	1.88E-06	2	Dap
-0.832094924	0.025824663	1.93E-06	2	Wdfy1
-0.521316674	0.026134267	1.96E-06	2	Eif1ad
-0.514792745	0.026210634	1.96E-06	2	Slc37a2
-0.674290337	0.026267963	1.97E-06	2	Endod1
-0.299341885	0.026273637	1.97E-06	2	M6pr
-0.78385207	0.027260638	2.04E-06	2	Trpv2
-0.501106963	0.027825167	2.08E-06	2	Sugt1
-0.501482647	0.028942712	2.17E-06	2	Eif2s1
-0.776660037	0.029051427	2.18E-06	2	Jak2
-0.335621079	0.02909563	2.18E-06	2	Atp5b
-0.362099783	0.029656755	2.22E-06	2	Fis1
-0.267288425	0.029834096	2.23E-06	2	Atp6v0b
0.567579448	0.030280927	2.27E-06	2	Srgap2
-0.257400348	0.03062208	2.29E-06	2	Pnpla2
-0.274134191	0.031224867	2.34E-06	2	Uqcr10
-0.531663768	0.031423387	2.35E-06	2	Ece2
-0.637658528	0.03160733	2.37E-06	2	Fuca2
1.136161642	0.031882422	2.39E-06	2	Nxpe5
-0.60631089	0.032123841	2.41E-06	2	Mak16
-0.805888014	0.032486611	2.43E-06	2	Cyp4f18
-0.616059386	0.032621682	2.44E-06	2	Cirh1a
-0.566528942	0.032790331	2.46E-06	2	Eed
-0.409296297	0.033404474	2.50E-06	2	Timm13
-0.328527188	0.034603304	2.59E-06	2	Abca1
-1.071662058	0.03471634	2.60E-06	2	Itgb7
-0.799319529	0.035095478	2.63E-06	2	Ripk3
-0.810692237	0.03518674	2.64E-06	2	Adrbk2
-0.814456498	0.035284158	2.64E-06	2	Trp53inp2
-0.450864692	0.035521643	2.66E-06	2	Gng10
-0.472673776	0.035750036	2.68E-06	2	Dctn4
-0.506230339	0.03575974	2.68E-06	2	Dctn3
-0.548918868	0.037358467	2.80E-06	2	Ict1
-0.427884823	0.037479104	2.81E-06	2	Cd97
-0.489616054	0.038708914	2.90E-06	2	Pttg1
-0.743563181	0.038732568	2.90E-06	2	Thbs1
-0.281414547	0.039140528	2.93E-06	2	Ndufa8
0.424326032	0.039182606	2.93E-06	2	Inpp4a
-0.420900842	0.0401434	3.01E-06	2	Rab6a
0.483834485	0.04072202	3.05E-06	2	Arhgef3
-0.530351456	0.040815805	3.06E-06	2	Gtpbp4
-0.578535569	0.041740816	3.13E-06	2	Plcg2
-0.839859725	0.042556481	3.19E-06	2	Inf2
0.422302466	0.042563157	3.19E-06	2	Zfhx3
-0.853500748	0.04368839	3.27E-06	2	Sntb2
0.604974137	0.043773694	3.28E-06	2	Ppp1r21
-0.696344953	0.044341313	3.32E-06	2	Pqlc3
-0.405754616	0.047550562	3.56E-06	2	Cd84
-0.682364869	0.048406969	3.63E-06	2	Utp18
-0.506842443	0.049034568	3.67E-06	2	Mob4
-0.83803279	0.05009589	3.75E-06	2	Ifitm1
-0.648220997	0.050903418	3.81E-06	2	Srxn1
0.855262624	0.052223364	3.91E-06	2	Dusp7
-0.443801764	0.054965606	4.12E-06	2	Rhoc
-0.554294257	0.060755795	4.55E-06	2	Arih2

-0.449160518	0.062773166	4.70E-06	2	Gch1
-0.9195327	0.063022706	4.72E-06	2	Aim1
-0.897498126	0.065217824	4.88E-06	2	1810011H11Rik
-0.32729963	0.066244934	4.96E-06	2	Fnip1
-0.576041236	0.066698617	5.00E-06	2	Hsd3b7
-0.609872835	0.067707385	5.07E-06	2	Cd276
-1.008518306	0.067961288	5.09E-06	2	Slc2a6
0.610907543	0.069488156	5.20E-06	2	Ifi203
-0.312196495	0.069799318	5.23E-06	2	Rnasek
-0.343575917	0.070236883	5.26E-06	2	Tmem206
-0.443525633	0.072002218	5.39E-06	2	Chd7
-0.496873646	0.072618575	5.44E-06	2	Tbc1d15
0.66103787	0.07595699	5.69E-06	2	Pid1
-0.390512954	0.076624262	5.74E-06	2	Etv3
-0.341946468	0.078177487	5.86E-06	2	Nedd8
-0.634820885	0.078420095	5.87E-06	2	Smox
-0.355032733	0.079500014	5.95E-06	2	Alas1
-0.359397607	0.079996024	5.99E-06	2	Dusp11
0.727175326	0.080474074	6.03E-06	2	Pikfyve
-0.405571659	0.08085428	6.06E-06	2	Yif1b
-0.479327315	0.080866826	6.06E-06	2	Acer3
-1.340114296	0.083685539	6.27E-06	2	Nes
-0.375941662	0.087649205	6.56E-06	2	Btg1
-0.595911945	0.091450301	6.85E-06	2	Rcbtb2
-1.393467102	0.091894329	6.88E-06	2	Ccr7
-0.438015189	0.091984734	6.89E-06	2	H13
-0.271923536	0.09201017	6.89E-06	2	1700017B05Rik
-0.508478774	0.092027106	6.89E-06	2	Myof
0.358161955	0.092158714	6.90E-06	2	Aif1
-0.455688077	0.093468365	7.00E-06	2	Zak
0.422120092	0.095594509	7.16E-06	2	Btg2
-0.377243502	0.096549929	7.23E-06	2	Ist1
-0.416039534	0.098055151	7.34E-06	2	Rheb
-0.499621986	0.098059183	7.34E-06	2	Tlr2
-0.272229953	0.100582069	7.53E-06	2	Atp6v1e1
0.896772471	0.101375343	7.59E-06	2	Slc25a37
-0.914590791	0.101490312	7.60E-06	2	Pik3cb
-0.532067493	0.10173745	7.62E-06	2	Rbm7
-0.401846863	0.10401124	7.79E-06	2	Plgrkt
0.746890603	0.10618408	7.95E-06	2	Hps3
-0.478113113	0.109020172	8.17E-06	2	Tomm70a
-0.320499314	0.109672467	8.21E-06	2	Commd4
0.625882893	0.109942957	8.23E-06	2	Tubgcp5
0.633251692	0.117839455	8.83E-06	2	Kbtbd11
-0.390423523	0.119382825	8.94E-06	2	Rpsa
-0.557974666	0.119927661	8.98E-06	2	Gnl3
0.607878889	0.123718591	9.27E-06	2	Mat2a
-0.388062853	0.124848918	9.35E-06	2	Dtx4
-0.693774931	0.126314164	9.46E-06	2	Srpk1
-0.458704108	0.127267999	9.53E-06	2	Tmbim4
-0.831395632	0.12997902	9.73E-06	2	Ubr4
-0.542412958	0.130374996	9.76E-06	2	Atp7a
-0.345417231	0.131651216	9.86E-06	2	Eif3h
-0.435209546	0.134849704	1.01E-05	2	Slc27a1
-0.361591953	0.135360768	1.01E-05	2	Hint2
-0.407410824	0.136044263	1.02E-05	2	Bax
-0.907977971	0.139567517	1.05E-05	2	Mthfd2
0.343848435	0.14271452	1.07E-05	2	Epsti1
-0.496290563	0.144002848	1.08E-05	2	Icam1
-0.491889222	0.145490164	1.09E-05	2	Dap3
-0.363210492	0.147193528	1.10E-05	2	Macf1

-0.519059491	0.151758294	1.14E-05	2	Cav2
-0.512702854	0.157340849	1.18E-05	2	Hsd17b10
-0.381932622	0.163306357	1.22E-05	2	Cmip
-0.323326216	0.167573591	1.26E-05	2	Npm1
-0.385326499	0.168059703	1.26E-05	2	Galnt7
-0.389852889	0.168358397	1.26E-05	2	Hmgn5
-0.299162159	0.169044918	1.27E-05	2	Wdr1
-0.384003409	0.174139069	1.30E-05	2	Scarb2
-0.344931397	0.187439821	1.40E-05	2	Eif6
-0.353953454	0.187900825	1.41E-05	2	Usp14
-0.510614948	0.189204906	1.42E-05	2	Mark3
-0.315916468	0.190975713	1.43E-05	2	Baiap2
-0.507996644	0.193263143	1.45E-05	2	Fem1b
-1.129084023	0.194339508	1.46E-05	2	Pcp4l1
-0.318381249	0.194451275	1.46E-05	2	Hn1
-0.37645764	0.199823282	1.50E-05	2	Vapa
-0.347495905	0.199875276	1.50E-05	2	Polr1d
-0.556402652	0.201182602	1.51E-05	2	Brpf1
-0.567306988	0.206343292	1.55E-05	2	Ppfibp1
-0.335371944	0.208108255	1.56E-05	2	Serbp1
-0.399681031	0.210996845	1.58E-05	2	Cenpa
-0.567507328	0.21772948	1.63E-05	2	Enoph1
-0.423846037	0.2186071	1.64E-05	2	Mdfic
0.411527265	0.223455303	1.67E-05	2	Lmo2
-0.447297106	0.226537463	1.70E-05	2	Zmat5
-0.363749473	0.231574931	1.73E-05	2	Ndufb8
-0.297325578	0.233170482	1.75E-05	2	Zfp207
-0.407454729	0.237167343	1.78E-05	2	Rpl18a
-0.522419168	0.2491358	1.87E-05	2	Prnp
-0.258145253	0.258838367	1.94E-05	2	Srp19
-0.264284847	0.263380949	1.97E-05	2	Itgb1
0.254481676	0.263574584	1.97E-05	2	App
0.39646492	0.269442851	2.02E-05	2	Irf2bp2
-0.935269858	0.271936898	2.04E-05	2	Ly6c2
-0.305761399	0.272297493	2.04E-05	2	Ube2h
-0.443729571	0.272799477	2.04E-05	2	Tmem134
-0.343917708	0.273735084	2.05E-05	2	Cds2
0.432285667	0.275160171	2.06E-05	2	Adrbk1
0.371455604	0.280870587	2.10E-05	2	Idh1
-0.519133744	0.288687421	2.16E-05	2	Mrpl35
-0.675667098	0.289059864	2.16E-05	2	Sowahc
0.450883849	0.292131176	2.19E-05	2	Vrk2
-0.324110502	0.2922926	2.19E-05	2	Arl8b
-0.474688397	0.296090924	2.22E-05	2	Fbxo6
-1.286322511	0.299388112	2.24E-05	2	H2-Oa
-0.290452847	0.300145392	2.25E-05	2	Atp5c1
-0.378820125	0.300744035	2.25E-05	2	Atp6v0d1
-0.447774159	0.301819374	2.26E-05	2	Gtf2b
-0.83662719	0.302937646	2.27E-05	2	Arap2
0.918350695	0.307698659	2.30E-05	2	Kitl
-0.254608874	0.312580115	2.34E-05	2	Ola1
-0.378087804	0.315022865	2.36E-05	2	Hcfc1r1
-0.38340213	0.316887301	2.37E-05	2	Txnl1
0.580968313	0.31961122	2.39E-05	2	Rnf150
0.513284114	0.323377442	2.42E-05	2	Cd36
0.67735612	0.323632427	2.42E-05	2	Mtus1
-0.475227515	0.329076041	2.46E-05	2	Nop58
-0.378076304	0.329288164	2.47E-05	2	C1qbp
-0.269087664	0.334993101	2.51E-05	2	Tubb4b
0.461105349	0.33642498	2.52E-05	2	Rbpj
-0.730121156	0.33805101	2.53E-05	2	Slc35c2

-0.378845735	0.338156948	2.53E-05	2	Cct2
0.679754695	0.340906834	2.55E-05	2	Nuak1
-0.258733406	0.343014081	2.57E-05	2	Eif4g2
-0.512304526	0.343608911	2.57E-05	2	Hck
-0.754729186	0.349726631	2.62E-05	2	Phlpp1
-0.40226415	0.349814948	2.62E-05	2	Snrpd3
-0.325376672	0.350059046	2.62E-05	2	Rnf19b
-0.682620901	0.355821758	2.66E-05	2	Mrpl38
-0.507141683	0.357692591	2.68E-05	2	Lactb
0.410566662	0.360414559	2.70E-05	2	Laptm4a
-0.426187631	0.366553637	2.75E-05	2	Rsl1d1
-0.345428803	0.366561587	2.75E-05	2	Riok3
-0.40049591	0.370487708	2.77E-05	2	Lsm3
-0.593143595	0.378085332	2.83E-05	2	Ppa1
-0.356998206	0.381476792	2.86E-05	2	Eny2
0.692263	0.384898184	2.88E-05	2	S1pr1
0.550043013	0.389122704	2.91E-05	2	Arhgef6
0.70843375	0.389651725	2.92E-05	2	Cd38
-0.658199612	0.392317245	2.94E-05	2	Fem1c
-0.863090795	0.39348271	2.95E-05	2	Coro2a
-0.379376028	0.397741858	2.98E-05	2	Igsf8
-0.586389558	0.410205263	3.07E-05	2	Ak6
-0.296396856	0.416472374	3.12E-05	2	Ppp1ca
-0.449397543	0.416895588	3.12E-05	2	H2-DMb1
0.298433455	0.418061927	3.13E-05	2	Rsad2
-0.291679808	0.420342584	3.15E-05	2	Cox7a2
-0.540210674	0.420917688	3.15E-05	2	Jdp2
-0.618897613	0.422128253	3.16E-05	2	Gatad2a
-0.900057743	0.425603795	3.19E-05	2	Nr1h3
-0.33381166	0.428402182	3.21E-05	2	Ndufv2
-0.301358117	0.447733809	3.35E-05	2	Mdh1
-0.385234558	0.464551952	3.48E-05	2	Anxa7
-0.448703776	0.465240526	3.48E-05	2	Ccni
0.929184819	0.470897178	3.53E-05	2	Maged1
0.572890725	0.474936559	3.56E-05	2	Irf2
-0.371432388	0.475458568	3.56E-05	2	Nup50
-0.477003396	0.476645424	3.57E-05	2	Usmg5
0.297477168	0.479036213	3.59E-05	2	4632428N05Rik
-0.60292489	0.482772679	3.62E-05	2	Socs6
-0.794533337	0.496491053	3.72E-05	2	Gpr171
-0.526474843	0.497957764	3.73E-05	2	Gm11974
-0.423737452	0.498834379	3.74E-05	2	Gtf2h5
-0.340758604	0.506781111	3.80E-05	2	Mrps14
-0.4373918	0.51086339	3.83E-05	2	Otulin
-0.549081535	0.511831349	3.83E-05	2	Cetn2
-0.390385758	0.532454062	3.99E-05	2	Cyp4v3
0.33113387	0.534746369	4.00E-05	2	Zeb2
0.654568584	0.540461531	4.05E-05	2	Ptprj
-0.326066564	0.540759113	4.05E-05	2	Zmiz2
-0.251290425	0.559735861	4.19E-05	2	Rabggtb
-0.402013618	0.582151156	4.36E-05	2	Dnase1l1
-0.268794045	0.596420489	4.47E-05	2	Cct4
-0.347139026	0.601928792	4.51E-05	2	Vps26a
-0.403890253	0.609480465	4.56E-05	2	Slc35e4
-0.451731299	0.626922615	4.70E-05	2	Frrs1
-1.133526047	0.629271934	4.71E-05	2	Gdf3
-0.537920886	0.630372013	4.72E-05	2	Eif4e2
0.477247829	0.630474905	4.72E-05	2	Fli1
-0.566989765	0.636079747	4.76E-05	2	Ccrn4l
-0.559482682	0.640212067	4.79E-05	2	Atp11b
-0.443262136	0.643847522	4.82E-05	2	Ube2k

-0.514536638	0.652760655	4.89E-05	2	BC028528
-0.36886098	0.656839904	4.92E-05	2	Ptpmt1
-0.673199889	0.683392314	5.12E-05	2	Nostrin
-0.696313901	0.687870804	5.15E-05	2	Lmf1
-0.602984503	0.705546019	5.28E-05	2	Plaa
-0.52807367	0.707767451	5.30E-05	2	Zc3h12a
-0.277761151	0.711417689	5.33E-05	2	Ugp2
-0.749653712	0.713062846	5.34E-05	2	Mbp
-0.310381512	0.725327676	5.43E-05	2	Hspa9
-0.448987653	0.733276187	5.49E-05	2	Gtf2f2
-0.612443923	0.751650584	5.63E-05	2	Lsm1
-0.964515459	0.752475621	5.64E-05	2	Sep.06
-0.268006744	0.758474734	5.68E-05	2	Mex3c
-0.93964511	0.764003149	5.72E-05	2	Sorl1
-0.512200506	0.77895779	5.83E-05	2	Rasa2
-0.273601455	0.780247701	5.84E-05	2	Selk
-0.416680207	0.780571095	5.85E-05	2	Ccdc23
-0.984671506	0.794454134	5.95E-05	2	Itgal
-0.670224711	0.842650424	6.31E-05	2	Snx20
-0.684384318	0.864901529	6.48E-05	2	Pip5k1a
-0.297573656	0.867439915	6.50E-05	2	Zfp326
0.905801057	0.882165633	6.61E-05	2	Rnf169
0.85355152	0.923034525	6.91E-05	2	Evi5
-0.349335904	0.923960598	6.92E-05	2	Tmem14c
-0.290675103	0.925346403	6.93E-05	2	Ube2f
0.493627673	0.927736611	6.95E-05	2	Rnf145
0.395134743	0.930666892	6.97E-05	2	Rnf167
0.421758247	0.93228297	6.98E-05	2	Nfia
-0.334748878	0.937827456	7.02E-05	2	Ghitm
0.709565099	0.943393919	7.07E-05	2	Dalrd3
-0.411152151	0.947970308	7.10E-05	2	Prpf38a
0.281312246	0.952143799	7.13E-05	2	Ms4a6c
**0.571870252**	**0.978300756**	**7.33E-05**	**2**	**Fcgr1**
-0.32574505	0.988436808	7.40E-05	2	Tmem208
0.611036385	0.994134283	7.45E-05	2	Nfatc2
-0.275588644	1	7.54E-05	2	Fndc3b
-0.321602768	1	7.55E-05	2	Rpf1
-0.371333615	1	7.63E-05	2	Psme2b
-0.383623955	1	7.65E-05	2	Tmc6
-0.273418548	1	7.74E-05	2	Uqcrh
-0.374418007	1	7.75E-05	2	Psma2
0.397859243	1	7.84E-05	2	Il6ra
-0.503600297	1	7.93E-05	2	Nsf
-0.548314818	1	8.06E-05	2	Cpeb2
-0.320198514	1	8.14E-05	2	Cope
-0.333196618	1	8.17E-05	2	Mpc2
-0.305477586	1	8.18E-05	2	Spcs2
-0.440689758	1	8.28E-05	2	Htatip2
-0.303955472	1	8.43E-05	2	Psme3
0.354219739	1	8.43E-05	2	Siglece
-0.346482499	1	8.52E-05	2	Dnmt3a
0.555653256	1	8.67E-05	2	Fert2
-0.357795491	1	8.74E-05	2	Dyrk1a
-0.351052357	1	8.88E-05	2	Mlf2
-0.25343417	1	9.09E-05	2	Jkamp
-0.696648183	1	9.09E-05	2	Letm1
0.483635792	1	9.19E-05	2	Morc3
-0.521393632	1	9.20E-05	2	Chchd4
-0.331961209	1	9.35E-05	2	Atp5g3
-0.392072902	1	9.41E-05	2	Ifi27

-0.481672845	1	9.73E-05	2	Gnptab
-0.326650056	1	9.75E-05	2	Dmxl2
0.521630116	1	9.83E-05	2	Rassf4
0.479639206	1	9.89E-05	2	Uba7
-0.571082418	1	9.91E-05	2	Glipr2
-0.518741202	1	9.93E-05	2	Chic2
0.467928247	1	0.0001008	2	Samd9l
-0.27158721	1	0.0001012	2	Rab8a
-0.403087939	1	0.0001019	2	Rcan1
-0.277774518	1	0.0001022	2	Lpxn
-0.612797155	1	0.0001026	2	Rpl36al
-0.307582343	1	0.0001039	2	Psmd12
-0.403831425	1	0.0001064	2	Cox19
-0.411353657	1	0.0001067	2	Ppp2cb
0.517188247	1	0.0001083	2	Epb4.1l1
-0.438331331	1	0.000111	2	Emc3
-0.886746024	1	0.0001116	2	Dock5
0.410372985	1	0.0001119	2	Ankfy1
-0.496097854	1	0.0001121	2	B4galnt1
-0.500316945	1	0.0001122	2	Paf1
-0.674349273	1	0.000113	2	Snhg6
-0.285464163	1	0.0001138	2	Prdx5
0.345590567	1	0.0001151	2	Pou2f2
-0.309330334	1	0.0001153	2	Polr2i
-0.330797049	1	0.0001163	2	Stx8
-0.316665105	1	0.000119	2	Creg1
0.557818132	1	0.0001212	2	Xdh
0.678862947	1	0.0001233	2	Itfg3
-0.598454586	1	0.0001248	2	Ero1lb
-0.286816238	1	0.0001252	2	1810058I24Rik
-0.372393853	1	0.0001254	2	Elovl5
-0.477990704	1	0.0001267	2	Eif3l
-0.549528463	1	0.0001287	2	Srebf1
0.335092003	1	0.0001301	2	Dhrs3
-0.364909568	1	0.000131	2	Sec61b
0.680185235	1	0.0001312	2	Fchsd2
-0.254605726	1	0.0001337	2	Cox8a
-0.483522744	1	0.0001352	2	Esyt2
0.634901233	1	0.0001354	2	Zfp217
-0.322093658	1	0.0001359	2	Ranbp2
-0.261790041	1	0.0001361	2	Gm12338
0.459080274	1	0.0001363	2	Tlr4
-0.290231857	1	0.0001374	2	Ngdn
0.709605357	1	0.0001376	2	Sh3bp5
0.656246234	1	0.0001377	2	Pdgfc
0.668604257	1	0.0001398	2	Fam111a
-0.527031469	1	0.0001406	2	Sec24b
-0.339450843	1	0.0001425	2	Mrps6
-0.370786779	1	0.0001427	2	Vdac1
0.858143666	1	0.0001429	2	Agtrap
-0.466224056	1	0.0001429	2	Nfkbib
-0.49503885	1	0.0001446	2	1110008P14Rik
-0.543612877	1	0.0001454	2	Hivep1
-0.283281319	1	0.0001465	2	Smek2
-0.48157831	1	0.0001472	2	Snhg1
-0.373237096	1	0.0001524	2	Rps2
-0.476670786	1	0.0001525	2	Atp5g1
0.426354487	1	0.0001535	2	Aftph
-0.314680327	1	0.0001557	2	Degs1
0.681837891	1	0.0001558	2	Acat1
-0.747966374	1	0.000156	2	Osgin2

0.394328737	1	0.0001583	2	Ddx26b
-0.26427139	1	0.0001596	2	Sec11a
-0.572405391	1	0.0001607	2	Atp6v0a2
-0.435849542	1	0.0001621	2	Apbb1ip
-0.284763787	1	0.0001634	2	Bcl3
-0.589069218	1	0.0001699	2	Rasa4
-0.311334443	1	0.0001712	2	Zfp715
-0.286692121	1	0.0001723	2	Ppp2r5c
-0.437944641	1	0.0001743	2	Zc3hav1
-0.428911468	1	0.0001746	2	Orc4
-0.265408842	1	0.0001776	2	Ndufa12
-0.36540456	1	0.0001797	2	Polr1c
0.916431666	1	0.0001802	2	Rab24
-0.450601507	1	0.000183	2	Cops5
-0.756548007	1	0.0001852	2	Fmnl2
-0.31296814	1	0.0001859	2	Alg5
-0.354749888	1	0.0001863	2	Irf2bpl
-0.362836065	1	0.0001867	2	Cd300lb
-0.632994014	1	0.0001915	2	Mif
-0.661976156	1	0.0001966	2	Eva1b
0.621202388	1	0.0001972	2	Apoe
-0.334713	1	0.0002009	2	Srrm1
-0.539150049	1	0.0002036	2	Sec23b
-0.272553347	1	0.0002039	2	Tusc2
-0.296014096	1	0.0002041	2	Spcs1
-0.58946955	1	0.0002066	2	Evl
-0.30097248	1	0.0002114	2	Tmem184b
0.653642341	1	0.0002157	2	Tsc22d4
-0.271330171	1	0.0002176	2	Sf3b1
-0.576857276	1	0.000218	2	2510009E07Rik
-0.445270253	1	0.0002211	2	Diap1
-0.769695585	1	0.0002284	2	Abi3
-0.377468268	1	0.000234	2	Sub1
-0.491587735	1	0.0002341	2	Myl6
-0.34691633	1	0.0002355	2	Psmc1
0.574348449	1	0.0002369	2	Sec14l1
-0.458058939	1	0.0002375	2	Gatad1
-0.305680395	1	0.0002388	2	Ndufb6
0.690372028	1	0.0002401	2	Tk2
-0.560705926	1	0.0002431	2	Rin3
-0.418606738	1	0.0002446	2	Lonrf3
-0.703823411	1	0.0002461	2	Dgat1
-0.281740269	1	0.0002478	2	Tfg
-0.295345183	1	0.0002505	2	Atp6v1f
-0.306731839	1	0.0002525	2	Pdcl3
-0.337555361	1	0.0002546	2	1110008F13Rik
-0.290631891	1	0.0002566	2	Senp5
-0.552207708	1	0.000259	2	Spsb2
-0.262650519	1	0.0002594	2	Got2
0.55286734	1	0.0002674	2	Osbpl9
-0.448560495	1	0.0002675	2	Exoc2
-0.292076293	1	0.0002698	2	Mphosph10
-0.475742913	1	0.0002749	2	Tceb3
-0.352997141	1	0.0002766	2	Cs
-0.272800129	1	0.0002766	2	Ech1
-0.258120718	1	0.0002817	2	Shfm1
-0.335668546	1	0.0002838	2	Naa50
-0.334605655	1	0.0002845	2	Atxn10
-0.368080001	1	0.0002847	2	Pgk1
-0.27873564	1	0.0002915	2	Pgls
-0.430062103	1	0.000292	2	Fam63a

-0.588767436	1	0.000295	2	Wbp1l
-0.350125716	1	0.0002958	2	Stk10
0.539485118	1	0.0002994	2	Cd302
-0.281283611	1	0.0003018	2	Apobec1
-0.673575867	1	0.0003019	2	Nxt1
-0.476382577	1	0.0003044	2	Snrpa1
-0.380886423	1	0.0003085	2	Mrps28
-0.310080485	1	0.0003087	2	Irf5
-0.301345104	1	0.000325	2	Rps27l
-0.546636663	1	0.0003324	2	Tcp11l2
0.254539431	1	0.000334	2	AI607873
0.360204195	1	0.000338	2	Neat1
-0.353329525	1	0.0003407	2	Ubqln1
-0.261317628	1	0.0003413	2	Polr2g
-0.282850334	1	0.0003555	2	Stt3b
-0.261916909	1	0.0003561	2	Eif3a
-0.426653941	1	0.0003576	2	Lmnb1
0.541738217	1	0.0003592	2	Oasl2
-0.410932801	1	0.0003599	2	Nemf
-0.337582662	1	0.0003614	2	Psmc5
-0.410219388	1	0.0003623	2	Mtmr14
-0.302421894	1	0.0003663	2	Cpeb4
-0.333360903	1	0.0003788	2	Tsta3
-0.673574773	1	0.0003821	2	Dusp2
0.418658175	1	0.0003843	2	Ier2
-0.767691252	1	0.0003857	2	Tle1
-0.560003965	1	0.0003879	2	Scyl2
-0.564572019	1	0.0003933	2	Scimp
0.472548417	1	0.0003952	2	Fcho2
-0.338768315	1	0.0003981	2	Npepps
-0.309749789	1	0.0003983	2	Ssr1
-0.648955318	1	0.0004028	2	Mical1
-0.351524258	1	0.0004055	2	Hnrnpc
-0.311424763	1	0.0004067	2	Paip2
0.294945794	1	0.0004085	2	Ptpn18
0.709190181	1	0.0004139	2	Snx13
0.809143097	1	0.0004175	2	Micu1
-0.445112198	1	0.0004256	2	Ap1s1
-0.307181704	1	0.0004294	2	Kpna3
-0.673309398	1	0.0004317	2	Yars
-0.305357848	1	0.000433	2	Arid5b
0.444203945	1	0.0004496	2	Clec4a2
-1.0481551	1	0.0004498	2	Klhdc4
0.350041195	1	0.0004528	2	Atxn7l3b
-0.471402341	1	0.0004562	2	Mpp5
0.557969869	1	0.0004603	2	Add3
-0.422341665	1	0.0004631	2	Tmx4
-0.482958389	1	0.0004671	2	Cdk2ap2
-0.465780853	1	0.00047	2	Itpk1
-0.286661443	1	0.0004705	2	Gtf3a
-0.330419188	1	0.0004756	2	Setd3
-0.470639394	1	0.0004821	2	Ltv1
0.392350207	1	0.0004899	2	Aes
-0.494342832	1	0.0004966	2	Mllt6
-0.569602523	1	0.000499	2	Olfml3
-0.273854337	1	0.0005088	2	Rala
-0.657212363	1	0.0005238	2	Kansl3
-0.255750631	1	0.0005246	2	Txndc9
-0.559039747	1	0.000526	2	4833439L19Rik
-0.298984998	1	0.0005268	2	Cers5
-0.288443165	1	0.0005273	2	Polr2f

0.408166411	1	0.0005304	2	Bbx
-0.568795672	1	0.0005306	2	Slain2
-0.292246753	1	0.0005419	2	Piezo1
-0.422664115	1	0.0005455	2	Ino80
-0.425365584	1	0.0005557	2	2310022A10Rik
1.016699341	1	0.0005614	2	Tstd2
-0.339696668	1	0.0005626	2	Tmem167
0.539428989	1	0.0005723	2	Adam19
-0.260544326	1	0.0005738	2	Glrx5
-0.379930186	1	0.0005742	2	Bub3
-0.292050027	1	0.0005776	2	Baz1a
-0.500093145	1	0.0005908	2	Syne1
-0.34859467	1	0.0005926	2	Vps28
-0.329216868	1	0.0005961	2	Mtch2
-0.279198974	1	0.0006033	2	Ppm1h
0.296670313	1	0.0006042	2	Zswim6
-0.30808769	1	0.0006109	2	Il17ra
-0.317982164	1	0.0006146	2	Srsf7
-0.318993935	1	0.0006159	2	Med10
-0.403334169	1	0.0006435	2	Prelid1
0.390804008	1	0.0006449	2	Filip1l
-0.3520044	1	0.0006499	2	Clpp
-1.043940346	1	0.0006596	2	Samsn1
-0.620625608	1	0.0006612	2	Pgs1
-0.271483446	1	0.0006642	2	Etf1
-0.708877446	1	0.0006711	2	Tmem119
0.264762885	1	0.000678	2	Trim30d
0.731294947	1	0.0006855	2	Jup
-0.398852564	1	0.0006869	2	Emc4
-0.306541175	1	0.0006894	2	Atp5j
0.545700187	1	0.0006901	2	Tspan3
-0.397442584	1	0.0006952	2	Ube2j1
-0.375118635	1	0.0007053	2	Arid4b
-0.25686526	1	0.0007091	2	Naa38
-0.356964867	1	0.0007101	2	Xpo1
-0.323658563	1	0.0007108	2	Ppp6c
-0.270229882	1	0.0007144	2	Insig2
-0.553510669	1	0.0007235	2	Samd8
-0.286348807	1	0.0007298	2	Mrpl37
-0.499543645	1	0.0007313	2	Mbtd1
-0.387037914	1	0.0007401	2	Msra
-0.473045181	1	0.0007444	2	Cpsf6
-0.307270183	1	0.0007472	2	Tma7
-0.323351069	1	0.0007543	2	Atp5l
0.582992084	1	0.0007568	2	Fkbp15
0.276814824	1	0.0007642	2	Sft2d2
-0.709366101	1	0.0007698	2	Ubxn2a
0.61350728	1	0.0007717	2	Prkab1
0.514225211	1	0.0007757	2	Smim15
-0.262989555	1	0.0007766	2	Odf2
-0.368573356	1	0.0007819	2	Thoc2
-0.257837525	1	0.0007851	2	Gm3258
-0.614110031	1	0.0007881	2	AW112010
0.406384463	1	0.0007927	2	Ppp1r12c
-0.311650082	1	0.0008007	2	Aph1c
0.477781419	1	0.0008226	2	Rab1b
-0.478982365	1	0.0008306	2	Gga2
-0.450665945	1	0.0008343	2	Rfwd2
-0.782621307	1	0.0008343	2	Il1a
0.804766953	1	0.0008389	2	2810025M15Rik
-0.307807976	1	0.0008396	2	Atp5d

0.492313334	1	0.0008442	2	Clasp2
-0.350707245	1	0.0008464	2	Rars
-0.298615171	1	0.0008681	2	Mrpl4
-0.389211569	1	0.0008708	2	Dot1l
-0.447887314	1	0.0008724	2	Bhlhe41
-0.389198831	1	0.0008811	2	Higd1a
-0.472438356	1	0.0008975	2	Bop1
-0.267289667	1	0.0009039	2	Chchd3
-0.368906979	1	0.0009114	2	Lage3
-0.431903812	1	0.0009133	2	Abcb7
-0.306146756	1	0.0009386	2	Med28
-0.575709237	1	0.0009456	2	Ramp1
0.35099809	1	0.0009788	2	Tifab
-0.255384438	1	0.0010454	2	Cox6c
-0.569600938	1	0.0010605	2	Pop4
-0.4202542	1	0.0010607	2	B9d2
-0.444524964	1	0.0010642	2	Otud6b
-0.364416536	1	0.0010656	2	Tbpl1
0.425384365	1	0.0010758	2	Gtf2i
-0.270241541	1	0.0010794	2	Asap1
-0.348051431	1	0.0011012	2	Rbbp6
-0.292936309	1	0.001103	2	Mrpl54
0.789336895	1	0.0011143	2	Polg
0.345360397	1	0.0011301	2	Pcnp
-0.460033185	1	0.0011337	2	Mir22hg
0.616849601	1	0.0011446	2	Ephx1
-0.362450348	1	0.0011499	2	Mmd
-0.38012398	1	0.0011543	2	Mien1
0.591294591	1	0.0011712	2	Baz1b
0.390430388	1	0.0011744	2	Serpinb6a
-0.490552856	1	0.0011823	2	Avpi1
0.362950233	1	0.0011846	2	Rab7l1
-0.358986445	1	0.0011929	2	Akip1
-0.343201985	1	0.0012156	2	Gcc2
-0.26600505	1	0.0012237	2	Mrpl36
-0.441001588	1	0.0012475	2	Gas2l3
0.385845882	1	0.0012563	2	Gpr183
-0.429596641	1	0.0012751	2	Nrd1
0.346867791	1	0.0013149	2	Ubtf
0.383056118	1	0.0013179	2	Igf1r
-0.354276658	1	0.0013369	2	Josd1
-0.284484048	1	0.0013379	2	Npc1
0.585341854	1	0.0013429	2	Mid1ip1
0.467689437	1	0.0013457	2	Cd2ap
-0.432133775	1	0.0013503	2	Desi2
-0.320655387	1	0.0013512	2	2300009A05Rik
0.401648438	1	0.0013569	2	Epn1
-0.435284408	1	0.0013607	2	N4bp1
-0.681116775	1	0.0013783	2	Ncapg2
-0.261709335	1	0.0013864	2	Map3k2
-0.36045265	1	0.0013973	2	Cep83
-0.259767874	1	0.0014139	2	Minos1
-0.322977729	1	0.0014231	2	Pcmt1
-0.448069069	1	0.0014461	2	Fhod1
0.768421886	1	0.0014573	2	Ppip5k2
0.346309234	1	0.0014639	2	Cdr2
-0.281241541	1	0.0014732	2	Mrpl34
0.273129224	1	0.0014739	2	Gxylt1
0.314941532	1	0.0014894	2	Ncf1
-0.330252256	1	0.0014999	2	Kpna1
0.374624085	1	0.0015026	2	Tpcn1

-0.327140711	1	0.0015064	2	Wdr43
0.456538845	1	0.0015125	2	Atg13
0.331955125	1	0.0015262	2	Zbp1
-0.296152236	1	0.0015265	2	Slc39a6
0.665879869	1	0.0015316	2	Zbtb4
-0.361459038	1	0.0015463	2	Mvp
-0.364587833	1	0.0016244	2	Slc35b2
-0.432167399	1	0.0016251	2	Mrpl17
-0.336812187	1	0.0016369	2	Polr2j
-0.265176663	1	0.001637	2	Acsl4
-0.379621856	1	0.0016887	2	Ints12
-0.256866468	1	0.0017052	2	Cxcl2
-0.263785383	1	0.0017129	2	St3gal1
-0.372594241	1	0.0017187	2	Cds1
-0.311751614	1	0.0017202	2	Ngfrap1
-0.463606464	1	0.0017206	2	Cdc42ep4
-0.321962726	1	0.0017266	2	Rpl18
-0.320865089	1	0.0017451	2	Tmem192
-0.386211945	1	0.0017532	2	Dda1
-0.339933465	1	0.0017894	2	Polr3k
-0.312147703	1	0.0017998	2	Zmym2
0.772602585	1	0.0018449	2	Galk1
0.62314578	1	0.0018462	2	Dnajc13
-0.458531328	1	0.0018567	2	Bola3
0.511916863	1	0.001862	2	Tmem55b
-0.347725273	1	0.0018706	2	Mknk2
0.782098179	1	0.0018839	2	Prune2
0.624087962	1	0.0019063	2	Rere
-0.383574389	1	0.001919	2	Ahcyl2
-0.38156306	1	0.0019241	2	Dusp16
-0.31942269	1	0.0019256	2	Tnf
-0.591529387	1	0.0019325	2	Polr2b
0.559794555	1	0.0019361	2	2700089E24Rik
-0.254139744	1	0.0019494	2	H2-Ab1
-0.288024382	1	0.0019512	2	Zfand3
-0.372749088	1	0.001954	2	Fopnl
-0.276572107	1	0.001989	2	Katna1
0.345558263	1	0.0019916	2	Hip1
0.579918202	1	0.0020097	2	Rasa1
-0.287739465	1	0.0020307	2	Mrpl45
-0.282945615	1	0.0020445	2	Zbtb11
-0.39318578	1	0.0020624	2	Dnajb6
0.250485719	1	0.0021084	2	Naaa
-0.271303122	1	0.0021109	2	Cnppd1
-0.42459975	1	0.0021232	2	Ftsj3
0.454437066	1	0.0021288	2	Dnajb1
-0.302084676	1	0.0021331	2	Ythdf1
-0.327221878	1	0.0021391	2	Selplg
0.624664117	1	0.0021517	2	Dcxr
-0.493495075	1	0.0021575	2	Mgrn1
-0.458467315	1	0.0021823	2	Exosc9
0.283815584	1	0.0021978	2	Tdrd7
-0.437669572	1	0.002199	2	Mmadhc
-0.459335479	1	0.0022363	2	Dst
-0.293167911	1	0.0022494	2	Wac
-0.289352688	1	0.0022519	2	Chmp2b
-0.322705051	1	0.0022537	2	1500011K16Rik
-0.273144785	1	0.0022551	2	Ranbp1
0.554498632	1	0.0022581	2	Slc29a1
-0.427596657	1	0.0022654	2	Rpl19
-0.26738707	1	0.0022734	2	Pbrm1

-0.267383681	1	0.0023376	2	Med13l
0.462920795	1	0.0023397	2	Kif1b
-0.990413602	1	0.0023522	2	Grap2
-0.409564561	1	0.0023746	2	Tssc1
-0.39679977	1	0.002412	2	Fau
0.691861242	1	0.0024266	2	Cmklr1
-0.606223292	1	0.0024292	2	Zfp516
-0.251017914	1	0.0024386	2	Hspbp1
-0.607787427	1	0.0024463	2	Pls3
0.694001382	1	0.0024528	2	Pde1b
-0.533778357	1	0.002455	2	Pdgfb
-0.283152156	1	0.0024602	2	Snx17
-0.318355533	1	0.0024651	2	Cln5
-0.267379659	1	0.0025063	2	Tspan14
-0.288791452	1	0.0025211	2	Ssu72
-0.26472923	1	0.0025732	2	Isy1
0.727498021	1	0.0025765	2	Slc43a2
-0.315337093	1	0.002579	2	Slc11a1
0.557126954	1	0.0026093	2	Mgat1
-0.426785114	1	0.0026102	2	Samd4b
-0.403790557	1	0.0026241	2	Gyg
0.520532286	1	0.0026308	2	Rab31
-0.49326677	1	0.0026459	2	Pik3r1
-0.431042703	1	0.0026636	2	Srpr
-0.747392396	1	0.002693	2	Ptafr
-0.298206669	1	0.0027264	2	Kctd10
-0.436955044	1	0.0027284	2	Col14a1
-0.409588661	1	0.0027751	2	Eif3b
-0.40132479	1	0.0027821	2	Hmgcl
-0.258516205	1	0.0027987	2	Qdpr
-0.559202362	1	0.0028095	2	2310045N01Rik
-0.439091036	1	0.0028149	2	Mllt4
0.377693384	1	0.002823	2	Ypel3
-0.302948676	1	0.0028804	2	Sar1b
-0.274928838	1	0.0028947	2	Ufd1l
-0.346029511	1	0.0029064	2	Cmas
-0.290501786	1	0.0029615	2	Rchy1
-0.324141782	1	0.0029915	2	Inpp5b
-0.438238012	1	0.0030066	2	Spast
-0.424056436	1	0.0030233	2	D16Ertd472e
-0.264727422	1	0.0030293	2	Mad2l2
-0.260975737	1	0.0030367	2	Copg1
-0.598330786	1	0.0030633	2	Itgb1bp1
-0.475378958	1	0.0030727	2	Mtfr1l
0.40782849	1	0.003163	2	Tmem141
-0.255072197	1	0.0032037	2	Rpp21
-0.517653359	1	0.0032297	2	Dync1li1
0.443592015	1	0.0032585	2	Fbxw4
0.464828671	1	0.0032657	2	Pla2g16
-0.25567718	1	0.0033961	2	Vat1
0.342013707	1	0.0033987	2	Cd37
-0.389342752	1	0.0034154	2	Bcas2
-0.322639834	1	0.0034283	2	Klhdc3
0.941008798	1	0.0034385	2	Phyhd1
-0.277630868	1	0.0034413	2	Coa5
-0.436444832	1	0.003446	2	Vps26b
-0.338030798	1	0.0035047	2	Rps19
-0.589087692	1	0.0035298	2	Tbc1d1
0.397359826	1	0.0035992	2	Osbpl11
-0.819135355	1	0.0036358	2	Stat4
-0.391081817	1	0.0036681	2	Olfm1

0.341445888	1	0.0037123	2	Sdc3
0.264946404	1	0.0037266	2	Asah1
-0.285285825	1	0.0037338	2	Rbbp7
-0.374440086	1	0.0037498	2	Zfp592
-0.449121211	1	0.0037698	2	Fcgr4
0.442160703	1	0.0037724	2	Txndc16
0.514620395	1	0.0038031	2	Dync1li2
-0.653246951	1	0.0039417	2	Smad7
0.379474304	1	0.0039931	2	Ccdc134
0.536043146	1	0.0040336	2	Lman1
-0.251198434	1	0.0040851	2	Mrpl24
-0.382206762	1	0.0041236	2	Hat1
-0.251322794	1	0.0041251	2	Usp15
-0.422365401	1	0.0041402	2	Wdr48
0.283653937	1	0.004186	2	Kat6a
-0.312420734	1	0.0042007	2	Gpaa1
-0.328998938	1	0.0042093	2	Esco1
-0.535576074	1	0.0042108	2	Ube2d2a
-0.333430715	1	0.0042363	2	Ptp4a1
-0.433214428	1	0.0042952	2	Srf
0.487199075	1	0.0043165	2	Pkp4
-0.493261048	1	0.0043365	2	Creb5
-0.382590132	1	0.0043784	2	Hnrnpdl
0.402723181	1	0.0043792	2	Psmb9
-0.492548103	1	0.004418	2	Hscb
-0.300944251	1	0.0044247	2	Rsrc2
-0.27335001	1	0.0044943	2	Snx18
-0.264507702	1	0.0045757	2	Hbp1
-0.331863106	1	0.0046247	2	Trnau1ap
-0.297265688	1	0.0047783	2	Usp7
-0.268844717	1	0.0047795	2	Ikbkb
-0.284525035	1	0.0047907	2	Lamtor4
-0.261080288	1	0.004882	2	Ccdc53
0.452565917	1	0.0049387	2	Ncoa1
-0.56610182	1	0.0049924	2	Pmaip1
0.676895045	1	0.0050001	2	Mon2
-0.279436636	1	0.005009	2	Eif3i
-0.533453798	1	0.0050538	2	Lyar
-0.477998155	1	0.0050909	2	Pde1b
0.591854348	1	0.0051302	2	Sh3pxd2a
0.395146152	1	0.0051479	2	Nav1
0.446836117	1	0.0051905	2	Zeb2os
-0.454630917	1	0.0052338	2	Prkrir
0.655879019	1	0.0053007	2	Arhgap12
-0.26600505	1	0.0053187	2	Fam175b
-0.343920511	1	0.0053191	2	Trim27
-0.374118581	1	0.0053381	2	Fam134b
-0.401023504	1	0.0053439	2	Fubp1
-0.433946463	1	0.0053648	2	1700025G04Rik
-0.446258137	1	0.0053801	2	Rpl27a
-0.444477736	1	0.0053885	2	Cherp
-0.32377362	1	0.0054032	2	Aco2
-0.566514067	1	0.0054586	2	Tjp2
-0.338489102	1	0.0055622	2	Dohh
-0.562327729	1	0.005608	2	Lrch1
-0.419132708	1	0.0057013	2	Otud5
-0.363872126	1	0.0057044	2	Fxr2
0.859863918	1	0.0057457	2	Itpripl1
0.27322606	1	0.0057895	2	Ergic3
0.504648812	1	0.0058131	2	Clec4b1
-0.334771645	1	0.0058195	2	Prkaa1

-0.30126687	1	0.0059521	2	Ptrhd1
0.628786807	1	0.0059628	2	Tnfaip8l2
-0.267957446	1	0.0059713	2	Ndufb2
-0.322526632	1	0.0059946	2	Adap1
0.353646304	1	0.0061324	2	Crebrf
-0.545023677	1	0.0062859	2	Cspp1
-0.371679193	1	0.0063536	2	H2afy
-0.296665425	1	0.0064476	2	Mrpl32
-0.393144382	1	0.0064713	2	Nr4a1
0.401331829	1	0.0064855	2	Fads1
0.280627756	1	0.0064907	2	Irak3
0.440580649	1	0.006498	2	Pdia4
-0.258424331	1	0.0067237	2	Dhx9
-0.373109433	1	0.0067419	2	Stk16
0.450985205	1	0.0067608	2	Trib1
-0.467261913	1	0.0068422	2	Ube2z
0.28670093	1	0.0068838	2	Hprt
0.616200084	1	0.00693	2	Zfp467
-0.610587999	1	0.006973	2	Arl6ip5
-0.399823882	1	0.0070517	2	Ubqln2
-0.312268577	1	0.0070694	2	Tor1a
-0.295990193	1	0.0072184	2	Mrps16
-0.336989413	1	0.0072357	2	Gtf3c2
0.614972445	1	0.0072516	2	Strn4
-0.272603234	1	0.0072715	2	Ndufb10
-0.795733928	1	0.0074213	2	Kif21b
-0.281462092	1	0.0075426	2	Plk3
0.409864436	1	0.0076716	2	G3bp2
-0.308647019	1	0.0076813	2	Ash2l
-0.275874902	1	0.0076955	2	Erp44
-0.675430347	1	0.0078925	2	Rapgef1
0.417022258	1	0.0079192	2	Eml4
-0.576047345	1	0.0079277	2	Hspa13
0.282203172	1	0.0080033	2	Ap1s2
-0.273851871	1	0.0080224	2	Traf6
0.325721952	1	0.0080519	2	Leng8
-0.428502584	1	0.0080862	2	Fam129a
-0.270846757	1	0.0083102	2	Sepn1
-0.319736326	1	0.0083459	2	Zmiz1
-0.50497373	1	0.0084248	2	Rpl31
-0.271604546	1	0.0086222	2	Etv6
-0.396804833	1	0.0086266	2	Lpcat2
-0.376584348	1	0.0087005	2	Oxa1l
-0.342527348	1	0.0088488	2	Dcun1d5
0.392455962	1	0.0088769	2	Casp8
-0.286462743	1	0.0089203	2	Dock2
-0.308955233	1	0.008998	2	Acd
0.654456552	1	0.0090478	2	Tbc1d14
-0.409783149	1	0.0091401	2	Psmd3
-0.319529603	1	0.0091744	2	Cyld
-0.270024914	1	0.0091989	2	Cyc1
-0.368801241	1	0.0092217	2	Kif3a
0.281366335	1	0.0092478	2	Rtp4
0.580404251	1	0.0093138	2	Abcc1
0.299685011	1	0.0093952	2	Rsrp1
-0.25865773	1	0.0093964	2	Strap
0.4247052	1	0.009468	2	Ptpra
0.610780727	1	0.0096951	2	Tmem214
-0.26386382	1	0.009804	2	Ythdf2
0.268623712	1	0.0098272	2	Hmox2
-0.788186675	2.62E-23	1.97E-27	3	Ly6e

0.752622516	1.29E-15	9.66E-20	3	Cd72
-0.750696883	7.37E-14	5.52E-18	3	Ifitm3
-0.933922958	1.16E-13	8.70E-18	3	Ccl9
0.516649481	1.31E-13	9.79E-18	3	Hexb
-1.277773409	1.56E-13	1.17E-17	3	Mrc1
0.510564986	4.08E-13	3.06E-17	3	Lpl
-0.806415806	1.03E-11	7.73E-16	3	Ccl6
0.383011122	1.10E-11	8.26E-16	3	Mpeg1
0.567928571	2.06E-11	1.54E-15	3	Tgfbr1
0.560010433	1.17E-10	8.75E-15	3	Cd9
0.401189832	5.52E-10	4.14E-14	3	Cxcl16
-1.194269686	3.78E-09	2.83E-13	3	Pf4
0.642214054	7.13E-09	5.34E-13	3	Cpd
0.296228517	1.11E-08	8.28E-13	3	Lgals3
-1.635550152	1.19E-08	8.89E-13	3	F13a1
0.734465501	3.15E-08	2.36E-12	3	Syne1
0.494035126	5.86E-08	4.39E-12	3	Gusb
-1.537533496	6.12E-08	4.58E-12	3	Ccl8
-0.53194022	8.61E-08	6.45E-12	3	H2-Ab1
0.796750036	8.78E-08	6.58E-12	3	Syt11
-1.555428345	9.13E-08	6.84E-12	3	Cbr2
0.821505545	9.69E-08	7.26E-12	3	Pdgfb
0.369687336	1.50E-07	1.12E-11	3	Clic4
-1.413929145	1.63E-07	1.22E-11	3	Ccl5
0.392199858	2.78E-07	2.08E-11	3	Ms4a7
0.686439576	2.94E-07	2.20E-11	3	2210408F21Rik
0.39156518	3.16E-07	2.37E-11	3	Trem2
-0.739171897	3.48E-07	2.61E-11	3	Ifi27l2a
0.697632894	5.70E-07	4.27E-11	3	Fam102b
-0.602680686	7.70E-07	5.77E-11	3	H2-Eb1
0.254019239	9.25E-07	6.93E-11	3	Iqgap1
0.40811956	9.37E-07	7.02E-11	3	Slamf9
-0.489108831	1.04E-06	7.80E-11	3	H2-Aa
0.464795472	1.17E-06	8.76E-11	3	Ckb
0.44340605	1.42E-06	1.06E-10	3	Abcg1
0.555051559	1.91E-06	1.43E-10	3	Dpep2
-1.037180368	2.61E-06	1.95E-10	3	Cfh
0.28143296	2.69E-06	2.01E-10	3	Anxa5
1.082915775	3.81E-06	2.85E-10	3	Pcp4l1
0.647839001	6.28E-06	4.71E-10	3	Nes
0.360605291	7.44E-06	5.57E-10	3	Gatm
0.411588859	1.00E-05	7.49E-10	3	Slamf7
-0.726693574	1.01E-05	7.59E-10	3	H2-DMa
0.730689632	1.20E-05	8.96E-10	3	Cdk18
0.515648294	1.26E-05	9.47E-10	3	Epb4.1l2
1.020020537	1.44E-05	1.08E-09	3	Serpine1
0.469979419	1.53E-05	1.15E-09	3	Glipr1
0.394390535	1.66E-05	1.25E-09	3	Gng5
0.336510723	1.88E-05	1.41E-09	3	Lsp1
0.607744242	2.22E-05	1.66E-09	3	Zmynd15
0.566325698	2.28E-05	1.71E-09	3	Atp5sl
0.592313775	4.34E-05	3.25E-09	3	Flot2
0.581333666	4.95E-05	3.70E-09	3	Atp8b4
0.348459706	5.24E-05	3.92E-09	3	Capg
0.714120096	5.81E-05	4.35E-09	3	Tnfsf13b
0.602290889	6.75E-05	5.05E-09	3	Ptk2b
0.618390427	8.13E-05	6.09E-09	3	Pdlim7
0.76819815	9.01E-05	6.75E-09	3	Cadm1
0.324709703	0.000107196	8.03E-09	3	Tkt
-0.986713076	0.000120233	9.00E-09	3	Ccl7
0.555326901	0.000127272	9.53E-09	3	Abcc3

0.38923441	0.000129971	9.73E-09	3	Lipa
-0.40774171	0.000155894	1.17E-08	3	Cd74
0.412568	0.000164469	1.23E-08	3	Cd84
-0.809013366	0.000179577	1.34E-08	3	Vps37b
0.82072286	0.000193033	1.45E-08	3	Adcy3
0.416670666	0.000196998	1.48E-08	3	Rasgef1b
0.255441765	0.000220828	1.65E-08	3	Itgb1
0.347215916	0.000252766	1.89E-08	3	5730416F02Rik
-1.251524914	0.000273238	2.05E-08	3	Mgl2
0.562144609	0.000308628	2.31E-08	3	Spryd7
-1.623488131	0.000367822	2.75E-08	3	C4b
0.570377835	0.00043636	3.27E-08	3	Kcnn4
0.577899641	0.000484317	3.63E-08	3	Slc7a7
-1.028019988	0.000500736	3.75E-08	3	Folr2
0.622463788	0.000543951	4.07E-08	3	Ncapg2
0.487867462	0.000736268	5.51E-08	3	2010111I01Rik
0.55670523	0.000888971	6.66E-08	3	Ptchd1
0.294369042	0.001041681	7.80E-08	3	Gngt2
0.513697397	0.001215259	9.10E-08	3	Spn
-1.032399612	0.001435702	1.08E-07	3	Sema4a
0.44731359	0.00148721	1.11E-07	3	Gpr137b
0.472222035	0.001515235	1.13E-07	3	Apobr
0.522503349	0.001944483	1.46E-07	3	Dnase1l1
0.535108237	0.002370086	1.78E-07	3	Prkaca
-0.588472847	0.002407824	1.80E-07	3	Ccl2
0.372995954	0.002477882	1.86E-07	3	Igf1
-0.851774976	0.00261284	1.96E-07	3	Tmem176a
0.464567809	0.002863208	2.14E-07	3	Lat2
-0.582683341	0.002913656	2.18E-07	3	Cfp
0.739498286	0.003135432	2.35E-07	3	Slmap
0.635592505	0.003192103	2.39E-07	3	Cx3cr1
-1.019474027	0.003693322	2.77E-07	3	Ccnd2
0.296179155	0.004060756	3.04E-07	3	Aprt
0.576187008	0.004232244	3.17E-07	3	Itgax
-0.933179255	0.004369637	3.27E-07	3	Gas6
0.497865237	0.004477666	3.35E-07	3	Fbxo32
0.48770149	0.005839196	4.37E-07	3	Tmcc3
0.556230616	0.005955736	4.46E-07	3	Senp2
-0.512197426	0.005984468	4.48E-07	3	Sepp1
-1.015558345	0.006892316	5.16E-07	3	C3
0.484926932	0.006982463	5.23E-07	3	Tmem189
0.317869312	0.007021579	5.26E-07	3	Tuba1c
0.857492521	0.007472454	5.60E-07	3	Vash1
0.601303331	0.007666162	5.74E-07	3	Slc6a8
-0.68608101	0.007670821	5.75E-07	3	Zcchc11
0.351629167	0.008169865	6.12E-07	3	Alcam
0.463504504	0.008607843	6.45E-07	3	Nceh1
0.368071021	0.008668803	6.49E-07	3	Furin
0.422192511	0.008978475	6.72E-07	3	Myo1e
0.45796751	0.00940157	7.04E-07	3	Stmn1
0.341007192	0.01077834	8.07E-07	3	Vdac2
0.265325773	0.01080269	8.09E-07	3	Fyb
0.602168814	0.013535358	1.01E-06	3	Tmem71
0.282927939	0.013857714	1.04E-06	3	Col4a3bp
0.478419959	0.014612096	1.09E-06	3	Stk38l
0.369282306	0.014783997	1.11E-06	3	Axl
-1.147988732	0.014804984	1.11E-06	3	Rnasel
0.325224229	0.014880214	1.11E-06	3	Flna
0.483469607	0.015401718	1.15E-06	3	Dlat
0.261673475	0.016306772	1.22E-06	3	Abhd12
0.81868486	0.018588701	1.39E-06	3	Speg

0.286543779	0.018726792	1.40E-06	3	Gsn
0.56819218	0.019493932	1.46E-06	3	Mdfic
-1.257539884	0.020175357	1.51E-06	3	Cp
0.45938483	0.020666817	1.55E-06	3	Tars
-0.829279355	0.02116184	1.58E-06	3	Maf
0.478830579	0.022297843	1.67E-06	3	Apbb2
0.42487726	0.025565824	1.91E-06	3	Stx3
-0.610641624	0.025848819	1.94E-06	3	Ms4a6b
0.46700331	0.026266786	1.97E-06	3	Asph
0.767878515	0.027104592	2.03E-06	3	Slc35e4
0.392012666	0.027501419	2.06E-06	3	Itga6
0.387883728	0.027579661	2.07E-06	3	Fbxo3
0.366818959	0.031420005	2.35E-06	3	Mmp12
0.355817368	0.032367964	2.42E-06	3	Nucb1
-0.585325807	0.032657457	2.45E-06	3	Slc9a9
0.396654831	0.035151405	2.63E-06	3	Gpr137b-ps
0.28464376	0.037002883	2.77E-06	3	Pld3
0.700117796	0.037269512	2.79E-06	3	Igf2r
0.490172781	0.039845737	2.98E-06	3	Tmem237
-1.728786255	0.040237452	3.01E-06	3	Lyve1
0.274457051	0.040469556	3.03E-06	3	Reep5
-0.48412812	0.041777397	3.13E-06	3	Ccr2
0.415489104	0.042939217	3.22E-06	3	Zfand2a
-1.53714824	0.045099985	3.38E-06	3	Tbc1d4
0.299516379	0.045760073	3.43E-06	3	Lmna
0.361244747	0.046153638	3.46E-06	3	Ndufb4
-1.177623193	0.047037482	3.52E-06	3	Fgfr1
0.393978195	0.048301626	3.62E-06	3	Myof
0.353017424	0.048622215	3.64E-06	3	Trp53inp2
0.369954624	0.050308429	3.77E-06	3	Mapk3
0.716361591	0.050903922	3.81E-06	3	Zfand2b
0.382202617	0.05233217	3.92E-06	3	Cope
-1.349422402	0.055531928	4.16E-06	3	H2-Oa
0.762107511	0.060403847	4.52E-06	3	D8Ertd82e
0.331852097	0.068466477	5.13E-06	3	Itgb5
0.656120788	0.069564402	5.21E-06	3	Dtx4
0.437146027	0.070264209	5.26E-06	3	Olfml3
0.48132071	0.071392523	5.35E-06	3	Pik3cb
0.360603393	0.07217737	5.41E-06	3	Kcmf1
0.616157819	0.07227185	5.41E-06	3	Hivep3
-1.275239848	0.072662301	5.44E-06	3	Clec10a
-0.750074484	0.07425419	5.56E-06	3	Itsn1
0.449626273	0.074275112	5.56E-06	3	Creb5
-0.441989019	0.078249794	5.86E-06	3	Ifitm2
-0.65038721	0.081194325	6.08E-06	3	Sidt2
0.650259583	0.085282505	6.39E-06	3	Mmgt1
0.269688272	0.086030996	6.44E-06	3	Uqcrb
0.688724389	0.091103709	6.82E-06	3	Mmgt2
0.469925951	0.094901358	7.11E-06	3	Slc37a2
0.538743218	0.095758539	7.17E-06	3	Tle1
-1.206517389	0.100595114	7.53E-06	3	Ly6a
0.589100086	0.101787868	7.62E-06	3	Lxn
0.543361754	0.112927285	8.46E-06	3	Map7d1
0.526115317	0.112978274	8.46E-06	3	Arhgap22
0.452940247	0.116738478	8.74E-06	3	Tes
0.555021614	0.133121043	9.97E-06	3	AI427809
-0.787291367	0.133236213	9.98E-06	3	Marcksl1
-1.357685141	0.14072466	1.05E-05	3	Il1r2
0.680312387	0.151316692	1.13E-05	3	Tpx2
-1.246854228	0.153002002	1.15E-05	3	Ltc4s
0.561368828	0.157703068	1.18E-05	3	Vopp1

0.282142433	0.168520627	1.26E-05	3	Fam134b
0.330859371	0.169140936	1.27E-05	3	Bcl2l1
0.268376356	0.170408417	1.28E-05	3	Tspo
0.680225676	0.17265888	1.29E-05	3	2510009E07Rik
-0.631628812	0.173122492	1.30E-05	3	H2-DMb1
-0.934519523	0.175100624	1.31E-05	3	Cbfa2t3
-0.686267993	0.182801798	1.37E-05	3	Clec4n
0.399164107	0.187239916	1.40E-05	3	Cap1
0.602061823	0.187943772	1.41E-05	3	Stap1
0.550599068	0.188763717	1.41E-05	3	Daglb
0.275887861	0.198890023	1.49E-05	3	Plekhm3
0.618283605	0.21189253	1.59E-05	3	Arap3
-0.809355037	0.213574403	1.60E-05	3	Txnip
0.382331196	0.215389176	1.61E-05	3	Iscu
0.340627096	0.222812252	1.67E-05	3	Rnf111
0.315243727	0.229844939	1.72E-05	3	Vdac1
0.338893551	0.235150796	1.76E-05	3	2410089E03Rik
0.370277989	0.236582673	1.77E-05	3	Pparg
0.349143793	0.24825633	1.86E-05	3	Slc15a3
0.383921474	0.265099728	1.99E-05	3	Zfp385a
0.432017517	0.267710426	2.01E-05	3	Pilra
0.331935795	0.286913264	2.15E-05	3	Adam17
0.431369441	0.291038434	2.18E-05	3	AA467197
0.364133241	0.316029802	2.37E-05	3	Dda1
-1.210775721	0.328474946	2.46E-05	3	Rcn3
-0.292587129	0.329291405	2.47E-05	3	Itgal
-1.593861399	0.346704639	2.60E-05	3	Cd163
-0.852124589	0.348180906	2.61E-05	3	Fam46a
0.481300475	0.361234443	2.71E-05	3	Tmem109
-0.678760082	0.376263479	2.82E-05	3	P2ry10
0.32213421	0.379038125	2.84E-05	3	Apobec1
-1.077642292	0.380952395	2.85E-05	3	Nr4a2
0.371923452	0.392613432	2.94E-05	3	Ciz1
0.549775374	0.406195405	3.04E-05	3	Trio
0.401977567	0.445368186	3.34E-05	3	Slc39a10
-0.407284837	0.445468881	3.34E-05	3	Fcgr2b
0.613724274	0.466933611	3.50E-05	3	Synj2
0.326167168	0.497960545	3.73E-05	3	Csk
-0.939064765	0.501857827	3.76E-05	3	Ccr1
-0.930460962	0.506006229	3.79E-05	3	Bank1
0.30984823	0.517230978	3.87E-05	3	Bcl2l11
-0.492701454	0.553685872	4.15E-05	3	Il1b
0.596996511	0.556467778	4.17E-05	3	Manba
0.766148691	0.566174818	4.24E-05	3	Rai14
0.49353684	0.604921868	4.53E-05	3	Ercc6
0.407663216	0.63285859	4.74E-05	3	Soga1
0.281756494	0.639740494	4.79E-05	3	Hypk
0.363411017	0.643811406	4.82E-05	3	Ap3s2
-0.719777538	0.676672243	5.07E-05	3	Plbd1
-0.891439105	0.689571843	5.16E-05	3	Igfbp4
0.556132388	0.708108999	5.30E-05	3	L1cam
0.263488967	0.741902731	5.56E-05	3	Aldoa
0.556150845	0.801503445	6.00E-05	3	Kif13b
-0.544293225	0.827339208	6.20E-05	3	Btg2
-0.932112642	0.841089036	6.30E-05	3	Fcgrt
0.558429727	0.854892303	6.40E-05	3	Rusc2
0.477867575	0.873501592	6.54E-05	3	Map4k3
-0.615286484	0.875490403	6.56E-05	3	Egr1
-1.313638077	0.885548998	6.63E-05	3	Cmah
0.353482589	0.89088118	6.67E-05	3	Mrpl13
-0.283685539	0.929874315	6.96E-05	3	Klf2

0.382119672	0.941510919	7.05E-05	3	Prkcsh
0.647935514	1	8.10E-05	3	Angptl2
0.275763846	1	8.32E-05	3	Gdf15
0.306616362	1	8.34E-05	3	Sap30
0.617611527	1	8.53E-05	3	Tmem119
-0.483584919	1	8.86E-05	3	H2-T23
0.250423829	1	9.15E-05	3	Sema4d
0.269459895	1	9.15E-05	3	Ran
0.293596228	1	9.30E-05	3	Mmp14
-0.672983227	1	9.33E-05	3	Aoah
0.309303394	1	9.62E-05	3	Ywhag
0.324910224	1	9.74E-05	3	Naa16
0.302346142	1	9.80E-05	3	Ivns1abp
-0.731393989	1	0.0001	3	Slc15a2
0.314917619	1	0.000101	3	Scarb2
0.505935508	1	0.0001052	3	Fhl3
0.330372047	1	0.0001098	3	Lyn
0.497959634	1	0.0001116	3	Otulin
0.322981847	1	0.000113	3	Hcfc1r1
0.317243412	1	0.0001147	3	Rab20
0.593435971	1	0.0001196	3	Klc4
-0.453361977	1	0.0001234	3	Ier3
0.410986339	1	0.0001258	3	Srxn1
0.459953525	1	0.000127	3	Scoc
0.39486629	1	0.000131	3	Mrpl51
0.780490769	1	0.0001373	3	4931406P16Rik
0.27713998	1	0.0001378	3	Pitpnc1
0.311464448	1	0.0001388	3	Mlf2
-0.668514512	1	0.0001406	3	Gda
-0.340620309	1	0.0001416	3	Serinc3
0.271365202	1	0.0001424	3	Tec
0.257942725	1	0.0001425	3	Psme3
0.252543826	1	0.0001432	3	Spcs1
0.540730511	1	0.0001439	3	Pdgfa
0.345138706	1	0.000144	3	4833439L19Rik
-1.593489845	1	0.0001455	3	Ccr7
0.277381749	1	0.0001456	3	Lair1
0.684618851	1	0.0001461	3	Gyk
0.523388817	1	0.0001467	3	Xylt2
0.541282736	1	0.0001535	3	Col14a1
0.330468228	1	0.000154	3	Lrpprc
0.305220541	1	0.0001552	3	Ppt2
-1.630533922	1	0.0001562	3	Nkg7
0.271601834	1	0.00016	3	Erbb2ip
0.277609494	1	0.0001727	3	Anpep
-0.657909649	1	0.0001764	3	Tsc22d3
0.315192621	1	0.000185	3	Rhoc
0.337948981	1	0.0001854	3	Acsl4
-0.347256596	1	0.0001878	3	Cd36
-0.651089682	1	0.0001965	3	Stat4
0.860014575	1	0.0001995	3	Nelfcd
-1.472395617	1	0.0002027	3	Ccl24
0.345475176	1	0.0002175	3	Tor1aip1
0.357978966	1	0.000228	3	Prdx4
-0.618184142	1	0.0002302	3	Dguok
0.304946182	1	0.0002311	3	Ggh
0.258809445	1	0.0002425	3	Capns1
0.275176653	1	0.0002442	3	Hsd17b11
0.345287224	1	0.0002482	3	Tns3
-1.379788476	1	0.0002601	3	Ednrb
0.571749059	1	0.0002632	3	Abi3

0.271340612	1	0.0002652	3	Uap1l1
0.383504371	1	0.0002666	3	Gas2l3
0.25836232	1	0.0002674	3	Lpxn
-0.524357659	1	0.000268	3	Ubn1
0.314256971	1	0.0002688	3	Pmepa1
-0.538886119	1	0.0002695	3	Emb
-0.502645358	1	0.000273	3	Irf7
0.449275525	1	0.0002809	3	Dbndd2
0.43306726	1	0.0002836	3	Msrb2
0.416930659	1	0.000286	3	Ilkap
0.335301778	1	0.0003151	3	Actn1
0.540381823	1	0.0003157	3	Ppp2r1b
0.341363712	1	0.000318	3	Camk2d
0.853613033	1	0.0003263	3	Cenpe
0.252269864	1	0.0003307	3	Med11
0.407116383	1	0.0003326	3	Milr1
0.276567451	1	0.0003352	3	Aga
0.616495338	1	0.0003386	3	Shmt2
0.307113065	1	0.0003414	3	Ank
-1.129285685	1	0.0003438	3	Plac8
-0.962515788	1	0.0003496	3	Hspa1b
0.488129569	1	0.0003519	3	Mpp6
0.879760943	1	0.0003667	3	Trim36
0.250017995	1	0.000372	3	Spg7
-0.491529702	1	0.0003792	3	Klf4
0.312318283	1	0.0003816	3	Aldh9a1
0.58827074	1	0.0003889	3	Ermp1
-0.426424214	1	0.0003932	3	Rsbn1
0.354690837	1	0.0003949	3	Eif3g
-0.661974181	1	0.0003953	3	P2ry12
0.895579432	1	0.0003975	3	Prc1
0.379242774	1	0.0004084	3	Fam86
0.312942972	1	0.0004265	3	Tsg101
-0.595778617	1	0.0004383	3	Wdr41
0.28959425	1	0.0004436	3	Mdm2
0.297833604	1	0.0004487	3	Hpgds
0.482174356	1	0.0004495	3	2510003E04Rik
-0.606840052	1	0.0004683	3	Prpf39
-0.689340387	1	0.0004695	3	Mthfr
0.486143665	1	0.0004754	3	Psmg2
-0.668379113	1	0.0004897	3	Tns1
0.273117989	1	0.0005123	3	Anxa3
-0.561527953	1	0.0005183	3	Trps1
0.256471096	1	0.000527	3	Cpeb2
-0.657059849	1	0.0005285	3	Ago1
0.300808502	1	0.0005349	3	Hp1bp3
-1.443245581	1	0.000542	3	F10
0.407836785	1	0.0005467	3	Cox20
0.40604849	1	0.0005646	3	Extl3
0.277314702	1	0.0005705	3	Hadh
-0.415540985	1	0.0005708	3	Jun
-1.116906588	1	0.0005711	3	Fcna
0.480568291	1	0.0005785	3	Pkp4
-0.371618191	1	0.0005858	3	Fam117b
0.337985509	1	0.0005956	3	Exoc2
0.370351715	1	0.0006034	3	Arap1
0.444943098	1	0.0006352	3	Bmyc
0.432965852	1	0.0006534	3	Exoc6b
0.41191883	1	0.000654	3	Xpo1
0.362551267	1	0.0006557	3	Coro2a
0.478705166	1	0.000658	3	Clk4

-0.662133505	1	0.0006632	3	Ccl12
-0.527944707	1	0.0006831	3	Cdk11b
0.437776154	1	0.0006834	3	Mvb12b
0.265863972	1	0.0006892	3	Ppp1r18
0.36819791	1	0.0006957	3	Yme1l1
-0.797983358	1	0.0007083	3	Dennd1c
-0.453035667	1	0.0007272	3	Pfkfb3
0.287059926	1	0.0007282	3	Galnt7
-0.895940045	1	0.0007312	3	Gimap6
0.41324397	1	0.0007405	3	G6pdx
0.30088553	1	0.0007526	3	Park7
-1.180809438	1	0.0007533	3	Apoe
0.459551942	1	0.0007706	3	Acp1
0.398383459	1	0.0007864	3	Spen
-0.530921376	1	0.0007927	3	Dgat1
0.368919933	1	0.000797	3	Pon2
0.520522265	1	0.0008106	3	Tpm1
-1.175275567	1	0.0008164	3	Fxyd2
0.366106214	1	0.0008206	3	Npc1
0.505441589	1	0.0008233	3	Zranb1
0.252895454	1	0.000844	3	Tspan31
0.276800996	1	0.0008529	3	Cdip1
0.649322297	1	0.0008623	3	Ifnar1
0.330726962	1	0.0008714	3	Rnf6
0.550412209	1	0.0008737	3	Bcap29
-0.655429477	1	0.0008808	3	Ap2a2
0.420164605	1	0.0008955	3	F11r
0.458713558	1	0.0008977	3	Tmem55a
0.335216322	1	0.0009272	3	Cd300lb
0.49544241	1	0.0009424	3	Tpgs1
0.264639727	1	0.0009503	3	Psma6
0.447004148	1	0.0009846	3	Tnfaip1
0.379717454	1	0.0009919	3	Atxn1
-0.711708955	1	0.000998	3	Cysltr1
-0.489251462	1	0.0010284	3	Atxn7
0.408674312	1	0.0010436	3	Pdlim4
-0.812900538	1	0.0010532	3	Rcsd1
-1.074732321	1	0.0010696	3	Oas2
0.402543325	1	0.0010831	3	Tnip1
0.360560529	1	0.0011141	3	Fibp
-0.455310407	1	0.0011243	3	Crem
0.294528008	1	0.0011331	3	Slc25a39
0.298464993	1	0.00117	3	Tmbim4
0.330735185	1	0.0011842	3	Spsb2
0.685008535	1	0.0011891	3	Nedd4l
-0.503081205	1	0.0011931	3	Tmem173
0.450785311	1	0.0011985	3	Znrf1
0.33933269	1	0.0012005	3	Eif2a
0.456038264	1	0.0012162	3	Wdr74
0.321624372	1	0.0012171	3	Ppm1g
0.372820661	1	0.0012293	3	Dhrs1
0.362762782	1	0.0012302	3	Cdk14
0.358650332	1	0.0012336	3	Ebi3
0.350391821	1	0.0012464	3	Slc2a1
-0.556119108	1	0.0012644	3	Mndal
0.385826955	1	0.0012775	3	Rffl
0.304064575	1	0.0012828	3	Klhdc10
0.400621423	1	0.0012995	3	Pik3ca
0.381354315	1	0.0013243	3	Itgav
0.351698316	1	0.001361	3	Abcd1
0.359263953	1	0.0013803	3	Calu

-0.526752268	1	0.0014063	3	Zfp445
0.66646679	1	0.0014157	3	Mki67
-0.418506757	1	0.0014187	3	Slfn5
-0.86067023	1	0.0014551	3	Ahr
0.281666558	1	0.0014608	3	Csnk1e
-0.855330709	1	0.0014633	3	Agtrap
0.36653908	1	0.001484	3	Hmgn5
0.331572271	1	0.0015012	3	Zbtb21
0.352116736	1	0.0015039	3	Pisd
0.369896049	1	0.0015689	3	Brcc3
-0.295036266	1	0.0015808	3	Jund
-0.749404355	1	0.0015848	3	Gm1966
-0.77301874	1	0.0016052	3	Morc3
0.600413623	1	0.0016371	3	Phb
0.276421599	1	0.0016387	3	Adam8
0.410882859	1	0.0016425	3	Frrs1
0.547259562	1	0.0016507	3	Mak16
0.277869276	1	0.0016532	3	Gnl2
-0.29071773	1	0.0016557	3	Srrm2
0.328447573	1	0.0016574	3	Wdr83os
0.317628987	1	0.0017275	3	Cgrrf1
0.273624726	1	0.0017749	3	Rnf5
0.302057956	1	0.0017957	3	Pfkp
-0.695603529	1	0.0018494	3	Hilpda
0.271346752	1	0.0018613	3	Vps28
-0.49387628	1	0.001868	3	Clec7a
0.324652347	1	0.0018728	3	Prnp
-0.48973289	1	0.0019087	3	Bbip1
0.456871923	1	0.0019192	3	Pdcd10
0.431691135	1	0.0019581	3	Fbxw7
-0.604399133	1	0.0019679	3	Alox5
0.424524417	1	0.0019937	3	Wdr11
0.266915241	1	0.0020127	3	Dpysl2
0.267079049	1	0.0020158	3	Scp2
-0.441812807	1	0.0020189	3	Il4ra
0.561475113	1	0.0020196	3	Fgd4
0.297474795	1	0.002062	3	Snx17
0.251222197	1	0.0020818	3	Fndc3b
-0.64165411	1	0.0020818	3	Lbr
-0.27839807	1	0.0020933	3	Igsf6
-0.712097616	1	0.0021151	3	Cd33
0.473134839	1	0.0021222	3	Ppp2r5e
0.264795003	1	0.0021628	3	Map3k2
-0.279123237	1	0.0021635	3	Chd7
-0.272675881	1	0.0021956	3	Srgn
-0.608786356	1	0.0022768	3	Hpgd
0.440520482	1	0.0022821	3	Vps18
-0.800776267	1	0.0023081	3	Dok2
-0.501796201	1	0.0023168	3	Top2b
0.337887998	1	0.0023176	3	Fbxo11
0.343642084	1	0.0023631	3	Slain2
0.316762796	1	0.0023868	3	Eif2b2
0.545627546	1	0.0024246	3	Cd180
0.254852215	1	0.002428	3	Kansl1
0.284190418	1	0.0024446	3	Gart
-0.396935195	1	0.0025016	3	H2afj
-0.521638988	1	0.0025325	3	Zc3hav1
-0.658273931	1	0.0025427	3	Ier2
0.587076988	1	0.0025612	3	Top2a
0.597892014	1	0.0026234	3	Vps16
-0.409569576	1	0.0026618	3	Zcchc6

-0.728992919	1	0.0026881	3	Arsb
0.373623868	1	0.0027028	3	Tbc1d9b
-0.576137124	1	0.0027079	3	Cd24a
0.415954806	1	0.0027302	3	Sntb2
0.276489446	1	0.0027351	3	Ostm1
0.41124648	1	0.0027369	3	Tpcn1
0.332418003	1	0.0027458	3	Plec
-0.57237035	1	0.002755	3	Snhg8
0.74234908	1	0.0028768	3	Kat7
0.288020685	1	0.0029195	3	Dnmt3a
0.478051546	1	0.0029338	3	Lrp12
-0.265135066	1	0.0029453	3	Pnrc1
0.409936555	1	0.0029661	3	Akap8l
0.366243271	1	0.0030516	3	Farsb
0.720611599	1	0.0030994	3	BC005624
0.406673207	1	0.003186	3	Enoph1
0.265671775	1	0.0032072	3	Serf2
0.377169969	1	0.0032367	3	Htatsf1
-0.357764997	1	0.0033085	3	Gbp3
0.442136473	1	0.0033238	3	Myo9b
0.404877875	1	0.0034422	3	Lap3
0.457989448	1	0.0034681	3	Dynlt3
0.33775894	1	0.0035702	3	Topbp1
0.297956584	1	0.0036206	3	U2af1l4
0.315976152	1	0.0036374	3	Uba52
0.271733241	1	0.0036957	3	Cuedc2
-0.721122168	1	0.003706	3	AW112010
0.265447609	1	0.0037553	3	Hmg20b
0.291918938	1	0.0037569	3	Paip2
0.252930169	1	0.0038895	3	Rab1b
-0.304711231	1	0.0038925	3	Tspyl1
0.27602774	1	0.0038934	3	Dhps
-0.650543025	1	0.0038946	3	Gbp7
-0.365348621	1	0.0039047	3	Nktr
0.469462667	1	0.0039467	3	Mtx1
0.308099999	1	0.0040722	3	Med8
-0.438630242	1	0.0040786	3	Cnp
0.32747885	1	0.0040856	3	Hibadh
-0.393712721	1	0.004092	3	Abcf3
0.503821156	1	0.0041215	3	Ptgr1
0.338007186	1	0.0041237	3	Slc9a1
0.329899427	1	0.0041352	3	Cnot6
0.310305225	1	0.0041599	3	Dync1i2
0.319313851	1	0.0041867	3	Arid1b
0.710042883	1	0.0042278	3	Gdf3
0.332902105	1	0.0043177	3	Ccdc23
0.506628966	1	0.0043476	3	Thoc5
0.335662225	1	0.0043832	3	Atp8a1
-0.29047103	1	0.0044063	3	Gadd45gip1
0.346040821	1	0.0044714	3	Ramp1
0.250693324	1	0.0045163	3	Yipf4
-0.620497534	1	0.0045687	3	Ahsa2
-0.286279077	1	0.0045716	3	Snx2
0.36294712	1	0.0046047	3	Mar.05
-0.472244475	1	0.0046776	3	Batf3
-0.488608451	1	0.004699	3	St8sia4
0.287523308	1	0.0048208	3	S100a13
-0.456154975	1	0.0048405	3	Snrpa1
-0.347434802	1	0.0049703	3	Anxa6
-0.496244498	1	0.0049862	3	Gas7
0.282677394	1	0.0049958	3	Adam9

0.299322408	1	0.0050862	3	Lyst
0.36983742	1	0.0051096	3	Fmnl3
0.313806857	1	0.0051772	3	Ubap1
0.250786502	1	0.0052245	3	Emc7
-0.363794296	1	0.0052298	3	Map3k14
-0.39999679	1	0.0052937	3	Clec4a2
0.294801919	1	0.0056317	3	Ccni
-0.345991082	1	0.0056407	3	Cd97
0.280235118	1	0.0056508	3	Gab2
-0.577873289	1	0.0056694	3	Epsti1
-0.30748277	1	0.0056858	3	Tmem176b
-1.362999246	1	0.0058224	3	Ifitm1
-0.448026914	1	0.005831	3	Tmem141
0.296117817	1	0.005846	3	Ap3b1
-0.529813862	1	0.0058692	3	Pisd-ps1
-0.266887073	1	0.00599	3	Efr3a
-0.56374884	1	0.0060188	3	Samm50
0.327689594	1	0.0060937	3	Trim47
0.284144512	1	0.0060985	3	Arhgap17
-0.54821883	1	0.0061222	3	Oas1a
0.306509415	1	0.0061261	3	Sgcb
-0.503721938	1	0.0061913	3	Dusp7
0.611723237	1	0.0062069	3	Adap1
0.263783868	1	0.006246	3	Sirt7
-0.46404394	1	0.0062482	3	Malt1
-0.433336495	1	0.0063753	3	Atf7ip
0.439036787	1	0.0065844	3	Cops8
-0.789681011	1	0.0066073	3	Itgb7
0.271343533	1	0.0066219	3	Samd8
0.256050766	1	0.0066599	3	Hpcal1
-0.352034964	1	0.0070635	3	Tiparp
0.274232289	1	0.0070688	3	Paics
0.270433642	1	0.0071292	3	Gpatch11
0.278526486	1	0.0072156	3	Dapk1
0.310198885	1	0.0073209	3	Atp2b4
-0.474598929	1	0.0073232	3	Rgl1
0.260036824	1	0.0073716	3	Cttnbp2nl
-0.287563913	1	0.0073988	3	Kbtbd2
-0.325703959	1	0.0076355	3	Itga4
-0.297479055	1	0.0076606	3	Kctd12
0.664739422	1	0.0076606	3	Zufsp
0.402296693	1	0.0076797	3	Fam214b
0.277434003	1	0.0076943	3	Klhl6
0.342801653	1	0.0078693	3	Supt20
-0.821981546	1	0.0079117	3	Birc2
0.314912781	1	0.0079252	3	Thumpd3
-0.659046711	1	0.0079967	3	Stat2
-0.749136601	1	0.0080136	3	Lifr
0.296255129	1	0.008209	3	Fblim1
0.277563427	1	0.0082152	3	Prr3
0.395382548	1	0.0082712	3	Mrps26
-0.435188608	1	0.0083213	3	Aftph
0.489043825	1	0.0083537	3	Bap1
-0.383339559	1	0.0084435	3	Wrn
0.253199699	1	0.0084533	3	Atp6v1d
-0.604731371	1	0.008533	3	Rtp4
-0.382601485	1	0.0085999	3	Sde2
0.358170831	1	0.0087778	3	Mpv17
-1.058574471	1	0.0088709	3	Cd209a
0.409522347	1	0.0089325	3	Rhbdf2
0.288853757	1	0.0089713	3	Dhx30

-0.378742659	1	0.0089882	3	Sft2d2
0.310655295	1	0.0090334	3	Emc3
0.327186481	1	0.009118	3	Zmat2
-0.435920821	1	0.0091656	3	Sp140
-0.389706775	1	0.0092008	3	Slc12a7
0.300401421	1	0.0092143	3	Tmem206
0.356119675	1	0.0092151	3	Smarca4
0.34124951	1	0.0092823	3	Psen2
-0.294091841	1	0.0093074	3	Marcks
0.258594536	1	0.0093445	3	Pyurf
-0.699506794	1	0.0093992	3	Sorbs3
-0.644496344	1	0.0094933	3	Riok1
-0.485549209	1	0.0095852	3	Nfxl1
-0.492442667	1	0.0096615	3	Snhg12
0.271786216	1	0.0096751	3	Plekhm2
0.557441946	1	0.0098542	3	Pde8a
-0.343818859	1	0.009908	3	Ist1
0.258665334	1	0.0099458	3	Wdr91
1.561685081	3.97E-44	2.97E-48	4	L1cam
1.007600775	1.79E-43	1.34E-47	4	Disp1
1.185419396	6.02E-37	4.51E-41	4	Aasdhppt
1.461880036	4.95E-33	3.71E-37	4	Hsd11b1
0.84455724	5.90E-30	4.42E-34	4	Sccpdh
1.834560724	9.51E-30	7.12E-34	4	Atrip
1.480771761	2.72E-29	2.04E-33	4	Setmar
1.560568471	1.93E-28	1.44E-32	4	Kdm2b
0.86635699	2.97E-28	2.22E-32	4	Ttll1
1.042367105	3.53E-28	2.65E-32	4	Tbc1d2
1.188601142	4.59E-28	3.44E-32	4	Terf2ip
1.319559079	5.95E-27	4.46E-31	4	Zfp580
0.967031737	2.44E-25	1.82E-29	4	Enox2
0.785362131	5.66E-25	4.24E-29	4	Ldlrap1
1.054570887	9.03E-25	6.76E-29	4	Acsl1
1.024816176	6.04E-23	4.52E-27	4	Zbtb42
0.995013413	9.41E-23	7.05E-27	4	Creb3l2
1.247838326	1.00E-22	7.53E-27	4	Nudt14
0.776497206	1.10E-22	8.20E-27	4	Osbpl1a
1.76296599	1.38E-22	1.04E-26	4	Lrp8
1.057134795	1.78E-22	1.33E-26	4	Irak3
1.300746819	2.21E-22	1.66E-26	4	Lyrm1
0.95083067	2.50E-22	1.87E-26	4	2310061I04Rik
1.047105005	4.43E-22	3.32E-26	4	Snx20
1.294397215	5.12E-22	3.83E-26	4	Spn
0.777076397	1.18E-21	8.80E-26	4	Itpr3
1.457445781	3.41E-21	2.55E-25	4	Dnase1l3
0.847738706	1.22E-20	9.11E-25	4	Mrps18a
1.13777006	1.42E-20	1.07E-24	4	Nes
0.955696768	3.94E-20	2.95E-24	4	Tspan4
0.812828997	7.92E-20	5.93E-24	4	Pde8a
0.710831662	9.42E-20	7.06E-24	4	Ttc28
1.176266163	1.37E-19	1.03E-23	4	Med9
1.033957819	2.84E-19	2.13E-23	4	Idh3g
0.876826511	3.70E-19	2.77E-23	4	Cxcr3
0.579421464	4.42E-19	3.31E-23	4	Zfp74
1.151905779	6.82E-19	5.11E-23	4	Pdlim7
0.972723638	7.50E-19	5.62E-23	4	Tnip1
0.798520822	7.80E-19	5.84E-23	4	Gamt
1.271160125	1.48E-18	1.10E-22	4	Mkl2
0.832245313	1.78E-18	1.33E-22	4	Prkra
1.411930473	2.42E-18	1.82E-22	4	4931428F04Rik
0.780607828	3.26E-18	2.44E-22	4	Golga1

0.865678636	6.84E-18	5.12E-22	4	1600020E01Rik
1.07256112	9.09E-18	6.80E-22	4	Alg3
0.84183796	9.20E-18	6.89E-22	4	Ammecr1
1.846137507	1.10E-17	8.22E-22	4	Apoc2
0.625812192	1.80E-17	1.35E-21	4	Gemin4
0.783454975	3.95E-17	2.96E-21	4	Polr1a
1.459291186	4.93E-17	3.69E-21	4	Pkd2
0.852048003	5.14E-17	3.85E-21	4	Tomm70a
0.712745539	5.62E-17	4.21E-21	4	Cxcl1
0.454719676	5.89E-17	4.41E-21	4	Rrm2b
0.904363311	6.24E-17	4.67E-21	4	Nenf
0.759819746	9.99E-17	7.48E-21	4	Cd72
0.68193518	1.21E-16	9.08E-21	4	Cep170b
1.123011424	1.58E-16	1.18E-20	4	Gm5547
0.772946715	1.70E-16	1.28E-20	4	Plekha1
0.776600895	2.03E-16	1.52E-20	4	Gcc2
1.034205576	2.05E-16	1.54E-20	4	Myo1e
-1.045112379	2.67E-16	2.00E-20	4	Gm1821
1.076779082	2.72E-16	2.04E-20	4	Ptchd1
1.172020017	2.81E-16	2.11E-20	4	Ppp1r13l
0.68640999	2.84E-16	2.12E-20	4	Polr3a
1.056404797	3.21E-16	2.40E-20	4	Tmem159
0.68205099	3.38E-16	2.53E-20	4	2810002D19Rik
0.804264328	3.45E-16	2.59E-20	4	Dhrs4
1.460754409	3.89E-16	2.91E-20	4	Smox
-0.760963512	5.01E-16	3.75E-20	4	Pfn1
1.081140362	5.97E-16	4.47E-20	4	Snx9
0.951344503	6.24E-16	4.68E-20	4	Gdpd1
1.718862111	7.88E-16	5.90E-20	4	Bik
1.032211108	8.66E-16	6.49E-20	4	Fadd
0.780262045	9.03E-16	6.76E-20	4	Mrps24
0.839058353	1.19E-15	8.90E-20	4	Ndrg1
0.703862908	1.27E-15	9.53E-20	4	Qdpr
1.158348757	1.31E-15	9.78E-20	4	Angptl2
1.393494842	1.60E-15	1.20E-19	4	Atg9a
0.804206096	1.76E-15	1.32E-19	4	Atg2b
0.6076209	1.87E-15	1.40E-19	4	Suox
1.011772032	1.94E-15	1.45E-19	4	Atp2b4
0.76424665	2.09E-15	1.56E-19	4	Ptafr
1.014832292	2.12E-15	1.59E-19	4	Htr2b
1.122017275	2.15E-15	1.61E-19	4	Apbb2
1.417387099	2.68E-15	2.01E-19	4	Cep97
0.769000421	3.14E-15	2.35E-19	4	Glipr1
1.044637463	3.52E-15	2.64E-19	4	Slc39a11
0.740854708	4.13E-15	3.10E-19	4	Sec24b
0.48071925	4.16E-15	3.12E-19	4	Clec4b1
0.790448957	4.19E-15	3.14E-19	4	Itgax
0.924238473	5.01E-15	3.75E-19	4	Pdlim2
0.539198928	5.10E-15	3.82E-19	4	Pot1a
0.821372199	5.33E-15	3.99E-19	4	Clec4d
0.614366887	5.95E-15	4.45E-19	4	Ltn1
0.440667969	6.04E-15	4.52E-19	4	Taf5l
0.830437867	7.95E-15	5.95E-19	4	Cdr2
0.939354776	8.56E-15	6.41E-19	4	Phospho2
0.889024781	9.02E-15	6.75E-19	4	P2ry10
0.683583952	9.38E-15	7.03E-19	4	Papd7
0.715821518	1.62E-14	1.21E-18	4	Kansl3
0.668090152	1.72E-14	1.29E-18	4	Fbxl17
-0.816516946	1.72E-14	1.29E-18	4	Rps26
0.927428705	1.75E-14	1.31E-18	4	Lactb2
0.647505017	1.91E-14	1.43E-18	4	D11Wsu47e

0.881711304	2.33E-14	1.74E-18	4	Mrpl45
0.549576414	2.92E-14	2.18E-18	4	Map4k3
1.440702124	2.93E-14	2.20E-18	4	Gpr84
0.901598323	3.15E-14	2.36E-18	4	Pyurf
0.999754437	3.80E-14	2.84E-18	4	Prss30
0.613550013	5.06E-14	3.79E-18	4	Hivep1
0.567079291	5.42E-14	4.06E-18	4	Stmn4
0.902919071	6.42E-14	4.81E-18	4	Fcrl1
0.781407777	6.97E-14	5.22E-18	4	Spsb2
0.844784765	7.22E-14	5.41E-18	4	Fmn1
0.643927937	7.75E-14	5.81E-18	4	Pqlc3
0.738187224	1.07E-13	8.01E-18	4	Sdccag8
0.757596105	1.07E-13	8.02E-18	4	Mrps35
0.690532275	1.11E-13	8.29E-18	4	Mrps23
0.744721484	1.21E-13	9.05E-18	4	Zfand2a
1.26262354	1.24E-13	9.32E-18	4	Ddx56
-0.845256436	1.34E-13	1.00E-17	4	Mir6236
0.900666499	1.41E-13	1.05E-17	4	Farsa
1.207264925	1.41E-13	1.06E-17	4	Btd
0.591642713	1.75E-13	1.31E-17	4	Gtf2f2
0.737194407	1.84E-13	1.38E-17	4	Ubr2
0.589535794	1.96E-13	1.47E-17	4	Ighmbp2
0.57057105	2.18E-13	1.63E-17	4	Mir22hg
0.626594906	2.26E-13	1.69E-17	4	Slc9a6
0.64637047	2.34E-13	1.75E-17	4	Get4
0.824894322	2.63E-13	1.97E-17	4	Ppp2r5a
0.592208222	2.69E-13	2.01E-17	4	Leng1
0.661833772	2.74E-13	2.05E-17	4	Ccdc66
0.88601726	2.90E-13	2.17E-17	4	Pglyrp1
1.198576632	2.94E-13	2.20E-17	4	Zdhhc16
0.299550244	3.22E-13	2.41E-17	4	H2-Ke6
0.704840359	3.70E-13	2.77E-17	4	Fgd3
1.044476183	3.87E-13	2.90E-17	4	Fam98a
0.330041015	5.18E-13	3.88E-17	4	Vgll4
0.762785936	5.33E-13	3.99E-17	4	Tgm2
1.044143352	6.07E-13	4.55E-17	4	Gcsh
1.324691039	6.19E-13	4.64E-17	4	Ccdc15
0.68651192	6.23E-13	4.67E-17	4	Ndufb10
0.851653866	7.26E-13	5.43E-17	4	Dctpp1
0.755929438	7.56E-13	5.66E-17	4	Fam110a
1.219130454	7.68E-13	5.75E-17	4	Mrm1
0.690719781	7.93E-13	5.94E-17	4	Tfpt
0.797594753	8.26E-13	6.18E-17	4	Htra2
0.681286831	8.75E-13	6.55E-17	4	Vash1
0.690262101	8.93E-13	6.69E-17	4	Gpr137b
0.890430186	1.06E-12	7.97E-17	4	Kcnn4
0.69728928	1.07E-12	7.99E-17	4	Mphosph10
0.297767874	1.15E-12	8.59E-17	4	Ppcs
0.898390863	1.22E-12	9.14E-17	4	Ranbp3
0.515479102	1.23E-12	9.20E-17	4	Rrp36
0.657262316	1.24E-12	9.31E-17	4	Nsf
1.030192961	1.27E-12	9.55E-17	4	Ltv1
1.231728029	1.57E-12	1.18E-16	4	Zfp296
0.652464973	1.87E-12	1.40E-16	4	Snupn
0.616049906	2.29E-12	1.72E-16	4	Cpsf2
0.682331502	2.44E-12	1.83E-16	4	Fam107b
-1.013812863	2.45E-12	1.84E-16	4	Zfp36
0.821021508	2.56E-12	1.91E-16	4	Mvd
0.669702817	2.56E-12	1.92E-16	4	Dpep2
0.893195312	2.60E-12	1.95E-16	4	2310009B15Rik
0.634212265	2.73E-12	2.04E-16	4	Echdc1

0.800493293	2.77E-12	2.08E-16	4	Coq7
0.613258592	2.95E-12	2.21E-16	4	Stx8
0.882269196	3.17E-12	2.38E-16	4	Parn
-0.50048498	3.24E-12	2.43E-16	4	Cst3
0.659540324	3.98E-12	2.98E-16	4	Psma6
0.928534996	3.99E-12	2.99E-16	4	Cand1
0.63806837	4.34E-12	3.25E-16	4	Cd320
0.994841004	4.46E-12	3.34E-16	4	Cspg4
0.565899741	4.84E-12	3.63E-16	4	Nudcd3
0.689096848	4.90E-12	3.67E-16	4	Itpk1
0.86433404	5.61E-12	4.20E-16	4	Plcl1
0.634510169	5.85E-12	4.38E-16	4	Gbas
0.409812495	5.98E-12	4.48E-16	4	Pttg1
0.79601951	6.29E-12	4.71E-16	4	Bag6
0.598908451	6.37E-12	4.77E-16	4	Nop58
0.678824494	6.62E-12	4.96E-16	4	4833439L19Rik
1.0489202	8.13E-12	6.09E-16	4	Ece2
0.540684854	8.53E-12	6.39E-16	4	Arhgap22
0.626558768	8.56E-12	6.41E-16	4	Gpatch2
0.808137102	9.26E-12	6.94E-16	4	Arl5c
0.66511749	1.09E-11	8.15E-16	4	Aldoc
0.766635055	1.13E-11	8.47E-16	4	Phlpp1
1.016590338	1.20E-11	8.98E-16	4	Fbxw2
0.700113792	1.26E-11	9.41E-16	4	Mrpl54
0.290271903	1.28E-11	9.59E-16	4	Heatr6
0.752718886	1.53E-11	1.14E-15	4	Fbxl15
0.744330977	1.58E-11	1.18E-15	4	Cd180
0.923936203	1.73E-11	1.30E-15	4	Aak1
0.974342144	2.22E-11	1.67E-15	4	Plrg1
0.741243	2.26E-11	1.70E-15	4	Slc25a39
0.547062106	2.77E-11	2.07E-15	4	Hif1an
0.764935292	3.01E-11	2.25E-15	4	Slc35e4
0.5830576	3.03E-11	2.27E-15	4	Fnta
0.573414824	3.16E-11	2.37E-15	4	Tinf2
0.446173314	3.40E-11	2.55E-15	4	Prps1
0.460046457	3.75E-11	2.81E-15	4	Gtf2f1
0.566257732	4.04E-11	3.02E-15	4	Tmx4
0.658310332	4.20E-11	3.14E-15	4	Igf1
1.127240053	4.36E-11	3.27E-15	4	Afmid
0.572595788	4.45E-11	3.33E-15	4	Galnt7
0.696496506	4.52E-11	3.39E-15	4	Ddb2
0.482651341	4.62E-11	3.46E-15	4	Zbtb17
0.510388453	4.65E-11	3.48E-15	4	Prkch
-1.25843235	5.05E-11	3.78E-15	4	Lyz1
0.717110676	5.09E-11	3.81E-15	4	Maf1
0.677276656	5.26E-11	3.94E-15	4	Golgb1
0.423055837	5.56E-11	4.16E-15	4	Fam129a
0.546532497	5.65E-11	4.23E-15	4	Fam50a
0.506277725	5.74E-11	4.30E-15	4	Srfbp1
0.711454383	6.35E-11	4.76E-15	4	Bre
0.686155707	6.48E-11	4.85E-15	4	Tmem66
-0.332428903	6.51E-11	4.88E-15	4	B2m
0.589345945	6.52E-11	4.88E-15	4	Fam217b
0.542177645	7.17E-11	5.37E-15	4	Cx3cr1
0.549270782	7.63E-11	5.71E-15	4	Sec61a2
0.535137266	7.97E-11	5.97E-15	4	Uhrf1bp1
0.913304683	8.02E-11	6.00E-15	4	Wdr75
0.630294352	8.57E-11	6.42E-15	4	Traf7
0.710124305	8.74E-11	6.54E-15	4	Nudt19
0.631463645	8.95E-11	6.70E-15	4	Sod2
0.605194442	9.26E-11	6.94E-15	4	Dtx2

0.753123695	9.27E-11	6.94E-15	4	Rwdd4a
0.741738108	1.03E-10	7.74E-15	4	Mrps34
0.995097258	1.04E-10	7.77E-15	4	Gnptg
0.653568322	1.11E-10	8.28E-15	4	Polr2f
0.627876567	1.13E-10	8.47E-15	4	Polr2e
0.513616864	1.17E-10	8.79E-15	4	Cacul1
0.550546726	1.24E-10	9.30E-15	4	Pcyt1a
0.690221422	1.25E-10	9.35E-15	4	Surf4
0.480779399	1.35E-10	1.01E-14	4	Smad1
0.470491027	1.41E-10	1.06E-14	4	Mrpl34
0.41151355	1.48E-10	1.11E-14	4	Wdr18
0.905530569	1.50E-10	1.12E-14	4	Sez6l2
0.828891488	1.50E-10	1.13E-14	4	Nemf
0.522058287	1.50E-10	1.13E-14	4	Exosc4
0.609501689	1.68E-10	1.26E-14	4	Atp8b4
0.63469175	1.70E-10	1.28E-14	4	Rilpl1
0.353082718	1.74E-10	1.30E-14	4	Rexo1
0.777606173	1.77E-10	1.33E-14	4	Scyl2
0.792403941	1.81E-10	1.36E-14	4	Rilpl2
0.451135706	1.86E-10	1.40E-14	4	Nsdhl
0.609561302	1.95E-10	1.46E-14	4	Pfkp
0.832299009	2.08E-10	1.55E-14	4	Zbtb48
0.55039292	2.22E-10	1.67E-14	4	Ndrg3
0.844091312	2.28E-10	1.71E-14	4	Acot7
0.748212158	2.31E-10	1.73E-14	4	Cdk2ap2
0.628211075	2.68E-10	2.00E-14	4	Ppap2c
0.555500797	2.75E-10	2.06E-14	4	Zdhhc3
0.600921581	2.81E-10	2.10E-14	4	Cux1
0.711036137	2.84E-10	2.13E-14	4	Polr2i
0.656212711	2.86E-10	2.14E-14	4	Acot13
0.870663411	3.16E-10	2.37E-14	4	Cd276
0.707262317	3.35E-10	2.51E-14	4	Cblb
0.263795319	3.36E-10	2.52E-14	4	Btbd7
1.067297292	3.54E-10	2.65E-14	4	Trnau1ap
0.583816277	3.67E-10	2.75E-14	4	Zbtb34
0.459708457	3.85E-10	2.88E-14	4	Slc25a15
0.707329987	3.97E-10	2.98E-14	4	Stxbp1
0.967380391	4.43E-10	3.32E-14	4	Eif3b
0.657280449	4.48E-10	3.36E-14	4	Fam129b
0.736265593	4.50E-10	3.37E-14	4	Ercc6l2
0.606767929	4.53E-10	3.39E-14	4	Kdm1b
0.69146814	4.89E-10	3.66E-14	4	Pvr
0.633719953	5.33E-10	3.99E-14	4	Cmtm8
0.452376559	5.40E-10	4.05E-14	4	Mtmr10
0.713035688	6.02E-10	4.51E-14	4	Bag3
0.693726327	6.82E-10	5.11E-14	4	Pef1
0.607248792	7.10E-10	5.32E-14	4	A430005L14Rik
0.638278048	7.48E-10	5.60E-14	4	Slc11a2
0.603355884	7.59E-10	5.69E-14	4	Inpp5b
0.827521053	8.20E-10	6.14E-14	4	Mtg2
0.704648328	8.52E-10	6.38E-14	4	Fyn
0.631220251	9.00E-10	6.74E-14	4	Asap1
0.513491644	9.83E-10	7.36E-14	4	Cyp4v3
0.358134928	1.05E-09	7.84E-14	4	Emc8
0.790720118	1.07E-09	8.03E-14	4	Fam20b
0.451936484	1.09E-09	8.16E-14	4	Copz2
0.554942862	1.14E-09	8.53E-14	4	Pdia3
0.557691108	1.18E-09	8.85E-14	4	Zfp672
0.511576835	1.21E-09	9.06E-14	4	Extl3
0.584026297	1.42E-09	1.07E-13	4	Rac2
0.575673762	1.43E-09	1.07E-13	4	Exoc6b

0.486991984	1.45E-09	1.09E-13	4	Cytip
-0.337124849	1.47E-09	1.10E-13	4	Actb
0.636802338	1.51E-09	1.13E-13	4	Tep1
0.581707954	1.54E-09	1.15E-13	4	Adam8
0.704127979	1.55E-09	1.16E-13	4	Zdhhc4
0.478865054	1.60E-09	1.20E-13	4	Sugt1
0.678843987	1.63E-09	1.22E-13	4	Orc2
0.431218414	1.70E-09	1.27E-13	4	Ola1
0.325091273	1.78E-09	1.34E-13	4	Apex1
0.51219213	1.79E-09	1.34E-13	4	Slc15a3
0.979311099	1.94E-09	1.46E-13	4	Nr1h3
0.870563572	2.02E-09	1.52E-13	4	AI462493
0.889774328	2.03E-09	1.52E-13	4	Rusc2
0.789274453	2.03E-09	1.52E-13	4	Dcstamp
0.746538442	2.19E-09	1.64E-13	4	Pld3
0.591474533	2.24E-09	1.68E-13	4	Gpr126
0.564577372	2.35E-09	1.76E-13	4	Plec
0.416142397	2.47E-09	1.85E-13	4	Pbxip1
0.623295915	2.48E-09	1.86E-13	4	Il6st
0.577599687	2.56E-09	1.92E-13	4	Cct6a
0.889332769	2.57E-09	1.92E-13	4	Tchp
0.624717464	2.58E-09	1.93E-13	4	Lrig2
0.56070828	2.65E-09	1.98E-13	4	Sntb2
0.458900203	2.80E-09	2.10E-13	4	Ntan1
0.568700208	3.06E-09	2.29E-13	4	Cd200r1
0.781892838	3.23E-09	2.42E-13	4	D17Wsu104e
0.663816957	3.45E-09	2.58E-13	4	Tmem219
0.594597473	3.48E-09	2.61E-13	4	Anxa3
0.280532922	3.49E-09	2.61E-13	4	Akr1e1
1.252767365	3.51E-09	2.63E-13	4	Pinx1
0.59869032	3.51E-09	2.63E-13	4	Eif2b4
1.102391782	3.66E-09	2.74E-13	4	Pigq
0.729250039	3.68E-09	2.76E-13	4	Zfp142
0.369327336	3.69E-09	2.76E-13	4	Exoc4
0.623906668	3.86E-09	2.89E-13	4	Slc2a6
0.582881767	3.99E-09	2.99E-13	4	Pdgfa
0.418458819	4.41E-09	3.30E-13	4	Serpine1
0.283070991	4.42E-09	3.31E-13	4	1110001J03Rik
0.606512042	4.49E-09	3.36E-13	4	Atp7a
0.836312436	4.52E-09	3.39E-13	4	Mtx2
0.691476956	4.66E-09	3.49E-13	4	Cep250
1.077581674	4.77E-09	3.57E-13	4	Rasa4
1.271670957	4.94E-09	3.70E-13	4	Emr4
0.476505448	5.00E-09	3.75E-13	4	Klhl11
1.079177962	5.06E-09	3.79E-13	4	E130309D02Rik
0.560189332	5.30E-09	3.97E-13	4	Slc35e1
0.6955469	5.41E-09	4.05E-13	4	Rbm33
0.492060152	5.50E-09	4.12E-13	4	Dnaja3
0.600390312	6.05E-09	4.53E-13	4	Srf
0.532739425	6.11E-09	4.57E-13	4	Sec63
1.543833212	6.22E-09	4.66E-13	4	Yars
0.388848754	6.34E-09	4.74E-13	4	Rcan1
0.448052984	6.47E-09	4.84E-13	4	Bmf
0.779554208	6.61E-09	4.95E-13	4	Mrpl53
0.540694333	7.31E-09	5.47E-13	4	Ppp2ca
-0.349413389	7.44E-09	5.57E-13	4	Tmsb4x
0.666564794	7.92E-09	5.93E-13	4	Syt11
0.46267925	8.02E-09	6.01E-13	4	Mcm5
0.562672766	8.29E-09	6.21E-13	4	Ifitm1
0.388078611	8.47E-09	6.34E-13	4	Cmtm4
0.610219663	8.97E-09	6.71E-13	4	Il7r

0.384603528	9.09E-09	6.81E-13	4	Msl2
1.243359761	9.10E-09	6.82E-13	4	Ttyh2
0.666398808	9.50E-09	7.11E-13	4	Mpp6
0.403255453	9.71E-09	7.27E-13	4	Nsmce2
1.037414075	1.08E-08	8.07E-13	4	Ift122
0.53608587	1.10E-08	8.25E-13	4	Ccnd2
0.526694227	1.10E-08	8.25E-13	4	Crat
0.531510854	1.12E-08	8.37E-13	4	Tpd52l2
0.401140211	1.16E-08	8.69E-13	4	Smarca4
0.648116529	1.17E-08	8.76E-13	4	S100a1
0.446153309	1.22E-08	9.12E-13	4	Rab6a
0.520845032	1.25E-08	9.37E-13	4	Comtd1
0.392199319	1.26E-08	9.42E-13	4	Alg5
0.397984769	1.28E-08	9.58E-13	4	Triap1
0.620862535	1.29E-08	9.64E-13	4	Fgd4
0.251857277	1.36E-08	1.02E-12	4	Rcbtb2
0.66349316	1.38E-08	1.03E-12	4	Ppa1
0.472025807	1.40E-08	1.05E-12	4	Eri1
0.567672829	1.42E-08	1.06E-12	4	Fuca1
0.755134932	1.45E-08	1.09E-12	4	Chmp2b
0.471975255	1.57E-08	1.17E-12	4	Znrd1
0.473765606	1.57E-08	1.17E-12	4	Clec1b
0.536289101	1.59E-08	1.19E-12	4	Pak1ip1
0.580307401	1.61E-08	1.20E-12	4	G530011O06Rik
0.586689446	1.64E-08	1.23E-12	4	St7l
0.690677905	1.81E-08	1.36E-12	4	Crlf2
0.689669656	1.84E-08	1.38E-12	4	Fam168a
0.64513237	1.87E-08	1.40E-12	4	Scoc
0.63891971	1.88E-08	1.41E-12	4	Hspbp1
0.546802874	1.90E-08	1.43E-12	4	2210408F21Rik
0.523913157	1.92E-08	1.44E-12	4	Flnb
0.521716874	1.97E-08	1.47E-12	4	Tcp1
0.452965184	1.98E-08	1.48E-12	4	Psmd8
0.666422548	2.08E-08	1.56E-12	4	Ubash3b
0.790801916	2.21E-08	1.66E-12	4	Mefv
0.621169659	2.49E-08	1.87E-12	4	Gsto1
0.573601088	2.49E-08	1.87E-12	4	Eif3l
1.064199063	2.80E-08	2.10E-12	4	Hipk2
0.670826889	2.81E-08	2.10E-12	4	Wdr55
0.497149193	2.85E-08	2.14E-12	4	B230118H07Rik
0.549590561	2.89E-08	2.17E-12	4	AB041803
0.76010664	2.98E-08	2.23E-12	4	Eif2ak4
0.763062901	3.00E-08	2.25E-12	4	Rnf14
0.50890907	3.03E-08	2.27E-12	4	Lpl
0.455784547	3.52E-08	2.64E-12	4	Ubqln1
0.737094568	3.77E-08	2.82E-12	4	Sfswap
0.479397817	3.87E-08	2.90E-12	4	Kat8
0.493476888	3.93E-08	2.94E-12	4	Srp19
0.484803449	3.94E-08	2.95E-12	4	Tacc1
0.481234819	4.00E-08	2.99E-12	4	Icam1
0.372051267	4.01E-08	3.00E-12	4	Znhit2
0.595369586	4.27E-08	3.20E-12	4	Timm17a
0.589131583	4.28E-08	3.21E-12	4	Lmf1
0.434081414	4.28E-08	3.21E-12	4	Itgb1
0.534096989	4.31E-08	3.23E-12	4	Hint2
0.342258947	4.61E-08	3.45E-12	4	Hsd17b4
0.752944991	4.62E-08	3.46E-12	4	Slc12a2
0.775842098	4.83E-08	3.61E-12	4	Mier2
0.550393707	4.85E-08	3.63E-12	4	Fiz1
0.556958867	4.86E-08	3.64E-12	4	Sra1
0.471757289	5.02E-08	3.76E-12	4	Neurl3

0.932510708	5.05E-08	3.78E-12	4	Arhgef7
-1.197267167	5.08E-08	3.80E-12	4	Tgif1
0.948498083	5.09E-08	3.81E-12	4	D6Wsu163e
0.527597954	5.11E-08	3.83E-12	4	Gusb
0.499440436	5.24E-08	3.93E-12	4	Alkbh2
0.527099984	5.46E-08	4.09E-12	4	Naa38
0.589818699	5.56E-08	4.16E-12	4	Ccdc77
0.489850465	5.79E-08	4.34E-12	4	1110007C09Rik
0.539812913	5.84E-08	4.38E-12	4	B4galt5
0.625218253	6.04E-08	4.52E-12	4	Chordc1
0.847794613	6.08E-08	4.56E-12	4	Cnnm2
0.353039371	6.34E-08	4.75E-12	4	Ndufs2
0.396644861	6.52E-08	4.88E-12	4	Arhgef3
0.402610207	6.68E-08	5.00E-12	4	Pdlim1
0.526549081	6.86E-08	5.14E-12	4	Sqrdl
0.334918116	6.88E-08	5.15E-12	4	Ppcdc
0.505979181	7.12E-08	5.33E-12	4	2010111I01Rik
0.407890749	7.18E-08	5.38E-12	4	Ppfibp1
0.51249031	7.31E-08	5.48E-12	4	Tmem205
0.562080896	7.39E-08	5.53E-12	4	Mrpl20
0.73666109	7.39E-08	5.54E-12	4	Zbtb20
1.239984478	7.58E-08	5.68E-12	4	Tbl1x
0.586732706	7.65E-08	5.73E-12	4	Rps19bp1
0.462514994	7.69E-08	5.76E-12	4	Arid4b
0.553617431	7.85E-08	5.88E-12	4	R3hdm1
1.078503197	7.88E-08	5.90E-12	4	Snrnp35
0.597329368	7.96E-08	5.96E-12	4	Psmd12
0.869677861	8.01E-08	6.00E-12	4	Ercc8
0.484842459	8.11E-08	6.08E-12	4	Pqlc1
0.643474696	8.29E-08	6.21E-12	4	Anapc5
0.532136102	8.30E-08	6.22E-12	4	Eif4enif1
0.752557144	8.32E-08	6.23E-12	4	Gmeb1
0.622321602	8.74E-08	6.55E-12	4	Rab28
0.472592658	9.04E-08	6.77E-12	4	Flii
0.763991031	9.08E-08	6.80E-12	4	Crem
0.843436671	9.16E-08	6.86E-12	4	Cpsf3
0.319766404	9.21E-08	6.90E-12	4	Utrn
0.623373505	9.69E-08	7.26E-12	4	G6pc3
0.560679028	1.02E-07	7.64E-12	4	Mfsd6
0.300649106	1.11E-07	8.32E-12	4	Rnf214
0.930423	1.15E-07	8.58E-12	4	Trim33
0.479078018	1.16E-07	8.66E-12	4	Anapc13
0.910922467	1.17E-07	8.74E-12	4	Kctd10
0.579282792	1.17E-07	8.76E-12	4	Rnf220
0.260226168	1.18E-07	8.82E-12	4	Mrpl48
0.672832222	1.20E-07	8.96E-12	4	Baiap2
0.6355559	1.20E-07	8.97E-12	4	Rpp30
0.264044199	1.21E-07	9.10E-12	4	Abcb9
0.377921428	1.24E-07	9.26E-12	4	Etfdh
0.581747995	1.26E-07	9.41E-12	4	Dtnbp1
1.039526727	1.31E-07	9.80E-12	4	Cep83
0.302384921	1.33E-07	9.95E-12	4	Sypl
0.514350157	1.35E-07	1.01E-11	4	Dst
0.38445202	1.37E-07	1.02E-11	4	Fam134c
0.38774274	1.39E-07	1.04E-11	4	Ulk1
0.596658445	1.47E-07	1.10E-11	4	Chchd5
0.349511784	1.50E-07	1.12E-11	4	Cdkn2aipnl
0.654765007	1.66E-07	1.25E-11	4	Mob1a
0.792114256	1.73E-07	1.29E-11	4	Diap1
0.531640495	1.76E-07	1.31E-11	4	Hvcn1
1.41678516	1.76E-07	1.32E-11	4	Mycbp

0.694239672	1.78E-07	1.34E-11	4	Pold3
0.528247702	1.99E-07	1.49E-11	4	Tspan13
0.570737629	2.04E-07	1.53E-11	4	Polr3k
0.425063466	2.19E-07	1.64E-11	4	BC003965
0.70464154	2.22E-07	1.66E-11	4	Psmd1
0.443147142	2.25E-07	1.68E-11	4	Carkd
0.699929174	2.35E-07	1.76E-11	4	Actn1
0.637961388	2.43E-07	1.82E-11	4	Ipo7
0.719223719	2.46E-07	1.85E-11	4	Rc3h1
0.411686487	2.56E-07	1.91E-11	4	Tars2
0.566963108	2.57E-07	1.92E-11	4	Gorasp2
0.358969642	2.65E-07	1.99E-11	4	Tlk1
0.642867711	2.66E-07	1.99E-11	4	Rabl3
0.449235381	2.82E-07	2.12E-11	4	Trim2
0.250874027	2.84E-07	2.12E-11	4	Endod1
1.294428421	2.85E-07	2.13E-11	4	Stradb
0.322328594	3.03E-07	2.27E-11	4	Erap1
0.452752348	3.04E-07	2.28E-11	4	Mrpl24
0.585918963	3.14E-07	2.35E-11	4	Cdc25b
0.40908358	3.19E-07	2.39E-11	4	Psph
0.58443609	3.23E-07	2.42E-11	4	Emc4
0.717393762	3.25E-07	2.43E-11	4	Gab2
0.481199385	3.29E-07	2.46E-11	4	Nap1l4
0.490998311	3.40E-07	2.54E-11	4	Atp5g3
0.319867438	3.51E-07	2.63E-11	4	Fam20c
0.464610763	3.55E-07	2.66E-11	4	Tmem229b
0.438592066	3.56E-07	2.67E-11	4	Rbm10
0.589520771	3.58E-07	2.68E-11	4	Cpd
0.595920816	3.66E-07	2.74E-11	4	Zranb1
0.71657801	3.72E-07	2.79E-11	4	Eva1b
0.527379033	3.72E-07	2.79E-11	4	Mrpl16
0.543497756	3.77E-07	2.82E-11	4	Hck
0.50196176	3.93E-07	2.95E-11	4	Napa
0.338220114	4.06E-07	3.04E-11	4	Ptprcap
0.515078412	4.18E-07	3.13E-11	4	Rtca
0.451773617	4.42E-07	3.31E-11	4	Dbndd2
1.170820597	4.43E-07	3.32E-11	4	Vps13a
0.298469801	4.56E-07	3.41E-11	4	Mamdc2
0.390050787	4.67E-07	3.50E-11	4	Peak1
0.437717648	4.82E-07	3.61E-11	4	Gde1
0.629905032	4.87E-07	3.65E-11	4	Slc11a1
0.439200356	4.93E-07	3.69E-11	4	Oxr1
0.507746239	4.99E-07	3.73E-11	4	Setd2
0.843602667	4.99E-07	3.74E-11	4	1110012L19Rik
0.435394425	5.46E-07	4.09E-11	4	Tmem55a
0.333869083	5.65E-07	4.23E-11	4	Brcc3
0.421149567	5.74E-07	4.30E-11	4	Abtb2
0.446913349	5.82E-07	4.36E-11	4	Mrps25
0.443321235	5.94E-07	4.45E-11	4	Xpo7
0.405227434	5.95E-07	4.46E-11	4	Slc3a2
0.869337138	5.99E-07	4.49E-11	4	Kctd17
0.409980865	6.03E-07	4.52E-11	4	Ykt6
0.534348474	6.21E-07	4.65E-11	4	Anxa6
0.457266607	6.24E-07	4.68E-11	4	Casc3
0.613140685	6.27E-07	4.70E-11	4	Rhoc
1.060084688	6.29E-07	4.71E-11	4	Hmgcr
0.491260468	6.34E-07	4.75E-11	4	Snrnp25
0.57786437	6.38E-07	4.78E-11	4	Ndufaf2
0.355124073	6.47E-07	4.85E-11	4	Sec61a1
0.516604959	6.60E-07	4.94E-11	4	Ammecr1l
-0.282024513	6.65E-07	4.98E-11	4	Malat1

0.377309035	7.07E-07	5.29E-11	4	Ptpra
0.555102422	7.13E-07	5.34E-11	4	Lrrfip1
0.306158725	7.17E-07	5.37E-11	4	N4bp2
0.508801148	7.31E-07	5.48E-11	4	Brd2
0.268568417	7.33E-07	5.49E-11	4	Igbp1
0.988275925	7.34E-07	5.50E-11	4	Gpatch4
0.392289566	7.35E-07	5.51E-11	4	Runx3
0.506246004	7.41E-07	5.55E-11	4	Rabac1
0.293902305	7.51E-07	5.62E-11	4	Tia1
0.468558534	8.13E-07	6.09E-11	4	Birc6
0.485629787	8.27E-07	6.20E-11	4	Laptm4b
0.267149246	8.79E-07	6.59E-11	4	Stap1
0.5184028	8.89E-07	6.66E-11	4	Trappc5
0.318356274	8.91E-07	6.67E-11	4	Immt
0.569038818	9.03E-07	6.76E-11	4	Wdr91
0.498018257	9.20E-07	6.89E-11	4	Kif16b
0.544539233	1.06E-06	7.91E-11	4	BC004004
0.567605432	1.07E-06	7.99E-11	4	Ywhaq
0.270270071	1.07E-06	8.03E-11	4	Bach1
0.890308419	1.08E-06	8.07E-11	4	Dgcr14
0.510817377	1.14E-06	8.51E-11	4	Ndufa8
0.54777262	1.15E-06	8.59E-11	4	Chid1
0.29610713	1.16E-06	8.68E-11	4	Tbpl1
0.495692604	1.17E-06	8.74E-11	4	Limd2
0.367429738	1.18E-06	8.87E-11	4	Psmc5
1.002444612	1.20E-06	9.02E-11	4	Ago1
0.92533716	1.21E-06	9.04E-11	4	Stk16
0.339871417	1.24E-06	9.32E-11	4	Sap30l
0.58955221	1.30E-06	9.73E-11	4	Lrpprc
0.292867764	1.34E-06	1.00E-10	4	Zmpste24
0.369742191	1.37E-06	1.03E-10	4	Zdhhc20
0.494007281	1.38E-06	1.04E-10	4	Nipbl
-1.001757279	1.40E-06	1.05E-10	4	Cebpb
0.784188836	1.46E-06	1.09E-10	4	Ndufaf5
0.648726064	1.47E-06	1.10E-10	4	Slk
0.50245013	1.50E-06	1.12E-10	4	Letmd1
0.614840281	1.53E-06	1.14E-10	4	Elp5
0.402664278	1.53E-06	1.15E-10	4	Map7d1
0.518826505	1.55E-06	1.16E-10	4	AK010878
0.409942456	1.59E-06	1.19E-10	4	Ppt1
0.383931922	1.62E-06	1.21E-10	4	Sltm
0.425464616	1.63E-06	1.22E-10	4	Havcr2
0.377372807	1.66E-06	1.24E-10	4	Zfp637
0.68582345	1.84E-06	1.38E-10	4	Pik3r5
0.609876813	1.84E-06	1.38E-10	4	Mtf2
0.590730588	1.89E-06	1.42E-10	4	Dohh
0.821517571	1.91E-06	1.43E-10	4	Ntmt1
0.357260363	1.93E-06	1.45E-10	4	Gadd45g
0.522326772	2.00E-06	1.50E-10	4	Slc39a9
-0.436702393	2.00E-06	1.50E-10	4	Junb
0.766929973	2.09E-06	1.57E-10	4	Bud13
0.298451498	2.10E-06	1.57E-10	4	Pus7
0.535963285	2.10E-06	1.58E-10	4	Tmem86a
0.418357225	2.37E-06	1.78E-10	4	Cd97
0.446801244	2.46E-06	1.84E-10	4	Mob2
0.432466039	2.48E-06	1.86E-10	4	Slamf9
0.621562723	2.48E-06	1.86E-10	4	Stk24
0.493175813	2.53E-06	1.89E-10	4	Coa3
0.351542521	2.55E-06	1.91E-10	4	Eea1
0.678085562	2.57E-06	1.93E-10	4	Cyb5r3
0.532959972	2.58E-06	1.94E-10	4	Prpf40a

0.701766716	2.69E-06	2.01E-10	4	R3hcc1l
0.330950175	2.77E-06	2.08E-10	4	Ttc17
0.391171907	2.82E-06	2.11E-10	4	Aip
0.373087664	2.82E-06	2.11E-10	4	Lsp1
0.804223203	2.87E-06	2.15E-10	4	Trappc1
0.470978045	2.88E-06	2.16E-10	4	Trex1
0.649875676	2.92E-06	2.19E-10	4	Dstn
0.918762595	3.06E-06	2.29E-10	4	Mfsd11
0.411358072	3.07E-06	2.30E-10	4	Ythdf2
0.542307854	3.10E-06	2.33E-10	4	Cd151
0.61303553	3.13E-06	2.35E-10	4	Elovl1
0.507388239	3.18E-06	2.38E-10	4	Cd93
0.630402393	3.18E-06	2.38E-10	4	Skiv2l2
0.401465367	3.21E-06	2.40E-10	4	Vipas39
0.467185108	3.22E-06	2.41E-10	4	Arih2
0.414950283	3.33E-06	2.50E-10	4	Polr2a
0.331790639	3.50E-06	2.62E-10	4	Gemin7
0.990499732	3.68E-06	2.75E-10	4	Ttc27
0.754380511	3.81E-06	2.85E-10	4	Tmem70
0.445710399	3.88E-06	2.90E-10	4	Usp16
0.292315935	4.01E-06	3.00E-10	4	Prps2
0.320385607	4.12E-06	3.09E-10	4	Mcm6
0.432122319	4.27E-06	3.20E-10	4	Syngr1
0.507268649	4.34E-06	3.25E-10	4	Smn1
0.410658785	4.41E-06	3.30E-10	4	Tmem179b
0.483463512	4.41E-06	3.31E-10	4	Lasp1
0.40652275	4.42E-06	3.31E-10	4	Elovl5
0.449191354	4.72E-06	3.54E-10	4	Trak1
0.580904763	4.79E-06	3.59E-10	4	Tmem42
0.302467771	4.83E-06	3.62E-10	4	Prdx6
0.568506338	5.15E-06	3.86E-10	4	Mrps6
0.593807886	5.28E-06	3.96E-10	4	Crkl
0.485308739	5.33E-06	3.99E-10	4	Il1rn
0.780081866	5.38E-06	4.03E-10	4	Plac8
-0.558748467	5.39E-06	4.04E-10	4	Rps18
0.422597926	5.53E-06	4.14E-10	4	Fopnl
0.444329733	5.59E-06	4.18E-10	4	Aco1
0.484428075	5.71E-06	4.28E-10	4	Pilrb2
0.252547209	5.75E-06	4.30E-10	4	Snrnp48
0.26598424	5.78E-06	4.33E-10	4	Blzf1
0.512647663	5.84E-06	4.37E-10	4	Tmem11
0.632867562	5.86E-06	4.39E-10	4	Akr1b10
0.591241523	5.94E-06	4.45E-10	4	Glg1
0.27454811	6.12E-06	4.59E-10	4	Ralbp1
0.406657511	6.15E-06	4.60E-10	4	Eprs
0.412077804	6.61E-06	4.95E-10	4	Stk38
0.633553171	6.63E-06	4.97E-10	4	Smarcd1
-1.798949118	6.66E-06	4.99E-10	4	Maf
0.402669152	6.76E-06	5.06E-10	4	Ifnar2
0.427568778	6.84E-06	5.13E-10	4	Cep350
0.262372307	6.86E-06	5.13E-10	4	H2-Oa
0.848670438	6.96E-06	5.21E-10	4	Trim35
0.532627216	7.33E-06	5.49E-10	4	D930015E06Rik
0.384323358	7.35E-06	5.50E-10	4	Api5
0.538580849	7.35E-06	5.50E-10	4	Cwc25
0.996135759	7.45E-06	5.58E-10	4	Mrps30
0.554929166	7.58E-06	5.68E-10	4	Qtrt1
0.337217572	7.67E-06	5.75E-10	4	Gdf15
0.548168654	7.81E-06	5.85E-10	4	Msra
0.71758966	7.97E-06	5.97E-10	4	Por
0.555554987	7.98E-06	5.98E-10	4	Pds5b

0.377367489	7.98E-06	5.98E-10	4	Sptan1
0.469652622	8.03E-06	6.01E-10	4	Fis1
0.401457714	8.08E-06	6.05E-10	4	Trem2
0.405049456	8.41E-06	6.30E-10	4	Cdk18
0.267544802	8.52E-06	6.38E-10	4	Aarsd1
0.264413502	8.61E-06	6.45E-10	4	Cks1b
0.585385	8.85E-06	6.63E-10	4	Alcam
0.987809147	9.09E-06	6.81E-10	4	Ercc6
1.221738821	9.17E-06	6.87E-10	4	Naf1
0.679955716	9.57E-06	7.16E-10	4	Rps6kb2
-0.63392573	9.57E-06	7.17E-10	4	Jund
0.664764247	9.60E-06	7.19E-10	4	Abcd4
0.354962768	9.66E-06	7.24E-10	4	Sde2
0.347676803	9.78E-06	7.32E-10	4	Jdp2
0.693582068	9.80E-06	7.34E-10	4	Zfp318
0.553711386	1.00E-05	7.51E-10	4	Rfc3
0.336989436	1.02E-05	7.65E-10	4	Pon3
0.848657816	1.02E-05	7.65E-10	4	Pptc7
0.349646827	1.03E-05	7.72E-10	4	Wasf2
0.474182632	1.05E-05	7.88E-10	4	A930013F10Rik
0.405000001	1.05E-05	7.89E-10	4	Syne1
0.569010657	1.09E-05	8.16E-10	4	Tmed10
0.46798247	1.10E-05	8.21E-10	4	Upf2
0.679068273	1.10E-05	8.26E-10	4	Sh3tc1
0.378063412	1.15E-05	8.58E-10	4	Gpr132
0.711924857	1.15E-05	8.64E-10	4	Ms4a4b
0.365152504	1.18E-05	8.80E-10	4	Csnk1d
0.39134483	1.18E-05	8.81E-10	4	Atg101
0.450133565	1.18E-05	8.83E-10	4	Ints4
0.39596556	1.21E-05	9.08E-10	4	Sigmar1
0.57814634	1.35E-05	1.01E-09	4	Cd5
0.341022353	1.39E-05	1.04E-09	4	Pdzd8
0.571467296	1.43E-05	1.07E-09	4	Cltb
0.518950027	1.48E-05	1.11E-09	4	Letm1
0.447616351	1.48E-05	1.11E-09	4	Zfp524
0.771912889	1.49E-05	1.11E-09	4	Wdr74
0.464749644	1.53E-05	1.15E-09	4	Nucb2
0.379964915	1.54E-05	1.15E-09	4	Rwdd1
0.31015813	1.56E-05	1.17E-09	4	Pigf
0.57057105	1.59E-05	1.19E-09	4	Clasp1
0.982767817	1.63E-05	1.22E-09	4	Ttc12
0.365467038	1.63E-05	1.22E-09	4	Nhp2
0.674240275	1.66E-05	1.24E-09	4	Stam2
0.369979802	1.72E-05	1.29E-09	4	Rit1
0.771743879	1.72E-05	1.29E-09	4	Myo10
0.635776553	1.74E-05	1.30E-09	4	Scd1
0.448324138	1.74E-05	1.30E-09	4	Slc25a33
0.266273466	1.84E-05	1.38E-09	4	Mrpl17
0.45601458	1.95E-05	1.46E-09	4	Pggt1b
0.40244211	1.98E-05	1.48E-09	4	Capg
0.377528517	1.98E-05	1.48E-09	4	Ctcf
0.347300147	1.98E-05	1.49E-09	4	Ddx24
0.31857327	1.99E-05	1.49E-09	4	Cbr3
0.689275843	2.04E-05	1.53E-09	4	Napg
-0.579499432	2.12E-05	1.59E-09	4	H2-K1
0.445939499	2.19E-05	1.64E-09	4	Mdm2
0.493320232	2.24E-05	1.68E-09	4	Phactr4
0.482750708	2.24E-05	1.68E-09	4	Ext1
0.784851599	2.25E-05	1.69E-09	4	Ticam1
0.313028442	2.27E-05	1.70E-09	4	Ech1
0.37653284	2.34E-05	1.75E-09	4	Arl5b

0.288741256	2.36E-05	1.77E-09	4	Ap1g1
0.637488801	2.39E-05	1.79E-09	4	Ift43
0.583985444	2.42E-05	1.81E-09	4	Atad5
0.512516247	2.48E-05	1.85E-09	4	Fdx1
0.890475144	2.59E-05	1.94E-09	4	Arhgap6
0.603692868	2.63E-05	1.97E-09	4	Mmgt1
0.296093085	2.64E-05	1.98E-09	4	Dnttip2
0.376072463	2.64E-05	1.98E-09	4	Vimp
-0.619656124	2.65E-05	1.98E-09	4	Sat1
1.177587474	2.65E-05	1.99E-09	4	Nudt6
0.340887301	2.66E-05	1.99E-09	4	Prpf38a
0.558266882	2.69E-05	2.02E-09	4	Fam96b
0.532144782	2.79E-05	2.09E-09	4	Rest
0.502444989	2.80E-05	2.10E-09	4	Chmp6
0.418439881	2.91E-05	2.18E-09	4	Uvrag
1.033862792	2.96E-05	2.22E-09	4	Caprin1
0.395156389	3.04E-05	2.28E-09	4	Sdf4
0.401418727	3.13E-05	2.35E-09	4	Uba1
0.435373373	3.14E-05	2.35E-09	4	S100a4
0.330461	3.19E-05	2.39E-09	4	Tcf3
0.305942638	3.21E-05	2.41E-09	4	Foxn3
0.844208678	3.22E-05	2.41E-09	4	Gm5617
0.374500252	3.45E-05	2.58E-09	4	Rps6ka1
0.37429829	3.62E-05	2.71E-09	4	Tln1
0.446757261	3.87E-05	2.90E-09	4	Slc6a8
0.279250567	3.91E-05	2.92E-09	4	Ccdc23
0.301325587	3.91E-05	2.93E-09	4	Usp8
0.287979817	3.95E-05	2.96E-09	4	Adam17
0.365107517	3.97E-05	2.97E-09	4	Vdac2
0.853657039	4.27E-05	3.20E-09	4	Ermp1
0.300368598	4.47E-05	3.35E-09	4	3110043O21Rik
-0.750363291	4.49E-05	3.36E-09	4	Rpl39
0.372978079	4.50E-05	3.37E-09	4	Arpp19
0.263928051	4.53E-05	3.39E-09	4	Prkcb
0.634521564	4.74E-05	3.55E-09	4	Arfgap1
0.565366894	4.79E-05	3.59E-09	4	Dhfr
0.317837314	4.84E-05	3.62E-09	4	Fkrp
0.724558488	4.84E-05	3.63E-09	4	Yif1a
0.506301155	4.86E-05	3.64E-09	4	D8Ertd82e
0.564780446	4.89E-05	3.66E-09	4	Ube2a
0.548757101	4.98E-05	3.73E-09	4	Rpain
0.425063466	4.99E-05	3.74E-09	4	Ercc5
0.309645896	5.04E-05	3.77E-09	4	Ncf4
0.386666284	5.04E-05	3.78E-09	4	Ddost
-1.244826887	5.05E-05	3.78E-09	4	Ier3
0.3901819	5.11E-05	3.83E-09	4	Ptpn6
0.441417847	5.52E-05	4.13E-09	4	Bop1
0.579480139	5.52E-05	4.13E-09	4	Cyb5d2
0.48080105	5.58E-05	4.18E-09	4	Slc48a1
0.890469386	5.90E-05	4.42E-09	4	Arhgap10
0.422241953	6.04E-05	4.52E-09	4	Hsd17b12
0.432560049	6.24E-05	4.67E-09	4	Msantd4
0.679687891	6.44E-05	4.82E-09	4	Traf1
0.407240983	6.67E-05	5.00E-09	4	Cox7a2
0.421870893	7.02E-05	5.26E-09	4	Rhoh
0.347905099	7.08E-05	5.31E-09	4	Ell2
0.306821399	7.17E-05	5.37E-09	4	Tfam
0.303030859	7.39E-05	5.53E-09	4	Phf7
0.457078796	7.40E-05	5.54E-09	4	Akip1
0.415387266	7.50E-05	5.62E-09	4	Aprt
0.965457291	7.78E-05	5.83E-09	4	Pdzd11

0.449345809	7.82E-05	5.86E-09	4	Arhgap24
0.427786752	7.88E-05	5.90E-09	4	Grcc10
1.212467896	7.95E-05	5.95E-09	4	Nrf1
0.531260778	7.95E-05	5.96E-09	4	Lrrc59
0.605500008	8.19E-05	6.14E-09	4	Rad52
-0.748608722	8.76E-05	6.56E-09	4	Rpl37a
0.422974571	8.82E-05	6.60E-09	4	Cox5a
0.629577131	8.82E-05	6.60E-09	4	Cct7
0.476356761	8.86E-05	6.64E-09	4	Abcc1
0.319139449	8.94E-05	6.70E-09	4	4921524J17Rik
0.390739517	9.14E-05	6.85E-09	4	Ckb
0.424162121	9.21E-05	6.89E-09	4	Abcg1
0.369597927	9.29E-05	6.96E-09	4	Anxa7
0.351847056	9.38E-05	7.02E-09	4	Chchd1
0.490515647	9.39E-05	7.03E-09	4	Cdt1
0.419793993	9.47E-05	7.09E-09	4	Tmem208
0.52561943	9.73E-05	7.29E-09	4	Daglb
0.281010695	0.000101806	7.62E-09	4	Josd1
0.304135324	0.000105232	7.88E-09	4	U2surp
0.815937271	0.00010677	8.00E-09	4	Vps41
0.260416122	0.000107671	8.06E-09	4	Tmem43
0.534075242	0.000108381	8.12E-09	4	Fra10ac1
0.293468572	0.000108417	8.12E-09	4	Prpf38b
0.401168238	0.000109969	8.24E-09	4	2310036O22Rik
0.958854333	0.000111097	8.32E-09	4	Map4k2
0.620659266	0.00011245	8.42E-09	4	Dync1h1
0.939353797	0.000112685	8.44E-09	4	Srpr
0.318255236	0.000112739	8.44E-09	4	2410006H16Rik
0.326835798	0.000112795	8.45E-09	4	Nceh1
0.256004274	0.000112838	8.45E-09	4	Pde4b
0.287015925	0.000113807	8.52E-09	4	Jtb
0.337604161	0.000115503	8.65E-09	4	Lxn
0.786453644	0.000117424	8.79E-09	4	Dcun1d5
0.607737198	0.000119414	8.94E-09	4	Stx5a
0.368014997	0.000121719	9.12E-09	4	Chd2
0.347341679	0.00012281	9.20E-09	4	Bmi1
0.413614109	0.000123501	9.25E-09	4	Sec31a
0.255648725	0.000126036	9.44E-09	4	Acadm
0.441584965	0.000127188	9.53E-09	4	Slc7a6os
0.399264468	0.000127238	9.53E-09	4	Lipa
0.870188945	0.000127622	9.56E-09	4	Slc50a1
0.289161468	0.000133751	1.00E-08	4	Nob1
0.48222188	0.000138188	1.03E-08	4	Ubr5
0.478633555	0.000140796	1.05E-08	4	Prpf31
0.351596314	0.000145229	1.09E-08	4	Dhrs1
0.303102151	0.000153437	1.15E-08	4	Phf23
0.284579821	0.000153604	1.15E-08	4	Nat9
0.322931582	0.000154513	1.16E-08	4	Mtmr14
0.271571331	0.00016266	1.22E-08	4	Epc1
0.482517405	0.00016311	1.22E-08	4	Opa1
0.512371976	0.000163763	1.23E-08	4	Trappc9
0.782161154	0.000164432	1.23E-08	4	Extl2
0.516201178	0.000166548	1.25E-08	4	Igf2r
0.483027158	0.000166747	1.25E-08	4	Zbp1
-1.663303291	0.000167481	1.25E-08	4	Mrc1
0.325721023	0.000169988	1.27E-08	4	Trappc4
0.273700992	0.000170289	1.28E-08	4	Smu1
0.361367217	0.00017235	1.29E-08	4	Rhoq
0.355330128	0.000177613	1.33E-08	4	Ralgapa2
0.660482941	0.000183811	1.38E-08	4	Dctn3
0.320367453	0.000186707	1.40E-08	4	Lrp1

0.339204675	0.000187776	1.41E-08	4	Ndufa10
0.344142024	0.000188951	1.42E-08	4	Ccdc107
0.34520703	0.000189068	1.42E-08	4	Eif3d
0.282701994	0.000189804	1.42E-08	4	Wdr48
0.345629512	0.000190518	1.43E-08	4	Vps36
0.581319921	0.00019708	1.48E-08	4	Sh2b2
0.710352239	0.000201791	1.51E-08	4	Prpf6
0.52306268	0.000202621	1.52E-08	4	Kbtbd3
0.317076637	0.000204607	1.53E-08	4	Top1
0.548225025	0.000205227	1.54E-08	4	Iah1
0.291631256	0.000205928	1.54E-08	4	Cct8
0.36961384	0.000206203	1.54E-08	4	Psme2b
0.41594729	0.000207369	1.55E-08	4	Sppl2a
0.554661717	0.000211058	1.58E-08	4	Ptplb
0.541340656	0.000212841	1.59E-08	4	Adcy3
1.089145332	0.000220476	1.65E-08	4	Hdac7
0.340933812	0.000221604	1.66E-08	4	Cnih4
0.345707808	0.000224108	1.68E-08	4	Cirh1a
0.795416631	0.000233117	1.75E-08	4	Tmem135
0.319153827	0.000236272	1.77E-08	4	Tgfbr1
0.816215439	0.000241956	1.81E-08	4	Dnajc4
0.382010951	0.000244363	1.83E-08	4	Ripk3
0.268702841	0.00024849	1.86E-08	4	Pop4
0.489287571	0.000251413	1.88E-08	4	Cdc16
0.482602025	0.00025256	1.89E-08	4	Gatad1
0.31652942	0.000258773	1.94E-08	4	Cbl
0.525140286	0.000267031	2.00E-08	4	Rasa1
0.383671369	0.000268683	2.01E-08	4	Metap1d
0.359446813	0.000280512	2.10E-08	4	Clic4
0.320923207	0.00028127	2.11E-08	4	Ctdspl
0.947284177	0.000282426	2.12E-08	4	Wdr83
0.447425829	0.000286767	2.15E-08	4	Pold4
0.332733915	0.000293747	2.20E-08	4	Lin37
0.275698365	0.000293826	2.20E-08	4	Snap23
0.351902586	0.000294996	2.21E-08	4	Rcor1
0.680399733	0.00029788	2.23E-08	4	Prkrip1
0.386638709	0.000302592	2.27E-08	4	Mtor
0.494924268	0.000302974	2.27E-08	4	Bcl2
0.337489185	0.000303593	2.27E-08	4	Ric8
0.440415076	0.000310634	2.33E-08	4	Chmp7
0.48494811	0.0003125	2.34E-08	4	Arl2
0.613654636	0.000313366	2.35E-08	4	Vps8
0.83143347	0.000318556	2.39E-08	4	Pcp4l1
-1.920145276	0.00032261	2.42E-08	4	C4b
0.272081238	0.000327427	2.45E-08	4	Dnase1l1
0.473802149	0.000335289	2.51E-08	4	Galns
0.644789205	0.000339483	2.54E-08	4	Tmed1
0.344689248	0.000346235	2.59E-08	4	Ssb
1.322129392	0.000348307	2.61E-08	4	Rhof
0.478259711	0.000350694	2.63E-08	4	Arid2
0.363638844	0.000353488	2.65E-08	4	Mfn2
0.623508093	0.000362057	2.71E-08	4	Mbp
0.335207137	0.000375957	2.82E-08	4	Smek2
-1.0144294	0.000382674	2.87E-08	4	Coro1a
0.261796953	0.000382712	2.87E-08	4	Abhd5
0.350792042	0.00038591	2.89E-08	4	Sh3kbp1
0.293393561	0.000391165	2.93E-08	4	Map4
-0.576330796	0.000397005	2.97E-08	4	Rps3a1
1.164357249	0.000406468	3.04E-08	4	Trim16
0.39501866	0.000420391	3.15E-08	4	Churc1
0.384425185	0.000422967	3.17E-08	4	Os9

0.386828437	0.000426934	3.20E-08	4	Cct5
0.340797847	0.000429799	3.22E-08	4	Tns3
-1.505183569	0.000441316	3.31E-08	4	Ccl8
0.330144791	0.00044653	3.34E-08	4	Fnip2
0.250360554	0.000449644	3.37E-08	4	Snw1
0.76884463	0.00045156	3.38E-08	4	Ceacam1
0.602982962	0.000467438	3.50E-08	4	Abcg3
0.578152422	0.000485797	3.64E-08	4	Ehd1
0.361182573	0.000490035	3.67E-08	4	Hcfc1r1
0.40669372	0.000495136	3.71E-08	4	Lpxn
0.745831263	0.000497039	3.72E-08	4	1190002N15Rik
0.390521094	0.000499294	3.74E-08	4	4931406P16Rik
0.304048808	0.000501468	3.76E-08	4	Vps29
0.364618018	0.000504766	3.78E-08	4	Fundc2
0.466265307	0.000512999	3.84E-08	4	Ankrd33b
0.809300274	0.000526377	3.94E-08	4	Zfp790
0.337550923	0.000536285	4.02E-08	4	Tlr2
0.472266775	0.000544397	4.08E-08	4	Sh2d2a
0.280262191	0.000544688	4.08E-08	4	Elmo1
0.373191635	0.000548618	4.11E-08	4	Fam86
0.397893561	0.000549099	4.11E-08	4	Mta3
0.49455447	0.000554765	4.15E-08	4	Ap2s1
0.532774159	0.000561837	4.21E-08	4	Paf1
0.296969346	0.000605863	4.54E-08	4	Rsl1d1
0.471162301	0.000618506	4.63E-08	4	Spast
0.567063594	0.00061965	4.64E-08	4	Wbp4
0.269100957	0.000628052	4.70E-08	4	Bloc1s1
0.434032136	0.000631717	4.73E-08	4	Pros1
0.618640599	0.00064	4.79E-08	4	Glt8d1
0.258780532	0.000642533	4.81E-08	4	Pds5a
0.331430393	0.000649579	4.87E-08	4	Nckap1l
0.816459247	0.000680123	5.09E-08	4	Zfp131
0.436676826	0.000681353	5.10E-08	4	Wrn
0.276433777	0.000681655	5.11E-08	4	Tnfsf9
0.742598502	0.000689705	5.17E-08	4	Rbm15
0.438241128	0.000700465	5.25E-08	4	Usp5
0.426510359	0.000708822	5.31E-08	4	Hspa9
0.307160577	0.000709818	5.32E-08	4	2810474O19Rik
0.370240928	0.000711747	5.33E-08	4	Ube2k
0.829194351	0.000720911	5.40E-08	4	Slc16a3
-0.54869862	0.000743682	5.57E-08	4	Klf6
0.33986559	0.00076971	5.76E-08	4	Lmna
0.302003373	0.000769984	5.77E-08	4	Ccdc101
0.289364642	0.000775164	5.81E-08	4	Uqcrc1
0.958105037	0.000787447	5.90E-08	4	Insl6
0.290687388	0.000806971	6.04E-08	4	Mapre1
0.71884214	0.000825403	6.18E-08	4	Nup50
0.646023675	0.000827561	6.20E-08	4	Osgin1
-0.845304243	0.000855666	6.41E-08	4	Eif4a1
0.459665885	0.000869568	6.51E-08	4	2810428I15Rik
0.343503691	0.000896903	6.72E-08	4	Diablo
0.505332298	0.000897306	6.72E-08	4	Fhod1
-1.569293532	0.000901461	6.75E-08	4	Thbs1
0.265271022	0.000910656	6.82E-08	4	Entpd5
0.384241472	0.000910783	6.82E-08	4	Ngrn
0.255504161	0.000914095	6.85E-08	4	Lhfpl2
0.373490869	0.000914188	6.85E-08	4	AF251705
0.679519891	0.000917828	6.87E-08	4	Pibf1
0.471142662	0.000942565	7.06E-08	4	Isg20
-0.328277827	0.000982368	7.36E-08	4	Cfl1
0.931069309	0.000998738	7.48E-08	4	Hes1

0.471468546	0.001009847	7.56E-08	4	Rbm28
0.365193277	0.001014751	7.60E-08	4	Trim27
0.465769512	0.001026582	7.69E-08	4	Arcn1
0.288241779	0.001035435	7.75E-08	4	Mdp1
-0.657913034	0.001046971	7.84E-08	4	Rpl3
0.2691134	0.001052009	7.88E-08	4	Stt3b
0.406065188	0.001057731	7.92E-08	4	Me2
-0.500885119	0.001066067	7.98E-08	4	Rpl18a
0.273098197	0.001084845	8.12E-08	4	Ddx27
0.455166711	0.001088073	8.15E-08	4	Psmg2
-1.235839601	0.001096167	8.21E-08	4	Hmgb2
0.383229326	0.001101443	8.25E-08	4	Aamdc
0.531416538	0.001124862	8.42E-08	4	Dmxl2
0.473755979	0.001129173	8.46E-08	4	Timm50
0.268037727	0.001134759	8.50E-08	4	Tmbim4
0.322750352	0.001135249	8.50E-08	4	Pgd
0.482883037	0.001148668	8.60E-08	4	Golph3l
0.53150594	0.001164689	8.72E-08	4	Pqlc2
0.401099186	0.001170828	8.77E-08	4	Coa7
0.380321891	0.001198183	8.97E-08	4	Uchl5
0.669146655	0.001211003	9.07E-08	4	Ccdc22
0.41324413	0.001252525	9.38E-08	4	Pnkp
0.270143378	0.001271169	9.52E-08	4	Map3k3
0.604055039	0.001271198	9.52E-08	4	Stag2
0.304036834	0.001284501	9.62E-08	4	Rbm14
0.269128665	0.001307354	9.79E-08	4	Pmm1
0.316011178	0.001310134	9.81E-08	4	Mtmr6
0.293891791	0.00133415	9.99E-08	4	Sel1l
0.315074828	0.001347557	1.01E-07	4	Srrm2
0.348621771	0.001437982	1.08E-07	4	Grap
0.308857718	0.001444913	1.08E-07	4	Sergef
0.936730251	0.001471826	1.10E-07	4	Ampd2
0.80896052	0.001495381	1.12E-07	4	Mtif3
0.33162782	0.00149925	1.12E-07	4	Emilin2
0.441924564	0.001515759	1.14E-07	4	Cops4
-0.555044796	0.001548328	1.16E-07	4	Serinc3
0.475187853	0.001549456	1.16E-07	4	Abl2
0.33412943	0.001580052	1.18E-07	4	Bud31
0.44546671	0.001583788	1.19E-07	4	Lsm1
0.602658825	0.001605183	1.20E-07	4	Bcas2
0.346089074	0.001606095	1.20E-07	4	Psmc3
0.561432933	0.001652242	1.24E-07	4	Nrd1
0.486303255	0.001675932	1.26E-07	4	H2-K2
0.491673407	0.0017414	1.30E-07	4	Mcur1
0.579482807	0.001743618	1.31E-07	4	Desi2
0.260616733	0.001762211	1.32E-07	4	Ak2
0.308483655	0.001781375	1.33E-07	4	Sh3bp5
0.347237994	0.001842014	1.38E-07	4	Gng2
0.300668836	0.001850662	1.39E-07	4	Fastkd2
0.283738779	0.001872329	1.40E-07	4	Dph3
0.498354778	0.001919287	1.44E-07	4	Mrpl11
0.327501878	0.001944728	1.46E-07	4	Nav1
0.286006825	0.001957175	1.47E-07	4	Kcnq1ot1
0.2777246	0.00196024	1.47E-07	4	Gatad2a
0.558458814	0.001984947	1.49E-07	4	Kmt2e
0.584355546	0.001993046	1.49E-07	4	Nt5dc3
0.270669108	0.002005517	1.50E-07	4	Pgls
0.467273718	0.00204949	1.53E-07	4	Usp9x
0.428310808	0.002086114	1.56E-07	4	Actr1b
0.552712489	0.002092498	1.57E-07	4	Slc5a3
0.65854901	0.002130784	1.60E-07	4	Csnk1g2

0.316324277	0.00216203	1.62E-07	4	Zfp710
0.261314978	0.002183707	1.64E-07	4	Mtpn
0.331237686	0.002219997	1.66E-07	4	Psmb6
-1.26676056	0.002234723	1.67E-07	4	Ppp4c
0.624681767	0.002239318	1.68E-07	4	Fam195a
0.256809923	0.002352052	1.76E-07	4	Ppm1g
0.729042612	0.002392493	1.79E-07	4	Zkscan6
0.51217115	0.002423521	1.82E-07	4	Ddx10
0.281821859	0.002507034	1.88E-07	4	Flna
0.329614715	0.00254309	1.90E-07	4	Stx7
0.371744633	0.002567928	1.92E-07	4	Lrrc75a
0.348201856	0.002576606	1.93E-07	4	Tspo
0.319894645	0.002650494	1.99E-07	4	Clptm1l
0.380284708	0.002663634	1.99E-07	4	Nsmce1
0.305544922	0.00267123	2.00E-07	4	Il2rg
0.355239147	0.002673416	2.00E-07	4	Nsfl1c
0.355275155	0.002808255	2.10E-07	4	Abcd1
0.394899228	0.002830032	2.12E-07	4	Bcap31
0.327189136	0.00285017	2.13E-07	4	Rrp1
0.313505078	0.002901109	2.17E-07	4	Hsbp1
0.561332711	0.002934359	2.20E-07	4	A630007B06Rik
1.014612246	0.002959439	2.22E-07	4	Rmdn3
0.685181779	0.003069608	2.30E-07	4	Stx11
0.497479045	0.003207364	2.40E-07	4	Txn2
0.56436112	0.003282024	2.46E-07	4	Adck4
0.28309028	0.003340332	2.50E-07	4	Commd4
0.792469012	0.003343307	2.50E-07	4	Mrpl43
0.268056842	0.003383477	2.53E-07	4	Gpbp1
0.467856235	0.003448161	2.58E-07	4	St3gal1
0.387057934	0.003463262	2.59E-07	4	Fbxo28
0.291357889	0.003473259	2.60E-07	4	Glud1
0.369233403	0.003528465	2.64E-07	4	Stk40
-0.410142348	0.003609822	2.70E-07	4	Gm6654
-1.002412537	0.003629532	2.72E-07	4	Uqcrb
0.273288233	0.003634752	2.72E-07	4	Tbc1d5
0.300438488	0.003664937	2.74E-07	4	Cttnbp2nl
0.419629806	0.003690196	2.76E-07	4	Dctn2
0.435276735	0.003709052	2.78E-07	4	Rps6ka3
0.314063826	0.003729894	2.79E-07	4	Ptpn1
0.387792071	0.003775647	2.83E-07	4	Ufl1
-1.15351616	0.003782132	2.83E-07	4	Zfp36l1
0.69984879	0.003782285	2.83E-07	4	Kctd13
0.294976369	0.003816831	2.86E-07	4	Gabpa
0.472495037	0.003858037	2.89E-07	4	Usp15
0.539065779	0.003921386	2.94E-07	4	Lancl2
0.550044476	0.003953796	2.96E-07	4	Elf4
0.286951349	0.003983858	2.98E-07	4	Zfp868
0.339046349	0.003985164	2.98E-07	4	Nucb1
0.33709674	0.00401159	3.00E-07	4	Dennd4a
0.616021501	0.004115901	3.08E-07	4	Gpr65
0.477235424	0.004197946	3.14E-07	4	Mapkap1
0.391823793	0.004297897	3.22E-07	4	Hbs1l
0.345464217	0.004348995	3.26E-07	4	Enpp1
0.402827045	0.004415365	3.31E-07	4	Ctla2a
0.462658995	0.004463122	3.34E-07	4	Eif4ebp3
0.294627754	0.004553302	3.41E-07	4	Cyp4f18
0.509924713	0.004575567	3.43E-07	4	Hbp1
0.491985779	0.004670328	3.50E-07	4	Shkbp1
0.279175536	0.004719411	3.53E-07	4	Plcb2
0.297173636	0.004745958	3.55E-07	4	Atp6v0e
0.76765367	0.004835392	3.62E-07	4	Ftsj3

-1.597502427	0.005014177	3.76E-07	4	Oas2
0.318919939	0.005101401	3.82E-07	4	Ddx21
0.436126886	0.005113786	3.83E-07	4	Mapk8
-1.367058707	0.005180136	3.88E-07	4	Fcgr2b
0.484275498	0.005199826	3.89E-07	4	Napsa
0.6299831	0.0052426	3.93E-07	4	Tnfaip2
0.542351198	0.005245409	3.93E-07	4	Txndc11
0.477447358	0.005269622	3.95E-07	4	Unc45a
0.737034655	0.005270378	3.95E-07	4	Hint3
0.395855041	0.005295445	3.97E-07	4	Hivep3
0.266981726	0.005296707	3.97E-07	4	Gpnmb
0.263099597	0.005447327	4.08E-07	4	Nt5dc1
0.424447555	0.005594936	4.19E-07	4	Ndufb9
-1.140097624	0.005603096	4.20E-07	4	Jun
0.373220382	0.005675953	4.25E-07	4	Glt25d1
0.251150591	0.005797888	4.34E-07	4	Gadd45b
-0.289735015	0.005846728	4.38E-07	4	Lyz2
0.54170037	0.005917527	4.43E-07	4	Dennd1c
0.260054117	0.005942569	4.45E-07	4	Nup98
0.339787254	0.00602744	4.51E-07	4	Sqstm1
0.556807016	0.006077206	4.55E-07	4	Tbcd
0.860778022	0.006117365	4.58E-07	4	Slc25a13
0.273828524	0.006164171	4.62E-07	4	Ufm1
0.336965723	0.006183697	4.63E-07	4	Ezr
0.335875726	0.006359469	4.76E-07	4	Aff1
0.339789709	0.006369173	4.77E-07	4	Rnf185
0.295577186	0.006428671	4.81E-07	4	Trp53inp2
0.409222447	0.006529915	4.89E-07	4	Vps33b
0.39755682	0.006701942	5.02E-07	4	Psmd3
0.882748911	0.006802238	5.09E-07	4	Map2k3os
0.254734673	0.006842804	5.12E-07	4	Chrac1
-0.536082353	0.006911818	5.18E-07	4	Rps10
0.5510248	0.006912824	5.18E-07	4	Dennd2c
0.369256273	0.006971305	5.22E-07	4	Wapal
0.474678438	0.007209913	5.40E-07	4	Tbc1d13
0.270471188	0.007251253	5.43E-07	4	Map2k4
-0.628743338	0.007715451	5.78E-07	4	Sepw1
0.459186086	0.007863502	5.89E-07	4	Ero1lb
0.252588697	0.008146132	6.10E-07	4	Apoa1bp
0.303192535	0.008239722	6.17E-07	4	Acadl
0.364870828	0.008256391	6.18E-07	4	Stra13
0.431889431	0.008356179	6.26E-07	4	Nudt8
0.571346986	0.008457646	6.33E-07	4	Bfar
0.805896787	0.008697619	6.51E-07	4	Nup155
0.619688664	0.008712925	6.53E-07	4	Bcl2l11
0.288651611	0.008723334	6.53E-07	4	Lsm6
0.290039246	0.008815999	6.60E-07	4	Riok3
0.301598597	0.009035641	6.77E-07	4	Glod4
0.251185399	0.009051424	6.78E-07	4	Sepn1
0.616317416	0.009066347	6.79E-07	4	Narg2
1.355630884	0.009074964	6.80E-07	4	Krit1
0.508801518	0.00921586	6.90E-07	4	Akap8
0.491762593	0.00934717	7.00E-07	4	Phf20
0.537288727	0.009378863	7.02E-07	4	Arhgap21
0.78033227	0.009459165	7.08E-07	4	Dhrs7b
0.308452187	0.009541909	7.15E-07	4	Tax1bp1
-1.316682265	0.009623022	7.21E-07	4	Rassf2
0.696037786	0.009663856	7.24E-07	4	Ccdc174
0.289165276	0.009703793	7.27E-07	4	Stat6
0.352538315	0.009945651	7.45E-07	4	Lst1
0.562071108	0.009996521	7.49E-07	4	Acadvl

0.498741312	0.010061038	7.54E-07	4	Tpm1
-0.277966228	0.010116435	7.58E-07	4	Mir682
0.318126524	0.010143116	7.60E-07	4	Fam53b
0.340588231	0.010204647	7.64E-07	4	Lats2
0.48725732	0.010360966	7.76E-07	4	Mybpc3
0.671923544	0.010410195	7.80E-07	4	Mbip
0.568829093	0.010643148	7.97E-07	4	Lonp2
0.618813719	0.011214313	8.40E-07	4	Trip4
0.25574876	0.011905143	8.92E-07	4	Rbbp7
0.303082209	0.012100515	9.06E-07	4	Rnasek
0.427067762	0.012134624	9.09E-07	4	Plekha3
0.582481913	0.012350309	9.25E-07	4	Senp6
0.284784743	0.012521858	9.38E-07	4	Smc3
0.494628527	0.012806414	9.59E-07	4	Sssca1
0.353316176	0.013603288	1.02E-06	4	Fut11
0.439469056	0.013904313	1.04E-06	4	Rell1
0.30686202	0.013936418	1.04E-06	4	Cd209a
0.334336666	0.014160481	1.06E-06	4	Taldo1
-0.433841035	0.014804896	1.11E-06	4	Tpm3
0.379404703	0.014934431	1.12E-06	4	Zfp180
0.292352507	0.015143704	1.13E-06	4	Plagl2
0.565867618	0.015342436	1.15E-06	4	Lars
0.405214595	0.015384175	1.15E-06	4	Snx14
0.489662103	0.015441727	1.16E-06	4	Brpf1
0.685225915	0.015610125	1.17E-06	4	Ddhd2
0.292719347	0.015826681	1.19E-06	4	Gtf2h5
0.273119499	0.016370738	1.23E-06	4	Vps28
0.269852528	0.016855983	1.26E-06	4	Mrpl39
-0.955328771	0.016896208	1.27E-06	4	Ddx39b
0.277967987	0.017205992	1.29E-06	4	Mprip
0.42342008	0.01731122	1.30E-06	4	Arfgap3
0.659308834	0.017442402	1.31E-06	4	Actr8
0.310341098	0.017588612	1.32E-06	4	Krtcap2
0.307008393	0.017906535	1.34E-06	4	S100a6
0.255247919	0.01829252	1.37E-06	4	Emr1
0.283256563	0.018337059	1.37E-06	4	Zfand3
0.424339091	0.018618194	1.39E-06	4	Pex2
0.632702831	0.018910807	1.42E-06	4	Polr3d
0.611072015	0.018993822	1.42E-06	4	Fmnl3
0.730406576	0.019036698	1.43E-06	4	1810011H11Rik
0.315410557	0.019196208	1.44E-06	4	Actr1a
0.462265626	0.019624188	1.47E-06	4	Tuba1a
0.597664974	0.019659509	1.47E-06	4	Mmd
-0.579545574	0.019754052	1.48E-06	4	Rpl6
0.266007071	0.019792101	1.48E-06	4	Mdh2
0.526580991	0.020048898	1.50E-06	4	Impa1
-0.274943393	0.020111113	1.51E-06	4	Itm2b
-1.906908409	0.020388229	1.53E-06	4	Cd163
0.426373224	0.020753976	1.55E-06	4	Dusp3
0.391954064	0.021806244	1.63E-06	4	Srp14
0.308377523	0.022128957	1.66E-06	4	Aar2
-1.510410354	0.022216255	1.66E-06	4	Chil3
0.258490266	0.022224434	1.66E-06	4	Tusc2
0.332662504	0.022585559	1.69E-06	4	Ankrd13c
-1.034789829	0.022916444	1.72E-06	4	Ptges3
0.252389501	0.023285113	1.74E-06	4	Smc1a
0.308531212	0.023364175	1.75E-06	4	Dusp2
0.568537021	0.023957591	1.79E-06	4	Tmed3
0.397803458	0.024452233	1.83E-06	4	Serpinb6a
-1.547036107	0.025044645	1.88E-06	4	Sowahc
-1.200318097	0.025540949	1.91E-06	4	Cmas

0.554842509	0.025750143	1.93E-06	4	Git2
0.411228884	0.027191108	2.04E-06	4	Asxl1
0.352436278	0.027696181	2.07E-06	4	Tm2d3
0.25393117	0.027956285	2.09E-06	4	Fcrls
0.29890806	0.027975659	2.10E-06	4	Lnpep
0.272867821	0.028129332	2.11E-06	4	Dpcd
0.806507266	0.028245927	2.12E-06	4	Dgka
-0.400719382	0.028289122	2.12E-06	4	Lgmn
0.463889992	0.028407063	2.13E-06	4	Mettl14
0.318300873	0.029289271	2.19E-06	4	Fam195b
0.252892987	0.02935139	2.20E-06	4	Iws1
0.345020759	0.030044611	2.25E-06	4	Dis3l
0.365423	0.030237786	2.26E-06	4	Cstf3
0.709696092	0.030981376	2.32E-06	4	Tsr3
0.358386441	0.031153253	2.33E-06	4	Mphosph8
0.419735803	0.031616791	2.37E-06	4	Tnip2
0.319369037	0.031697764	2.37E-06	4	Rgl2
0.383133438	0.032196468	2.41E-06	4	Acap2
0.537097809	0.03239658	2.43E-06	4	1600002K03Rik
0.275368764	0.03243967	2.43E-06	4	Dennd1b
0.274221453	0.03324063	2.49E-06	4	Aup1
0.369122131	0.033287703	2.49E-06	4	2810004N23Rik
-0.625527713	0.034137711	2.56E-06	4	Ly6e
0.475808665	0.034645357	2.59E-06	4	Atpaf2
-1.099291123	0.037070381	2.78E-06	4	Ssfa2
0.258326218	0.037274885	2.79E-06	4	Tmem256
0.322784617	0.037489751	2.81E-06	4	Nkiras2
0.386506931	0.037804495	2.83E-06	4	Epb4.1
-0.271938761	0.037813576	2.83E-06	4	NP-904328.1
0.873975894	0.03792522	2.84E-06	4	Pex11b
0.264747246	0.038279285	2.87E-06	4	Stk4
0.383150256	0.039419247	2.95E-06	4	Dync1li1
0.464765836	0.039571957	2.96E-06	4	Ccdc59
0.260712265	0.040013253	3.00E-06	4	Ier3ip1
0.559823612	0.040304165	3.02E-06	4	Gas2l1
0.731660986	0.040392082	3.03E-06	4	Pdxdc1
0.32150496	0.040620255	3.04E-06	4	Rab11a
0.260247777	0.041121883	3.08E-06	4	Pfdn1
0.30287171	0.042557968	3.19E-06	4	Chmp2a
0.256540148	0.042620378	3.19E-06	4	Osgep
0.270998231	0.043002869	3.22E-06	4	Agap1
0.28189575	0.043063846	3.23E-06	4	Nars
0.270321012	0.043265684	3.24E-06	4	0610037L13Rik
0.359981185	0.043349893	3.25E-06	4	Ptpn12
0.617187004	0.043485264	3.26E-06	4	Cyp4f13
0.271756787	0.043596735	3.27E-06	4	Cox19
-0.656107351	0.044134602	3.31E-06	4	Rpl35a
-1.871113124	0.044529116	3.34E-06	4	F13a1
0.290378039	0.045374754	3.40E-06	4	Slc39a7
0.312263162	0.046210088	3.46E-06	4	Mob3a
0.505175161	0.046614091	3.49E-06	4	Apoc4
0.529663236	0.046943	3.52E-06	4	Mms19
0.325025973	0.047313147	3.54E-06	4	Foxk1
0.463262365	0.048551233	3.64E-06	4	Gabarapl1
0.621955542	0.05045534	3.78E-06	4	Ate1
0.325035466	0.051266444	3.84E-06	4	Csnk1e
0.258144013	0.051587235	3.86E-06	4	Creg1
0.477648035	0.052093053	3.90E-06	4	Elmsan1
0.969272443	0.052120059	3.90E-06	4	Smpdl3b
-1.405066014	0.05280626	3.95E-06	4	Acat1
-1.012730969	0.052860417	3.96E-06	4	Tmed2

-1.118896517	0.052862174	3.96E-06	4	Mmp19
0.46979936	0.05315845	3.98E-06	4	Hsd17b10
0.577281778	0.053447326	4.00E-06	4	Slc4a2
0.361666312	0.053645359	4.02E-06	4	Uqcr10
0.470746562	0.054457929	4.08E-06	4	Slamf7
0.453687961	0.055824267	4.18E-06	4	Ercc1
-1.549329621	0.056741126	4.25E-06	4	Cmah
0.63324469	0.057635497	4.32E-06	4	Fam120b
0.25731636	0.060818714	4.56E-06	4	Rtn4
0.259665269	0.063414669	4.75E-06	4	Arl4a
0.257481508	0.063836176	4.78E-06	4	Snx15
0.285899184	0.065259905	4.89E-06	4	Slc1a5
0.275244265	0.065560552	4.91E-06	4	Bcl2l13
0.579449313	0.068586944	5.14E-06	4	Cd22
0.283726999	0.069058943	5.17E-06	4	Tnip3
-0.279743503	0.069582527	5.21E-06	4	Mrps15
0.268201965	0.069753241	5.22E-06	4	Ndufs8
-0.25560954	0.070075425	5.25E-06	4	Rpl18
0.862950367	0.071262739	5.34E-06	4	Faf1
0.520911425	0.07134637	5.34E-06	4	Gmps
0.327856043	0.071622876	5.36E-06	4	Rab10os
0.288192221	0.071717156	5.37E-06	4	Ssu72
0.321911269	0.072253777	5.41E-06	4	Sms
-0.609990213	0.073863575	5.53E-06	4	Rps15a
0.299108781	0.07425053	5.56E-06	4	Zwint
0.36927467	0.074420621	5.57E-06	4	Acvrl1
0.503236179	0.076228111	5.71E-06	4	Surf1
0.429640133	0.076797462	5.75E-06	4	Rgs12
0.354495653	0.078499624	5.88E-06	4	Cmklr1
0.626518519	0.08331367	6.24E-06	4	Manea
0.257723817	0.08374077	6.27E-06	4	Smpdl3a
-1.155464035	0.085662571	6.42E-06	4	Ncstn
0.611430462	0.086497538	6.48E-06	4	Xrn1
1.070936783	0.088174791	6.60E-06	4	Pip5k1a
0.310844519	0.090841079	6.80E-06	4	Rpl31
0.445138097	0.092627118	6.94E-06	4	Noa1
0.271777651	0.097267505	7.28E-06	4	Ppp2r4
0.441168276	0.097664824	7.31E-06	4	2210013O21Rik
0.315882134	0.098040577	7.34E-06	4	Grap2
-0.614958274	0.099761751	7.47E-06	4	Cebpa
0.419128731	0.101276276	7.59E-06	4	Nop14
0.272537482	0.1034169	7.75E-06	4	Avl9
0.65050323	0.103587353	7.76E-06	4	Sap30bp
0.693579573	0.103750157	7.77E-06	4	Lztr1
0.312025729	0.104006903	7.79E-06	4	Vezf1
0.306753976	0.104240019	7.81E-06	4	Efcab14
0.260457834	0.107406956	8.04E-06	4	Sec62
0.314131905	0.110908513	8.31E-06	4	Dr1
0.674711808	0.111419541	8.34E-06	4	Tecpr1
0.278910228	0.112619587	8.43E-06	4	Cox6a1
0.323906981	0.117649684	8.81E-06	4	Incenp
0.255582283	0.122176641	9.15E-06	4	Rab10
0.75290592	0.125231529	9.38E-06	4	Ccs
0.364026422	0.127023585	9.51E-06	4	Vasp
1.535674939	0.130908603	9.80E-06	4	Ccdc28a
1.176434307	0.131365878	9.84E-06	4	Cyb561d2
0.527037716	0.133483401	1.00E-05	4	Adam15
0.518362512	0.135481084	1.01E-05	4	Tpst2
0.452613418	0.136535216	1.02E-05	4	Guf1
0.67455474	0.138179358	1.03E-05	4	Ipo4
0.713207966	0.13971936	1.05E-05	4	Tyk2

0.383584971	0.142178875	1.06E-05	4	Pofut2
-0.322480293	0.142313335	1.07E-05	4	C1qc
-0.713680924	0.14284744	1.07E-05	4	Rps25
0.260889068	0.144921023	1.09E-05	4	Pon2
0.27975256	0.150643742	1.13E-05	4	Qars
0.665309804	0.152082645	1.14E-05	4	Vps51
0.368879553	0.154703184	1.16E-05	4	Vapb
0.616258196	0.154752842	1.16E-05	4	Mef2d
0.433831956	0.157503797	1.18E-05	4	Gfm1
-0.847368359	0.160812347	1.20E-05	4	Rps16
0.287323574	0.165809709	1.24E-05	4	Gpatch2l
0.569714432	0.166270075	1.25E-05	4	Zfp68
0.522989003	0.166654812	1.25E-05	4	Pdrg1
-1.174240397	0.17037653	1.28E-05	4	Ewsr1
-1.464303326	0.170457423	1.28E-05	4	Slfn5
0.253888201	0.182341255	1.37E-05	4	Fgr
0.345304648	0.18259045	1.37E-05	4	Cinp
1.047236269	0.182621036	1.37E-05	4	6430548M08Rik
0.491232511	0.186432379	1.40E-05	4	Abcf1
0.31345329	0.193920171	1.45E-05	4	Zmat5
0.702707309	0.195300459	1.46E-05	4	Speg
0.549049446	0.202863672	1.52E-05	4	1700112E06Rik
0.333316304	0.203699629	1.53E-05	4	2010107E04Rik
0.262181879	0.204895802	1.53E-05	4	D1Ertd622e
0.257475336	0.208994954	1.57E-05	4	Zc3h7a
0.272321237	0.211831745	1.59E-05	4	Npy
0.250827216	0.212947011	1.59E-05	4	Cox8a
0.425422382	0.215743731	1.62E-05	4	Snrpb2
0.276308924	0.219280304	1.64E-05	4	Orai1
0.738476484	0.219835115	1.65E-05	4	Zak
0.965104353	0.221312581	1.66E-05	4	Cd244
0.268665594	0.222542539	1.67E-05	4	Ggh
-0.351379231	0.22463056	1.68E-05	4	Ccnd1
0.631717124	0.225270075	1.69E-05	4	Brap
0.341181982	0.225648117	1.69E-05	4	Gtf3a
0.270846157	0.234074376	1.75E-05	4	Ehbp1l1
-1.219140404	0.234808619	1.76E-05	4	Ifi204
-0.458883044	0.23524219	1.76E-05	4	Mir703
0.270737194	0.237399002	1.78E-05	4	Ssh2
-1.158395569	0.239581506	1.79E-05	4	Gpr183
0.429045949	0.239755568	1.80E-05	4	Pitpnb
0.507557363	0.245966919	1.84E-05	4	Eif4ebp1
0.615319151	0.247304921	1.85E-05	4	Tmco4
0.328953559	0.250569128	1.88E-05	4	Txndc9
0.340987183	0.254375711	1.91E-05	4	Stam
0.389305187	0.254795076	1.91E-05	4	Nf2
0.255741911	0.254964444	1.91E-05	4	Scamp4
0.314788723	0.260076435	1.95E-05	4	Ccl5
0.752145962	0.262077771	1.96E-05	4	Ldlrad4
-0.529975683	0.266260703	1.99E-05	4	Rps19
0.30704151	0.267577771	2.00E-05	4	Ube3b
0.443978913	0.272136346	2.04E-05	4	Cgrrf1
-1.464738138	0.273373654	2.05E-05	4	Tbc1d4
0.611806548	0.275023656	2.06E-05	4	Agpat6
-1.106412905	0.279923222	2.10E-05	4	Hmox1
0.259773431	0.284440393	2.13E-05	4	Tmem183a
-1.151437946	0.288126498	2.16E-05	4	Abi3
0.682988907	0.294398754	2.20E-05	4	Xpnpep1
1.084600222	0.296233721	2.22E-05	4	Taf1
-0.485009629	0.296892316	2.22E-05	4	Rps7
0.604352243	0.297039575	2.22E-05	4	Ebi3

0.455261202	0.301818553	2.26E-05	4	Ggnbp2
0.323227366	0.305301758	2.29E-05	4	Pogz
0.334232861	0.312101198	2.34E-05	4	2310009A05Rik
0.34137043	0.316347142	2.37E-05	4	Fam3a
0.311724092	0.325694797	2.44E-05	4	Brd4
0.510500326	0.336206021	2.52E-05	4	Clip1
0.436830872	0.338613178	2.54E-05	4	Kif1c
0.291900972	0.344749622	2.58E-05	4	Emc10
0.256369215	0.349944143	2.62E-05	4	Hdlbp
1.20666069	0.350621272	2.63E-05	4	Il18bp
0.309964996	0.358162941	2.68E-05	4	Mrpl37
0.329829941	0.363054859	2.72E-05	4	Med13
-0.788456641	0.377025505	2.82E-05	4	Btf3
0.255941724	0.384455725	2.88E-05	4	Atxn1
-1.013271773	0.386462174	2.89E-05	4	Atxn2
0.280899729	0.40022868	3.00E-05	4	Wars
-1.07014412	0.404079438	3.03E-05	4	Hnrnpl
-1.519768516	0.414315524	3.10E-05	4	Fgl2
0.323922717	0.415456287	3.11E-05	4	Cry1
0.308196492	0.425155917	3.18E-05	4	Pea15a
0.305471109	0.42629281	3.19E-05	4	Ubl5
-1.358250655	0.457776926	3.43E-05	4	Rnasel
0.489413148	0.461467601	3.46E-05	4	Fbxo3
1.288556012	0.472500791	3.54E-05	4	Cdadc1
0.496127296	0.498649244	3.73E-05	4	Bhlhe41
1.010175141	0.508878175	3.81E-05	4	Supv3l1
0.308178701	0.512687219	3.84E-05	4	Ccdc47
0.458442688	0.522130415	3.91E-05	4	Yeats2
0.339262689	0.53083359	3.98E-05	4	Ube3c
0.25709405	0.53155096	3.98E-05	4	Tubg1
0.265884329	0.537051949	4.02E-05	4	Rnh1
0.281792253	0.543859661	4.07E-05	4	Camsap2
-0.686185098	0.545196758	4.08E-05	4	Matk
0.432917128	0.547557611	4.10E-05	4	Srxn1
0.356311471	0.569942574	4.27E-05	4	Xylt2
-0.805688609	0.584718611	4.38E-05	4	Aftph
0.254206751	0.617109038	4.62E-05	4	1700017B05Rik
0.473613275	0.621302064	4.65E-05	4	AU040320
-1.226317763	0.63585865	4.76E-05	4	Safb
0.631358925	0.645160215	4.83E-05	4	Klrb1b
-0.700389231	0.677715627	5.08E-05	4	Atp5l
0.477396082	0.679495499	5.09E-05	4	Polb
-0.704668731	0.682023824	5.11E-05	4	Ywhah
1.061360162	0.699134259	5.24E-05	4	Glcci1
-0.993622807	0.73338645	5.49E-05	4	Atp5k
0.294113384	0.755832564	5.66E-05	4	Sdhb
-0.382775557	0.761655979	5.70E-05	4	Gm4832
0.356505326	0.762999886	5.71E-05	4	Mylip
-0.542608524	0.767782412	5.75E-05	4	Ifitm2
0.433822646	0.789507086	5.91E-05	4	Tulp4
0.310111689	0.806665203	6.04E-05	4	Ttc33
0.360858461	0.807259917	6.05E-05	4	Atm
0.293487108	0.819677688	6.14E-05	4	Gstm1
0.332579118	0.822230618	6.16E-05	4	Fas
0.619963805	0.826547162	6.19E-05	4	Trak2
-1.118760078	0.827546494	6.20E-05	4	Dcaf8
0.399962488	0.835821292	6.26E-05	4	Irs2
0.353692472	0.857507486	6.42E-05	4	Lyar
0.28694356	0.867136111	6.49E-05	4	Hmgxb3
0.308226504	0.868353964	6.50E-05	4	Ubn1
0.270439173	0.881126985	6.60E-05	4	Uqcrq

0.340404478	0.881398971	6.60E-05	4	Tbcc
0.373250291	0.944078445	7.07E-05	4	Ncor1
0.961273674	0.945577604	7.08E-05	4	Ano10
0.407215223	0.971965697	7.28E-05	4	Plekha2
-1.012495074	0.973679599	7.29E-05	4	Ctnnb1
0.276969116	0.977580062	7.32E-05	4	Med6
-0.399261003	0.979393968	7.34E-05	4	Gm6548
0.381801702	0.985266613	7.38E-05	4	Gimap6
0.438219331	1	7.52E-05	4	Srebf1
0.284937538	1	7.55E-05	4	Gclm
0.295529414	1	7.82E-05	4	Pilrb1
0.252480447	1	7.88E-05	4	Degs1
0.418361292	1	8.11E-05	4	Rora
0.314661528	1	8.42E-05	4	Ppp1r15b
0.419782186	1	8.49E-05	4	Tomm34
0.265058287	1	8.85E-05	4	Gsdmd
0.32098781	1	8.88E-05	4	Psen1
0.317646897	1	8.95E-05	4	Adrbk1
-1.419765917	1	9.05E-05	4	Rcn3
0.570880026	1	9.06E-05	4	Stxbp6
-0.587170493	1	9.13E-05	4	Sepp1
0.29843746	1	9.60E-05	4	Arl11
0.337357886	1	9.76E-05	4	Aimp1
-0.359978979	1	9.79E-05	4	Sap30
0.330558664	1	9.97E-05	4	2310039H08Rik
1.43970006	1	0.0001013	4	Slc25a38
0.318561775	1	0.0001019	4	Mafg
0.529159074	1	0.0001049	4	Tppp3
-1.433554051	1	0.0001061	4	Ccl7
0.406015271	1	0.0001072	4	Ctbp2
0.323866635	1	0.0001075	4	Ocrl
-1.058462992	1	0.0001091	4	Golga2
0.44215311	1	0.000111	4	Uspl1
0.455258766	1	0.000112	4	Shc1
0.387969342	1	0.0001146	4	Pomt1
0.368774315	1	0.0001184	4	Crebbp
0.607295099	1	0.0001185	4	Hspa13
0.726703212	1	0.0001193	4	Arl13b
0.369387308	1	0.0001195	4	Alkbh1
0.274855172	1	0.0001248	4	Mga
0.298493843	1	0.0001287	4	Psmb4
-0.495889279	1	0.0001288	4	Rps8
0.259713007	1	0.0001304	4	Hint1
0.602734835	1	0.000131	4	Myo7a
0.272451571	1	0.0001313	4	Trmt1
0.289998652	1	0.0001326	4	Ndufa9
-1.221404831	1	0.0001337	4	Wdfy3
0.325746626	1	0.0001343	4	Abhd17c
-0.743671832	1	0.0001361	4	Ndufb4
0.367418789	1	0.000138	4	Trim26
-0.666276063	1	0.0001403	4	Mir684-1
0.549482086	1	0.0001417	4	Synj2bp
0.432263719	1	0.0001422	4	Taf15
0.560430985	1	0.0001433	4	Madd
-0.862019961	1	0.0001455	4	Gmfg
0.291794162	1	0.0001462	4	Tmem164
0.455916508	1	0.0001469	4	Cdc40
0.303693725	1	0.000148	4	Maea
0.589757794	1	0.0001535	4	Lrrc8a
0.513251083	1	0.000154	4	1810026J23Rik
0.526877307	1	0.000161	4	Gse1

-1.422800621	1	0.0001614	4	Stard8
0.454287293	1	0.0001616	4	Hgs
-1.12583559	1	0.0001654	4	Nqo2
-0.571373918	1	0.0001655	4	Prcp
0.355253319	1	0.0001719	4	Nupr1
-1.072565267	1	0.0001761	4	Sft2d1
-0.28944071	1	0.0001829	4	Crip1
0.322717962	1	0.000186	4	BC028528
0.953115098	1	0.0001879	4	Tjp2
-1.173432447	1	0.0001893	4	Nfil3
-1.385678182	1	0.0001902	4	Gda
-0.943583961	1	0.0001915	4	Lman1
0.776181437	1	0.0001969	4	Zpr1
-0.255753102	1	0.0002023	4	Dusp22
-1.084736734	1	0.0002028	4	Nkap
0.420199428	1	0.0002036	4	Ap1m1
0.29880067	1	0.0002053	4	Klhdc3
0.269917266	1	0.0002058	4	Cdc37l1
-0.364198653	1	0.0002065	4	Shfm1
-0.276570328	1	0.0002091	4	Fam98c
0.457799819	1	0.0002102	4	Bcl2l1
0.287937468	1	0.0002123	4	Nod1
0.34302475	1	0.0002141	4	Adap1
-1.061097709	1	0.0002203	4	Adap2
0.567280775	1	0.0002225	4	Fhl3
0.250521136	1	0.0002254	4	Syk
0.349194827	1	0.0002257	4	Ube2j1
0.439705016	1	0.0002267	4	Samd8
0.347887117	1	0.0002296	4	Spns1
-1.192890711	1	0.0002397	4	Mfsd7b
0.301174873	1	0.0002416	4	Coq10a
0.323113124	1	0.0002445	4	Trem1
-0.727050149	1	0.0002528	4	Gnb1
-0.983032565	1	0.0002534	4	Khdrbs1
0.353140419	1	0.0002545	4	Fbxo32
0.414041582	1	0.0002575	4	Rps6kb1
-0.423983564	1	0.0002594	4	Rps2
0.274904258	1	0.0002621	4	Lcmt1
0.581808181	1	0.0002675	4	Xrcc6
-0.942919091	1	0.0002727	4	Cox7c
0.61628857	1	0.0002782	4	Vhl
0.346663759	1	0.0002801	4	Aldh3b1
1.02742208	1	0.0002816	4	Ago3
-1.212299472	1	0.0002817	4	Vrk2
0.311256579	1	0.0002817	4	Eif3i
-1.011914418	1	0.0002948	4	Arf5
0.31242183	1	0.0003004	4	Rpl7
-1.065355182	1	0.0003046	4	Abl1
0.351670902	1	0.0003068	4	Cdk5
-1.008923966	1	0.0003083	4	Rsrp1
0.332956126	1	0.0003112	4	Ssr1
0.262408676	1	0.0003171	4	Rnf187
0.880974721	1	0.0003195	4	Plxdc2
0.413514254	1	0.0003232	4	Kif2a
0.422289539	1	0.0003249	4	Emc9
0.50200564	1	0.0003385	4	5830432E09Rik
-1.095846769	1	0.0003448	4	Vcam1
0.371120384	1	0.0003501	4	Parvb
0.390116436	1	0.0003604	4	Aph1a
-0.967887369	1	0.0003676	4	Ccl9
-1.089487564	1	0.0003757	4	Chuk

-1.363335844	1	0.0003792	4	Gsr
0.355671473	1	0.0003865	4	Cd84
0.443970729	1	0.0003911	4	Cdk6
0.403840569	1	0.0003924	4	Ercc3
0.534085648	1	0.0003973	4	Ehmt1
-0.275957771	1	0.000403	4	Nostrin
-0.710151401	1	0.0004061	4	Tpt1
0.260962455	1	0.0004115	4	Slfn8
0.250669757	1	0.0004139	4	Fam172a
-0.862790822	1	0.0004207	4	Atp5g1
0.3738736	1	0.0004216	4	Eif3f
-0.954623867	1	0.0004245	4	Zfp703
0.293448951	1	0.0004772	4	Ncoa5
-0.363704381	1	0.0004859	4	Cxcl10
0.575714741	1	0.0004874	4	Rassf3
0.364576614	1	0.0004922	4	Frmd8
-0.554090277	1	0.0005101	4	Arpc3
-0.438182219	1	0.000511	4	2900060B14Rik
0.318258907	1	0.0005329	4	Vdac1
0.661140766	1	0.0005526	4	Ing1
1.058245206	1	0.0005538	4	Ogfrl1
-0.488969079	1	0.0005799	4	Psmb7
-0.494965723	1	0.0005814	4	Btg1
0.411935728	1	0.0005834	4	Cdc73
-0.963648744	1	0.0005836	4	Rnf181
0.418216394	1	0.000597	4	Lpgat1
-0.262022123	1	0.0006015	4	Nipa2
-0.877216737	1	0.0006442	4	Rab3gap1
0.431880125	1	0.000647	4	Txndc5
-1.288690728	1	0.0006535	4	Adar
-0.482578865	1	0.0006573	4	Sf3b4
-0.33199376	1	0.0006637	4	Zzef1
0.415418282	1	0.0006718	4	Bbx
-1.118774756	1	0.0006904	4	Gpx3
0.344763524	1	0.0006972	4	Ubxn6
0.31148742	1	0.000709	4	Fam160b1
-0.362524167	1	0.000711	4	Gclc
-0.56631329	1	0.0007193	4	Pcbp2
0.457940705	1	0.0007199	4	Abhd11
0.258784819	1	0.000723	4	Eepd1
0.272537482	1	0.0007305	4	Sash1
0.307941369	1	0.0007314	4	Hcst
0.339697598	1	0.0007445	4	Copb1
0.493192655	1	0.0007562	4	Gpatch11
0.35419735	1	0.0007594	4	Commd9
0.666343288	1	0.0007624	4	Kdm5b
0.37373506	1	0.0007633	4	Deb1
0.469623288	1	0.0007672	4	Ubtd1
-0.750478687	1	0.0007891	4	Prrc2a
0.298518052	1	0.0007892	4	Pik3r1
-1.18420887	1	0.0007902	4	Zbtb4
-0.333830181	1	0.0007981	4	Clta
0.391259823	1	0.0008021	4	Irf5
0.71167344	1	0.0008127	4	Nkg7
0.441341064	1	0.0008133	4	Tmem140
0.432890274	1	0.0008197	4	2310045N01Rik
1.08922311	1	0.0008441	4	Gosr1
-0.471893226	1	0.0008857	4	Snx5
-1.061590029	1	0.0008967	4	Ccl2
-0.709266905	1	0.0009219	4	Rhob
-0.295692031	1	0.0009467	4	Txlna

-0.874721652	1	0.0009532	4	Psmb3
-0.278588688	1	0.0009559	4	Slc25a46
0.388904666	1	0.0009623	4	Smarcc2
-0.337366399	1	0.0009835	4	Psme2
-1.161814174	1	0.000997	4	Gbp7
-0.676906408	1	0.0010088	4	Kctd12
-0.319086269	1	0.0010161	4	Rpl37
-1.288066905	1	0.0010266	4	Foxj2
-1.116796829	1	0.0010397	4	Agps
-1.00610969	1	0.0010681	4	Gnb2
0.277104757	1	0.0010743	4	Rhbdd1
-1.209067059	1	0.0010793	4	Nfxl1
-1.016311651	1	0.0010851	4	Egr2
0.33013533	1	0.0011191	4	Il10
0.485895338	1	0.0011261	4	Zfp516
-0.308785941	1	0.0011288	4	Dok1
0.43229632	1	0.0011386	4	Mrpl36
-1.067070127	1	0.0011422	4	Nfu1
0.514845476	1	0.0011427	4	Larp1
-1.041881986	1	0.001161	4	Trps1
0.768259286	1	0.0011793	4	Atmin
-1.147775294	1	0.0011942	4	Snrpf
-0.448994268	1	0.0011991	4	Marcks
0.352263678	1	0.0012132	4	P2ry6
0.350251596	1	0.0012141	4	Vps52
0.433281667	1	0.0012262	4	Anapc2
0.312836	1	0.0012481	4	Kmt2b
0.257399067	1	0.0012563	4	Fzr1
0.382624283	1	0.0012717	4	Wdr77
0.976945869	1	0.0012917	4	Smim13
-0.359350818	1	0.0012924	4	Etfa
0.292175745	1	0.0013284	4	Myeov2
-0.379641741	1	0.0013329	4	Hexim1
0.261343335	1	0.0013509	4	Vcpip1
0.466846984	1	0.0013515	4	Bet1
0.707273569	1	0.0013628	4	Ampd3
-0.628579475	1	0.0013691	4	Atp5h
-1.186257421	1	0.001373	4	Pf4
0.254942217	1	0.0013759	4	Hras
0.621042891	1	0.001383	4	Zmym5
0.806667793	1	0.0013859	4	Taf5
-0.780123878	1	0.0014066	4	Sema4a
-0.327082055	1	0.0014251	4	Eno1b
-0.270349291	1	0.0014344	4	Pxdc1
0.473757035	1	0.0014492	4	Pkp4
0.375721172	1	0.0014589	4	Ppfibp2
-1.135685628	1	0.0014909	4	Birc2
-0.878366268	1	0.001505	4	Dtx3l
-0.856574461	1	0.0015489	4	Irf2bp2
-0.489202564	1	0.001565	4	Unc93b1
-0.268382713	1	0.0016243	4	Mon1a
0.285167341	1	0.0016431	4	Cbfa2t3
-0.405625553	1	0.0016843	4	Phax
-0.666080528	1	0.0017394	4	Set
-0.942911542	1	0.0017517	4	Sh3bp1
-0.2936935	1	0.0017564	4	Rnf20
0.399517914	1	0.0017614	4	Rad17
-0.252493367	1	0.001784	4	H3f3b
-0.914462833	1	0.001785	4	Ube2m
0.461449428	1	0.0017889	4	Gps2
0.254809379	1	0.0018378	4	Nt5dc2

0.571135664	1	0.001883	4	Scnm1
-1.190171564	1	0.0018855	4	Fgfr1
0.979576334	1	0.0019265	4	Srm
0.555792166	1	0.001931	4	Appl1
-0.271145611	1	0.0019335	4	Ip6k1
-0.459694127	1	0.0019495	4	Cd74
0.298513937	1	0.0019543	4	Aifm1
-0.953460848	1	0.0020329	4	Slc43a2
-0.774029028	1	0.0020399	4	Pcna
-0.593811911	1	0.0020814	4	Rpl36al
0.272288788	1	0.0020904	4	5031439G07Rik
-1.034927598	1	0.0020915	4	Ints6
-0.259566722	1	0.0021027	4	Spg7
-1.04454382	1	0.0021121	4	Osm
0.33130561	1	0.0021938	4	Nomo1
0.276039604	1	0.0022372	4	Vamp3
-0.251675211	1	0.0023126	4	Setd8
-0.722769686	1	0.0023187	4	Nr4a1
0.619812523	1	0.0023191	4	Rsbn1
-0.614989479	1	0.002366	4	Ccl6
0.299304316	1	0.0023845	4	Tbc1d22a
0.412821191	1	0.0023917	4	Lrwd1
-0.308834314	1	0.0024666	4	Calr
-0.305288062	1	0.0024797	4	Fut8
-0.581004564	1	0.00255	4	Slc29a1
0.418851131	1	0.0025645	4	1500011K16Rik
0.539579297	1	0.0026162	4	Herc1
-1.099957104	1	0.0026427	4	Arl8a
0.886172066	1	0.0026707	4	Pou2f1
-1.23744894	1	0.0026708	4	Wdfy4
-1.166338374	1	0.0026867	4	Pmaip1
-1.154237352	1	0.0027153	4	4833420G17Rik
0.348341917	1	0.002773	4	Slc25a44
0.39794316	1	0.0027819	4	Crip2
0.304615377	1	0.0028339	4	Pop7
-0.924784109	1	0.0028533	4	Dnajc19
-0.784274666	1	0.0028811	4	Dusp6
-0.313742478	1	0.0028836	4	5730416F02Rik
0.255600064	1	0.0030602	4	Poglut1
-0.296483863	1	0.0031915	4	Msl1
0.431393555	1	0.0032134	4	Def6
-0.348962193	1	0.003295	4	Jade2
0.348916145	1	0.0033348	4	Tcerg1
-1.084584976	1	0.0033563	4	Naaa
-0.331895227	1	0.0033804	4	AI837181
-0.561885417	1	0.0034199	4	Psenen
-1.046716146	1	0.0034342	4	Tfe3
0.416909031	1	0.0034468	4	Ptdss1
-1.141567766	1	0.0034493	4	Srgap2
0.637652151	1	0.0034514	4	Upf3a
-1.241928405	1	0.0035262	4	Irf7
0.253517484	1	0.0035382	4	Dhx8
-0.289567381	1	0.0035505	4	Tmsb10
0.259520905	1	0.0035959	4	Kif3a
0.370748014	1	0.0036125	4	Tmem192
-0.253100868	1	0.0036561	4	Pabpc1
-0.737655886	1	0.0036957	4	Chmp1a
0.448392853	1	0.0037016	4	Gpcpd1
-0.933131444	1	0.003725	4	Wipf2
0.316714463	1	0.0038339	4	Gm12942
-0.291010695	1	0.0038371	4	5430427O19Rik

0.49185678	1	0.0038693	4	Impad1
-0.250065209	1	0.0039469	4	Rnf44
0.558186195	1	0.0039562	4	Sik1
-0.497190313	1	0.0040369	4	Eps15l1
-0.399049119	1	0.0040512	4	Adap2
-1.0054896	1	0.0040619	4	Mfge8
-0.267807127	1	0.0040759	4	Fam219b
-0.25079655	1	0.0041631	4	1500012F01Rik
-0.556803454	1	0.0042589	4	Midn
0.562367558	1	0.0043052	4	Plod3
-1.270607408	1	0.0043236	4	Clec10a
-0.297943588	1	0.004351	4	Osgin2
-0.77826693	1	0.004365	4	Clec4n
-0.565664549	1	0.0045085	4	Csde1
-0.306681014	1	0.0045174	4	Med21
1.054017489	1	0.0045445	4	Rpp25l
-0.302391028	1	0.0045833	4	Atxn1l
-0.332537313	1	0.0046426	4	Umps
0.896624728	1	0.0046779	4	Zc3h4
1.146465314	1	0.0047224	4	Lsg1
-0.439520007	1	0.0047847	4	Rbm39
0.307566104	1	0.0047848	4	Ext2
-0.290334323	1	0.0047848	4	D17Wsu92e
0.861695167	1	0.0048269	4	Mcoln2
-0.620610634	1	0.0048419	4	Fli1
0.301118032	1	0.0049073	4	Map3k2
-0.899889586	1	0.0049245	4	Cmpk1
-0.587868847	1	0.0049567	4	Nufip2
-0.728380111	1	0.0052294	4	Hnrnpa1
-1.095543233	1	0.0052527	4	Zfhx3
-1.020963854	1	0.0053362	4	Ralgapa1
-0.266331769	1	0.0054254	4	Ptpro
-0.425114006	1	0.0055382	4	Cd40
-0.789349304	1	0.0057857	4	Sh3gl1
0.473416663	1	0.0059344	4	Sap18
-0.550701292	1	0.0059625	4	Snrpd2
-1.127663926	1	0.0059799	4	Arpc1a
-0.882706132	1	0.0059934	4	Psma5
-0.964766305	1	0.0060581	4	Cysltr1
-0.645470107	1	0.0061062	4	Purb
0.37796905	1	0.0062182	4	Nfam1
0.366857518	1	0.0062827	4	Ddx6
-0.9225254	1	0.0063776	4	Fn1
-1.041059905	1	0.0064045	4	Ino80d
0.472849366	1	0.0064103	4	Lin7c
-1.202014551	1	0.0064203	4	Olfm1
-0.957953278	1	0.0065836	4	C3
0.433303007	1	0.0066141	4	Eno3
0.47251014	1	0.0066361	4	Naca
-0.956355919	1	0.0066688	4	Vmp1
-0.897189763	1	0.0066893	4	Fyttd1
-0.991231841	1	0.006714	4	Tm9sf1
-0.272748016	1	0.0067581	4	Psd4
0.546227647	1	0.0067946	4	Dnlz
-0.506430767	1	0.0068216	4	Slc25a5
-0.77901014	1	0.0068802	4	1810022K09Rik
0.276542555	1	0.0068912	4	Rnf139
-0.803040511	1	0.0069102	4	Rab5c
-0.650764111	1	0.0070434	4	Tma7
-0.811134485	1	0.007122	4	Synrg
0.575561477	1	0.007412	4	Arap2

-0.396745991	1	0.0075038	4	App
-1.031789818	1	0.0075504	4	Mrpl3
0.253824059	1	0.0075878	4	Iqgap1
-1.102063353	1	0.0076823	4	Ang
-0.917540206	1	0.0077152	4	Taf4a
-1.033430425	1	0.007729	4	4932438A13Rik
-1.087273565	1	0.007854	4	Cited2
-0.320810252	1	0.0078673	4	Soga1
-0.400926245	1	0.0079333	4	Cd53
-0.294743395	1	0.0079514	4	Mtmr4
-0.354139514	1	0.0079557	4	Dusp1
0.413572563	1	0.0080131	4	Ap1g2
-0.863342394	1	0.0081339	4	Tmem165
0.27971767	1	0.0082101	4	Jade1
-0.274969237	1	0.0082756	4	C1qa
-0.300172029	1	0.0084849	4	Creb5
-0.379317238	1	0.0085546	4	Kansl1
-1.014371074	1	0.0085792	4	Tns1
-0.299316734	1	0.0090399	4	Nans
-0.685745825	1	0.0092465	4	Plxnd1
-1.234640081	1	0.009248	4	Oasl1
0.526273368	1	0.0094072	4	Elk3
0.317152179	1	0.0094677	4	Pcnt
0.378750083	1	0.0095408	4	Usp45
0.532087283	1	0.009588	4	Prelid1
-1.016755091	1	0.0095924	4	Rbm47
-0.255351177	1	0.0099675	4	Pbx3
-1.795984227	1.25E-32	9.39E-37	5	C1qc
-1.422824816	1.28E-32	9.62E-37	5	C1qb
-1.838456837	1.02E-29	7.64E-34	5	C1qa
0.971909889	8.84E-26	6.62E-30	5	Tmsb10
-1.976061496	1.43E-25	1.07E-29	5	C3ar1
-1.808998682	9.41E-24	7.05E-28	5	Ms4a7
-1.123672056	3.82E-23	2.86E-27	5	Lyz2
-1.741611547	5.48E-23	4.11E-27	5	Timp2
0.827199065	5.94E-22	4.45E-26	5	H2-Aa
1.57816466	1.04E-21	7.81E-26	5	H2-DMb2
-1.579720167	1.11E-21	8.35E-26	5	Sepp1
-1.827149374	2.27E-21	1.70E-25	5	Emr1
0.807122734	2.67E-21	2.00E-25	5	H2-Ab1
-0.734394316	1.06E-20	7.95E-25	5	Ctsb
-1.462752027	2.86E-20	2.14E-24	5	Fcgr3
0.833147018	4.03E-20	3.02E-24	5	H2-Eb1
0.771421076	7.27E-19	5.44E-23	5	Cd74
-1.704787192	7.38E-19	5.53E-23	5	Mafb
1.725883157	2.77E-18	2.07E-22	5	Spint2
-2.062918699	5.02E-18	3.76E-22	5	Igf1
**-1.182506725**	**5.13E-18**	**3.84E-22**	**5**	**Csf1r**
1.804269389	2.31E-17	1.73E-21	5	P2ry10
0.973711306	3.31E-17	2.48E-21	5	Rps19
1.435494185	3.45E-17	2.58E-21	5	Cytip
-1.643653273	1.38E-16	1.03E-20	5	Trem2
-1.402321552	3.08E-16	2.31E-20	5	Abca1
0.821004019	6.00E-16	4.49E-20	5	Lsp1
-1.939223106	1.85E-15	1.39E-19	5	Cd84
0.928317584	6.19E-15	4.64E-19	5	Sub1
-1.153956199	2.10E-14	1.57E-18	5	Dhrs3
-1.533146392	2.21E-14	1.65E-18	5	Ccl3
-0.91347448	4.49E-14	3.36E-18	5	Ctsd
0.52306473	5.21E-14	3.90E-18	5	Rpl41
2.013846527	1.29E-13	9.69E-18	5	H2-Eb2

-1.154514027	1.33E-13	9.96E-18	5	Cxcl2
-2.076124534	1.84E-13	1.38E-17	5	Serpinb6a
-1.764557565	2.63E-13	1.97E-17	5	C5ar1
0.577018035	3.06E-13	2.29E-17	5	Rps9
-1.150465938	4.64E-13	3.48E-17	5	Nrp1
-1.372497901	5.07E-13	3.80E-17	5	Stab1
0.478634936	5.21E-13	3.90E-17	5	Ifitm7
-0.751168878	6.56E-13	4.91E-17	5	Grn
0.466216388	8.60E-13	6.44E-17	5	Tmsb4x
-1.342529476	9.31E-13	6.97E-17	5	Tnf
1.774598393	1.07E-12	8.04E-17	5	Flt3
1.039999294	1.35E-12	1.01E-16	5	S100a11
-1.524741088	2.51E-12	1.88E-16	5	Tmem106a
-1.260676748	2.82E-12	2.11E-16	5	Rhob
-1.999727406	3.89E-12	2.91E-16	5	Pmp22
-0.993886513	7.61E-12	5.70E-16	5	Pltp
-1.441985794	7.69E-12	5.76E-16	5	Tgfbr2
-0.910432694	9.62E-12	7.21E-16	5	Fyb
-0.802729875	1.36E-11	1.02E-15	5	Cd63
-1.325200727	1.47E-11	1.10E-15	5	Glul
-1.07387063	2.95E-11	2.21E-15	5	5430435G22Rik
-1.521493596	3.44E-11	2.58E-15	5	Ecm1
-1.2240538	3.67E-11	2.75E-15	5	Zeb2
-1.844860094	3.85E-11	2.88E-15	5	Itgb5
-1.342082057	4.80E-11	3.60E-15	5	Cd81
-1.446035643	8.68E-11	6.50E-15	5	Pla2g7
-0.695782887	1.85E-10	1.38E-14	5	Lamp1
0.49856138	2.60E-10	1.94E-14	5	Rplp0
-1.554620261	3.33E-10	2.49E-14	5	Cd93
-1.87147633	3.65E-10	2.73E-14	5	Pf4
0.809714791	4.49E-10	3.36E-14	5	Rpl18
-1.198067507	6.21E-10	4.65E-14	5	4632428N05Rik
0.416443636	6.25E-10	4.68E-14	5	Rps14
1.009407112	8.60E-10	6.44E-14	5	Syngr2
-1.613452404	8.72E-10	6.53E-14	5	Slc11a1
-1.042045742	8.90E-10	6.66E-14	5	Lipa
-2.294686022	9.80E-10	7.34E-14	5	Folr2
0.608014948	1.03E-09	7.70E-14	5	Cd52
2.055183219	1.03E-09	7.72E-14	5	Btla
0.90551	1.16E-09	8.72E-14	5	Psme2
-0.758982773	1.22E-09	9.11E-14	5	Cd14
0.435254695	1.69E-09	1.27E-13	5	Mir682
0.607765996	1.74E-09	1.30E-13	5	Gm6654
0.501024835	2.24E-09	1.67E-13	5	Rps15
-1.636417548	2.44E-09	1.83E-13	5	Fcrls
-1.879491716	3.08E-09	2.31E-13	5	Rnase4
1.457052147	3.50E-09	2.62E-13	5	Jak2
-1.479230516	3.60E-09	2.70E-13	5	Nfic
0.562450764	3.61E-09	2.70E-13	5	Rpl14
0.694021179	3.81E-09	2.85E-13	5	Rpl6
-1.122542498	5.10E-09	3.82E-13	5	Ehd4
0.648383612	9.23E-09	6.92E-13	5	Rpl18a
0.556877361	1.48E-08	1.11E-12	5	Rpl14-ps1
0.612258173	1.56E-08	1.17E-12	5	Rps18
-1.272328069	1.73E-08	1.29E-12	5	Blvrb
0.498634385	2.28E-08	1.71E-12	5	Rps20
-0.887384361	2.35E-08	1.76E-12	5	AI607873
-1.086415171	2.45E-08	1.83E-12	5	Camk1
-1.354541877	3.19E-08	2.39E-12	5	Acp2
-1.400747874	3.51E-08	2.63E-12	5	Mrc1
-0.977763724	3.56E-08	2.67E-12	5	Ninj1

0.451508075	4.64E-08	3.48E-12	5	Gm1821
0.495599461	4.67E-08	3.50E-12	5	Rps3a1
-0.835097223	4.78E-08	3.58E-12	5	Metrnl
0.478805732	4.96E-08	3.72E-12	5	Rps4x
0.720209633	5.05E-08	3.78E-12	5	Gas5
0.925821167	5.17E-08	3.87E-12	5	Rpl35a
0.298424434	5.56E-08	4.17E-12	5	Rps19-ps3
-1.862787323	5.86E-08	4.39E-12	5	Ccl7
0.550719148	7.34E-08	5.50E-12	5	Rps2
-1.018358687	7.95E-08	5.96E-12	5	Tgfbr1
-0.904135416	9.44E-08	7.07E-12	5	Ccl4
0.44157582	9.91E-08	7.43E-12	5	Rps26
0.723468671	1.00E-07	7.49E-12	5	Rpl39
-1.993873548	1.07E-07	8.00E-12	5	Cbr2
-1.523972919	1.08E-07	8.06E-12	5	Ccl2
-1.301588811	1.50E-07	1.12E-11	5	Ctsl
-1.510190327	1.59E-07	1.19E-11	5	Msr1
-1.898646311	1.70E-07	1.27E-11	5	Cpd
0.304788438	2.22E-07	1.67E-11	5	Actb
1.487964252	2.24E-07	1.68E-11	5	Il1r2
0.451471654	2.41E-07	1.81E-11	5	Rplp2
-1.526996403	2.51E-07	1.88E-11	5	Plau
-0.794702856	3.09E-07	2.31E-11	5	Ctsc
-1.859634738	3.20E-07	2.40E-11	5	Rab31
0.43332952	3.37E-07	2.52E-11	5	Rpl8
-1.965237745	3.62E-07	2.71E-11	5	Tspan4
-1.629320082	3.71E-07	2.78E-11	5	Tmem37
0.739074946	3.83E-07	2.87E-11	5	Rpsa
-0.766523333	4.65E-07	3.48E-11	5	Mpeg1
1.652037356	5.75E-07	4.31E-11	5	H2-Ob
1.777929109	6.23E-07	4.67E-11	5	Klrd1
-0.872365972	6.84E-07	5.12E-11	5	Trf
-1.605843338	7.12E-07	5.33E-11	5	Gas7
1.49726315	7.39E-07	5.54E-11	5	Samsn1
1.317755884	7.83E-07	5.86E-11	5	Itgb7
-0.826716066	8.08E-07	6.05E-11	5	Plin2
0.514796877	8.32E-07	6.23E-11	5	Rps11
-1.248229665	8.54E-07	6.40E-11	5	Itpripl2
-1.93813626	9.01E-07	6.75E-11	5	Wwp1
0.564936961	9.10E-07	6.82E-11	5	Gm6251
-0.502329265	1.04E-06	7.80E-11	5	Itm2b
-0.71355356	1.09E-06	8.16E-11	5	Ets2
-1.241760316	1.18E-06	8.87E-11	5	Hpgds
2.553557516	1.25E-06	9.40E-11	5	Ccr7
0.636538802	1.30E-06	9.72E-11	5	Btg1
1.945741254	1.45E-06	1.09E-10	5	Glipr2
0.392429029	1.46E-06	1.09E-10	5	Rpl32
-0.639688697	1.53E-06	1.15E-10	5	Lamp2
0.676155249	1.54E-06	1.15E-10	5	Rps7
-0.668836926	1.65E-06	1.24E-10	5	Tpp1
-1.134294674	1.86E-06	1.40E-10	5	S100a1
-1.715540419	1.93E-06	1.44E-10	5	Tmem9b
1.014624139	2.29E-06	1.72E-10	5	Gpr132
0.477837897	2.33E-06	1.75E-10	5	Srgn
0.619546436	2.50E-06	1.87E-10	5	Rpl13a
-1.333755987	2.68E-06	2.01E-10	5	Nceh1
-1.287538304	2.74E-06	2.05E-10	5	Cln8
-1.329700784	3.04E-06	2.28E-10	5	Egr1
1.46628834	3.11E-06	2.33E-10	5	Dpp4
-0.702300427	3.37E-06	2.53E-10	5	Aif1
-1.081147734	3.41E-06	2.55E-10	5	Rab3il1

0.370433928	3.55E-06	2.66E-10	5	Calm1
-1.171308465	4.01E-06	3.00E-10	5	Cebpa
0.522648899	4.29E-06	3.21E-10	5	Coro1a
0.463708247	4.92E-06	3.68E-10	5	Pfn1
0.332302362	6.30E-06	4.72E-10	5	Eef1a1
-1.174659577	6.56E-06	4.91E-10	5	P2ry6
-0.586080615	6.74E-06	5.05E-10	5	Hexa
0.33750737	7.09E-06	5.31E-10	5	Rps24
-1.018406098	8.01E-06	6.00E-10	5	Blnk
-1.466304562	8.29E-06	6.21E-10	5	Lpl
-0.749368761	8.75E-06	6.55E-10	5	Ccl6
-1.228998228	9.34E-06	6.99E-10	5	Zfhx3
1.265833176	9.37E-06	7.02E-10	5	Strbp
-0.710989091	1.02E-05	7.62E-10	5	Mtss1
-0.741206104	1.02E-05	7.65E-10	5	Abhd12
-0.669434429	1.10E-05	8.26E-10	5	Cyfip1
-0.777174061	1.25E-05	9.40E-10	5	Ms4a6d
-1.069624418	1.28E-05	9.57E-10	5	Gdf15
-1.288283191	1.33E-05	9.94E-10	5	Tanc2
0.710341327	1.36E-05	1.02E-09	5	Rps15a-ps6
-0.969862783	1.58E-05	1.19E-09	5	Cd302
-1.023357735	1.62E-05	1.22E-09	5	Nucb1
-0.654466974	1.64E-05	1.23E-09	5	Cndp2
0.500752431	1.77E-05	1.32E-09	5	Shfm1
-0.605402649	1.95E-05	1.46E-09	5	Psap
-0.762864713	2.47E-05	1.85E-09	5	Apobec1
-1.237656941	2.52E-05	1.89E-09	5	Fcgr1
-1.219673387	2.86E-05	2.14E-09	5	Sash1
0.368081462	3.05E-05	2.28E-09	5	Gm11517
-0.733957731	3.31E-05	2.48E-09	5	Atp6v1a
0.586405912	3.33E-05	2.49E-09	5	Mir703
2.111862338	3.37E-05	2.53E-09	5	Tbc1d4
-0.499823151	3.45E-05	2.58E-09	5	Ctsa
0.389818933	3.60E-05	2.69E-09	5	Rps5
-0.917623863	3.93E-05	2.95E-09	5	Rhoc
-0.920732523	4.07E-05	3.05E-09	5	Gusb
0.433433204	4.12E-05	3.08E-09	5	Gnb2l1
-0.560295296	4.47E-05	3.35E-09	5	Serinc3
-0.868172405	4.50E-05	3.37E-09	5	Snx6
1.218084353	4.87E-05	3.64E-09	5	Rab11fip1
-1.035875959	4.90E-05	3.67E-09	5	Pon2
1.908672006	4.90E-05	3.67E-09	5	Tmem39a
0.501539309	4.93E-05	3.69E-09	5	Atox1
-0.885197013	5.09E-05	3.81E-09	5	Sec14l1
-1.88228843	5.18E-05	3.88E-09	5	Rgl1
-0.853313158	5.90E-05	4.42E-09	5	Lat2
-0.989229882	6.24E-05	4.67E-09	5	Lair1
-1.059569346	6.32E-05	4.74E-09	5	2610507B11Rik
0.723127949	6.32E-05	4.74E-09	5	Bcl2a1d
-0.937296297	6.64E-05	4.98E-09	5	Cd300ld
-0.594842593	7.00E-05	5.24E-09	5	Capg
-0.912072924	7.77E-05	5.82E-09	5	Il10rb
-0.836647686	7.78E-05	5.82E-09	5	Creg1
-0.69683324	7.78E-05	5.82E-09	5	Sirpa
-1.716477862	8.51E-05	6.37E-09	5	F13a1
0.319303953	9.75E-05	7.30E-09	5	Rpl13
0.545338943	0.000102499	7.68E-09	5	Rpl22
-0.460032485	0.000103499	7.75E-09	5	Cybb
-0.651739007	0.000108097	8.10E-09	5	Pea15a
-1.073449316	0.000121528	9.10E-09	5	Pld3
0.555439427	0.00013043	9.77E-09	5	Rpl37a

-0.45277911	0.000133198	9.98E-09	5	Nfkbiz
-0.523110357	0.000161836	1.21E-08	5	Lrp1
-0.687090312	0.000164816	1.23E-08	5	Clec12a
-0.692695646	0.000175794	1.32E-08	5	Mfsd1
0.654772621	0.000187314	1.40E-08	5	Rpl29
0.336924133	0.000196769	1.47E-08	5	Cox4i1
-1.404321783	0.000198599	1.49E-08	5	Dst
-0.56540014	0.000220265	1.65E-08	5	Atp6ap1
0.33516284	0.000221598	1.66E-08	5	Pabpc1
0.706557813	0.000222667	1.67E-08	5	S100a4
-0.605354102	0.000256815	1.92E-08	5	Tmem50a
-0.422953052	0.000257371	1.93E-08	5	Gns
-0.445446349	0.000257637	1.93E-08	5	Anxa5
-1.121274667	0.000265739	1.99E-08	5	Mpp1
-0.727937241	0.000272657	2.04E-08	5	Rab11fip5
0.822378354	0.000275789	2.07E-08	5	H2afz
1.765259496	0.000289052	2.16E-08	5	4930523C07Rik
0.914356604	0.000309401	2.32E-08	5	Rpl36al
0.481443804	0.00031561	2.36E-08	5	Rps8
-1.508665721	0.000321839	2.41E-08	5	Cfh
0.807311783	0.000341409	2.56E-08	5	Bhlhe40
1.298781326	0.000361183	2.71E-08	5	Plscr1
-0.636004278	0.000365504	2.74E-08	5	Tlr2
1.252893194	0.000397238	2.98E-08	5	Klrb1b
-0.479061663	0.000404319	3.03E-08	5	Cd68
-0.545859252	0.000412682	3.09E-08	5	Atp6v0a1
0.258494576	0.000456294	3.42E-08	5	Gm4832
-0.721560282	0.000456761	3.42E-08	5	Ier3
-1.370799852	0.000498815	3.74E-08	5	Slc37a2
-1.406079414	0.000536242	4.02E-08	5	Rin2
-1.461061192	0.000554433	4.15E-08	5	Gstm1
-1.433475564	0.000555173	4.16E-08	5	Cxcl10
-0.487594805	0.000594928	4.46E-08	5	Akr1a1
-0.475132176	0.000597659	4.48E-08	5	Lgals1
0.769567723	0.000649697	4.87E-08	5	Rps16
-1.054962825	0.000663698	4.97E-08	5	Cd36
-0.999149518	0.000711983	5.33E-08	5	Cx3cr1
-1.085612313	0.000731139	5.48E-08	5	Rassf4
-0.699131466	0.000747065	5.60E-08	5	Tmem66
-1.272016505	0.000765064	5.73E-08	5	Rcan1
1.420378744	0.00077527	5.81E-08	5	Ramp3
-0.503103423	0.000803941	6.02E-08	5	Lgals3bp
-0.639510792	0.000864426	6.47E-08	5	Slamf9
1.443342703	0.000869342	6.51E-08	5	Ifitm1
-0.516911256	0.000870708	6.52E-08	5	Myof
0.707910291	0.000897857	6.72E-08	5	Ifi30
-0.652901685	0.000902439	6.76E-08	5	Hexb
-0.666375135	0.000912357	6.83E-08	5	Lmna
-1.667199593	0.000982944	7.36E-08	5	Ccl12
-1.254543794	0.001099773	8.24E-08	5	Cxcl1
-0.881215943	0.001165764	8.73E-08	5	Dstn
0.326098049	0.001211809	9.08E-08	5	Gm13826
-0.682253668	0.001243438	9.31E-08	5	Lgals9
-0.562444862	0.00132278	9.91E-08	5	Dab2
0.608865808	0.001446784	1.08E-07	5	Gm2a
-0.634063998	0.001467656	1.10E-07	5	Ppt1
-1.092401491	0.001480099	1.11E-07	5	P2rx4
-1.131705274	0.001485094	1.11E-07	5	Tlr7
-0.462859568	0.001761934	1.32E-07	5	Cyth4
-1.38673635	0.001838888	1.38E-07	5	Asph
0.391414559	0.001874566	1.40E-07	5	H2-K1

-0.79878316	0.001947175	1.46E-07	5	Arap1
0.747573611	0.002065705	1.55E-07	5	Myl6
-1.449330603	0.002130842	1.60E-07	5	Gas6
-1.56553336	0.002138569	1.60E-07	5	Alas1
1.615689657	0.002241143	1.68E-07	5	Avpi1
1.517147604	0.002255868	1.69E-07	5	H2-Oa
-0.654325407	0.002280228	1.71E-07	5	Usp8
-0.642594956	0.002409923	1.80E-07	5	Ptplad2
-0.746766044	0.00256426	1.92E-07	5	Ptpre
-1.070555474	0.002732535	2.05E-07	5	Ifi27
-1.471863135	0.002988568	2.24E-07	5	Apbb2
0.300242623	0.003117674	2.33E-07	5	Rplp1
-0.387117484	0.003122832	2.34E-07	5	Unc93b1
-0.615916625	0.003338852	2.50E-07	5	2010111I01Rik
2.273794048	0.003349539	2.51E-07	5	Ccl22
-0.664775307	0.003355948	2.51E-07	5	Hspa1a
0.788685687	0.003358875	2.52E-07	5	H2-DMa
-1.116050077	0.003477992	2.60E-07	5	Sypl
0.882694876	0.003562025	2.67E-07	5	H2afy
-1.64961799	0.003587111	2.69E-07	5	Pon3
-1.095632872	0.003663792	2.74E-07	5	Itga6
1.305884602	0.003741222	2.80E-07	5	Myo1g
0.337435409	0.003995238	2.99E-07	5	Rps15a-ps4
0.809790121	0.004158004	3.11E-07	5	Psme2b
1.281845196	0.004284167	3.21E-07	5	Zbtb18
-0.545323939	0.00433235	3.24E-07	5	Jun
-0.734960823	0.00437883	3.28E-07	5	Myo5a
-1.674812389	0.00443467	3.32E-07	5	Cdk18
-0.859113063	0.004470465	3.35E-07	5	Clec4a3
-0.801140188	0.004574993	3.43E-07	5	Tmem189
1.472828826	0.004756237	3.56E-07	5	Map3k14
-0.592319742	0.004936813	3.70E-07	5	Laptm4a
0.454251858	0.005182225	3.88E-07	5	Crip1
-0.673388614	0.005532139	4.14E-07	5	Dnase2a
-0.450827619	0.005586942	4.18E-07	5	Sdc3
0.599967705	0.005716717	4.28E-07	5	Rps23
1.007182021	0.005814062	4.35E-07	5	Mbtd1
0.635934774	0.005886362	4.41E-07	5	Eif5a
-0.667785828	0.006141358	4.60E-07	5	Hprt
-0.387960154	0.006342424	4.75E-07	5	Mfsd11
-0.955300566	0.006356743	4.76E-07	5	Oxct1
-0.532297302	0.006653537	4.98E-07	5	Aplp2
-1.123502069	0.006810822	5.10E-07	5	Fcgrt
-1.054057904	0.007216097	5.40E-07	5	Gab2
-0.274244012	0.007329837	5.49E-07	5	Scamp2
1.829594285	0.007444398	5.58E-07	5	Cd209a
-0.37585472	0.007557875	5.66E-07	5	Map7d1
-1.094892666	0.007901204	5.92E-07	5	Uap1l1
-0.442464693	0.008508832	6.37E-07	5	Lgmn
-0.987818936	0.008744998	6.55E-07	5	Atp13a2
-1.415895989	0.008758337	6.56E-07	5	Daglb
-0.561899074	0.009296543	6.96E-07	5	Axl
0.515995192	0.009344879	7.00E-07	5	Rps15a
0.782470483	0.009933834	7.44E-07	5	Psmb8
0.976778013	0.010672492	7.99E-07	5	Napsa
-0.665644448	0.010698899	8.01E-07	5	Renbp
-0.438895031	0.010818988	8.10E-07	5	Gp49a
-0.977384658	0.010879307	8.15E-07	5	Ptpra
-0.505957814	0.011092277	8.31E-07	5	Rtn4
0.516175476	0.011480715	8.60E-07	5	Rpl35
-0.643112416	0.01162034	8.70E-07	5	Fam89b

-0.389066437	0.011820389	8.85E-07	5	Lilrb4
-0.994115347	0.011899466	8.91E-07	5	Frmd4b
-0.929344578	0.013522356	1.01E-06	5	Aprt
-1.930770168	0.014045456	1.05E-06	5	Stard8
-1.606560562	0.014659232	1.10E-06	5	Acaa2
-0.878389524	0.014845947	1.11E-06	5	Sgk1
0.301578769	0.015148147	1.13E-06	5	Hsp90ab1
-0.517271381	0.015201929	1.14E-06	5	Atf3
-1.063960718	0.015264681	1.14E-06	5	Clec4d
-0.640666432	0.0152813	1.14E-06	5	Mapk3
-0.537845049	0.015683864	1.17E-06	5	Ski
-0.615300461	0.016124914	1.21E-06	5	Mef2a
-0.537852015	0.016156048	1.21E-06	5	Tnfsf12
-0.378935928	0.017348326	1.30E-06	5	Qk
-1.056035419	0.017783881	1.33E-06	5	Milr1
-0.498249795	0.01851302	1.39E-06	5	Mapre2
1.598777839	0.018699962	1.40E-06	5	Klrk1
-0.94436074	0.018877368	1.41E-06	5	Pmepa1
-0.710446435	0.019992621	1.50E-06	5	Ccl9
-0.373794246	0.021199885	1.59E-06	5	Snx5
-0.481727796	0.021513555	1.61E-06	5	Macf1
-0.649006224	0.022051365	1.65E-06	5	Ano6
1.302878465	0.022067449	1.65E-06	5	Crem
1.242019537	0.022962399	1.72E-06	5	Gcnt2
1.027829187	0.023341716	1.75E-06	5	Lmnb1
-0.663362298	0.024505466	1.84E-06	5	Lrpap1
-1.546991385	0.024560528	1.84E-06	5	P2ry12
-0.73639947	0.026189392	1.96E-06	5	0610031J06Rik
-1.071706323	0.026771757	2.01E-06	5	Dnase1l1
-1.917777964	0.027947494	2.09E-06	5	Nes
-1.615305537	0.029552425	2.21E-06	5	Pdgfa
1.333730804	0.030575769	2.29E-06	5	Cd24a
-1.190119884	0.031328629	2.35E-06	5	Slc43a2
0.980026748	0.032012624	2.40E-06	5	Plbd1
-0.61652601	0.033494629	2.51E-06	5	Eea1
-0.52633165	0.033795036	2.53E-06	5	Mef2c
-0.660283218	0.035167881	2.63E-06	5	Cttnbp2nl
-0.30331375	0.035511337	2.66E-06	5	Vamp3
-1.58602892	0.036457217	2.73E-06	5	Wdfy3
-1.377995418	0.036617213	2.74E-06	5	Clec5a
0.935422225	0.03708674	2.78E-06	5	Got1
-0.359171058	0.037860728	2.84E-06	5	App
-0.923276727	0.038675695	2.90E-06	5	Fnip2
-0.999176087	0.038919994	2.91E-06	5	Mrps18a
-0.979257629	0.039326721	2.95E-06	5	Plbd2
-0.547844184	0.039710616	2.97E-06	5	Emp1
-0.438892702	0.040948769	3.07E-06	5	Sdcbp
-0.85871138	0.041701847	3.12E-06	5	Gde1
0.779191444	0.04325522	3.24E-06	5	Ece1
-0.52065538	0.043943779	3.29E-06	5	Ier2
-0.337676091	0.044216267	3.31E-06	5	Tcn2
-0.948516433	0.052058051	3.90E-06	5	Por
-1.467288022	0.057572454	4.31E-06	5	Mt2
-0.61198205	0.059945738	4.49E-06	5	Pafah1b2
0.819711981	0.060965298	4.57E-06	5	Csf2rb
0.538601029	0.063196506	4.73E-06	5	Pcbp2
-0.54900198	0.063217026	4.73E-06	5	Wbp2
-0.423486008	0.064000446	4.79E-06	5	Itgam
-0.318363443	0.067149095	5.03E-06	5	Tgfbi
0.610703794	0.067172971	5.03E-06	5	Tma7
-1.199617303	0.067802671	5.08E-06	5	Itfg3

-0.652812617	0.069756761	5.22E-06	5	Cyb5r3
-0.615047797	0.070415469	5.27E-06	5	U2af1l4
0.793620274	0.072346224	5.42E-06	5	U2af1
-0.600554893	0.072683014	5.44E-06	5	Ccrl2
1.034168539	0.072885311	5.46E-06	5	Cd274
-0.602826024	0.074589516	5.59E-06	5	Tubb6
-1.345998883	0.078150985	5.85E-06	5	Mtmr10
-0.675848022	0.078151476	5.85E-06	5	Ms4a6b
-0.785688702	0.078357525	5.87E-06	5	Plod3
-0.58276868	0.081527371	6.11E-06	5	Plk2
-1.10663974	0.082671982	6.19E-06	5	Idh2
-0.597535513	0.08324489	6.23E-06	5	Prkacb
-0.575505576	0.084798463	6.35E-06	5	Jmjd1c
-0.779622751	0.085997457	6.44E-06	5	Phlda1
-0.573792282	0.08617901	6.45E-06	5	Atp6v1b2
0.704053391	0.086675777	6.49E-06	5	Btf3
-0.531499218	0.088539945	6.63E-06	5	Fam3c
0.704111648	0.089470802	6.70E-06	5	Map3k1
-0.749608998	0.090342729	6.77E-06	5	Pilra
0.4668398	0.092551863	6.93E-06	5	Npm1
-0.26064809	0.094564015	7.08E-06	5	Anxa3
-0.773339906	0.098091842	7.35E-06	5	Cited2
-0.395806509	0.10556635	7.91E-06	5	Rgs10
0.972717565	0.106066362	7.94E-06	5	Bloc1s2
-0.560789958	0.10620783	7.95E-06	5	Npc1
-1.117678132	0.106870097	8.00E-06	5	Rasa1
0.578051171	0.107732128	8.07E-06	5	Vasp
-0.668255934	0.110329535	8.26E-06	5	Dok3
0.968654939	0.111739802	8.37E-06	5	Tspan13
-0.385534165	0.111845642	8.38E-06	5	Adam17
1.216696643	0.118837563	8.90E-06	5	Traf1
-1.525248543	0.119239776	8.93E-06	5	Igfbp4
1.197784992	0.122359193	9.16E-06	5	Uck2
-1.433396592	0.123221067	9.23E-06	5	Xdh
-0.38651101	0.125058176	9.37E-06	5	Tmem59
-1.290714342	0.125069219	9.37E-06	5	Angptl2
1.027559138	0.125381572	9.39E-06	5	Nup210
-1.366944045	0.125635799	9.41E-06	5	Hk3
-0.581716326	0.126703808	9.49E-06	5	Fam21
-1.366953126	0.127083887	9.52E-06	5	Tmem86a
-0.817028042	0.129400826	9.69E-06	5	Hspa1b
-1.387406192	0.129856701	9.73E-06	5	Pla2g15
-0.552343967	0.138304861	1.04E-05	5	Ilk
-0.524769711	0.138569431	1.04E-05	5	Lmo2
-1.208752291	0.140176639	1.05E-05	5	Hmg20b
0.487881034	0.141832606	1.06E-05	5	Ifitm2
0.375030641	0.147020204	1.10E-05	5	Uba52
0.903402903	0.147069251	1.10E-05	5	Etv3
-0.978490675	0.152775115	1.14E-05	5	Vma21
-0.652317239	0.160389752	1.20E-05	5	Plxnb2
-1.521768535	0.164177285	1.23E-05	5	Lifr
-0.972706734	0.169333143	1.27E-05	5	Tlr13
-0.427481355	0.16955399	1.27E-05	5	Rrbp1
1.014074156	0.173242595	1.30E-05	5	Gpr171
-0.847556622	0.173782922	1.30E-05	5	Lyz1
-0.385384962	0.174387952	1.31E-05	5	Fos
-0.802526699	0.17553806	1.31E-05	5	Vat1
-0.418045153	0.179220096	1.34E-05	5	Wfdc17
-0.943466933	0.18189491	1.36E-05	5	Pdlim4
-0.792231648	0.182925922	1.37E-05	5	Tns3
-0.84097121	0.18381819	1.38E-05	5	Fcgr4

0.304304959	0.190533198	1.43E-05	5	Rps21
-0.769401558	0.191386567	1.43E-05	5	Snx30
-0.508401619	0.192708738	1.44E-05	5	Tpd52
1.164565997	0.193256449	1.45E-05	5	Gm11974
-0.427110917	0.19327564	1.45E-05	5	Os9
-0.330975328	0.198500421	1.49E-05	5	Atp2b1
-1.186778658	0.199879214	1.50E-05	5	Tmem205
-1.462318227	0.20011192	1.50E-05	5	Ltc4s
-0.686992113	0.203426988	1.52E-05	5	Dnmt3a
-0.660960249	0.203893549	1.53E-05	5	Cpt1a
-0.447888433	0.205327122	1.54E-05	5	Sdc4
-0.340820249	0.205374509	1.54E-05	5	Mat2a
1.265947851	0.212302873	1.59E-05	5	Ahr
-0.468891112	0.217604923	1.63E-05	5	Fuca1
-0.523128556	0.222556574	1.67E-05	5	Ergic3
-1.091724855	0.222673526	1.67E-05	5	Idh1
-1.441202972	0.224265132	1.68E-05	5	Lonrf3
0.722057601	0.226049101	1.69E-05	5	Il2rg
0.619449515	0.226663346	1.70E-05	5	Il1b
-0.652749375	0.227219736	1.70E-05	5	Plec
0.448694804	0.237211328	1.78E-05	5	S100a6
2.157322267	0.237936457	1.78E-05	5	Ly6d
-1.52194803	0.238844276	1.79E-05	5	Maf
-0.322418151	0.240582434	1.80E-05	5	Rabac1
-0.987321735	0.241055074	1.81E-05	5	Gas2l3
-0.774161437	0.254548904	1.91E-05	5	Cebpg
-1.659944644	0.272681082	2.04E-05	5	Dusp6
-0.756285218	0.272831002	2.04E-05	5	Blvra
-0.341637714	0.285963698	2.14E-05	5	Stom
-1.06534556	0.29052641	2.18E-05	5	Ube3a
-0.572301101	0.301551456	2.26E-05	5	Entpd1
1.271375174	0.30773888	2.30E-05	5	B3gnt5
-1.310163487	0.311487639	2.33E-05	5	Lhfpl2
-1.432998032	0.311705319	2.33E-05	5	Cmklr1
-0.444803789	0.329941428	2.47E-05	5	Ppp1r12c
-0.41607631	0.330537849	2.48E-05	5	Pfdn1
-0.806942406	0.338148231	2.53E-05	5	1110058L19Rik
-0.895520105	0.340727599	2.55E-05	5	Pdxk
-0.807937957	0.372344474	2.79E-05	5	Alg5
-0.581864214	0.375381948	2.81E-05	5	Zfand2a
-0.474979029	0.38764589	2.90E-05	5	Psmd12
0.508317216	0.397226983	2.98E-05	5	Rps10
-0.374522356	0.398068597	2.98E-05	5	Ociad1
-0.602646379	0.42806453	3.21E-05	5	Nupr1
-0.547476793	0.430087003	3.22E-05	5	Adam15
-0.374773675	0.440125906	3.30E-05	5	Emp3
-1.285937565	0.441839672	3.31E-05	5	Abca9
0.33258979	0.444645555	3.33E-05	5	Rps3
-0.467973172	0.447106666	3.35E-05	5	Tbxas1
-1.698698246	0.456922828	3.42E-05	5	Ccl8
-0.311194944	0.46367709	3.47E-05	5	Cenpb
-0.5200636	0.468811112	3.51E-05	5	Rragc
1.090869045	0.482212852	3.61E-05	5	Grasp
-0.44072602	0.509824285	3.82E-05	5	Krtcap2
-0.277699198	0.515753594	3.86E-05	5	Rasgef1b
-0.845253353	0.517274323	3.87E-05	5	Dynlt3
0.280992257	0.535975432	4.01E-05	5	Sh3bgrl3
0.777613564	0.548242619	4.11E-05	5	Nr4a2
-1.387057205	0.559801914	4.19E-05	5	Pura
-1.484194694	0.564637289	4.23E-05	5	Itga9
-0.748575585	0.583438277	4.37E-05	5	Fam195b

-1.084905842	0.585760634	4.39E-05	5	Galc
0.982923423	0.589072349	4.41E-05	5	Gng10
-0.486071657	0.590501222	4.42E-05	5	Scamp3
1.34783991	0.601414563	4.50E-05	5	Stat4
1.0078021	0.602506618	4.51E-05	5	Pmvk
0.776716976	0.605956659	4.54E-05	5	Dennd4a
-0.48714057	0.614579637	4.60E-05	5	Fam46a
-1.318051698	0.620504894	4.65E-05	5	Cyb561d2
0.662803069	0.631048928	4.73E-05	5	Sec61b
-0.678087908	0.631723819	4.73E-05	5	Ndufs7
-1.176095447	0.633136969	4.74E-05	5	1810011H11Rik
-0.682195114	0.636518173	4.77E-05	5	Ppt2
-1.569463489	0.636601729	4.77E-05	5	Fblim1
-2.070394552	0.640882635	4.80E-05	5	Lyve1
-1.244311169	0.663346973	4.97E-05	5	Slc16a10
0.320347549	0.663834115	4.97E-05	5	Lrrc58
-0.801947471	0.68674538	5.14E-05	5	Tmem43
-0.566397652	0.690602908	5.17E-05	5	Gng2
-0.555043873	0.692862809	5.19E-05	5	Bin1
-0.605324605	0.704845269	5.28E-05	5	Glipr1
0.434602522	0.707461453	5.30E-05	5	Cnbp
1.170829145	0.716852064	5.37E-05	5	Pvrl1
-0.310125354	0.721073538	5.40E-05	5	Rnf13
0.366763363	0.738677082	5.53E-05	5	Naca
0.844167162	0.739183041	5.54E-05	5	Psma5
-1.203982239	0.75304195	5.64E-05	5	Slc29a1
-0.862692918	0.762700358	5.71E-05	5	Ctnnd1
-0.589906078	0.77508714	5.81E-05	5	Atp6v1e1
0.284738471	0.804381853	6.02E-05	5	Hnrnpf
-0.458261456	0.818170129	6.13E-05	5	Ncf1
-0.966462662	0.828168311	6.20E-05	5	Phb
-0.603093587	0.838673933	6.28E-05	5	Sptlc2
-1.029035505	0.849646653	6.36E-05	5	Abcc3
-1.393575654	0.850779461	6.37E-05	5	Slc6a8
-0.566927962	0.866464972	6.49E-05	5	Gnaq
-1.287978709	0.878546229	6.58E-05	5	Arap3
-0.9564384	0.943307571	7.06E-05	5	Ppm1h
-0.45585644	0.954298926	7.15E-05	5	Szrd1
0.894742956	0.977319814	7.32E-05	5	Malt1
-0.436203874	0.989063324	7.41E-05	5	Lamtor4
-0.543018359	0.992273875	7.43E-05	5	Arhgap17
-0.7073461	0.993577365	7.44E-05	5	Itgav
-0.595429288	0.995720377	7.46E-05	5	Pttg1ip
0.477232888	1	7.67E-05	5	Ywhaz
-0.504859525	1	7.95E-05	5	Cpsf2
-0.535267715	1	8.05E-05	5	Ifnar1
-0.299015507	1	8.23E-05	5	Nckap1l
-0.733783956	1	8.25E-05	5	Bag3
-1.164419451	1	8.36E-05	5	Map4k3
-0.485458444	1	8.41E-05	5	Mmp14
-0.336760072	1	8.47E-05	5	Usf2
1.04137251	1	8.48E-05	5	Rhof
-0.636072285	1	8.64E-05	5	Man1c1
-0.340923036	1	8.78E-05	5	Magt1
-0.339085232	1	8.78E-05	5	Itgb1
-0.429349333	1	8.78E-05	5	Bag6
-1.173686264	1	8.86E-05	5	Slc9a9
-0.697199049	1	8.97E-05	5	Sfmbt1
-0.578090027	1	9.04E-05	5	Gatm
0.613838274	1	9.20E-05	5	Hmgb2
-0.742058353	1	9.20E-05	5	Pacsin2

-0.330234334	1	9.35E-05	5	Nisch
-0.441325524	1	9.36E-05	5	Grina
-1.037697608	1	9.45E-05	5	Ulk2
-0.361843147	1	9.58E-05	5	Ash1l
-0.611459496	1	9.59E-05	5	Bcl2l1
-0.289637721	1	9.70E-05	5	Atp6v1h
-0.665856005	1	9.92E-05	5	Lxn
-0.671053954	1	9.99E-05	5	Casp4
-0.329112895	1	0.0001	5	AF251705
-0.358927861	1	0.0001009	5	Mylip
-0.455960248	1	0.0001011	5	Peak1
-0.512068615	1	0.0001066	5	Cebpb
0.977476416	1	0.0001078	5	Sep.06
-0.547350271	1	0.0001089	5	Rnpep
-0.664741147	1	0.0001089	5	Lilra5
0.933637736	1	0.0001091	5	Isy1
0.694511906	1	0.0001099	5	Rpl22l1
-0.807861597	1	0.0001103	5	Ccrn4l
1.056584205	1	0.0001116	5	Birc2
-0.380192209	1	0.000113	5	Ly86
0.417295461	1	0.0001159	5	Sik3
-0.701053969	1	0.0001179	5	Ascc3
-0.581301622	1	0.0001198	5	Pitpnc1
-0.815639467	1	0.0001218	5	Add1
-0.652814698	1	0.0001241	5	Myo1e
-0.285860496	1	0.0001246	5	Prkcb
-0.574781567	1	0.0001261	5	Igsf6
-1.244539112	1	0.0001287	5	Crat
-0.531500263	1	0.0001319	5	Trip11
-0.326629162	1	0.0001331	5	Rap2b
-0.464770623	1	0.0001367	5	Vdac2
-0.378059836	1	0.0001373	5	A230046K03Rik
-1.358578875	1	0.0001385	5	Igf2r
-0.673003978	1	0.0001468	5	Rufy1
0.45893057	1	0.0001479	5	Gm6402
-0.538900711	1	0.0001504	5	Rnf7
-0.769257999	1	0.0001521	5	Mrps15
-0.268664554	1	0.0001543	5	Rcbtb2
-1.142194751	1	0.0001547	5	Arhgap22
-0.570670789	1	0.0001556	5	Polr2f
-0.680952456	1	0.0001561	5	Leprot
-0.563991506	1	0.0001574	5	Nrp2
-0.711915124	1	0.0001579	5	Kpnb1
0.618732767	1	0.0001589	5	Sik1
1.166200449	1	0.00016	5	Arl5c
-1.060364733	1	0.0001602	5	Rnf150
-1.238079662	1	0.0001639	5	Gtf3a
-0.882721643	1	0.0001653	5	Tbpl1
-0.632870708	1	0.0001667	5	Cmtm3
1.077376292	1	0.0001671	5	Cmtr1
-0.962572843	1	0.0001683	5	Sepn1
-0.64186369	1	0.0001685	5	Capn2
-0.419765401	1	0.0001738	5	Atp1b3
-1.552526134	1	0.000177	5	P2rx7
-0.458308584	1	0.0001778	5	Rock2
-0.684632451	1	0.0001786	5	Gmfb
-0.483214453	1	0.0001804	5	Prdx5
-0.750242635	1	0.0001804	5	Pik3cg
-0.461556009	1	0.0001831	5	Vkorc1
0.842920931	1	0.0001839	5	Spint1
-1.526140722	1	0.0001853	5	Slco2b1

-0.86326547	1	0.0001883	5	Mpv17
-0.65017453	1	0.0001896	5	Iqgap2
-1.201354672	1	0.0001906	5	Trim47
-0.550227222	1	0.0001906	5	Cpeb4
-0.725844192	1	0.0001922	5	Mbd3
-0.837476424	1	0.0001935	5	Selm
-0.34174276	1	0.000195	5	Itgb2
-0.455538962	1	0.0001953	5	Hook3
-0.470391443	1	0.0001964	5	Ech1
-0.349840982	1	0.0001981	5	Rab14
-1.529967641	1	0.0002	5	Pkp4
-1.069749835	1	0.0002	5	Pofut2
0.736499579	1	0.0002038	5	Slc7a11
-0.754230793	1	0.0002128	5	Cgrrf1
-0.77849581	1	0.0002156	5	Stx3
-0.851045195	1	0.0002217	5	Hcst
-0.965951586	1	0.0002222	5	Mgat1
-0.268313207	1	0.0002224	5	Fcer1g
-1.092498879	1	0.0002307	5	Pros1
-0.294704474	1	0.0002323	5	Snx2
-0.335472202	1	0.0002329	5	Napa
-1.688193069	1	0.0002339	5	Syngr1
-0.591683636	1	0.0002368	5	Ap3d1
-0.868709526	1	0.000239	5	Hgsnat
-0.276178472	1	0.0002393	5	Rell1
-0.323175494	1	0.0002414	5	Fam111a
-1.07170922	1	0.0002426	5	Hpgd
-0.347752308	1	0.0002452	5	Elovl1
-1.359764485	1	0.0002492	5	Oxr1
-0.360119031	1	0.0002505	5	Spred1
-0.458013973	1	0.0002517	5	Metap2
-1.234041404	1	0.000252	5	Arl11
-0.707419457	1	0.0002539	5	Ldlrap1
-0.494497449	1	0.0002559	5	Tnfaip2
-1.559338511	1	0.0002593	5	C4b
-0.644639366	1	0.0002616	5	Pitpnb
-0.589539365	1	0.0002623	5	Ap1s2
-0.566021705	1	0.0002631	5	Lpcat3
-0.47373965	1	0.0002634	5	Fcho2
-0.763419222	1	0.0002661	5	Serpinb8
-0.720923813	1	0.0002751	5	Neu1
-0.56053922	1	0.0002755	5	Sod2
-0.281073544	1	0.0002766	5	Eif2ak1
-1.111390897	1	0.0002776	5	Olfml3
0.757413136	1	0.0002776	5	Rel
0.601609669	1	0.0002797	5	Hnrnpa1
0.73024465	1	0.0002833	5	Dnajb6
0.6230168	1	0.0002839	5	Ube2j2
-1.433557409	1	0.0002842	5	Ctla2b
0.419636084	1	0.0002865	5	Rpl7
-0.632130315	1	0.000291	5	BC031181
0.523538507	1	0.0002937	5	Rab8b
-0.297993306	1	0.0002962	5	Ebp
0.334311148	1	0.0002966	5	Eif4a1
-0.470172929	1	0.0002983	5	Cuedc2
-0.551266538	1	0.0003007	5	Ifngr2
-0.331427374	1	0.0003032	5	Ypel3
-0.430347953	1	0.0003073	5	Arhgef3
-0.387320444	1	0.0003094	5	Rnf187
-0.325812637	1	0.0003128	5	Epb4.1l2
-0.286189623	1	0.000315	5	Eif4ebp1

0.299828922	1	0.000327	5	Cst3
-0.768347011	1	0.0003284	5	Syt11
0.393915281	1	0.0003316	5	Rpl3
-1.88257877	1	0.0003359	5	Cd163
-0.578999616	1	0.0003382	5	Slc25a28
-0.893616692	1	0.0003408	5	P4ha1
-0.334407618	1	0.0003424	5	Coa3
-0.372180341	1	0.0003429	5	Pnp
-0.801120596	1	0.0003478	5	Dtnbp1
-0.413414973	1	0.000348	5	Jak1
-0.529078517	1	0.0003486	5	Tec
0.921984816	1	0.0003505	5	Ccnd2
0.59694126	1	0.0003507	5	Cd82
-0.584654652	1	0.0003609	5	Lima1
-0.54083807	1	0.000369	5	Clock
-0.301861475	1	0.0003703	5	Mdm2
0.840250962	1	0.000371	5	Adrbk2
0.576833763	1	0.0003731	5	Atp5l
-0.575606037	1	0.0003846	5	Mrpl57
-0.618941557	1	0.0003869	5	Cadm1
-0.594939003	1	0.0003873	5	Lacc1
-0.442276095	1	0.0003938	5	Smc1a
0.761852216	1	0.0003964	5	Nsa2
-0.732362388	1	0.0003965	5	Leprotl1
-0.281616686	1	0.0003966	5	Pld4
-0.523080647	1	0.0003985	5	Adcy7
-1.315092134	1	0.0004003	5	Fmn1
-1.070991825	1	0.0004068	5	Rfc1
-0.526181689	1	0.0004121	5	Map4
-1.327928194	1	0.000416	5	Ltbp3
0.773096401	1	0.0004205	5	Elmsan1
-0.463088835	1	0.0004214	5	Tmem234
-0.54023024	1	0.0004241	5	Baz1b
-0.499003574	1	0.0004342	5	Atp8b4
-0.407073876	1	0.0004375	5	Hsbp1
-0.861887736	1	0.0004397	5	Aldh3b1
-1.402836726	1	0.0004407	5	Fgd4
-1.508428661	1	0.0004437	5	Fam20c
-0.393576485	1	0.0004472	5	Ppp1r18
0.496925296	1	0.0004524	5	Eef1b2
-0.553803508	1	0.0004697	5	Stt3b
-0.440242687	1	0.0004759	5	Aip
-1.097706734	1	0.0004775	5	Plekhg3
-0.322366986	1	0.000489	5	Tmem109
-0.850246158	1	0.0004891	5	Dpep2
-0.813941785	1	0.0004907	5	Itsn1
-0.270347031	1	0.0004969	5	Evi2a
-1.203842153	1	0.000515	5	Cpq
-0.40977277	1	0.0005172	5	Adam10
-0.881352066	1	0.0005259	5	Snapc5
-1.0473474	1	0.0005278	5	Ntpcr
-0.338310629	1	0.0005279	5	Ifi27l2a
-0.362381433	1	0.0005322	5	Tecr
-0.394894779	1	0.000536	5	Tmem30a
-0.709937566	1	0.0005418	5	B4galt6
-1.459028099	1	0.0005423	5	Fez2
-0.583525702	1	0.0005451	5	Man2a1
-0.974426395	1	0.0005464	5	Kidins220
1.457774207	1	0.0005516	5	Mcemp1
-0.410411161	1	0.0005619	5	Fundc2
-0.514806463	1	0.0005699	5	D17Wsu92e

1.110990465	1	0.0005755	5	Arap2
-0.370972099	1	0.0005836	5	Lst1
-0.840025279	1	0.000603	5	Ip6k1
0.585965603	1	0.0006034	5	Heatr3
-1.122597754	1	0.0006064	5	Aoah
-0.463036011	1	0.0006094	5	Parp2
-0.753455666	1	0.0006232	5	Mrpl13
-0.326111406	1	0.000627	5	Jkamp
-0.325240969	1	0.0006369	5	Sdhb
-0.506825443	1	0.000645	5	Nagk
-0.315446404	1	0.0006451	5	Man2b1
-0.318124433	1	0.0006513	5	Fkbp2
-0.282876772	1	0.0006516	5	Dlgap4
-1.075322038	1	0.0006562	5	Ophn1
-0.442200492	1	0.0006622	5	Lrp6
-0.89356742	1	0.0006624	5	Tmem55a
-0.287411559	1	0.0006637	5	Ap1b1
-1.094453487	1	0.000668	5	Tmem106b
-1.125888066	1	0.0006777	5	Gpnmb
-0.683458926	1	0.0006798	5	Slc7a7
-0.265869112	1	0.0006896	5	Wdr26
-0.650108262	1	0.0006906	5	0610012G03Rik
-0.437978241	1	0.000704	5	Exoc6b
-0.614139265	1	0.0007101	5	Diap2
-0.490185817	1	0.0007136	5	Frrs1
-0.308218297	1	0.0007177	5	Wbp4
-0.725047776	1	0.0007249	5	Prdx4
-0.48065619	1	0.0007379	5	Tmbim1
0.255783356	1	0.0007521	5	Cox6b1
-0.857408894	1	0.0007526	5	Ubqln2
-0.431419618	1	0.0007551	5	Tlk1
-0.512265816	1	0.000756	5	Ctns
-0.376613828	1	0.0007733	5	Derl1
-0.510572816	1	0.0007742	5	Psmc5
-0.342699874	1	0.0007874	5	Gsk3b
-0.893935258	1	0.000788	5	Clcn7
-0.496539995	1	0.0007906	5	Atp6v1c1
-0.271031884	1	0.0007952	5	S100a13
-0.432245626	1	0.0007978	5	Acsl4
-0.925262248	1	0.0008006	5	Plod1
-0.618477539	1	0.0008177	5	Senp2
-0.339628705	1	0.0008194	5	Gadd45g
-0.567483946	1	0.0008334	5	Dnajc13
-1.008091164	1	0.0008368	5	Tmem206
-0.582557208	1	0.0008437	5	Agps
-0.439208225	1	0.0008506	5	Bag1
-0.316546302	1	0.0008522	5	Tsc22d3
-0.882099367	1	0.0008626	5	Acp5
0.442049846	1	0.0008683	5	Ppp1ca
-1.056307932	1	0.0008802	5	Aldh9a1
-0.282898403	1	0.0008874	5	Tor1aip1
-0.364969097	1	0.0008943	5	Ssh2
-1.124428307	1	0.0008957	5	Rfc2
-0.726335722	1	0.0009083	5	Tmcc3
-0.620153733	1	0.0009162	5	Apc
-0.40869423	1	0.0009173	5	Txn1
-0.292005158	1	0.0009212	5	Tmed3
-0.370780875	1	0.0009244	5	Slc15a3
-1.04529448	1	0.0009268	5	Nbr1
-0.906671008	1	0.000936	5	Atp2b4
-0.531586233	1	0.0009388	5	Kif1b

-1.147523461	1	0.0009553	5	Col14a1
-0.285631543	1	0.0009557	5	Slc6a6
0.51746133	1	0.0009567	5	Srsf3
0.535007701	1	0.0009585	5	Rpl23
-1.011820692	1	0.0009613	5	St3gal6
-0.272952144	1	0.0009761	5	Kdm7a
-0.632498688	1	0.000994	5	Prrc1
-0.864315865	1	0.0009964	5	Scoc
-0.356677018	1	0.0010052	5	Scaf11
-0.843985581	1	0.0010115	5	Sgsh
-0.675627708	1	0.0010188	5	Pik3ca
-0.293755399	1	0.0010274	5	Apobr
-0.816605656	1	0.0010317	5	Agap3
0.887644053	1	0.0010328	5	Runx3
-0.267529332	1	0.0010337	5	Ehbp1l1
-0.34246911	1	0.0010473	5	Gpr65
-0.591158506	1	0.001048	5	Nlrp3
-0.974467562	1	0.001055	5	Cyp4v3
-0.660875738	1	0.0010626	5	Med15
-0.520850291	1	0.0010647	5	Cds2
0.314137102	1	0.0010656	5	Rpl4
-0.7739743	1	0.0010817	5	Smagp
-0.345416875	1	0.0010846	5	Hip1
-1.149136989	1	0.0010877	5	Tex264
-0.295838677	1	0.001088	5	Mctp1
-0.273147777	1	0.0010925	5	Rbm5
-0.310793679	1	0.0010949	5	Cdt1
-0.649070704	1	0.0010978	5	Adap2
-0.660522129	1	0.0011022	5	Usp24
-0.646371858	1	0.0011088	5	Hadh
0.784924995	1	0.0011171	5	Map4k1
0.947626845	1	0.0011229	5	Vps37b
-0.804930834	1	0.0011236	5	Stxbp5
-0.970408038	1	0.0011237	5	Arhgap10
-0.426653285	1	0.0011366	5	1810058I24Rik
-0.328523016	1	0.0011497	5	BC003331
-0.827612322	1	0.0011579	5	1700017B05Rik
-0.306420219	1	0.0011597	5	Bsg
-1.175929419	1	0.0011616	5	Creb5
-0.764215406	1	0.0011661	5	Mfsd5
-0.470185364	1	0.0011663	5	Cox20
-1.021331795	1	0.0011718	5	Tmem214
-0.714118984	1	0.0011861	5	Ephx1
-0.434312123	1	0.00119	5	Atp7a
-0.78717283	1	0.0011966	5	Baiap2
0.354551319	1	0.0012132	5	Msn
-0.788402902	1	0.0012395	5	Ncln
-0.540681884	1	0.0012768	5	Trappc2l
-1.069682199	1	0.001277	5	Alox5
-0.295919981	1	0.0012774	5	Polr2j
-0.361834209	1	0.0012929	5	Cab39
0.615328858	1	0.0012944	5	Psme1
-0.267597417	1	0.001296	5	Csf2ra
-1.253090814	1	0.0013276	5	Ptgs1
-1.090525165	1	0.0013278	5	Ch25h
-0.439984354	1	0.0013421	5	Cers2
-0.262680087	1	0.0013441	5	1110007C09Rik
1.637732508	1	0.0013649	5	Cyb561a3
0.857686053	1	0.0013658	5	Cbfa2t3
-0.329565914	1	0.0013852	5	Flna
0.442810747	1	0.001424	5	Pcgf5

0.26011652	1	0.0015057	5	Son
-0.401012668	1	0.0015127	5	Abi1
-0.662562497	1	0.0015167	5	Actr8
-0.283985049	1	0.0015243	5	Glg1
-0.438210863	1	0.0015368	5	Utp11l
-0.48810304	1	0.0015479	5	Usp48
-0.745515705	1	0.0015508	5	Slc31a1
-0.413677797	1	0.0015579	5	Itgb1bp1
1.44078882	1	0.0015638	5	Tbc1d8
-0.913584408	1	0.0015741	5	Stard3
-0.481026815	1	0.0016089	5	Usp14
-0.533324976	1	0.001611	5	Chordc1
-0.523101835	1	0.0016146	5	Bre
0.346127535	1	0.0016153	5	Rpl37
-0.327649271	1	0.0016193	5	Myo1f
0.813371681	1	0.0016242	5	Fig4
-0.43720449	1	0.0016298	5	Cops8
0.340952663	1	0.00165	5	Rhog
-0.463456196	1	0.0016547	5	Abcc1
-0.395200345	1	0.0016548	5	Lasp1
1.497940082	1	0.0016551	5	Plac8
0.504352357	1	0.0016589	5	Rpl36
1.751016047	1	0.0016825	5	Rogdi
-0.751217023	1	0.0016862	5	Tbc1d9b
0.606584877	1	0.0016891	5	Sod1
-0.456198877	1	0.0017097	5	Osbpl8
0.754512381	1	0.0017183	5	Atp11b
0.535327415	1	0.0017408	5	Hnrnpc
-0.883588477	1	0.0017444	5	Atg7
-0.369529051	1	0.0017581	5	Stau1
-0.366257621	1	0.0017898	5	Klf4
-0.294513173	1	0.0017959	5	Ccdc23
0.351012352	1	0.0018112	5	Pfdn5
-0.364343513	1	0.0018257	5	Grcc10
-0.526269984	1	0.0018307	5	Atraid
-0.297926292	1	0.001856	5	Ccpg1
-0.408189963	1	0.0018643	5	Wdr91
-0.351111216	1	0.0018672	5	Kcnn4
-0.361633174	1	0.0018787	5	Zbtb7a
-0.299630095	1	0.0019201	5	Nap1l4
-1.203032582	1	0.0019279	5	Hes1
0.555100467	1	0.0019479	5	Fmnl1
-0.710856368	1	0.0019534	5	Tmem223
-1.218511167	1	0.0019653	5	Fmnl3
-0.33545956	1	0.0020092	5	Apoa1bp
-0.48953225	1	0.0020382	5	Psmd1
-0.665251548	1	0.0020614	5	Ndufa9
0.589412798	1	0.0020646	5	Cnn2
-0.86410027	1	0.0020794	5	Pcbd2
-0.440743452	1	0.0021228	5	Atp2a2
-0.964561988	1	0.0021251	5	Tgfbrap1
-1.216286265	1	0.002138	5	Kcnk13
-1.105346096	1	0.0021395	5	Rsad2
0.698706747	1	0.0021703	5	Etv6
-0.30662056	1	0.0021705	5	Manf
0.260294458	1	0.0021793	5	Sumo1
-0.437359724	1	0.0021805	5	Psen2
-0.565757673	1	0.0021862	5	Med10
-0.563971833	1	0.0022141	5	Mgea5
-0.260683229	1	0.0022165	5	Peli1
-0.474746159	1	0.00222	5	Jmjd6

0.478276647	1	0.0022343	5	Arpp19
-0.91274685	1	0.0022364	5	Topors
-0.526980969	1	0.0022731	5	Errfi1
-1.490847303	1	0.0022742	5	Gpx3
-0.985078522	1	0.0022863	5	Slc35e4
0.388410909	1	0.002313	5	Eif2s2
-1.27192721	1	0.002329	5	Gbp7
-0.928840069	1	0.0023493	5	Cdr2
-0.814944708	1	0.0023564	5	Slc25a46
-0.359897378	1	0.0023618	5	Tnfaip8l2
-0.309022784	1	0.0023863	5	Tuba1c
-1.271112289	1	0.0024169	5	Agpat2
-0.320765789	1	0.0024317	5	Rab1
1.041204778	1	0.0025023	5	Trim11
-0.667071703	1	0.0025066	5	Mob2
0.644819279	1	0.0025223	5	Mir684-1
-0.375979575	1	0.0025838	5	Tmx1
-0.398745545	1	0.0025854	5	Rbm47
-0.873359862	1	0.0025923	5	Cpsf1
-0.545173908	1	0.0025976	5	Wasf2
-0.554753965	1	0.0025994	5	Dpm2
-0.343745196	1	0.0026275	5	Casp1
-0.722004479	1	0.0026369	5	Nfam1
0.929930959	1	0.0026507	5	2810008D09Rik
-0.652108665	1	0.0026724	5	Cnot8
-0.29322768	1	0.0027468	5	Spcs1
-0.876181766	1	0.0027538	5	Extl3
-0.693977745	1	0.0028076	5	B3gnt2
0.940090152	1	0.0028092	5	Phlpp1
-0.353150454	1	0.0028413	5	Mar.02
-0.548179246	1	0.0028439	5	Rraga
-0.301460802	1	0.0028503	5	Akap8l
-0.838010539	1	0.0028592	5	Arhgef12
-0.506760578	1	0.0028695	5	Lcp2
1.09054527	1	0.0028798	5	Ms4a4c
-0.983595981	1	0.0028821	5	Cant1
0.803284533	1	0.0029174	5	Slc38a1
0.963770701	1	0.0029537	5	Bri3bp
-0.751239494	1	0.0029643	5	Agpat5
-0.762885219	1	0.0029737	5	Tnfrsf11a
-1.105840892	1	0.0030038	5	Vps13c
-0.515545799	1	0.0030318	5	Cav2
-0.774144593	1	0.0030483	5	Calu
-0.704458272	1	0.0030642	5	Dmxl2
-0.861236058	1	0.0030848	5	Fam160b1
0.549587342	1	0.0031085	5	Gm15421
-0.2592313	1	0.003134	5	Mknk1
-0.532427859	1	0.0031384	5	Rtcb
-0.376466897	1	0.0031675	5	Fermt3
-0.472993782	1	0.0031817	5	Ifi203
0.453447576	1	0.003199	5	Tmem161b
-0.283833032	1	0.0032095	5	Dgkz
-1.280969137	1	0.0032233	5	Nr1h3
-0.370269674	1	0.0032803	5	Eid1
-0.413766705	1	0.0033266	5	Fnbp1l
-0.40421189	1	0.0033284	5	Sec24b
-0.729262666	1	0.0034062	5	Ccdc132
-0.402802242	1	0.0034309	5	Rap1gds1
-0.560463819	1	0.003432	5	Dtx4
-1.09036639	1	0.0034603	5	Spn
0.795814429	1	0.0034661	5	Fgfr1op

-0.922189237	1	0.0034692	5	Ckap5
-0.355777776	1	0.0034713	5	Nudc
-0.422296407	1	0.003487	5	Slc39a10
-0.875136197	1	0.0035024	5	Fhl3
0.742737013	1	0.0035106	5	Wdfy4
-0.515021125	1	0.0035333	5	Gapvd1
-0.657034097	1	0.0035432	5	Dhrs7
1.094975604	1	0.0035556	5	Uap1
0.856981564	1	0.0035601	5	Rusc1
-0.777047573	1	0.0035909	5	Evi5
-0.890459322	1	0.0036108	5	Abhd4
-0.319874051	1	0.0036167	5	Alkbh5
-0.546514939	1	0.0036237	5	Lamtor3
-0.426430895	1	0.003645	5	Lman2
-0.311483772	1	0.0036505	5	Fam120b
-0.71632122	1	0.0036648	5	Spp1
-0.846908203	1	0.0036668	5	Lars
-0.389646576	1	0.0037311	5	Wbp5
-0.803847538	1	0.0037452	5	Socs6
-0.444618386	1	0.0037663	5	Ptov1
-1.217827359	1	0.0037824	5	Sqrdl
-0.435155737	1	0.0037994	5	D19Bwg1357e
0.706453884	1	0.003814	5	H2-DMb1
-0.617992024	1	0.0038208	5	Pisd
-1.380806367	1	0.003827	5	Dok2
-0.351931492	1	0.0038394	5	Cln5
-0.285767385	1	0.0038691	5	St6galnac4
1.17043282	1	0.0038724	5	Irf4
0.660937736	1	0.0038728	5	Arl2bp
1.112598933	1	0.003903	5	Ppie
-0.738228029	1	0.0039604	5	Msrb1
-0.480062212	1	0.0039744	5	Fam63b
-1.040352293	1	0.0039909	5	Slc7a8
-0.46806709	1	0.0040405	5	Tubb2a
-0.508577751	1	0.0040571	5	Drg1
-0.369447849	1	0.0040734	5	Dync1h1
0.434742793	1	0.0041183	5	Nubp2
-0.443307685	1	0.0041849	5	Ubap1
-0.255055295	1	0.0041979	5	Romo1
-0.288923097	1	0.0042141	5	Znfx1
-0.894892391	1	0.0042924	5	Cklf
-0.739803453	1	0.0043111	5	Mrpl28
-0.276293344	1	0.0043917	5	Plekho2
-0.733552646	1	0.0043936	5	Tbk1
-0.392162156	1	0.0044342	5	Mtpn
-0.621777922	1	0.0044403	5	Itpripl1
-0.729271894	1	0.004451	5	Mmp12
-0.374910185	1	0.0044612	5	Lpxn
-0.29845102	1	0.004463	5	Rabep1
-0.540868939	1	0.0044695	5	Rars
-0.336396834	1	0.0044878	5	Rbms1
-0.456707245	1	0.0045402	5	Tmem55b
-0.335953727	1	0.0045556	5	Ankhd1
-0.310930827	1	0.0045729	5	Zfr
-0.498155842	1	0.004582	5	Smim15
-0.367487421	1	0.0045858	5	Adrbk1
-0.313652634	1	0.0046004	5	Pfkfb3
-0.266146285	1	0.0046281	5	Prdx1
-0.356924224	1	0.0047064	5	Plcl2
-0.646769498	1	0.0047262	5	Ints1
-0.946222277	1	0.0047315	5	Nt5dc2

0.872004676	1	0.0048059	5	Vrk1
-0.856304123	1	0.0048505	5	Arfrp1
-1.015027158	1	0.0048978	5	Smim20
0.307803744	1	0.0049112	5	Prcp
0.914264389	1	0.0049775	5	Map4k4
-0.323932569	1	0.0050062	5	Gpr137b-ps
0.590074072	1	0.0050338	5	Cox17
-0.36448843	1	0.0050437	5	Brpf1
-1.631235218	1	0.0051227	5	Cp
-0.361900909	1	0.005129	5	Atp6v1d
-0.856840968	1	0.0051406	5	Sord
-0.607256445	1	0.0051489	5	Sh3bp2
0.481051598	1	0.005151	5	Ndufa6
-0.314824812	1	0.0052096	5	Ptprj
-0.671825578	1	0.0052569	5	Naip2
-0.943810816	1	0.005261	5	Heatr5a
-0.57542288	1	0.0052931	5	Cln3
-0.332219024	1	0.0053905	5	Samd9l
0.284424107	1	0.0054227	5	Sugt1
-0.748337831	1	0.0054333	5	Nfxl1
0.408310213	1	0.0054343	5	Dazap2
-0.848301207	1	0.0054378	5	Srm
-0.498286563	1	0.0055333	5	Gda
-0.262592601	1	0.0055403	5	Ddx21
-0.870205423	1	0.005546	5	Ube4b
-0.796421313	1	0.0055702	5	Fnta
-0.253788309	1	0.0055958	5	Fes
-0.260107131	1	0.0056563	5	Arl6ip1
-0.463226788	1	0.0057261	5	Mad2l2
0.787395073	1	0.0057657	5	Cdkn1b
-0.648735294	1	0.0057709	5	Emc1
-0.669404998	1	0.0057735	5	Ptpn7
-0.752330431	1	0.0058283	5	Tbc1d14
-0.646535492	1	0.0058713	5	Fech
0.698444837	1	0.0059016	5	Cpne2
-0.669491323	1	0.005958	5	Irf3
-0.487233857	1	0.0059793	5	Acsl1
-0.534866677	1	0.0059845	5	Pla2g16
-0.250771415	1	0.0060376	5	Ahsa1
-0.695268568	1	0.0061131	5	Bfar
-0.341709793	1	0.0061213	5	Nkap
-0.266553283	1	0.0061298	5	Bscl2
-0.346534219	1	0.0061682	5	Znhit1
-1.205368526	1	0.0062131	5	Slc38a6
1.150159401	1	0.0063309	5	Itpr1
-0.254359996	1	0.0063497	5	Suco
-1.465480922	1	0.0063965	5	Il10
0.700511578	1	0.006414	5	Smg5
-0.3619125	1	0.006424	5	Arhgap5
-0.613135998	1	0.0064402	5	Sipa1
0.802551931	1	0.0064416	5	Eif4a2
-0.903593515	1	0.0064703	5	Slc39a11
0.512129665	1	0.0064791	5	Selplg
-0.379029937	1	0.0065254	5	Gtf2i
-0.702406915	1	0.0065593	5	Sbf2
0.371999603	1	0.0065942	5	Cox6c
0.985981516	1	0.006612	5	Utp18
-0.875041673	1	0.0066123	5	Ncoa2
-0.750840918	1	0.0067386	5	Gclm
-0.333510068	1	0.0067713	5	Osm
-1.023228014	1	0.0067849	5	Rnf128

-0.321430338	1	0.0068594	5	Tcirg1
-0.498569503	1	0.0068971	5	Chmp2b
-0.595211128	1	0.0069838	5	Plekha3
-0.503862595	1	0.0070057	5	Cd72
-0.324374467	1	0.0070395	5	2810004N23Rik
-0.306587071	1	0.0071031	5	Otulin
-1.42266004	1	0.0071122	5	Lyl1
-0.626390567	1	0.0071322	5	Lemd2
-0.311144035	1	0.0071826	5	Ppm1g
0.766631887	1	0.0072799	5	Aebp2
1.024568086	1	0.0072996	5	Rbm38
-0.339649346	1	0.0073162	5	Nipbl
-1.17376595	1	0.0073285	5	Zfp704
-0.259741023	1	0.007333	5	Eftud2
0.509708403	1	0.0073442	5	Rpl38
-0.903090677	1	0.0073822	5	Enox2
-0.449053744	1	0.0073882	5	Ggh
1.024236851	1	0.0074572	5	Rfxap
-0.341900492	1	0.0074758	5	Arrb2
-0.256435805	1	0.0075406	5	Fam129b
-1.139190846	1	0.0075702	5	Fam213b
-0.277275162	1	0.0075846	5	Abcg1
-1.131403407	1	0.0075966	5	Msl3
-0.431264257	1	0.0076416	5	Commd9
0.334678591	1	0.0076897	5	Uqcrh
-0.259388694	1	0.0077187	5	Myo9b
-0.428715017	1	0.0077309	5	Mgat2
-0.338600405	1	0.0077467	5	Tfe3
0.776532535	1	0.0077729	5	Mllt6
-0.279058858	1	0.0078658	5	Mrpl42
-0.298467502	1	0.0078684	5	Mapkap1
0.850845529	1	0.0080518	5	Fyn
-0.629824958	1	0.0080555	5	Adi1
-0.735698375	1	0.0080645	5	Tpcn1
-0.944456112	1	0.0080707	5	Inpp5k
-0.34802624	1	0.0080824	5	2210016F16Rik
-0.520123419	1	0.0081272	5	Mrpl48
-0.949310237	1	0.0081733	5	Msra
-0.581886823	1	0.0082564	5	Gxylt1
-1.041524489	1	0.0082939	5	2510009E07Rik
0.458123535	1	0.0083213	5	Basp1
-0.489860264	1	0.0084064	5	Rps19bp1
-0.693033886	1	0.0084212	5	Usp5
-0.300968167	1	0.0084848	5	Ralgapb
-0.745014768	1	0.0085636	5	1110057K04Rik
-0.323267726	1	0.00857	5	Fxyd2
-1.030656157	1	0.0086613	5	Tm7sf3
0.378276972	1	0.0086636	5	Tpm4
-0.403585196	1	0.0086872	5	Ids
-0.455949148	1	0.0087299	5	Slc48a1
0.318276657	1	0.0087559	5	Pim1
0.675211843	1	0.0088325	5	Agpat4
-0.369435966	1	0.008852	5	Ralb
-0.625071284	1	0.0088593	5	Ptpmt1
0.809554458	1	0.0088731	5	Sin3a
0.981434623	1	0.0088999	5	Ccser2
-0.345671051	1	0.0089276	5	Parp14
-0.318238917	1	0.0089618	5	Dynll2
0.622899422	1	0.0089914	5	Tmem131
-0.612443871	1	0.0090001	5	Tmem242
-0.626644213	1	0.0090162	5	Pmm2

0.698612996	1	0.0091224	5	Smchd1
-0.7134104	1	0.0091565	5	Snapin
-0.562133395	1	0.0091643	5	Nus1
-0.319116425	1	0.0092024	5	Napg
-0.272869255	1	0.0093125	5	Vapa
-0.267219949	1	0.0093625	5	Cd164
-0.766693564	1	0.0093699	5	Dnajc4
-0.925938821	1	0.0093944	5	Ilkap
-0.943463454	1	0.009397	5	Sep.08
-0.500093459	1	0.0093993	5	Tmem120a
0.548063206	1	0.0094012	5	Uvrag
-0.472073527	1	0.0094038	5	Spns1
0.287390474	1	0.0094684	5	Cmip
-0.301314509	1	0.0095071	5	Ggnbp2
-0.439025471	1	0.0095324	5	Parvb
-0.29976581	1	0.0095387	5	Fam168a
-0.480771477	1	0.0095413	5	Arhgef2
-0.657193204	1	0.0095781	5	Agtrap
-1.135614909	1	0.00958	5	Ttyh2
-0.458511991	1	0.009587	5	Tm2d3
-0.253973414	1	0.0095993	5	2210013O21Rik
0.550110106	1	0.0096134	5	Taf10
0.474248459	1	0.0096382	5	Psma7
-0.362896909	1	0.0096752	5	Srgap2
-0.991248842	1	0.0096774	5	Il18bp
-0.381006709	1	0.0096885	5	Med8
-0.630946384	1	0.0096948	5	Srpr
-0.276188274	1	0.0097369	5	Rpl7l1
0.622566296	1	0.00974	5	Prdx6
-0.621376933	1	0.0098245	5	Hbegf
1.463843116	1	0.0098707	5	Serpinb9
-0.502958041	1	0.0099918	5	Strn4
2.030581647	1.09E-40	8.18E-45	6	Klrc1
2.694960433	7.27E-31	5.44E-35	6	Cd3e
3.35997087	1.01E-28	7.53E-33	6	Cd3g
2.642595667	7.41E-26	5.55E-30	6	Ptprcap
2.49453385	6.05E-25	4.53E-29	6	Gata3
3.100310605	7.57E-25	5.67E-29	6	Cd96
2.862403485	5.26E-24	3.94E-28	6	Itk
2.786529187	5.35E-24	4.01E-28	6	Fasl
1.872914424	7.87E-24	5.90E-28	6	Bcl11b
2.172726167	1.93E-23	1.45E-27	6	Gimap3
2.63386361	3.05E-23	2.29E-27	6	Sep.01
2.470757751	4.06E-21	3.04E-25	6	Klre1
2.055125118	2.98E-19	2.23E-23	6	Ikzf3
2.751684564	6.17E-19	4.62E-23	6	Ms4a4b
2.856070665	1.69E-18	1.27E-22	6	Cd247
3.229915294	2.62E-18	1.96E-22	6	Cd3d
2.954288682	2.71E-17	2.03E-21	6	Sh2d2a
2.311410219	2.72E-17	2.04E-21	6	Skap1
2.074984821	8.13E-17	6.09E-21	6	Ets1
3.144490941	2.20E-16	1.65E-20	6	Lat
2.376754827	1.59E-15	1.19E-19	6	Xcl1
2.651876072	2.57E-15	1.93E-19	6	Gimap4
2.421922008	2.74E-15	2.05E-19	6	Lck
2.246544251	1.32E-13	9.92E-18	6	Ifng
1.860891452	2.69E-13	2.01E-17	6	Acap1
1.619796947	2.81E-13	2.10E-17	6	Clnk
1.771904845	7.51E-13	5.62E-17	6	Faah
2.811666212	1.53E-11	1.14E-15	6	Il2rb
1.416841343	2.27E-11	1.70E-15	6	Ltb

2.670382995	3.95E-11	2.96E-15	6	Cd8a
2.880247093	4.08E-11	3.06E-15	6	Icos
2.293829638	5.83E-11	4.36E-15	6	Actn2
2.900505758	1.06E-10	7.97E-15	6	Thy1
2.841790965	6.32E-10	4.74E-14	6	Ctsw
2.074203635	1.94E-09	1.45E-13	6	Ppp1r16b
2.225982458	1.97E-09	1.47E-13	6	Ablim1
-0.735161222	3.77E-09	2.82E-13	6	Ftl1
0.785863672	5.14E-09	3.85E-13	6	Tmsb10
2.975413515	7.78E-09	5.83E-13	6	Gzmb
-0.885313182	8.36E-09	6.26E-13	6	Ctss
1.855781051	8.88E-09	6.65E-13	6	Ikzf2
-1.544649932	1.25E-08	9.40E-13	6	Tgfbi
3.141747981	1.30E-08	9.74E-13	6	Nkg7
2.565541038	2.44E-08	1.83E-12	6	Pdcd1
-1.481660382	4.83E-08	3.61E-12	6	Pltp
2.647850982	5.25E-08	3.94E-12	6	Cxcr6
-0.743744726	2.14E-07	1.60E-11	6	Ctsb
-1.272091013	2.76E-07	2.07E-11	6	Ctsh
1.661489559	3.82E-07	2.86E-11	6	Pkp3
-1.32557845	4.49E-07	3.37E-11	6	Lamp2
2.463269228	5.72E-07	4.28E-11	6	Ccl5
1.283047891	5.79E-07	4.34E-11	6	Cd82
-1.086870716	7.24E-07	5.42E-11	6	Cd68
2.150005505	1.36E-06	1.02E-10	6	Ptpn22
2.013542227	1.99E-06	1.49E-10	6	AW112010
2.138055852	2.35E-06	1.76E-10	6	Gramd3
2.120311878	2.52E-06	1.89E-10	6	Zap70
1.807303338	3.22E-06	2.41E-10	6	F2r
-0.907366186	4.65E-06	3.48E-10	6	Ctsz
-1.001639665	9.31E-06	6.97E-10	6	Lilrb4
-1.083071657	1.08E-05	8.07E-10	6	Hexa
-0.869004567	1.39E-05	1.04E-09	6	Lyz2
2.292690876	1.54E-05	1.15E-09	6	Ctla2a
2.145928427	1.90E-05	1.42E-09	6	Camk4
1.589220038	2.27E-05	1.70E-09	6	Kif21b
0.459177862	2.47E-05	1.85E-09	6	Rps15a-ps4
-0.81922866	2.85E-05	2.14E-09	6	Cst3
-1.028036392	3.80E-05	2.85E-09	6	C1qa
-1.189803228	3.93E-05	2.94E-09	6	Cd63
-1.066632049	5.54E-05	4.15E-09	6	Wfdc17
2.285520881	5.95E-05	4.46E-09	6	Tbc1d10c
-0.863277095	7.36E-05	5.51E-09	6	Cybb
-1.60527207	7.81E-05	5.85E-09	6	Nrros
-0.787833415	8.67E-05	6.49E-09	6	C1qb
-1.272779133	0.000114105	8.55E-09	6	Basp1
-1.111713431	0.000125493	9.40E-09	6	Cxcl16
-0.879381247	0.000131387	9.84E-09	6	Ctsa
1.530175097	0.000158419	1.19E-08	6	P2ry10
0.571975736	0.000162631	1.22E-08	6	H2-K1
-1.488338771	0.000199643	1.50E-08	6	Ifi30
-1.354400997	0.000242298	1.81E-08	6	Emr1
-1.143760013	0.000261258	1.96E-08	6	Ifitm2
-0.9932566	0.000273808	2.05E-08	6	Alox5ap
-0.708685305	0.000301571	2.26E-08	6	Tyrobp
-0.839670545	0.000379741	2.84E-08	6	Grn
2.004154065	0.000474292	3.55E-08	6	Cd2
-1.856384265	0.000481627	3.61E-08	6	Slc11a1
-1.059774107	0.000563534	4.22E-08	6	App
-1.707909957	0.000627902	4.70E-08	6	Tlr2
-1.036914023	0.00065498	4.91E-08	6	Sdc3

-1.041363242	0.000722255	5.41E-08	6	Mpeg1
1.832381014	0.000731135	5.48E-08	6	Lax1
-1.031559801	0.000799816	5.99E-08	6	Atp6v0b
**-0.971176513**	**0.000815887**	**6.11E-08**	**6**	**Csf1r**
0.700253034	0.000821725	6.15E-08	6	Gm6251
-0.836759305	0.000847203	6.35E-08	6	Anxa5
-0.90213342	0.000911797	6.83E-08	6	Cd14
-1.459432185	0.000977504	7.32E-08	6	Cndp2
1.05594449	0.00120163	9.00E-08	6	Rasgrp1
-1.09990862	0.001349046	1.01E-07	6	Ms4a7
-1.49251263	0.001810358	1.36E-07	6	Pirb
1.715007837	0.001816544	1.36E-07	6	Cd28
1.944653135	0.001837245	1.38E-07	6	Ifi47
-1.396808075	0.001841422	1.38E-07	6	Mafb
-0.755379389	0.002070501	1.55E-07	6	Gp49a
-1.019542817	0.002288997	1.71E-07	6	Asah1
-1.265667687	0.002483274	1.86E-07	6	Ifitm3
-1.621034215	0.002824721	2.12E-07	6	H2-DMa
-0.711731035	0.003034734	2.27E-07	6	Lamp1
-1.311192646	0.003659962	2.74E-07	6	AF251705
-0.862500338	0.004125437	3.09E-07	6	Lgmn
-0.87375168	0.005272538	3.95E-07	6	Hexb
-1.00738823	0.005311885	3.98E-07	6	Gm2a
-1.165609844	0.007371489	5.52E-07	6	Trem2
0.903644178	0.007396476	5.54E-07	6	Rac2
-1.451124083	0.008403276	6.29E-07	6	Dusp3
0.457052834	0.008934727	6.69E-07	6	Rps4x
-2.008689388	0.009059665	6.79E-07	6	Glt25d1
-2.046434569	0.009620674	7.21E-07	6	Pilra
-0.890035367	0.010008829	7.50E-07	6	Psap
-1.331560589	0.010459461	7.83E-07	6	Gusb
-1.246173946	0.010810533	8.10E-07	6	Syk
-1.014768443	0.011374621	8.52E-07	6	Sirpa
0.496382083	0.012292413	9.21E-07	6	Rps15
-0.901981212	0.012298258	9.21E-07	6	Cd83
-0.734632329	0.014218158	1.06E-06	6	C1qc
-1.040796657	0.014280793	1.07E-06	6	Spi1
0.483017202	0.015688538	1.17E-06	6	Rps20
-1.377208401	0.015796574	1.18E-06	6	Sgpl1
-1.08132177	0.018535771	1.39E-06	6	Trf
-1.808486182	0.020653942	1.55E-06	6	Csf2rb
1.013355127	0.021546247	1.61E-06	6	Osbpl3
0.616145732	0.021736164	1.63E-06	6	Gm1821
2.372868304	0.023525992	1.76E-06	6	Cd8b1
-0.782705706	0.026033651	1.95E-06	6	Ctsc
0.762000156	0.027390313	2.05E-06	6	Sub1
-0.726645185	0.030312649	2.27E-06	6	Lgals3
-1.025577318	0.036046315	2.70E-06	6	Plekho2
-1.383686165	0.036448607	2.73E-06	6	Ctsl
-0.980371622	0.037412311	2.80E-06	6	Cstb
-1.510336569	0.037576744	2.81E-06	6	Ms4a6c
-1.337865795	0.038109408	2.85E-06	6	Cyfip1
1.324154553	0.040120153	3.00E-06	6	Lbh
-1.280886411	0.04092072	3.06E-06	6	Cfp
0.490852769	0.042307878	3.17E-06	6	Rpl13a
-1.135942681	0.042448144	3.18E-06	6	5430435G22Rik
-0.573327715	0.047347828	3.55E-06	6	Npc2
-0.825537767	0.05732552	4.29E-06	6	Atf3
-1.015348525	0.057482019	4.31E-06	6	Ctnna1
-0.712153227	0.05751654	4.31E-06	6	Akr1a1
-1.478039728	0.058673215	4.39E-06	6	Msr1

0.452174203	0.06326308	4.74E-06	6	Rps26
-0.967589646	0.064525849	4.83E-06	6	Timp2
-1.426830322	0.065674176	4.92E-06	6	Myof
2.946073074	0.072835703	5.46E-06	6	Gzma
-0.912068756	0.074562167	5.58E-06	6	Abca1
-0.653173621	0.075330269	5.64E-06	6	Ctsd
-0.795286347	0.083956642	6.29E-06	6	Ly86
-1.260276855	0.084839154	6.35E-06	6	Ptpre
1.282152075	0.085061556	6.37E-06	6	Rnf138
0.558503009	0.085648081	6.41E-06	6	Rplp0
1.909940296	0.089013816	6.67E-06	6	Gimap6
1.528623166	0.089914797	6.73E-06	6	Pdcd4
-0.853548293	0.091363766	6.84E-06	6	Kctd12
1.054733627	0.095019482	7.12E-06	6	Ogt
-1.6159544	0.100478857	7.53E-06	6	Fnip2
1.774065586	0.106323769	7.96E-06	6	1190002F15Rik
1.835623479	0.108038306	8.09E-06	6	Serpinb9
1.088037621	0.108781913	8.15E-06	6	Hmgb2
0.683966671	0.111507927	8.35E-06	6	Rabgap1l
-1.138945586	0.116470599	8.72E-06	6	Igf1
0.288616847	0.123505489	9.25E-06	6	B2m
-1.034160498	0.123792615	9.27E-06	6	Ccl6
0.716709853	0.123883132	9.28E-06	6	Btg1
1.868516442	0.125132692	9.37E-06	6	Grap2
-0.771222369	0.132614041	9.93E-06	6	Mt1
-0.36809319	0.13366738	1.00E-05	6	Fth1
-1.870874775	0.135491511	1.01E-05	6	Il1rn
-1.095504917	0.137335444	1.03E-05	6	Ms4a6d
-0.656070039	0.137816353	1.03E-05	6	Fcgr3
-0.920104266	0.152070667	1.14E-05	6	Pea15a
-0.665356171	0.15365845	1.15E-05	6	Gabarap
1.169996934	0.162931748	1.22E-05	6	Hcst
-1.285904335	0.164871491	1.23E-05	6	Mfsd1
1.242700678	0.168476746	1.26E-05	6	Smad7
1.449994479	0.169219182	1.27E-05	6	Dusp2
-0.834463902	0.176105491	1.32E-05	6	Got1
0.403028569	0.18226244	1.37E-05	6	Mir682
1.48470309	0.18858306	1.41E-05	6	2700094K13Rik
-0.695324612	0.189337686	1.42E-05	6	Sdcbp
-0.91226117	0.190339073	1.43E-05	6	H2-Eb1
-0.811418591	0.194459822	1.46E-05	6	Nceh1
0.662149332	0.207201707	1.55E-05	6	Aes
0.624184135	0.211710226	1.59E-05	6	Rps15a-ps6
1.050596546	0.218664129	1.64E-05	6	Ccnd2
-0.761087892	0.23716515	1.78E-05	6	Sh3bgrl
1.673003197	0.238629251	1.79E-05	6	Fam102a
-1.191216627	0.243766476	1.83E-05	6	Cd300ld
1.427382916	0.256504569	1.92E-05	6	Dgat1
0.368614152	0.258570444	1.94E-05	6	Rps5
-1.073615381	0.264571373	1.98E-05	6	Plekho1
-0.867260101	0.265550372	1.99E-05	6	H2-Aa
0.921744583	0.267608727	2.00E-05	6	Map4k2
-0.92159409	0.271849422	2.04E-05	6	Mbnl2
1.173408969	0.275767716	2.07E-05	6	Ppdpf
2.520802073	0.283984997	2.13E-05	6	Cd163l1
0.570539883	0.290027866	2.17E-05	6	Rps18
0.454209331	0.29366718	2.20E-05	6	Rpl4
1.543855059	0.293747746	2.20E-05	6	Il18rap
-1.482577777	0.294053208	2.20E-05	6	Gnaq
-0.829238663	0.30080618	2.25E-05	6	H2-Ab1
-1.109175746	0.30415365	2.28E-05	6	Cers2

-1.026197858	0.310404203	2.32E-05	6	Aif1
1.466707261	0.323125063	2.42E-05	6	Cd7
1.468646899	0.335885785	2.52E-05	6	Dgka
0.563341588	0.346911154	2.60E-05	6	Rps15a
-1.526706515	0.347347072	2.60E-05	6	Tbc1d9
-1.404823574	0.351582816	2.63E-05	6	Lst1
-1.865107135	0.356439748	2.67E-05	6	Gla
-0.974262243	0.359361285	2.69E-05	6	Atp6v1b2
0.37569789	0.368015101	2.76E-05	6	Rpl32
-0.421596807	0.368727224	2.76E-05	6	Tpp1
-0.791336327	0.373983828	2.80E-05	6	Tpd52
1.078225519	0.37830574	2.83E-05	6	Bcl2
-1.25754295	0.378830613	2.84E-05	6	S100a1
-0.751446458	0.394923354	2.96E-05	6	Marcks
-0.709652926	0.401071002	3.00E-05	6	Unc93b1
-1.317350847	0.428961125	3.21E-05	6	Ifngr2
-0.924947212	0.431907437	3.23E-05	6	Ninj1
-1.195576111	0.452906922	3.39E-05	6	Tmem106a
-1.088005711	0.463362974	3.47E-05	6	Trim25
1.176712787	0.468420178	3.51E-05	6	Mettl7a1
-1.428903333	0.475619974	3.56E-05	6	Clec7a
1.499220661	0.531675696	3.98E-05	6	Fasn
-1.215557009	0.53371134	4.00E-05	6	Pla2g7
-1.314078645	0.540949631	4.05E-05	6	Anxa1
-1.040734002	0.551340689	4.13E-05	6	Slamf9
0.796339884	0.574258105	4.30E-05	6	Rps16
0.657780103	0.577900273	4.33E-05	6	Rpl18a
-0.698939058	0.577901556	4.33E-05	6	Metrnl
-0.68777191	0.585913164	4.39E-05	6	Cxcl2
-0.56980407	0.588921025	4.41E-05	6	Atox1
-0.847920247	0.630705405	4.72E-05	6	Csf2ra
-0.692774634	0.630784756	4.72E-05	6	Plek
-0.409934232	0.63288687	4.74E-05	6	Gpx1
-0.844829758	0.633256214	4.74E-05	6	Tmed10
0.651400867	0.635669401	4.76E-05	6	Npm1
1.024497441	0.640759924	4.80E-05	6	Zfp36l2
0.617062103	0.66211583	4.96E-05	6	Mbnl1
-1.244819584	0.664599766	4.98E-05	6	Dnase2a
-1.257387026	0.667244586	5.00E-05	6	Skap2
-0.687591587	0.688745856	5.16E-05	6	C3ar1
-1.297863866	0.705116011	5.28E-05	6	Scarb2
-1.204638633	0.709139159	5.31E-05	6	Lpl
-0.765384429	0.721665574	5.40E-05	6	Lmna
0.78700084	0.732355207	5.48E-05	6	Prkar1a
-1.704457202	0.747780007	5.60E-05	6	Nuak2
0.561657149	0.756098389	5.66E-05	6	Gm4832
-1.397034485	0.773982694	5.80E-05	6	Pid1
-0.926785354	0.815447636	6.11E-05	6	Stab1
-1.592156909	0.817937624	6.13E-05	6	Cib1
-1.047426393	0.840757885	6.30E-05	6	Alcam
-1.327239425	0.851105764	6.37E-05	6	H2-DMb1
0.372733105	0.856582827	6.42E-05	6	Pfn1
-0.978791239	0.860812458	6.45E-05	6	Rab3il1
-0.731868554	0.869805226	6.51E-05	6	Abhd12
-1.561489279	0.944844285	7.08E-05	6	Emilin2
-0.859783773	0.972386823	7.28E-05	6	Tnfrsf1a
-1.432080104	0.973745688	7.29E-05	6	Tgm2
1.140592268	0.98216976	7.36E-05	6	Itgal
-0.68267668	1	7.62E-05	6	Cd74
1.176630858	1	7.62E-05	6	Snrpf
-0.750290688	1	7.67E-05	6	AI607873

-1.204088323	1	7.73E-05	6	Rbpj
-1.066609924	1	7.91E-05	6	Ncf1
0.708054694	1	7.99E-05	6	Snrpg
0.862991136	1	8.40E-05	6	Selplg
-1.449740151	1	8.41E-05	6	Acer3
1.739354853	1	8.52E-05	6	Lime1
0.618956478	1	8.54E-05	6	Coro1a
-1.194977076	1	8.63E-05	6	Gsn
0.548199448	1	8.70E-05	6	Cd52
-0.592096565	1	8.94E-05	6	Scpep1
-0.73864879	1	9.15E-05	6	Dab2
-0.863407816	1	9.29E-05	6	Nckap1l
-0.825021274	1	9.39E-05	6	Hsd17b11
-0.600985308	1	9.45E-05	6	Clta
-1.16914508	1	9.60E-05	6	Fes
0.510169058	1	9.66E-05	6	Rps21
-0.724032067	1	9.66E-05	6	Atpif1
-0.96884855	1	9.76E-05	6	Swap70
-0.820746449	1	9.81E-05	6	Creg1
-0.542432203	1	9.90E-05	6	Rtn4
-0.74035264	1	0.0001008	6	Rgs10
-0.990044632	1	0.0001021	6	Itgb5
-1.299578504	1	0.000103	6	Hck
0.693543902	1	0.0001047	6	Myl12b
-1.388189792	1	0.0001091	6	Rab11fip5
-1.297025806	1	0.0001098	6	Prcp
1.138152581	1	0.0001107	6	Cyfip2
-1.194513248	1	0.0001161	6	Cd300a
0.367183976	1	0.000117	6	Rps19-ps3
1.191935118	1	0.0001181	6	Emb
1.291690587	1	0.0001226	6	Mfng
-0.850492086	1	0.0001233	6	Ndufb9
0.496435481	1	0.0001248	6	Rpl18
-0.756581555	1	0.0001252	6	Rnf130
-0.627627033	1	0.0001263	6	Atp6ap1
-0.567216797	1	0.0001263	6	Rragc
0.78866041	1	0.0001276	6	Rbm38
-1.041556299	1	0.0001306	6	Camk1
1.493834553	1	0.0001331	6	Nfatc3
-0.825527163	1	0.0001443	6	Gns
0.467938059	1	0.00015	6	Pabpc1
-1.278083921	1	0.0001522	6	Pepd
1.315291178	1	0.0001605	6	Nedd9
1.385469974	1	0.0001607	6	Ssbp3
-0.796380714	1	0.0001638	6	Lrp1
-1.155804278	1	0.0001702	6	Rnf13
-0.878645723	1	0.0001733	6	Rnh1
-1.204442366	1	0.0001739	6	P2rx4
-0.88109197	1	0.0001744	6	Rasgef1b
-1.011760854	1	0.0001786	6	Rab31
-0.778136709	1	0.0001868	6	Pip4k2a
-0.91278743	1	0.00019	6	Apobec1
0.376318404	1	0.0001901	6	Rpl8
-1.42998061	1	0.0001953	6	Mpp1
-0.996429781	1	0.0001954	6	Ccl9
-1.313315255	1	0.0002	6	Fcgr1
-0.765606215	1	0.0002002	6	Ap3d1
-0.597416335	1	0.0002008	6	Ecm1
-0.474294613	1	0.0002044	6	Taok1
0.865196278	1	0.0002093	6	Atp11b
0.815514192	1	0.0002111	6	Rps8

-0.801678537	1	0.000222	6	Irf5
0.517093043	1	0.0002221	6	Mir703
-0.938965073	1	0.000226	6	Hpgds
-0.441583513	1	0.000228	6	Iqgap1
-1.662651381	1	0.0002319	6	Hip1
-0.714061172	1	0.0002347	6	Man2b1
0.780489927	1	0.0002486	6	Psip1
-0.673022777	1	0.0002507	6	Tubb6
-0.725178967	1	0.0002558	6	Cd81
-1.333525849	1	0.000259	6	Ralb
-1.222519756	1	0.0002636	6	Cd72
-0.687245449	1	0.0002674	6	Jmjd1c
0.644805333	1	0.0002727	6	Rps3a1
-1.221957225	1	0.0002746	6	Dpep2
-0.665971774	1	0.0002827	6	Slc6a6
-0.47295192	1	0.0002831	6	Nfe2l2
-1.696124667	1	0.0002876	6	Zmynd15
-0.476009514	1	0.0002885	6	Sqstm1
1.170611369	1	0.0003121	6	Tfam
-0.628792893	1	0.0003191	6	Clec12a
-1.261688182	1	0.0003198	6	Tnfaip2
-0.908537177	1	0.0003228	6	Fndc3a
-1.18836653	1	0.0003241	6	Ncf2
-0.641851403	1	0.0003289	6	Smdt1
-0.364941858	1	0.0003306	6	Vim
-0.896263398	1	0.0003515	6	Fuca1
-0.772644831	1	0.0003547	6	Blvrb
0.890567057	1	0.0003582	6	Gstp1
-1.283657076	1	0.0003617	6	Atp6v1c1
0.781975929	1	0.0003635	6	Ncoa2
0.998400392	1	0.000378	6	Rps2
-0.663544612	1	0.0003825	6	Plin2
-0.757193707	1	0.0003844	6	Manf
-0.522363624	1	0.0003928	6	Vamp8
-1.603771151	1	0.0003996	6	Clec4d
-1.19206535	1	0.0004231	6	Reep3
-0.650514834	1	0.0004253	6	Dync1h1
-1.038006759	1	0.0004292	6	Gdf15
-0.596323348	1	0.0004296	6	Pld4
-0.663984593	1	0.000443	6	Psma7
-1.082950355	1	0.0004553	6	Dstn
0.543830799	1	0.000477	6	Gm6654
-1.68535257	1	0.0004787	6	Atp13a2
0.710284871	1	0.0004839	6	S100a10
0.646737313	1	0.0004989	6	Ncapd2
1.008740598	1	0.0005009	6	Akirin2
1.467775484	1	0.0005021	6	Klrd1
-0.935415742	1	0.0005031	6	Anxa3
-0.71356914	1	0.0005036	6	Ncf4
-0.824920594	1	0.0005132	6	Fcrls
-0.926432563	1	0.0005135	6	Gas7
-1.365019818	1	0.0005259	6	Tns3
-1.162910515	1	0.0005299	6	Stom
-0.940647799	1	0.0005348	6	Trib1
-0.740532373	1	0.0005383	6	Ap2s1
1.261771083	1	0.0005507	6	Nsmaf
-1.236985394	1	0.000551	6	Sel1l
1.10938087	1	0.0005545	6	Dnajc9
-0.719692607	1	0.0005549	6	Zfand5
-1.251282099	1	0.000579	6	Nav1
-0.370274477	1	0.0005797	6	Fcer1g

-1.099866283	1	0.0005803	6	Spred1
-1.109962436	1	0.0005823	6	Zfp263
0.522810018	1	0.0005894	6	Gas5
0.585087672	1	0.0006004	6	Rhof
-1.723332117	1	0.0006113	6	Mmp14
-1.087790141	1	0.0006123	6	Lyz1
-1.237706101	1	0.0006193	6	Ash1l
1.155200545	1	0.0006266	6	Ubtf
0.427093605	1	0.0006448	6	Eef2
-0.868062572	1	0.0006448	6	Arl8b
0.765176376	1	0.0006474	6	Tmem154
-1.351439017	1	0.0006539	6	Sash1
1.890255504	1	0.0006573	6	Heca
-0.93044618	1	0.0006595	6	Eif4ebp1
0.632930127	1	0.0006658	6	Rps7
-1.023282752	1	0.0006726	6	Cebpa
-1.39843968	1	0.0006742	6	Vps41
0.975805822	1	0.0006881	6	Fam107b
-1.463620673	1	0.0006996	6	Plbd1
1.417956064	1	0.0007008	6	Asb6
-0.947587815	1	0.0007048	6	Plau
-0.745572749	1	0.0007072	6	Daglb
0.947424062	1	0.0007139	6	Prr3
-1.108338002	1	0.0007144	6	Cttnbp2nl
-0.530639706	1	0.00074	6	Mrpl17
-0.92761312	1	0.0007767	6	Emp1
0.385112724	1	0.0007838	6	H3f3b
-0.811174387	1	0.0007874	6	Itgam
-1.141948602	1	0.0007921	6	Lactb
-0.538281495	1	0.0007944	6	Dhrs3
-1.559843672	1	0.0007969	6	Fam20c
-0.818274718	1	0.0007982	6	Cpeb4
0.443109044	1	0.0008151	6	Rpl39
1.079791846	1	0.0008189	6	2810417H13Rik
-1.443152889	1	0.0008239	6	Map1lc3a
-0.970543797	1	0.000828	6	Pld3
-0.721708667	1	0.0008356	6	Erp29
-1.317718707	1	0.0008417	6	Pqlc1
0.317733975	1	0.0008586	6	Rpl41
-1.381051459	1	0.0008675	6	Actn1
-0.901232158	1	0.0008785	6	Dnase1l1
-1.658274443	1	0.0008793	6	Mtmr10
0.764475312	1	0.0008943	6	Nr3c1
-1.020074053	1	0.0008975	6	Tcirg1
0.298512413	1	0.0009022	6	Rps24
-0.658083681	1	0.0009029	6	Qk
0.987135895	1	0.0009033	6	Mta2
-0.604501407	1	0.0009123	6	Rnasek
-0.430543578	1	0.0009142	6	Asap1
0.851620108	1	0.0009257	6	Spn
-1.245091471	1	0.0009283	6	Clec5a
-0.845437986	1	0.000937	6	Rsu1
0.750713756	1	0.0009373	6	Stk17b
-1.120273906	1	0.0009514	6	Tmem37
-1.124373689	1	0.000953	6	Plod3
-0.719998439	1	0.0009668	6	Tcf4
-1.472730458	1	0.0009843	6	Kcnn4
-0.428100538	1	0.0010073	6	Atp2b1
-1.021466226	1	0.0010189	6	Pcyt1a
-0.764768264	1	0.001021	6	Anxa4
-0.710783735	1	0.0010254	6	Tuba1c

-1.272064084	1	0.0010259	6	Ctnnd1
1.007633682	1	0.0010344	6	Zcchc11
0.292519627	1	0.0010576	6	Rps14
-1.088092329	1	0.0010634	6	Ifnar1
-0.783700542	1	0.001066	6	Cenpb
-0.54492088	1	0.0010662	6	Pkm
0.668709744	1	0.0010727	6	Dnajb6
-1.185299043	1	0.0011059	6	Rit1
-0.8305845	1	0.0011224	6	Lair1
-0.554355298	1	0.0011298	6	Nrp1
-0.703399869	1	0.0011483	6	C5ar1
-0.944425545	1	0.0011714	6	Vat1
-0.894667511	1	0.0011717	6	Clcn7
0.664959104	1	0.0012169	6	Shisa5
-0.758609626	1	0.0012224	6	Degs1
-0.377435917	1	0.0012331	6	Atp6v1a
-0.665714814	1	0.0012457	6	Zeb2
-0.993105483	1	0.0012729	6	Dram2
-0.589344659	1	0.0012743	6	Uqcr11
1.737604784	1	0.001278	6	Cst7
-0.415824549	1	0.0012807	6	Rrbp1
-0.538105747	1	0.001284	6	Phf23
-0.437944401	1	0.001284	6	Fos
-0.584357447	1	0.0012847	6	Tnf
-0.744719067	1	0.0012853	6	Ier3
-0.723614668	1	0.001297	6	Fam21
-0.67956993	1	0.0013127	6	Dbi
-0.936689327	1	0.0013163	6	Sypl
-1.680805773	1	0.0013192	6	Stx3
-0.980690142	1	0.0013335	6	Ptpro
-0.642460912	1	0.0013604	6	Senp2
-1.10216796	1	0.0013647	6	Clec4a3
-0.889794245	1	0.0013665	6	Arhgap17
-0.607650146	1	0.0013683	6	Prkcd
-1.02914984	1	0.0013726	6	Uap1l1
-0.305321677	1	0.001374	6	Rnpep
-0.935820476	1	0.0013802	6	Cd84
-1.337936863	1	0.0013844	6	Naaa
-0.564671272	1	0.0014146	6	Csrnp1
-1.165294547	1	0.001428	6	Fcgrt
-1.430082652	1	0.001437	6	Gga3
-0.546177145	1	0.0014483	6	Nucb1
-1.10812476	1	0.0014689	6	Rhoc
-0.920908607	1	0.0014755	6	Gngt2
0.624721385	1	0.0014918	6	Serbp1
-1.012660187	1	0.0015016	6	5031439G07Rik
-1.24709335	1	0.0015048	6	Tep1
-0.56496691	1	0.0015058	6	Ap2a2
0.511199255	1	0.0015075	6	Gmfg
-1.1385822	1	0.0015198	6	Uvrag
-1.127084766	1	0.0015244	6	Acp2
-1.088082285	1	0.0015466	6	Slc29a3
-0.804626914	1	0.0015512	6	Fam129b
-1.018325763	1	0.0015897	6	P2rx7
-0.818888134	1	0.0015926	6	Tmbim1
0.836965189	1	0.0016248	6	Cish
-1.227263242	1	0.0016298	6	Pvr
-0.730703713	1	0.0016361	6	Myadm
-0.864090831	1	0.0016645	6	Slc15a3
1.333342836	1	0.0016806	6	Tshz1
0.334819529	1	0.0016861	6	Bysl

-0.535554183	1	0.0016971	6	Serpinb6a
-1.044611158	1	0.0017019	6	Pf4
-1.289686441	1	0.0017076	6	Dctpp1
1.222040488	1	0.0017105	6	Rpa1
-1.236224923	1	0.0017305	6	Gpr137b-ps
-1.333384503	1	0.0017372	6	Rnf115
-0.618068127	1	0.0017456	6	Dnajb11
0.480235571	1	0.0017477	6	S100a11
-0.689094338	1	0.0017485	6	Vamp3
-0.455771154	1	0.0017598	6	Tmx1
1.460569945	1	0.0017825	6	Itgb7
0.88923287	1	0.0017842	6	Rassf5
-1.006739398	1	0.00179	6	Mrpl54
-0.506130727	1	0.001819	6	Cuedc2
-0.451896601	1	0.0018334	6	Ptpra
1.177734432	1	0.0018591	6	Vps37b
-0.793904055	1	0.0018692	6	P2ry6
-0.687588361	1	0.0019165	6	Nfe2l1
0.749620036	1	0.0019192	6	Pmf1
-0.600705006	1	0.0019219	6	Pdia6
-0.687543094	1	0.0019477	6	Pgls
-0.35798909	1	0.0020291	6	Picalm
-1.243194359	1	0.0020345	6	Gpr137b
-1.377838989	1	0.0020428	6	Sh3pxd2a
-0.842887123	1	0.0020651	6	Glipr1
0.833193786	1	0.0020726	6	Rpl36al
-0.742539936	1	0.0021116	6	Idh2
-0.780250515	1	0.0021357	6	Txndc17
0.607650012	1	0.0021461	6	Ndufa13
-0.84552311	1	0.0021569	6	Ergic3
-0.832294501	1	0.0021725	6	Vipas39
-0.881258142	1	0.0021795	6	Frmd4b
-1.172822613	1	0.0021892	6	Eif2a
0.367277464	1	0.0022144	6	Rps3
0.823597953	1	0.0022399	6	Gltscr2
-0.585725918	1	0.0022564	6	Capg
-1.242109523	1	0.0022728	6	Cxcl1
-0.826595701	1	0.0022821	6	Filip1l
-1.123930097	1	0.0023093	6	Hist1h2bc
-1.364419573	1	0.0023254	6	Ccl12
-1.233389094	1	0.0023262	6	Ntpcr
1.225014156	1	0.0023358	6	Trp53inp1
-0.536292341	1	0.0023692	6	Hspa9
-0.820204	1	0.0023738	6	Cd86
-0.605193645	1	0.0023816	6	Farsb
-1.716436459	1	0.0023921	6	Wdfy4
1.048795244	1	0.0023936	6	Tmpo
-0.526366258	1	0.0024054	6	Rab10
-1.037415235	1	0.0024234	6	Myo9a
-1.379118867	1	0.0024289	6	Hbp1
-0.447528091	1	0.0024415	6	Chchd1
-1.048734748	1	0.0024446	6	Mitf
-0.309719299	1	0.0024512	6	Emp3
-0.715086946	1	0.0024615	6	Atp6v0a1
-0.404312822	1	0.0024693	6	Fam105a
-1.291373589	1	0.0024949	6	Rufy1
-0.65185705	1	0.0025041	6	Brk1
-0.551350336	1	0.0025525	6	St3gal1
1.412406386	1	0.0025631	6	Rora
-0.996633597	1	0.0025767	6	Galc
-1.55237584	1	0.0026014	6	Fn1

1.158375269	1	0.0026244	6	Rapgef1
-1.028333159	1	0.0026716	6	Plk2
-0.957855006	1	0.0027037	6	Selm
-1.25681596	1	0.0027085	6	Ephx1
-0.625285166	1	0.0027146	6	Rab11a
0.667654792	1	0.0027563	6	Eml4
-0.400045654	1	0.0027645	6	Rel
-1.453126062	1	0.0027835	6	Clec4n
-1.295257268	1	0.0028392	6	Ptplad2
-0.883669901	1	0.0028611	6	Ndufb10
-1.335359738	1	0.0029615	6	Sh3bp2
-0.366344712	1	0.0030056	6	Cyba
0.894846738	1	0.0030129	6	Lbr
-0.872253939	1	0.0030361	6	Cpne3
-0.544084611	1	0.0030379	6	Sh3pxd2b
0.935993829	1	0.003063	6	Per1
-0.615343135	1	0.003077	6	Atp6v0d1
-0.639620763	1	0.0030786	6	Plaur
-0.74717899	1	0.0030888	6	Coro1c
0.336369143	1	0.003099	6	Rps19
1.161305006	1	0.0031268	6	Ttc5
-0.455384002	1	0.0032255	6	Nfkbiz
-0.676300738	1	0.0032408	6	Dync1i2
1.113080039	1	0.0032462	6	Cd69
-0.721741818	1	0.0032693	6	Lgals3bp
-1.231371865	1	0.0032718	6	Rab5c
0.660625836	1	0.0033484	6	Cbx4
0.592697572	1	0.0033562	6	Rpl6
-0.58794742	1	0.0033782	6	Rnf149
2.146706085	1	0.0033843	6	Ly6c2
1.39182431	1	0.0033944	6	H2afv
0.510725307	1	0.0033971	6	Eif1
-1.267986883	1	0.0034185	6	Cgrrf1
-0.795183505	1	0.0034245	6	Capn2
-0.597988455	1	0.003433	6	Mef2a
0.802259268	1	0.0035086	6	Srsf7
-0.358856311	1	0.00352	6	Cltc
-0.602205374	1	0.003535	6	Dctn2
-1.509869919	1	0.0035498	6	Dok3
-0.902525539	1	0.0035997	6	Slc7a7
-0.414435519	1	0.0036377	6	Litaf
-0.334766947	1	0.0036399	6	Mcl1
-0.827823459	1	0.0036848	6	Msra
-0.336088848	1	0.003692	6	Hspa5
0.715693881	1	0.003707	6	Rpsa
-0.705544951	1	0.0037155	6	Herpud1
-0.688889611	1	0.0037243	6	Gm6377
0.363340677	1	0.0037261	6	Rpl14-ps1
-0.858398339	1	0.0037933	6	Nus1
-1.293889631	1	0.0037977	6	Mgat1
-0.420097862	1	0.0038097	6	Uqcrq
-0.401065554	1	0.0038127	6	Phb2
-0.518135379	1	0.0038712	6	Pomp
-1.057820974	1	0.0039718	6	Il4ra
0.449193359	1	0.0039759	6	Tsc22d2
-0.631887597	1	0.0039874	6	Ube2f
-0.376284979	1	0.0040059	6	Gltp
1.267783281	1	0.0040211	6	Rnf145
-0.551614579	1	0.0040452	6	Cux1
0.331067847	1	0.0040742	6	Rps11
-0.327024985	1	0.0040979	6	Acaa1a

1.057234732	1	0.0041473	6	Stat4
-0.566296605	1	0.0042065	6	Smim7
-1.198961894	1	0.0042131	6	Mdfic
-0.906207872	1	0.0043059	6	Ehd1
-0.748595834	1	0.0043134	6	Wwp1
-1.558639892	1	0.0043141	6	Lima1
-0.372151951	1	0.0043632	6	Atp6v1e1
-0.554480009	1	0.0044031	6	Abcg1
-1.488416924	1	0.0044234	6	Rbm47
-0.404633547	1	0.004485	6	Rnase4
-0.493623533	1	0.0044931	6	Exoc5
-0.652863067	1	0.0045685	6	Eif1a
-0.342012845	1	0.0045837	6	Ano6
-0.585339104	1	0.0045937	6	Lamtor1
-1.438331571	1	0.004648	6	Syngr1
-0.476914324	1	0.0046568	6	Anapc13
-0.797482659	1	0.0046677	6	Tnip1
-1.378300989	1	0.0046916	6	Cdr2
-0.431091606	1	0.0046974	6	Rala
-1.403915355	1	0.0046992	6	Igsf6
-0.598005576	1	0.0047099	6	Lipa
-0.619114106	1	0.0047126	6	Myo5a
-0.412214969	1	0.0047863	6	Fnip1
-0.736378354	1	0.0047919	6	Diap2
-0.607071408	1	0.0048156	6	Fcgr2b
-1.153800376	1	0.0048174	6	Fh1
-1.197752504	1	0.0048238	6	Slc39a1
-0.67865514	1	0.0048308	6	Il10rb
-1.068680742	1	0.0048564	6	Rgl1
-1.05674391	1	0.0049109	6	Hs6st1
-0.35238992	1	0.0049699	6	Chmp2a
-0.684681596	1	0.0049793	6	Lrrc59
0.477855895	1	0.0049828	6	Tnfaip3
-0.442667642	1	0.0051259	6	Zfp36
-0.636579387	1	0.0051754	6	Hpgd
-1.356268935	1	0.0051909	6	BC028528
0.83490903	1	0.0052028	6	Emg1
-0.776563286	1	0.0052103	6	Mef2c
-0.45994144	1	0.0052367	6	Wasf2
-1.468270791	1	0.0052537	6	Cd300lf
-0.813825749	1	0.0053514	6	Aprt
-0.359454307	1	0.0053566	6	Arl4c
-0.577291101	1	0.0053834	6	Casp4
-1.068822845	1	0.0053838	6	Il1b
0.868874381	1	0.0054125	6	Ikzf1
-0.597318655	1	0.005499	6	Mapre2
0.566386562	1	0.0055037	6	Eif3h
-0.49866488	1	0.0055284	6	Snap23
-0.630738026	1	0.0055671	6	Maf1
-0.578369274	1	0.0055709	6	Eps8
1.020533909	1	0.0055778	6	Ptpn7
-1.40497338	1	0.0056032	6	Bst1
-0.814690651	1	0.0056045	6	Snx1
-0.877032845	1	0.0056251	6	Rrp1
-1.0317269	1	0.0056278	6	Tlr13
0.905476786	1	0.0056425	6	Elf1
-1.146964235	1	0.0056711	6	Nagk
-0.805129795	1	0.0056799	6	Cnih4
-0.915181858	1	0.0057382	6	Psmd7
-0.478259638	1	0.0058291	6	Lims1
-0.714143521	1	0.005839	6	Cic

-0.65677854	1	0.0058485	6	Rhob
1.622909807	1	0.0058906	6	D16Ertd472e
-0.794759585	1	0.0059012	6	Snx30
-0.711317776	1	0.0059554	6	Slc8b1
-0.534762211	1	0.0059689	6	Pip5k1c
1.078032921	1	0.0059699	6	Ints6
0.722048071	1	0.0059751	6	Vps72
-1.418921993	1	0.0060436	6	Fgr
-0.48066206	1	0.0060613	6	Ehd4
-0.67267899	1	0.006081	6	Tgfbr1
0.737611419	1	0.0061122	6	Nr1d1
0.56754501	1	0.0061141	6	Prkdc
0.847187606	1	0.0061258	6	Alyref
-0.825410109	1	0.0061767	6	Ppp4r2
0.402224665	1	0.0061851	6	Rpl7
-0.953641719	1	0.0062054	6	Plin3
1.105574576	1	0.006218	6	Rmnd5b
-0.864322563	1	0.0062215	6	Renbp
0.610229023	1	0.0062414	6	Cnn2
-0.317630493	1	0.0062582	6	Ptms
-0.371358911	1	0.0063387	6	Sat1
-0.734163898	1	0.0063495	6	Pon3
1.028009666	1	0.0063657	6	2410004B18Rik
-1.132260738	1	0.0063871	6	Nrp2
-1.159607389	1	0.0063884	6	Gabarapl1
1.510873921	1	0.0063953	6	Sep.06
-1.187859864	1	0.0064354	6	Zak
0.821406126	1	0.0064639	6	Pla2g16
-0.490129617	1	0.0064867	6	Il10ra
-0.709133294	1	0.00657	6	Cd93
-0.516223047	1	0.0066267	6	Wdr26
0.998124598	1	0.0066574	6	Satb1
-0.927133743	1	0.0066601	6	Irf1
-0.57080552	1	0.006672	6	Atp7a
-0.354455918	1	0.0066871	6	Ywhae
-1.159689449	1	0.0066972	6	Folr2
0.755283443	1	0.0067041	6	Srsf3
0.359851889	1	0.0067522	6	Srgn
-0.902498223	1	0.0067972	6	Cd302
-0.831189468	1	0.0068013	6	Mrc1
0.984403376	1	0.0069642	6	Crbn
-1.270950874	1	0.0070167	6	Dusp16
-0.384201282	1	0.00705	6	Sdf2
-1.223218278	1	0.007138	6	Cdc34
-0.388021057	1	0.0071578	6	Map7d1
-0.523008541	1	0.0071759	6	Baz2b
-0.927133743	1	0.0071882	6	Rap2a
0.333167091	1	0.0072426	6	Hnrnpf
-0.792420081	1	0.0072702	6	Elovl1
0.449840618	1	0.0073761	6	Rplp2
-0.419541468	1	0.0073789	6	Ccrl2
-1.136779113	1	0.0073958	6	Adam15
-0.422997008	1	0.0074362	6	Nudt9
-1.101699474	1	0.0075438	6	Bcl3
-0.82591799	1	0.0076093	6	Usp8
-0.670777456	1	0.0076139	6	Ndufs8
0.689288717	1	0.007636	6	Cmc1
-1.439347251	1	0.0076361	6	Mgrn1
-0.2828752	1	0.0076731	6	Mrpl52
0.614828676	1	0.0077848	6	Nfatc1
-0.452764165	1	0.0077849	6	Setd2

-0.649794979	1	0.0078363	6	Rpain
0.591036414	1	0.00793	6	Ifngr1
-1.018413294	1	0.0079704	6	Rab32
-0.458299694	1	0.0080273	6	Sepp1
-0.767267454	1	0.008076	6	Axl
-1.038675519	1	0.0080938	6	Srsf9
-0.718916591	1	0.0081705	6	Psma6
0.844907959	1	0.0081817	6	Hk1
-0.352625998	1	0.008208	6	Ctnnb1
1.050446032	1	0.0082099	6	Dnajc1
-0.584709181	1	0.0082893	6	Grb2
-0.671995717	1	0.0082971	6	Ccl2
-0.638105775	1	0.0083111	6	Nampt
-0.390536975	1	0.008349	6	Ech1
-1.408366401	1	0.0083651	6	Slc37a2
-0.364521876	1	0.0084823	6	Ccl3
-0.941470441	1	0.0085215	6	Myo9b
-0.389095027	1	0.0085528	6	Mir692-1
0.663841605	1	0.0085595	6	B3glct
0.739165817	1	0.0086084	6	Orai2
0.532880391	1	0.0086214	6	Rpl37
0.985717367	1	0.0086714	6	Alad
-0.893358833	1	0.0086851	6	Mrpl11
-1.404772865	1	0.0087321	6	Kif1c
-0.300391224	1	0.0087662	6	Glud1
-1.001790799	1	0.008792	6	Mtfr1l
-0.811796286	1	0.0087977	6	Cebpg
-0.290886719	1	0.0088539	6	Atp6ap2
-0.350271275	1	0.00893	6	Stard3
-0.407081196	1	0.0089346	6	Ergic2
-0.847095028	1	0.0089854	6	Cmtm3
-0.584665229	1	0.009034	6	Cops5
0.959170452	1	0.0090673	6	Mmd
-0.674473419	1	0.0091057	6	Map3k5
0.70652421	1	0.0091285	6	Ncor1
-1.144760455	1	0.009144	6	Msmo1
-0.781122787	1	0.009152	6	Mpc2
-0.830356227	1	0.0092495	6	Spp1
-0.523167692	1	0.009268	6	Ncoa7
-1.193554118	1	0.0092761	6	Adrbk2
-1.064597467	1	0.0093116	6	Mmp12
0.540835042	1	0.0093554	6	Tmem66
-0.838271292	1	0.0093795	6	Htra2
-0.49742892	1	0.0093979	6	Gatm
-0.847606018	1	0.0094296	6	Ndrg1
-0.468244347	1	0.0094631	6	Dynlt3
0.984435161	1	0.0095294	6	Sh2b1
0.387081242	1	0.0095682	6	Rpl14
-0.820114429	1	0.0096063	6	Kdm5c
-0.616264033	1	0.0096171	6	Lmo2
-0.509344263	1	0.0096432	6	Tmem208
-0.59565018	1	0.0096662	6	Ids
-0.938384542	1	0.0097044	6	0610012G03Rik
0.409734188	1	0.0097303	6	Mir684-1
-1.034741275	1	0.0097342	6	Rnf111
-1.081400927	1	0.0097369	6	Cyp27a1
1.002469857	1	0.0097391	6	Sh3gl1
-1.240049558	1	0.0097654	6	Mfsd11
-1.45113244	1	0.0098185	6	Ttyh2
-0.458682332	1	0.0098408	6	N4bp1
1.159069568	1	0.0098671	6	Nfyb

-1.010252546	1	0.0098813	6	Atp8a1
1.77645789	1.76E-11	1.32E-15	7	Ccl7
1.416369902	1.90E-10	1.42E-14	7	Ccl2
1.361740983	6.67E-10	5.00E-14	7	Cbr2
1.320544478	5.50E-09	4.12E-13	7	F13a1
1.132567537	2.38E-08	1.78E-12	7	Mrc1
0.961772705	2.68E-07	2.01E-11	7	Ccl3
0.849597623	3.46E-07	2.59E-11	7	Cxcl2
0.881897796	7.42E-07	5.56E-11	7	Ccl4
1.271946141	1.00E-06	7.52E-11	7	Folr2
-2.299760056	1.79E-06	1.34E-10	7	Gngt2
1.607586862	2.14E-06	1.60E-10	7	Ccl12
1.492956917	5.86E-06	4.39E-10	7	Rgl1
1.250488919	6.05E-06	4.53E-10	7	Cd163
1.241755741	7.79E-06	5.83E-10	7	Lyve1
1.150771199	1.03E-05	7.69E-10	7	Gas6
1.050062613	1.41E-05	1.06E-09	7	Pf4
0.66468407	1.77E-05	1.32E-09	7	Sepp1
-1.917280834	4.83E-05	3.62E-09	7	Lpl
0.971577239	6.64E-05	4.97E-09	7	Ier3
0.969098754	7.37E-05	5.52E-09	7	C4b
0.833252947	0.000161478	1.21E-08	7	AI607873
1.070370148	0.000173558	1.30E-08	7	Marcksl1
1.509583574	0.000178493	1.34E-08	7	Ltc4s
1.233417545	0.000258059	1.93E-08	7	Fcgrt
0.700633828	0.000473225	3.54E-08	7	Ifitm3
-0.919236513	0.000583347	4.37E-08	7	Lgals3
-2.104255065	0.000681703	5.11E-08	7	Lhfpl2
0.452860952	0.00118203	8.85E-08	7	C1qc
-1.834759026	0.001257566	9.42E-08	7	Anxa1
-1.033633075	0.001414949	1.06E-07	7	Cd52
0.582677359	0.001468991	1.10E-07	7	Plek
0.547440191	0.001934072	1.45E-07	7	Serinc3
0.634418917	0.002200385	1.65E-07	7	Jund
1.144415885	0.003904412	2.92E-07	7	Cxcl10
0.667503508	0.003957268	2.96E-07	7	Dab2
0.590912802	0.004318314	3.23E-07	7	Ifitm2
0.565239869	0.005077689	3.80E-07	7	Ly6e
-1.322557587	0.006230793	4.67E-07	7	Aprt
-2.118086411	0.007055533	5.28E-07	7	Gpnmb
-1.091500611	0.00925923	6.93E-07	7	Anxa2
1.704034429	0.009468054	7.09E-07	7	Ccl24
0.752684125	0.010330954	7.74E-07	7	Ccl8
-1.914302411	0.011790875	8.83E-07	7	Adam8
0.743647834	0.013511146	1.01E-06	7	Ifi27l2a
1.441576329	0.014559317	1.09E-06	7	Timd4
1.025385452	0.016399719	1.23E-06	7	Cfh
-0.763897155	0.017552626	1.31E-06	7	Atox1
1.695472588	0.021579842	1.62E-06	7	Ifit3
-0.946473029	0.0220635	1.65E-06	7	S100a6
1.144415383	0.024268135	1.82E-06	7	Mtmr12
1.106165011	0.027023161	2.02E-06	7	Isg15
0.75035731	0.03259009	2.44E-06	7	Ctsc
-1.049183466	0.034102648	2.55E-06	7	Gm2a
0.828353131	0.035166723	2.63E-06	7	C5ar1
-1.099379019	0.037895816	2.84E-06	7	Cd72
-1.711023081	0.039065661	2.93E-06	7	Csf2rb2
-1.473406866	0.04209778	3.15E-06	7	Pfkp
1.313330315	0.042446461	3.18E-06	7	Pla2g2d
-0.89450811	0.04309925	3.23E-06	7	Arpp19
-1.708381398	0.044905833	3.36E-06	7	Mmp12

1.375998805	0.04980838	3.73E-06	7	Cp
-1.457682714	0.053959117	4.04E-06	7	Ncf4
-0.737471862	0.061809914	4.63E-06	7	Mpeg1
0.522509294	0.067576062	5.06E-06	7	Klf6
-1.221820874	0.070162639	5.25E-06	7	Clec7a
1.050214819	0.072602855	5.44E-06	7	Abca9
-0.722072338	0.07514335	5.63E-06	7	Ddost
-1.442369892	0.07839703	5.87E-06	7	Polr2e
0.355720714	0.080296057	6.01E-06	7	C1qb
-1.352829062	0.082012318	6.14E-06	7	Ezr
0.936202914	0.089768008	6.72E-06	7	Trib1
1.116571955	0.096056885	7.19E-06	7	Igfbp4
-1.055965567	0.097176072	7.28E-06	7	Fam107b
-1.528045	0.097921688	7.33E-06	7	Fn1
-1.977171328	0.099920797	7.48E-06	7	Il7r
-1.274168835	0.111162374	8.33E-06	7	Cd9
0.851943309	0.11920661	8.93E-06	7	Pmp22
0.582217451	0.129431716	9.69E-06	7	Cltc
-2.118768628	0.131190366	9.83E-06	7	Hilpda
0.713014983	0.137344181	1.03E-05	7	Cd81
1.425044364	0.152072075	1.14E-05	7	Npl
-0.75093935	0.178380854	1.34E-05	7	Alcam
-0.879824694	0.189013128	1.42E-05	7	AB124611
0.594626691	0.191616348	1.44E-05	7	Stab1
-1.8106221	0.196455332	1.47E-05	7	Fam20c
0.97509775	0.201162941	1.51E-05	7	Mnda
-1.31397132	0.209245504	1.57E-05	7	Nhp2
0.43224953	0.210304939	1.58E-05	7	Atp2b1
-1.291785525	0.221346793	1.66E-05	7	Api5
0.47806712	0.227132712	1.70E-05	7	Cdk2
-1.325986926	0.25132503	1.88E-05	7	Yy1
0.339767736	0.258497535	1.94E-05	7	Ubc
0.852060789	0.277081351	2.08E-05	7	Ccl9
1.031201972	0.285484852	2.14E-05	7	Usp18
-0.81408228	0.30237111	2.26E-05	7	Lsp1
0.989773824	0.306932609	2.30E-05	7	Birc3
0.888902254	0.310314992	2.32E-05	7	Plau
1.277002609	0.334993642	2.51E-05	7	Colec12
**0.414658624**	**0.344796762**	**2.58E-05**	**7**	**Csf1r**
0.592200592	0.349874651	2.62E-05	7	Aoah
-1.235271902	0.359656155	2.69E-05	7	Jak2
-1.795281936	0.375533226	2.81E-05	7	Itgax
-1.300882497	0.39868163	2.99E-05	7	Sel1l
-1.409393893	0.416501214	3.12E-05	7	Mrpl30
-0.75560279	0.422464907	3.16E-05	7	Sdhb
-1.069187608	0.431559788	3.23E-05	7	Abcg1
0.945486208	0.440997478	3.30E-05	7	Gas7
1.162052024	0.4415454	3.31E-05	7	Errfi1
0.971270663	0.458223396	3.43E-05	7	Cd209f
0.965381146	0.465538354	3.49E-05	7	Hmox1
-1.387793113	0.465679517	3.49E-05	7	Malt1
-1.593107359	0.48826895	3.66E-05	7	Tspan13
-1.862702523	0.495987315	3.71E-05	7	Tes
0.384967823	0.49631505	3.72E-05	7	Mcl1
-1.699018664	0.503964271	3.77E-05	7	Cytip
-0.854828381	0.517141259	3.87E-05	7	Myeov2
-0.986335592	0.527650052	3.95E-05	7	Vdac2
-1.72981113	0.529555971	3.97E-05	7	Tgm2
-0.90322523	0.537723537	4.03E-05	7	Mmp14
-2.212023966	0.542667405	4.06E-05	7	Napsa
0.60664591	0.555350631	4.16E-05	7	Aif1

0.802629475	0.561203506	4.20E-05	7	Rnase4
-1.182230044	0.568153476	4.26E-05	7	Zfp207
0.490315249	0.568583701	4.26E-05	7	Cd14
-1.412522353	0.616782858	4.62E-05	7	Bhlhe40
0.795568281	0.619746657	4.64E-05	7	Runx1
-0.923337891	0.626716398	4.69E-05	7	Ifi30
0.653974138	0.666216443	4.99E-05	7	Ednrb
-1.311145967	0.708794752	5.31E-05	7	Dpep2
-1.826786062	0.732371162	5.49E-05	7	Mrps34
1.524236589	0.758900255	5.68E-05	7	Rcan1
-0.735102451	0.760701971	5.70E-05	7	Aldoa
-0.435531793	0.794203123	5.95E-05	7	Rps3
-1.849239554	0.814256965	6.10E-05	7	Pdlim7
0.252886333	0.822446816	6.16E-05	7	Cst3
1.34376789	0.830769554	6.22E-05	7	Fxyd2
-0.998282622	0.853615644	6.39E-05	7	Csf2rb
-0.915549062	0.906347449	6.79E-05	7	Copb2
-1.175534802	0.920846964	6.90E-05	7	Commd8
-0.830420426	0.970308624	7.27E-05	7	S100a11
0.493271909	1	7.52E-05	7	Mt1
0.6378805	1	7.53E-05	7	Tnf
-1.153368235	1	7.56E-05	7	Spp1
-1.103633145	1	7.60E-05	7	Acp5
-0.417642168	1	7.83E-05	7	Pbxip1
-1.98142922	1	8.10E-05	7	Syngr1
-0.88075929	1	8.18E-05	7	Tpm4
-1.120105641	1	8.34E-05	7	Sema4d
-1.406421978	1	8.65E-05	7	Yipf3
-1.055896803	1	8.66E-05	7	Polr2g
-0.432325549	1	8.74E-05	7	Rpl32
1.780176328	1	8.96E-05	7	Elk1
-0.812299039	1	9.26E-05	7	Rpl23
-1.625343114	1	9.29E-05	7	Eepd1
-1.20882817	1	9.30E-05	7	Ssr2
-0.877345154	1	9.79E-05	7	Rgs1
-0.908139694	1	0.0001009	7	Pik3cb
0.526239654	1	0.0001037	7	Zfp36
-1.254643255	1	0.0001102	7	Slc37a2
0.640947964	1	0.0001111	7	Plekhg5
-0.993454876	1	0.0001112	7	Eef1d
-1.715651908	1	0.0001118	7	Tmem43
1.009351556	1	0.0001171	7	P2ry12
0.486354778	1	0.0001242	7	Tmem176b
-0.649338355	1	0.0001261	7	Psap
-0.999524757	1	0.0001347	7	Stk24
-1.101605798	1	0.0001369	7	Ptpre
1.202299576	1	0.0001389	7	Ifit2
-1.847661151	1	0.0001396	7	Emb
-1.049453934	1	0.0001404	7	Ssrp1
-0.959303318	1	0.0001433	7	Mien1
-0.685744706	1	0.0001451	7	Dad1
-1.521889763	1	0.0001454	7	Fgr
-0.922721815	1	0.0001464	7	Tmem160
0.320959058	1	0.0001498	7	Lyz2
0.630532623	1	0.0001568	7	Cd83
-0.448693461	1	0.0001592	7	Rilpl2
0.704874875	1	0.0001608	7	Kdm6b
0.662550775	1	0.000167	7	Ppp1r15a
-1.699610247	1	0.0001699	7	Gcc2
1.394158496	1	0.0001782	7	Tfpi
-0.943106262	1	0.0001813	7	Smpdl3a

-0.918126425	1	0.0001825	7	Myo1f
1.394635416	1	0.000184	7	Lpin1
-0.83077192	1	0.0001903	7	Rnh1
-0.742655424	1	0.0001907	7	Smdt1
-1.705030488	1	0.0001935	7	Hint2
0.860090728	1	0.000209	7	Gda
-0.83012289	1	0.0002191	7	Tomm70a
-0.563700449	1	0.0002227	7	Psmc4
-1.090513864	1	0.0002373	7	Tmed3
1.271218993	1	0.0002391	7	Chd1
1.899955717	1	0.0002426	7	Oasl1
-0.273132941	1	0.0002463	7	Psen2
-1.425089708	1	0.0002479	7	Fuca2
1.348402221	1	0.0002522	7	Calml4
-1.017898687	1	0.0002665	7	Ndufb7
0.497995963	1	0.0002851	7	Zfp36l1
-1.136562351	1	0.0002863	7	Ndrg1
-0.329393375	1	0.0002905	7	Caprin1
0.535241856	1	0.0002908	7	Nfkbiz
-0.33395121	1	0.0002933	7	Gm13826
0.483536961	1	0.0002965	7	Trf
-0.521314908	1	0.0003056	7	Fxyd5
-0.559042748	1	0.0003098	7	Scarb2
-0.899184763	1	0.0003179	7	Hspe1
-0.938237946	1	0.0003247	7	Glipr1
1.528364265	1	0.000329	7	Ifit1
-1.109198681	1	0.0003295	7	Rnf7
-0.554755304	1	0.0003319	7	Cox6a1
-1.087142102	1	0.0003335	7	Stat6
-0.704032866	1	0.0003433	7	Ccdc12
-0.700060562	1	0.0003467	7	Cd47
0.944426245	1	0.0003522	7	Samd9l
-1.144164597	1	0.0003591	7	Slc25a39
-1.589972189	1	0.0003639	7	Scd2
-1.651861014	1	0.0003657	7	Cops5
-0.712102482	1	0.0003683	7	Cct4
0.703560982	1	0.0003695	7	Ehd4
-1.000042758	1	0.0003699	7	Gpr137b-ps
-0.52397963	1	0.0003763	7	Cotl1
-0.559091289	1	0.0003785	7	Ndufa2
-0.609831318	1	0.0003827	7	Myl12a
0.318096845	1	0.0003879	7	NP-904329.1
-1.164567727	1	0.0004089	7	Pdhb
-0.411908603	1	0.0004159	7	Ppp1ca
-0.928527995	1	0.0004227	7	Psmc2
-0.675524792	1	0.0004305	7	Pum1
0.290587238	1	0.0004514	7	2900060B14Rik
-0.583602458	1	0.0004576	7	Psmd1
0.848648277	1	0.0004679	7	Slc9a9
-1.775023655	1	0.0004802	7	Slc7a11
0.649970725	1	0.0004826	7	Tmem127
-0.257146292	1	0.0004837	7	Pdia6
0.707328253	1	0.0004885	7	Wwp1
-0.710051161	1	0.00049	7	Psma6
-1.551273853	1	0.0005016	7	1700123O20Rik
-0.469303349	1	0.0005039	7	Gns
-1.598411868	1	0.0005042	7	Galnt6
0.748012995	1	0.00052	7	Zfhx3
-1.094181357	1	0.0005359	7	Wsb2
-0.89944764	1	0.0005376	7	Bin2
-0.887615363	1	0.0005397	7	Pld3

-0.575436662	1	0.0005398	7	Ggh
-1.881484078	1	0.000541	7	Mrpl34
1.190306364	1	0.0005453	7	Ifi203
-0.605906298	1	0.000551	7	Chp1
0.384179071	1	0.0005705	7	C1qa
-0.849026542	1	0.0005745	7	Vps28
1.383218191	1	0.0005786	7	Etv1
-0.521230615	1	0.0005856	7	H13
-1.546177539	1	0.0005884	7	Frrs1
-1.853278418	1	0.0005888	7	Itgal
-1.746340514	1	0.0005899	7	Cadm1
-0.485351944	1	0.0005968	7	Snx1
0.312448722	1	0.0006196	7	Slc46a3
-1.13622289	1	0.0006216	7	Cpd
-1.137569663	1	0.0006255	7	Hsd17b10
-0.699797717	1	0.0006386	7	Snrpg
-0.986771325	1	0.0006522	7	Dusp2
-0.834806857	1	0.0006525	7	Il2rg
-0.783674809	1	0.0006717	7	Ptpn6
-0.686458987	1	0.000685	7	Ndufc1
-0.668378324	1	0.0007155	7	Ndufa6
-0.781344606	1	0.0007359	7	Lipa
-1.223108924	1	0.0007619	7	Otulin
-1.030596118	1	0.0007642	7	Ube2a
-0.729678115	1	0.0007699	7	Rpl3
-0.826231084	1	0.0007722	7	Gsn
0.77347241	1	0.0007812	7	Maf
0.574379703	1	0.000786	7	Csrnp1
-0.60264618	1	0.0007893	7	Acsl4
1.095167475	1	0.0008005	7	Hip1
-0.91316952	1	0.0008312	7	Stk4
-0.701560161	1	0.0008341	7	Mgea5
-1.243738432	1	0.0008393	7	Fam173a
-1.207182627	1	0.0008413	7	Pgk1
0.369470657	1	0.0008436	7	Fcgr3
-0.663691113	1	0.0008482	7	Mical1
0.506889308	1	0.0008689	7	Kctd12
-0.680687825	1	0.000882	7	Psmc1
-0.937469574	1	0.0008823	7	Psmd12
-1.406222759	1	0.0008856	7	Gsdmd
-0.519502798	1	0.000886	7	Itgb2
1.440503038	1	0.0008906	7	Optn
-0.53852644	1	0.000907	7	Gnas
0.676311304	1	0.000938	7	Zfp703
-0.942305384	1	0.00094	7	Sep.11
-1.654977953	1	0.0009457	7	Kcnn4
-1.562679408	1	0.0009464	7	Msl1
-1.064817421	1	0.0009515	7	Ndufb6
-0.555076704	1	0.0009661	7	Creg1
-0.612892452	1	0.0009703	7	Trappc4
-0.844669782	1	0.000979	7	Rsrp1
-0.473451887	1	0.0009813	7	M6pr
-0.825312349	1	0.0009839	7	Mrpl4
1.421275244	1	0.0009888	7	Spic
0.862784948	1	0.0009894	7	Ap2a2
-1.596036758	1	0.001003	7	Gpr35
-1.637188439	1	0.0010089	7	Arl5c
1.453409594	1	0.001027	7	Pld1
-1.030206392	1	0.0010302	7	Tpm1
-0.763112082	1	0.0010489	7	Hpcal1
-1.514235835	1	0.0010506	7	Rhoq

-0.76717036	1	0.001063	7	Nemf
-0.752306631	1	0.0010895	7	Sla
-0.729863196	1	0.0010927	7	Stx3
-1.599181929	1	0.0011013	7	AA467197
1.27099505	1	0.0011175	7	Mylk
0.450038212	1	0.0011286	7	Cebpb
-0.351219312	1	0.0011354	7	Rtfdc1
-1.409364499	1	0.0011482	7	Usp4
-0.819748621	1	0.0011598	7	Tmbim4
-1.511083083	1	0.0011781	7	Zranb2
0.533043019	1	0.0011782	7	Slc15a2
-1.427016197	1	0.001198	7	Odc1
-0.571613845	1	0.0012072	7	Pomp
-0.374294679	1	0.0012156	7	Bcas2
0.595421539	1	0.0012165	7	Cflar
-1.012007366	1	0.0012331	7	Trappc6a
-1.103295961	1	0.0012334	7	Pdpn
-0.527293931	1	0.0012377	7	Actn4
1.038791552	1	0.0012486	7	Ophn1
0.521469454	1	0.0012493	7	Tgfbr2
-1.546918292	1	0.0012557	7	Ptprs
-1.531540511	1	0.001258	7	Cdkn2aipnl
-1.216346731	1	0.0012594	7	Cnot7
-0.764335838	1	0.00126	7	Eif3g
0.486817915	1	0.0012605	7	Efhd2
-0.499641414	1	0.0012621	7	Snrpe
-1.18357194	1	0.0012649	7	Apex1
-1.340182646	1	0.0012725	7	Apbb2
-0.835692626	1	0.0012869	7	1810026B05Rik
-0.636642914	1	0.0012941	7	Cox5a
-0.601727009	1	0.0013043	7	Ppib
-1.626586907	1	0.0013077	7	Stx8
-1.475555094	1	0.0013127	7	Arrdc1
-0.268043291	1	0.0013132	7	Cyfip2
1.0491996	1	0.0013206	7	Itsn1
-0.895490682	1	0.0013278	7	2410015M20Rik
-0.93758922	1	0.00137	7	Srsf11
1.130847611	1	0.0013741	7	Rnasel
-0.426693616	1	0.0013888	7	Paip2
-0.930072673	1	0.0013994	7	Ngdn
0.966383077	1	0.0014231	7	Slco2b1
0.615999471	1	0.0014354	7	Hivep2
-2.001790059	1	0.0014366	7	Nkg7
-0.672278042	1	0.0014634	7	Bcl2l11
0.893967456	1	0.0014789	7	Ppp1r10
-0.60446795	1	0.0014914	7	Timm13
-1.005924599	1	0.0015004	7	Clptm1l
-0.638150905	1	0.001514	7	Tmsb10
0.793938064	1	0.0015221	7	Ubtf
-1.575657713	1	0.0015297	7	Zak
-1.301833788	1	0.001531	7	Glrx3
-1.08095256	1	0.0015687	7	Apoa1bp
0.574643184	1	0.0015713	7	Hpgd
0.781678032	1	0.0015885	7	Gmfg
-0.529534114	1	0.0016055	7	Psmb1
-1.232208151	1	0.0016056	7	Plxnc1
0.470296264	1	0.0016226	7	Mob3c
0.999527097	1	0.0016293	7	Ddx60
-0.607792254	1	0.0016401	7	Ech1
-1.0331763	1	0.0016457	7	Csnk2b
-0.918780945	1	0.0016493	7	R3hdm1

1.218239524	1	0.0016777	7	Tmod1
0.950539084	1	0.00169	7	Grap
-0.523242231	1	0.0016911	7	Minos1
-0.606648751	1	0.0017357	7	Dnm2
-0.502618023	1	0.0017463	7	Itga6
-1.425323384	1	0.0017671	7	Gcsh
-0.530195172	1	0.001785	7	Sirpa
-1.292233567	1	0.0017888	7	Smc4
-1.573398154	1	0.0018012	7	Polr2b
0.679524668	1	0.0018079	7	Vwa5a
1.252143664	1	0.0018149	7	Hmga2-ps1
-0.577021723	1	0.0018172	7	Pcbp1
-0.806752661	1	0.0018352	7	Zfand2a
-0.5038257	1	0.0018472	7	S100a13
-0.75109963	1	0.0018475	7	Atp8b4
0.304396186	1	0.0018498	7	Mir6236
-1.059223512	1	0.0018663	7	Otud4
-0.865252898	1	0.0018665	7	Dtx4
-1.087711668	1	0.0018719	7	Smim11
-0.714522762	1	0.0018792	7	S100a4
-0.437862429	1	0.0018946	7	Tmem147
0.539445473	1	0.0018948	7	Insig1
-0.741056647	1	0.0018981	7	Anxa7
-0.526422016	1	0.0019201	7	Gusb
0.883031242	1	0.001939	7	Fcna
-1.483215237	1	0.0019454	7	Rnf128
-0.450354879	1	0.0019906	7	Uqcrfs1
-1.206561458	1	0.0020022	7	Fabp5
0.78667031	1	0.0020134	7	Tmem88
-1.532984421	1	0.0020171	7	Mmp19
-0.723701241	1	0.0020288	7	Mrpl20
-0.933672766	1	0.0020316	7	Dap3
-1.044748596	1	0.0020526	7	Vdac1
-0.287690589	1	0.002068	7	Gab2
-1.029154605	1	0.0020837	7	Pstpip1
-0.617066541	1	0.0021011	7	BC031181
-0.495849811	1	0.0021217	7	Pttg1ip
-1.315242767	1	0.0021317	7	Cd300lf
-0.482917925	1	0.0021361	7	Atp5e
1.189581843	1	0.0021386	7	Rsad2
0.535814048	1	0.0021399	7	Eif4a1
0.507185443	1	0.0021423	7	Sfpq
-0.962789337	1	0.0021491	7	Prdx6
-1.241071071	1	0.0021526	7	Atp2b4
-0.701856465	1	0.0021696	7	Coro1a
-0.890425101	1	0.0021917	7	Msrb1
-0.620189313	1	0.0021929	7	Anp32e
0.798381759	1	0.0022067	7	Nme4
0.788153915	1	0.0022166	7	Sdc4
1.105868172	1	0.0022369	7	Hrh1
-0.403413382	1	0.0022956	7	Ptk2b
-0.575079777	1	0.0022958	7	Capg
-1.222357379	1	0.0023011	7	BC004004
-1.125372443	1	0.0023047	7	Tmem134
-0.603490441	1	0.0023292	7	Psmb6
-0.66591051	1	0.0023381	7	Axl
-0.49309916	1	0.0023585	7	Capns1
0.935171454	1	0.0023629	7	Cxcl3
-0.629061631	1	0.0023716	7	Cnih1
-0.691252182	1	0.0023719	7	Smim14
0.385306607	1	0.0023856	7	Nr4a1

-1.296019353	1	0.0024057	7	Pqlc3
-1.174787845	1	0.0024191	7	Sdhd
-0.601113454	1	0.0024341	7	Psmc6
-0.936967257	1	0.0024508	7	Ak2
-0.721020858	1	0.0024945	7	Rpp21
-1.271935372	1	0.002508	7	Agpat4
-1.10885709	1	0.0025124	7	Cox14
-1.309410987	1	0.0025145	7	Pin1
0.762703425	1	0.0025465	7	Ifih1
-0.29071146	1	0.0025478	7	Cd300a
-1.403030549	1	0.0025561	7	Gnptg
0.64948944	1	0.002568	7	Rtp4
-1.648370038	1	0.0025891	7	Ict1
-0.975118805	1	0.0025951	7	Gtf3c2
-0.618421834	1	0.0026271	7	Selplg
-0.375571401	1	0.0026294	7	Tgfbr1
0.454032428	1	0.0026372	7	Snx3
-0.357280738	1	0.0026759	7	Ccar1
-0.890100007	1	0.0026788	7	Nceh1
0.378503645	1	0.0026898	7	Cmah
-0.767998442	1	0.0027204	7	Rnpep
-1.160955525	1	0.0027245	7	Timm10b
-0.487049162	1	0.0027358	7	Rbm5
-0.403618441	1	0.0027402	7	Mrpl52
-0.467431251	1	0.0027412	7	Tmem123
-0.267126725	1	0.0027446	7	Eef1a1
-0.488459606	1	0.0027496	7	Cxcl16
1.141634287	1	0.0027526	7	Ppard
-1.459650439	1	0.0027777	7	Galns
0.676547637	1	0.0027795	7	Snx2
-0.390280803	1	0.0027844	7	Sri
0.868707756	1	0.0027894	7	Rapgef2
-0.844899008	1	0.0027969	7	2700029M09Rik
-0.68571631	1	0.002801	7	Tnfaip2
0.670499343	1	0.002802	7	Tiparp
-0.542571548	1	0.0028076	7	Tln1
-0.75877823	1	0.0028456	7	Snapc5
-0.426781999	1	0.0028491	7	Psmc3
-1.021342739	1	0.0028608	7	Ubp1
-0.255149007	1	0.0028653	7	Cops6
-0.911099286	1	0.0029108	7	Sdf2l1
-0.60894336	1	0.0029974	7	Scamp3
-0.439099056	1	0.0030361	7	Rer1
-0.546615208	1	0.0030534	7	Atp6v1b2
0.437377104	1	0.0030665	7	Kmt2e
-0.517327884	1	0.0030727	7	Nmt1
-1.149729839	1	0.0030903	7	Wdr74
-1.169652685	1	0.0031099	7	Lpcat3
-1.172700671	1	0.0031175	7	Rab4b
-0.976047895	1	0.0031228	7	Dnajc2
-1.670257959	1	0.0031262	7	Nes
1.093130891	1	0.0031281	7	Gpr34
0.440978825	1	0.0031329	7	Fgfr1
0.677791602	1	0.0031449	7	Clec10a
-0.689180175	1	0.0031525	7	Lmo4
-1.217066905	1	0.0031754	7	Map4k3
-1.521527224	1	0.0031777	7	Sec13
0.323025314	1	0.0031875	7	Lgmn
0.44734956	1	0.003202	7	Gp49a
-1.489162977	1	0.0032216	7	Ddx39
-1.292632498	1	0.0032477	7	Crnkl1

-1.356796163	1	0.0032479	7	Metap1d
-0.444289115	1	0.0032497	7	Ctsh
-0.362874566	1	0.0032916	7	Sf3b6
-1.662297158	1	0.0033125	7	Sorl1
1.018960929	1	0.0033573	7	Alox5
-0.559800699	1	0.003367	7	Gm3258
-0.754914609	1	0.0033706	7	Tns3
-0.488788463	1	0.0034129	7	Rab2a
-0.896484221	1	0.0034323	7	Lpgat1
-1.040101497	1	0.0034553	7	Leprotl1
0.936578914	1	0.0034618	7	Casp4
0.272247005	1	0.0034838	7	Clta
-0.646728292	1	0.0034929	7	Snd1
-0.934883537	1	0.003502	7	Uqcrc2
-0.986248128	1	0.0035058	7	Akip1
-0.594405012	1	0.0035307	7	Fubp1
-0.437480712	1	0.0035323	7	Tmem66
-1.464007996	1	0.0036294	7	Chchd3
0.902308212	1	0.0036619	7	Mndal
-0.284959682	1	0.0036875	7	Eif3f
-1.145245911	1	0.0036914	7	Amdhd2
-1.363801446	1	0.00371	7	Ddx47
0.737828449	1	0.003716	7	Gm6377
-1.105921006	1	0.0037198	7	Tlk1
-1.463403077	1	0.0037208	7	Vps4a
-1.336450557	1	0.0037474	7	Mllt4
-0.827553595	1	0.0037624	7	Tbc1d10b
-0.854937248	1	0.0037865	7	Paf1
-0.527942951	1	0.0038041	7	Eif4a3
0.831773759	1	0.0038269	7	Kansl1l
-0.801889795	1	0.0038523	7	Use1
-0.703934162	1	0.0038781	7	Dtnbp1
-0.643867571	1	0.0039123	7	Eif3l
0.437219818	1	0.003962	7	Ccl6
-0.797244763	1	0.0040196	7	Psmd3
-1.52835655	1	0.0040687	7	Cyp4f18
0.784171453	1	0.00416	7	Tank
0.771213687	1	0.0041725	7	Tlr8
0.473402683	1	0.0041791	7	Pag1
-0.274194219	1	0.0041848	7	Ifi27
-0.727360339	1	0.0042429	7	Stt3b
-0.415866258	1	0.0042684	7	Ostf1
-0.888134225	1	0.0042935	7	Ykt6
-1.569733944	1	0.0043138	7	Havcr2
-0.628302891	1	0.0043143	7	Cct3
-1.62143597	1	0.0043897	7	Cyb5r1
-0.36513933	1	0.0044057	7	Actr1a
-0.470094748	1	0.0044474	7	Bola2
0.994481692	1	0.0044718	7	Ifi204
-0.704686163	1	0.0044755	7	Clec4e
-0.74758285	1	0.004479	7	Pdcd6
-1.424356522	1	0.0044952	7	Comtd1
-0.52739851	1	0.0045102	7	Cd44
-0.676281892	1	0.0045803	7	Irf8
-0.993479238	1	0.0045829	7	Slc16a3
-0.932960994	1	0.0046044	7	Golgb1
-0.532507043	1	0.0046066	7	Hvcn1
-0.598340568	1	0.0046415	7	Mtpn
-1.151155611	1	0.0046533	7	Surf2
-0.65387118	1	0.0046713	7	Hsd17b12
-0.817327545	1	0.0047075	7	Brd8

-1.467542932	1	0.0047691	7	Abi3
-0.391636203	1	0.0047708	7	Metap2
-0.264970983	1	0.0047783	7	Map4k4
-0.480300897	1	0.0048148	7	Basp1
-1.336490334	1	0.0048161	7	Sipa1
-1.23353872	1	0.0048186	7	Thbs1
-0.827659079	1	0.0048578	7	Pqbp1
0.537732822	1	0.0049029	7	Faf1
-0.548087565	1	0.0049505	7	Tkt
-0.287920226	1	0.0049854	7	Lair1
-1.124148519	1	0.0050002	7	Larp1
0.960122508	1	0.0050065	7	Tns1
-0.789925686	1	0.0050498	7	St8sia4
-0.685365652	1	0.0050595	7	Hsp90aa1
-0.35222439	1	0.0050784	7	Cyba
-0.802391981	1	0.0051	7	Ddx42
0.60245166	1	0.0051798	7	Jmjd1c
1.124359806	1	0.0052051	7	Arid5a
-0.536133355	1	0.0052067	7	Rac2
0.307276476	1	0.0052651	7	Dazap2
-0.47311349	1	0.0052673	7	Sec61a1
-0.977152831	1	0.0053472	7	Lman2
0.860129684	1	0.0053764	7	Slfn5
-0.444481996	1	0.0054294	7	Rchy1
-1.199076126	1	0.0054353	7	Rgcc
-0.269947649	1	0.0054852	7	Rps14
-0.41733213	1	0.0054857	7	Akr1a1
0.971501186	1	0.0055358	7	Ppm1k
-0.654323044	1	0.0055627	7	Gpr132
-0.490853913	1	0.0055823	7	Arf6
-1.258028784	1	0.0056075	7	Tle1
-1.715788754	1	0.0056156	7	F10
-0.696532467	1	0.0056201	7	Mmd
-0.279435308	1	0.0056286	7	Hnrnpd
-0.551069464	1	0.0056512	7	Sgk1
0.515211152	1	0.0056551	7	Zfand5
-0.291200936	1	0.0056577	7	Tmem242
-0.892835713	1	0.0057057	7	Poldip3
-0.446219452	1	0.0057263	7	Emilin2
-0.802631222	1	0.0057421	7	Ubald1
-0.458973377	1	0.0057514	7	Ndufa3
-1.153207042	1	0.0058305	7	Plin3
-0.686962193	1	0.005879	7	Pdcd6ip
-0.661862458	1	0.0059027	7	Ssr1
-0.355159233	1	0.0059146	7	Ifngr2
0.691704648	1	0.0059226	7	Rbm47
-0.554497189	1	0.0059743	7	Eif3i
-1.244373785	1	0.0060049	7	Ccr2
-0.556286	1	0.0060144	7	Cox7a2
-0.725565027	1	0.006049	7	Plbd1
-1.485793181	1	0.006083	7	Lsm3
0.704701434	1	0.0061063	7	Fcgr2b
-1.15841015	1	0.0061318	7	Ltbp3
-1.438999574	1	0.006137	7	Ercc1
-0.594676286	1	0.0061941	7	Tbxas1
-0.475459221	1	0.0062151	7	Psma1
-0.374452711	1	0.0062587	7	Rit1
0.35941542	1	0.0064058	7	Ahnak
-0.328743145	1	0.0064084	7	Osbpl8
-1.034960834	1	0.0064264	7	Glt25d1
-0.866373612	1	0.0064776	7	Lat2

-0.417846601	1	0.0064802	7	Senp2
-0.833833687	1	0.0064922	7	Snx10
-0.845506924	1	0.0064957	7	Gm11974
-1.311267371	1	0.0064972	7	Cisd2
-0.500606344	1	0.0065137	7	Flna
-0.57091215	1	0.0065187	7	Trem2
-1.243122127	1	0.0065289	7	Cyb561d2
-0.800026509	1	0.0066047	7	Dctn4
-0.60205535	1	0.0066083	7	Eif3b
-0.527604514	1	0.006623	7	Psmd8
-1.397840014	1	0.0067291	7	Esf1
-1.105682086	1	0.0068025	7	Camta2
-0.872116876	1	0.0068488	7	Smim3
1.329830468	1	0.006887	7	Vsig4
-0.740467208	1	0.0069634	7	Snhg1
-0.844708597	1	0.0069705	7	Lims1
-0.902762747	1	0.0069789	7	Mgst1
0.825600406	1	0.0070178	7	Sorbs3
-0.453166591	1	0.0070192	7	Ndufb3
-1.193481065	1	0.0070349	7	Aebp2
-1.202718708	1	0.0070406	7	Mrps26
-0.8950466	1	0.007077	7	Dennd5a
-0.820793213	1	0.0070855	7	Mrpl17
-1.217640144	1	0.0070999	7	Plekha1
0.297198969	1	0.0071299	7	Wfdc17
0.664142307	1	0.0071376	7	Tspan31
0.777719705	1	0.0071936	7	Cd38
-1.073701582	1	0.0072091	7	Plrg1
-0.50056964	1	0.0072157	7	Xbp1
-0.916033548	1	0.007269	7	Gfpt1
-0.786487055	1	0.0072893	7	Myo1e
-1.299187043	1	0.0073068	7	Cep83
-0.2893173	1	0.0073296	7	Eprs
-0.91131186	1	0.0073394	7	Rab11fip1
-0.449975	1	0.007358	7	Igsf8
-0.997988363	1	0.0073585	7	E430025E21Rik
-0.764124297	1	0.0074521	7	Lbr
-0.481910813	1	0.0075035	7	Slc29a3
-0.354691789	1	0.0075135	7	Hp1bp3
-1.025354092	1	0.0076013	7	Uggt1
-1.151680623	1	0.0076875	7	Zbed6
-1.377010337	1	0.0077136	7	Scd1
-0.358235949	1	0.0077139	7	Mar.02
-1.106475422	1	0.007754	7	Gnl1
-0.885055525	1	0.0077615	7	Tmem248
-1.365193281	1	0.0077703	7	Cdk4
-0.431453634	1	0.0077739	7	Psme2b
-0.426659351	1	0.007793	7	Rpl14-ps1
-1.815966527	1	0.0077994	7	Spn
-0.982047791	1	0.0078222	7	Srp19
-1.099034999	1	0.0078403	7	Mak16
-0.612055094	1	0.0078591	7	Esd
-1.03542587	1	0.0078713	7	Elf4
-0.512110723	1	0.0078969	7	Derl1
0.401626332	1	0.0079049	7	Rnd3
-0.272811221	1	0.0079136	7	Pik3cd
-0.76080399	1	0.0079399	7	Vmp1
0.557115481	1	0.0079475	7	Fnip1
-1.122996298	1	0.0079821	7	Gtf2h2
-1.265041908	1	0.0080032	7	Apobr
-0.422003505	1	0.0080299	7	Tmem14c

-1.033612289	1	0.0080314	7	Prkrir
0.695246302	1	0.0080591	7	Tcf4
-1.266263656	1	0.0080702	7	Plcb2
-0.379448742	1	0.0081412	7	Rab6a
-0.439824716	1	0.0081591	7	AF251705
-0.990059471	1	0.0081688	7	Arhgap25
-0.59070138	1	0.0081811	7	Bcl2l1
-1.14848062	1	0.0081815	7	Mrpl32
-0.44340787	1	0.008218	7	Strap
-0.565515729	1	0.0082411	7	Sin3b
-0.383709941	1	0.0082515	7	Rassf3
0.544762226	1	0.0082672	7	Sema4a
0.295248216	1	0.0082785	7	Marcks
-1.173009892	1	0.0083033	7	Wbp1l
-0.70466177	1	0.0083603	7	Fyn
-1.22815696	1	0.0084176	7	Smad4
1.068440067	1	0.0084192	7	Serpinb8
0.326269162	1	0.0085622	7	Clpp
1.28585914	1	0.008609	7	Cyr61
-0.458505441	1	0.0086374	7	Serp1
-1.088995167	1	0.008672	7	Cav2
-1.184314827	1	0.0087097	7	Lnpep
0.550523255	1	0.0087195	7	Mgl2
-0.428823098	1	0.0087216	7	Man2b1
-0.808368045	1	0.0087625	7	B4galnt1
-0.441799246	1	0.0088256	7	Ran
-0.393703849	1	0.0088372	7	Srp14
-0.885159265	1	0.0088472	7	Abhd5
0.823779657	1	0.0088745	7	Abl2
-0.904349734	1	0.0088869	7	Appl1
0.285027907	1	0.0088903	7	App
-0.801537084	1	0.0089615	7	Rps6kb1
-0.892121019	1	0.009018	7	Pilra
-1.001407111	1	0.0091469	7	Ank
-0.500558538	1	0.0091739	7	H2afy
-1.088134977	1	0.0091953	7	Cd276
-0.498223687	1	0.0092392	7	Ankrd13a
-0.348351915	1	0.0092705	7	AW112010
-0.71124838	1	0.0093056	7	Ndufs7
-1.192527224	1	0.0093151	7	Cpq
-0.520852724	1	0.0093426	7	Psma7
-0.636226482	1	0.0093565	7	Srsf3
-0.341199833	1	0.0094239	7	Coro1b
-0.39777749	1	0.0094417	7	Sdc3
-0.693325769	1	0.0094513	7	Sepn1
-0.568019995	1	0.0094537	7	Emc1
-1.365724449	1	0.0095327	7	Igf2r
-0.694725223	1	0.0095745	7	Tmem126a
-1.303651188	1	0.0096179	7	Isyna1
-0.800925691	1	0.009661	7	Ptpmt1
-0.418845538	1	0.0096811	7	Mrpl12
0.276680143	1	0.0097388	7	Lilrb4
-1.222150039	1	0.0097727	7	Ercc6
1.03713577	1	0.0098434	7	Tlcd2
-0.275995509	1	0.0098857	7	Ptprc
-0.598110834	1	0.0098865	7	Pgls
-0.352950547	1	0.0099021	7	Chmp3
-0.420043012	1	0.0099046	7	Soat1
1.994565199	9.55E-125	7.15E-129	8	Leprel4
2.210960677	4.61E-123	3.45E-127	8	Ramp2
2.422862263	4.37E-115	3.28E-119	8	Fbln2

3.411462899	1.33E-106	9.97E-111	8	Ltbp4
2.017218999	5.84E-106	4.37E-110	8	Lhfp
2.137409181	1.87E-100	1.40E-104	8	AU021092
1.685912598	2.88E-100	2.16E-104	8	Tie1
1.798220907	4.57E-97	3.42E-101	8	Tshz2
2.730907071	2.90E-93	2.17E-97	8	Adh7
2.132599167	3.61E-92	2.70E-96	8	Selp
2.141666889	4.54E-91	3.40E-95	8	Sulf1
1.531508348	9.21E-91	6.90E-95	8	Parm1
2.507646008	1.35E-89	1.01E-93	8	Lims2
3.199058181	2.48E-89	1.86E-93	8	Bmp4
2.150703442	1.50E-87	1.12E-91	8	Gja4
1.511514227	2.73E-86	2.04E-90	8	Col4a3
1.494156562	3.58E-82	2.68E-86	8	Parva
1.275655914	5.21E-82	3.90E-86	8	Twist1
2.941986373	1.99E-80	1.49E-84	8	Adh1
1.581167939	2.54E-76	1.90E-80	8	Gpr116
1.862189063	2.75E-76	2.06E-80	8	Krt18
1.639045512	3.06E-76	2.29E-80	8	Phldb2
2.982250697	4.31E-74	3.23E-78	8	Ptprb
2.250589798	1.30E-73	9.71E-78	8	Ptgis
2.184613004	5.55E-73	4.16E-77	8	Fmo1
1.873428466	7.39E-73	5.54E-77	8	Tinagl1
1.513894218	1.97E-72	1.48E-76	8	Tspan7
1.786200394	9.13E-72	6.84E-76	8	Ecscr
2.143568019	1.20E-71	8.99E-76	8	Tek
2.177080011	2.24E-71	1.68E-75	8	Smoc1
1.785962352	6.61E-70	4.95E-74	8	Gkn3
1.572600412	7.43E-70	5.57E-74	8	Cdh13
1.389988072	1.79E-69	1.34E-73	8	Tspan6
3.267922669	1.62E-68	1.21E-72	8	Clu
1.894449756	1.62E-68	1.22E-72	8	Msx1
2.071719496	2.28E-68	1.71E-72	8	Boc
1.628034558	2.74E-68	2.05E-72	8	Car8
1.599100174	2.28E-67	1.71E-71	8	Aldh3a1
1.057969201	2.58E-67	1.93E-71	8	Pvrl2
2.477119965	3.34E-67	2.50E-71	8	Ctgf
3.191310265	4.24E-66	3.18E-70	8	Rbp1
1.385325838	1.79E-65	1.34E-69	8	Ndn
1.122035219	1.79E-65	1.34E-69	8	Npr2
1.848098888	2.30E-65	1.72E-69	8	Trabd2b
1.26253545	3.07E-65	2.30E-69	8	Bcar1
1.021198315	3.28E-65	2.46E-69	8	Exoc3l
0.938269639	9.08E-65	6.80E-69	8	Pcdh9
1.440361582	9.09E-65	6.81E-69	8	Nanos2
2.551271327	1.13E-64	8.50E-69	8	Emcn
1.867523553	1.18E-64	8.86E-69	8	Ephx2
3.909848884	2.33E-64	1.74E-68	8	Cytl1
2.064471313	2.54E-64	1.90E-68	8	Gpr4
1.730812507	5.76E-64	4.31E-68	8	Lmcd1
2.866997986	2.18E-62	1.64E-66	8	Sfrp1
1.581167939	8.02E-62	6.00E-66	8	Pkhd1l1
2.05666445	1.19E-61	8.88E-66	8	Egfl7
2.073273278	8.52E-61	6.38E-65	8	Wnk4
1.115540207	1.47E-60	1.10E-64	8	Sardh
1.019715139	3.92E-59	2.94E-63	8	Pla2g5
2.631600256	6.14E-58	4.60E-62	8	Mmrn2
1.886684233	6.29E-58	4.71E-62	8	Fermt2
0.993654052	4.37E-57	3.28E-61	8	Wipf3
1.667216389	4.56E-57	3.41E-61	8	Clec14a
0.982104357	6.96E-57	5.21E-61	8	Gpam

1.162644376	7.56E-57	5.67E-61	8	2900041M22Rik
2.264115754	1.30E-56	9.73E-61	8	Pcolce2
1.134979933	2.50E-56	1.87E-60	8	Tmod2
1.856196883	3.52E-54	2.63E-58	8	1500015O10Rik
1.005325468	7.56E-54	5.66E-58	8	Ugt8a
1.429759073	1.18E-53	8.86E-58	8	Nnmt
0.983248365	2.07E-53	1.55E-57	8	1700030C10Rik
1.757972127	3.30E-53	2.47E-57	8	Jam2
1.506234544	4.60E-53	3.44E-57	8	Mtap7d3
1.485857759	5.07E-53	3.80E-57	8	Smtnl2
1.271305924	5.26E-53	3.94E-57	8	Ddah1
1.273745694	1.02E-52	7.62E-57	8	Irf6
1.576752125	1.16E-52	8.65E-57	8	Cnn3
0.992686466	5.80E-51	4.34E-55	8	Cdc42ep5
2.232890406	7.37E-51	5.52E-55	8	Aldh1a1
0.918837577	7.82E-51	5.85E-55	8	Myzap
0.937301116	9.20E-49	6.89E-53	8	Cyp4b1
0.862077694	9.20E-49	6.89E-53	8	Gdf6
3.623339186	1.56E-48	1.17E-52	8	Tm4sf1
2.347851572	3.06E-48	2.29E-52	8	Frzb
1.499437017	1.47E-47	1.10E-51	8	Snora68
1.265608566	6.12E-46	4.58E-50	8	Rps6kl1
1.787920693	1.08E-45	8.10E-50	8	BC100451
1.64343971	5.56E-45	4.17E-49	8	Lox
1.375837596	1.04E-44	7.78E-49	8	Il9r
1.353611714	1.31E-44	9.83E-49	8	Gnmt
1.145339967	1.35E-44	1.01E-48	8	Ncam1
1.844076073	1.38E-44	1.03E-48	8	Eln
1.624025172	1.44E-44	1.08E-48	8	Tlcd1
2.553811475	5.96E-44	4.46E-48	8	Ace
0.793270123	1.13E-42	8.45E-47	8	Tead2
1.516509001	4.66E-42	3.49E-46	8	Tmtc1
1.120987991	5.44E-42	4.08E-46	8	Notch4
1.106923061	8.89E-42	6.66E-46	8	Tcf7l1
1.319325782	9.09E-42	6.81E-46	8	Ston1
2.175502191	1.09E-41	8.13E-46	8	Nedd4
1.526715435	6.50E-41	4.87E-45	8	Adcy4
1.564728834	1.18E-40	8.81E-45	8	Prex2
1.394851317	1.55E-40	1.16E-44	8	Foxc1
2.476635304	3.22E-40	2.41E-44	8	Timp3
1.858813276	1.41E-38	1.06E-42	8	Efna5
1.932172128	1.82E-38	1.37E-42	8	Spon1
1.003394965	3.86E-38	2.89E-42	8	Ushbp1
1.039285433	4.15E-38	3.11E-42	8	Hoxa5
0.833849355	4.76E-38	3.56E-42	8	Mn1
2.490597974	5.25E-38	3.93E-42	8	Ccdc80
2.500720518	5.96E-38	4.46E-42	8	Sod3
0.839604517	7.13E-38	5.34E-42	8	Fam109b
0.929748887	7.86E-38	5.89E-42	8	Ntn4
1.598365173	1.08E-37	8.12E-42	8	Rab4a
1.541432804	1.99E-37	1.49E-41	8	Klk8
2.147678028	3.37E-37	2.53E-41	8	Cyp1b1
3.246086822	3.69E-37	2.77E-41	8	Bgn
1.736761862	9.12E-37	6.83E-41	8	Fbln5
1.434206058	9.89E-37	7.40E-41	8	Gata6
2.39206954	1.03E-36	7.75E-41	8	Pecam1
1.711611537	1.92E-36	1.44E-40	8	Pdgfrb
1.956092629	2.43E-35	1.82E-39	8	Prelp
1.585268039	3.14E-35	2.35E-39	8	Osbpl5
1.811992354	1.65E-34	1.23E-38	8	Col5a2
0.889949399	2.64E-34	1.97E-38	8	Plvap

1.210189378	2.64E-34	1.97E-38	8	Megf6
0.932472521	2.90E-34	2.17E-38	8	Rasgrf2
0.862211742	4.13E-34	3.09E-38	8	Slc4a3
1.040727395	4.23E-34	3.17E-38	8	Fzd6
0.845044241	5.43E-34	4.07E-38	8	Tcte2
3.22320969	1.08E-33	8.08E-38	8	Cpe
2.227245648	1.41E-33	1.05E-37	8	Sdpr
2.078611322	1.41E-33	1.06E-37	8	Mxra8
1.57982902	1.65E-33	1.24E-37	8	Sh3d21
2.215370924	1.22E-32	9.16E-37	8	Fxyd6
1.492231633	1.59E-32	1.19E-36	8	Zfp423
2.455069594	2.34E-32	1.75E-36	8	Mmp2
1.107738105	4.36E-32	3.26E-36	8	Ephb4
1.247360043	4.61E-32	3.45E-36	8	Mcc
1.459264584	5.52E-32	4.13E-36	8	Msrb3
0.951232821	5.87E-31	4.40E-35	8	Bmx
0.992401461	5.87E-31	4.40E-35	8	She
1.037882442	6.33E-31	4.74E-35	8	Baiap3
2.532316952	1.07E-30	8.03E-35	8	Edn1
1.42494459	2.73E-30	2.05E-34	8	Tmem204
1.541809243	3.17E-30	2.38E-34	8	Wwtr1
1.582528946	6.22E-30	4.66E-34	8	Sox5
0.776761849	1.09E-29	8.16E-34	8	Fxyd7
1.359114032	1.89E-29	1.42E-33	8	Card10
0.971794748	4.53E-29	3.39E-33	8	Hap1
1.364801177	2.08E-28	1.56E-32	8	Zfp507
1.985005163	2.13E-28	1.59E-32	8	Cdc42bpa
1.176995245	3.03E-28	2.27E-32	8	Pdzk1ip1
1.241741084	3.03E-28	2.27E-32	8	Gata2
1.361221943	3.28E-28	2.46E-32	8	Foxc2
1.169683731	3.84E-28	2.88E-32	8	Cdh5
0.819294708	5.28E-28	3.96E-32	8	Kcnt2
1.236946243	6.65E-28	4.98E-32	8	Grb10
2.21309258	1.30E-27	9.71E-32	8	Nfib
1.821294747	2.06E-27	1.54E-31	8	Meg3
1.078467723	4.95E-27	3.71E-31	8	Oxld1
1.917855883	6.66E-27	4.98E-31	8	C230052I12Rik
1.432007868	9.09E-27	6.81E-31	8	Rasip1
1.49216333	2.14E-26	1.60E-30	8	Aph1b
1.126820919	4.01E-26	3.00E-30	8	Hid1
1.354530831	5.43E-26	4.07E-30	8	Ppap2a
1.72313876	5.47E-26	4.09E-30	8	Hspg2
0.933490706	6.65E-26	4.98E-30	8	9930014A18Rik
1.373782742	8.63E-26	6.46E-30	8	S100a16
1.346493142	1.22E-25	9.11E-30	8	1700056E22Rik
0.926241971	2.25E-25	1.68E-29	8	Acer2
2.352970678	4.43E-25	3.32E-29	8	Sparc
1.527573946	1.31E-24	9.80E-29	8	Cdc42ep2
2.21650175	4.24E-24	3.17E-28	8	Pam
2.208985083	6.07E-24	4.55E-28	8	Dos
1.753787767	6.32E-24	4.74E-28	8	Itgae
1.755642958	2.47E-23	1.85E-27	8	Tmem158
1.192972834	2.94E-23	2.20E-27	8	Adat2
2.705471234	5.78E-23	4.33E-27	8	Mgp
2.636013803	6.09E-23	4.56E-27	8	Vwf
1.486777783	6.17E-23	4.62E-27	8	Stard13
1.141446702	6.55E-23	4.90E-27	8	Lysmd2
1.116799942	7.36E-23	5.52E-27	8	Spns2
1.87496869	1.07E-22	7.98E-27	8	A630089N07Rik
0.707198934	1.18E-22	8.82E-27	8	Adcy6
1.653055168	1.37E-22	1.03E-26	8	Ece1

2.07367058	2.13E-22	1.59E-26	8	Mfap5
1.933447044	3.58E-22	2.68E-26	8	Antxr1
1.201860871	3.71E-22	2.78E-26	8	Zscan18
1.414679237	3.88E-22	2.91E-26	8	Tenc1
2.564915751	3.97E-22	2.97E-26	8	Fstl1
1.294189129	6.52E-22	4.88E-26	8	Dyrk1b
1.205154462	6.58E-22	4.93E-26	8	Dsel
0.61319863	6.61E-22	4.95E-26	8	Ehd2
1.125877212	8.50E-22	6.36E-26	8	Tex14
2.709984885	1.17E-21	8.76E-26	8	Igfbp7
1.125299022	1.34E-21	1.00E-25	8	2510002D24Rik
1.975656811	2.82E-21	2.11E-25	8	Icam2
1.420680915	4.02E-21	3.01E-25	8	Gtf2ird2
1.571626685	4.55E-21	3.41E-25	8	Lamb2
1.515846969	6.21E-21	4.65E-25	8	D3Ertd254e
1.811464086	2.06E-20	1.54E-24	8	Gstt1
0.529901771	3.02E-20	2.26E-24	8	Inca1
1.841075501	3.08E-20	2.31E-24	8	Fxyd1
1.342651824	3.15E-20	2.36E-24	8	9430020K01Rik
0.631741156	3.33E-20	2.49E-24	8	Cdon
1.19985177	4.56E-20	3.41E-24	8	BC051142
1.400790847	4.93E-20	3.69E-24	8	Smad6
1.529128807	6.27E-20	4.70E-24	8	Sorbs1
1.232117726	7.91E-20	5.93E-24	8	Gbp4
1.039986928	9.28E-20	6.95E-24	8	A530099J19Rik
1.576848741	1.09E-19	8.15E-24	8	Synpo
1.871889515	1.29E-19	9.67E-24	8	Lpar1
2.059591743	2.16E-19	1.62E-23	8	Fhl1
1.029277478	3.64E-19	2.72E-23	8	Nadk2
2.01766279	3.82E-19	2.86E-23	8	Lphn1
0.65822402	4.22E-19	3.16E-23	8	Nek8
1.987867816	5.62E-19	4.21E-23	8	Cav1
1.336155439	6.37E-19	4.77E-23	8	Magee1
0.816526202	8.61E-19	6.45E-23	8	Plat
1.864364262	1.01E-18	7.57E-23	8	Ptrf
0.845526175	2.37E-18	1.78E-22	8	E130311K13Rik
1.275998698	2.81E-18	2.10E-22	8	Tanc1
1.649420744	3.10E-18	2.32E-22	8	Gid4
1.972851125	4.22E-18	3.16E-22	8	Cgnl1
1.021401216	4.41E-18	3.30E-22	8	2810001G20Rik
1.626419818	5.49E-18	4.11E-22	8	Pcdh19
1.122659448	5.85E-18	4.38E-22	8	Gm5512
0.783535489	5.88E-18	4.40E-22	8	Znrd1as
1.206659774	1.87E-17	1.40E-21	8	Dock6
0.787768229	1.95E-17	1.46E-21	8	Mxra7
0.765948727	2.05E-17	1.54E-21	8	Gm527
0.976356223	2.39E-17	1.79E-21	8	Bicd1
0.686718037	2.41E-17	1.81E-21	8	Abca8b
1.039986928	3.08E-17	2.30E-21	8	Top1mt
1.408165107	3.60E-17	2.69E-21	8	Ablim1
1.750342534	3.98E-17	2.98E-21	8	Nfia
1.238641046	4.32E-17	3.24E-21	8	Tacc2
1.365677986	4.56E-17	3.42E-21	8	Pop1
1.350482928	7.35E-17	5.51E-21	8	Rab26os
1.137700431	7.67E-17	5.75E-21	8	Adm
1.123706508	8.78E-17	6.57E-21	8	Ears2
1.703401303	9.25E-17	6.92E-21	8	Htra1
0.896848906	1.50E-16	1.12E-20	8	Nr3c2
1.342950632	1.71E-16	1.28E-20	8	Endog
1.234479597	2.65E-16	1.98E-20	8	Xcl1
1.452423052	3.03E-16	2.27E-20	8	Snora78

1.032647252	4.55E-16	3.41E-20	8	Sox7
0.645343799	5.07E-16	3.80E-20	8	Spaca6
2.006467921	5.74E-16	4.30E-20	8	Slc43a3
1.648750278	9.59E-16	7.18E-20	8	Adamts1
1.175752237	1.13E-15	8.43E-20	8	4933433G15Rik
0.733627236	1.29E-15	9.66E-20	8	Ajuba
1.18826987	1.38E-15	1.03E-19	8	Polr3g
0.941298414	1.54E-15	1.16E-19	8	Itga1
2.785854621	1.74E-15	1.31E-19	8	Dcn
1.470643599	1.76E-15	1.32E-19	8	Cald1
1.104689181	2.82E-15	2.11E-19	8	Pik3ip1
1.149293775	3.65E-15	2.73E-19	8	Bcdin3d
1.887691644	4.24E-15	3.18E-19	8	Xcr1
1.369975982	6.96E-15	5.21E-19	8	Mrpl49
1.147146672	8.15E-15	6.10E-19	8	Klhl2
1.235787977	4.59E-14	3.44E-18	8	Ccdc102a
1.396044759	4.87E-14	3.65E-18	8	Zfp664
1.067538652	6.46E-14	4.84E-18	8	Gm14403
1.084640582	8.71E-14	6.52E-18	8	Smarcd3
1.833122961	9.28E-14	6.95E-18	8	Mical2
1.549655485	1.13E-13	8.44E-18	8	Cyr61
1.643944633	1.25E-13	9.37E-18	8	Ctla2a
1.405517039	1.28E-13	9.61E-18	8	Rhbdd2
1.055959982	1.51E-13	1.13E-17	8	Pdia5
0.876399403	2.39E-13	1.79E-17	8	Palm
0.681343551	2.40E-13	1.79E-17	8	Ggnbp1
1.011156193	2.55E-13	1.91E-17	8	Asap2
1.860174604	2.85E-13	2.14E-17	8	Ift27
1.421659316	3.18E-13	2.38E-17	8	Otud7b
1.070111128	4.59E-13	3.44E-17	8	Bbs4
0.872846522	5.12E-13	3.83E-17	8	Slc9a3r2
0.937870009	5.13E-13	3.84E-17	8	Amotl2
1.323968181	6.01E-13	4.50E-17	8	Elp4
1.704513157	6.21E-13	4.65E-17	8	Fosl1
0.640764364	7.20E-13	5.39E-17	8	Rab19
0.892635205	7.44E-13	5.57E-17	8	2310022B05Rik
1.385337227	8.44E-13	6.32E-17	8	Epn2
0.604102987	9.33E-13	6.99E-17	8	Slc19a2
1.13655692	1.21E-12	9.04E-17	8	Pigc
1.609417655	1.26E-12	9.41E-17	8	Rhbdf1
1.138730187	1.69E-12	1.26E-16	8	Megf8
0.631660045	1.81E-12	1.36E-16	8	Marveld2
1.075364539	2.34E-12	1.76E-16	8	Plekhh3
0.965570517	2.41E-12	1.80E-16	8	Hepacam2
1.528794424	2.50E-12	1.87E-16	8	Nxn
1.500356558	2.65E-12	1.99E-16	8	Cd200
0.89582966	2.96E-12	2.22E-16	8	E130012A19Rik
0.846949084	3.32E-12	2.49E-16	8	Hoxb3
1.161237457	3.38E-12	2.53E-16	8	Cdc23
1.426972013	3.59E-12	2.69E-16	8	Alkbh6
1.289839664	3.61E-12	2.70E-16	8	Gem
0.891122177	6.24E-12	4.67E-16	8	Nr4a3
0.937909792	9.41E-12	7.05E-16	8	Rab11fip2
1.019093613	1.12E-11	8.36E-16	8	Klhl24
1.27600681	1.22E-11	9.13E-16	8	Hebp2
0.747214402	1.24E-11	9.29E-16	8	Ints2
1.063894454	1.51E-11	1.13E-15	8	Tctn3
1.499917902	1.98E-11	1.48E-15	8	Areg
0.866670348	2.00E-11	1.49E-15	8	6720489N17Rik
1.499737543	2.59E-11	1.94E-15	8	2900026A02Rik
1.290401133	2.77E-11	2.07E-15	8	Spef1

1.252513679	2.86E-11	2.14E-15	8	Pcnx
1.619274179	2.87E-11	2.15E-15	8	Vcam1
0.874972964	3.32E-11	2.49E-15	8	Dbt
1.382880223	4.61E-11	3.45E-15	8	Tagln
1.245188862	4.94E-11	3.70E-15	8	Tpgs2
1.496254028	5.14E-11	3.85E-15	8	Ifi205
0.828008361	5.66E-11	4.24E-15	8	Lcor
1.313734647	5.99E-11	4.49E-15	8	Tbc1d10c
1.749787783	6.36E-11	4.76E-15	8	Nbeal1
1.334871525	6.67E-11	5.00E-15	8	Rad52
0.508743545	6.90E-11	5.17E-15	8	Gpr82
0.936385209	8.60E-11	6.44E-15	8	Abi2
1.093877787	1.06E-10	7.96E-15	8	Homer3
1.1695359	1.11E-10	8.30E-15	8	Leprel2
1.199842599	1.27E-10	9.48E-15	8	Ifit1
1.400131729	1.30E-10	9.76E-15	8	Fbxo25
1.336312598	1.31E-10	9.80E-15	8	Dpysl3
0.785032998	1.74E-10	1.31E-14	8	Acyp1
0.928661581	1.79E-10	1.34E-14	8	B3gnt7
1.097245234	1.88E-10	1.41E-14	8	Ttc38
0.53027849	1.97E-10	1.48E-14	8	Ildr1
0.785644251	2.03E-10	1.52E-14	8	Htra4
1.067682313	2.43E-10	1.82E-14	8	Tmem9
1.235556221	2.71E-10	2.03E-14	8	Acsf3
1.177022025	2.94E-10	2.20E-14	8	Taf4b
0.812764557	3.62E-10	2.71E-14	8	D3Ertd751e
0.899318163	5.22E-10	3.91E-14	8	Plxdc2
0.937779425	5.22E-10	3.91E-14	8	Phykpl
1.273040469	5.96E-10	4.47E-14	8	Gng11
0.778227473	9.53E-10	7.14E-14	8	Myom1
0.995297297	1.01E-09	7.59E-14	8	Slc2a4rg-ps
1.276063713	1.11E-09	8.28E-14	8	Mta1
0.806900375	1.17E-09	8.77E-14	8	Cryzl1
1.436436328	1.20E-09	8.98E-14	8	Epas1
0.770376778	1.32E-09	9.86E-14	8	Naip6
0.959839735	1.34E-09	1.00E-13	8	Zfp518b
1.160076601	1.39E-09	1.04E-13	8	Golim4
0.797373909	1.59E-09	1.19E-13	8	Map6
1.141532094	1.72E-09	1.29E-13	8	Trim62
1.518600816	1.73E-09	1.30E-13	8	Syne2
0.999043417	1.74E-09	1.30E-13	8	Ikbip
0.980829253	1.83E-09	1.37E-13	8	Zdhhc24
1.420734058	1.86E-09	1.39E-13	8	Arl16
0.90954287	1.98E-09	1.48E-13	8	Rab33b
0.515859414	2.88E-09	2.16E-13	8	Mr1
0.415352829	2.95E-09	2.21E-13	8	Sipa1l2
0.826014342	3.25E-09	2.43E-13	8	Slc40a1
1.572817329	3.51E-09	2.63E-13	8	Mfsd3
0.806482417	3.52E-09	2.63E-13	8	Fam53a
0.879327919	3.52E-09	2.63E-13	8	Sel1l3
0.764489405	3.54E-09	2.65E-13	8	Ifng
0.862341744	3.88E-09	2.91E-13	8	Sgce
0.742245267	4.00E-09	3.00E-13	8	Gzma
0.655687759	4.49E-09	3.36E-13	8	Ccdc137
0.820780758	5.67E-09	4.25E-13	8	Zfp120
1.255778307	5.96E-09	4.47E-13	8	Kdelc2
0.967890071	6.67E-09	4.99E-13	8	Mrpl9
0.570723765	7.86E-09	5.89E-13	8	6530402F18Rik
0.922487216	9.10E-09	6.81E-13	8	Sdcbp2
1.091981442	9.59E-09	7.18E-13	8	Cyth3
0.634576651	1.03E-08	7.70E-13	8	Zgpat

1.058679883	1.11E-08	8.34E-13	8	B3glct
0.906096497	1.46E-08	1.10E-12	8	Usp53
0.898853473	1.47E-08	1.10E-12	8	Rnase6
1.264914787	1.54E-08	1.15E-12	8	Zbtb22
0.936558064	1.84E-08	1.38E-12	8	Gpr160
1.291584052	1.93E-08	1.45E-12	8	Zfp26
1.315079491	1.98E-08	1.48E-12	8	Cycs
0.997526011	1.99E-08	1.49E-12	8	Id1
0.829335447	2.06E-08	1.54E-12	8	Med16
0.927318546	2.12E-08	1.59E-12	8	Eva1b
1.29497415	2.17E-08	1.62E-12	8	Mrps11
1.177972979	2.38E-08	1.78E-12	8	Bpgm
1.720994912	2.47E-08	1.85E-12	8	Serpinh1
1.181857794	2.58E-08	1.93E-12	8	Dcp1b
1.630986249	2.85E-08	2.13E-12	8	Bphl
0.586633576	2.95E-08	2.21E-12	8	Gins4
0.848704066	3.29E-08	2.46E-12	8	Mar.07
0.908981932	3.42E-08	2.56E-12	8	Gimap4
0.73419126	3.99E-08	2.99E-12	8	Gzmb
0.971298888	4.62E-08	3.46E-12	8	Bcs1l
0.461582527	4.73E-08	3.54E-12	8	Ifi44
1.18546624	4.88E-08	3.65E-12	8	Cox18
0.918325413	5.27E-08	3.95E-12	8	Pak4
0.944800911	5.42E-08	4.06E-12	8	Pex14
0.840887264	5.83E-08	4.37E-12	8	Grpel2
1.111765417	6.14E-08	4.60E-12	8	Slc39a14
0.957282166	6.82E-08	5.11E-12	8	Rcn3
1.266295535	7.05E-08	5.28E-12	8	Ly6a
1.12990562	7.45E-08	5.58E-12	8	Uhmk1
0.961373788	8.36E-08	6.26E-12	8	Sptlc1
1.197597819	9.19E-08	6.88E-12	8	H2-Q4
1.144121693	9.69E-08	7.26E-12	8	Kremen1
1.026776629	1.03E-07	7.72E-12	8	Usp39
1.536389931	1.12E-07	8.37E-12	8	Crbn
0.589157467	1.40E-07	1.05E-11	8	Camk4
0.643791685	1.41E-07	1.05E-11	8	Med14
0.715499127	1.56E-07	1.17E-11	8	Lysmd3
0.649451071	1.66E-07	1.24E-11	8	Cd28
0.766136962	1.70E-07	1.27E-11	8	Apoe
1.081758791	2.04E-07	1.53E-11	8	Oasl1
0.991515337	2.29E-07	1.71E-11	8	Ubxn2a
0.658071899	2.43E-07	1.82E-11	8	Sugp1
0.940300731	2.47E-07	1.85E-11	8	Ginm1
1.075772303	2.67E-07	2.00E-11	8	Lrrc42
0.723396628	2.77E-07	2.07E-11	8	Vars
0.531666996	3.03E-07	2.27E-11	8	Gm11696
1.024833699	3.26E-07	2.44E-11	8	Itpr2
0.636480207	3.75E-07	2.81E-11	8	Slc22a15
0.934549217	3.82E-07	2.86E-11	8	Cd24a
1.059167987	3.86E-07	2.89E-11	8	Csrp2
1.373092849	4.27E-07	3.20E-11	8	Crim1
1.013160981	4.55E-07	3.41E-11	8	AW549877
0.733850875	4.75E-07	3.56E-11	8	Nol11
0.805496368	5.32E-07	3.98E-11	8	1110059G10Rik
1.291811806	5.37E-07	4.02E-11	8	Aven
1.19918147	5.52E-07	4.13E-11	8	Gm16523
0.814465106	5.79E-07	4.34E-11	8	Gm20324
0.527354926	6.01E-07	4.50E-11	8	Kif1a
0.71347411	6.05E-07	4.53E-11	8	Whamm
1.091709976	7.06E-07	5.29E-11	8	Lmf2
0.683086734	7.53E-07	5.64E-11	8	Cobll1

1.549417222	7.54E-07	5.65E-11	8	Ptar1
0.686425635	8.86E-07	6.63E-11	8	Lsm1
1.285652025	9.15E-07	6.85E-11	8	Slfn10-ps
0.704003237	1.00E-06	7.51E-11	8	Slc23a3
1.035599447	1.04E-06	7.76E-11	8	Fnbp1l
0.9248034	1.10E-06	8.21E-11	8	Pogk
0.612210418	1.10E-06	8.23E-11	8	Socs5
1.234154973	1.36E-06	1.02E-10	8	Ctdspl
1.460819155	1.40E-06	1.05E-10	8	Map3k12
0.821244858	1.41E-06	1.05E-10	8	Gprc5a
1.058223185	1.50E-06	1.12E-10	8	Hspbp1
0.920061717	1.56E-06	1.17E-10	8	Clstn1
0.826794743	1.91E-06	1.43E-10	8	Myh10
1.221661026	2.12E-06	1.59E-10	8	Tcf12
0.678434458	2.22E-06	1.66E-10	8	Zfp788
1.169321346	2.23E-06	1.67E-10	8	Arhgap23
1.487840402	2.38E-06	1.78E-10	8	Selo
1.185953638	2.40E-06	1.80E-10	8	Ogfod2
0.75252381	2.45E-06	1.83E-10	8	Plgrkt
0.732102148	2.45E-06	1.84E-10	8	Cdc14a
0.850946099	2.55E-06	1.91E-10	8	Map3k3
1.188862862	2.70E-06	2.02E-10	8	Ranbp10
1.076734119	2.88E-06	2.16E-10	8	C030006K11Rik
1.05424942	3.47E-06	2.60E-10	8	Prpf18
0.929877387	3.68E-06	2.76E-10	8	Oaf
0.723348817	3.84E-06	2.88E-10	8	0610009L18Rik
0.876399403	4.19E-06	3.14E-10	8	Cd3eap
0.638700893	4.42E-06	3.31E-10	8	Tmem106b
0.908550195	4.62E-06	3.46E-10	8	Ankrd46
0.60558363	4.73E-06	3.54E-10	8	Sp100
1.690207629	5.10E-06	3.82E-10	8	Maged2
1.086220289	5.73E-06	4.29E-10	8	Id3
0.977863023	5.83E-06	4.36E-10	8	Trio
1.081760449	6.23E-06	4.67E-10	8	Ift122
1.035030137	6.52E-06	4.88E-10	8	Myo1c
0.918369303	7.51E-06	5.62E-10	8	Ubap2
0.594190965	7.73E-06	5.79E-10	8	Zfp945
0.806636713	7.92E-06	5.93E-10	8	Sptbn1
1.005808676	8.14E-06	6.09E-10	8	Hmgn3
0.669346303	8.14E-06	6.10E-10	8	Pwwp2a
1.154430713	8.21E-06	6.15E-10	8	Hmgcr
0.756176723	8.31E-06	6.23E-10	8	Fmnl2
0.740363317	8.43E-06	6.31E-10	8	Rcn1
1.219632658	8.49E-06	6.36E-10	8	Mfge8
0.845435122	8.70E-06	6.51E-10	8	Cnih1
1.032265621	9.04E-06	6.77E-10	8	Ilvbl
0.64600836	9.20E-06	6.89E-10	8	Kif13a
0.734516182	1.03E-05	7.73E-10	8	Efemp2
0.86170345	1.06E-05	7.91E-10	8	Myo6
0.768363822	1.08E-05	8.09E-10	8	Erlec1
1.004842492	1.09E-05	8.20E-10	8	Impa2
1.041910127	1.10E-05	8.25E-10	8	Mapk9
0.789787148	1.12E-05	8.41E-10	8	Ago1
0.468454442	1.16E-05	8.66E-10	8	Zfp869
0.905837767	1.18E-05	8.87E-10	8	Acad9
0.786688609	1.27E-05	9.55E-10	8	Fam114a2
0.638571529	1.28E-05	9.62E-10	8	Nrp
1.133039516	1.32E-05	9.90E-10	8	Tex264
1.156635313	1.33E-05	9.95E-10	8	Cntln
1.310416898	1.51E-05	1.13E-09	8	Hyal1
1.00807033	1.60E-05	1.20E-09	8	Cers4

0.758616367	1.68E-05	1.26E-09	8	Cmtm8
0.660690362	1.72E-05	1.29E-09	8	Pycr2
0.860267335	1.75E-05	1.31E-09	8	Scimp
0.892135092	1.82E-05	1.36E-09	8	Gmpr2
0.665053313	1.84E-05	1.38E-09	8	Ddx23
1.074811411	1.87E-05	1.40E-09	8	Wrb
0.983261921	1.90E-05	1.42E-09	8	Zmym3
0.732546801	1.94E-05	1.46E-09	8	Zwint
0.731215325	1.96E-05	1.47E-09	8	Trub1
0.756797672	1.96E-05	1.47E-09	8	Chst14
0.633798617	2.11E-05	1.58E-09	8	Ldlrad4
-1.173037137	2.14E-05	1.60E-09	8	Lgmn
1.105361623	2.14E-05	1.60E-09	8	Ccdc93
1.335621704	2.21E-05	1.65E-09	8	Gpr84
1.014156362	2.24E-05	1.68E-09	8	Arid5b
0.842151082	2.25E-05	1.69E-09	8	Fam3c
0.784949869	2.33E-05	1.74E-09	8	Ifnb1
0.931897391	2.35E-05	1.76E-09	8	Rasgrp3
1.139391172	2.43E-05	1.82E-09	8	Zfp652
0.722113343	2.62E-05	1.96E-09	8	Itpr1
0.616832343	2.65E-05	1.98E-09	8	Cdhr3
0.932755911	2.96E-05	2.22E-09	8	Dcaf17
0.611659344	3.06E-05	2.29E-09	8	Osgin2
0.958774169	3.24E-05	2.42E-09	8	Prkcsh
0.568122984	3.27E-05	2.45E-09	8	Nr6a1
0.751657397	3.31E-05	2.48E-09	8	Fndc4
0.798165757	3.35E-05	2.51E-09	8	Zbtb4
0.697575576	3.38E-05	2.53E-09	8	Tmtc4
0.817784064	3.41E-05	2.55E-09	8	Hmgn5
0.825452433	3.42E-05	2.56E-09	8	Hscb
1.119913131	3.43E-05	2.57E-09	8	Nmb
0.316126585	3.52E-05	2.64E-09	8	Irg1
1.388038392	3.60E-05	2.70E-09	8	Adss
0.613848192	3.74E-05	2.80E-09	8	Ncbp1
0.805248712	3.80E-05	2.85E-09	8	Cep57
0.483645295	3.88E-05	2.91E-09	8	Fpr2
0.622638775	3.93E-05	2.95E-09	8	Prpf40b
0.813623442	3.99E-05	2.99E-09	8	Apopt1
1.076852996	4.09E-05	3.06E-09	8	Insl6
-1.065348569	4.31E-05	3.23E-09	8	Pfn1
0.738007445	4.34E-05	3.25E-09	8	Spry2
0.934636552	4.54E-05	3.40E-09	8	Utrn
0.61536948	5.05E-05	3.78E-09	8	Fh1
1.226056472	5.17E-05	3.87E-09	8	Cog2
0.910566446	5.46E-05	4.09E-09	8	Ppp6r3
1.037628539	5.48E-05	4.10E-09	8	Adpgk
0.733500726	5.58E-05	4.18E-09	8	Tsen54
0.350564655	5.65E-05	4.23E-09	8	Dnmbp
1.108331428	5.91E-05	4.42E-09	8	Jag1
0.8318262	6.20E-05	4.64E-09	8	Ptplad1
0.80556584	6.38E-05	4.78E-09	8	Glg1
1.012728	6.43E-05	4.81E-09	8	Lrsam1
0.587795749	6.45E-05	4.83E-09	8	Trmt12
0.722508598	6.56E-05	4.91E-09	8	Hnmt
0.750092356	7.10E-05	5.32E-09	8	Il18bp
0.951879273	7.13E-05	5.34E-09	8	Fn1
0.72316011	7.18E-05	5.38E-09	8	Phkb
0.72884097	7.30E-05	5.47E-09	8	Vps37c
0.616226099	7.31E-05	5.48E-09	8	Mbtps1
0.601383873	7.41E-05	5.55E-09	8	Srpk2
0.82087388	7.70E-05	5.77E-09	8	Sptan1

0.774977199	8.15E-05	6.10E-09	8	Angptl2
0.529901771	8.36E-05	6.26E-09	8	Zc3h6
0.722226242	9.01E-05	6.75E-09	8	Rapgef5
1.518886797	9.29E-05	6.96E-09	8	Cdk6
0.403353175	9.48E-05	7.10E-09	8	Slc2a8
0.523671221	9.95E-05	7.45E-09	8	Slc4a7
0.764101588	0.000100866	7.55E-09	8	Mlxip
0.6193543	0.000101403	7.59E-09	8	Sema6d
0.626158901	0.000101712	7.62E-09	8	Trmt61b
0.580973016	0.00010598	7.94E-09	8	Tyw5
1.208407401	0.000106239	7.96E-09	8	Setd5
1.110278769	0.000116621	8.73E-09	8	Alkbh1
0.650202767	0.000118113	8.85E-09	8	Hes1
0.693808075	0.000118699	8.89E-09	8	Haao
0.789453712	0.000121837	9.13E-09	8	Cd300lg
0.685077404	0.000124483	9.32E-09	8	H2-M2
0.639968178	0.000127926	9.58E-09	8	Timeless
1.037627304	0.000129091	9.67E-09	8	Srp68
0.701346367	0.000133599	1.00E-08	8	Chchd6
0.409215285	0.000134309	1.01E-08	8	Plac8
0.518851935	0.000138636	1.04E-08	8	B930059L03Rik
0.902142071	0.000142545	1.07E-08	8	Man1a
0.764218623	0.000143342	1.07E-08	8	Dgke
0.688542598	0.000144018	1.08E-08	8	Soga1
0.942093362	0.000144848	1.08E-08	8	Fars2
0.479735443	0.000155169	1.16E-08	8	Slc19a1
0.921891476	0.000169426	1.27E-08	8	Icam1
0.706873678	0.000170478	1.28E-08	8	Tigd2
0.610137734	0.000173651	1.30E-08	8	Cfl2
0.539530033	0.000187532	1.40E-08	8	Kmo
0.978031266	0.000203915	1.53E-08	8	Usp31
0.709692099	0.000204526	1.53E-08	8	Lyst
0.959464431	0.000206304	1.55E-08	8	Lman2l
1.021760018	0.000208291	1.56E-08	8	Rasgrp4
0.38967243	0.000213674	1.60E-08	8	St3gal5
1.003883673	0.000226037	1.69E-08	8	Cd19
0.525314607	0.000234595	1.76E-08	8	Usp22
0.535144247	0.000235305	1.76E-08	8	Rit1
0.980829253	0.000238826	1.79E-08	8	Agps
0.872967396	0.000243036	1.82E-08	8	Pbx1
0.328752721	0.000247477	1.85E-08	8	Chmp1a
0.858512272	0.00025699	1.92E-08	8	Zfp574
0.734883642	0.000264434	1.98E-08	8	Zfp768
0.842376679	0.000266688	2.00E-08	8	Cept1
0.874542875	0.000269405	2.02E-08	8	F2r
0.857909799	0.000284883	2.13E-08	8	Selm
0.815931128	0.000284906	2.13E-08	8	Flot1
0.647217626	0.000285089	2.14E-08	8	Igflr1
0.946645194	0.000287167	2.15E-08	8	Rilp
0.99814855	0.000297198	2.23E-08	8	Mkks
0.680635562	0.000311214	2.33E-08	8	Rps6kc1
0.794699549	0.000340871	2.55E-08	8	Zfp758
1.040494484	0.000346053	2.59E-08	8	Ercc4
0.651715909	0.000364018	2.73E-08	8	Tbc1d16
0.705768018	0.000364569	2.73E-08	8	Mrpl2
1.179563839	0.000365905	2.74E-08	8	Spin1
0.911229892	0.000366536	2.75E-08	8	Kctd10
0.751657397	0.000392059	2.94E-08	8	Rpusd4
0.881208129	0.00039884	2.99E-08	8	Il6
0.89711422	0.000409179	3.06E-08	8	Btla
0.644242462	0.000412601	3.09E-08	8	Letm2

0.949705296	0.000422872	3.17E-08	8	Dusp8
1.152356565	0.000438302	3.28E-08	8	Akap2
0.554481221	0.000442778	3.32E-08	8	Ermard
0.831305909	0.000449694	3.37E-08	8	Dohh
1.142699824	0.000450993	3.38E-08	8	E4f1
1.137628412	0.000462663	3.47E-08	8	Lamc1
0.557256654	0.000466847	3.50E-08	8	Nub1
0.973210465	0.000468856	3.51E-08	8	Gpatch2l
0.629108421	0.000469327	3.52E-08	8	Gabpb2
0.969716359	0.000469807	3.52E-08	8	Prpsap1
0.99395628	0.00047139	3.53E-08	8	Eif2b2
0.938083745	0.000478919	3.59E-08	8	Ednrb
1.052753168	0.000490964	3.68E-08	8	Hirip3
0.722212447	0.000491122	3.68E-08	8	Fam210a
1.773851683	0.000508557	3.81E-08	8	Ppm1m
0.621964781	0.000534028	4.00E-08	8	Pura
0.394788297	0.000548954	4.11E-08	8	Araf
0.755962599	0.000571469	4.28E-08	8	Prkacb
0.86842028	0.000582569	4.36E-08	8	Nek9
0.838126895	0.000584979	4.38E-08	8	Tmem173
0.669410334	0.000610585	4.57E-08	8	Kank2
0.950554087	0.000626134	4.69E-08	8	Spib
0.764334067	0.000630333	4.72E-08	8	Dst
0.963201678	0.000645935	4.84E-08	8	Inpp5f
0.91644141	0.000648385	4.86E-08	8	Ms4a1
0.929448355	0.00068003	5.09E-08	8	Tex30
0.563803323	0.000688528	5.16E-08	8	Ccdc32
0.623304938	0.000700679	5.25E-08	8	Wdr44
0.911316321	0.00078057	5.85E-08	8	Ercc1
1.306802238	0.000806112	6.04E-08	8	Ptgfrn
0.499416053	0.000811661	6.08E-08	8	Nfil3
0.405379612	0.000818638	6.13E-08	8	Phb
0.517505801	0.000838811	6.28E-08	8	Trmt2a
0.876688567	0.000865263	6.48E-08	8	Gabarapl1
0.902577056	0.000876247	6.56E-08	8	Wdr77
0.444583281	0.000882804	6.61E-08	8	Nfix
0.401068899	0.000883953	6.62E-08	8	Mtmr9
0.64238392	0.000928815	6.96E-08	8	Gpatch8
0.738755282	0.000934733	7.00E-08	8	Pcmtd1
0.87368197	0.000968784	7.26E-08	8	Gas2l1
0.843160086	0.000994674	7.45E-08	8	Jdp2
1.098162013	0.00103908	7.78E-08	8	Zfp35
0.670571298	0.001049054	7.86E-08	8	Mff
0.748620021	0.001098042	8.22E-08	8	Ankrd17
0.723282216	0.001175772	8.81E-08	8	Eif4g1
0.862740503	0.001209222	9.06E-08	8	Otud5
0.856163684	0.001262306	9.45E-08	8	F11r
0.843996906	0.001292076	9.68E-08	8	Hyal2
0.815808982	0.00135765	1.02E-07	8	Ppfibp1
0.532587366	0.001378472	1.03E-07	8	Timm9
0.965173196	0.001393832	1.04E-07	8	Diap1
0.952880461	0.001419056	1.06E-07	8	Gpd2
0.89418826	0.00144785	1.08E-07	8	Dnajb2
0.958890707	0.001473911	1.10E-07	8	Sorbs3
0.799867727	0.001479745	1.11E-07	8	Dpp7
2.701455472	0.001624476	1.22E-07	8	Serping1
0.760277361	0.001625478	1.22E-07	8	Zbtb11
0.818759502	0.001633315	1.22E-07	8	Snx16
1.106565689	0.001667619	1.25E-07	8	Slc25a25
0.970787693	0.001669229	1.25E-07	8	B3gnt5
1.299975951	0.001675216	1.25E-07	8	Crip2

1.210456395	0.001707751	1.28E-07	8	Nuak1
0.653844087	0.001720171	1.29E-07	8	Zfp715
0.649171059	0.001732869	1.30E-07	8	Aatf
0.902939572	0.001738268	1.30E-07	8	Gpsm1
0.680474629	0.00175894	1.32E-07	8	Sec24d
0.882848845	0.001769708	1.33E-07	8	Laptm4b
1.109549603	0.001803032	1.35E-07	8	Clcn3
0.534911641	0.001853815	1.39E-07	8	1700123O20Rik
1.357850992	0.001891844	1.42E-07	8	E230008N13Rik
0.712827955	0.001928507	1.44E-07	8	Emc3
0.707788731	0.001947355	1.46E-07	8	Add3
0.691956966	0.001999778	1.50E-07	8	Tmed3
0.740250412	0.002021942	1.51E-07	8	Ntan1
0.834460714	0.002075889	1.55E-07	8	Pilrb1
2.05823799	0.002113738	1.58E-07	8	Aebp1
0.895827475	0.002151058	1.61E-07	8	Rhoq
0.874139121	0.002157508	1.62E-07	8	Fblim1
0.818700651	0.002175171	1.63E-07	8	Dopey1
1.017139477	0.002189322	1.64E-07	8	Fam78a
0.844126199	0.002208129	1.65E-07	8	Hdac5
0.435243482	0.002262759	1.69E-07	8	Sec13
1.12454113	0.002273161	1.70E-07	8	Lrrc32
0.891753603	0.002277666	1.71E-07	8	1810032O08Rik
0.786381742	0.002351141	1.76E-07	8	Ptges2
1.046014199	0.002454141	1.84E-07	8	Havcr2
0.886181942	0.002509991	1.88E-07	8	Dkc1
0.501015458	0.002574482	1.93E-07	8	Gopc
1.940870824	0.002593456	1.94E-07	8	Rarres2
0.729139801	0.002596808	1.94E-07	8	Men1
-0.497354603	0.002619535	1.96E-07	8	Ctsb
0.652168528	0.002738448	2.05E-07	8	Ppp2r5e
1.234343076	0.002868066	2.15E-07	8	Zrsr1
0.755874178	0.002872537	2.15E-07	8	Frrs1
0.440481583	0.00290335	2.17E-07	8	Map3k4
0.695951547	0.002916157	2.18E-07	8	Rusc1
0.948577111	0.002993813	2.24E-07	8	Col4a2
0.559285504	0.003034395	2.27E-07	8	1700021K19Rik
0.551668329	0.003169924	2.37E-07	8	Rapgef1
0.6924207	0.003243549	2.43E-07	8	Hdhd2
0.725778842	0.003363089	2.52E-07	8	Lpp
0.655375595	0.003425893	2.57E-07	8	Ampd2
0.874355698	0.003429387	2.57E-07	8	Tceanc2
0.847361334	0.003523891	2.64E-07	8	Aga
0.667210584	0.003549638	2.66E-07	8	Nenf
0.571577821	0.003625222	2.72E-07	8	Chtop
1.095823952	0.003745271	2.81E-07	8	Osbpl7
0.401687519	0.003865524	2.90E-07	8	Parp9
0.510213816	0.003872744	2.90E-07	8	Pop5
0.83113908	0.003963121	2.97E-07	8	Ubfd1
0.494248668	0.003984451	2.98E-07	8	Cox14
0.495379146	0.004193611	3.14E-07	8	Fam78b
1.151944418	0.004268895	3.20E-07	8	Mrpl1
0.435470326	0.004560465	3.42E-07	8	Pi4k2b
0.882868637	0.004639035	3.47E-07	8	Aspa
0.539769418	0.004762684	3.57E-07	8	Ndufaf2
0.473264755	0.004815082	3.61E-07	8	Blk
0.630001703	0.004835279	3.62E-07	8	Psmd10
0.792423652	0.004894627	3.67E-07	8	Mrpl11
0.45294073	0.005122666	3.84E-07	8	Plekhg2
0.647684806	0.005146051	3.85E-07	8	Firre
0.746181877	0.005189951	3.89E-07	8	Imp3

0.916290732	0.005214644	3.91E-07	8	Fam199x
0.603143709	0.005230739	3.92E-07	8	Scyl2
0.429135124	0.005546528	4.15E-07	8	Fance
0.796955376	0.005747849	4.30E-07	8	Cpsf1
0.570039463	0.005810944	4.35E-07	8	Scd1
0.386346709	0.005896751	4.42E-07	8	Thada
0.715627809	0.005964172	4.47E-07	8	Dhx16
0.673457884	0.006062692	4.54E-07	8	Enoph1
0.757577541	0.006089031	4.56E-07	8	Rif1
0.677389961	0.006090007	4.56E-07	8	Cenpw
0.708154482	0.006196074	4.64E-07	8	Slmo2
1.013628766	0.006263206	4.69E-07	8	Cdc42ep3
0.776761849	0.006402948	4.80E-07	8	Zdhhc17
0.496479637	0.006989791	5.24E-07	8	Gorab
0.483524033	0.007054382	5.28E-07	8	Arhgap31
0.943126375	0.007492521	5.61E-07	8	Ctsf
1.025854102	0.007590346	5.68E-07	8	Zfp580
0.542549726	0.007753851	5.81E-07	8	Ube2h
0.703605596	0.00782847	5.86E-07	8	Hectd1
0.829328627	0.007915261	5.93E-07	8	5830418K08Rik
0.663027469	0.008106067	6.07E-07	8	Ptgs1
0.580745324	0.008116207	6.08E-07	8	Polr2c
0.521131533	0.008187852	6.13E-07	8	Zfp62
0.741188709	0.008201969	6.14E-07	8	Slc25a28
0.288187761	0.008243599	6.17E-07	8	Lsr
0.675187214	0.008789517	6.58E-07	8	Cwc27
0.752706626	0.008889479	6.66E-07	8	Nol8
0.54909572	0.008905765	6.67E-07	8	Rsf1
0.649748791	0.008993145	6.74E-07	8	Pla2g16
0.885506103	0.009123362	6.83E-07	8	Drosha
1.300009993	0.009326497	6.99E-07	8	AI837181
0.715789256	0.009469517	7.09E-07	8	Tmem57
0.557681335	0.009622268	7.21E-07	8	St7l
0.75353277	0.009749839	7.30E-07	8	Msl3
0.45395514	0.01004458	7.52E-07	8	Mettl25
0.649096058	0.010063605	7.54E-07	8	Srrt
0.996735825	0.010126385	7.58E-07	8	Hspb11
0.683024173	0.010274932	7.70E-07	8	Gmeb2
0.575259864	0.01027569	7.70E-07	8	Ccnd2
1.01228141	0.010319274	7.73E-07	8	Calcrl
0.33086411	0.010371418	7.77E-07	8	Snrnp25
0.609965887	0.010657536	7.98E-07	8	Hnrnph3
0.572529146	0.010767409	8.06E-07	8	Plbd2
0.374832973	0.011044243	8.27E-07	8	P2ry10
0.41709133	0.011281176	8.45E-07	8	Tap1
0.538159746	0.011295534	8.46E-07	8	Med13l
0.625675282	0.011310761	8.47E-07	8	Adipor2
0.633827772	0.012183907	9.13E-07	8	Aldh4a1
-0.429956965	0.012336092	9.24E-07	8	Actb
0.643654086	0.012364598	9.26E-07	8	Axl
0.716262482	0.012392197	9.28E-07	8	Pddc1
0.524955347	0.012421339	9.30E-07	8	Ccdc28a
0.608998424	0.012457711	9.33E-07	8	Osgin1
0.896606889	0.012479205	9.35E-07	8	Rrp9
0.682105812	0.012695533	9.51E-07	8	Lbr
0.54731346	0.013145167	9.85E-07	8	Gdi1
0.761263984	0.013177118	9.87E-07	8	Mpc2
0.818008146	0.013335566	9.99E-07	8	Timm22
0.761981932	0.01339092	1.00E-06	8	Mrps23
0.978650436	0.014072792	1.05E-06	8	Denr
0.35734789	0.014451727	1.08E-06	8	Hspa14

0.588483304	0.01451567	1.09E-06	8	Pkd1
0.34439767	0.014942768	1.12E-06	8	Phactr2
0.559823451	0.015211063	1.14E-06	8	Map3k9
0.667138871	0.015579589	1.17E-06	8	Snhg9
0.590096932	0.015698781	1.18E-06	8	Gm16062
0.847438986	0.015962426	1.20E-06	8	Cd59a
0.421786432	0.016250036	1.22E-06	8	Fam73a
0.702794237	0.016365613	1.23E-06	8	4931406C07Rik
0.788384689	0.017010116	1.27E-06	8	Zfand2b
0.505829718	0.017034708	1.28E-06	8	Zfp644
1.309015942	0.017052361	1.28E-06	8	Dtx2
0.699132912	0.017059645	1.28E-06	8	Zmynd11
0.675074034	0.017419095	1.30E-06	8	Ddt
0.537653015	0.017630998	1.32E-06	8	Camsap2
0.895246885	0.01776805	1.33E-06	8	Zfp512
0.487982339	0.017992296	1.35E-06	8	Crebrf
0.390180425	0.018047174	1.35E-06	8	Pibf1
0.597090032	0.01820032	1.36E-06	8	Spty2d1
0.67326699	0.018211042	1.36E-06	8	Bod1
0.936121881	0.018329523	1.37E-06	8	Slc39a6
1.169014729	0.018575914	1.39E-06	8	Irak3
0.677985422	0.018679386	1.40E-06	8	Stk40
1.113974411	0.018941581	1.42E-06	8	Cactin
0.524863977	0.01992141	1.49E-06	8	Nudt1
0.606793249	0.020190951	1.51E-06	8	Klhl21
0.649942993	0.020264118	1.52E-06	8	Abcf2
0.394309796	0.02038708	1.53E-06	8	Abcc5
0.535595157	0.020471329	1.53E-06	8	Gm14005
0.367467233	0.020511078	1.54E-06	8	Birc2
0.4248953	0.020591528	1.54E-06	8	Atg10
0.335092375	0.021171631	1.59E-06	8	Qpctl
0.489600985	0.021497478	1.61E-06	8	Rnf113a2
0.964570385	0.022496308	1.68E-06	8	Zufsp
0.618955738	0.022505467	1.69E-06	8	Klrb1b
0.501020996	0.022968781	1.72E-06	8	Nxpe5
1.029838836	0.022970463	1.72E-06	8	Fancm
0.681966196	0.023075471	1.73E-06	8	Gmps
0.573729751	0.023308035	1.75E-06	8	Lima1
0.830178792	0.023619303	1.77E-06	8	Mthfr
0.600894643	0.023629953	1.77E-06	8	Ggps1
0.516121311	0.023753345	1.78E-06	8	Mtf2
0.940600239	0.024835626	1.86E-06	8	Plbd1
0.419900876	0.024845257	1.86E-06	8	Zfp410
0.518731184	0.025895916	1.94E-06	8	Tnpo2
0.819535063	0.026076281	1.95E-06	8	Mtmr10
0.816050152	0.026162426	1.96E-06	8	AU022252
0.503128882	0.026216087	1.96E-06	8	Pik3r1
0.53361235	0.026618055	1.99E-06	8	Wdyhv1
0.546463279	0.027211953	2.04E-06	8	Lpar6
0.989750547	0.028000591	2.10E-06	8	Daam1
0.650915827	0.028120793	2.11E-06	8	Dld
0.540379281	0.028717999	2.15E-06	8	Kctd5
-0.453358302	0.029010236	2.17E-06	8	Tmsb4x
0.351461905	0.029722845	2.23E-06	8	Batf3
0.622708627	0.030590648	2.29E-06	8	Sap18
0.516038993	0.030855624	2.31E-06	8	Phf5a
0.840362837	0.031020955	2.32E-06	8	Rwdd2b
0.465683814	0.03118717	2.34E-06	8	Fkrp
0.743737836	0.03206255	2.40E-06	8	Rrp1b
0.562898125	0.032080562	2.40E-06	8	Serpine1
0.304770778	0.032350337	2.42E-06	8	Usp12

0.490055862	0.032434437	2.43E-06	8	Nmral1
0.778128188	0.033440156	2.50E-06	8	Cfh
0.671294672	0.033814348	2.53E-06	8	Ifi35
0.6695436	0.033945991	2.54E-06	8	Ccdc174
0.508868491	0.034322945	2.57E-06	8	Tprgl
0.981351992	0.034369023	2.57E-06	8	Cdt1
0.627766163	0.035384075	2.65E-06	8	Ddx26b
0.700161533	0.035637527	2.67E-06	8	Abcb9
0.307139244	0.035958756	2.69E-06	8	E030024N20Rik
0.739682867	0.03698348	2.77E-06	8	Yme1l1
0.525686608	0.03699576	2.77E-06	8	Slc25a38
0.561252301	0.037663447	2.82E-06	8	Mief1
0.992285226	0.037686393	2.82E-06	8	Narfl
0.383109375	0.0382957	2.87E-06	8	Ppa1
0.727399636	0.039030682	2.92E-06	8	Fech
0.712454568	0.040878512	3.06E-06	8	Ep400
0.502550633	0.041477999	3.11E-06	8	Tap2
0.767553349	0.042205259	3.16E-06	8	Calml4
0.450917825	0.042208982	3.16E-06	8	Slc44a2
0.66951221	0.043596868	3.27E-06	8	Pxn
0.48573929	0.043740298	3.28E-06	8	Syvn1
0.491940039	0.044889155	3.36E-06	8	Ccdc82
-0.507145753	0.045848512	3.43E-06	8	Ctss
0.432437517	0.045959542	3.44E-06	8	Klhl6
1.006280571	0.046234948	3.46E-06	8	2310033P09Rik
0.612222441	0.04625439	3.46E-06	8	Erap1
0.671888408	0.047722154	3.57E-06	8	Rpp14
0.547565274	0.047805964	3.58E-06	8	Atg2b
0.644384473	0.047951487	3.59E-06	8	Ptov1
0.981836276	0.048149578	3.61E-06	8	Calu
0.564093136	0.048316322	3.62E-06	8	1600002K03Rik
0.284926708	0.048316863	3.62E-06	8	Klhl22
0.675658659	0.048436025	3.63E-06	8	Micu3
0.578164215	0.048607954	3.64E-06	8	Akt1
-0.42301978	0.049597016	3.71E-06	8	B2m
0.619340333	0.04965795	3.72E-06	8	Dhdds
0.463746434	0.049922065	3.74E-06	8	Lman1
0.28534048	0.05289384	3.96E-06	8	Kansl1l
0.631979635	0.053396288	4.00E-06	8	Ccpg1
1.044100079	0.053399691	4.00E-06	8	Gramd4
0.831355588	0.054295211	4.07E-06	8	Rras
0.447363823	0.055459318	4.15E-06	8	Casp3
0.477265072	0.055464084	4.15E-06	8	Mmp13
0.319908277	0.055816979	4.18E-06	8	Strn
0.75222565	0.057984974	4.34E-06	8	Grsf1
0.449173962	0.058262179	4.36E-06	8	Cenpa
0.492344359	0.058517594	4.38E-06	8	Ppme1
0.330045518	0.058677145	4.39E-06	8	Pdk1
0.591186333	0.059113569	4.43E-06	8	Fam134c
0.37005307	0.059226787	4.44E-06	8	Cped1
0.656592285	0.063108024	4.73E-06	8	Cdc123
0.375184971	0.063603673	4.76E-06	8	Cdc40
0.541449673	0.063808218	4.78E-06	8	Tmem64
0.508555475	0.064443117	4.83E-06	8	Galc
0.470141597	0.064681606	4.84E-06	8	Pnpla8
0.647039853	0.064821414	4.85E-06	8	Fgfr1op2
0.281691002	0.06504648	4.87E-06	8	Itpripl1
0.624538777	0.066623725	4.99E-06	8	Tmem50b
0.778770213	0.066915389	5.01E-06	8	Cdc37l1
0.512597531	0.068174929	5.11E-06	8	Tnip3
0.683794086	0.068697696	5.15E-06	8	D330023K18Rik

0.409086083	0.069055463	5.17E-06	8	Atp11a
0.823515844	0.069297379	5.19E-06	8	Acss1
1.358513155	0.070546054	5.28E-06	8	Col4a1
0.467618042	0.070624518	5.29E-06	8	Dexi
0.634696131	0.071550018	5.36E-06	8	Chuk
0.548628524	0.071815972	5.38E-06	8	Il2rb
0.550605237	0.072386496	5.42E-06	8	Ptpn4
0.407016163	0.076584928	5.74E-06	8	Ctdsp1
0.659368963	0.077327001	5.79E-06	8	Pygm
0.50625202	0.080127728	6.00E-06	8	Sec23ip
0.772276684	0.081501169	6.10E-06	8	Mfng
0.748052801	0.081987011	6.14E-06	8	Rbpms
0.373754262	0.082899141	6.21E-06	8	Nufip1
0.586494129	0.083444666	6.25E-06	8	Sh3glb2
0.995223639	0.083591296	6.26E-06	8	Eif2b1
0.86363319	0.083794234	6.28E-06	8	Amz1
0.528015388	0.083916122	6.28E-06	8	Slc25a19
0.551691333	0.08402502	6.29E-06	8	5430437J10Rik
0.656682822	0.084232275	6.31E-06	8	Itpripl2
0.89837831	0.08481994	6.35E-06	8	Enpp4
0.675922913	0.085355539	6.39E-06	8	Cltb
0.490477578	0.085410546	6.40E-06	8	Apbb2
1.144227991	0.086367208	6.47E-06	8	Dnm1
0.256108811	0.086861628	6.51E-06	8	Zcchc11
1.727188038	0.088354652	6.62E-06	8	C3
0.721722851	0.088487353	6.63E-06	8	Lrpprc
0.572047392	0.090934448	6.81E-06	8	Ndrg3
0.517049394	0.090962191	6.81E-06	8	AI314180
0.510668502	0.091140582	6.83E-06	8	Ivd
0.661520538	0.091225815	6.83E-06	8	Cpq
0.348602448	0.092039987	6.89E-06	8	Frmd8
0.452072419	0.092116813	6.90E-06	8	Ppp1r15a
0.360116377	0.092415933	6.92E-06	8	Mtmr2
0.359003234	0.093035271	6.97E-06	8	Ccdc66
0.333054312	0.093608992	7.01E-06	8	1190002N15Rik
0.790155332	0.095265551	7.13E-06	8	Hgs
0.994176363	0.097522203	7.30E-06	8	Ankrd11
0.770363187	0.097564735	7.31E-06	8	Slc7a7
0.598007724	0.097966863	7.34E-06	8	Herc6
0.423863519	0.09818199	7.35E-06	8	Casd1
0.371104275	0.099668892	7.46E-06	8	Rsbn1l
0.740728326	0.100352339	7.52E-06	8	Dopey2
0.545181309	0.104068041	7.79E-06	8	Kansl1
0.541005285	0.104422377	7.82E-06	8	Cdk2ap2
0.641856914	0.107291916	8.04E-06	8	Sfr1
0.686003671	0.107449945	8.05E-06	8	Dcaf5
0.413788463	0.108665814	8.14E-06	8	Taf7
0.337577498	0.109327603	8.19E-06	8	Pkp3
-0.399393491	0.110867817	8.30E-06	8	Fcer1g
0.708526466	0.111639146	8.36E-06	8	Synrg
0.621970371	0.11218646	8.40E-06	8	Ccnl2
0.528385741	0.113020493	8.46E-06	8	Smad2
1.06015111	0.114566664	8.58E-06	8	Ddx51
0.364267133	0.11554028	8.65E-06	8	Sh2d1b1
0.563407047	0.115697861	8.67E-06	8	Dhx29
0.469772542	0.117635204	8.81E-06	8	Gtf2e2
0.667462434	0.119870402	8.98E-06	8	BC005561
0.352432032	0.121242976	9.08E-06	8	Elmsan1
0.527300989	0.12128667	9.08E-06	8	Sltm
0.756610388	0.122032589	9.14E-06	8	Rps6ka4
0.551201606	0.123806748	9.27E-06	8	Zc3hav1

0.579157159	0.125480967	9.40E-06	8	Dhx30
0.728391986	0.126596563	9.48E-06	8	Hsdl2
0.442711775	0.128370497	9.61E-06	8	Vezf1
0.631453612	0.129235397	9.68E-06	8	Pmm1
0.689508906	0.129332673	9.69E-06	8	Cmas
0.747428764	0.130124055	9.75E-06	8	Fbxo18
0.484170438	0.130877797	9.80E-06	8	Phf13
0.656459249	0.13208413	9.89E-06	8	Gtf2a2
0.561864615	0.133856962	1.00E-05	8	Kank3
0.288782615	0.134007732	1.00E-05	8	Nae1
0.660452535	0.141299615	1.06E-05	8	Actn1
0.320874481	0.144687703	1.08E-05	8	Epc1
0.648658043	0.147518697	1.10E-05	8	Itga9
0.579334116	0.148451613	1.11E-05	8	Insr
0.291107907	0.149715082	1.12E-05	8	Ddit3
0.756694961	0.152074261	1.14E-05	8	Sla
0.260568837	0.152313953	1.14E-05	8	Clpx
0.373807257	0.15645234	1.17E-05	8	Usp6nl
0.670729886	0.156899939	1.18E-05	8	Dnm2
0.838714117	0.157290965	1.18E-05	8	Rbm10
0.540338559	0.160116194	1.20E-05	8	Mafk
0.410253568	0.160396347	1.20E-05	8	Sf3a1
0.424541255	0.161312363	1.21E-05	8	Vegfa
0.412001164	0.164357782	1.23E-05	8	Zbed6
0.508759334	0.165237703	1.24E-05	8	Fgd6
0.646435587	0.165815924	1.24E-05	8	Rprd1b
0.79449073	0.166555724	1.25E-05	8	Tada2b
1.01973025	0.166650459	1.25E-05	8	Maged1
0.614915849	0.169087768	1.27E-05	8	Psmd1
0.777924918	0.169868105	1.27E-05	8	Rcl1
0.515907358	0.170299564	1.28E-05	8	Eif3h
0.480077369	0.176933243	1.33E-05	8	Syap1
0.668239441	0.179793705	1.35E-05	8	Yipf6
0.555697993	0.180526745	1.35E-05	8	Rab18
0.839329691	0.181050543	1.36E-05	8	Zscan21
0.552374627	0.18309951	1.37E-05	8	Bmp2k
0.719150337	0.185503436	1.39E-05	8	Vipas39
0.512510028	0.190107832	1.42E-05	8	Prss30
0.868380376	0.191441142	1.43E-05	8	9430076C15Rik
0.412820736	0.19415386	1.45E-05	8	Mier1
0.503356646	0.194924271	1.46E-05	8	Smad3
0.664624349	0.198327282	1.49E-05	8	Fubp3
1.010262639	0.198383883	1.49E-05	8	Cd79b
0.421408911	0.198953118	1.49E-05	8	Nop14
0.417177133	0.201337143	1.51E-05	8	Casp6
0.749850895	0.202288695	1.52E-05	8	Las1l
0.733997127	0.20491613	1.53E-05	8	Ing4
0.625065211	0.208573226	1.56E-05	8	Cul1
0.844988627	0.209178166	1.57E-05	8	Syne1
0.345935477	0.213429387	1.60E-05	8	Shprh
0.433770315	0.214427629	1.61E-05	8	Atf7ip
0.456078132	0.215667685	1.62E-05	8	Alkbh4
0.576390663	0.216443478	1.62E-05	8	Preb
0.294475778	0.217777753	1.63E-05	8	Pdzd8
0.650775999	0.21895631	1.64E-05	8	Plcb4
0.548489667	0.21919782	1.64E-05	8	Stub1
0.929052018	0.21937537	1.64E-05	8	Snord104
0.326883801	0.224977769	1.68E-05	8	Scyl1
0.496670154	0.234274565	1.75E-05	8	Dars2
0.487030943	0.234478385	1.76E-05	8	Rock2
0.381118267	0.235613111	1.76E-05	8	Gmnn

0.505393352	0.236100564	1.77E-05	8	Pdap1
0.386411316	0.236782711	1.77E-05	8	Egr1
0.649528125	0.23828239	1.78E-05	8	Ist1
0.828061353	0.245093324	1.84E-05	8	Gstm5
0.661437479	0.245109061	1.84E-05	8	Irf9
0.43399759	0.247329635	1.85E-05	8	Plxna2
0.445994223	0.248637475	1.86E-05	8	Mtif3
0.280498633	0.252700416	1.89E-05	8	Ramp3
0.373717036	0.25598586	1.92E-05	8	Cdk5
0.424918685	0.257827221	1.93E-05	8	Ino80b
0.560037499	0.260082651	1.95E-05	8	Gskip
1.274770236	0.260870298	1.95E-05	8	Hdgfrp3
0.674049735	0.274484011	2.06E-05	8	Tbc1d20
0.431364481	0.275879803	2.07E-05	8	Mfap1a
0.588017581	0.288769345	2.16E-05	8	Snhg12
0.750160598	0.290153911	2.17E-05	8	N6amt2
0.386452404	0.294549496	2.21E-05	8	Tubb4b
0.463830682	0.295423519	2.21E-05	8	Sec24a
1.439827109	0.298948467	2.24E-05	8	C1ra
1.081700484	0.299782295	2.25E-05	8	Slc30a4
0.391807051	0.302774624	2.27E-05	8	Sdhc
0.586654587	0.303764935	2.28E-05	8	Med27
0.80503443	0.303857053	2.28E-05	8	Cspp1
0.399623527	0.31007246	2.32E-05	8	Nudt14
0.711463685	0.310765661	2.33E-05	8	Usp47
0.64545153	0.312850085	2.34E-05	8	Mepce
0.338471498	0.315603665	2.36E-05	8	Sik2
0.435534797	0.317387049	2.38E-05	8	Trim11
0.677637129	0.320058423	2.40E-05	8	Rnf14
0.538581305	0.320716157	2.40E-05	8	Zfp821
0.624586797	0.322749818	2.42E-05	8	Cbx1
0.47155522	0.323611334	2.42E-05	8	Samd4b
0.32515775	0.325759935	2.44E-05	8	Fam129a
0.490462727	0.32660614	2.45E-05	8	Ier3
0.878824229	0.327105732	2.45E-05	8	Trappc5
0.513600741	0.330154199	2.47E-05	8	Sep.02
0.726433853	0.337210775	2.53E-05	8	Nudt2
0.567001279	0.343182945	2.57E-05	8	Fem1c
0.792068524	0.344336134	2.58E-05	8	Fam135a
0.470182536	0.347985422	2.61E-05	8	Ap3m1
0.848269816	0.349415007	2.62E-05	8	Ssfa2
0.667942083	0.349714274	2.62E-05	8	Mrpl3
0.311798221	0.35361957	2.65E-05	8	Inpp4b
0.818050152	0.355238853	2.66E-05	8	Zfpl1
0.720744377	0.355366604	2.66E-05	8	Pmpca
0.351647327	0.35559044	2.66E-05	8	Yars2
0.608381206	0.357835812	2.68E-05	8	Btbd1
0.522832798	0.362209043	2.71E-05	8	Paip2
0.293911796	0.366632633	2.75E-05	8	Me2
0.39254405	0.37410227	2.80E-05	8	Metap1d
0.451439349	0.381071514	2.85E-05	8	Ccl7
0.45735167	0.387600642	2.90E-05	8	Eif2s3y
0.410790084	0.391973296	2.94E-05	8	Dhx57
0.507344607	0.396154731	2.97E-05	8	Ash1l
0.750294289	0.398181914	2.98E-05	8	Cenpv
1.0447274	0.398323849	2.98E-05	8	Sash3
0.463058216	0.399936762	3.00E-05	8	Ankrd12
0.403035716	0.404371415	3.03E-05	8	Capns1
0.667917793	0.404677842	3.03E-05	8	Fam46c
0.527391099	0.405205074	3.03E-05	8	Pdlim1
0.497302842	0.42425625	3.18E-05	8	Polr2i

-1.037102543	0.426325006	3.19E-05	8	Ctsc
1.10498595	0.435679446	3.26E-05	8	Tlcd2
0.686761817	0.439759615	3.29E-05	8	Ermp1
0.651179277	0.443720557	3.32E-05	8	Git1
0.886733886	0.448587658	3.36E-05	8	Jade1
0.371118979	0.456333386	3.42E-05	8	Ubr4
0.481842843	0.45813001	3.43E-05	8	Ccl5
-0.719885113	0.458625326	3.43E-05	8	Rps26
0.485590725	0.460407616	3.45E-05	8	Rpn1
0.751872301	0.464924365	3.48E-05	8	Snip1
0.419704486	0.465949165	3.49E-05	8	Wdr37
0.452140867	0.472882187	3.54E-05	8	Gm4285
1.013197864	0.474846964	3.56E-05	8	Gbp9
0.67537751	0.480721366	3.60E-05	8	Csf2rb2
1.268625893	0.486121304	3.64E-05	8	Herpud2
0.898449935	0.49050722	3.67E-05	8	Batf
-0.922686982	0.492273413	3.69E-05	8	Fcgr3
0.569929557	0.501212974	3.75E-05	8	Aebp2
0.555020831	0.504244463	3.78E-05	8	Pctp
0.677912372	0.506059488	3.79E-05	8	Timm17b
0.573984615	0.510936196	3.83E-05	8	Tns1
0.982053993	0.519610902	3.89E-05	8	Ltbp3
0.346938159	0.52248303	3.91E-05	8	Polr2j
0.555995296	0.528755882	3.96E-05	8	Acot7
0.456911413	0.533603353	4.00E-05	8	Uba6
0.451030226	0.534317016	4.00E-05	8	Smc1a
0.536676553	0.540504036	4.05E-05	8	Add1
0.468512859	0.542158462	4.06E-05	8	Copa
-0.367782508	0.550237538	4.12E-05	8	Ftl1
0.415525416	0.550261445	4.12E-05	8	Itga5
-0.44731603	0.558033016	4.18E-05	8	C1qb
1.204061571	0.561623764	4.21E-05	8	Cstf2
0.308498864	0.56518378	4.23E-05	8	Cog8
0.340521598	0.565859096	4.24E-05	8	Cep350
0.759939863	0.566571815	4.24E-05	8	D17Wsu104e
0.563985788	0.570371266	4.27E-05	8	Ctsw
0.330074297	0.572615305	4.29E-05	8	Surf1
0.278112621	0.581571623	4.36E-05	8	Memo1
0.679471234	0.58259051	4.36E-05	8	Rpl23
0.446233166	0.587506668	4.40E-05	8	Rnf169
0.623995035	0.600928871	4.50E-05	8	Met
0.529482571	0.601319554	4.50E-05	8	Peli2
0.668926088	0.612709942	4.59E-05	8	Polr2g
0.32545639	0.614241738	4.60E-05	8	Acvrl1
0.692501394	0.642411318	4.81E-05	8	Themis2
0.43154489	0.645061648	4.83E-05	8	Usp34
0.521233833	0.653797432	4.90E-05	8	Rexo1
0.379816572	0.654427353	4.90E-05	8	Ift20
0.523671221	0.660706085	4.95E-05	8	Tsc22d1
0.578499646	0.677283721	5.07E-05	8	Ap3m2
0.79329093	0.678504153	5.08E-05	8	Mesdc2
0.410808818	0.688229146	5.15E-05	8	Oraov1
0.32963382	0.689326358	5.16E-05	8	Tmem176a
0.526705378	0.691302328	5.18E-05	8	Dnajb4
0.689110848	0.701778832	5.26E-05	8	Mars
0.438801746	0.702619907	5.26E-05	8	E2f1
0.445062891	0.705171342	5.28E-05	8	Gart
0.484112215	0.706669717	5.29E-05	8	Fam76b
0.699583245	0.713696894	5.35E-05	8	Ift88
0.650156996	0.715927845	5.36E-05	8	Rnf219
0.271207235	0.726542309	5.44E-05	8	Jade2

0.528762819	0.741100192	5.55E-05	8	Elf2
0.397033187	0.743203471	5.57E-05	8	Tango2
0.49400262	0.744531756	5.58E-05	8	Cct2
0.323897729	0.751149212	5.63E-05	8	Wdr8
0.611330794	0.751895963	5.63E-05	8	Rcbtb1
0.446079968	0.753021659	5.64E-05	8	Stxbp3b
1.097655155	0.75641558	5.67E-05	8	Ddah2
0.66599661	0.75806717	5.68E-05	8	Smim8
0.35640751	0.76095404	5.70E-05	8	Katna1
0.292833229	0.768871846	5.76E-05	8	Kmt2c
0.458359719	0.769026993	5.76E-05	8	Cbx6
0.320061127	0.788326506	5.90E-05	8	Fam193a
1.043871063	0.788425933	5.90E-05	8	Rhoj
0.323188761	0.789240385	5.91E-05	8	Ghdc
0.289239777	0.78948932	5.91E-05	8	Tusc2
0.498417224	0.834805549	6.25E-05	8	Slco2b1
-0.436466541	0.837682509	6.27E-05	8	Cfl1
0.363925252	0.864288427	6.47E-05	8	Skil
0.434775263	0.869638824	6.51E-05	8	Cul5
0.421701689	0.870660951	6.52E-05	8	Nek7
0.346786169	0.871125829	6.52E-05	8	Snapc5
0.279191091	0.872403971	6.53E-05	8	Sdhaf2
0.475248009	0.878554003	6.58E-05	8	Exoc3
0.270721209	0.879954763	6.59E-05	8	Ppfibp2
0.558050413	0.887991908	6.65E-05	8	Flcn
0.40647416	0.894794453	6.70E-05	8	Ext2
-1.656262994	0.915026033	6.85E-05	8	Bicd2
0.65029237	0.921041948	6.90E-05	8	Tfpi
0.412459834	0.922339538	6.91E-05	8	Mfhas1
0.372176535	0.9372584	7.02E-05	8	Ampd3
0.314086594	0.938260887	7.03E-05	8	Msl1
0.849493251	0.948452073	7.10E-05	8	Rfx7
0.331713493	0.950572123	7.12E-05	8	Dhcr7
0.530413935	0.953673513	7.14E-05	8	Rabggtb
0.522521664	0.96476672	7.23E-05	8	Dph6
0.800200759	0.966141691	7.24E-05	8	Vps37b
0.743225276	0.967434707	7.25E-05	8	Vbp1
0.493142814	1	7.51E-05	8	Galk1
0.776077202	1	7.74E-05	8	Optn
0.42255565	1	7.82E-05	8	Ube2e3
0.501990837	1	7.98E-05	8	Anks3
0.34367156	1	8.04E-05	8	Slc48a1
0.397081742	1	8.09E-05	8	Swap70
0.587997636	1	8.11E-05	8	Yaf2
0.523257228	1	8.18E-05	8	Dennd4c
0.431340272	1	8.18E-05	8	Exoc1
0.279575485	1	8.25E-05	8	Srsf10
0.897887147	1	8.32E-05	8	Nomo1
0.74110092	1	8.44E-05	8	Ergic3
0.619462299	1	8.46E-05	8	Ephx1
0.554509339	1	8.47E-05	8	Paf1
0.507310682	1	8.49E-05	8	Erich1
0.446618026	1	8.52E-05	8	Eif2a
0.501264276	1	8.59E-05	8	Phf3
0.618720593	1	8.61E-05	8	Tmem11
0.287682072	1	8.64E-05	8	Ccnd3
0.47278951	1	8.71E-05	8	Ppp2r1b
0.459120732	1	8.71E-05	8	Caml
0.421000732	1	8.76E-05	8	Lyve1
-0.84704866	1	9.00E-05	8	Gm1821
0.784716666	1	9.08E-05	8	Akap7

0.478578036	1	9.31E-05	8	Syn1
0.537482516	1	9.31E-05	8	Cpd
0.541354612	1	9.40E-05	8	Arrb1
0.401829698	1	9.41E-05	8	Tmem147
0.518603345	1	9.46E-05	8	Mapre2
1.027791578	1	9.51E-05	8	Ap5s1
0.475296853	1	9.78E-05	8	Atg3
0.340616398	1	9.83E-05	8	Agap3
0.28128122	1	9.87E-05	8	Fam189b
0.285394346	1	9.92E-05	8	Pom121
0.483531411	1	0.0001011	8	Taf1b
0.44670115	1	0.0001013	8	Dyrk1a
0.300555275	1	0.0001016	8	P2rx7
0.554451471	1	0.000102	8	Mrpl40
0.442418221	1	0.0001023	8	Ints12
0.599556237	1	0.0001035	8	Slc16a3
0.371469324	1	0.00011	8	Asb2
-0.878650165	1	0.0001106	8	Mir6236
0.362216722	1	0.0001124	8	9130401M01Rik
0.399492754	1	0.0001126	8	Tapbp
0.3931798	1	0.0001133	8	Cic
0.402601875	1	0.0001148	8	Nr4a2
0.344679559	1	0.0001151	8	Aco2
2.154794966	1	0.0001159	8	Serpina3n
0.424880296	1	0.0001159	8	Ndufv1
0.520552712	1	0.0001167	8	Gm15708
0.340387063	1	0.0001168	8	Txndc11
0.307991955	1	0.0001174	8	A630001G21Rik
0.361576466	1	0.0001183	8	Zfp106
0.364598559	1	0.0001186	8	Pdlim7
0.544226098	1	0.0001193	8	Ttc4
0.445342781	1	0.0001203	8	Pten
0.53166433	1	0.0001234	8	Chd8
0.500813988	1	0.0001237	8	Guk1
0.532753248	1	0.0001244	8	Herpud1
0.576069115	1	0.0001245	8	Strada
0.744996989	1	0.0001246	8	Gnpat
0.522425046	1	0.0001249	8	Ncor1
0.46536325	1	0.0001253	8	Eci2
0.369944072	1	0.0001253	8	Rnf11
0.631550605	1	0.0001275	8	Fahd2a
0.409513497	1	0.0001281	8	Vps18
0.488352768	1	0.0001292	8	Dnajc25
-0.295046148	1	0.0001295	8	Fth1
0.467276394	1	0.0001299	8	Bet1l
0.322005535	1	0.0001305	8	Clcn4-2
0.405503908	1	0.0001305	8	Psmd4
0.415656114	1	0.0001313	8	Rbms1
0.614575648	1	0.0001319	8	Tmem62
0.543165352	1	0.0001335	8	Slc25a4
0.647413987	1	0.0001341	8	Tcf25
0.688580681	1	0.0001342	8	Arhgef2
0.278192359	1	0.0001345	8	Dnaja3
0.689033356	1	0.0001359	8	Igfbp4
0.501751819	1	0.000136	8	Ganab
0.661103011	1	0.0001363	8	Glipr1
0.417216793	1	0.0001368	8	Phb2
0.366642375	1	0.0001371	8	Rcan1
0.684788557	1	0.0001382	8	Dapk1
0.684559488	1	0.0001401	8	Slc38a1
0.261052647	1	0.0001409	8	Slc12a7

0.42863124	1	0.0001413	8	Uqcrc2
0.374270706	1	0.0001454	8	Zfp110
0.507315568	1	0.0001457	8	Eif4g3
0.659679533	1	0.0001468	8	Hmgxb3
0.338006182	1	0.0001481	8	Kdm3a
0.610389022	1	0.0001484	8	Zcwpw1
0.475625439	1	0.0001499	8	Arl3
0.465564558	1	0.0001516	8	Cct7
0.5150829	1	0.0001523	8	Rrp1
0.396387658	1	0.0001526	8	Timp2
0.484910004	1	0.0001537	8	Gm3219
0.277836149	1	0.0001545	8	Slc1a5
0.533334965	1	0.0001556	8	Gaa
0.512152989	1	0.0001581	8	Slc26a11
0.733905982	1	0.0001588	8	Wars
0.388161901	1	0.0001636	8	Ipo7
0.365533667	1	0.0001661	8	Mrpl30
0.528421386	1	0.00017	8	Qser1
0.275237467	1	0.0001705	8	Smim24
0.488268511	1	0.0001709	8	Cbfa2t2
0.284937428	1	0.0001755	8	Tbc1d9
-1.129992928	1	0.0001774	8	Camta2
0.545485966	1	0.0001783	8	Fopnl
0.40055679	1	0.0001807	8	Anxa6
0.499903575	1	0.0001836	8	Mettl9
0.434960799	1	0.0001838	8	Snrpd1
0.652550996	1	0.0001843	8	Gkap1
1.013823543	1	0.0001858	8	Lrch3
0.399602307	1	0.0001863	8	Trmt1
0.345193578	1	0.0001911	8	Pphln1
0.429173481	1	0.0001911	8	Mbtd1
0.373247961	1	0.0001934	8	Yeats2
0.457834473	1	0.0001976	8	Nme3
0.272055614	1	0.0001978	8	Gtf3c2
0.576230982	1	0.0002013	8	Mien1
0.354426207	1	0.0002037	8	Npepps
0.775724811	1	0.0002047	8	Eif1ad
0.265531832	1	0.0002065	8	Nr1h3
0.548490523	1	0.0002067	8	Rbck1
1.121508808	1	0.0002072	8	Slc35c2
0.361780964	1	0.000208	8	Atf2
0.423695459	1	0.000208	8	Ano6
0.327757959	1	0.0002084	8	Snrpa1
0.528277075	1	0.0002086	8	Ccnyl1
0.417732191	1	0.0002096	8	Acap2
0.463865571	1	0.0002112	8	1110038F14Rik
0.360002734	1	0.0002131	8	Iqsec2
0.827696893	1	0.0002144	8	Ict1
0.25882361	1	0.0002208	8	Zfp622
1.112797192	1	0.0002209	8	Taf2
0.306677415	1	0.0002214	8	Phospho2
0.444900961	1	0.0002215	8	Acaa2
0.582400295	1	0.000222	8	Yif1b
-0.325665718	1	0.0002228	8	Gpx1
0.496628999	1	0.0002228	8	Il1r2
1.924081889	1	0.0002238	8	Wbp1l
0.727590631	1	0.0002284	8	Grpel1
0.5286306	1	0.0002317	8	Cox20
0.414994213	1	0.0002324	8	2010012O05Rik
0.563965226	1	0.0002344	8	Pwwp2b
0.55033589	1	0.0002346	8	Gtf2h1

0.362211053	1	0.0002347	8	Mrpl15
0.514055762	1	0.0002366	8	Ints1
0.550208501	1	0.000241	8	Bcl2l11
0.298150682	1	0.0002413	8	Cant1
0.390802153	1	0.0002445	8	Klf4
0.550191696	1	0.0002458	8	Adamtsl5
0.300224852	1	0.0002459	8	Ankrd10
0.482786232	1	0.000246	8	Arhgap15
0.459069963	1	0.0002484	8	Insig2
0.624276716	1	0.0002489	8	Cep152
0.270293728	1	0.0002495	8	Mrs2
0.569942445	1	0.0002495	8	Phlpp1
0.39171414	1	0.00025	8	Spcs2
0.388030764	1	0.0002553	8	Chp1
0.520793055	1	0.0002587	8	Zranb3
0.642758688	1	0.0002587	8	Eng
0.68096444	1	0.0002611	8	Dcp2
0.617444921	1	0.0002631	8	Trappc8
0.371948805	1	0.0002632	8	Reps2
0.761763773	1	0.0002637	8	Siglec1
0.257804429	1	0.000264	8	Nup205
0.478266655	1	0.0002657	8	Tspan17
0.384781507	1	0.0002671	8	Ankle2
0.274636028	1	0.0002671	8	Trim44
0.427911414	1	0.0002692	8	Fbxw8
0.581269093	1	0.0002693	8	Oat
1.071190459	1	0.0002718	8	Khdrbs1
0.332662516	1	0.000273	8	Glod4
0.406536979	1	0.000273	8	Zranb2
0.291523508	1	0.0002738	8	Tmem214
0.430961823	1	0.0002748	8	Zfp296
0.37277479	1	0.0002793	8	Mlx
0.319663615	1	0.0002854	8	Parvb
0.256729823	1	0.0002863	8	Pdrg1
0.426418015	1	0.0002867	8	Ddb2
0.339704526	1	0.000289	8	Impad1
0.348628083	1	0.0002913	8	Lsm4
0.384222244	1	0.0002923	8	Cstf3
0.351592256	1	0.0002924	8	Zcchc9
0.350229222	1	0.0002927	8	Galnt2
0.48255565	1	0.0002946	8	B9d2
0.341741012	1	0.0002969	8	B4galt1
0.570490366	1	0.0002976	8	Srsf11
0.440155483	1	0.0003007	8	Dda1
0.657631014	1	0.0003009	8	Tppp3
0.851654941	1	0.000306	8	Pus3
0.467970813	1	0.0003062	8	Ctbp2
0.25673901	1	0.0003091	8	Mrps7
0.484977925	1	0.00031	8	Pign
0.375707604	1	0.0003117	8	Rexo2
0.384458844	1	0.000312	8	Eif4h
0.374785374	1	0.0003127	8	Raf1
0.978114729	1	0.0003174	8	Rnpc3
-0.602341737	1	0.0003211	8	H2-K1
0.647369399	1	0.0003213	8	Klf9
0.261706586	1	0.0003226	8	Fyn
0.587652827	1	0.0003228	8	Srd5a3
0.364719724	1	0.0003331	8	Nt5m
0.85429119	1	0.000335	8	Mrpl36
0.357732585	1	0.000338	8	Tmppe
1.668199044	1	0.0003395	8	Spsb1

0.478712387	1	0.0003402	8	Tnfrsf1a
0.95960185	1	0.0003405	8	Trim39
0.534894334	1	0.0003417	8	Mknk2
0.614114851	1	0.0003424	8	Agfg1
0.344573597	1	0.0003442	8	Cyld
-1.644561309	1	0.0003486	8	Bcl10
0.540144849	1	0.00035	8	Xrn2
0.325286297	1	0.0003528	8	Comtd1
0.260435545	1	0.0003541	8	Nadk
0.533845352	1	0.0003544	8	Atxn10
-1.607066981	1	0.0003559	8	Plxnc1
-0.486293511	1	0.0003567	8	Lyz2
0.307537446	1	0.0003571	8	Mrpl12
0.305700578	1	0.0003579	8	Suv420h2
0.851250846	1	0.0003583	8	Tomm20
0.576409897	1	0.0003608	8	Tmem60
0.335765267	1	0.0003662	8	Parp1
0.660456372	1	0.0003678	8	Ttc1
0.469712974	1	0.0003689	8	Wbp1
-1.579424983	1	0.0003691	8	Msr1
0.322262406	1	0.0003708	8	Fcgrt
0.392244043	1	0.0003721	8	Map3k1
0.28488939	1	0.0003738	8	Eif2ak3
0.523957414	1	0.0003817	8	Ube2z
0.343883352	1	0.0003829	8	Hbp1
0.250124878	1	0.0003832	8	Stx2
0.396090775	1	0.0003833	8	Ndufa9
0.735177937	1	0.0003843	8	Psmg2
0.342404866	1	0.0003902	8	Pja2
0.33217914	1	0.0003941	8	Mesdc1
0.548312333	1	0.0003972	8	Gramd1b
0.272746289	1	0.0003974	8	Cep120
-1.766071474	1	0.0003977	8	Kat7
0.559823451	1	0.0003999	8	Notch2
0.291388722	1	0.000404	8	Ap5z1
0.356748722	1	0.0004086	8	Polr2f
0.437253291	1	0.0004117	8	Rab11fip1
0.285448433	1	0.0004172	8	Abcd3
0.311420838	1	0.0004185	8	Cntrl
1.813335794	1	0.0004191	8	Heatr5a
0.305058704	1	0.0004226	8	Atad5
0.420571246	1	0.0004227	8	Spred1
0.664491925	1	0.000424	8	Aktip
0.558153134	1	0.000425	8	Bcap29
0.48143372	1	0.0004265	8	Tmem2
0.581222781	1	0.0004282	8	Txndc15
0.260100124	1	0.0004295	8	Fkbp3
0.393596451	1	0.0004374	8	Pold3
0.328401544	1	0.0004389	8	Hint2
0.315543235	1	0.0004409	8	Sgms1
0.461710816	1	0.000442	8	Bcl2l1
0.274180444	1	0.0004434	8	Arhgap35
0.394127033	1	0.0004444	8	Pabpn1
0.337898927	1	0.00045	8	Plk2
0.253530873	1	0.0004504	8	Eif2b4
0.58055602	1	0.0004519	8	Bfar
0.444290551	1	0.0004553	8	Wdr4
0.522695713	1	0.0004595	8	Psmc2
0.545269801	1	0.0004603	8	Dpm1
0.602621818	1	0.0004662	8	Prr24
0.263192822	1	0.0004692	8	Pnp

0.408301521	1	0.0004713	8	Atp5j
0.604108928	1	0.000475	8	Cast
0.43027193	1	0.0004784	8	Thbs1
0.392304581	1	0.000489	8	Eif4a3
0.288512982	1	0.0004926	8	Ugp2
0.480744089	1	0.0004931	8	Lrrc59
0.263388123	1	0.0004964	8	Cyp27a1
0.36776584	1	0.0004974	8	Vps25
0.309929147	1	0.0004979	8	Laptm4a
0.538574852	1	0.000498	8	Map2k7
0.549507926	1	0.0005054	8	Serf1
0.330374586	1	0.0005145	8	Carhsp1
0.363412559	1	0.0005187	8	Mafg
0.402467835	1	0.0005203	8	Dhrs4
0.32177233	1	0.0005224	8	Rangap1
0.306405548	1	0.0005297	8	Arid4b
0.484401643	1	0.0005298	8	Mgat1
0.341693122	1	0.0005324	8	Rab43
0.572624238	1	0.0005341	8	Zmiz2
0.34385078	1	0.0005431	8	Grcc10
0.39707386	1	0.0005727	8	Zfp472
0.392805312	1	0.0005742	8	Mios
0.478584322	1	0.0005784	8	Mocs1
0.386247756	1	0.0005874	8	Otud1
0.482745968	1	0.0005903	8	Mrpl43
0.350587735	1	0.000598	8	Rala
-0.759989535	1	0.0005987	8	Rps19
0.715059486	1	0.0006045	8	Crk
0.484438419	1	0.0006077	8	Pbrm1
0.295407272	1	0.0006081	8	Gnl3
0.619800071	1	0.0006108	8	Rb1
0.2686259	1	0.0006136	8	Rc3h1
0.455767563	1	0.0006159	8	Vps36
0.35282269	1	0.0006264	8	Spen
0.441118265	1	0.0006273	8	Elovl1
0.343619813	1	0.0006316	8	Prpf39
0.573222052	1	0.0006384	8	Atp5d
0.718602606	1	0.0006394	8	Dedd2
0.795455538	1	0.0006426	8	Parp3
0.396035762	1	0.0006498	8	Ppapdc1b
0.519813936	1	0.0006543	8	Ppp1r10
0.371362696	1	0.0006554	8	Tbxas1
0.422548357	1	0.0006563	8	Pde4b
0.387370362	1	0.0006593	8	Stag2
0.685091412	1	0.000664	8	Tiparp
-1.554895772	1	0.0006818	8	Eif4e
0.32119781	1	0.0006838	8	Ids
0.406215577	1	0.0006949	8	Icosl
0.522886339	1	0.0007016	8	Asph
0.45009009	1	0.0007029	8	Sergef
0.633086007	1	0.0007035	8	Spg20
0.461134914	1	0.0007056	8	Mtm1
-1.430383842	1	0.0007066	8	Spast
0.365236185	1	0.0007195	8	Tmco4
0.691430895	1	0.0007222	8	Ppan
0.429115459	1	0.0007291	8	Sgsh
0.332676809	1	0.0007369	8	Vamp4
0.346292798	1	0.0007371	8	Qars
0.398041048	1	0.0007442	8	Zyx
0.763449918	1	0.0007447	8	Cdk19
0.737870524	1	0.0007461	8	Cnst

0.606703462	1	0.0007503	8	Dap3
0.487592131	1	0.0007554	8	Uspl1
0.429682655	1	0.0007668	8	Pcnt
0.502342253	1	0.0007729	8	Pnpo
0.47429188	1	0.0007735	8	Zfp53
0.47563596	1	0.0007738	8	Fryl
0.273601299	1	0.0007739	8	Fam65b
0.468353177	1	0.0007821	8	Uggt1
-1.518434189	1	0.0007862	8	Map2k1
-1.427086757	1	0.0007967	8	Pla2g15
0.319240443	1	0.0007974	8	Mrpl47
0.466872684	1	0.0008071	8	Mcmbp
0.266373741	1	0.0008075	8	NP-904338.1
0.660672002	1	0.0008109	8	Hemk1
0.287562204	1	0.0008111	8	Rbmx
0.556557761	1	0.0008143	8	C2cd2l
0.622518709	1	0.0008213	8	Il6st
0.298729236	1	0.000853	8	Cep250
0.311641233	1	0.0008545	8	Cox16
0.285237083	1	0.0008622	8	Prdm1
0.374706109	1	0.0008634	8	Rab2a
0.469882151	1	0.0008786	8	Mak16
0.328504067	1	0.000892	8	Sp140
0.358280081	1	0.0009018	8	Ndufa8
0.562719547	1	0.0009109	8	Arl14ep
0.499880755	1	0.0009109	8	Hibch
0.722567472	1	0.0009132	8	Rab3d
0.254387826	1	0.0009186	8	Chst12
0.270724697	1	0.0009226	8	Anp32a
0.876668359	1	0.0009228	8	Mus81
0.524624714	1	0.0009282	8	Birc5
0.291443985	1	0.0009299	8	Dnm1l
0.319697002	1	0.0009306	8	Casp2
0.543374566	1	0.0009334	8	Dhps
0.578169196	1	0.0009403	8	Snx20
0.262323641	1	0.0009415	8	Ctnnb1
-1.568429304	1	0.000942	8	Ythdf3
0.382268658	1	0.0009448	8	Suco
0.250757727	1	0.0009453	8	Tmbim1
0.513271901	1	0.0009477	8	Psmf1
0.511213296	1	0.0009482	8	Vkorc1
0.256910414	1	0.0009502	8	Dlst
0.374043149	1	0.0009656	8	Tmem140
0.255203521	1	0.0009706	8	Sema4d
0.270925148	1	0.0009895	8	Rab11fip5
0.254452267	1	0.0009964	8	Brd2
0.577692435	1	0.0009995	8	Jmy
0.389006256	1	0.0010001	8	Ttc3
0.556770238	1	0.001005	8	Nme6
0.463602464	1	0.0010103	8	Smarca2
0.792097699	1	0.0010156	8	Tubg1
0.622633578	1	0.0010271	8	Diap2
0.383599865	1	0.001032	8	Snrk
0.851926275	1	0.0010388	8	Usp33
0.462933115	1	0.0010402	8	Gpatch4
0.831045864	1	0.0010451	8	Serpinb6b
0.353294515	1	0.0010511	8	Mettl16
0.439034923	1	0.0010566	8	Rftn1
0.505405556	1	0.0010753	8	Mtmr1
0.336049861	1	0.0010772	8	Cxcl1
0.486416659	1	0.0010866	8	Trap1

0.955413811	1	0.0010983	8	Smim11
0.313700133	1	0.0011	8	Utp6
0.348530333	1	0.0011025	8	Pik3c2a
0.31121257	1	0.0011071	8	Tfip11
0.257554062	1	0.0011141	8	Afg3l2
0.415476771	1	0.0011345	8	Ibtk
0.341861828	1	0.0011417	8	Gnptg
0.36317557	1	0.0011431	8	Aqr
0.343891357	1	0.0011475	8	Rab8a
0.371837192	1	0.0011479	8	Stag1
0.348760434	1	0.0011517	8	Tm7sf3
0.411165761	1	0.0011577	8	Carm1
0.30329469	1	0.0011593	8	Taf1d
0.261924769	1	0.001181	8	1500012F01Rik
0.524814551	1	0.0011844	8	Csrp1
0.379841076	1	0.0011844	8	Zfp52
-1.553616417	1	0.0011905	8	Dirc2
0.276793067	1	0.0011985	8	Anxa2
0.257529034	1	0.0011991	8	Manf
-0.389985506	1	0.0011996	8	Cyba
0.254273069	1	0.0012065	8	Limd1
0.339300152	1	0.0012077	8	Nat9
0.362009463	1	0.0012148	8	Dapp1
0.360310663	1	0.0012154	8	Ndufv2
0.329620364	1	0.0012162	8	Wbp2
0.43699949	1	0.0012483	8	Morc3
-0.519803388	1	0.001274	8	Lars2
0.480362616	1	0.0012773	8	Plau
0.468447712	1	0.0012911	8	Mpp6
0.429865136	1	0.0013067	8	Kdm6a
0.481678661	1	0.0013077	8	Anxa7
0.340919074	1	0.0013234	8	Itch
0.700020854	1	0.0013424	8	Slx4
0.364573249	1	0.0013674	8	Atp8a1
0.333721972	1	0.0013824	8	Eif3d
0.405192091	1	0.0014032	8	Kpna3
0.284515843	1	0.001409	8	Mbd6
0.528155481	1	0.0014153	8	Lrrk2
0.378134882	1	0.0014161	8	Fam172a
0.552374627	1	0.0014186	8	Ddx41
0.278112621	1	0.0014294	8	Dus3l
0.444143902	1	0.0014361	8	Stap1
0.335224845	1	0.001452	8	Erlin2
0.411077483	1	0.0014563	8	Mkl1
0.906256273	1	0.0014681	8	Gpaa1
0.40097498	1	0.0014888	8	Glrx5
0.359652328	1	0.0014982	8	Nupr1
0.452784467	1	0.0015018	8	Tysnd1
0.733340251	1	0.0015045	8	Thbd
0.418197781	1	0.0015359	8	Cdk2
0.343678371	1	0.0015427	8	Smox
0.386516953	1	0.0015499	8	Tmx4
0.282498985	1	0.0015633	8	Park7
0.352754476	1	0.0015841	8	Surf2
0.253809232	1	0.0015907	8	Khsrp
0.48503275	1	0.0015972	8	Smad7
0.605305584	1	0.0016089	8	Ccdc23
-0.629137061	1	0.001615	8	Rps15a
0.83967319	1	0.0016197	8	Lipo1
0.360747692	1	0.001632	8	Stt3a
0.610286141	1	0.0016782	8	Prkar1a

0.264733579	1	0.001683	8	Nucb2
0.372349282	1	0.0016982	8	Polr2m
0.359886462	1	0.001701	8	Lsm6
0.298950396	1	0.0017058	8	0610010F05Rik
-1.471177749	1	0.0017341	8	Clec4n
-0.330526016	1	0.0017342	8	Tyrobp
0.554507582	1	0.0017763	8	Pcyox1
0.262354039	1	0.0017816	8	Asxl1
0.638373453	1	0.0017884	8	Ado
0.327140097	1	0.0017903	8	Bud31
-1.296763235	1	0.001795	8	Mgat2
0.566763364	1	0.0018252	8	Gatsl3
0.508531137	1	0.0018331	8	Ube3c
0.301135582	1	0.0018412	8	Anapc16
0.617141545	1	0.0018571	8	Gm15421
0.357732585	1	0.001858	8	Donson
0.267926082	1	0.0018594	8	Stom
0.435957525	1	0.0018623	8	Tbk1
0.507495651	1	0.0018777	8	Mt2
0.400629435	1	0.0018836	8	Swi5
-0.278840455	1	0.001895	8	Calm1
0.535963544	1	0.0018955	8	Map2k3
0.508518634	1	0.0019236	8	Ap1g1
-0.440421358	1	0.0019574	8	Arpc5
0.25485328	1	0.0019579	8	Riok1
0.784885928	1	0.0019699	8	Tmcc3
0.280791973	1	0.0019866	8	Lmo4
0.655848117	1	0.001988	8	Tmem237
0.540197647	1	0.0019981	8	Cr1l
0.26145106	1	0.0020473	8	Fxyd2
0.29824753	1	0.0020636	8	Snx13
0.372799222	1	0.0020766	8	Rac2
0.450516742	1	0.0020865	8	Sqrdl
0.461236915	1	0.0020964	8	Mphosph6
0.492195737	1	0.0021444	8	Procr
0.362354099	1	0.0021465	8	Ndufb10
0.259026817	1	0.0021607	8	Eps8
0.284296338	1	0.0022095	8	Pde1b
0.457834983	1	0.0022146	8	Pbxip1
0.713039988	1	0.0022476	8	Mlec
0.688872365	1	0.0022639	8	Cdc42bpg
0.331506389	1	0.0023091	8	Irgq
0.615227596	1	0.0023129	8	Higd1a
1.084609776	1	0.002313	8	Pofut1
0.756403445	1	0.0023263	8	Abhd14a
0.307681077	1	0.0023315	8	Tbc1d10b
0.417408857	1	0.0023436	8	Bank1
0.316666137	1	0.0023606	8	Kansl3
0.288597045	1	0.0023885	8	Gatc
0.401837973	1	0.0023984	8	Sgcb
0.491121074	1	0.0024174	8	Tesk1
0.407510632	1	0.0024229	8	Mad2l2
0.257575086	1	0.0024427	8	Nin
-0.500720178	1	0.0024453	8	C1qa
0.456879636	1	0.0024801	8	Phf20l1
0.299113772	1	0.0025023	8	Sec62
0.458523946	1	0.0025124	8	Zfp623
-1.442048421	1	0.0025145	8	Phf20
0.38411795	1	0.0025235	8	Aplp2
0.330642089	1	0.0025359	8	Cyfip2
0.671393604	1	0.0025499	8	Glcci1

-1.448286037	1	0.0025881	8	Cacul1
0.32759249	1	0.0025924	8	Mrpl35
0.594746041	1	0.0026158	8	Adamts10
0.267942873	1	0.0026173	8	Serpinb6a
-0.479888841	1	0.0026228	8	Cybb
0.546332312	1	0.0026397	8	Dgkd
0.325747709	1	0.0026695	8	Cog1
1.092995823	1	0.0026939	8	Tecr
-1.158398114	1	0.0027157	8	Fcho2
0.681278524	1	0.0027222	8	Samm50
0.26331987	1	0.0027255	8	Polk
0.269381233	1	0.0027294	8	2410006H16Rik
0.257598391	1	0.0027482	8	Stk24
0.363850911	1	0.002774	8	Mrpl14
0.258172603	1	0.0027786	8	Cops5
0.834332253	1	0.0028386	8	Per1
0.373344351	1	0.0028646	8	Cds2
0.359910826	1	0.0029068	8	Zfp217
0.536972782	1	0.0029187	8	Ttc12
0.333039973	1	0.0029197	8	Esco1
0.255087523	1	0.0029396	8	Dph5
0.396370378	1	0.00297	8	Rpl22l1
0.258766094	1	0.0029759	8	Tbrg1
0.455154344	1	0.0029782	8	Tuba1a
0.534059781	1	0.0030032	8	Pitpnb
0.283393372	1	0.00301	8	Ldha
0.294179437	1	0.0030315	8	Helb
0.390383844	1	0.0030482	8	Btbd2
0.745323276	1	0.0030578	8	Txndc5
0.30553969	1	0.0030809	8	Fzr1
0.365739497	1	0.0031405	8	Ptms
0.490194321	1	0.0031532	8	Hps5
0.33443942	1	0.0031954	8	Zfp667
0.358281343	1	0.0032049	8	Emc10
0.695404116	1	0.0032342	8	Ankrd27
0.497977481	1	0.0032421	8	Mtg2
0.869408911	1	0.0032644	8	Lypla1
-1.328378381	1	0.0032729	8	Tssc1
-1.341222866	1	0.0032732	8	Cat
0.45539567	1	0.0033572	8	Vimp
0.263070494	1	0.003404	8	Akt3
0.345767798	1	0.0034362	8	Hgsnat
0.852479219	1	0.0034402	8	Rbbp5
-1.358444203	1	0.0034848	8	Slc27a1
0.870303286	1	0.0034893	8	Urgcp
-1.441621709	1	0.0035013	8	Usp3
0.361714725	1	0.0035454	8	Coa6
0.515951006	1	0.0035485	8	Eif2d
0.478757881	1	0.0035494	8	Btbd10
0.459895768	1	0.0035696	8	Tatdn2
0.372289895	1	0.0035705	8	Ccdc86
0.301763681	1	0.003601	8	Cers5
0.580508921	1	0.0036546	8	Chmp4b
0.349835006	1	0.0036864	8	Snap29
0.602403418	1	0.0036888	8	Il4ra
0.380061443	1	0.0036992	8	Ssb
0.452659153	1	0.0037024	8	Cct6a
-1.276943944	1	0.0037052	8	Fam105a
0.341295826	1	0.0037271	8	Bmpr2
0.447363823	1	0.0037671	8	Usp2
0.300861379	1	0.0038156	8	Pdcd11

0.37306849	1	0.0038651	8	Traf5
0.605912215	1	0.0038654	8	Igf1r
0.374342215	1	0.0039139	8	Irf1
-0.639771218	1	0.0039591	8	Dazap2
0.273953358	1	0.0039626	8	Irf3
0.307290227	1	0.0039813	8	S100a13
-1.028561776	1	0.0039989	8	Imp4
-0.886333477	1	0.0040049	8	Cebpb
0.334596832	1	0.0040272	8	Nfkbid
0.414296879	1	0.004058	8	Clp1
-1.41723859	1	0.004058	8	Cd38
0.287236052	1	0.0040953	8	Ywhae
-0.995991679	1	0.0040982	8	0610012G03Rik
0.606338359	1	0.0041165	8	Prrc2a
-0.25890384	1	0.004127	8	Inpp5k
0.414672754	1	0.0041837	8	Pelp1
0.378475888	1	0.004213	8	Mllt1
-1.410409813	1	0.0042157	8	Prosc
-1.113838162	1	0.0042199	8	Rbm22
-1.259473907	1	0.0042511	8	Cdc42ep4
0.867158629	1	0.0042946	8	Cd22
0.5999349	1	0.0043107	8	Clpp
0.298759109	1	0.0043147	8	Tmem59
0.352715521	1	0.0043177	8	Cinp
0.269747452	1	0.0043191	8	Abhd17a
0.499293442	1	0.0043212	8	Itgal
-1.416993115	1	0.0043489	8	Ahcyl2
0.308170151	1	0.004353	8	Eid1
0.297818704	1	0.0043571	8	Krr1
0.277106957	1	0.0044106	8	Tipin
0.58426298	1	0.0045152	8	Pdlim2
-1.178274781	1	0.0045891	8	Trafd1
0.30624553	1	0.0047004	8	Wdr33
0.455224603	1	0.0047039	8	2810004N23Rik
0.527290803	1	0.0047098	8	Mrps10
-1.262179708	1	0.004713	8	Sowahc
0.458161355	1	0.0047392	8	Mylk
-0.609828765	1	0.0047507	8	Rpl18
-1.230558123	1	0.0047647	8	Tmem189
-0.267101905	1	0.0047694	8	Ptger4
0.903575226	1	0.00478	8	Sh3bp2
0.382577056	1	0.0047926	8	Phxr4
0.437043324	1	0.0048047	8	Hps4
-1.357889119	1	0.0048584	8	Smad1
0.25838743	1	0.0049061	8	Rpl7
-0.738529166	1	0.004936	8	Sat1
-0.405772009	1	0.0049499	8	Mir682
-1.688442514	1	0.0050005	8	Pfdn2
-1.307053257	1	0.0050086	8	Mybbp1a
0.396764933	1	0.00511	8	Zfp704
0.459295553	1	0.0051407	8	Scmh1
0.294094434	1	0.0051961	8	Cox7a2
0.500130852	1	0.0052668	8	Mat2b
0.402695726	1	0.0052843	8	Ndufs4
-1.54484125	1	0.0052955	8	Tbc1d1
0.381650813	1	0.0052987	8	Coa5
0.313349946	1	0.0053013	8	Dnajb1
0.300413943	1	0.0053143	8	Rcn2
-1.610649802	1	0.0053262	8	Dok2
0.772189484	1	0.0053409	8	Cp
2.154119734	1	0.0054415	8	Smarcd2

-1.104830801	1	0.005442	8	Irf2bp2
0.275614279	1	0.0054452	8	Appl1
-0.463561976	1	0.0054708	8	Wfdc17
0.434753519	1	0.0054807	8	Ppt1
1.012179439	1	0.0054982	8	Xpo6
-0.783675923	1	0.0055072	8	Atp5j2
-0.933453188	1	0.005512	8	Dnaja1
0.423957484	1	0.0055477	8	Dpf2
0.511552632	1	0.0055909	8	Agpat6
0.280930397	1	0.005627	8	Ptpro
-0.559110099	1	0.0056448	8	Tpd52
-0.763152062	1	0.0056555	8	Cyth4
0.39034989	1	0.005668	8	Nrm
0.277728881	1	0.0056766	8	Snrpe
0.367332905	1	0.0056845	8	Wdtc1
0.451285321	1	0.0057231	8	Elf1
-0.656002924	1	0.0057389	8	Unc93b1
1.098191545	1	0.005765	8	1500011K16Rik
0.294863432	1	0.0060988	8	Hook2
-1.358129417	1	0.0061107	8	Atg13
0.270702312	1	0.0061263	8	Myo1e
0.566402173	1	0.0061386	8	Mllt4
0.476688399	1	0.0061548	8	Scaper
0.517631678	1	0.006165	8	Pim1
0.465943544	1	0.0062224	8	Rbm39
-1.026570606	1	0.0063388	8	Sarnp
0.260293402	1	0.0063868	8	H13
0.298187459	1	0.0064045	8	Rbm15
-1.088165468	1	0.0066284	8	Lhfpl2
0.303988458	1	0.0066606	8	Igsf8
-1.315624123	1	0.0066709	8	Gtf2f2
-1.247652769	1	0.0067055	8	Cxcr4
0.256199985	1	0.0067313	8	Pilra
0.254901398	1	0.0067408	8	Nr4a1
0.265921811	1	0.0067821	8	Tmco3
-1.360948601	1	0.0067854	8	Kdm1a
0.259122579	1	0.0067937	8	Traf3
0.289734361	1	0.0069459	8	Dad1
0.378416575	1	0.0070004	8	Nqo2
0.388041632	1	0.0070318	8	Brd3
0.327194069	1	0.007076	8	Ndufs7
-1.272028027	1	0.0071252	8	Gng2
-1.511318558	1	0.0071257	8	Gramd3
-1.43512942	1	0.0071523	8	Abcc1
0.280226627	1	0.0071829	8	Cep83
0.250493533	1	0.0072613	8	Jmjd6
0.390247188	1	0.0072681	8	Rab3gap1
0.349506069	1	0.0072731	8	Mocs2
0.490309518	1	0.0073834	8	0610037L13Rik
0.45739842	1	0.0073842	8	Slc6a8
-0.31666322	1	0.0073868	8	Brix1
-0.448235691	1	0.0073887	8	Mir692-1
-1.27895419	1	0.0074681	8	Psen2
0.616403507	1	0.0074697	8	Rpl3
0.556736155	1	0.0075125	8	Bambi
-0.44899547	1	0.0075721	8	Sdcbp
0.629155254	1	0.0076065	8	Tiam1
0.430460378	1	0.007614	8	Morf4l1
-0.551337493	1	0.0076372	8	Arpc4
0.464784713	1	0.0076389	8	Cox10
0.301643119	1	0.0076477	8	Bbx

0.419704486	1	0.0077005	8	Mcm2
-1.242221724	1	0.0077154	8	Cldn25
-1.370889627	1	0.0077972	8	Cherp
0.298762349	1	0.0078552	8	Ddx21
1.038242921	1	0.0078618	8	Cfb
-0.602609563	1	0.0078671	8	Gm6251
0.376606157	1	0.0080041	8	4932438A13Rik
0.259270281	1	0.0080303	8	Myeov2
-0.579427298	1	0.0080482	8	Rps18
0.255893	1	0.0081032	8	Ubxn6
-1.319298042	1	0.0081208	8	Pou2f2
-1.198356684	1	0.0081741	8	Pofut2
-1.329981738	1	0.0081822	8	Cyb561d2
0.257510133	1	0.0082951	8	Huwe1
0.280842216	1	0.0083561	8	Rbbp8
-0.310022029	1	0.0083662	8	Pkp4
0.420196964	1	0.0083998	8	Tbcd
-1.206070892	1	0.0084105	8	Fabp5
-0.554884574	1	0.0084554	8	Actr2
-1.186959045	1	0.0085234	8	Kat6a
0.352319559	1	0.0085407	8	Rev3l
0.259287607	1	0.0085545	8	Abr
-1.431756735	1	0.0085681	8	Pet100
0.251382948	1	0.0085872	8	Ifi27
0.441348373	1	0.0086441	8	Taldo1
0.663606339	1	0.0087646	8	Atxn7l3b
0.582745725	1	0.0088038	8	Zfp236
0.255411745	1	0.0088235	8	Ppm1a
-0.830137552	1	0.0088843	8	Tgif1
0.311941699	1	0.0089352	8	Mpv17
0.409126692	1	0.0089927	8	Aptx
0.505159886	1	0.0090649	8	Tacc3
-1.04015344	1	0.009135	8	Thoc6
0.447210055	1	0.0091594	8	9030617O03Rik
0.2934377	1	0.0092329	8	Dcaf11
-0.343401682	1	0.0092941	8	C1qc
1.040271118	1	0.0093727	8	Rbbp6
0.27418589	1	0.0094087	8	B3gnt1
-1.14808447	1	0.0094328	8	Slc15a4
-0.266193379	1	0.0094481	8	Slc30a9
-1.316692169	1	0.0094576	8	Srp72
0.279313823	1	0.009601	8	Nt5dc3
0.325033125	1	0.0096554	8	Vps35
0.839032484	1	0.0097059	8	Prnp
0.331831857	1	0.0098365	8	Akap10
0.397497664	1	0.0098573	8	Hiat1
0.690231226	1	0.0098745	8	Baz2b
-1.088837479	1	0.0099105	8	Cpt1a
-1.090377743	1	0.0099592	8	Tra2b
1.708736591	5.00E-104	3.74E-108	9	Smyd1
1.431223517	4.07E-100	3.05E-104	9	E230025N22Rik
1.355748808	1.18E-99	8.87E-104	9	Klk1
2.074949266	1.08E-98	8.12E-103	9	B4galt2
2.088530546	2.13E-91	1.60E-95	9	Nrxn2
1.473657479	7.23E-83	5.41E-87	9	Klf8
1.871945948	9.07E-81	6.79E-85	9	Gm1673
1.7401716	9.98E-80	7.48E-84	9	AI464131
1.761123648	1.80E-77	1.35E-81	9	Rad54b
1.308448679	1.97E-75	1.48E-79	9	Crybb3
1.374680792	1.32E-73	9.89E-78	9	Spaca1
2.19266843	1.97E-68	1.47E-72	9	Map6

1.419729137	1.64E-67	1.23E-71	9	Havcr1
1.401295436	3.26E-64	2.44E-68	9	Bahcc1
1.368296964	6.55E-62	4.91E-66	9	Polq
2.364100334	8.69E-62	6.51E-66	9	Ccne2
1.481261429	9.13E-62	6.84E-66	9	Esco2
2.94636572	2.13E-59	1.59E-63	9	Tcf19
1.732122037	3.74E-56	2.80E-60	9	Rad51ap1
0.993884867	3.07E-55	2.30E-59	9	Lipn
1.010406744	1.05E-53	7.87E-58	9	Casq1
1.588805467	1.69E-53	1.26E-57	9	Pole
1.587258677	3.66E-53	2.74E-57	9	Ccne1
2.374029195	1.20E-52	9.00E-57	9	Gdpd5
3.083234271	4.53E-52	3.39E-56	9	Gins1
1.376604711	8.77E-52	6.57E-56	9	Mcm8
1.39579901	2.07E-51	1.55E-55	9	Calhm2
1.897764938	4.83E-51	3.62E-55	9	Telo2
1.531962532	2.76E-50	2.06E-54	9	Foxd2os
0.8017515	1.03E-49	7.71E-54	9	Bpifa6
0.998923364	2.59E-49	1.94E-53	9	Lrp11
1.320015604	6.54E-49	4.90E-53	9	Gtse1
1.359205073	1.21E-47	9.03E-52	9	Trmt44
1.236530772	3.33E-47	2.49E-51	9	Npnt
2.156129691	4.45E-47	3.33E-51	9	Rnf121
1.838003204	4.90E-47	3.67E-51	9	D6Ertd527e
1.220878278	1.25E-46	9.36E-51	9	Fads2
1.962263188	4.46E-45	3.34E-49	9	Chaf1a
1.644476538	5.20E-45	3.89E-49	9	C78339
1.717767684	5.84E-45	4.37E-49	9	Bend6
1.705222683	4.86E-44	3.64E-48	9	Spib
1.720051145	1.07E-43	7.98E-48	9	Cyp20a1
1.283498688	1.13E-43	8.46E-48	9	Tonsl
1.647562814	6.85E-43	5.13E-47	9	1110051M20Rik
1.410753149	1.91E-42	1.43E-46	9	A430033K04Rik
1.928032692	6.05E-42	4.53E-46	9	Gm16039
1.927450133	7.13E-42	5.34E-46	9	Prss46
2.166761406	7.14E-42	5.35E-46	9	Col4a1
1.465649408	7.38E-42	5.52E-46	9	Hmga2
0.763623228	3.87E-41	2.90E-45	9	Gucy1b3
1.308503502	6.48E-41	4.86E-45	9	Gemin6
1.88186903	1.32E-40	9.90E-45	9	Athl1
1.303254146	2.03E-40	1.52E-44	9	Chtf18
1.636353288	5.09E-40	3.81E-44	9	Lipt2
1.557763014	5.09E-40	3.81E-44	9	Gm20748
1.900477306	9.70E-40	7.27E-44	9	Csrnp2
1.729261594	1.94E-39	1.45E-43	9	Fam219a
1.297730939	2.98E-39	2.23E-43	9	2200002D01Rik
1.646989131	5.09E-39	3.82E-43	9	Tmem107
1.42671551	1.97E-38	1.47E-42	9	1110008L16Rik
2.506614213	2.36E-38	1.77E-42	9	Sep.03
1.51363786	1.14E-37	8.51E-42	9	Dgat2
1.996804147	1.24E-37	9.29E-42	9	Spryd4
1.889460695	4.68E-37	3.51E-41	9	Dcaf4
1.159339207	5.48E-37	4.10E-41	9	Tbx21
1.365562616	5.57E-37	4.17E-41	9	Zdhhc2
1.264400948	7.30E-37	5.47E-41	9	Gm614
1.157364873	1.17E-36	8.76E-41	9	Ap5b1
1.353239396	2.65E-36	1.99E-40	9	Hectd2
1.554137338	3.74E-36	2.80E-40	9	Heatr2
2.087796633	1.22E-35	9.17E-40	9	Gm16515
1.483198711	4.81E-35	3.60E-39	9	Bcl9
2.689163096	6.98E-35	5.23E-39	9	Bend4

1.069072266	9.16E-35	6.86E-39	9	Zfp873
2.439727733	1.20E-34	8.98E-39	9	Cspg4
2.401895045	2.89E-34	2.16E-38	9	Chaf1b
2.115558483	4.07E-34	3.05E-38	9	Tex9
1.236008	6.08E-34	4.55E-38	9	Wdr60
1.972169642	7.20E-34	5.39E-38	9	Pgm2
0.680596843	1.11E-33	8.35E-38	9	Tmem52b
1.112244437	7.84E-33	5.87E-37	9	Angpt2
1.344937508	8.54E-33	6.39E-37	9	Accs
2.192182648	1.46E-32	1.09E-36	9	Ccr9
2.117455313	1.86E-32	1.39E-36	9	Asf1b
1.643510647	2.67E-32	2.00E-36	9	Gnb1l
1.728789375	4.05E-32	3.03E-36	9	Gpr180
1.279589194	7.77E-32	5.82E-36	9	Lix1
1.138938891	2.22E-31	1.67E-35	9	Fsd1l
1.258971758	2.70E-31	2.02E-35	9	Zfp408
1.387481614	3.20E-31	2.40E-35	9	Aasdh
2.377308997	4.55E-31	3.41E-35	9	E2f2
1.492635908	8.74E-31	6.54E-35	9	BC017158
1.513909004	1.27E-30	9.54E-35	9	Ankrd32
1.850512053	4.66E-30	3.49E-34	9	Ndc1
1.393682428	9.78E-30	7.33E-34	9	Cdhr3
2.423559927	1.24E-29	9.31E-34	9	Cenpm
1.394727835	1.27E-29	9.52E-34	9	Abcb9
1.483416956	1.77E-29	1.33E-33	9	Rmdn1
1.666482554	2.91E-29	2.18E-33	9	Trmu
1.536873399	2.93E-29	2.19E-33	9	Areg
0.963556987	4.68E-29	3.51E-33	9	Tmem144
1.117404788	6.53E-29	4.89E-33	9	4930427A07Rik
1.150158815	1.04E-28	7.76E-33	9	Cpt2
1.249087548	1.10E-28	8.20E-33	9	Stbd1
1.722010009	3.58E-28	2.68E-32	9	Rab34
1.64642509	4.34E-28	3.25E-32	9	Wdr86
1.803833118	7.13E-28	5.34E-32	9	Zfp597
1.155490458	1.02E-27	7.64E-32	9	Nup43
1.473927601	1.17E-27	8.77E-32	9	Dtl
1.101662328	1.86E-27	1.40E-31	9	Fam189a2
1.423306959	4.77E-27	3.57E-31	9	Ffar2
1.482047902	7.02E-27	5.26E-31	9	BC027231
1.899677522	1.03E-26	7.68E-31	9	Tmem263
1.826309517	1.21E-26	9.09E-31	9	Mpi
1.4445129	1.32E-26	9.86E-31	9	Ptgfrn
1.324418957	3.16E-26	2.37E-30	9	Lrrc42
1.485334894	7.19E-26	5.39E-30	9	Bub1b
1.660373696	1.07E-25	8.02E-30	9	Asb2
1.522268777	1.46E-25	1.09E-29	9	Dut
1.180771442	4.83E-25	3.62E-29	9	1810055G02Rik
0.704031131	5.79E-25	4.34E-29	9	Hoxa7
1.806940805	6.34E-25	4.75E-29	9	Cox6a2
1.855845399	7.67E-25	5.75E-29	9	Map2k5
1.012957693	8.49E-25	6.36E-29	9	Txnrd3
1.909134266	9.46E-25	7.08E-29	9	Cd109
1.168765648	9.92E-25	7.43E-29	9	Gpr85
1.059179357	1.42E-24	1.06E-28	9	Ccsap
1.268179239	1.42E-24	1.07E-28	9	Spata2l
1.793645888	3.55E-24	2.66E-28	9	Ssx2ip
1.957261509	3.56E-24	2.67E-28	9	Gm14005
1.361970303	3.93E-24	2.94E-28	9	Tmem126b
1.709107563	4.34E-24	3.25E-28	9	Diap3
1.774663437	4.58E-24	3.43E-28	9	Rpa2
2.056040377	8.30E-24	6.21E-28	9	Dcbld2

1.057921756	9.15E-24	6.86E-28	9	Nanos1
1.604492698	1.32E-23	9.87E-28	9	Homer3
1.107103038	1.69E-23	1.27E-27	9	Hist1h4h
1.377472275	1.94E-23	1.45E-27	9	Mcm4
1.337366916	2.30E-23	1.73E-27	9	Fam65a
1.363069104	3.07E-23	2.30E-27	9	Noc4l
1.652903821	3.10E-23	2.32E-27	9	Lrrc28
1.429260583	3.19E-23	2.39E-27	9	Fbrsl1
1.332433248	4.71E-23	3.53E-27	9	Clpb
1.387212725	4.72E-23	3.54E-27	9	Pex26
1.271676037	1.04E-22	7.78E-27	9	Chchd6
1.505614841	1.12E-22	8.37E-27	9	Gins2
1.742450202	1.52E-22	1.14E-26	9	Usp1
1.81034159	2.31E-22	1.73E-26	9	Psat1
1.042735703	2.38E-22	1.79E-26	9	Mtbp
1.084631471	2.69E-22	2.01E-26	9	Ube2e2
1.694165983	3.97E-22	2.97E-26	9	Aig1
0.838598071	5.21E-22	3.91E-26	9	Hist2h2be
0.986177331	7.35E-22	5.51E-26	9	Pfkfb2
1.321546218	8.16E-22	6.11E-26	9	Morn2
1.239682894	1.01E-21	7.54E-26	9	Tpm2
2.112876318	1.03E-21	7.70E-26	9	Stambp
1.394355465	1.38E-21	1.03E-25	9	Ccdc91
1.359041605	2.03E-21	1.52E-25	9	Mlh1
1.877925613	2.24E-21	1.68E-25	9	Pnpla6
0.5442604	7.65E-21	5.73E-25	9	Ttc30a1
1.255426086	8.26E-21	6.19E-25	9	Zfp771
1.462490997	1.06E-20	7.91E-25	9	Ezh2
1.189941043	1.25E-20	9.36E-25	9	Pigo
1.412611669	1.56E-20	1.17E-24	9	Lyrm9
1.697647707	1.62E-20	1.21E-24	9	Haus4
2.447511163	2.12E-20	1.59E-24	9	Tprkb
1.549909622	2.48E-20	1.86E-24	9	3110040N11Rik
1.415737116	3.01E-20	2.25E-24	9	Ttpal
1.945066889	3.12E-20	2.33E-24	9	Pmm1
0.980918187	3.21E-20	2.40E-24	9	Loxl3
0.558246642	3.34E-20	2.50E-24	9	Mapk13
1.542622976	4.41E-20	3.30E-24	9	Dnah2
0.991424537	4.45E-20	3.33E-24	9	Tatdn3
1.414092877	5.42E-20	4.06E-24	9	Vti1a
1.131515291	5.79E-20	4.33E-24	9	Fignl1
1.380109938	7.60E-20	5.69E-24	9	Tstd3
1.047099672	9.17E-20	6.87E-24	9	Mcm10
1.129092853	1.14E-19	8.51E-24	9	Dnmbp
1.851705371	1.28E-19	9.61E-24	9	Ncapg
1.235557441	1.57E-19	1.18E-23	9	Xcr1
1.40423996	1.86E-19	1.39E-23	9	Rfc4
0.974924615	1.98E-19	1.48E-23	9	Arhgef5
1.51729017	2.37E-19	1.78E-23	9	Ccdc163
0.889100886	2.71E-19	2.03E-23	9	Fancg
1.487726488	3.65E-19	2.74E-23	9	Cdc37l1
1.75879934	3.76E-19	2.82E-23	9	Ubl4
1.181793568	3.85E-19	2.88E-23	9	Dnph1
1.318250492	4.25E-19	3.19E-23	9	Cenpv
1.283927596	4.61E-19	3.45E-23	9	Ada
1.388208695	4.65E-19	3.48E-23	9	Zranb3
1.67073966	4.82E-19	3.61E-23	9	Gmnn
1.62364457	5.31E-19	3.97E-23	9	Mcm5
1.187800756	6.20E-19	4.64E-23	9	Mcat
0.662866142	7.47E-19	5.59E-23	9	Dscc1
1.771127024	1.20E-18	8.99E-23	9	Flnb

2.667145978	1.73E-18	1.30E-22	9	Npy
0.924607575	1.74E-18	1.31E-22	9	Gcfc2
1.180332838	2.17E-18	1.62E-22	9	Mpp7
1.645233965	2.45E-18	1.84E-22	9	Smyd2
1.529964967	2.95E-18	2.21E-22	9	2210015D19Rik
0.986822494	3.17E-18	2.37E-22	9	Nmrk1
1.287455932	3.21E-18	2.40E-22	9	Tipin
1.046251705	3.63E-18	2.72E-22	9	Lrp4
1.081188648	5.49E-18	4.11E-22	9	Ints5
1.289260942	8.68E-18	6.50E-22	9	Scrib
1.285437577	9.68E-18	7.25E-22	9	Slc27a4
1.203577716	9.76E-18	7.31E-22	9	E2f6
1.1525687	1.38E-17	1.03E-21	9	Ctdspl
1.658421153	1.84E-17	1.37E-21	9	Usp38
1.947141785	1.84E-17	1.38E-21	9	Ivd
1.028045087	1.94E-17	1.45E-21	9	Rbm43
1.107549593	1.96E-17	1.47E-21	9	Ttc37
0.890691976	2.11E-17	1.58E-21	9	2700099C18Rik
1.439819099	2.20E-17	1.64E-21	9	Vps16
1.88054434	2.26E-17	1.69E-21	9	Snap47
0.930009645	2.52E-17	1.89E-21	9	Mlkl
1.438681551	2.55E-17	1.91E-21	9	Lrrc75a
1.694045276	2.82E-17	2.11E-21	9	Abhd11
1.316513778	3.03E-17	2.27E-21	9	Ptges3l
1.106147743	3.06E-17	2.29E-21	9	Cd34
1.443076501	3.24E-17	2.42E-21	9	Btbd2
1.270559788	3.32E-17	2.49E-21	9	Srr
1.461874759	3.47E-17	2.60E-21	9	Prim2
1.537558642	3.81E-17	2.85E-21	9	Carm1
1.345085594	3.98E-17	2.98E-21	9	Rbl1
1.264691539	4.78E-17	3.58E-21	9	Mib2
1.953863528	5.22E-17	3.91E-21	9	Clcc1
1.220686934	6.50E-17	4.87E-21	9	Dxo
1.133215972	6.91E-17	5.17E-21	9	R74862
1.485750238	7.63E-17	5.71E-21	9	Map3k9
1.702719605	8.98E-17	6.72E-21	9	Prkar2a
1.526612882	9.72E-17	7.28E-21	9	5730408K05Rik
1.32726391	1.00E-16	7.49E-21	9	Timp1
1.48242584	1.00E-16	7.51E-21	9	Pmvk
1.033997693	1.07E-16	8.00E-21	9	Plxdc1
0.827784462	1.33E-16	9.99E-21	9	Orc5
1.125520788	1.46E-16	1.09E-20	9	Gmppb
1.22673244	1.46E-16	1.10E-20	9	E230008N13Rik
1.301202093	1.63E-16	1.22E-20	9	Ece1
1.189175919	1.69E-16	1.27E-20	9	Dyrk2
1.643845925	1.87E-16	1.40E-20	9	Ctu2
1.408549262	2.01E-16	1.50E-20	9	Mad2l1
1.035849495	2.02E-16	1.51E-20	9	Col4a2
1.443891386	3.42E-16	2.56E-20	9	Cse1l
1.266858594	3.48E-16	2.61E-20	9	Psmg1
1.807952439	4.22E-16	3.16E-20	9	Cds1
1.200797835	4.26E-16	3.19E-20	9	Nup210
0.738039752	4.35E-16	3.25E-20	9	Fastkd5
1.1220606	4.52E-16	3.39E-20	9	1810034E14Rik
1.25282112	4.90E-16	3.67E-20	9	Nup160
1.180373627	5.04E-16	3.78E-20	9	Espl1
0.99360925	5.18E-16	3.88E-20	9	Rad51d
0.721887112	5.82E-16	4.36E-20	9	Mlst8
1.026312368	9.74E-16	7.30E-20	9	Runx2
1.843019778	1.05E-15	7.84E-20	9	Tmem63a
0.857763172	1.09E-15	8.18E-20	9	Traf4

1.532937227	1.14E-15	8.52E-20	9	A530064D06Rik
1.305096671	1.24E-15	9.27E-20	9	Casp2
0.774463443	1.27E-15	9.55E-20	9	Rad51
1.311346876	1.45E-15	1.08E-19	9	Polr1e
0.923605657	1.47E-15	1.10E-19	9	Gbe1
1.61903624	1.93E-15	1.45E-19	9	Riok1
1.415482846	2.49E-15	1.87E-19	9	Tmed8
0.684960282	2.64E-15	1.98E-19	9	Zfp951
1.360836118	2.98E-15	2.23E-19	9	Aaas
1.065999703	3.25E-15	2.44E-19	9	Apitd1
1.405544502	3.31E-15	2.48E-19	9	Slc25a44
1.560072932	3.34E-15	2.50E-19	9	Fert2
0.986449341	3.92E-15	2.93E-19	9	Zfp959
1.100025586	4.26E-15	3.19E-19	9	Hn1l
1.489215422	4.33E-15	3.25E-19	9	Zyg11b
1.571984179	4.72E-15	3.53E-19	9	Gm3219
1.779425231	6.00E-15	4.49E-19	9	H2-Oa
1.898731467	6.34E-15	4.75E-19	9	Dctpp1
0.904298747	6.82E-15	5.11E-19	9	Cenph
1.073013533	7.24E-15	5.42E-19	9	Snupn
1.495050219	7.86E-15	5.89E-19	9	Ccdc25
1.432173742	8.54E-15	6.39E-19	9	Gdap2
1.068586711	9.24E-15	6.92E-19	9	Tut1
1.253708882	1.22E-14	9.13E-19	9	Tk1
0.943860389	1.28E-14	9.55E-19	9	Enho
1.079278115	1.31E-14	9.78E-19	9	Smurf1
1.720754034	1.38E-14	1.03E-18	9	Tmem2
1.142819162	1.74E-14	1.30E-18	9	Cox18
0.705190767	1.80E-14	1.35E-18	9	Pim2
1.223331852	1.94E-14	1.46E-18	9	Pusl1
1.076498596	1.96E-14	1.47E-18	9	Haus7
1.279098007	2.38E-14	1.78E-18	9	Dhcr7
0.833465702	2.41E-14	1.81E-18	9	Kntc1
0.912660562	2.71E-14	2.03E-18	9	Alkbh4
1.471022432	2.86E-14	2.14E-18	9	Nlrp1b
0.912778414	3.00E-14	2.25E-18	9	Slc25a13
1.114001002	3.01E-14	2.25E-18	9	Atrip
1.123563598	3.05E-14	2.28E-18	9	Mcm2
1.027112172	3.23E-14	2.42E-18	9	Paxip1
1.279439258	3.27E-14	2.45E-18	9	Vps45
1.749863476	3.39E-14	2.54E-18	9	Ipo11
1.451855636	3.48E-14	2.61E-18	9	Nf2
1.423681252	3.55E-14	2.66E-18	9	Gtpbp1
1.185881657	3.55E-14	2.66E-18	9	Sco2
0.939775177	3.98E-14	2.98E-18	9	Bloc1s4
1.337888337	4.79E-14	3.59E-18	9	Nbeal2
1.226231234	5.71E-14	4.28E-18	9	Osbpl2
1.123748262	6.06E-14	4.54E-18	9	1700052N19Rik
0.94022876	7.18E-14	5.38E-18	9	Ric8b
0.666313905	8.56E-14	6.41E-18	9	Gcc1
1.147008048	9.10E-14	6.82E-18	9	Retsat
0.733155238	9.35E-14	7.00E-18	9	Ifitm6
0.854009566	1.02E-13	7.65E-18	9	Zfp346
0.805570368	1.11E-13	8.28E-18	9	Zfp946
1.285066309	1.23E-13	9.19E-18	9	Pola1
1.400907383	1.32E-13	9.87E-18	9	Taf6
1.137013293	1.33E-13	9.95E-18	9	Actl6a
1.508394774	1.47E-13	1.10E-17	9	Spire1
1.491445677	1.55E-13	1.16E-17	9	Ftsj1
1.152823539	1.69E-13	1.26E-17	9	Tbc1d19
1.504548561	2.10E-13	1.57E-17	9	Herc6

1.069885164	2.27E-13	1.70E-17	9	Arap2
1.048680381	2.37E-13	1.78E-17	9	Casc5
0.581301672	3.09E-13	2.32E-17	9	Mms22l
0.551229069	3.53E-13	2.65E-17	9	Cdc6
0.974520167	3.72E-13	2.79E-17	9	Cep57
0.83618426	3.73E-13	2.79E-17	9	Zadh2
0.910504795	4.05E-13	3.03E-17	9	B230217O12Rik
1.564754909	4.15E-13	3.11E-17	9	Zeb1
2.068570466	4.31E-13	3.23E-17	9	Mcm6
1.241963397	4.41E-13	3.30E-17	9	Rcbtb1
0.958093758	4.64E-13	3.47E-17	9	Dars2
1.158803722	4.83E-13	3.62E-17	9	Rnf24
1.105831223	5.37E-13	4.02E-17	9	Wdr47
1.645547202	5.64E-13	4.22E-17	9	Hells
0.810592581	5.95E-13	4.46E-17	9	Ppp1r3b
0.974924615	6.64E-13	4.97E-17	9	Acot11
0.983451143	6.76E-13	5.07E-17	9	Dppa3
1.265085968	6.93E-13	5.19E-17	9	Rpgrip1
1.221797507	8.30E-13	6.22E-17	9	Lonp1
1.658472711	8.64E-13	6.47E-17	9	Cdc42ep4
0.702676436	1.07E-12	8.05E-17	9	Klhl8
1.470453164	1.16E-12	8.68E-17	9	Tbc1d4
1.449129074	1.27E-12	9.49E-17	9	Kifap3
0.947924247	1.28E-12	9.59E-17	9	Eif5a2
1.277783523	1.50E-12	1.12E-16	9	Srgap3
0.960686524	1.57E-12	1.17E-16	9	Pik3c3
1.133855929	1.66E-12	1.24E-16	9	Thyn1
0.878956437	2.06E-12	1.55E-16	9	Mmachc
1.461705831	2.08E-12	1.56E-16	9	Dhx8
1.100064019	2.11E-12	1.58E-16	9	Mrps11
1.027628551	2.24E-12	1.68E-16	9	2810025M15Rik
1.061450542	2.27E-12	1.70E-16	9	Haus8
1.209825389	2.47E-12	1.85E-16	9	Cox19
0.987561042	2.49E-12	1.87E-16	9	Tmem238
0.906382422	2.52E-12	1.88E-16	9	Elk1
0.946525505	2.68E-12	2.00E-16	9	Ttll12
0.925263018	2.83E-12	2.12E-16	9	Ercc6l
0.824096056	3.03E-12	2.27E-16	9	Tmem154
1.091978295	3.83E-12	2.87E-16	9	Pla2g4a
1.602121493	4.03E-12	3.02E-16	9	Trmt1
0.836297067	4.71E-12	3.53E-16	9	Rasgrp3
1.111917693	4.77E-12	3.57E-16	9	Ei24
1.746397663	5.34E-12	4.00E-16	9	Pdpn
1.127592714	5.64E-12	4.23E-16	9	Trit1
1.222134421	5.80E-12	4.34E-16	9	Pknox1
0.803850737	5.94E-12	4.45E-16	9	Cd3eap
1.431679964	6.27E-12	4.70E-16	9	Mrps15
1.286543958	6.46E-12	4.84E-16	9	Sdhaf2
1.128022914	6.77E-12	5.07E-16	9	Hip1r
1.379532338	7.12E-12	5.33E-16	9	Leng1
0.887456825	7.47E-12	5.60E-16	9	Rbm12
1.317074983	7.90E-12	5.92E-16	9	5830418K08Rik
1.193513387	8.71E-12	6.52E-16	9	1700025G04Rik
0.775951557	8.76E-12	6.56E-16	9	Mbnl3
0.753874099	8.92E-12	6.68E-16	9	Pglyrp1
1.314536703	9.12E-12	6.83E-16	9	BC029214
1.253972597	9.88E-12	7.40E-16	9	Ramp3
0.907044777	1.10E-11	8.22E-16	9	Mllt1
1.385119088	1.20E-11	8.98E-16	9	Plac8
1.272549864	1.29E-11	9.70E-16	9	Pls3
0.809400758	1.31E-11	9.79E-16	9	Zdhhc14

0.885675387	1.32E-11	9.88E-16	9	Diexf
0.840687063	1.42E-11	1.06E-15	9	Prr5l
0.781810715	1.44E-11	1.08E-15	9	Atf7
0.766513449	1.45E-11	1.09E-15	9	6330407A03Rik
0.77326286	1.69E-11	1.27E-15	9	Cdca3
0.638331269	1.75E-11	1.31E-15	9	Tbcd
1.29282342	1.85E-11	1.38E-15	9	Ctdp1
0.797456296	2.03E-11	1.52E-15	9	Lsm11
0.983032706	2.13E-11	1.59E-15	9	Ckap2l
0.887501526	2.50E-11	1.87E-15	9	Tlcd2
1.046261313	2.57E-11	1.92E-15	9	Nrm
1.113640436	2.59E-11	1.94E-15	9	Tubgcp2
1.08639465	2.63E-11	1.97E-15	9	Arl13b
1.099229718	2.71E-11	2.03E-15	9	AB041803
0.812803132	2.78E-11	2.08E-15	9	Rnf141
0.934553419	2.83E-11	2.12E-15	9	Prmt3
0.890307013	3.12E-11	2.34E-15	9	Rqcd1
0.867146107	3.39E-11	2.54E-15	9	Myom1
1.823388647	3.43E-11	2.57E-15	9	Trappc9
1.157609881	3.60E-11	2.69E-15	9	Cherp
2.286680281	4.12E-11	3.09E-15	9	Agpat4
1.368057127	4.19E-11	3.14E-15	9	Ykt6
1.569155113	4.83E-11	3.62E-15	9	Pou2f1
1.316691317	5.07E-11	3.80E-15	9	Ide
1.55390642	5.17E-11	3.87E-15	9	Top2a
1.022190094	5.30E-11	3.97E-15	9	Gpr137
1.0941463	6.27E-11	4.69E-15	9	Tsc2
0.91052247	6.57E-11	4.92E-15	9	Ogfod3
0.92301841	6.86E-11	5.14E-15	9	Tmem260
1.096347411	7.71E-11	5.77E-15	9	Ubap1
1.134205727	8.45E-11	6.33E-15	9	Was
1.072920891	9.35E-11	7.00E-15	9	Dopey2
1.216946509	9.40E-11	7.04E-15	9	Ampd3
1.080698991	9.41E-11	7.05E-15	9	Ak3
0.382583171	1.06E-10	7.92E-15	9	Rad51c
0.984050251	1.11E-10	8.32E-15	9	Crip2
1.342654839	1.15E-10	8.65E-15	9	Trim11
1.287745972	1.28E-10	9.60E-15	9	Rdh13
1.254490333	1.37E-10	1.02E-14	9	Gys1
1.567689414	1.44E-10	1.08E-14	9	Lsm3
0.856180208	1.48E-10	1.11E-14	9	Cdyl2
1.035719763	1.50E-10	1.12E-14	9	Syce2
1.498183395	1.51E-10	1.13E-14	9	Snrpd3
1.153225638	1.51E-10	1.13E-14	9	Klf16
1.208867271	1.52E-10	1.13E-14	9	Scarb1
1.104918397	1.52E-10	1.14E-14	9	Dhx38
1.267767055	1.55E-10	1.16E-14	9	Lyst
0.760128079	1.58E-10	1.18E-14	9	4933427D14Rik
1.043612069	1.67E-10	1.25E-14	9	Akip1
0.900053898	1.88E-10	1.41E-14	9	Spsb1
1.13323825	1.91E-10	1.43E-14	9	Cnot11
0.862525896	1.96E-10	1.47E-14	9	Palld
1.208769602	1.98E-10	1.49E-14	9	Rrn3
1.468169363	2.32E-10	1.73E-14	9	Lima1
0.629038802	2.36E-10	1.77E-14	9	Zfp319
1.346162095	2.54E-10	1.90E-14	9	Mtmr10
1.27090825	2.58E-10	1.93E-14	9	Hirip3
1.647503517	2.68E-10	2.01E-14	9	Rfc1
0.872433834	2.79E-10	2.09E-14	9	Fam132a
1.468842737	2.92E-10	2.19E-14	9	Gpr35
1.346478675	3.23E-10	2.42E-14	9	Nsmce1

1.479321234	3.48E-10	2.61E-14	9	Gtf3a
1.285739601	4.21E-10	3.15E-14	9	Ngfrap1
1.438742838	5.14E-10	3.85E-14	9	Pde4a
1.225889712	5.29E-10	3.96E-14	9	Dus1l
0.690863874	5.29E-10	3.96E-14	9	Wbp1
0.996505974	5.80E-10	4.35E-14	9	Brwd1
0.911406644	5.98E-10	4.48E-14	9	Adamtsl5
1.25478458	6.05E-10	4.53E-14	9	Thoc7
1.108232184	6.17E-10	4.62E-14	9	Prim1
1.123713244	6.47E-10	4.85E-14	9	Pmpcb
0.631960719	6.81E-10	5.10E-14	9	Chek2
0.709316142	6.85E-10	5.13E-14	9	Arhgef18
1.08058191	7.88E-10	5.90E-14	9	Tceanc2
0.918670683	8.14E-10	6.10E-14	9	Ndufaf6
0.789640093	8.19E-10	6.14E-14	9	Mri1
0.99495841	8.42E-10	6.30E-14	9	Ino80e
1.402882278	8.43E-10	6.31E-14	9	Acot7
0.721594415	8.51E-10	6.37E-14	9	Lyrm5
0.979741404	8.64E-10	6.47E-14	9	Ubox5
1.194682636	9.49E-10	7.11E-14	9	Drg1
0.973442035	9.81E-10	7.35E-14	9	Tmcc2
1.739722515	1.05E-09	7.86E-14	9	Gpr114
0.926607699	1.06E-09	7.92E-14	9	Atf7ip
0.890536637	1.07E-09	8.04E-14	9	Lnp
1.076932285	1.08E-09	8.07E-14	9	Usp5
0.959437465	1.08E-09	8.07E-14	9	Dusp18
0.966753552	1.09E-09	8.13E-14	9	Ripk2
1.239988227	1.14E-09	8.56E-14	9	9130230L23Rik
0.767073318	1.24E-09	9.28E-14	9	Ncs1
0.838177878	1.33E-09	9.99E-14	9	Snip1
1.208903129	1.35E-09	1.01E-13	9	Sec31a
0.65105873	1.40E-09	1.05E-13	9	Gpx7
0.754398914	1.52E-09	1.14E-13	9	Cox15
0.72405351	1.54E-09	1.15E-13	9	Clspn
1.250979658	1.54E-09	1.15E-13	9	Ptplb
0.812557601	1.54E-09	1.16E-13	9	Ddx50
1.32957804	1.56E-09	1.17E-13	9	Tmem184c
0.918457202	1.67E-09	1.25E-13	9	Depdc5
1.392179185	1.86E-09	1.40E-13	9	Lzic
0.994177271	1.93E-09	1.44E-13	9	Mtif2
1.480372814	2.10E-09	1.57E-13	9	Nes
1.442477275	2.11E-09	1.58E-13	9	Ptdss1
0.985707424	2.17E-09	1.63E-13	9	Mina
1.127162108	2.23E-09	1.67E-13	9	Nxt1
1.183019486	2.43E-09	1.82E-13	9	Igf2bp2
0.781136123	2.70E-09	2.02E-13	9	Pcca
0.944530691	2.89E-09	2.17E-13	9	Mphosph9
0.935336771	3.00E-09	2.25E-13	9	Adora2b
0.877049859	3.03E-09	2.27E-13	9	Wdr12
0.868643839	3.16E-09	2.37E-13	9	Zfp938
1.11789153	3.26E-09	2.44E-13	9	Ufsp2
0.890431116	3.28E-09	2.46E-13	9	Aarsd1
0.583309559	3.30E-09	2.47E-13	9	Ankrd49
0.714281679	3.33E-09	2.49E-13	9	Rbks
1.417709405	3.51E-09	2.63E-13	9	Dpm1
0.914302678	3.57E-09	2.67E-13	9	Tmem209
1.207874361	3.58E-09	2.68E-13	9	2310061I04Rik
1.667382322	3.60E-09	2.69E-13	9	Spc24
1.194076004	3.71E-09	2.78E-13	9	AI314180
0.823702284	4.22E-09	3.16E-13	9	Ptcd3
1.302597174	4.29E-09	3.21E-13	9	Urm1

1.167245802	4.32E-09	3.24E-13	9	Poldip2
0.912127094	4.59E-09	3.43E-13	9	Trim35
0.851604923	4.62E-09	3.46E-13	9	Rnaseh2a
1.023566422	4.63E-09	3.47E-13	9	Mavs
1.374329208	4.72E-09	3.54E-13	9	Cers5
0.624997793	5.01E-09	3.75E-13	9	4831440E17Rik
1.122939727	5.03E-09	3.77E-13	9	Gm608
1.038698054	5.73E-09	4.29E-13	9	Atad3a
1.403603696	5.96E-09	4.47E-13	9	Cdk18
0.817725571	5.99E-09	4.49E-13	9	Actr5
0.800741556	6.08E-09	4.55E-13	9	Speg
0.87208954	6.10E-09	4.57E-13	9	Zfp746
1.049631928	6.14E-09	4.60E-13	9	Napg
0.916917603	6.56E-09	4.91E-13	9	Galnt2
1.027474261	6.59E-09	4.94E-13	9	Nudt2
0.980184715	6.67E-09	4.99E-13	9	Lyrm4
1.023972843	6.96E-09	5.21E-13	9	Cluh
0.827041426	7.37E-09	5.52E-13	9	Prr24
1.097155665	7.49E-09	5.61E-13	9	Hspa14
1.073650679	8.47E-09	6.34E-13	9	Luzp1
0.832772004	9.16E-09	6.86E-13	9	Fbxo42
0.989924961	9.61E-09	7.20E-13	9	Utp14a
1.164729985	9.79E-09	7.34E-13	9	Phf5a
1.070715979	9.90E-09	7.41E-13	9	Klrb1b
0.752946885	1.06E-08	7.91E-13	9	Tbc1d20
1.133487893	1.10E-08	8.22E-13	9	Gcnt1
0.728011549	1.10E-08	8.26E-13	9	Sh3rf1
0.950354901	1.10E-08	8.27E-13	9	Fbxo6
0.600893741	1.11E-08	8.28E-13	9	Gtpbp6
1.326043653	1.12E-08	8.41E-13	9	Sympk
0.870638823	1.18E-08	8.81E-13	9	Rfng
1.16362141	1.19E-08	8.95E-13	9	AA414768
0.94673239	1.25E-08	9.39E-13	9	Slu7
1.671636831	1.28E-08	9.57E-13	9	Il1r2
0.78396664	1.33E-08	9.96E-13	9	Ampd2
0.963982643	1.56E-08	1.17E-12	9	Mrpl46
1.082954283	1.60E-08	1.19E-12	9	Cab39l
1.059332314	1.63E-08	1.22E-12	9	Npat
1.165257681	1.76E-08	1.32E-12	9	Tcp1
1.146744449	1.79E-08	1.34E-12	9	Agap3
0.668837295	2.10E-08	1.57E-12	9	Micall1
0.866179679	2.19E-08	1.64E-12	9	Rabep2
0.898211974	2.22E-08	1.66E-12	9	Taf15
0.867193025	2.23E-08	1.67E-12	9	Pogz
0.671857057	2.28E-08	1.71E-12	9	Pggt1b
1.05933726	2.36E-08	1.77E-12	9	Zbtb1
1.071731349	2.44E-08	1.83E-12	9	Foxo1
0.855004056	2.79E-08	2.09E-12	9	Ccndbp1
1.301090034	2.87E-08	2.15E-12	9	Gpd2
1.230937768	3.19E-08	2.39E-12	9	Tmx4
1.24723462	3.31E-08	2.48E-12	9	Gnl3
1.003246231	3.70E-08	2.77E-12	9	Nfx1
1.063993602	3.71E-08	2.78E-12	9	Cpsf3l
0.897485082	3.80E-08	2.85E-12	9	Prrg2
0.748071045	4.07E-08	3.05E-12	9	Rrp15
0.81999096	4.12E-08	3.09E-12	9	Orc6
0.662845039	4.46E-08	3.34E-12	9	Nme6
0.821808807	4.74E-08	3.55E-12	9	Zc3h12d
0.579221194	5.10E-08	3.82E-12	9	Snora31
1.249266235	5.18E-08	3.88E-12	9	Isyna1
0.860581843	5.26E-08	3.94E-12	9	Plrg1

1.138454409	5.35E-08	4.01E-12	9	Tmem223
0.802354515	5.37E-08	4.02E-12	9	Aim2
1.050674393	5.49E-08	4.11E-12	9	Phf10
0.897044288	5.70E-08	4.27E-12	9	Senp1
0.663094408	5.96E-08	4.46E-12	9	Pcsk7
1.080320041	6.12E-08	4.58E-12	9	Eif1ad
1.164590612	6.15E-08	4.61E-12	9	Mrpl12
1.144431124	6.50E-08	4.87E-12	9	Zak
0.748871545	6.53E-08	4.89E-12	9	Ppat
0.581551512	6.55E-08	4.90E-12	9	Sp3os
0.934836895	6.78E-08	5.08E-12	9	Hectd3
0.981035417	6.86E-08	5.13E-12	9	Cdk14
1.032062512	6.93E-08	5.19E-12	9	Yif1a
0.992728877	7.82E-08	5.85E-12	9	Pomt1
0.517327517	8.78E-08	6.58E-12	9	Gm20324
1.323150525	9.16E-08	6.86E-12	9	Myd88
0.887751748	9.27E-08	6.94E-12	9	Ppp2cb
1.098600162	9.36E-08	7.01E-12	9	Phf20
0.684269548	9.73E-08	7.29E-12	9	Rhof
0.791404206	1.00E-07	7.49E-12	9	Sik3
0.901544295	1.11E-07	8.29E-12	9	Zdhhc18
0.92024969	1.13E-07	8.46E-12	9	Tatdn1
1.155178397	1.15E-07	8.62E-12	9	Tinf2
0.466968726	1.16E-07	8.66E-12	9	BC035044
1.291513609	1.20E-07	8.96E-12	9	Adam8
0.927327226	1.24E-07	9.28E-12	9	Clns1a
0.847332539	1.34E-07	1.00E-11	9	Lyar
0.713473801	1.37E-07	1.03E-11	9	Rfx3
0.981356911	1.45E-07	1.08E-11	9	Rab5a
1.104462297	1.46E-07	1.09E-11	9	Pon3
0.9552479	1.50E-07	1.12E-11	9	Crk
1.133158794	1.56E-07	1.17E-11	9	Anapc15
0.6684894	1.57E-07	1.17E-11	9	Stx17
1.141975218	1.58E-07	1.19E-11	9	Apex2
0.680902215	1.64E-07	1.23E-11	9	Tyk2
1.254360823	1.64E-07	1.23E-11	9	Clec5a
0.854162835	1.73E-07	1.29E-11	9	Arl4a
1.220878278	1.73E-07	1.29E-11	9	Snrpa1
0.499268009	1.81E-07	1.36E-11	9	Gramd4
0.913850796	1.84E-07	1.38E-11	9	Znhit2
0.802184697	1.89E-07	1.42E-11	9	Xlr4b
1.041770675	1.93E-07	1.45E-11	9	Uvrag
1.085307452	2.11E-07	1.58E-11	9	Naa38
1.042261761	2.26E-07	1.70E-11	9	Slc2a4rg-ps
1.767008459	2.30E-07	1.72E-11	9	Gpr4
0.766541796	2.31E-07	1.73E-11	9	Mycbp
1.185114498	2.43E-07	1.82E-11	9	AI462493
1.042696378	2.48E-07	1.85E-11	9	Bcap31
0.634411497	2.50E-07	1.87E-11	9	H2-Ke2
0.94087154	2.54E-07	1.90E-11	9	Psd4
1.273775225	2.59E-07	1.94E-11	9	Trmt6
1.021095823	2.66E-07	2.00E-11	9	Cyp4f16
0.699225213	2.68E-07	2.01E-11	9	Ticam2
0.837064122	2.91E-07	2.18E-11	9	Nolc1
1.273300503	2.98E-07	2.23E-11	9	Ankzf1
0.686966169	3.00E-07	2.25E-11	9	Ggct
0.846466435	3.14E-07	2.35E-11	9	Mut
0.902810999	3.23E-07	2.42E-11	9	Tusc1
0.76700057	3.24E-07	2.42E-11	9	Eftud2
1.217342873	3.31E-07	2.48E-11	9	Mpc2
0.675477869	3.32E-07	2.48E-11	9	Cirbp

0.642372119	3.37E-07	2.52E-11	9	Nlrp1a
0.819020011	3.38E-07	2.53E-11	9	Atg2a
1.075517504	3.41E-07	2.55E-11	9	Nmt2
0.885166852	3.45E-07	2.58E-11	9	Smim4
1.067371732	3.99E-07	2.99E-11	9	Pi4k2a
0.732795914	4.07E-07	3.05E-11	9	Acd
0.772044331	4.54E-07	3.40E-11	9	9630033F20Rik
0.977030555	4.67E-07	3.50E-11	9	Mfsd5
0.932216643	4.98E-07	3.73E-11	9	Mctp1
0.907951586	5.11E-07	3.83E-11	9	Ipmk
0.91793292	5.15E-07	3.86E-11	9	Acbd4
1.290697517	5.25E-07	3.93E-11	9	Polr2e
2.515764105	5.27E-07	3.94E-11	9	Tsen15
1.1237791	5.61E-07	4.20E-11	9	Zfml
0.833910977	5.78E-07	4.33E-11	9	Pde1b
0.852555221	5.89E-07	4.41E-11	9	Nomo1
0.845794585	6.01E-07	4.50E-11	9	Cdk1
1.133392055	6.04E-07	4.52E-11	9	Mvb12b
0.915191901	6.34E-07	4.75E-11	9	Akap11
0.989637517	6.38E-07	4.78E-11	9	Rbbp7
0.97450724	6.63E-07	4.97E-11	9	Trak2
0.940729945	6.72E-07	5.03E-11	9	Fars2
0.940725252	6.82E-07	5.11E-11	9	Amica1
0.927474899	6.95E-07	5.21E-11	9	Ift52
0.575689762	7.18E-07	5.37E-11	9	Csf1
0.825711923	7.23E-07	5.41E-11	9	Homer1
1.002914926	7.36E-07	5.51E-11	9	Fyttd1
0.620943702	7.95E-07	5.95E-11	9	Apeh
0.775213905	8.16E-07	6.11E-11	9	Piga
0.768055205	8.22E-07	6.15E-11	9	Pwwp2b
1.463681025	8.37E-07	6.27E-11	9	P2ry14
0.927474899	8.74E-07	6.54E-11	9	Rab3gap2
0.986392849	9.58E-07	7.18E-11	9	Esyt2
1.010169858	1.02E-06	7.63E-11	9	Med9
1.300583898	1.09E-06	8.13E-11	9	Poldip3
0.630065747	1.09E-06	8.14E-11	9	Exosc7
1.937497042	1.13E-06	8.45E-11	9	Zmym1
0.925141786	1.13E-06	8.50E-11	9	Emc1
0.607892303	1.17E-06	8.76E-11	9	Mcm7
0.691490637	1.19E-06	8.88E-11	9	Pdp1
0.659442654	1.20E-06	8.99E-11	9	Il1rl1
0.921237328	1.20E-06	9.02E-11	9	Cisd1
1.050410011	1.26E-06	9.43E-11	9	Ppp1r2
1.368281712	1.36E-06	1.02E-10	9	Fbrs
1.066195486	1.37E-06	1.02E-10	9	Enpp1
0.956242076	1.46E-06	1.09E-10	9	Cnpy3
0.904518523	1.59E-06	1.19E-10	9	Siva1
0.961271499	1.75E-06	1.31E-10	9	Bcl3
0.953534456	1.76E-06	1.32E-10	9	Elof1
1.058693345	1.76E-06	1.32E-10	9	Gmpr
0.890562112	1.86E-06	1.39E-10	9	Zhx1
0.794696993	1.87E-06	1.40E-10	9	Slc25a19
0.740508266	1.93E-06	1.45E-10	9	Klhdc4
-1.137821681	1.94E-06	1.46E-10	9	H2-K1
0.745389796	1.97E-06	1.47E-10	9	Pcp4l1
0.95107231	2.13E-06	1.60E-10	9	Cenpb
0.981941824	2.19E-06	1.64E-10	9	Ube2d2a
0.519477956	2.44E-06	1.83E-10	9	Tmem222
0.96307175	2.46E-06	1.84E-10	9	Trerf1
1.217247009	2.46E-06	1.84E-10	9	Usp3
0.736996359	2.49E-06	1.86E-10	9	Ralgapa1

0.63416853	2.56E-06	1.92E-10	9	Rprd1b
1.181847564	2.56E-06	1.92E-10	9	Zbtb18
0.522961072	2.66E-06	1.99E-10	9	Mov10
0.564110198	2.71E-06	2.03E-10	9	Gm166
-1.725822415	2.75E-06	2.06E-10	9	Sepp1
0.362856455	2.92E-06	2.18E-10	9	Igfbp6
0.715682666	2.95E-06	2.21E-10	9	Btbd6
1.369849055	3.07E-06	2.30E-10	9	Tubgcp4
1.39760722	3.19E-06	2.39E-10	9	Klrk1
0.667265379	3.59E-06	2.69E-10	9	Lztr1
0.950728685	3.70E-06	2.77E-10	9	Cct3
1.062364776	3.81E-06	2.85E-10	9	Itga6
0.698732633	3.84E-06	2.87E-10	9	Fancm
1.043892551	3.97E-06	2.97E-10	9	Aven
0.717972508	3.98E-06	2.98E-10	9	Gm12942
0.768316776	4.22E-06	3.16E-10	9	Bfar
0.934124568	4.28E-06	3.21E-10	9	Galnt12
1.078363086	4.35E-06	3.26E-10	9	Trnau1ap
1.057400997	4.42E-06	3.31E-10	9	Tspan4
0.611580564	4.69E-06	3.51E-10	9	Papolg
0.933123024	4.74E-06	3.55E-10	9	Anapc1
0.633857762	4.92E-06	3.69E-10	9	Lyrm1
1.281235804	4.99E-06	3.73E-10	9	Mrpl35
0.737331349	5.15E-06	3.86E-10	9	Tpx2
0.701889344	5.20E-06	3.89E-10	9	Prr5
1.024333828	5.23E-06	3.92E-10	9	Dpp4
0.971123816	5.65E-06	4.23E-10	9	Txnl1
0.668313049	5.68E-06	4.26E-10	9	Lsm14b
1.61959912	6.09E-06	4.56E-10	9	Wdfy4
0.943623032	6.09E-06	4.56E-10	9	Rae1
1.201397143	6.29E-06	4.71E-10	9	Zfp592
0.771526023	6.33E-06	4.74E-10	9	Apex1
0.897113987	6.43E-06	4.81E-10	9	Adh5
1.032835415	6.51E-06	4.87E-10	9	Scaf4
0.767403951	6.52E-06	4.88E-10	9	Dhfr
0.886200231	6.57E-06	4.92E-10	9	Smg1
0.987805568	6.60E-06	4.94E-10	9	Pde6d
0.827586892	6.86E-06	5.14E-10	9	Aifm1
1.040718099	7.04E-06	5.27E-10	9	Trip6
0.871276159	7.07E-06	5.29E-10	9	Nme1
1.090752455	7.09E-06	5.31E-10	9	Churc1
0.623254757	7.28E-06	5.45E-10	9	Prkar2b
0.85071162	7.47E-06	5.60E-10	9	Cwf19l2
0.790009983	7.48E-06	5.60E-10	9	4930503L19Rik
0.551640507	7.71E-06	5.78E-10	9	Dnlz
0.551788666	8.10E-06	6.06E-10	9	Atp8b2
0.538452923	8.80E-06	6.59E-10	9	Ddah2
1.851961267	9.31E-06	6.97E-10	9	Hdac5
0.441358261	9.59E-06	7.18E-10	9	Cyp4f13
0.732698184	9.67E-06	7.24E-10	9	Ubxn8
0.805852113	9.73E-06	7.29E-10	9	Zfp513
0.560542101	9.79E-06	7.33E-10	9	Cd40
0.479465764	9.83E-06	7.37E-10	9	Ifitm1
0.526938181	9.88E-06	7.40E-10	9	Cox11
0.737579711	1.03E-05	7.69E-10	9	Shkbp1
0.481740043	1.04E-05	7.78E-10	9	2610306M01Rik
0.866839832	1.04E-05	7.83E-10	9	Gnptab
0.863333964	1.05E-05	7.84E-10	9	Pde3b
0.654664656	1.05E-05	7.84E-10	9	Mcemp1
0.601304378	1.06E-05	7.93E-10	9	Ccdc88b
0.677791792	1.06E-05	7.97E-10	9	Gorab

0.928535526	1.06E-05	7.97E-10	9	Axin1
0.937604147	1.09E-05	8.13E-10	9	Fam110a
0.736001179	1.13E-05	8.46E-10	9	Tgs1
1.009585896	1.15E-05	8.61E-10	9	Med8
1.092140315	1.17E-05	8.79E-10	9	Ccl12
0.828781623	1.18E-05	8.82E-10	9	Slc20a1
0.571736196	1.18E-05	8.86E-10	9	Smad4
0.708772736	1.20E-05	8.96E-10	9	Rfwd3
1.031148544	1.20E-05	8.99E-10	9	Nans
0.638149632	1.24E-05	9.28E-10	9	Krr1
0.817257921	1.25E-05	9.34E-10	9	Slc31a1
0.426128019	1.28E-05	9.60E-10	9	5430416N02Rik
0.872703951	1.32E-05	9.89E-10	9	Med25
0.819539833	1.35E-05	1.01E-09	9	Sephs1
0.827038098	1.36E-05	1.02E-09	9	Rnf135
0.849767491	1.37E-05	1.02E-09	9	Abhd5
0.793279021	1.38E-05	1.03E-09	9	2700094K13Rik
0.553460097	1.39E-05	1.04E-09	9	Pus1
0.800456856	1.39E-05	1.04E-09	9	Edc4
0.565313809	1.40E-05	1.05E-09	9	Hdc
0.605935641	1.44E-05	1.08E-09	9	Ciapin1
0.886164026	1.45E-05	1.08E-09	9	Ltv1
0.785355417	1.46E-05	1.10E-09	9	Pank2
0.811941806	1.48E-05	1.11E-09	9	Usp39
0.452860518	1.50E-05	1.12E-09	9	Rc3h2
1.064947877	1.52E-05	1.13E-09	9	Chchd4
1.068633933	1.59E-05	1.19E-09	9	Angptl2
0.881755788	1.63E-05	1.22E-09	9	Fam46c
0.613395005	1.64E-05	1.23E-09	9	Tmem120b
0.922860104	1.66E-05	1.24E-09	9	Tnfrsf11a
1.257362195	1.70E-05	1.27E-09	9	Dhps
-0.716861763	1.73E-05	1.29E-09	9	Itm2b
0.297327987	1.75E-05	1.31E-09	9	Slc25a38
0.911841157	1.77E-05	1.33E-09	9	Abr
0.767026216	1.78E-05	1.34E-09	9	Tubb5
0.820607584	1.81E-05	1.35E-09	9	Peak1
0.744931095	1.83E-05	1.37E-09	9	Tom1l2
0.862771054	1.85E-05	1.39E-09	9	Csnk2a1
0.674713288	1.88E-05	1.40E-09	9	Ndufv1
0.66027613	1.89E-05	1.41E-09	9	Rbm10
0.729106148	1.90E-05	1.42E-09	9	Impa1
0.724276923	1.90E-05	1.43E-09	9	Med10
0.674194666	1.98E-05	1.48E-09	9	Ptcd2
0.865665834	2.01E-05	1.50E-09	9	Imp4
0.711729302	2.01E-05	1.51E-09	9	Scly
0.723745075	2.10E-05	1.57E-09	9	Ash2l
0.819496165	2.12E-05	1.59E-09	9	Acly
0.926888547	2.18E-05	1.63E-09	9	Rnf187
0.438320582	2.22E-05	1.66E-09	9	Orc2
0.447193107	2.23E-05	1.67E-09	9	Dlg3
0.94186926	2.27E-05	1.70E-09	9	Kpnb1
0.559134249	2.29E-05	1.71E-09	9	Vps37c
-1.664085368	2.30E-05	1.72E-09	9	Zfp36
0.659985895	2.34E-05	1.75E-09	9	Smim12
0.480461172	2.43E-05	1.82E-09	9	Tjp2
0.553729696	2.48E-05	1.86E-09	9	Zfp317
1.14796252	2.59E-05	1.94E-09	9	Trim12a
0.541673076	2.61E-05	1.95E-09	9	Rab3gap1
0.902555989	2.61E-05	1.95E-09	9	Trex1
0.936513006	2.62E-05	1.96E-09	9	Eif4e2
0.448863146	2.65E-05	1.98E-09	9	1110004E09Rik

0.718117878	2.70E-05	2.02E-09	9	9430008C03Rik
0.843186442	2.80E-05	2.10E-09	9	Mrpl28
0.805068433	2.82E-05	2.11E-09	9	Rpa1
0.925755236	2.84E-05	2.13E-09	9	Mcts1
0.60115147	2.86E-05	2.14E-09	9	Mrps27
0.828161749	2.98E-05	2.23E-09	9	Lig3
1.086786505	2.99E-05	2.24E-09	9	Gopc
0.724765426	3.03E-05	2.27E-09	9	Imp3
0.90256004	3.03E-05	2.27E-09	9	Jmjd6
0.764452231	3.10E-05	2.33E-09	9	Dyrk3
0.371314252	3.11E-05	2.33E-09	9	Vps9d1
0.7132904	3.22E-05	2.41E-09	9	Phf13
0.493655902	3.35E-05	2.51E-09	9	Zfp664
1.256494605	3.36E-05	2.51E-09	9	2010012O05Rik
0.891278243	3.51E-05	2.63E-09	9	Zfr
0.821744084	3.63E-05	2.72E-09	9	Ndufv2
1.016565526	3.66E-05	2.74E-09	9	Ppil4
0.526978621	3.69E-05	2.76E-09	9	Phtf2
0.475563869	3.73E-05	2.79E-09	9	Eno2
0.736211506	3.87E-05	2.90E-09	9	Rhbdd1
0.864905246	3.90E-05	2.92E-09	9	Tctex1d2
0.627376709	4.15E-05	3.11E-09	9	Prosc
0.688141173	4.17E-05	3.12E-09	9	Zfp709
1.473531843	4.22E-05	3.16E-09	9	1500011B03Rik
0.853179634	4.24E-05	3.18E-09	9	Pts
0.786747768	4.39E-05	3.29E-09	9	Wwc2
0.472041076	4.41E-05	3.30E-09	9	Serpinb9
0.833150932	4.42E-05	3.31E-09	9	Baz2a
0.573546881	4.63E-05	3.47E-09	9	Tbc1d14
0.735444644	4.71E-05	3.53E-09	9	Pdha1
1.268347264	4.71E-05	3.53E-09	9	Eif2ak3
0.701489584	4.71E-05	3.53E-09	9	E330033B04Rik
0.63012652	4.75E-05	3.56E-09	9	Arfgap3
0.565611428	4.80E-05	3.60E-09	9	Yipf1
0.741210493	4.89E-05	3.66E-09	9	Htatip2
1.000553297	4.93E-05	3.69E-09	9	Napsa
0.52600696	4.93E-05	3.69E-09	9	Lancl2
0.905723861	4.96E-05	3.72E-09	9	Pik3ca
0.713175296	4.97E-05	3.72E-09	9	Slc7a1
0.826380104	5.21E-05	3.90E-09	9	Whsc1
0.826953586	5.25E-05	3.93E-09	9	Acsl5
1.137357752	5.27E-05	3.95E-09	9	Pramef8
0.567097182	5.32E-05	3.98E-09	9	Timm50
0.985107445	5.37E-05	4.02E-09	9	Cdc37
0.704394042	5.45E-05	4.08E-09	9	Nme4
0.65225355	5.51E-05	4.13E-09	9	Pogk
0.512623315	5.52E-05	4.14E-09	9	Mrps12
0.874007335	5.60E-05	4.19E-09	9	Fkbp15
0.686056302	5.80E-05	4.34E-09	9	Dgcr14
0.50020041	5.82E-05	4.36E-09	9	Rcl1
0.512536269	5.85E-05	4.38E-09	9	Morc2a
0.666539916	6.04E-05	4.52E-09	9	Lrch1
0.368033768	6.23E-05	4.67E-09	9	Dcun1d3
0.691423026	6.26E-05	4.69E-09	9	Chd1l
1.023497705	6.30E-05	4.72E-09	9	Gtf2f1
0.709781725	6.46E-05	4.83E-09	9	Zzef1
0.836940698	6.65E-05	4.98E-09	9	Camk2g
0.956289705	6.67E-05	5.00E-09	9	Srp19
0.790654386	6.67E-05	5.00E-09	9	BC028528
0.785471229	6.73E-05	5.04E-09	9	Nrp2
0.657486526	6.97E-05	5.22E-09	9	R3hcc1l

0.982987447	7.36E-05	5.51E-09	9	Dcaf12
0.494935333	7.51E-05	5.63E-09	9	Polr1a
0.691064536	7.67E-05	5.74E-09	9	Arpp19
0.5547317	7.76E-05	5.81E-09	9	Ube4b
0.620323407	7.77E-05	5.82E-09	9	Stim1
0.682772064	7.93E-05	5.94E-09	9	Ssrp1
1.018419381	7.98E-05	5.98E-09	9	Akr1b8
1.242927374	8.00E-05	5.99E-09	9	Prdx4
1.018881198	8.08E-05	6.05E-09	9	Ugp2
1.311918795	8.08E-05	6.05E-09	9	Mthfd2
0.562896044	8.34E-05	6.24E-09	9	Srm
0.5234504	8.89E-05	6.66E-09	9	Isy1
1.097975073	8.90E-05	6.67E-09	9	Efemp2
0.770196145	9.08E-05	6.80E-09	9	Uspl1
0.590086212	9.11E-05	6.82E-09	9	Ttc12
0.909610285	9.16E-05	6.86E-09	9	Mbtd1
0.965966937	9.35E-05	7.00E-09	9	Ctps2
0.937818788	9.55E-05	7.15E-09	9	Rpp21
0.653615509	9.82E-05	7.35E-09	9	Golga3
0.507137546	0.000100598	7.53E-09	9	Dkc1
0.53954341	0.000100821	7.55E-09	9	Fhl3
1.08203948	0.000100828	7.55E-09	9	Polr2f
0.728042877	0.000105186	7.88E-09	9	Ift22
0.751899765	0.000107605	8.06E-09	9	Gfer
1.013318849	0.000107886	8.08E-09	9	0610011F06Rik
1.042447923	0.000109013	8.16E-09	9	Mrps25
0.879902166	0.00010915	8.17E-09	9	Map3k14
0.754044974	0.000114389	8.57E-09	9	Dph3
0.898158528	0.000116023	8.69E-09	9	Aph1c
0.660697359	0.000117396	8.79E-09	9	Lrp8
0.655630221	0.000117595	8.81E-09	9	Ids
0.813873093	0.000118004	8.84E-09	9	Tpm1
1.083646233	0.0001188	8.90E-09	9	Tec
0.866207414	0.000118864	8.90E-09	9	G6pdx
1.011589039	0.000119322	8.94E-09	9	Cops7a
0.78529578	0.000122948	9.21E-09	9	Clec4e
0.678663252	0.000129599	9.71E-09	9	Chst11
0.576667643	0.000130134	9.75E-09	9	Med31
1.118298365	0.000132062	9.89E-09	9	Ilkap
0.993695134	0.000132971	9.96E-09	9	Lonrf3
0.552784279	0.000133405	9.99E-09	9	Crcp
0.81425789	0.000135438	1.01E-08	9	Sdf4
1.001389042	0.000142576	1.07E-08	9	Aldh3b1
0.821423078	0.000150716	1.13E-08	9	Pin1
0.742458092	0.000151449	1.13E-08	9	Aprt
0.530882274	0.000151836	1.14E-08	9	Itgb1bp1
1.006290083	0.000165689	1.24E-08	9	Olfm1
0.682816929	0.000167477	1.25E-08	9	Rpl27a
0.411615925	0.000168694	1.26E-08	9	Gm2027
0.644743594	0.0001696	1.27E-08	9	Rab12
0.566628042	0.000171371	1.28E-08	9	Cdyl
0.80510672	0.000174846	1.31E-08	9	Exosc4
0.567780455	0.000176594	1.32E-08	9	Icmt
0.605174415	0.000179129	1.34E-08	9	Ppm1m
0.576009098	0.000182138	1.36E-08	9	Polr2c
0.653752173	0.000183699	1.38E-08	9	D10Wsu102e
0.547516286	0.000187141	1.40E-08	9	Uchl4
0.622379316	0.000187518	1.40E-08	9	Trak1
0.561109472	0.000187614	1.41E-08	9	Nuak2
1.008577182	0.000188702	1.41E-08	9	Agpat6
0.719950766	0.000190535	1.43E-08	9	Rab22a

0.922452132	0.00019968	1.50E-08	9	Arl6ip5
0.704802511	0.000202722	1.52E-08	9	Gapvd1
0.81235571	0.000206521	1.55E-08	9	Spcs3
0.642537061	0.000209697	1.57E-08	9	Ccdc82
0.467106476	0.000212582	1.59E-08	9	Fam58b
0.654696078	0.000217662	1.63E-08	9	Tiprl
0.826340217	0.000219496	1.64E-08	9	8430427H17Rik
0.852995882	0.000222417	1.67E-08	9	Plscr1
0.65004188	0.000230814	1.73E-08	9	Tmem68
0.413709635	0.000236295	1.77E-08	9	Phf11c
0.773552132	0.000248186	1.86E-08	9	Eprs
0.893349432	0.000256089	1.92E-08	9	Fgfr1op
0.909971499	0.000260838	1.95E-08	9	Nop58
0.712444692	0.000269087	2.02E-08	9	Supt16
0.583245847	0.000269746	2.02E-08	9	2810417H13Rik
0.730272971	0.000276007	2.07E-08	9	Nod2
0.704857044	0.000279593	2.09E-08	9	Pi4kb
0.816269419	0.000283878	2.13E-08	9	Sdhd
0.579054145	0.000290943	2.18E-08	9	Ccr7
0.454078603	0.000296348	2.22E-08	9	Hspa4l
0.800413684	0.000305298	2.29E-08	9	Ccdc109b
0.623196363	0.000307533	2.30E-08	9	Dhx9
0.819135938	0.000309014	2.31E-08	9	Ppm1h
0.708155216	0.000310992	2.33E-08	9	Sf3b6
0.770108222	0.000319294	2.39E-08	9	Zcwpw1
0.921333386	0.000320008	2.40E-08	9	Anapc4
0.582956137	0.000323962	2.43E-08	9	Slc35b4
0.942743215	0.00032915	2.47E-08	9	Hiat1
0.873327688	0.000331099	2.48E-08	9	Atxn1l
0.564420234	0.000342188	2.56E-08	9	Poc1b
0.45748366	0.000348284	2.61E-08	9	Tfpi
0.884994104	0.000355212	2.66E-08	9	Dnajc4
0.64855311	0.000355451	2.66E-08	9	Fubp1
0.732783945	0.000356419	2.67E-08	9	2300009A05Rik
0.800479203	0.000358213	2.68E-08	9	Ptrh2
0.865792249	0.000361415	2.71E-08	9	Sipa1
0.571646655	0.000365813	2.74E-08	9	Nup54
0.739479418	0.000372219	2.79E-08	9	Pqlc1
0.394061992	0.000373921	2.80E-08	9	Rps24
0.701089984	0.000374593	2.81E-08	9	B630005N14Rik
1.042619255	0.000375203	2.81E-08	9	E2f1
1.047053852	0.000376978	2.82E-08	9	Sgsm2
0.747814177	0.000380907	2.85E-08	9	Gabpb1
0.619432649	0.00039314	2.94E-08	9	Yipf5
0.677107845	0.000422729	3.17E-08	9	Plk3
1.101167827	0.000425327	3.19E-08	9	Lrwd1
0.771407388	0.000427127	3.20E-08	9	Taf5l
0.771554117	0.000431894	3.23E-08	9	Ncbp1
0.640253873	0.000435152	3.26E-08	9	Fam204a
0.951208821	0.000439204	3.29E-08	9	Sos1
0.334973883	0.000443352	3.32E-08	9	Pde7a
-0.559910095	0.000452818	3.39E-08	9	Malat1
0.835788242	0.000452834	3.39E-08	9	Pdlim1
0.418408156	0.000453522	3.40E-08	9	Ppp1r13l
0.431782416	0.000460826	3.45E-08	9	Dhodh
0.877152098	0.000466939	3.50E-08	9	Psmd1
0.611140842	0.00048915	3.66E-08	9	Msmo1
1.147111421	0.000506445	3.79E-08	9	Selm
0.638551595	0.000519371	3.89E-08	9	Atp2b4
0.610675208	0.000535637	4.01E-08	9	Pkm
0.880789846	0.000568858	4.26E-08	9	Mkl1

0.489490332	0.000571784	4.28E-08	9	Dhdds
0.50517905	0.000578748	4.33E-08	9	Zfp275
0.565189392	0.000582555	4.36E-08	9	Atg13
0.752677067	0.00058456	4.38E-08	9	Cd200r1
0.831811762	0.000585931	4.39E-08	9	Snhg1
0.319245941	0.000587914	4.40E-08	9	Polk
0.384019282	0.000597665	4.48E-08	9	Nusap1
0.753514839	0.000610995	4.58E-08	9	Dapp1
0.628343517	0.000649734	4.87E-08	9	Parvb
0.495470236	0.000650323	4.87E-08	9	Lmnb2
0.664670057	0.000650553	4.87E-08	9	Zfp637
0.421998073	0.00067	5.02E-08	9	Commd5
0.562922199	0.000671958	5.03E-08	9	Serbp1
1.187136209	0.000686003	5.14E-08	9	Hs6st1
0.666950032	0.000702572	5.26E-08	9	Kit
0.535591615	0.000708679	5.31E-08	9	Orai1
0.667624047	0.000718321	5.38E-08	9	Brix1
0.783455363	0.000743062	5.57E-08	9	Mrpl38
0.787851433	0.000785373	5.88E-08	9	Hcst
0.69100735	0.000795803	5.96E-08	9	Slc25a24
0.794712015	0.000811838	6.08E-08	9	Trappc6a
0.646161784	0.000824112	6.17E-08	9	Cnot3
0.61716096	0.000828577	6.21E-08	9	Atp5d
0.498932443	0.000846169	6.34E-08	9	Sel1l3
0.727787502	0.000850184	6.37E-08	9	Plxna1
0.666635113	0.00085576	6.41E-08	9	Vps26b
0.450845955	0.000859516	6.44E-08	9	Kctd3
0.610544851	0.000880539	6.59E-08	9	Clk3
0.775614086	0.000880875	6.60E-08	9	Kdm1b
0.750002231	0.000893066	6.69E-08	9	Nfyc
0.872806176	0.000893154	6.69E-08	9	Rilpl1
0.649417436	0.000898877	6.73E-08	9	Smim11
0.562521359	0.000906465	6.79E-08	9	Irf3
0.818943589	0.000915103	6.85E-08	9	Ergic1
0.535339734	0.00094939	7.11E-08	9	Arrdc1
0.702206151	0.00095427	7.15E-08	9	Tcp11l1
0.668090224	0.000957391	7.17E-08	9	Nop2
0.902616266	0.000967464	7.25E-08	9	Gpr171
0.707054046	0.00096945	7.26E-08	9	Tprgl
0.476902852	0.000985686	7.38E-08	9	Mta1
0.650768739	0.001000101	7.49E-08	9	Kbtbd4
0.341117515	0.001016505	7.61E-08	9	Heatr5a
0.530809602	0.001048079	7.85E-08	9	Brcc3
0.802275509	0.00106665	7.99E-08	9	Ppm1g
0.809963566	0.00106894	8.01E-08	9	Slc16a6
0.685289485	0.001069068	8.01E-08	9	5830432E09Rik
0.813593334	0.001076168	8.06E-08	9	Ubqln4
0.835705971	0.001081471	8.10E-08	9	Nop14
0.629109614	0.001083235	8.11E-08	9	Mtf1
0.679794597	0.001102535	8.26E-08	9	Capn2
0.678536117	0.001110913	8.32E-08	9	A430005L14Rik
0.61272023	0.001111005	8.32E-08	9	Itgb2
0.587907562	0.001137702	8.52E-08	9	Zfyve21
0.722603866	0.001141848	8.55E-08	9	Cope
0.953326604	0.001154227	8.64E-08	9	Casp4
0.416850003	0.001174506	8.80E-08	9	Zfp68
1.011821173	0.001176656	8.81E-08	9	Tgm2
0.764668365	0.001180318	8.84E-08	9	Gpr137b-ps
0.413818141	0.00118069	8.84E-08	9	Rtcb
0.513694905	0.001183537	8.86E-08	9	Senp7
0.549582392	0.00118364	8.86E-08	9	Rab28

0.36924234	0.001202419	9.01E-08	9	Luc7l
0.324879788	0.001268933	9.50E-08	9	Nae1
0.425283334	0.001270625	9.52E-08	9	Usp19
0.527487661	0.001271622	9.52E-08	9	Acta2
0.861916776	0.001282609	9.61E-08	9	Apbb2
0.648110341	0.001287353	9.64E-08	9	Utp6
0.704107197	0.001351692	1.01E-07	9	Spp1
0.547421958	0.001370796	1.03E-07	9	Slc2a6
1.026186674	0.001386417	1.04E-07	9	H2-Ob
0.58154605	0.001398582	1.05E-07	9	Zfp715
0.606592131	0.001423979	1.07E-07	9	Aqr
0.432064889	0.001429679	1.07E-07	9	Rgs18
0.788751744	0.001430203	1.07E-07	9	Prrc2b
0.401584029	0.001471493	1.10E-07	9	Csrp2bp
0.623907973	0.001489129	1.12E-07	9	Klhl7
0.60319803	0.001491284	1.12E-07	9	Mtus1
0.513242405	0.00151298	1.13E-07	9	Ebna1bp2
0.571432774	0.001516399	1.14E-07	9	Suz12
0.766427865	0.001526096	1.14E-07	9	Jkamp
0.71405083	0.00155445	1.16E-07	9	Asxl2
0.368090482	0.001558513	1.17E-07	9	Arl6ip4
0.406955892	0.001574982	1.18E-07	9	Cep76
0.975619685	0.001589193	1.19E-07	9	Otud5
0.7412021	0.001598176	1.20E-07	9	Ndufs6
0.866414824	0.001703958	1.28E-07	9	Nup155
0.701044773	0.001751586	1.31E-07	9	Cdc42se2
0.711344311	0.001799361	1.35E-07	9	Lrch3
0.373262149	0.001803763	1.35E-07	9	Plxna4os1
0.637270595	0.001810491	1.36E-07	9	Socs7
0.427343408	0.001836895	1.38E-07	9	Rps3
0.579152377	0.001916147	1.44E-07	9	Slc2a1
0.677535119	0.001922323	1.44E-07	9	Mrpl42
0.639587449	0.001925304	1.44E-07	9	C1qbp
0.42511913	0.001934192	1.45E-07	9	Plxna4
0.722746686	0.001938668	1.45E-07	9	Cytip
0.623776426	0.002013506	1.51E-07	9	Mettl23
0.380416591	0.002016944	1.51E-07	9	Phlpp2
0.592611536	0.002019988	1.51E-07	9	Tpr
0.868100343	0.002045316	1.53E-07	9	Map4k4
0.524503801	0.00211756	1.59E-07	9	Tmem129
0.431979441	0.002121567	1.59E-07	9	Nrp
0.915507551	0.002179226	1.63E-07	9	Tmed3
0.571349611	0.002192902	1.64E-07	9	Sfxn1
0.917511888	0.002278569	1.71E-07	9	Zdhhc7
1.881022695	0.002324585	1.74E-07	9	Keap1
1.776404081	0.002337909	1.75E-07	9	Ralgps2
0.347488953	0.002338701	1.75E-07	9	Alad
0.550391765	0.002354437	1.76E-07	9	Ddx6
0.497323804	0.002362494	1.77E-07	9	Sertad2
0.775822331	0.00238856	1.79E-07	9	Plxnb2
0.576692042	0.002462335	1.84E-07	9	Brd8
0.776944152	0.002508699	1.88E-07	9	N6amt1
0.555476262	0.00252429	1.89E-07	9	Ccs
-1.015282008	0.002543922	1.91E-07	9	Junb
0.535277513	0.002604712	1.95E-07	9	Cdk5
0.663956235	0.002606183	1.95E-07	9	Paf1
0.978623041	0.002640849	1.98E-07	9	Atp7a
1.215079129	0.002713518	2.03E-07	9	Dusp16
0.474973238	0.002725917	2.04E-07	9	Senp5
0.368466609	0.002776586	2.08E-07	9	Chid1
0.744062778	0.002778025	2.08E-07	9	Fundc2

0.71679069	0.002841603	2.13E-07	9	1110008F13Rik
0.971393593	0.002863592	2.14E-07	9	Gmps
0.578336711	0.002898679	2.17E-07	9	Gpr107
0.559864903	0.002998605	2.25E-07	9	Commd3
0.498481766	0.003000214	2.25E-07	9	Havcr2
0.883452381	0.003057053	2.29E-07	9	Milr1
0.77985916	0.003097905	2.32E-07	9	Lmf2
0.543063034	0.003115777	2.33E-07	9	Fam134b
0.812206371	0.003214544	2.41E-07	9	Rnf7
0.60068379	0.003232754	2.42E-07	9	Hspa13
0.565033893	0.003242821	2.43E-07	9	Pml
0.339350614	0.003273326	2.45E-07	9	Thap3
0.553749726	0.003321582	2.49E-07	9	Inpp5b
0.860865512	0.003328727	2.49E-07	9	Sec61a1
0.528372089	0.003338029	2.50E-07	9	Tmem50b
0.429664313	0.00334925	2.51E-07	9	Chd1
0.803242313	0.003424211	2.56E-07	9	Ube2l3
0.856548283	0.003426705	2.57E-07	9	Hat1
0.421597605	0.003518679	2.64E-07	9	Ly6d
0.671391771	0.00357762	2.68E-07	9	Acadvl
0.701517988	0.003598318	2.69E-07	9	Slc39a6
0.555585549	0.00364298	2.73E-07	9	Aldh4a1
0.420484113	0.003666717	2.75E-07	9	Ncaph2
0.725949245	0.003704789	2.77E-07	9	Mtmr1
0.567740667	0.003766942	2.82E-07	9	Rars
0.886174983	0.003773745	2.83E-07	9	Fn1
0.4127812	0.003779677	2.83E-07	9	Rcor3
0.521320926	0.003847628	2.88E-07	9	Mcur1
0.620924875	0.003890909	2.91E-07	9	Pdf
0.63176597	0.00397467	2.98E-07	9	Fosl1
0.424848131	0.00403006	3.02E-07	9	Srpr
0.772927467	0.004067336	3.05E-07	9	Ybx3
0.314590572	0.004080191	3.06E-07	9	Pdcd1lg2
0.631486546	0.004080694	3.06E-07	9	Atp5g3
0.276715062	0.004094841	3.07E-07	9	Mdm1
0.486253581	0.004099873	3.07E-07	9	Fat3
0.637972807	0.004173566	3.13E-07	9	Blvra
0.360536163	0.004258572	3.19E-07	9	Manea
0.84984529	0.004294743	3.22E-07	9	0610010K14Rik
0.660653135	0.004431177	3.32E-07	9	Nop9
0.545568554	0.004494133	3.37E-07	9	Rpl14
0.715494996	0.004555141	3.41E-07	9	Ift27
0.749520644	0.004572332	3.42E-07	9	Setd1a
0.420535072	0.004627658	3.47E-07	9	Rtn1
0.596292822	0.004628761	3.47E-07	9	Cers2
0.752191182	0.004737533	3.55E-07	9	Upf3a
0.582673703	0.004738429	3.55E-07	9	Leprot
0.422129531	0.004751253	3.56E-07	9	Ctr9
0.384486462	0.005014982	3.76E-07	9	Brip1
0.635705018	0.00502294	3.76E-07	9	Ptk2b
0.603131578	0.005070841	3.80E-07	9	S100a11
1.104643854	0.005292648	3.96E-07	9	Eif1b
0.612462445	0.005360519	4.01E-07	9	Epg5
0.402455556	0.005415229	4.06E-07	9	Clasp2
0.860518126	0.005418108	4.06E-07	9	Uso1
0.566932638	0.005418783	4.06E-07	9	Orc4
0.431587237	0.005491164	4.11E-07	9	Lancl1
0.635624406	0.005523872	4.14E-07	9	Sec11c
0.687451533	0.005581075	4.18E-07	9	Csf2rb2
0.572431277	0.005706473	4.27E-07	9	Scamp5
0.765943031	0.005757785	4.31E-07	9	Hk2

0.752260971	0.00577921	4.33E-07	9	Ltbp3
0.688466324	0.005842111	4.38E-07	9	Inf2
0.477665611	0.006043952	4.53E-07	9	Hk1
-0.863390875	0.006309255	4.73E-07	9	Cybb
0.662770444	0.006387565	4.78E-07	9	Arhgap6
0.610362643	0.006465489	4.84E-07	9	BC003331
0.346118151	0.006579329	4.93E-07	9	Nsun6
0.527427562	0.006628773	4.96E-07	9	Ppp6r3
0.668868142	0.006631247	4.97E-07	9	Sh3pxd2b
0.670415397	0.006814578	5.10E-07	9	Mar.02
0.498088756	0.006843467	5.13E-07	9	Ywhab
0.929165722	0.006871219	5.15E-07	9	Relb
0.452395142	0.006947276	5.20E-07	9	Ino80
1.049780971	0.007027009	5.26E-07	9	Dda1
0.732940155	0.007087403	5.31E-07	9	Itpripl1
0.587387777	0.00729552	5.46E-07	9	Stx3
0.459213512	0.007318067	5.48E-07	9	Mkrn2
0.611366316	0.007480892	5.60E-07	9	Adss
0.602141368	0.007564383	5.67E-07	9	Cops6
1.541089994	0.007575298	5.67E-07	9	Net1
0.572793924	0.007909425	5.92E-07	9	1810011H11Rik
0.483059243	0.008026814	6.01E-07	9	Sema6b
0.517758459	0.008148826	6.10E-07	9	Tmem126a
0.589676541	0.008168178	6.12E-07	9	Dnm2
0.320633873	0.008235446	6.17E-07	9	Rpa3
0.52713515	0.008309521	6.22E-07	9	Becn1
0.659999981	0.008416585	6.30E-07	9	Pomp
0.484725696	0.008421596	6.31E-07	9	Rpp25l
0.658778423	0.008525669	6.39E-07	9	Adam9
0.763734222	0.008675363	6.50E-07	9	Cd80
0.449319837	0.008682754	6.50E-07	9	Fancl
0.578161952	0.008716299	6.53E-07	9	Mdh2
0.519378733	0.008859092	6.64E-07	9	Ptgs1
0.276665086	0.008944449	6.70E-07	9	Peli2
0.68027367	0.009126441	6.84E-07	9	Snx20
-1.745927765	0.009133338	6.84E-07	9	Ifi27l2a
0.977397445	0.009414264	7.05E-07	9	Exosc5
0.381417449	0.009599537	7.19E-07	9	AA987161
0.615916389	0.00964093	7.22E-07	9	Lars
0.424085591	0.009828367	7.36E-07	9	Kat7
0.639219518	0.009837081	7.37E-07	9	Lamtor3
0.298103281	0.009923311	7.43E-07	9	Vps11
0.468285785	0.010025376	7.51E-07	9	Gyg
0.72150133	0.010301554	7.72E-07	9	Rsu1
0.648475808	0.010425404	7.81E-07	9	Jmjd1c
-2.363702634	0.010476441	7.85E-07	9	Acp5
0.550992046	0.010746694	8.05E-07	9	Ubqln1
0.649428033	0.010876328	8.15E-07	9	Birc2
0.557158348	0.011152641	8.35E-07	9	Ndufa6
0.589805245	0.011526182	8.63E-07	9	Asap1
0.892955414	0.011534135	8.64E-07	9	Dyrk1a
0.29403589	0.011604984	8.69E-07	9	D10Jhu81e
0.481561806	0.011755039	8.80E-07	9	Lrrc8c
0.562620406	0.011856874	8.88E-07	9	Vash1
0.806840883	0.012504248	9.37E-07	9	Pold3
0.379660304	0.01256167	9.41E-07	9	Morc3
0.625406657	0.012625072	9.46E-07	9	Lrrfip1
0.483635778	0.012681685	9.50E-07	9	Cbx5
0.820775739	0.012742071	9.54E-07	9	Ash1l
0.605209542	0.012926621	9.68E-07	9	Prdx6
0.523851528	0.01295205	9.70E-07	9	Pink1

0.463285726	0.013003574	9.74E-07	9	Gga3
0.658951374	0.013188506	9.88E-07	9	Gm15421
0.436498923	0.013307026	9.97E-07	9	Abcb6
0.794931534	0.013584457	1.02E-06	9	Paics
0.283541143	0.013898993	1.04E-06	9	Bcl2
0.508874843	0.014295418	1.07E-06	9	Psmd2
0.616650896	0.01445969	1.08E-06	9	Pitrm1
0.552725803	0.014477396	1.08E-06	9	Pdgfb
-0.900142858	0.014534065	1.09E-06	9	Cltc
0.541226811	0.014647562	1.10E-06	9	Tbc1d23
0.500015698	0.014771509	1.11E-06	9	Tmcc1
0.434450463	0.015121459	1.13E-06	9	Atp5e
0.450821312	0.015586191	1.17E-06	9	Gss
0.646978906	0.015952265	1.19E-06	9	Bola3
0.694140303	0.016113708	1.21E-06	9	Ppp1r11
0.545708207	0.016507611	1.24E-06	9	P4hb
0.736980664	0.016557245	1.24E-06	9	Slc39a7
0.681144125	0.016583874	1.24E-06	9	Tbcb
0.559093066	0.016642242	1.25E-06	9	Hdgf
0.615676097	0.016689226	1.25E-06	9	Slc35f6
0.372553661	0.016712752	1.25E-06	9	Rps14
0.556619699	0.016837669	1.26E-06	9	Hspa9
1.197882323	0.016868585	1.26E-06	9	Rcc2
0.516620142	0.016945739	1.27E-06	9	Uqcr10
0.565243592	0.016963678	1.27E-06	9	Ascc3
-1.660813511	0.017062595	1.28E-06	9	Zeb2
0.551161178	0.017203809	1.29E-06	9	Smdt1
0.549087778	0.017596193	1.32E-06	9	Hipk1
0.699360898	0.017658097	1.32E-06	9	Slc25a11
0.492424897	0.017708115	1.33E-06	9	Galnt6
0.273376113	0.017725074	1.33E-06	9	Arhgef6
0.828780629	0.018080181	1.35E-06	9	Lrrc59
0.597446284	0.018423954	1.38E-06	9	Erp29
0.464928316	0.018941219	1.42E-06	9	Smc6
0.679286189	0.019511545	1.46E-06	9	Pik3cg
0.683046665	0.019513555	1.46E-06	9	Gm12338
0.573464513	0.020501065	1.54E-06	9	Ift20
0.588296259	0.020755017	1.55E-06	9	Irf5
0.569428272	0.020922159	1.57E-06	9	Mrpl57
0.396036809	0.021415011	1.60E-06	9	Gtf2e2
-1.710111286	0.021523274	1.61E-06	9	Cebpb
0.46400522	0.021562616	1.61E-06	9	1700123O20Rik
1.038162225	0.022907407	1.72E-06	9	Nek9
0.275593594	0.023199252	1.74E-06	9	Hlcs
0.411333903	0.023271552	1.74E-06	9	Dcun1d5
0.867865326	0.023348221	1.75E-06	9	Aptx
0.734238311	0.023403373	1.75E-06	9	Mesdc2
0.491575349	0.023476841	1.76E-06	9	Pgap2
-1.989419009	0.024286099	1.82E-06	9	Sec11a
0.36906767	0.02488923	1.86E-06	9	Nasp
0.506703642	0.025012998	1.87E-06	9	Ldha
0.609050763	0.025077228	1.88E-06	9	Cct4
0.537005229	0.02594854	1.94E-06	9	Nhlrc3
0.448405537	0.025994556	1.95E-06	9	Tcerg1
0.5442604	0.026194835	1.96E-06	9	Tmem18
0.612266027	0.027024036	2.02E-06	9	Alcam
1.305410993	0.027126948	2.03E-06	9	Aim1
0.41134194	0.027496123	2.06E-06	9	Mcm3
0.550249852	0.027789702	2.08E-06	9	Smc1a
0.590250549	0.028203842	2.11E-06	9	Zfp263
0.841856459	0.028304005	2.12E-06	9	Fam220a

0.615741202	0.028615256	2.14E-06	9	Ubxn2a
0.389199056	0.028755117	2.15E-06	9	Ufm1
0.523703909	0.029082991	2.18E-06	9	Ctnna1
0.33918963	0.029893655	2.24E-06	9	Appl1
0.715267996	0.030781839	2.31E-06	9	Ehmt2
1.229896179	0.030866645	2.31E-06	9	Papd7
0.543991619	0.030895737	2.31E-06	9	Batf
0.418790931	0.031320062	2.35E-06	9	Tmem173
0.562922199	0.031789477	2.38E-06	9	Sepn1
0.392648867	0.032114258	2.41E-06	9	1110057K04Rik
0.571795727	0.032278827	2.42E-06	9	Tmem147
0.952671018	0.032416017	2.43E-06	9	Wdr46
0.541479616	0.032686898	2.45E-06	9	Gabarapl1
0.312436682	0.032755566	2.45E-06	9	Msto1
0.736014954	0.032784574	2.46E-06	9	Igf2r
0.516549923	0.032797104	2.46E-06	9	Anapc5
0.576543905	0.032946534	2.47E-06	9	Arhgap1
0.575270637	0.03314649	2.48E-06	9	Lime1
0.587472401	0.033221787	2.49E-06	9	Cyb5r4
0.624014914	0.03325519	2.49E-06	9	Psmc2
0.754233488	0.033476935	2.51E-06	9	Eif3i
0.923905348	0.033565815	2.51E-06	9	Alkbh3
0.530140973	0.033642443	2.52E-06	9	Lats2
0.873685321	0.033822243	2.53E-06	9	Cdk12
0.652894241	0.033883809	2.54E-06	9	Agfg2
0.284917286	0.034016832	2.55E-06	9	Parp2
0.519567787	0.034079893	2.55E-06	9	Tmem29
0.442360835	0.03441884	2.58E-06	9	Dnttip2
0.542500308	0.034452473	2.58E-06	9	Ankrd17
0.74442853	0.034742714	2.60E-06	9	Pdlim7
0.289560579	0.035070185	2.63E-06	9	Ccnd2
0.63690062	0.035724588	2.68E-06	9	Spred1
0.697521162	0.035788612	2.68E-06	9	B430306N03Rik
0.729571495	0.036326664	2.72E-06	9	Fam198b
0.370272933	0.036436575	2.73E-06	9	Gtf2h2
0.666711024	0.036588841	2.74E-06	9	Clptm1l
0.430659284	0.036914323	2.76E-06	9	Cbx7
0.493412497	0.037029206	2.77E-06	9	Tada1
0.374842248	0.038305993	2.87E-06	9	Ncdn
0.602947866	0.03850927	2.88E-06	9	Ubtd1
0.478816492	0.038720653	2.90E-06	9	Slc38a1
0.410090935	0.039386292	2.95E-06	9	Ephx1
0.591585237	0.0395619	2.96E-06	9	Slc25a4
0.615289702	0.039694765	2.97E-06	9	Txndc12
0.43619169	0.039924443	2.99E-06	9	Rbbp4
0.638497822	0.041344023	3.10E-06	9	Gtf2h5
0.576135608	0.041730114	3.13E-06	9	Nifk
0.444202379	0.041864909	3.14E-06	9	Pold2
0.564188696	0.041917271	3.14E-06	9	2810474O19Rik
-1.888621694	0.042769434	3.20E-06	9	Yipf3
0.541185422	0.043573197	3.26E-06	9	Bag1
1.155263716	0.043678042	3.27E-06	9	Spint1
0.338489098	0.043689653	3.27E-06	9	Cenpj
-1.313177227	0.044060428	3.30E-06	9	Tgif1
0.493327215	0.044141898	3.31E-06	9	Scamp2
0.256578327	0.044264087	3.32E-06	9	Ercc8
0.28534462	0.04428578	3.32E-06	9	Lnx2
0.583859973	0.044726965	3.35E-06	9	Spata5
0.397055157	0.044964701	3.37E-06	9	Rpf2
0.492519177	0.045123946	3.38E-06	9	Lsp1
0.549824919	0.04528369	3.39E-06	9	2010107E04Rik

1.137272709	0.045630079	3.42E-06	9	Cct5
1.526569412	0.046706595	3.50E-06	9	Siah2
0.500141095	0.046772679	3.50E-06	9	Msantd4
0.473879603	0.047179242	3.53E-06	9	Lypla1
0.537396257	0.047265901	3.54E-06	9	Pycard
0.358787249	0.047441814	3.55E-06	9	Ctsf
0.715630241	0.047701961	3.57E-06	9	Sp140
0.538073902	0.050207469	3.76E-06	9	Il18rap
-2.006702716	0.050253597	3.76E-06	9	Emb
0.504407536	0.050444769	3.78E-06	9	Hnrnpa2b1
-1.112312869	0.050457541	3.78E-06	9	Gm1821
0.522152102	0.050602923	3.79E-06	9	Alg5
0.455268758	0.050915246	3.81E-06	9	Glyr1
0.508963847	0.051304123	3.84E-06	9	Exosc8
0.430672949	0.052156789	3.91E-06	9	Cast
0.803902384	0.052436028	3.93E-06	9	Metap2
0.398972407	0.054384259	4.07E-06	9	Rinl
0.530628251	0.054872445	4.11E-06	9	Coro7
0.544493202	0.055353154	4.15E-06	9	Ppil2
0.615317402	0.055425492	4.15E-06	9	Timm13
0.726779605	0.055595316	4.16E-06	9	Cisd2
0.257796602	0.055932247	4.19E-06	9	Fam210b
0.496609921	0.056794766	4.25E-06	9	Sbf2
0.551402633	0.058135608	4.35E-06	9	Oxct1
0.64947625	0.058672077	4.39E-06	9	Pilra
0.359338061	0.05896005	4.42E-06	9	Rpph1
0.47587012	0.059475332	4.45E-06	9	Msh6
-0.436182684	0.061036349	4.57E-06	9	B2m
0.348471732	0.061265946	4.59E-06	9	Abhd8
0.479890682	0.061298514	4.59E-06	9	Apc
0.392846698	0.061886556	4.64E-06	9	Dbndd2
1.016786588	0.062201218	4.66E-06	9	Mob2
0.541410583	0.062969291	4.72E-06	9	Git2
0.60074086	0.063183463	4.73E-06	9	Tmx1
0.549016216	0.064549858	4.83E-06	9	Txndc17
0.522287131	0.064736474	4.85E-06	9	Ankrd13a
0.60332082	0.064915687	4.86E-06	9	Fech
0.627552918	0.0661052	4.95E-06	9	Spdl1
0.676637646	0.06696416	5.02E-06	9	Plec
0.54010239	0.06798827	5.09E-06	9	Pign
0.493139859	0.068134541	5.10E-06	9	Tmem109
0.505116459	0.068738126	5.15E-06	9	Eif4ebp3
0.494337091	0.069307229	5.19E-06	9	Tmem43
0.501140394	0.069820269	5.23E-06	9	Etf1
0.257070575	0.070288094	5.26E-06	9	Smad1
0.658945273	0.071177726	5.33E-06	9	Cd86
0.480424837	0.071744612	5.37E-06	9	Oxa1l
0.437115762	0.072015581	5.39E-06	9	Nt5e
0.490885939	0.072495926	5.43E-06	9	Arhgap25
1.112620778	0.072540052	5.43E-06	9	Snx30
2.008567491	0.074471011	5.58E-06	9	Wdr48
0.470402368	0.074957991	5.61E-06	9	Gdpd1
0.71553212	0.075231029	5.63E-06	9	Echdc1
0.552790266	0.075299091	5.64E-06	9	Acox3
0.588568025	0.076355871	5.72E-06	9	Rftn1
0.278624225	0.076565452	5.73E-06	9	Tmem219
1.222275541	0.07758283	5.81E-06	9	Sdad1
0.626664033	0.078430415	5.87E-06	9	Tmem128
0.705665867	0.081442709	6.10E-06	9	Brpf1
0.322896208	0.082401491	6.17E-06	9	Klc1
-0.34270848	0.082835558	6.20E-06	9	Fth1

0.731273797	0.082998921	6.22E-06	9	Napa
0.602420239	0.083513037	6.25E-06	9	Nr1h3
0.388203142	0.083552924	6.26E-06	9	Lage3
0.829905812	0.083683878	6.27E-06	9	Insig2
0.710333885	0.083717824	6.27E-06	9	Ndufc2
0.62339772	0.083774536	6.27E-06	9	Ssu72
0.559848436	0.086454249	6.48E-06	9	Tmx3
-0.986993502	0.090686071	6.79E-06	9	Mir6236
0.716921049	0.091642415	6.86E-06	9	Uqcrc2
0.451599805	0.091903839	6.88E-06	9	Snx12
0.4965932	0.093042671	6.97E-06	9	Nsfl1c
0.331986521	0.093970116	7.04E-06	9	Hectd1
0.30276771	0.0943713	7.07E-06	9	Tomm5
1.299760811	0.094902021	7.11E-06	9	Hyou1
0.441810166	0.095826275	7.18E-06	9	Sdf2
0.506411571	0.095846419	7.18E-06	9	Agpat5
0.393540255	0.096241901	7.21E-06	9	Cyb561d2
0.413059688	0.096608688	7.24E-06	9	Hnrnpu
0.450845955	0.097175968	7.28E-06	9	Nqo2
0.653956147	0.097349315	7.29E-06	9	Samsn1
0.443374165	0.097638355	7.31E-06	9	Rptor
-1.175649424	0.097787579	7.32E-06	9	Ctsc
0.400964088	0.098727296	7.39E-06	9	Gdi2
0.257090754	0.098844843	7.40E-06	9	Prmt7
0.57872887	0.099052822	7.42E-06	9	Gcsh
0.903854043	0.099539108	7.45E-06	9	Pcif1
0.555511731	0.099577443	7.46E-06	9	Csf2rb
0.503685349	0.102539673	7.68E-06	9	Kdm5c
0.345537859	0.102923541	7.71E-06	9	Ccdc9
0.2961779	0.105460511	7.90E-06	9	Tamm41
0.376021559	0.105551289	7.91E-06	9	Wipi1
0.826362546	0.10562759	7.91E-06	9	Camta1
0.687997758	0.105983476	7.94E-06	9	Aup1
0.878718537	0.106474358	7.97E-06	9	Prpf4b
-2.266647187	0.106579456	7.98E-06	9	Ccr2
0.538599794	0.107196045	8.03E-06	9	Cox17
0.440474758	0.108313888	8.11E-06	9	Bclaf1
0.422666384	0.109253249	8.18E-06	9	Fam213a
-2.313406408	0.109364644	8.19E-06	9	AW112010
0.365937628	0.109876848	8.23E-06	9	Zc3h15
0.429657017	0.110661958	8.29E-06	9	Prpf8
0.455891249	0.111958178	8.39E-06	9	Txnl4a
0.317789058	0.112054975	8.39E-06	9	Tyms-ps
0.56340187	0.112151515	8.40E-06	9	Slamf7
0.381413173	0.112425602	8.42E-06	9	Ttyh2
0.387515685	0.112644559	8.44E-06	9	Dpy19l4
0.453682524	0.113895236	8.53E-06	9	Hepacam2
0.49312679	0.115297138	8.64E-06	9	Cdk11b
0.578687742	0.116935927	8.76E-06	9	Usp34
-1.931297127	0.117987208	8.84E-06	9	Prpsap1
0.583177111	0.118213035	8.85E-06	9	S100a4
0.433424249	0.118232169	8.86E-06	9	Casp3
0.543341704	0.11897014	8.91E-06	9	Slc25a33
0.54280995	0.119758632	8.97E-06	9	Pip5k1c
0.416649275	0.120656303	9.04E-06	9	Rapsn
0.859754347	0.121496337	9.10E-06	9	Nfkbib
0.534152195	0.122116266	9.15E-06	9	Srrt
0.479508278	0.123327438	9.24E-06	9	Mgst3
0.534788935	0.1243008	9.31E-06	9	Dctn1
0.484690519	0.126802455	9.50E-06	9	Dnajc18
0.555032497	0.128144642	9.60E-06	9	Mcu

0.583582813	0.12822865	9.60E-06	9	Hexim1
0.59632935	0.129049635	9.67E-06	9	Inpp5d
0.433790011	0.129303217	9.68E-06	9	Exosc1
0.766509169	0.130170526	9.75E-06	9	Mrps24
0.265256426	0.131567635	9.85E-06	9	Slc8b1
0.800049109	0.132031098	9.89E-06	9	Stard3nl
0.423616132	0.132776359	9.94E-06	9	Smim3
0.629884814	0.132845129	9.95E-06	9	Mrpl21
0.294571724	0.132861035	9.95E-06	9	Prnp
0.399962016	0.133378853	9.99E-06	9	Smad2
0.647974437	0.133497823	1.00E-05	9	Taf4b
0.891746157	0.135161092	1.01E-05	9	Gpr132
0.863756341	0.136345311	1.02E-05	9	Timm22
0.913379535	0.137074764	1.03E-05	9	Ccdc22
0.469966433	0.13859931	1.04E-05	9	Lactb
0.622133909	0.140008085	1.05E-05	9	Mfsd6
0.498482553	0.142954612	1.07E-05	9	Golga4
0.511582074	0.143709009	1.08E-05	9	Klf3
1.438549197	0.14444392	1.08E-05	9	Usp31
0.473210259	0.144542402	1.08E-05	9	Pde8a
0.391409265	0.145471448	1.09E-05	9	Sf3b3
-1.970519638	0.145523112	1.09E-05	9	Rassf1
0.648753236	0.145985107	1.09E-05	9	Cd276
0.494123434	0.146841198	1.10E-05	9	Mapkap1
0.269710292	0.147451748	1.10E-05	9	Ttc13
0.34295859	0.148461377	1.11E-05	9	Tmem167
0.539641454	0.150143908	1.12E-05	9	Necap1
0.315852949	0.151665075	1.14E-05	9	Nup214
0.693042228	0.153263221	1.15E-05	9	Fbxo34
0.611896125	0.154369934	1.16E-05	9	Creb3
0.427600329	0.156251498	1.17E-05	9	Sh3bp5
0.253089355	0.157161943	1.18E-05	9	Tmcc3
0.531006346	0.157298333	1.18E-05	9	Fnbp1
0.611978574	0.164287118	1.23E-05	9	Tlr8
0.614830144	0.166278163	1.25E-05	9	Polr3e
0.60229904	0.166563769	1.25E-05	9	Gch1
0.395629277	0.166794106	1.25E-05	9	Dynlt3
0.648201469	0.170241162	1.28E-05	9	Mrpl43
0.404267217	0.170625581	1.28E-05	9	Pfdn4
0.565774448	0.170781271	1.28E-05	9	Wdr74
0.450866677	0.171046796	1.28E-05	9	Tmem160
0.757791211	0.171591388	1.29E-05	9	Ddx21
0.506823122	0.174379849	1.31E-05	9	Hspa4
0.582596549	0.177435759	1.33E-05	9	Msrb2
0.442635846	0.178037037	1.33E-05	9	Ethe1
0.260056592	0.178096277	1.33E-05	9	Fbxw4
0.516339804	0.178634709	1.34E-05	9	Mrps34
0.530094395	0.179032463	1.34E-05	9	Plcg2
0.596015481	0.183600646	1.38E-05	9	9530068E07Rik
0.636870482	0.183730534	1.38E-05	9	Cnn2
0.337685275	0.185236659	1.39E-05	9	Socs4
0.572504728	0.185537031	1.39E-05	9	Ndufb6
-1.875297215	0.186051637	1.39E-05	9	Vwa5a
0.449417969	0.189133697	1.42E-05	9	Apbb1ip
0.519851435	0.189625029	1.42E-05	9	Ccdc130
0.498277328	0.192008662	1.44E-05	9	Sin3b
0.399998099	0.193511783	1.45E-05	9	Dera
0.423015014	0.196170797	1.47E-05	9	Kdm2a
0.662882714	0.196992395	1.48E-05	9	Cog4
0.313563746	0.197204352	1.48E-05	9	Arpc5l
0.545302609	0.198602876	1.49E-05	9	Arsb

0.391209225	0.199411661	1.49E-05	9	Cisd3
0.734723114	0.199450717	1.49E-05	9	Ppwd1
0.514345126	0.20086678	1.50E-05	9	Mphosph10
0.4483822	0.201473268	1.51E-05	9	Pbdc1
0.565295854	0.20213535	1.51E-05	9	Eif3f
0.448754314	0.20270731	1.52E-05	9	Mogs
0.636092562	0.204251099	1.53E-05	9	Tbc1d22a
-1.840547974	0.205407149	1.54E-05	9	Man1c1
0.294719377	0.205586906	1.54E-05	9	Alkbh7
0.467896658	0.209774143	1.57E-05	9	Abcc3
0.778364967	0.211257399	1.58E-05	9	Slc35e1
0.365009117	0.213420026	1.60E-05	9	Psip1
-1.338269724	0.216578284	1.62E-05	9	Ccl4
0.278365682	0.216711018	1.62E-05	9	Itpk1
0.623724571	0.219835197	1.65E-05	9	Mrpl18
0.564600084	0.221910506	1.66E-05	9	Iars
-1.949289346	0.224272109	1.68E-05	9	Rps6ka1
0.499208951	0.22433134	1.68E-05	9	Sec22b
1.919152129	0.229632337	1.72E-05	9	Lin54
0.503917562	0.235853768	1.77E-05	9	Sin3a
0.439756415	0.23605329	1.77E-05	9	Olfml3
0.531182934	0.240010404	1.80E-05	9	Rab31
-2.039508424	0.242897339	1.82E-05	9	Tnfsf9
-1.731266998	0.246097651	1.84E-05	9	Entpd1
0.536626775	0.246593417	1.85E-05	9	Slc7a5
-1.823463995	0.246666611	1.85E-05	9	Shoc2
0.398061153	0.248601858	1.86E-05	9	Usp14
0.463231985	0.248628553	1.86E-05	9	Mrpl52
0.387561948	0.249094654	1.87E-05	9	Zmynd15
0.430797874	0.250733663	1.88E-05	9	Dlgap4
0.334931012	0.250801973	1.88E-05	9	Smc4
0.403526855	0.251734892	1.89E-05	9	Caprin1
0.664977423	0.257194667	1.93E-05	9	Csrp2
0.305144805	0.258943203	1.94E-05	9	Arpc2
0.37394641	0.258996839	1.94E-05	9	Wdr82
-1.96398293	0.262657015	1.97E-05	9	1810043H04Rik
0.32176641	0.262741074	1.97E-05	9	Lmnb1
0.512924248	0.266201653	1.99E-05	9	Slc39a10
0.895116134	0.270016879	2.02E-05	9	Hivep1
0.397778469	0.270402972	2.03E-05	9	Vkorc1
-1.761853812	0.270592487	2.03E-05	9	Psen1
0.606568295	0.274396899	2.06E-05	9	Krtcap2
0.532152127	0.275718017	2.06E-05	9	Nhp2
0.428415819	0.278780192	2.09E-05	9	Frmd4a
0.562081296	0.27970429	2.09E-05	9	Ctbp2
0.294209396	0.285420729	2.14E-05	9	Lrrfip2
0.353361948	0.29159456	2.18E-05	9	Igf1r
0.408081001	0.291612729	2.18E-05	9	Myo1g
0.352480788	0.294915427	2.21E-05	9	Fen1
1.123850931	0.295564994	2.21E-05	9	Fam178a
0.484219515	0.295888153	2.22E-05	9	Sbds
0.756695937	0.296076214	2.22E-05	9	Gclm
0.325119856	0.299280799	2.24E-05	9	Il21r
0.648534386	0.302406827	2.26E-05	9	Malt1
0.455101859	0.304787368	2.28E-05	9	Pfkp
0.388116496	0.306770483	2.30E-05	9	Rap2c
-1.973495369	0.306856555	2.30E-05	9	Msrb1
0.300497561	0.307369037	2.30E-05	9	Ctdspl2
0.681624761	0.311305118	2.33E-05	9	Tceb3
1.111185676	0.317628954	2.38E-05	9	Rfc2
0.593978729	0.317721938	2.38E-05	9	Dnaja2

0.580825069	0.317877408	2.38E-05	9	Cd2ap
0.514287745	0.319034111	2.39E-05	9	Flot1
0.329732441	0.320590522	2.40E-05	9	Lrrc58
0.51714639	0.323010763	2.42E-05	9	Ccdc12
0.329328526	0.326774611	2.45E-05	9	Phf3
-1.054445978	0.326951115	2.45E-05	9	Sirpa
-1.946230985	0.32999014	2.47E-05	9	Atp6v0d2
0.422193089	0.330353719	2.47E-05	9	Cd300ld
0.365040495	0.337945121	2.53E-05	9	Ptplad2
0.598821759	0.339664796	2.54E-05	9	U2surp
0.359149861	0.344241826	2.58E-05	9	Dtymk
0.49784007	0.346262624	2.59E-05	9	Fam193b
0.356502631	0.349127568	2.61E-05	9	Mars
0.509403684	0.350805991	2.63E-05	9	Coro1b
0.375363468	0.352797891	2.64E-05	9	Cs
-1.726056765	0.355278161	2.66E-05	9	Rab5c
0.504897803	0.363781313	2.72E-05	9	Tnip1
-1.891694435	0.365245639	2.74E-05	9	Lgals8
0.259222666	0.368122407	2.76E-05	9	Cdca8
0.446196313	0.372089059	2.79E-05	9	Ncf1
-1.865059264	0.377985682	2.83E-05	9	Bag3
0.494249979	0.378621278	2.84E-05	9	Lsr
0.379931044	0.38001988	2.85E-05	9	Sertad1
0.473801936	0.390944801	2.93E-05	9	Mrpl10
0.312419151	0.39667388	2.97E-05	9	Atmin
0.478847535	0.401458846	3.01E-05	9	Taldo1
0.488379941	0.402801058	3.02E-05	9	Gipc1
-1.482333277	0.406023849	3.04E-05	9	Glul
0.610590942	0.41368634	3.10E-05	9	Zcrb1
0.594723774	0.415057694	3.11E-05	9	Pmepa1
-1.785128896	0.418712961	3.14E-05	9	Ttc3
-0.821542417	0.426191886	3.19E-05	9	Sat1
0.923535176	0.43319729	3.24E-05	9	Sugp1
0.465813556	0.433884993	3.25E-05	9	Os9
0.527442153	0.43393361	3.25E-05	9	Glt25d1
0.314368834	0.434618398	3.26E-05	9	Map3k6
0.560936897	0.437222102	3.27E-05	9	Mrpl20
-1.75205508	0.439341322	3.29E-05	9	Ankrd12
-1.670204986	0.439785389	3.29E-05	9	Jun
0.902154685	0.439871995	3.29E-05	9	Usp25
0.506839408	0.443112351	3.32E-05	9	Camk1d
0.807609507	0.443400199	3.32E-05	9	Tmem192
0.398816026	0.44570686	3.34E-05	9	Rwdd4a
0.637409455	0.446014109	3.34E-05	9	Zfp326
0.493025518	0.446784036	3.35E-05	9	Afg3l2
0.584161617	0.4500563	3.37E-05	9	Bach2
0.605385948	0.458489015	3.43E-05	9	Dis3
0.589752898	0.458963649	3.44E-05	9	Unc50
0.696276607	0.461463909	3.46E-05	9	Hbp1
0.346047318	0.468358022	3.51E-05	9	Tagln2
1.04753307	0.475315193	3.56E-05	9	1110059G10Rik
0.387218152	0.475539019	3.56E-05	9	Map2k3os
0.318660851	0.476123812	3.57E-05	9	Mrps6
0.396883481	0.479096988	3.59E-05	9	Emc10
0.474650435	0.481697826	3.61E-05	9	Ahi1
0.462370287	0.482141463	3.61E-05	9	Pdhb
0.429444725	0.491834274	3.68E-05	9	Ppp1r35
0.532425942	0.492058851	3.69E-05	9	Brca2
0.350157669	0.494310659	3.70E-05	9	Acap3
0.944040322	0.50020111	3.75E-05	9	Clcn4-2
0.452278949	0.501265519	3.75E-05	9	Akap9

-1.815457386		0.518683057	3.88E-05	9	Mea1
0.55986953		0.519041808	3.89E-05	9	Cdk2ap2
0.361721853		0.521864733	3.91E-05	9	Dcaf8
0.782366634		0.528207454	3.96E-05	9	Ech1
-1.748361431		0.536973867	4.02E-05	9	Snx8
0.500329875		0.539869784	4.04E-05	9	Ivns1abp
0.535822438		0.550237996	4.12E-05	9	Nrros
0.65645765		0.55041653	4.12E-05	9	Cdkn2aipnl
0.445169497		0.556396338	4.17E-05	9	Ktn1
0.412721166		0.565497839	4.24E-05	9	Rnpep
1.026358734		0.567601595	4.25E-05	9	Ccnyl1
0.295023479		0.569857207	4.27E-05	9	Mrpl40
-0.660211329		0.570448201	4.27E-05	9	Ctsh
0.406387581		0.57315736	4.29E-05	9	Pld4
0.429850049		0.575034399	4.31E-05	9	Thoc6
0.307871622		0.577012081	4.32E-05	9	Rrm1
0.387925518		0.578518392	4.33E-05	9	Bin2
0.461109453		0.587730515	4.40E-05	9	Adcy7
0.537627355		0.600693281	4.50E-05	9	Trappc2l
-0.733989535		0.606928672	4.55E-05	9	Lgmn
0.464217692		0.60797492	4.55E-05	9	Vgll4
-1.379337819		0.61143013	4.58E-05	9	Mafb
0.600745186		0.6137683	4.60E-05	9	Dynll2
0.585034831		0.614034613	4.60E-05	9	Diap2
0.6845891		0.619220146	4.64E-05	9	Isca2
0.290175569		0.620624031	4.65E-05	9	Ell2
0.453647832		0.621707837	4.66E-05	9	Hint1
0.263110029		0.625517673	4.68E-05	9	Mrps26
0.617382665		0.636243924	4.77E-05	9	Med20
0.489849689		0.638314225	4.78E-05	9	Tppp3
0.422780502		0.63844672	4.78E-05	9	Cpne8
0.717771327		0.656060134	4.91E-05	9	Ctdsp1
0.509440635		0.658307869	4.93E-05	9	Prkaa1
-1.664624328		0.663178366	4.97E-05	9	Zfp36l2
0.257881259		0.667493904	5.00E-05	9	Fam160b1
0.661490491		0.670037487	5.02E-05	9	Whamm
0.518247717		0.674369484	5.05E-05	9	Pmp22
-1.734479423		0.691736588	5.18E-05	9	Herc2
0.281741683		0.699598058	5.24E-05	9	Tmem30a
0.429526147		0.700413544	5.25E-05	9	Ube2b
0.333695631		0.703279936	5.27E-05	9	Cdan1
0.363574166		0.708873447	5.31E-05	9	Msi2
0.405858077		0.708922527	5.31E-05	9	Slc39a14
0.400688437		0.708997944	5.31E-05	9	Sugt1
-1.959563409		0.712916192	5.34E-05	9	Pttg1
0.405398739		0.713622662	5.34E-05	9	Jak2
0.383400787		0.736540889	5.52E-05	9	Mgat4b
0.494868872		0.741773127	5.56E-05	9	Polr2g
0.565505508		0.745769468	5.59E-05	9	Timm9
0.602976488		0.750681644	5.62E-05	9	Cdipt
0.428021766		0.753095399	5.64E-05	9	Hsdl2
0.649741173		0.776245567	5.81E-05	9	Ppt1
0.316786985		0.776935136	5.82E-05	9	2310009A05Rik
0.584648461		0.777936471	5.83E-05	9	Ecd
-1.794692337		0.779871122	5.84E-05	9	Mrpl36
0.437115762		0.780985785	5.85E-05	9	Wdr55
0.562880068		0.78770807	5.90E-05	9	Ptpre
0.286467713		0.796641113	5.97E-05	9	Tmem97
0.300569171		0.801634882	6.00E-05	9	Meaf6
0.449227904		0.816900545	6.12E-05	9	Galns
0.391321107		0.816902569	6.12E-05	9	Rab10

0.444069158	0.821931046	6.16E-05	9	1110007C09Rik
0.721688884	0.822702414	6.16E-05	9	Batf3
0.520862046	0.824407747	6.17E-05	9	Cbfa2t2
0.316243684	0.827448313	6.20E-05	9	Zbtb44
0.296017814	0.833244722	6.24E-05	9	Chrac1
0.252108047	0.847329709	6.35E-05	9	Trappc1
-2.026737478	0.853269928	6.39E-05	9	Hpgd
0.448783122	0.857069746	6.42E-05	9	Zc3h14
0.29686248	0.862647279	6.46E-05	9	Srsf1
0.518578901	0.869248505	6.51E-05	9	Mkks
0.537055016	0.870470953	6.52E-05	9	Gpr89
0.438502967	0.875617017	6.56E-05	9	Cox6a1
0.608467301	0.875976006	6.56E-05	9	Git1
0.290078997	0.880647864	6.60E-05	9	Gbf1
0.500629853	0.880661766	6.60E-05	9	Exosc10
0.443771064	0.891623891	6.68E-05	9	Chd2
0.273351275	0.892416534	6.68E-05	9	Bms1
0.34625922	0.893386812	6.69E-05	9	Rab6a
0.430601081	0.907084741	6.79E-05	9	Fto
0.515536611	0.934256583	7.00E-05	9	Tle4
0.440932792	0.9374739	7.02E-05	9	Rac2
0.257417606	0.93930601	7.03E-05	9	St6gal1
0.292434541	0.948348842	7.10E-05	9	Sae1
0.378615878	0.950427856	7.12E-05	9	Sf3b5
0.551502576	0.965066624	7.23E-05	9	Mrpl55
0.259808628	0.974395208	7.30E-05	9	Ubash3b
0.490707291	0.977669697	7.32E-05	9	Wbscr22
0.262522684	0.984825229	7.38E-05	9	Casp7
-1.90832391	0.989132198	7.41E-05	9	Rpain
-1.723129201	1	7.50E-05	9	Ankfy1
0.386694649	1	7.54E-05	9	Cdc34
0.354576411	1	7.62E-05	9	Pkd2
0.25452206	1	7.64E-05	9	Klhdc3
0.295188312	1	7.71E-05	9	Rps15
-1.809329956	1	7.82E-05	9	Chmp2b
0.422913886	1	7.84E-05	9	Tm2d2
0.287359752	1	7.99E-05	9	Mapk8ip3
-1.939719895	1	8.00E-05	9	Mrpl34
0.303698375	1	8.05E-05	9	Rexo4
0.254239859	1	8.20E-05	9	Dbnl
0.509349753	1	8.36E-05	9	Rhoc
-1.751495874	1	8.43E-05	9	Tmem86a
0.517564865	1	8.47E-05	9	Psma1
-1.735445911	1	8.48E-05	9	Galc
0.252902432	1	8.60E-05	9	Crem
0.48616953	1	8.68E-05	9	Lmna
0.538121645	1	8.80E-05	9	Prkd3
0.533603105	1	8.89E-05	9	Rps27a
0.356811319	1	9.14E-05	9	Larp1b
0.413306421	1	9.15E-05	9	Huwe1
0.258044245	1	9.18E-05	9	Ddx27
0.255679451	1	9.23E-05	9	L1cam
1.034334511	1	9.25E-05	9	Hook3
1.085757527	1	9.26E-05	9	Ier5l
0.369737536	1	9.26E-05	9	Grwd1
0.385977483	1	9.27E-05	9	Abca7
0.2712965	1	9.27E-05	9	Cks1b
0.364676812	1	9.29E-05	9	Fxr1
0.418019407	1	9.34E-05	9	Otulin
0.313430056	1	9.52E-05	9	Ccl7
-1.537645209	1	9.69E-05	9	Smarcc2

0.489692051	1	9.71E-05	9	Dpep2
0.398363645	1	9.94E-05	9	Snx6
0.408128226	1	9.95E-05	9	Lilra6
0.288898128	1	9.97E-05	9	Ddx49
0.37536581	1	0.000101	9	Elovl5
0.382896416	1	0.0001021	9	Srfbp1
0.486223102	1	0.0001032	9	Cdt1
0.296424236	1	0.0001055	9	Ndufaf4
0.356688314	1	0.0001064	9	Pcm1
0.262276349	1	0.0001067	9	Mrpl45
0.73923904	1	0.000111	9	Rsl1d1
0.737951077	1	0.0001112	9	Ndufb8
0.37086446	1	0.0001127	9	Gng2
0.258433861	1	0.0001141	9	Tbc1d8
-1.790538313	1	0.0001148	9	Fam214b
0.262276349	1	0.0001162	9	Uchl3
0.264421505	1	0.0001164	9	Ccdc132
0.427884139	1	0.0001168	9	Ppp2ca
-1.688438566	1	0.0001172	9	Ifi204
-1.340020668	1	0.0001177	9	Lmo2
-1.484093418	1	0.0001185	9	Atraid
-1.66712273	1	0.0001188	9	Tgfbrap1
0.377457183	1	0.0001193	9	Rps19bp1
0.354710566	1	0.0001223	9	Slc35b1
0.272472319	1	0.0001232	9	Sec14l1
-1.604853759	1	0.0001239	9	Prex1
0.338997274	1	0.000124	9	Fkbp3
0.467450454	1	0.0001247	9	Syvn1
0.443491221	1	0.0001247	9	Minos1
0.381721816	1	0.0001248	9	Rala
0.522044159	1	0.0001254	9	Ten1
0.490353654	1	0.000126	9	Plcb2
0.425431667	1	0.0001263	9	Vapa
0.259975564	1	0.0001269	9	Ch25h
-1.84814847	1	0.000128	9	Fam46a
-1.722574142	1	0.0001291	9	Mrps18a
0.264501044	1	0.0001291	9	Med4
-1.790146305	1	0.0001296	9	Raph1
0.532860974	1	0.0001305	9	Ndufs8
0.489162416	1	0.0001317	9	Usp7
-1.721283421	1	0.0001323	9	Pacsin2
0.401961711	1	0.0001334	9	Mecr
0.382583171	1	0.0001339	9	Pcmt1
0.524292649	1	0.0001341	9	Map1lc3a
0.459143085	1	0.0001349	9	Nkiras2
0.360540371	1	0.0001354	9	Anapc16
0.483877879	1	0.0001358	9	Edem3
0.348714724	1	0.0001362	9	S100a10
-0.60872866	1	0.0001366	9	Tpm3
0.356813113	1	0.000137	9	Yif1b
-1.744394788	1	0.0001373	9	B230219D22Rik
0.507316885	1	0.0001376	9	Snhg6
0.640865177	1	0.0001378	9	Nek6
0.274399224	1	0.0001382	9	1110058L19Rik
0.335251359	1	0.0001386	9	Dtd1
-1.411022884	1	0.0001387	9	Cd164
0.284075577	1	0.000139	9	Dgka
-1.798521492	1	0.0001391	9	Mgat1
-1.751057917	1	0.0001409	9	Rassf4
0.334893997	1	0.0001414	9	Emilin2
0.559629474	1	0.0001416	9	Chd9

-1.814501875	1	0.0001427	9	Lacc1
0.445618834	1	0.000143	9	Xiap
0.925947948	1	0.0001435	9	Ttc4
0.408594535	1	0.0001444	9	Yrdc
0.442445318	1	0.0001444	9	Tbrg4
0.423625229	1	0.000146	9	Ms4a6d
0.573011576	1	0.0001473	9	Hras
0.615063967	1	0.0001476	9	Eif3g
-1.741654252	1	0.0001479	9	Sidt2
0.401333182	1	0.0001479	9	Snx24
0.544462686	1	0.0001481	9	Vcpip1
0.304425248	1	0.0001505	9	Galk2
0.635577733	1	0.000152	9	Pld2
0.286584865	1	0.0001526	9	Utp3
0.337068315	1	0.0001548	9	Dgcr2
0.39102589	1	0.0001552	9	Parn
0.865506015	1	0.0001556	9	Runx3
0.302606107	1	0.0001562	9	Spty2d1
0.407712727	1	0.0001566	9	Hsd17b12
0.485391499	1	0.0001577	9	Rab3il1
0.409009671	1	0.0001578	9	Zmat5
0.380540269	1	0.0001587	9	Kcmf1
0.422288618	1	0.0001603	9	Bud31
0.890502428	1	0.0001629	9	Sms
0.359819178	1	0.0001636	9	Tpm4
0.465608835	1	0.0001653	9	Usp42
-1.569733523	1	0.0001668	9	Immt
0.513966358	1	0.0001702	9	Anxa3
-1.690581041	1	0.0001703	9	Mrpl15
0.359302285	1	0.0001708	9	Thumpd3
-1.82029295	1	0.0001717	9	Rgl1
0.301889996	1	0.0001737	9	Txn2
0.543643306	1	0.0001743	9	Phf6
0.672534374	1	0.0001746	9	Ndufb10
-1.820308934	1	0.0001752	9	Bcl2l11
0.389349052	1	0.0001756	9	Psmb4
-1.900671284	1	0.0001763	9	Cd38
0.285653772	1	0.0001811	9	Rpl41
0.401016783	1	0.0001819	9	Eea1
-1.631910768	1	0.0001831	9	Mbd3
0.371139656	1	0.0001843	9	1110004F10Rik
-1.726317897	1	0.0001848	9	Triap1
0.364047436	1	0.000186	9	Dpy30
0.464998942	1	0.0001863	9	Fmr1
0.259143089	1	0.000187	9	Myo1c
0.314343722	1	0.0001874	9	Stmn1
0.367690672	1	0.0001881	9	Mef2a
-1.526557352	1	0.0001914	9	Nucb1
0.263487736	1	0.0001952	9	Mlec
0.35536123	1	0.0001988	9	Mien1
0.341838113	1	0.0001991	9	Mgl2
0.380755115	1	0.0001995	9	Itga5
-0.787638555	1	0.0002003	9	Ier3
0.563303181	1	0.000201	9	Hsd17b10
-0.835420611	1	0.0002039	9	Atp2b1
0.36668213	1	0.0002052	9	Strap
0.330655415	1	0.0002068	9	Cd37
-1.826567283	1	0.0002076	9	Cep350
0.309014727	1	0.0002086	9	Ppp2r2d
0.338470863	1	0.0002111	9	Agl
0.378605168	1	0.0002118	9	Tmem208

0.449429434	1	0.000212	9	Cd84
0.36698493	1	0.0002135	9	Tuba4a
0.423307789	1	0.0002155	9	Mrps2
0.250061948	1	0.0002184	9	Larp4b
0.961050657	1	0.0002203	9	Fdft1
1.787073037	1	0.0002208	9	Siglech
-1.635935477	1	0.000221	9	Dgkz
0.400748118	1	0.0002227	9	Ercc3
0.287132996	1	0.0002231	9	Chmp1b
-1.836365398	1	0.0002233	9	Osm
0.48529082	1	0.0002239	9	Qdpr
-1.62343183	1	0.0002243	9	Elf4
0.282767043	1	0.0002282	9	Cnih4
-1.524417402	1	0.0002291	9	Hint2
-1.657854163	1	0.0002315	9	Cyp4f18
0.813593334	1	0.0002347	9	Ddit3
-1.58782948	1	0.0002352	9	Tbc1d5
-1.726970914	1	0.0002362	9	Cd300a
0.435920942	1	0.0002371	9	Anp32e
0.944737966	1	0.0002385	9	Ints3
0.495260019	1	0.0002394	9	Ppfibp1
-1.622695864	1	0.0002402	9	Ptpn23
-1.942658114	1	0.0002402	9	Trib1
-1.175320837	1	0.0002407	9	Ccl6
0.363581291	1	0.0002411	9	Cd52
0.279523578	1	0.0002421	9	Tox4
-1.732126659	1	0.0002422	9	Rsf1
-1.385173793	1	0.0002449	9	Tpp1
-1.635032559	1	0.0002459	9	Tmem261
-1.639799194	1	0.0002464	9	Man1a
0.952163542	1	0.0002474	9	Mfng
0.415981391	1	0.0002492	9	Camkk2
0.3083086	1	0.0002495	9	Kcnn4
0.441205171	1	0.0002512	9	Itgax
0.754582272	1	0.0002573	9	AA467197
-1.623840466	1	0.0002604	9	Kctd10
0.614799796	1	0.0002605	9	1810043G02Rik
-1.655196677	1	0.0002606	9	Fam213b
0.265568146	1	0.0002625	9	Rpl23
1.554683649	1	0.0002646	9	Gm16062
0.26284794	1	0.0002681	9	Nedd1
-1.345518878	1	0.0002689	9	Rassf2
-1.553757527	1	0.0002737	9	Tnfaip1
0.406453291	1	0.0002754	9	Sap30bp
0.297615977	1	0.0002755	9	Slc25a46
-1.002184398	1	0.0002775	9	AI607873
0.291755779	1	0.00028	9	Nemf
0.25331872	1	0.0002841	9	Ppapdc1b
0.481499025	1	0.0002842	9	Nr4a2
-1.847629585	1	0.0002845	9	Idh1
0.272326684	1	0.0002846	9	Nus1
0.351643439	1	0.000285	9	Ralgds
0.440510091	1	0.0002852	9	Siae
0.379907345	1	0.0002854	9	Usp36
-1.768211554	1	0.0002879	9	Itpr2
0.417830311	1	0.0002894	9	Josd2
-0.582461848	1	0.0002944	9	Hexa
-0.57386585	1	0.0002959	9	Rps26
0.355938363	1	0.000297	9	Epc2
0.279491349	1	0.0002989	9	Pgd
0.414374342	1	0.0003018	9	3110082I17Rik

-1.768616379	1	0.0003033	9	Id3
0.403207302	1	0.0003064	9	Crtc3
0.342096639	1	0.0003086	9	Dnajb11
-1.766548362	1	0.0003099	9	Pepd
0.363637816	1	0.0003099	9	2700029M09Rik
0.344124607	1	0.0003101	9	Ifi30
-1.666943858	1	0.0003153	9	Ccnl2
0.364802416	1	0.0003159	9	Psmc1
-1.553168645	1	0.0003164	9	Rnf111
-1.568534842	1	0.000317	9	Irf2bp2
-1.582150867	1	0.0003246	9	Yeats4
-0.295220643	1	0.00033	9	Actb
-1.723024214	1	0.0003329	9	Hvcn1
0.502539933	1	0.0003362	9	Pias3
-1.585516563	1	0.0003364	9	Mob3a
0.408175208	1	0.0003372	9	Tes
0.457187021	1	0.0003385	9	H2afy
0.454722072	1	0.0003386	9	Atp2a3
-1.951748248	1	0.0003388	9	Fcrls
-0.445090243	1	0.0003392	9	Npc2
-1.655553415	1	0.0003461	9	Agtrap
-1.626062336	1	0.0003464	9	Csnk1e
-1.602208481	1	0.0003493	9	Nsmce2
-1.524315027	1	0.0003512	9	Ube3a
-1.631437843	1	0.0003566	9	Slc25a37
0.341046842	1	0.0003584	9	Tnfaip8
-2.023584639	1	0.0003594	9	Igfbp4
0.687001374	1	0.0003609	9	Pygo2
0.787350354	1	0.0003629	9	Senp2
0.360782105	1	0.0003647	9	B3gnt2
0.519580651	1	0.0003671	9	Tmem256
0.332077168	1	0.0003781	9	Itgb7
0.541248349	1	0.0003818	9	Hipk3
0.478023041	1	0.0003822	9	Dock2
0.440463606	1	0.0003834	9	Esd
0.480483556	1	0.0003858	9	Mgrn1
0.286516415	1	0.0003869	9	Ramp1
0.302451554	1	0.000387	9	Acadl
-1.489826791	1	0.0003887	9	Fam63b
0.412769812	1	0.0003904	9	Igf1
-1.459835643	1	0.0003945	9	Tbxas1
0.311939234	1	0.0003955	9	Mis18a
-1.864714091	1	0.0003958	9	Ms4a6b
0.280543332	1	0.0003969	9	Rsrc2
0.5091878	1	0.0003971	9	Vav3
-1.523801989	1	0.0003979	9	Opa1
-1.599475493	1	0.0004029	9	Tra2a
0.251483157	1	0.0004032	9	Topbp1
0.820350116	1	0.0004041	9	Pafah1b3
0.3637894	1	0.0004044	9	Ints1
0.473510985	1	0.0004077	9	Cog5
0.340678667	1	0.0004091	9	Atp2c1
0.275771765	1	0.0004116	9	Adam17
-1.649625276	1	0.0004157	9	Abcc5
0.350981963	1	0.0004168	9	Ets2
-1.674387764	1	0.0004177	9	Hgsnat
0.556502151	1	0.0004224	9	Pdcd6
0.374544706	1	0.0004227	9	Psmb1
0.458979954	1	0.0004293	9	Cggbp1
-1.593868905	1	0.0004325	9	Rgl2
0.25281387	1	0.000433	9	Mrpl32

-1.503748649	1	0.0004342	9	Tmem65
-1.642258932	1	0.0004346	9	Asph
-1.469689786	1	0.0004373	9	Ostm1
-1.636910071	1	0.0004379	9	Fam63a
0.8094898	1	0.0004418	9	Sart1
-1.697573031	1	0.0004429	9	Irf2
0.60093677	1	0.0004489	9	Nostrin
0.473510985	1	0.0004503	9	Sh2d3c
0.329858651	1	0.0004526	9	Vipas39
-1.317750408	1	0.0004625	9	Zfp385a
0.575601496	1	0.0004656	9	Rpn2
-1.597729682	1	0.0004709	9	Maff
0.364965209	1	0.0004779	9	Glipr1
0.316847899	1	0.000478	9	Cpsf2
-1.561855916	1	0.0004922	9	Usp16
-1.450045893	1	0.0004945	9	Ms4a6c
1.802666408	1	0.0004946	9	Siglece
0.333979189	1	0.0005038	9	Frg1
-1.576591199	1	0.0005045	9	Evl
0.302751403	1	0.0005104	9	Cotl1
-1.639165711	1	0.0005109	9	Araf
-1.581701157	1	0.0005113	9	Mtmr3
1.353239396	1	0.0005141	9	Caap1
0.352513703	1	0.0005164	9	Lpcat2
0.306766038	1	0.0005234	9	Spen
0.571645974	1	0.0005287	9	Herpud1
0.336714045	1	0.0005411	9	Fam53a
0.424148117	1	0.0005475	9	Slc25a12
-1.38722837	1	0.0005552	9	Nipbl
-1.68648687	1	0.00056	9	Clec10a
0.983919038	1	0.000563	9	Cmtr1
-1.680217835	1	0.000568	9	Igsf6
-1.481881905	1	0.0005728	9	Mon2
0.322104991	1	0.0005782	9	H13
0.429103604	1	0.0005783	9	Cib1
0.389854651	1	0.0005804	9	Gmppa
-1.527232972	1	0.000593	9	Nipsnap3b
-1.373733942	1	0.0005931	9	Cln3
-1.784521744	1	0.0005955	9	1110008P14Rik
0.352605325	1	0.0006064	9	Tmed9
0.453498684	1	0.0006128	9	Lims1
0.348888494	1	0.0006159	9	Srrm1
0.396359942	1	0.0006226	9	Vav1
-1.532969101	1	0.0006287	9	Adam19
-1.576964213	1	0.0006312	9	Fnta
0.891210794	1	0.0006326	9	Itsn2
0.386345263	1	0.0006328	9	Vamp3
0.278836798	1	0.0006338	9	Ddx42
-1.658076747	1	0.0006364	9	Mxd1
0.404361743	1	0.0006481	9	Anks1
0.373001684	1	0.00065	9	Ptp4a3
0.321762218	1	0.0006506	9	Kdm1a
-1.647167386	1	0.0006508	9	Hspa1a
0.31066442	1	0.0006513	9	Csrp1
-1.523656656	1	0.0006552	9	Herpud2
1.786904486	1	0.000656	9	Trmt2a
-1.538087454	1	0.000662	9	Sep.09
-1.474157563	1	0.0006665	9	Psmd4
-1.574031497	1	0.0006733	9	Bmpr2
-1.465264198	1	0.0006763	9	Vps37a
0.27587299	1	0.0006774	9	Cpsf1

0.315418827	1	0.0006849	9	Ahcyl1
0.28308526	1	0.0006893	9	Pole4
0.396315923	1	0.0006919	9	Atpif1
0.414070257	1	0.0006919	9	Tuba1b
-1.493502455	1	0.0006936	9	Nagpa
0.362292763	1	0.0006967	9	Psme2b
0.277318894	1	0.0007004	9	Map2k3
-1.532886845	1	0.000703	9	Ino80d
-1.412895798	1	0.0007115	9	Pacs2
0.270609425	1	0.0007144	9	Atp6v0a2
0.399865895	1	0.0007161	9	Ppap2c
-1.463011236	1	0.0007199	9	Ywhaq
-1.601740499	1	0.0007237	9	Kcnk6
-1.620945662	1	0.0007286	9	Thbs1
-1.543859363	1	0.0007304	9	Mylip
0.334603018	1	0.0007366	9	Fgr
0.372276229	1	0.000744	9	Sptbn1
0.359282799	1	0.0007461	9	Pafah1b2
0.489832753	1	0.0007501	9	Narf
-1.765417535	1	0.0007552	9	Gas6
0.303444472	1	0.0007566	9	Phpt1
-1.469753337	1	0.0007581	9	Prkag1
-1.582581452	1	0.0007605	9	Aoah
1.097569763	1	0.0007641	9	Taz
-1.512659304	1	0.0007687	9	Cmklr1
0.346513652	1	0.0007746	9	Tnfrsf1a
-1.497176356	1	0.0007784	9	Snhg12
-1.308123691	1	0.0007838	9	Hist1h2bc
0.309736073	1	0.0007849	9	Soat1
-1.434983459	1	0.0007857	9	Trim47
0.299505224	1	0.0007944	9	Mtfr1l
-1.574774311	1	0.0007955	9	Snw1
0.440719721	1	0.0007967	9	Sssca1
0.75769151	1	0.000797	9	Nckipsd
-1.612472816	1	0.000798	9	Rap1gds1
-1.513489231	1	0.0007982	9	Hspbp1
-1.54377506	1	0.0008019	9	Aldh9a1
-1.529610097	1	0.0008031	9	Rb1
0.29124491	1	0.0008034	9	Ccdc47
-1.808208949	1	0.0008092	9	F10
0.391050012	1	0.0008127	9	Camsap2
-1.584355257	1	0.000815	9	C3
0.394584	1	0.0008166	9	Wdr5
-1.435999229	1	0.0008244	9	Rmnd5a
-1.547641765	1	0.0008249	9	Bbip1
0.488843757	1	0.0008288	9	Nol7
-0.652294661	1	0.0008342	9	Cd63
0.282233574	1	0.0008345	9	Dnajc9
-1.440412482	1	0.0008347	9	Tm7sf3
-1.521758121	1	0.0008367	9	Rfk
-1.528911529	1	0.0008371	9	Traf7
-0.637412365	1	0.000838	9	Tnfaip3
-1.206697743	1	0.0008396	9	Serf2
-1.486995634	1	0.0008481	9	Rgcc
1.137378933	1	0.0008518	9	1110038B12Rik
-1.471086966	1	0.0008564	9	Dcaf7
-1.461915189	1	0.0008619	9	Dpp7
0.320049975	1	0.0008638	9	Mrpl33
-1.615963221	1	0.0008658	9	Acbd5
-1.49414211	1	0.0008801	9	Ccrn4l
-1.623971945	1	0.0008828	9	Preb

0.37684111	1	0.0008954	9	Eif3c
-1.507220327	1	0.0008994	9	Sec13
-1.499338051	1	0.0009002	9	Pdxdc1
-1.441495658	1	0.0009006	9	Pdia4
0.413069707	1	0.0009146	9	Ola1
0.27558308	1	0.000917	9	Cpped1
-1.674231133	1	0.00092	9	Taf3
-1.567760302	1	0.000922	9	Nufip2
0.411494506	1	0.0009298	9	Rad23a
0.376248919	1	0.0009506	9	Etfa
0.555267079	1	0.0009605	9	Zfp954
-0.456999978	1	0.0009686	9	Pabpc1
0.280794979	1	0.0009688	9	Tcn2
-1.49153398	1	0.0009732	9	Deptor
-1.399925205	1	0.0009748	9	Slc7a7
0.351501585	1	0.0009923	9	Mrpl17
-1.39189917	1	0.0010147	9	Dap
-1.554562805	1	0.0010242	9	Dennd1a
-1.039004638	1	0.0010244	9	Lyz1
0.48281423	1	0.001025	9	Psmb8
0.334126275	1	0.0010324	9	Surf4
0.314019587	1	0.001036	9	Lig1
0.481609435	1	0.0010374	9	Aspscr1
0.377120467	1	0.0010554	9	Xpnpep1
-1.524409045	1	0.0010678	9	Arl11
-0.282049516	1	0.0011056	9	Jund
0.600372193	1	0.0011071	9	Akt1
-1.278124326	1	0.0011094	9	Srsf9
0.285492338	1	0.0011265	9	Srf
-1.331512662	1	0.0011358	9	Odf2
-1.480372905	1	0.0011424	9	Atp11c
-1.617305016	1	0.0011427	9	Ctla2b
-1.488264222	1	0.0011472	9	Ccdc115
0.254577126	1	0.0011503	9	Rfc3
-0.827280833	1	0.0011503	9	Unc93b1
0.485449912	1	0.0011552	9	Arsk
-1.446057145	1	0.001159	9	Srd5a3
-1.800706446	1	0.0011607	9	C4b
0.39727821	1	0.0011613	9	Cmc1
-1.482302771	1	0.001162	9	Mrpl24
0.39508828	1	0.0011625	9	Rbms1
-1.658723977	1	0.0011664	9	Egr1
0.261419573	1	0.0011875	9	Ttc9c
-1.173089119	1	0.0011905	9	Wsb1
-1.500848463	1	0.0012004	9	Cyhr1
-0.655841858	1	0.001202	9	Sh3glb1
0.383232114	1	0.0012132	9	Rplp2
-1.458987663	1	0.0012179	9	Rbm28
0.724718023	1	0.0012301	9	Noc3l
-1.402190728	1	0.0012338	9	Stag1
0.395905494	1	0.0012377	9	Limd2
-1.370183653	1	0.0012423	9	Tmem176b
-0.264461517	1	0.0012429	9	Tmsb4x
0.335685831	1	0.0012481	9	Gm2a
-1.454117948	1	0.001256	9	Ganab
-1.570817299	1	0.0012644	9	Lbh
-1.34546625	1	0.0012723	9	Ppfia1
0.507979953	1	0.0012835	9	Pkn1
0.328999688	1	0.0012856	9	Snrnp200
-1.610225624	1	0.0012907	9	Ubqln2
0.400436834	1	0.0012913	9	Pgk1

-1.501125637	1	0.0012969	9	Stx5a
-1.600172964	1	0.0012973	9	Tbc1d1
-1.484115664	1	0.0013026	9	Lpxn
-1.475961782	1	0.0013215	9	Psmd10
-1.549152352	1	0.0013224	9	Clec4n
-1.471180111	1	0.0013301	9	Fbxo32
-1.410029972	1	0.0013356	9	Carkd
-1.393348191	1	0.0013462	9	Wls
0.276064185	1	0.0013478	9	Map3k1
-1.515112275	1	0.0013503	9	Mical1
-1.52218033	1	0.0013535	9	Parvg
0.283609241	1	0.0013555	9	Vac14
-1.492975907	1	0.0013567	9	Dnm1l
0.28461668	1	0.0013645	9	Ndufb9
-0.879495477	1	0.0013683	9	Comt
-1.55184264	1	0.0013915	9	Pdrg1
-1.719709857	1	0.0013935	9	Ly6a
-1.150359051	1	0.0013945	9	Ppp1r15a
0.289053823	1	0.0013998	9	Wdfy3
-1.547603662	1	0.0014208	9	Cpsf7
0.749588961	1	0.0014247	9	Rnf4
0.277897729	1	0.0014366	9	Sparc
-1.427038983	1	0.0014509	9	Ptgr1
-1.538093563	1	0.0014517	9	Slc38a6
0.336478134	1	0.0014527	9	Fis1
-1.49218665	1	0.0014546	9	Creld2
-0.784532767	1	0.001458	9	Mir703
0.29878308	1	0.0014647	9	Cct7
-1.468503582	1	0.0014664	9	Larp7
0.315481087	1	0.0014711	9	Nenf
-1.36676249	1	0.0014842	9	Sec24a
0.328110947	1	0.0014909	9	Alg3
0.268862248	1	0.0014928	9	Cul1
-1.799489486	1	0.0015012	9	Dusp6
0.256144394	1	0.0015033	9	Gpn2
0.422646676	1	0.0015043	9	Bre
-1.426342362	1	0.0015142	9	Psmd6
-1.503048345	1	0.0015335	9	Akirin2
0.311743872	1	0.0015518	9	Mknk1
-1.373949719	1	0.001557	9	Col4a3bp
0.475772706	1	0.0015576	9	Erp44
0.539756668	1	0.0015677	9	Mettl5
-1.548426369	1	0.0015702	9	Arid4a
0.600806256	1	0.0015807	9	Gigyf2
0.549533176	1	0.0015871	9	1110012L19Rik
-1.5290561	1	0.0015923	9	Dnajb4
-1.598155941	1	0.0015938	9	Nme3
0.384005937	1	0.0015943	9	Ppt2
0.977501326	1	0.0015961	9	Psmf1
-1.214933378	1	0.0016019	9	Sgpl1
0.538518111	1	0.0016126	9	Rpia
0.295349606	1	0.0016145	9	Basp1
-1.341545825	1	0.0016334	9	Aga
-1.343314172	1	0.0016343	9	Zzz3
-1.351416236	1	0.001644	9	Polr2a
-0.329979179	1	0.0016452	9	C1qb
0.295641246	1	0.0016568	9	Slc25a39
0.704820465	1	0.0016596	9	Eapp
-1.528462192	1	0.001661	9	Naa60
-1.550274988	1	0.0016694	9	Scnm1
-1.430139282	1	0.0016718	9	Tanc2

-1.594452583	1	0.0016723	9	Cept1
-1.539871155	1	0.0016762	9	Ccl8
-1.133093057	1	0.0016769	9	Nr4a1
-1.474108829	1	0.0016779	9	Ankrd40
0.375730387	1	0.0016905	9	Tmem205
0.270612153	1	0.0017064	9	Mbnl2
0.868667351	1	0.001707	9	Sntb2
-1.5480151	1	0.0017074	9	Ezh1
0.297001376	1	0.0017153	9	Epn1
-1.580990678	1	0.001717	9	Rab35
0.379756686	1	0.0017288	9	Nufip1
-1.166092678	1	0.0017576	9	Spns1
-1.427116356	1	0.0017852	9	Gls
-1.363565956	1	0.0017884	9	Arpc1a
-1.44345648	1	0.0017909	9	Klhl9
-1.435590983	1	0.0017975	9	Rnd3
-0.799110228	1	0.0018016	9	Ifitm2
-1.293776243	1	0.0018235	9	Sptssa
-1.345091395	1	0.0018253	9	Prcp
-1.348797291	1	0.0018273	9	Ccdc86
-1.422385648	1	0.0018297	9	Prune2
0.721848651	1	0.001837	9	Tbce
0.260021219	1	0.0018436	9	M6pr
-1.384049129	1	0.0018511	9	Plekhm3
-1.644399339	1	0.0018748	9	Trim30a
-0.698933118	1	0.0018926	9	Rps7
0.265380178	1	0.0018929	9	Actr10
0.346492214	1	0.0018968	9	Cox8a
-1.450666645	1	0.0019212	9	Wrn
0.283924753	1	0.0019264	9	Ccl2
-1.4900189	1	0.0019272	9	Arglu1
-1.525684026	1	0.0019317	9	Mrpl11
-1.486983475	1	0.0019486	9	Ppp1r21
-1.482379034	1	0.0019606	9	Ctsk
-1.405452035	1	0.0019635	9	Rbl2
-1.590694685	1	0.0019664	9	Hagh
-1.434209131	1	0.0019678	9	Gnptg
-1.487367064	1	0.0019844	9	Grsf1
0.308754722	1	0.0019905	9	Rtn3
-1.546597731	1	0.0019954	9	Dnajc13
-1.592860253	1	0.0019981	9	Rcn3
-1.476559425	1	0.0020185	9	Xdh
-1.399947621	1	0.0020468	9	Nup88
-1.496959929	1	0.00206	9	2810004N23Rik
-1.387600628	1	0.0020713	9	Cd300lf
-1.096746302	1	0.0020852	9	Tnf
-1.415599615	1	0.0021023	9	Stard7
-1.444982874	1	0.0021051	9	Gga2
0.962178969	1	0.0021156	9	Tomm20
-1.385649408	1	0.0021231	9	Mat2a
-0.291119989	1	0.0021282	9	NP-904340.1
-2.015964391	1	0.00214	9	Lyve1
-0.280631	1	0.002145	9	Ctss
-1.398669121	1	0.0021506	9	Psme4
-1.554159141	1	0.0021554	9	Acox1
0.751571088	1	0.0021795	9	Ubr1
0.395318065	1	0.0021826	9	Eef1d
-1.436485609	1	0.0021871	9	Tpst2
0.275624662	1	0.0021889	9	Glud1
-1.334695614	1	0.0022046	9	Cd2bp2
-1.359400646	1	0.0022085	9	Gas2l3

0.285102834	1	0.0022112	9	Rps11
-1.378039884	1	0.0022508	9	Arhgap4
0.769700163	1	0.0022591	9	Egln1
-1.616596533	1	0.0022852	9	Cd274
-1.004328956	1	0.0022888	9	Dbi
-1.346502617	1	0.0022952	9	Xrn2
-1.373807982	1	0.0023006	9	4833420G17Rik
-1.404159824	1	0.0023025	9	Gorasp2
-1.517382553	1	0.0023026	9	Soga1
-1.463844498	1	0.0023134	9	Prdm2
-1.281632076	1	0.0023263	9	Ptprj
-1.50098566	1	0.0023349	9	Ttyh3
-1.428697661	1	0.0023478	9	Arhgef7
-1.352109304	1	0.0023635	9	Naglu
0.520964837	1	0.0023703	9	Slc25a15
-0.964620258	1	0.0023832	9	Tgfb1
0.304368991	1	0.002386	9	Atad5
-1.491121152	1	0.0024039	9	Scimp
-1.648323798	1	0.0024045	9	Lifr
-1.519432785	1	0.0024116	9	2810428I15Rik
-1.403179191	1	0.0024156	9	Trim27
-1.404502818	1	0.0024163	9	Nt5c2
0.29983945	1	0.0024314	9	Rpl27
-1.150103383	1	0.002432	9	Pxn
0.699575631	1	0.0024603	9	Hps5
-1.233177426	1	0.0024615	9	Anapc2
-1.297001231	1	0.0025003	9	Cyth1
-1.29086684	1	0.0025031	9	Fabp5
0.303107944	1	0.0025054	9	Gpnmb
-1.427163531	1	0.0025112	9	Smchd1
0.843022918	1	0.0025198	9	Atl2
-1.452025957	1	0.0025268	9	Rapgef1
-1.304194413	1	0.0025275	9	Specc1
-1.347595755	1	0.0025283	9	Ndufaf2
-0.814998124	1	0.0025397	9	Sirt2
-1.587475538	1	0.0025509	9	Nfatc1
-1.353975034	1	0.0025522	9	Zfp704
0.256578327	1	0.0025523	9	Tbk1
-1.397673601	1	0.0025619	9	Snx27
-1.34411102	1	0.0025795	9	Mmp12
-1.312037591	1	0.0025869	9	Zfp281
-1.282168844	1	0.002588	9	Tmem165
-1.394870062	1	0.0026044	9	R3hdm1
-1.371065688	1	0.0026167	9	Zfp36l1
-1.486343628	1	0.0026269	9	Pagr1a
-1.594497719	1	0.0026383	9	Fam126a
-1.636366286	1	0.0026423	9	Gsr
-1.354857588	1	0.0026428	9	2310035C23Rik
-1.435821106	1	0.0026431	9	Nosip
0.319274575	1	0.0026443	9	Eif1ax
-1.549930896	1	0.0026592	9	Klf9
-1.528152186	1	0.0026601	9	Vaultrc5
-1.346373076	1	0.0026812	9	Foxn2
-1.405214815	1	0.0026944	9	Inpp5f
-1.359691036	1	0.0026946	9	Ttc14
-1.374621077	1	0.0027034	9	Ccdc127
-1.343314172	1	0.0027038	9	Chchd7
-1.386777803	1	0.0027046	9	Sh3glb2
0.275260893	1	0.0027064	9	Uqcrq
-1.441001275	1	0.0027166	9	Ndufa10
0.273400854	1	0.0027173	9	Anxa2

0.267211584	1	0.0027423	9	Got2
-1.450908957	1	0.0027448	9	Taf1
-1.410210882	1	0.0027465	9	Vegfa
0.455423205	1	0.0027535	9	Stau1
-0.739055567	1	0.002756	9	Ifrd1
-1.459677372	1	0.0027634	9	Znfx1
-1.354358461	1	0.0027667	9	Rtca
-1.336221944	1	0.0027668	9	Ptafr
-1.505120726	1	0.0027824	9	Brd1
-1.413673942	1	0.002805	9	Trim41
-1.547960531	1	0.0028223	9	Gla
-1.390877652	1	0.002829	9	Mettl6
-1.607986182	1	0.0028458	9	Plekha2
0.35177059	1	0.0028512	9	Gbas
0.863267926	1	0.0028564	9	Dock1
-1.367748198	1	0.0028888	9	Crybg3
-1.473748902	1	0.0028912	9	Tsc22d1
-1.470864818	1	0.0028932	9	Fam20b
-1.513489231	1	0.0028957	9	Tagap
-1.361819326	1	0.0028957	9	Atxn2
0.284289962	1	0.0029294	9	Gpr137b
-1.166806585	1	0.0029302	9	Ormdl2
-0.388684608	1	0.0029393	9	H3f3b
0.33782125	1	0.0029508	9	Clpp
-0.702847511	1	0.0029561	9	Rpl39
0.347278895	1	0.0029703	9	Ifngr1
-1.408400312	1	0.0029802	9	Tns1
-1.351696118	1	0.002982	9	Glrx5
-1.432968604	1	0.0029821	9	1810013L24Rik
-1.599556889	1	0.0030002	9	Smarcd2
-1.084204388	1	0.0030012	9	Bcl6
-1.345479478	1	0.0030072	9	Chka
-1.425877678	1	0.0030245	9	B9d2
-1.38330359	1	0.003028	9	Fbxw7
-1.49218665	1	0.0030291	9	Bcor
-1.407736195	1	0.0030429	9	Stk40
-1.300148282	1	0.0030675	9	Klf13
0.289140585	1	0.003082	9	Atxn3
-1.408508614	1	0.0030883	9	Mllt10
-1.27083575	1	0.0030974	9	Rfwd2
-1.376812826	1	0.0031026	9	Atf2
-1.327320709	1	0.0031211	9	Tor3a
-1.307697343	1	0.0031247	9	Apoa1bp
0.299628418	1	0.0031299	9	Uba1
0.277772518	1	0.0031312	9	Stk17b
0.296604921	1	0.0031413	9	Cd93
-1.369716702	1	0.0031629	9	Parp9
-1.230409425	1	0.0031666	9	Dpf2
1.413732118	1	0.0031723	9	Slc17a5
-1.28604575	1	0.0031809	9	Rnf115
0.441638882	1	0.0032002	9	Flna
0.328197821	1	0.0032133	9	2310047M10Rik
-1.174167796	1	0.0032136	9	Sft2d1
-1.28958153	1	0.0032235	9	Sik1
-1.23829013	1	0.0032302	9	Kmt2b
0.327787416	1	0.0032314	9	Capg
-1.306553832	1	0.0032327	9	Atxn1
0.638782139	1	0.0032416	9	Eif2a
-0.402667301	1	0.0032548	9	Mir692-1
0.287790224	1	0.0032615	9	Prdx5
0.659890582	1	0.0033426	9	Dld

0.431640099	1	0.003345	9	S100a6
-1.56575076	1	0.0033691	9	Bank1
0.687776382	1	0.0033731	9	Oma1
-1.239970803	1	0.0033823	9	Vegfb
-1.449718152	1	0.0034561	9	Dram2
0.296451796	1	0.0034624	9	Aldoa
0.858808907	1	0.0034846	9	Ppfibp2
-1.372202548	1	0.003502	9	Ncln
-1.265897614	1	0.0035174	9	Rp9
-1.242049465	1	0.003538	9	Tesk1
-1.323658277	1	0.0035516	9	Calu
-1.251380416	1	0.0036129	9	Gigyf1
-1.4061041	1	0.003636	9	Zdhhc4
0.282430466	1	0.0036409	9	Tyms
-0.375235068	1	0.0036604	9	Lamp1
0.261919269	1	0.0036775	9	Cryzl1
-1.160790677	1	0.0036797	9	Hnrnpa0
0.30067004	1	0.0036818	9	C2cd2l
-1.379988252	1	0.0036961	9	Sav1
-1.333166952	1	0.0036983	9	Dcp2
0.307477134	1	0.0036991	9	Hn1
0.294209396	1	0.00372	9	Tiam1
-1.202805893	1	0.0037252	9	Bcl7b
-1.436593696	1	0.0037375	9	Ythdf3
-1.034381691	1	0.0037439	9	Rhob
0.254627107	1	0.0037459	9	Ddx41
0.96120418	1	0.0037576	9	BC018507
-1.079711027	1	0.0038231	9	Neat1
-1.365082961	1	0.0038415	9	Abhd4
-0.735747091	1	0.0038672	9	Tmbim6
-1.32246273	1	0.003947	9	Abl2
0.410334514	1	0.0039497	9	Ndufb3
-1.40236378	1	0.0039519	9	Prorsd1
0.366579223	1	0.0039873	9	Tmx2
-1.300331977	1	0.0039986	9	Hadha
0.277721896	1	0.0040147	9	Angel2
-1.435714269	1	0.0040224	9	Wars
-1.419115381	1	0.0040365	9	2310045N01Rik
-1.318009308	1	0.0040382	9	Rnf2
0.570754419	1	0.0040634	9	Ssna1
-1.43756175	1	0.0040927	9	Cd69
-1.373152288	1	0.0041232	9	Usp22
0.292155345	1	0.0041462	9	Tubb6
-1.304290433	1	0.0041855	9	Upf2
-1.400497011	1	0.0041876	9	Arhgap31
0.625671482	1	0.0042005	9	Slc12a6
-1.28530577	1	0.0042492	9	Strn4
0.352792867	1	0.0043037	9	Cd72
-1.292091724	1	0.0043951	9	Slc30a1
-1.31021301	1	0.004405	9	Evi5
0.285340675	1	0.0044242	9	Vps13d
-0.770371359	1	0.0044517	9	Eno1b
-1.47004184	1	0.0044574	9	Lpcat3
-1.442911208	1	0.0045272	9	H1f0
0.477695273	1	0.0045361	9	Mdn1
-1.420552592	1	0.0045379	9	Arl2
-1.329260125	1	0.0045746	9	Qars
-1.40943189	1	0.0046123	9	Myo7a
-1.38324016	1	0.0046161	9	Ncoa2
0.251520935	1	0.0046593	9	Ghitm
-1.490643287	1	0.0047078	9	Map2k2

-0.488802756	1	0.0047142	9	Sdcbp
-1.318747751	1	0.0047144	9	2210016F16Rik
-1.25299162	1	0.004742	9	Crkl
-1.220830822	1	0.00475	9	Gmfg
-1.403773343	1	0.0048175	9	Bscl2
-1.352664253	1	0.004862	9	Csnk1g3
0.519540505	1	0.0048853	9	Syngr1
0.256578327	1	0.0049102	9	St13
0.28215088	1	0.0049492	9	Pfkfb3
-1.288833753	1	0.0049596	9	Mbd6
-1.225685493	1	0.0049664	9	Brox
0.322204396	1	0.0049766	9	Cdc14a
-1.232207855	1	0.004993	9	Dhx30
-1.332110081	1	0.0050005	9	Rnf150
-1.3381028	1	0.0050049	9	Cyp4v3
0.284589778	1	0.0050962	9	Kpna1
-1.47169312	1	0.0051172	9	Mdp1
0.308377823	1	0.0051591	9	Fam53b
-0.320035037	1	0.0051628	9	Map3k11
-1.21857603	1	0.0051964	9	Akap8l
-1.40985773	1	0.005207	9	Slc39a11
-1.460082003	1	0.0052497	9	B4galt6
-1.257597481	1	0.0052743	9	Ints12
0.262031481	1	0.0052905	9	Psma2
-1.234612575	1	0.0053241	9	Snx13
-1.612567805	1	0.0053419	9	Lyl1
-1.537955069	1	0.0053514	9	Nkg7
0.481054156	1	0.0053705	9	Mrps17
0.262782429	1	0.0053915	9	Camk1
0.260142841	1	0.0054121	9	Gfpt1
-1.509685255	1	0.0054154	9	9030624J02Rik
0.286557561	1	0.0054242	9	Ttc1
-1.652350424	1	0.0054493	9	Mrc1
-1.252570513	1	0.0054641	9	Trpm7
-0.26238811	1	0.0054668	9	Ctsb
-0.8696659	1	0.0054714	9	Ubxn6
0.774889283	1	0.0055002	9	Tap1
0.382853426	1	0.0055342	9	Vps29
-1.413743689	1	0.0055478	9	Denr
-1.27598725	1	0.0055793	9	Uba7
0.253509271	1	0.005595	9	Lclat1
-1.316669059	1	0.0056584	9	Hpgds
0.256074422	1	0.0057079	9	Uvssa
-1.368534697	1	0.0057433	9	Pom121
-1.375568524	1	0.0057692	9	N4bp2l2
-1.245877385	1	0.0058941	9	Parp4
0.253156768	1	0.005895	9	Rchy1
-1.281434662	1	0.0059089	9	Tm2d3
-1.527492345	1	0.0059241	9	Mndal
1.49234167	1	0.0060277	9	Tacc3
-1.141521079	1	0.0061361	9	Cxcr4
-0.358455255	1	0.0061504	9	NP-904329.1
1.145394533	1	0.0062099	9	Il6st
-1.312230473	1	0.0062263	9	Mnt
-1.346267391	1	0.0062372	9	Echs1
0.486933755	1	0.0062475	9	B4galt5
0.60676226	1	0.0062878	9	D16Ertd472e
0.677395046	1	0.0063521	9	Pvrl1
0.715188773	1	0.006354	9	Dohh
-1.272572707	1	0.0063561	9	Gxylt1
0.32924037	1	0.0063563	9	Nab2

0.340409505	1	0.0063691	9	Eif6
-1.113229124	1	0.0064283	9	Plekho1
0.2611176	1	0.0064742	9	Zkscan1
-1.060659123	1	0.0064786	9	Ewsr1
-1.304353471	1	0.0064794	9	Adnp
-1.412266427	1	0.0065763	9	Gdf3
0.338055364	1	0.0066171	9	Fkbp4
-0.910301932	1	0.0066479	9	Uqcrb
-1.226934658	1	0.0066857	9	Cyth3
-1.46812623	1	0.0067623	9	Tial1
-1.37972753	1	0.0068124	9	Egr2
0.472751317	1	0.0068137	9	Phf12
-1.251624147	1	0.0068287	9	Bad
0.264836013	1	0.0068855	9	Clic4
-1.466068684	1	0.0069328	9	Pcnt
-1.269993211	1	0.0069332	9	Ap5s1
-1.398977874	1	0.0069486	9	Zcchc17
0.377755411	1	0.0069533	9	Cd209a
0.66036425	1	0.0069605	9	Elk3
-0.938388528	1	0.0069829	9	Gltp
-1.27341891	1	0.006989	9	Mfhas1
-1.357730515	1	0.0070187	9	Mepce
0.348734883	1	0.0070959	9	0610037L13Rik
0.26291485	1	0.0071007	9	Rnaseh2b
-1.309989363	1	0.007207	9	Hbs1l
0.622709033	1	0.0072332	9	Nip7
-1.299684823	1	0.0072594	9	Agpat2
-0.944323729	1	0.0072778	9	Psmb2
0.281656777	1	0.0072892	9	Fkbp1a
-1.363836353	1	0.0073535	9	Setd8
-1.294551614	1	0.0073667	9	Zfp800
0.503343172	1	0.0074368	9	Stk4
-0.425662101	1	0.0074407	9	Pfn1
-1.147013483	1	0.0074594	9	Kansl1
-1.277670945	1	0.0075004	9	Fli1
-1.303474486	1	0.0075671	9	Oas1a
-1.087544543	1	0.0076793	9	Arfgef1
-1.168218267	1	0.0076911	9	Dync1li2
-1.371435971	1	0.0077718	9	Dnpep
-1.468931756	1	0.0077735	9	Fgd4
0.351170597	1	0.0077761	9	Tmem131
0.344072386	1	0.0078496	9	Ptpn6
-1.339149965	1	0.0078508	9	Arhgef12
-1.107826409	1	0.0079651	9	Fh1
-1.315387342	1	0.0079901	9	Cul4b
-0.979083547	1	0.0079973	9	Ccl3
-1.116700666	1	0.00804	9	Plxnd1
-1.290846608	1	0.0080439	9	Tmem141
-1.369954018	1	0.0081405	9	Slc16a3
-0.847968697	1	0.0081888	9	Gm6402
0.250303623	1	0.0081935	9	2410006H16Rik
-1.26435807	1	0.0083109	9	Hscb
-1.287631171	1	0.0083603	9	Fmnl2
0.303682797	1	0.0083966	9	Kif1c
-1.375823061	1	0.0084584	9	Fam168b
-1.11930194	1	0.008462	9	Pnpla8
-1.554148405	1	0.0084715	9	Cyb5r1
0.766336145	1	0.0085649	9	Dnajc14
0.930009645	1	0.008593	9	Upf3b
-1.389874341	1	0.0087191	9	Il18bp
0.548720168	1	0.0087317	9	Utp14b

-1.237528897	1	0.0087366	9	Gtf2a1
0.258905413	1	0.008771	9	Dlg1
-1.121329673	1	0.0087734	9	Gnai3
-1.058158892	1	0.0088539	9	Midn
-1.15445348	1	0.0088712	9	Hars
-1.42629769	1	0.0089111	9	Rtfdc1
-1.368446928	1	0.0089841	9	Asxl1
-1.036816141	1	0.009067	9	Amdhd2
0.444970907	1	0.0090716	9	Cacna1d
-1.196943734	1	0.0090878	9	Matr3
-1.389027911	1	0.0090934	9	Cebpd
0.294140261	1	0.0091206	9	Pnisr
0.318471344	1	0.0091349	9	Sord
-0.601458141	1	0.0091393	9	Snx5
-1.407146081	1	0.0091514	9	Ncbp2
0.565916971	1	0.0091528	9	Ogdh
-1.296206558	1	0.0092159	9	Trim26
0.353468474	1	0.0092312	9	Abcf1
-0.773804122	1	0.0092465	9	Atp6v1f
-1.357241861	1	0.0092909	9	Sema4a
-1.197469399	1	0.0093206	9	Apaf1
-1.080144314	1	0.0093242	9	Lman1
-0.51663776	1	0.0094128	9	Pltp
0.281045945	1	0.0094716	9	Vti1b
0.621248468	1	0.0094904	9	Aco2
-1.163350095	1	0.0095135	9	Scamp1
-1.009769756	1	0.0095691	9	Mrfap1
-1.267798177	1	0.0095972	9	Sod2
-1.331732806	1	0.0096301	9	Sos2
0.395858845	1	0.0096463	9	Dek
-1.561589411	1	0.0096592	9	Mfap3
-1.167318566	1	0.0097023	9	Glb1
-1.253404799	1	0.0097167	9	Map4k3
0.319413754	1	0.0098617	9	Srpk2
-1.224732283	1	0.0098685	9	Mroh1
-1.197458123	1	0.0099842	9	Dnajb14
-1.067341835	1	0.0099901	9	Gak
-1.192992365	1	0.0099964	9	Sqrdl
1.868000817	8.17E-133	6.12E-137	10	Arhgap33
2.028809907	5.48E-131	4.10E-135	10	Neil3
1.102356884	1.65E-118	1.23E-122	10	Prodh
1.355375182	1.87E-104	1.40E-108	10	Zbtb32
2.048864859	1.41E-103	1.06E-107	10	Mss51
1.686898798	1.19E-101	8.91E-106	10	Shcbp1
1.721788615	8.90E-87	6.67E-91	10	1110046J04Rik
2.234875095	2.06E-86	1.55E-90	10	S1pr3
2.377454483	1.17E-84	8.79E-89	10	Sgol1
2.461355866	3.53E-83	2.64E-87	10	Lrrc32
1.243054638	5.22E-81	3.91E-85	10	Mex3a
1.391602542	2.39E-80	1.79E-84	10	Zwilch
1.204946989	4.93E-79	3.69E-83	10	Zfat
2.07736589	3.93E-78	2.94E-82	10	Ccnb1
0.925506933	1.05E-77	7.83E-82	10	Ccdc176
1.653562717	2.81E-77	2.11E-81	10	Mtx3
2.095016753	4.41E-76	3.30E-80	10	Ccdc61
1.647201355	1.25E-75	9.39E-80	10	Spag5
1.322218225	1.83E-74	1.37E-78	10	Fam185a
1.128694557	3.77E-71	2.82E-75	10	Sapcd2
0.792269563	5.48E-71	4.10E-75	10	Olfr110
1.765406891	1.23E-70	9.22E-75	10	Pigg
1.335128447	1.53E-68	1.15E-72	10	Mak

1.504207343	4.57E-67	3.42E-71	10	Cox6b2
0.594695089	1.21E-66	9.08E-71	10	Spta1
1.045892128	2.15E-65	1.61E-69	10	Slc9a5
2.586746691	3.68E-63	2.75E-67	10	Pemt
0.869708281	6.61E-63	4.95E-67	10	Hist1h2bj
1.106011389	1.15E-62	8.65E-67	10	3110057O12Rik
2.131883601	4.15E-62	3.11E-66	10	Bard1
2.008442361	7.72E-62	5.78E-66	10	Pbk
1.752094471	1.14E-61	8.52E-66	10	Fanca
1.919629263	5.31E-61	3.98E-65	10	Ocstamp
1.346358643	2.84E-60	2.13E-64	10	D130040H23Rik
2.377573983	1.50E-59	1.12E-63	10	Htra4
1.425822814	1.79E-59	1.34E-63	10	Il23a
1.298001754	1.90E-59	1.42E-63	10	Stag3
1.27410765	3.99E-59	2.99E-63	10	Rasl12
1.09798062	2.95E-58	2.21E-62	10	Fignl1
1.471110348	5.82E-58	4.36E-62	10	Mfsd12
0.917296482	9.10E-57	6.82E-61	10	Gm3336
2.363100905	1.34E-56	1.01E-60	10	D630041G03Rik
1.643924089	2.52E-56	1.89E-60	10	Nuf2
1.037097682	4.08E-56	3.06E-60	10	Hist1h1a
2.634435998	9.31E-56	6.97E-60	10	8030462N17Rik
1.264559391	1.39E-55	1.04E-59	10	Zfp606
2.465603828	6.81E-55	5.10E-59	10	Foxm1
1.332750055	5.42E-54	4.06E-58	10	F9
1.459093861	2.33E-53	1.75E-57	10	Mxd3
2.819404875	1.46E-52	1.09E-56	10	Myh11
2.391720741	1.76E-52	1.32E-56	10	Slamf6
0.791309409	2.84E-51	2.13E-55	10	Ano1
0.689308404	4.06E-51	3.04E-55	10	Ctnna3
1.001365016	4.57E-51	3.42E-55	10	Kcnmb1
0.657593477	7.14E-51	5.35E-55	10	Tube1
1.234894715	8.16E-50	6.11E-54	10	Rps6ka2
1.632213396	7.16E-49	5.36E-53	10	Mast4
1.549777389	1.74E-48	1.30E-52	10	Mmachc
1.705478977	1.02E-47	7.64E-52	10	Entpd2
1.787850608	1.81E-47	1.35E-51	10	Tcaim
1.201396389	2.06E-47	1.54E-51	10	Ankrd55
0.520335847	2.37E-47	1.77E-51	10	Ldb3
0.436767256	3.68E-47	2.75E-51	10	Ankrd2
0.951467907	1.03E-45	7.69E-50	10	Upp1
0.997031228	1.12E-45	8.39E-50	10	Ccdc138
1.965034161	1.85E-45	1.38E-49	10	Ccdc3
0.856470858	4.86E-45	3.64E-49	10	Synpo2
1.929144431	5.44E-45	4.07E-49	10	Kif4
0.687083313	5.59E-45	4.19E-49	10	Jph2
2.042606016	5.59E-45	4.19E-49	10	Cnn1
0.835656483	6.00E-45	4.49E-49	10	Slc6a4
1.39004167	2.63E-44	1.97E-48	10	Mboat1
1.592799907	2.89E-44	2.17E-48	10	Poc1a
2.105683021	3.91E-43	2.93E-47	10	Dmpk
1.822146079	5.07E-43	3.80E-47	10	Dmkn
1.659962272	6.33E-43	4.74E-47	10	Dlgap5
1.474418065	1.58E-42	1.18E-46	10	Lin52
1.455638687	1.89E-42	1.41E-46	10	Chchd6
1.991450791	6.10E-42	4.57E-46	10	Iqgap3
2.778122399	2.51E-41	1.88E-45	10	Tagln
1.118534703	2.56E-41	1.92E-45	10	Adc
2.133033688	3.62E-41	2.71E-45	10	Spc25
1.456853754	3.82E-40	2.86E-44	10	Fbxw17
0.384885068	6.24E-40	4.68E-44	10	Sost

0.912386223	7.36E-40	5.51E-44	10	Hlf
1.236667241	8.38E-40	6.28E-44	10	Olfr920
0.522696504	1.14E-39	8.57E-44	10	Zfp27
1.657706282	3.13E-39	2.34E-43	10	Cenpo
1.659066935	6.45E-39	4.83E-43	10	Hinfp
1.399847328	6.69E-39	5.01E-43	10	Cacna1f
1.207475435	1.20E-38	9.02E-43	10	Arhgef17
1.542160459	1.45E-38	1.08E-42	10	Kif15
1.827905549	2.13E-38	1.60E-42	10	Mis18bp1
1.691595325	5.13E-38	3.84E-42	10	Mtrf1
1.685245773	6.55E-38	4.91E-42	10	Thop1
1.232957622	7.66E-38	5.74E-42	10	D830031N03Rik
1.673941784	9.14E-38	6.85E-42	10	Recql5
1.99236493	1.17E-37	8.74E-42	10	Thtpa
1.252992146	1.25E-37	9.39E-42	10	Snapc3
1.95313398	1.85E-37	1.39E-41	10	Sgsm3
1.967575086	3.21E-37	2.40E-41	10	Rtel1
1.953991146	3.64E-37	2.73E-41	10	Rrp12
2.434855238	3.68E-37	2.75E-41	10	Slc43a3
2.412353041	4.18E-37	3.13E-41	10	Usp6nl
1.701039468	5.13E-37	3.84E-41	10	Fam222b
1.637801745	6.02E-37	4.51E-41	10	Dhdh
0.614032458	8.79E-37	6.59E-41	10	Flnc
1.002402678	2.60E-36	1.95E-40	10	Aurkb
0.894799681	2.78E-36	2.08E-40	10	Prr11
1.202008922	3.06E-36	2.29E-40	10	Cenpn
1.396677753	6.47E-36	4.85E-40	10	Tmem144
1.228129557	9.60E-36	7.19E-40	10	Ebag9
0.917075969	1.11E-35	8.34E-40	10	Lmln
1.307668549	1.54E-35	1.15E-39	10	Brca2
1.739702185	2.49E-35	1.86E-39	10	Rbms3
0.550683795	2.97E-35	2.22E-39	10	Lrfn4
1.045419576	3.72E-35	2.79E-39	10	Fam64a
1.619210156	4.69E-35	3.51E-39	10	Stil
1.210495865	7.19E-35	5.39E-39	10	Map1b
0.608028619	1.37E-34	1.02E-38	10	Slc2a10
1.525564051	1.62E-34	1.21E-38	10	5830415F09Rik
0.961831374	1.67E-34	1.25E-38	10	Sgca
0.810431429	1.89E-34	1.41E-38	10	Rab11fip3
1.095097347	2.86E-34	2.14E-38	10	4632434I11Rik
0.937811868	3.20E-34	2.39E-38	10	Sacs
1.453771421	8.40E-34	6.29E-38	10	Ckap2
1.08889757	2.97E-33	2.23E-37	10	Itga7
0.509402136	4.19E-33	3.14E-37	10	Gen1
0.755231947	7.12E-33	5.33E-37	10	Ska3
1.013973229	7.43E-33	5.57E-37	10	Pif1
1.051925175	9.00E-33	6.74E-37	10	Qtrtd1
1.585773256	1.25E-32	9.38E-37	10	Crispld2
1.750603572	1.60E-32	1.20E-36	10	Cd80
1.996458342	4.48E-32	3.35E-36	10	Usp11
1.39955574	1.33E-31	9.93E-36	10	Ncaph
1.112165255	3.24E-31	2.42E-35	10	Dtl
1.194441602	3.39E-31	2.54E-35	10	Snx33
1.136016471	3.82E-31	2.86E-35	10	Dmd
0.717124664	4.43E-31	3.32E-35	10	Zfp553
1.307031291	6.58E-31	4.93E-35	10	2610020H08Rik
1.802988204	9.10E-31	6.81E-35	10	Ttc12
0.991580312	9.50E-31	7.12E-35	10	Cdc45
1.025637688	1.12E-30	8.38E-35	10	Cep55
1.552906066	1.35E-30	1.01E-34	10	Crtam
1.934120941	2.48E-30	1.86E-34	10	Cdkn3

0.85052866	5.80E-30	4.34E-34	10	Nexn
0.906902129	6.25E-30	4.68E-34	10	Pex12
1.691239979	7.11E-30	5.33E-34	10	Tbck
1.499699972	7.20E-30	5.39E-34	10	Msh3
1.11140451	8.81E-30	6.60E-34	10	Gpr97
1.490025097	9.43E-30	7.06E-34	10	Pdcd1lg2
0.991333639	9.90E-30	7.42E-34	10	2810442I21Rik
1.230567294	1.85E-29	1.39E-33	10	Tk1
1.385196063	2.02E-29	1.51E-33	10	Spc24
2.070195593	5.06E-29	3.79E-33	10	Myl9
0.636966809	5.57E-29	4.17E-33	10	Nek2
1.686158598	5.99E-29	4.49E-33	10	Slfn10-ps
1.881767276	7.92E-29	5.93E-33	10	Eftud1
2.297310566	9.55E-29	7.15E-33	10	Ube2c
2.124640353	9.88E-29	7.40E-33	10	Dhx33
2.167990269	1.04E-28	7.77E-33	10	Pvt1
1.609968773	1.11E-28	8.31E-33	10	Dis3
1.290291421	1.16E-28	8.70E-33	10	Nxn
0.81756274	1.91E-28	1.43E-32	10	N4bp3
1.761020953	2.84E-28	2.13E-32	10	Rps6kb2
1.829149858	3.53E-28	2.64E-32	10	Rdh11
1.614857493	4.27E-28	3.20E-32	10	1190002F15Rik
1.523648436	4.43E-28	3.32E-32	10	B4galt7
1.05915122	5.93E-28	4.44E-32	10	Ltb4r1
1.687015129	6.93E-28	5.19E-32	10	Isg20l2
2.898085947	1.30E-27	9.72E-32	10	Cdca3
2.135321499	1.67E-27	1.25E-31	10	Vdr
1.264633849	1.79E-27	1.34E-31	10	Hspb6
0.962517059	2.18E-27	1.63E-31	10	Serpinb12
0.636966809	3.06E-27	2.29E-31	10	Ubash3a
1.349391522	3.11E-27	2.33E-31	10	Impa2
1.024010741	3.26E-27	2.45E-31	10	AW554918
1.459288326	5.42E-27	4.06E-31	10	5330426P16Rik
1.533875789	6.85E-27	5.13E-31	10	Tmem80
1.421627217	7.85E-27	5.88E-31	10	Mlh1
2.785026417	8.10E-27	6.07E-31	10	Tex2
1.064023179	8.31E-27	6.22E-31	10	Zfp874b
0.898214152	8.77E-27	6.57E-31	10	Piga
0.511727473	8.97E-27	6.72E-31	10	Myrf
1.246103731	9.33E-27	6.99E-31	10	Pus7l
1.63705949	9.49E-27	7.10E-31	10	Ccnb2
1.179905778	1.22E-26	9.11E-31	10	Zfyve1
2.121252763	2.24E-26	1.68E-30	10	Satb1
1.18524495	4.05E-26	3.03E-30	10	Hook2
1.473228577	4.29E-26	3.21E-30	10	Fam206a
1.37600367	4.32E-26	3.24E-30	10	Mlkl
1.187589017	5.00E-26	3.74E-30	10	Zfp1
1.385503621	5.37E-26	4.02E-30	10	Elac2
1.239865727	1.31E-25	9.78E-30	10	Trub2
0.775172727	2.08E-25	1.56E-29	10	Med26
1.857137503	2.59E-25	1.94E-29	10	Cald1
1.736717114	2.69E-25	2.02E-29	10	Vwa8
1.758115143	2.75E-25	2.06E-29	10	E2f5
0.828112951	2.92E-25	2.19E-29	10	Pcdh7
1.971708597	5.10E-25	3.82E-29	10	Cd209d
0.4851291	6.61E-25	4.95E-29	10	2310034O05Rik
1.583449518	8.57E-25	6.42E-29	10	Cdca8
1.055241705	1.06E-24	7.96E-29	10	Ccdc77
1.027007447	1.12E-24	8.39E-29	10	Pcyt2
1.102503344	2.43E-24	1.82E-28	10	Kif20b
1.63785779	3.32E-24	2.49E-28	10	Dph5

1.632635846	3.68E-24	2.76E-28	10	AU022793
0.912422634	5.64E-24	4.22E-28	10	Pacsin3
0.52088121	6.51E-24	4.88E-28	10	Mars2
0.647307694	8.49E-24	6.36E-28	10	Gstcd
1.055367657	9.76E-24	7.31E-28	10	Xylt1
0.751301565	1.04E-23	7.79E-28	10	Mastl
0.396699552	1.07E-23	8.04E-28	10	Kcnma1
0.386970091	1.39E-23	1.04E-27	10	Pacrg
1.177264157	1.85E-23	1.38E-27	10	5730455P16Rik
1.41605057	2.59E-23	1.94E-27	10	Rybp
2.126966579	3.18E-23	2.38E-27	10	9430016H08Rik
0.535066504	3.98E-23	2.98E-27	10	2310034G01Rik
1.02133284	4.27E-23	3.19E-27	10	Cdyl2
1.321155059	4.54E-23	3.40E-27	10	Abcc9
1.193134318	4.66E-23	3.49E-27	10	Rnf24
0.428923604	5.13E-23	3.84E-27	10	Tbc1d30
1.596967105	6.93E-23	5.19E-27	10	Zfp623
1.676901254	1.12E-22	8.38E-27	10	Tmco4
2.679545608	1.23E-22	9.18E-27	10	Zfp511
1.461466536	1.73E-22	1.30E-26	10	Trip6
1.479534595	1.77E-22	1.33E-26	10	Trim37
1.556851076	2.11E-22	1.58E-26	10	Suv39h1
1.383200094	2.33E-22	1.74E-26	10	Def8
1.185097725	2.84E-22	2.13E-26	10	Ncapg
1.000725693	3.82E-22	2.86E-26	10	Cep57l1
1.133363413	6.07E-22	4.54E-26	10	Kif11
1.122469485	6.20E-22	4.64E-26	10	Nol9
0.839328605	7.39E-22	5.53E-26	10	Knstrn
1.031806042	7.92E-22	5.93E-26	10	Usp53
1.662870245	9.59E-22	7.19E-26	10	Rarg
2.89694048	1.12E-21	8.38E-26	10	Ccl17
1.247883397	1.29E-21	9.66E-26	10	Hist1h4d
0.967273901	1.33E-21	9.98E-26	10	Npm3
0.905944129	1.80E-21	1.35E-25	10	Nalcn
0.954358264	2.03E-21	1.52E-25	10	Mphosph9
1.196532294	2.12E-21	1.59E-25	10	St7
1.32382145	2.68E-21	2.01E-25	10	Wdhd1
0.649296809	3.90E-21	2.92E-25	10	Cystm1
1.30982697	4.17E-21	3.12E-25	10	Diap3
2.558728176	4.26E-21	3.19E-25	10	Birc5
1.357756297	4.49E-21	3.36E-25	10	Polr1b
0.946872399	4.59E-21	3.44E-25	10	Fam73a
1.058098034	4.62E-21	3.46E-25	10	Unc119b
1.236129326	5.16E-21	3.87E-25	10	Pomt1
1.084055296	5.26E-21	3.94E-25	10	Ndc80
1.752013854	7.03E-21	5.27E-25	10	Ncapd3
0.777430638	7.05E-21	5.28E-25	10	Card9
1.634623999	7.77E-21	5.82E-25	10	Zswim7
0.952864349	1.06E-20	7.92E-25	10	Rpap2
1.475070749	1.19E-20	8.92E-25	10	Racgap1
1.70369335	1.32E-20	9.85E-25	10	Dcps
2.308743506	1.46E-20	1.09E-24	10	Cenpe
0.603515911	1.53E-20	1.14E-24	10	Zfp518b
1.175611477	1.64E-20	1.23E-24	10	Nol10
1.072651305	1.98E-20	1.48E-24	10	Mtrf1l
0.887964062	2.27E-20	1.70E-24	10	Zfp58
0.913531912	2.56E-20	1.92E-24	10	Hyal1
1.169830897	3.02E-20	2.26E-24	10	Lysmd3
1.456461227	3.58E-20	2.68E-24	10	Pwp2
0.862704395	3.76E-20	2.82E-24	10	Tubgcp6
1.001485578	3.96E-20	2.97E-24	10	Ppm1f

1.735371865	4.43E-20	3.32E-24	10	Chpf2
1.175352972	4.67E-20	3.50E-24	10	H2afx
1.184138755	6.12E-20	4.58E-24	10	Zfp429
1.543921058	7.83E-20	5.86E-24	10	Zcchc10
1.356314618	1.08E-19	8.12E-24	10	Slc30a6
0.91872133	1.15E-19	8.64E-24	10	Coprs
1.349034258	1.35E-19	1.01E-23	10	Cenpf
0.674372912	1.46E-19	1.09E-23	10	Tdp1
1.013459486	1.53E-19	1.15E-23	10	Pard6a
1.264842095	1.69E-19	1.27E-23	10	Zfp53
0.793846213	1.86E-19	1.39E-23	10	Ccna2
1.149673914	1.98E-19	1.49E-23	10	Klhl11
1.204618822	2.19E-19	1.64E-23	10	Ap3m2
1.363027706	2.49E-19	1.86E-23	10	Pnpt1
2.016965586	2.65E-19	1.98E-23	10	Tpx2
0.966771789	2.95E-19	2.21E-23	10	Adat2
0.99633344	3.08E-19	2.31E-23	10	Sirpb1a
1.360817984	3.37E-19	2.52E-23	10	Ccdc91
1.264315934	3.42E-19	2.56E-23	10	Emc9
0.835599159	4.24E-19	3.17E-23	10	Pi15
1.321433948	4.39E-19	3.29E-23	10	Alg8
0.653983365	4.50E-19	3.37E-23	10	Palld
1.262524461	6.51E-19	4.88E-23	10	Hemk1
1.092444844	6.55E-19	4.90E-23	10	Bid
1.065815496	6.83E-19	5.12E-23	10	Usp30
1.576693704	7.07E-19	5.29E-23	10	Cdh1
1.577084989	8.78E-19	6.57E-23	10	Crip2
1.6061455	1.13E-18	8.47E-23	10	Ppp2r5d
0.70748848	1.13E-18	8.48E-23	10	Brca1
1.289237456	1.33E-18	9.94E-23	10	Ttc37
1.989935489	1.61E-18	1.20E-22	10	Rad9a
2.155936628	1.73E-18	1.29E-22	10	Prc1
1.787632417	1.74E-18	1.30E-22	10	Mast2
1.946267643	1.94E-18	1.45E-22	10	Dkc1
1.45066898	2.17E-18	1.62E-22	10	Rffl
1.692598946	2.20E-18	1.65E-22	10	Ccdc163
1.874014989	2.46E-18	1.84E-22	10	D10Wsu102e
2.050348965	2.52E-18	1.89E-22	10	Slc7a1
0.740601699	2.91E-18	2.18E-22	10	Gm5512
0.863156782	2.92E-18	2.18E-22	10	Dppa3
1.267840772	3.16E-18	2.37E-22	10	Wipi2
2.078712567	3.26E-18	2.44E-22	10	Acta2
0.598367861	3.45E-18	2.59E-22	10	Zfp719
0.815495912	3.70E-18	2.77E-22	10	Adsl
1.551629329	4.13E-18	3.10E-22	10	Car13
0.998505937	4.76E-18	3.56E-22	10	Vezt
1.309294063	6.00E-18	4.49E-22	10	Gtf3c4
1.12468778	6.30E-18	4.72E-22	10	Cdk5rap1
1.728511222	7.42E-18	5.56E-22	10	Ttpal
1.601356316	7.59E-18	5.69E-22	10	Carm1
1.66903001	8.77E-18	6.57E-22	10	Hspg2
0.515881748	9.08E-18	6.80E-22	10	Grhl1
1.404572277	9.49E-18	7.11E-22	10	Gatsl3
1.025358147	1.01E-17	7.58E-22	10	Mvk
0.571034282	1.01E-17	7.58E-22	10	Serpine2
1.381921111	1.27E-17	9.49E-22	10	G2e3
0.833560401	1.74E-17	1.30E-21	10	Helz2
1.037779544	1.82E-17	1.36E-21	10	Snrnp35
0.835121581	2.32E-17	1.73E-21	10	Acbd4
1.214559785	2.45E-17	1.84E-21	10	Hdac3
0.921981547	3.08E-17	2.30E-21	10	Rapgef5

1.436334261	3.17E-17	2.38E-21	10	Rfc3
0.789008794	3.28E-17	2.46E-21	10	Frat2
1.395796689	3.48E-17	2.61E-21	10	Nup133
1.031878377	3.82E-17	2.86E-21	10	Abtb1
1.299233945	3.82E-17	2.86E-21	10	Gle1
1.204663035	4.90E-17	3.67E-21	10	Flad1
1.326636538	5.42E-17	4.06E-21	10	Smad3
0.880776918	6.65E-17	4.98E-21	10	9430020K01Rik
1.909663656	7.00E-17	5.24E-21	10	Dhdds
1.066158982	7.15E-17	5.36E-21	10	Smim24
1.836265948	7.16E-17	5.37E-21	10	Supt20
1.400970292	8.98E-17	6.73E-21	10	Chml
0.800603909	9.26E-17	6.93E-21	10	Zfp956
0.559492521	1.12E-16	8.35E-21	10	4632427E13Rik
1.302342312	1.59E-16	1.19E-20	10	1110001J03Rik
0.539338109	1.70E-16	1.27E-20	10	Capn10
1.459163905	1.79E-16	1.34E-20	10	Map4k1
1.698009364	1.89E-16	1.42E-20	10	Nup210
1.554383884	2.00E-16	1.50E-20	10	Nck1
1.775647575	2.01E-16	1.50E-20	10	Gpr171
1.425063199	2.26E-16	1.69E-20	10	Rdh13
1.173223999	2.36E-16	1.77E-20	10	Asb6
1.489161584	2.42E-16	1.81E-20	10	Fastkd2
0.934771228	2.64E-16	1.98E-20	10	2610034B18Rik
1.93408862	2.65E-16	1.99E-20	10	Cpe
1.072410497	2.69E-16	2.02E-20	10	Zfp948
1.587625924	2.89E-16	2.17E-20	10	Ckap2l
1.342977021	2.91E-16	2.18E-20	10	Dnajc9
0.924456998	3.03E-16	2.27E-20	10	Amica1
0.69279546	3.36E-16	2.52E-20	10	Exog
0.869603618	3.62E-16	2.71E-20	10	Gstt2
1.214559785	3.64E-16	2.73E-20	10	Zfp943
0.944284382	4.06E-16	3.04E-20	10	Bub1b
1.118485299	4.21E-16	3.15E-20	10	1110065P20Rik
0.974014917	4.41E-16	3.31E-20	10	Klf11
1.7643501	5.37E-16	4.02E-20	10	Csnk2a2
1.68536351	6.12E-16	4.58E-20	10	Rai14
1.905764015	9.19E-16	6.88E-20	10	Itga8
0.803689482	1.15E-15	8.64E-20	10	Abhd10
0.736375915	1.32E-15	9.90E-20	10	AI846148
0.73285286	1.39E-15	1.04E-19	10	Syn1
0.803629346	1.60E-15	1.20E-19	10	Aph1a
0.566206515	1.66E-15	1.24E-19	10	Elp6
1.533890884	1.66E-15	1.24E-19	10	Pelp1
0.906456766	1.82E-15	1.37E-19	10	Chtf18
2.069767657	1.99E-15	1.49E-19	10	Oma1
1.184475474	2.29E-15	1.71E-19	10	Rpp14
1.018613317	2.38E-15	1.79E-19	10	Gatsl2
0.940036563	2.68E-15	2.01E-19	10	A530054K11Rik
1.104785148	2.77E-15	2.07E-19	10	Wdr4
0.544229952	2.93E-15	2.20E-19	10	Hist1h3d
1.423893748	3.36E-15	2.52E-19	10	Ctgf
0.875403273	3.53E-15	2.64E-19	10	Gm10400
1.077368703	3.54E-15	2.65E-19	10	Angptl4
0.920416162	4.05E-15	3.03E-19	10	Mx2
1.181984232	4.29E-15	3.21E-19	10	Bola1
0.938223475	4.95E-15	3.71E-19	10	Tom1l2
1.563658858	5.14E-15	3.85E-19	10	Spdl1
0.997915792	5.52E-15	4.14E-19	10	Nicn1
1.806424164	5.84E-15	4.38E-19	10	Eefsec
1.441072654	5.98E-15	4.48E-19	10	Arhgap11a

1.352688212	6.32E-15	4.74E-19	10	Spint1
0.779490792	6.82E-15	5.11E-19	10	Gramd4
1.293492282	8.59E-15	6.43E-19	10	Dis3l
0.955722311	9.27E-15	6.94E-19	10	Aldh4a1
1.078548644	9.80E-15	7.34E-19	10	Unk
1.775739624	1.01E-14	7.60E-19	10	Taf4a
1.626740318	1.12E-14	8.38E-19	10	Myg1
1.369423702	1.16E-14	8.68E-19	10	A830080D01Rik
1.246103731	1.22E-14	9.13E-19	10	Cep128
0.945727731	1.23E-14	9.20E-19	10	Trmt61b
0.566882678	1.23E-14	9.25E-19	10	Tctn3
1.591292504	1.30E-14	9.71E-19	10	Ndufaf4
1.322181612	1.41E-14	1.05E-18	10	G530011O06Rik
1.028395372	1.52E-14	1.14E-18	10	Rbpms
1.141081752	1.58E-14	1.18E-18	10	Slc16a1
1.666220699	1.66E-14	1.24E-18	10	Maz
0.342185642	2.15E-14	1.61E-18	10	Sbk1
0.938068311	2.35E-14	1.76E-18	10	Prkci
1.601753354	2.62E-14	1.97E-18	10	Tacc3
0.857427338	2.92E-14	2.19E-18	10	Hic2
1.80779152	2.98E-14	2.23E-18	10	Hsf1
1.451365568	3.26E-14	2.44E-18	10	Pwp1
0.855254841	4.05E-14	3.03E-18	10	Depdc5
0.954146098	4.11E-14	3.08E-18	10	Trmt10a
1.229456286	4.46E-14	3.34E-18	10	Zfp790
0.818415486	4.51E-14	3.38E-18	10	Edc3
0.767888793	4.55E-14	3.41E-18	10	Pkmyt1
1.156109558	4.96E-14	3.72E-18	10	Xpa
0.708359898	5.78E-14	4.33E-18	10	Ndufaf6
0.819331188	5.92E-14	4.43E-18	10	Card11
1.061162701	6.87E-14	5.15E-18	10	Cdan1
1.338938574	7.46E-14	5.58E-18	10	Spata5
1.469533309	7.99E-14	5.98E-18	10	Sipa1l1
0.829954472	8.30E-14	6.22E-18	10	Nudt6
0.854960866	8.33E-14	6.24E-18	10	Pomgnt1
0.825937728	8.41E-14	6.30E-18	10	Pold2
0.717734742	9.75E-14	7.31E-18	10	Tspyl3
1.032366777	9.79E-14	7.33E-18	10	Gm16062
1.068350328	9.85E-14	7.38E-18	10	Pusl1
0.623998794	1.09E-13	8.18E-18	10	Kctd9
1.368190008	1.20E-13	9.00E-18	10	Atg4b
0.688351008	1.23E-13	9.19E-18	10	Cenpq
1.240802439	1.25E-13	9.40E-18	10	Mcat
0.637020226	1.40E-13	1.05E-17	10	Fastk
0.95397008	1.41E-13	1.06E-17	10	Casc5
0.961159232	1.51E-13	1.13E-17	10	Slc30a4
0.785143768	1.74E-13	1.30E-17	10	Smim4
1.173672552	1.75E-13	1.31E-17	10	5730408K05Rik
1.294865672	1.94E-13	1.45E-17	10	Dera
1.372238863	2.22E-13	1.66E-17	10	Bag4
0.335607226	2.36E-13	1.76E-17	10	Cdk5r1
1.686441582	2.63E-13	1.97E-17	10	Zbed6
1.380858832	3.19E-13	2.39E-17	10	Atg10
1.805312436	3.56E-13	2.67E-17	10	Smc2
0.870472156	3.63E-13	2.72E-17	10	1110059E24Rik
0.660357358	3.72E-13	2.78E-17	10	Alg6
0.968709864	3.79E-13	2.84E-17	10	2810417H13Rik
1.260244978	4.45E-13	3.34E-17	10	Lmnb2
0.863057846	5.00E-13	3.75E-17	10	Iqcb1
1.934056487	5.17E-13	3.87E-17	10	Fhl1
1.063922586	6.13E-13	4.59E-17	10	Vrk3

1.000136878	6.36E-13	4.77E-17	10	Rnf215
0.448922054	7.34E-13	5.50E-17	10	Snora65
0.501210165	7.61E-13	5.70E-17	10	Ddx28
1.449822834	7.64E-13	5.73E-17	10	Flrt3
0.828743947	9.10E-13	6.82E-17	10	Lmcd1
2.508920316	1.05E-12	7.84E-17	10	Mki67
0.690049419	1.05E-12	7.90E-17	10	Oraov1
1.397618666	1.20E-12	8.99E-17	10	Tspan5
1.043659832	1.29E-12	9.63E-17	10	Lin54
0.36042391	1.34E-12	1.00E-16	10	Slc24a1
1.359439274	1.45E-12	1.09E-16	10	Wash
1.261951022	1.58E-12	1.18E-16	10	Nxt1
1.18954305	1.62E-12	1.21E-16	10	Cd99l2
0.960398367	1.67E-12	1.25E-16	10	Tgfbr3
0.881878816	1.98E-12	1.49E-16	10	Kri1
0.865097902	2.04E-12	1.53E-16	10	Tmem181a
1.128537874	2.35E-12	1.76E-16	10	Rbm3
0.638218262	2.41E-12	1.80E-16	10	Pdp2
1.274786849	2.42E-12	1.82E-16	10	Ftsj1
0.914580982	2.51E-12	1.88E-16	10	Cnot11
0.840382723	2.63E-12	1.97E-16	10	2210016L21Rik
1.1491898	2.77E-12	2.07E-16	10	Ciita
1.297592404	2.82E-12	2.11E-16	10	Dkk3
1.348779894	3.03E-12	2.27E-16	10	Fgd6
0.799300082	3.27E-12	2.45E-16	10	Taf11
1.372776671	3.37E-12	2.53E-16	10	Lcorl
0.760553613	3.61E-12	2.70E-16	10	Ints10
0.914575171	3.65E-12	2.73E-16	10	Vps8
0.735651969	4.03E-12	3.02E-16	10	Fam167a
1.299981331	4.37E-12	3.27E-16	10	Zfp260
1.076713781	4.71E-12	3.53E-16	10	Dgcr8
1.023527955	5.13E-12	3.84E-16	10	Prrc1
0.636378624	5.24E-12	3.93E-16	10	2310010J17Rik
0.702300668	5.83E-12	4.36E-16	10	Smim5
1.65059163	5.84E-12	4.38E-16	10	Fam45a
0.521947189	6.00E-12	4.49E-16	10	Nsmf
0.731701449	6.13E-12	4.59E-16	10	Mad2l1bp
0.584810329	6.30E-12	4.72E-16	10	Cxx1a
0.641302888	7.11E-12	5.33E-16	10	Nrip1
2.050232083	7.13E-12	5.34E-16	10	F10
0.370949671	7.59E-12	5.69E-16	10	4921531C22Rik
1.384584611	7.64E-12	5.72E-16	10	Rnf103
0.465126652	7.67E-12	5.74E-16	10	4930578M01Rik
0.89093925	8.06E-12	6.03E-16	10	Atad3a
0.771082406	8.31E-12	6.23E-16	10	E2f6
1.229948291	8.83E-12	6.61E-16	10	Zfp52
1.683891112	9.07E-12	6.80E-16	10	Cd274
1.052295066	9.40E-12	7.04E-16	10	Exoc7
1.436688375	9.50E-12	7.12E-16	10	St3gal2
1.286430094	9.74E-12	7.29E-16	10	Ripk3
0.671608827	1.03E-11	7.69E-16	10	4930430F08Rik
1.204113039	1.03E-11	7.70E-16	10	Abcf2
0.788323201	1.10E-11	8.24E-16	10	Ift57
1.185043732	1.22E-11	9.17E-16	10	Jmjd8
0.933397948	1.25E-11	9.33E-16	10	Alkbh2
1.109410974	1.31E-11	9.81E-16	10	Pik3r6
1.416581076	1.46E-11	1.09E-15	10	Atp13a1
0.828181998	1.54E-11	1.15E-15	10	Sec61a2
1.121128739	1.54E-11	1.15E-15	10	Phyhd1
1.224388657	1.77E-11	1.32E-15	10	Ezh2
0.958014575	1.77E-11	1.33E-15	10	Cd5l

0.848021947	1.87E-11	1.40E-15	10	Kdm4c
0.625343379	1.88E-11	1.41E-15	10	Tmem216
1.158767094	2.07E-11	1.55E-15	10	Otud6b
1.595848118	2.12E-11	1.59E-15	10	Prkd3
1.577380368	2.12E-11	1.59E-15	10	Gmps
1.533741183	2.18E-11	1.63E-15	10	Zmym4
1.123529014	2.30E-11	1.72E-15	10	Dennd6a
0.680940517	2.36E-11	1.77E-15	10	Metrn
0.758593411	2.40E-11	1.80E-15	10	Kntc1
1.304056263	2.48E-11	1.86E-15	10	Tmem175
0.946180118	2.50E-11	1.87E-15	10	Ngrn
1.694845526	2.50E-11	1.88E-15	10	Cep63
0.686275242	2.55E-11	1.91E-15	10	F2r
1.261131218	2.61E-11	1.95E-15	10	Pdzd11
0.484482951	2.87E-11	2.15E-15	10	Yae1d1
0.56710646	3.05E-11	2.29E-15	10	Zfp367
1.584616321	3.30E-11	2.47E-15	10	Yars
1.346482107	3.38E-11	2.53E-15	10	Gdf3
0.875685107	3.39E-11	2.54E-15	10	Tug1
0.460947768	3.51E-11	2.63E-15	10	Ghdc
0.691564723	3.55E-11	2.66E-15	10	Il1rap
0.522481056	4.08E-11	3.06E-15	10	Pex11g
0.96511455	4.24E-11	3.17E-15	10	Trappc9
1.790906661	4.41E-11	3.30E-15	10	Arid5b
1.242168402	4.54E-11	3.40E-15	10	Wdfy1
1.422268649	4.77E-11	3.57E-15	10	Eci2
2.248650116	5.31E-11	3.98E-15	10	Top2a
1.686188378	6.06E-11	4.54E-15	10	Pmm2
0.936176842	6.16E-11	4.61E-15	10	Rpl9
0.67630267	6.77E-11	5.07E-15	10	Acaca
0.620119526	7.69E-11	5.76E-15	10	Snhg4
1.440430182	8.02E-11	6.00E-15	10	Prpf38a
1.01319768	8.11E-11	6.07E-15	10	Tmem41b
1.118498398	8.18E-11	6.13E-15	10	Atg14
0.584810329	8.66E-11	6.49E-15	10	Cbwd1
1.553883348	8.74E-11	6.55E-15	10	Ccdc132
1.381885111	9.01E-11	6.75E-15	10	Smarcc1
1.211527233	9.23E-11	6.91E-15	10	Ttf1
0.680589003	9.37E-11	7.02E-15	10	C920021L13Rik
0.742382155	9.75E-11	7.30E-15	10	Med20
1.313344831	1.03E-10	7.74E-15	10	Wdr45
0.419749336	1.16E-10	8.65E-15	10	Lphn1
0.882176499	1.36E-10	1.02E-14	10	Efna5
0.999172648	1.44E-10	1.08E-14	10	Mrps5
1.940974662	1.47E-10	1.10E-14	10	Cdc25a
0.793337482	1.67E-10	1.25E-14	10	Tlcd1
1.395122623	1.69E-10	1.27E-14	10	Rapgef1
1.236397614	1.75E-10	1.31E-14	10	2210015D19Rik
0.469316468	1.80E-10	1.35E-14	10	Ube2t
0.773443299	1.82E-10	1.36E-14	10	Fktn
0.889233366	1.87E-10	1.40E-14	10	Mylk
0.784793905	1.93E-10	1.45E-14	10	Kif23
1.12544752	1.98E-10	1.48E-14	10	2810013P06Rik
0.963768331	2.05E-10	1.54E-14	10	Fam122a
0.37819608	2.13E-10	1.59E-14	10	Gpatch2
1.458070299	2.24E-10	1.67E-14	10	Mrps11
1.332556437	2.25E-10	1.68E-14	10	Rbm34
0.622903809	2.26E-10	1.70E-14	10	Tada2a
0.696173734	2.40E-10	1.80E-14	10	Bloc1s4
0.49175078	2.66E-10	2.00E-14	10	Bend3
1.813833485	2.69E-10	2.02E-14	10	Parp2

1.695563415	2.92E-10	2.18E-14	10	Lmf2
0.733424452	2.96E-10	2.22E-14	10	Med24
0.857329696	3.28E-10	2.45E-14	10	Dnajb12
0.783661188	3.43E-10	2.57E-14	10	Xab2
0.517794293	3.60E-10	2.69E-14	10	Dolpp1
0.816309607	3.75E-10	2.81E-14	10	Lphn2
0.609886692	3.83E-10	2.87E-14	10	Jade3
1.72471247	3.86E-10	2.89E-14	10	Arfrp1
1.053816594	3.86E-10	2.89E-14	10	Klhl18
1.122582016	3.90E-10	2.92E-14	10	Zfp593
0.582845049	3.92E-10	2.94E-14	10	Igfbp6
0.905556114	4.13E-10	3.09E-14	10	Deb1
0.434503792	4.46E-10	3.34E-14	10	Creg2
1.568375562	4.57E-10	3.42E-14	10	Iah1
0.964445466	4.66E-10	3.49E-14	10	Phxr4
1.727488078	4.73E-10	3.54E-14	10	Mapkap1
0.539582753	5.08E-10	3.81E-14	10	Aplf
1.076185885	5.64E-10	4.22E-14	10	Smarcd1
1.340015824	5.66E-10	4.24E-14	10	Araf
1.052342135	6.48E-10	4.85E-14	10	Lrif1
1.328864101	6.56E-10	4.91E-14	10	Mad2l2
1.319051719	6.74E-10	5.05E-14	10	Sec24a
1.334617262	6.87E-10	5.15E-14	10	Naglu
0.852173983	7.44E-10	5.57E-14	10	1810026J23Rik
0.822706307	7.70E-10	5.77E-14	10	Usp22
1.13697002	7.98E-10	5.97E-14	10	Tmem198b
1.092287133	8.02E-10	6.01E-14	10	Eaf1
1.056749416	8.05E-10	6.03E-14	10	Ptgs2
1.109360204	8.10E-10	6.06E-14	10	Hps4
1.546236875	8.34E-10	6.25E-14	10	Ltbr
0.549907458	8.41E-10	6.30E-14	10	Lrrc27
1.039065602	8.47E-10	6.34E-14	10	Syvn1
1.282324783	8.56E-10	6.41E-14	10	Eif1ad
1.235409233	8.78E-10	6.57E-14	10	Dohh
1.425339379	9.31E-10	6.97E-14	10	AA467197
0.690842147	9.49E-10	7.11E-14	10	Snip1
1.169323669	9.62E-10	7.20E-14	10	Xrn1
1.405443882	9.80E-10	7.34E-14	10	Exosc9
0.567178066	9.87E-10	7.39E-14	10	Otud3
1.107356945	1.00E-09	7.52E-14	10	Mettl6
1.43209039	1.04E-09	7.78E-14	10	Hbs1l
0.819288366	1.04E-09	7.78E-14	10	Cdo1
0.889411643	1.05E-09	7.85E-14	10	Endov
0.90180143	1.07E-09	8.02E-14	10	Ankrd13c
1.178654996	1.10E-09	8.25E-14	10	Tssc1
1.016347332	1.27E-09	9.52E-14	10	Pml
1.010459479	1.28E-09	9.55E-14	10	Dcaf13
1.470840464	1.37E-09	1.03E-13	10	Man2a2
1.175365983	1.40E-09	1.05E-13	10	Phkb
1.015172989	1.41E-09	1.05E-13	10	Ndc1
0.936521035	1.58E-09	1.19E-13	10	Cops7a
0.538514907	1.70E-09	1.28E-13	10	Mpi
0.503803766	1.82E-09	1.37E-13	10	Cstf2t
0.871360763	1.88E-09	1.40E-13	10	Sephs1
1.334803883	1.90E-09	1.42E-13	10	Slc39a6
0.91681726	1.94E-09	1.46E-13	10	Cbll1
1.367200007	2.02E-09	1.52E-13	10	Aars
0.551828227	2.17E-09	1.63E-13	10	Disc1
0.689125002	2.19E-09	1.64E-13	10	Ccdc167
0.764505182	2.19E-09	1.64E-13	10	Men1
1.006804739	2.20E-09	1.65E-13	10	Mis18a

1.277417213	2.25E-09	1.69E-13	10	Ppap2c
0.750935245	2.32E-09	1.74E-13	10	1600010M07Rik
1.318022254	2.51E-09	1.88E-13	10	Fopnl
0.526041891	2.54E-09	1.90E-13	10	2410004N09Rik
0.78235769	2.64E-09	1.98E-13	10	Socs2
1.379563167	2.69E-09	2.02E-13	10	Ap5s1
0.361198504	2.72E-09	2.04E-13	10	Actg2
0.814839906	2.76E-09	2.07E-13	10	Lyrm9
1.334954044	2.77E-09	2.07E-13	10	Mllt4
1.090015637	2.88E-09	2.15E-13	10	Ticam1
1.485611947	3.00E-09	2.24E-13	10	Slc35b1
0.51161801	3.17E-09	2.37E-13	10	Slc17a5
1.232928919	3.31E-09	2.48E-13	10	B4galt3
1.295794857	3.41E-09	2.56E-13	10	Nfya
1.542571959	3.49E-09	2.61E-13	10	Tcof1
0.772426821	3.50E-09	2.62E-13	10	Dym
0.751193998	3.57E-09	2.68E-13	10	Arntl
0.987867538	3.82E-09	2.86E-13	10	Sh3pxd2a
1.106759837	4.25E-09	3.18E-13	10	Fgd4
0.691021237	4.54E-09	3.40E-13	10	Dcaf4
0.936680433	4.74E-09	3.55E-13	10	Slco3a1
0.743920859	5.84E-09	4.37E-13	10	Tysnd1
0.466199685	5.84E-09	4.37E-13	10	Tyms-ps
1.203934189	6.18E-09	4.63E-13	10	Aim2
0.552378804	6.38E-09	4.78E-13	10	Pmf1
1.327899366	6.41E-09	4.80E-13	10	Pcp4l1
0.790201045	6.60E-09	4.94E-13	10	Bin3
0.774389237	8.04E-09	6.02E-13	10	Steap3
0.652632926	8.38E-09	6.27E-13	10	Sox7
1.226679828	8.76E-09	6.56E-13	10	Gpr107
0.435734073	8.95E-09	6.71E-13	10	Rnf144b
1.166463696	9.48E-09	7.10E-13	10	Scaf8
1.014622101	9.84E-09	7.37E-13	10	Tmem203
0.401123585	1.01E-08	7.60E-13	10	Aurka
0.895650231	1.05E-08	7.89E-13	10	Ang
1.118357225	1.07E-08	7.98E-13	10	Snhg5
1.394180483	1.09E-08	8.13E-13	10	Cenpa
0.372778682	1.09E-08	8.20E-13	10	Rbmx2
1.144079393	1.14E-08	8.51E-13	10	Sec23b
0.948332971	1.20E-08	8.98E-13	10	Micu1
1.204016265	1.21E-08	9.09E-13	10	Kif1c
0.646336997	1.28E-08	9.57E-13	10	Ppil1
0.492892974	1.29E-08	9.63E-13	10	E130102H24Rik
0.730957125	1.30E-08	9.72E-13	10	Foxj3
1.367334544	1.31E-08	9.79E-13	10	Sec13
1.494477058	1.34E-08	1.00E-12	10	Prdm2
0.958875531	1.34E-08	1.00E-12	10	Ppfibp2
1.239769085	1.42E-08	1.06E-12	10	Ccrn4l
1.31545194	1.45E-08	1.08E-12	10	Ccdc25
0.563187865	1.49E-08	1.12E-12	10	Ccnyl1
1.003401446	1.55E-08	1.16E-12	10	Mecr
0.63754268	1.63E-08	1.22E-12	10	Mdm4
1.22673182	1.68E-08	1.26E-12	10	Farsb
0.83114011	1.73E-08	1.30E-12	10	Rnf40
1.058947291	1.75E-08	1.31E-12	10	Tmem71
0.865670741	1.76E-08	1.32E-12	10	1700112E06Rik
1.069285758	1.84E-08	1.37E-12	10	Cdk10
1.613584428	1.86E-08	1.39E-12	10	Gtf2f1
1.241140831	1.94E-08	1.45E-12	10	Thoc6
0.789859218	1.95E-08	1.46E-12	10	Lime1
1.05242426	1.96E-08	1.47E-12	10	Prmt10

0.704161614	1.98E-08	1.48E-12	10	Nrf1
1.287028067	2.01E-08	1.51E-12	10	Khk
0.878378951	2.02E-08	1.51E-12	10	Cwc27
0.765095857	2.10E-08	1.57E-12	10	Nln
1.309865504	2.21E-08	1.66E-12	10	Tmem120a
0.877648714	2.27E-08	1.70E-12	10	Chfr
1.634336393	2.28E-08	1.71E-12	10	Malt1
0.953263972	2.37E-08	1.78E-12	10	Ago3
1.319335114	2.51E-08	1.88E-12	10	Ube2d2a
0.494273254	2.54E-08	1.90E-12	10	Dgke
0.322790042	2.58E-08	1.93E-12	10	Tex9
0.685808788	2.61E-08	1.95E-12	10	Ccdc28a
0.280867736	2.61E-08	1.96E-12	10	Nup93
0.693322021	2.62E-08	1.96E-12	10	Gm5617
1.301059424	2.72E-08	2.04E-12	10	Tubb4b
1.150903904	2.89E-08	2.16E-12	10	2300009A05Rik
1.21836962	2.92E-08	2.19E-12	10	Opa1
1.415789548	3.10E-08	2.32E-12	10	Nfatc1
1.223480183	3.16E-08	2.36E-12	10	Vps37c
0.58181816	3.30E-08	2.47E-12	10	Hes6
0.641004888	3.31E-08	2.48E-12	10	Cdk1
1.255945562	3.37E-08	2.52E-12	10	Cks1b
0.625691722	3.53E-08	2.64E-12	10	Tmem184b
1.153924976	3.54E-08	2.65E-12	10	Pogz
0.54904389	3.57E-08	2.67E-12	10	Lnp
0.890093228	3.57E-08	2.67E-12	10	Matk
0.746389793	3.65E-08	2.74E-12	10	Kctd6
0.390888907	3.68E-08	2.76E-12	10	Fgfrl1
0.57356794	3.80E-08	2.84E-12	10	E130309D02Rik
1.307675521	3.95E-08	2.96E-12	10	Tmem214
1.854379954	4.05E-08	3.03E-12	10	Atg2a
0.674570795	4.11E-08	3.08E-12	10	Ttc39c
0.864115966	4.13E-08	3.09E-12	10	Get4
0.622393041	4.20E-08	3.14E-12	10	Zfp944
0.722700488	4.25E-08	3.18E-12	10	Ncam1
1.38390873	4.28E-08	3.20E-12	10	Inf2
0.847559318	4.30E-08	3.22E-12	10	Wdr81
0.607264321	4.36E-08	3.27E-12	10	Ubr7
1.363137159	4.46E-08	3.34E-12	10	Pofut2
1.261350188	4.49E-08	3.36E-12	10	Vac14
1.481191719	4.89E-08	3.66E-12	10	Fam20c
0.696621321	5.02E-08	3.76E-12	10	Sephs2
0.832600617	5.14E-08	3.85E-12	10	Umps
0.762134558	5.23E-08	3.92E-12	10	Srebf1
1.193554692	5.28E-08	3.95E-12	10	Slc4a7
1.292646885	5.34E-08	4.00E-12	10	Mlec
0.573554631	5.40E-08	4.04E-12	10	Cog8
1.468359314	5.48E-08	4.10E-12	10	Kcnq1ot1
1.362682807	5.48E-08	4.10E-12	10	Gadd45gip1
0.358393453	5.76E-08	4.31E-12	10	Dnaaf3
0.872900282	5.76E-08	4.32E-12	10	Rtn4ip1
1.171792371	6.07E-08	4.55E-12	10	Timm10
0.413671768	6.12E-08	4.59E-12	10	Rapsn
0.918076624	6.26E-08	4.69E-12	10	Nusap1
1.02026121	6.39E-08	4.78E-12	10	Ankib1
0.848093048	6.44E-08	4.82E-12	10	AU019823
0.474377387	6.73E-08	5.04E-12	10	Eci1
0.752978848	6.79E-08	5.08E-12	10	Wee1
1.107001391	6.99E-08	5.23E-12	10	Zrsr2
0.955985491	7.33E-08	5.49E-12	10	Osgin2
1.001530213	7.46E-08	5.59E-12	10	Vps39

1.398408354	7.49E-08	5.61E-12	10	Pdxk
0.385238363	7.67E-08	5.75E-12	10	Eid2b
1.376868375	8.44E-08	6.32E-12	10	Apoa1bp
0.926762032	8.53E-08	6.39E-12	10	Bet1l
0.977432661	8.68E-08	6.50E-12	10	Ubqln2
1.075278265	8.86E-08	6.63E-12	10	Sec24c
0.757816246	9.12E-08	6.83E-12	10	Lamb2
1.17584944	9.13E-08	6.84E-12	10	Mrpl37
0.933294953	9.59E-08	7.18E-12	10	Keap1
0.827795541	9.84E-08	7.37E-12	10	Rrm1
0.872258904	1.06E-07	7.92E-12	10	Zfyve19
0.977561718	1.07E-07	8.02E-12	10	Ppp2r1b
1.129463774	1.12E-07	8.41E-12	10	Irf3
1.383152449	1.20E-07	8.97E-12	10	Mrps17
0.670884938	1.22E-07	9.14E-12	10	Blmh
1.197338058	1.23E-07	9.18E-12	10	Msl3
1.223527444	1.27E-07	9.48E-12	10	Ralgds
0.835075482	1.29E-07	9.65E-12	10	Coro7
0.920274247	1.30E-07	9.73E-12	10	Ino80
0.84601663	1.30E-07	9.77E-12	10	Etfdh
1.129086133	1.31E-07	9.81E-12	10	Tmem248
0.622903809	1.38E-07	1.04E-11	10	Gnl3l
0.691696115	1.43E-07	1.07E-11	10	Kdm4b
0.711008356	1.46E-07	1.09E-11	10	Zscan26
0.492974784	1.49E-07	1.11E-11	10	Mcemp1
1.241406086	1.53E-07	1.14E-11	10	Gfpt1
1.276750301	1.53E-07	1.14E-11	10	Chchd3
0.602476641	1.64E-07	1.23E-11	10	Fam207a
1.385594557	1.72E-07	1.29E-11	10	Nfkb2
0.65389978	1.75E-07	1.31E-11	10	Ptrh2
1.324729575	1.78E-07	1.34E-11	10	Pnpla2
0.72576971	1.79E-07	1.34E-11	10	Dbf4
1.077073935	1.83E-07	1.37E-11	10	Asxl1
0.871179022	1.88E-07	1.41E-11	10	Reep4
0.478016415	1.88E-07	1.41E-11	10	Mfn2
1.056036177	1.89E-07	1.42E-11	10	Cipc
1.166865457	1.91E-07	1.43E-11	10	Trap1
0.911883135	2.02E-07	1.51E-11	10	Haus2
0.482921876	2.06E-07	1.54E-11	10	Coq5
0.769151579	2.08E-07	1.56E-11	10	Fem1b
0.924062975	2.14E-07	1.61E-11	10	Pcnt
0.455557201	2.15E-07	1.61E-11	10	Adck4
0.937198559	2.18E-07	1.63E-11	10	Cdc42bpb
1.461637982	2.31E-07	1.73E-11	10	Nsmce2
0.883834236	2.40E-07	1.80E-11	10	Prps1
0.928727956	2.44E-07	1.83E-11	10	Ubqln4
0.429437006	2.47E-07	1.85E-11	10	Dcaf17
0.52206645	2.51E-07	1.88E-11	10	Slc12a4
0.459721368	2.56E-07	1.92E-11	10	Slc41a1
0.723821188	2.64E-07	1.97E-11	10	Inpp5a
0.470028443	2.66E-07	1.99E-11	10	Snapc2
1.304139396	2.72E-07	2.04E-11	10	9930021J03Rik
1.172640945	2.80E-07	2.10E-11	10	Lpcat3
1.157627629	2.89E-07	2.17E-11	10	Srpr
0.42691545	2.92E-07	2.19E-11	10	Pbx3
0.965065133	2.98E-07	2.23E-11	10	Tspan2
0.749550823	2.99E-07	2.24E-11	10	Narfl
1.080242961	3.09E-07	2.31E-11	10	Traf1
1.145274414	3.23E-07	2.42E-11	10	Phospho2
1.235617597	3.25E-07	2.43E-11	10	Babam1
0.612787017	3.26E-07	2.44E-11	10	Trip10

1.048639979	3.27E-07	2.45E-11	10	Scd1
1.094762018	3.31E-07	2.48E-11	10	Dnajb14
0.596371152	3.33E-07	2.49E-11	10	Psmg3
0.931089872	3.48E-07	2.60E-11	10	Mthfd2
0.915947384	3.58E-07	2.68E-11	10	Katnbl1
1.177977409	3.59E-07	2.69E-11	10	Lrp12
0.917943223	3.66E-07	2.74E-11	10	Fam20b
0.557947031	3.70E-07	2.77E-11	10	Prpf3
0.877860874	3.72E-07	2.78E-11	10	Sec16a
0.964759963	3.72E-07	2.79E-11	10	Inpp5b
0.888459274	3.76E-07	2.81E-11	10	Oxsr1
0.582964885	3.80E-07	2.84E-11	10	Ramp3
0.707307496	3.90E-07	2.92E-11	10	Smg6
0.630118596	3.96E-07	2.97E-11	10	Nfyb
1.016328621	3.97E-07	2.98E-11	10	Dnajc4
0.831147854	4.05E-07	3.04E-11	10	Fez2
1.324478775	4.11E-07	3.08E-11	10	Mrpl17
0.287382417	4.25E-07	3.19E-11	10	Kitl
1.074872798	4.28E-07	3.21E-11	10	Cdc42ep4
0.536453948	4.33E-07	3.24E-11	10	Rab13
1.315927143	4.36E-07	3.26E-11	10	Ehd1
0.828073851	4.47E-07	3.34E-11	10	Rab5b
0.646531625	4.53E-07	3.39E-11	10	Akt3
1.311682154	4.59E-07	3.44E-11	10	Mybbp1a
1.327796052	4.64E-07	3.48E-11	10	Zfp704
0.752570601	4.72E-07	3.53E-11	10	Ints1
1.342882317	4.80E-07	3.60E-11	10	Pgs1
0.402488773	5.02E-07	3.76E-11	10	Retsat
0.890732568	5.03E-07	3.77E-11	10	Scaper
1.209560941	5.47E-07	4.09E-11	10	Tmppe
0.345148226	5.48E-07	4.11E-11	10	Nod2
1.081214416	5.58E-07	4.18E-11	10	Pdzd8
1.023425621	5.65E-07	4.23E-11	10	Tusc2
1.118546898	6.41E-07	4.80E-11	10	Ahr
1.297620271	6.54E-07	4.89E-11	10	Csf2rb
1.029499349	6.62E-07	4.96E-11	10	Timp1
0.585644242	6.63E-07	4.97E-11	10	Gmnn
0.961373665	7.00E-07	5.24E-11	10	Slc25a10
0.781589262	7.13E-07	5.34E-11	10	Gatad2b
0.472973069	7.13E-07	5.34E-11	10	Tyms
0.848364915	7.20E-07	5.39E-11	10	Mina
1.010487985	7.23E-07	5.41E-11	10	Aebp1
0.565392243	7.50E-07	5.62E-11	10	Zwint
1.002746002	8.08E-07	6.05E-11	10	Cmc2
0.665332492	8.18E-07	6.13E-11	10	Ddb2
0.360066839	8.30E-07	6.21E-11	10	Chst14
0.999009117	8.36E-07	6.26E-11	10	Osbpl1a
0.834492458	8.37E-07	6.27E-11	10	Crat
1.002646729	9.15E-07	6.85E-11	10	Suds3
0.628796478	1.00E-06	7.50E-11	10	Slc35a1
0.37819608	1.02E-06	7.65E-11	10	Engase
2.170894957	1.05E-06	7.85E-11	10	Eln
0.469695984	1.06E-06	7.92E-11	10	Nme4
1.040314518	1.09E-06	8.14E-11	10	Ranbp10
1.336447888	1.09E-06	8.16E-11	10	Arl6ip5
1.051256502	1.09E-06	8.20E-11	10	Ppil4
0.698602937	1.17E-06	8.73E-11	10	Rptor
1.067578775	1.18E-06	8.85E-11	10	Ercc1
0.503775385	1.20E-06	8.99E-11	10	6430548M08Rik
0.75734388	1.21E-06	9.06E-11	10	Ckap5
1.086792383	1.25E-06	9.38E-11	10	Srprb

1.163308613	1.27E-06	9.48E-11	10	Cd151
1.181734329	1.28E-06	9.58E-11	10	Trpc4ap
0.969187876	1.36E-06	1.02E-10	10	Prosc
0.829583376	1.38E-06	1.03E-10	10	1700037H04Rik
0.760299838	1.40E-06	1.05E-10	10	Pop4
1.097353545	1.47E-06	1.10E-10	10	Cyhr1
0.941201444	1.48E-06	1.11E-10	10	9030617O03Rik
1.23973119	1.52E-06	1.14E-10	10	Abcc5
0.44149093	1.59E-06	1.19E-10	10	Ubiad1
0.412590364	1.62E-06	1.21E-10	10	Nmrk1
1.330453566	1.65E-06	1.24E-10	10	Il17ra
0.740268587	1.69E-06	1.26E-10	10	Msh2
1.248082981	1.72E-06	1.29E-10	10	Sbno1
1.289055578	1.76E-06	1.32E-10	10	Tbk1
0.543451966	1.88E-06	1.41E-10	10	B9d2
0.476176488	1.94E-06	1.45E-10	10	Mpp7
1.323968371	1.94E-06	1.46E-10	10	Dirc2
0.453630198	1.98E-06	1.48E-10	10	Tmed1
0.947556159	2.01E-06	1.50E-10	10	D16Ertd472e
1.108625428	2.02E-06	1.52E-10	10	Hivep3
0.873754232	2.07E-06	1.55E-10	10	Arl13b
1.15139828	2.11E-06	1.58E-10	10	Arl5c
0.409637606	2.17E-06	1.63E-10	10	Lactb2
1.152337688	2.19E-06	1.64E-10	10	Ndufs8
1.114089443	2.26E-06	1.69E-10	10	Dap3
0.699479899	2.36E-06	1.77E-10	10	Cryab
0.672318601	2.37E-06	1.78E-10	10	Zfp346
1.05789717	2.53E-06	1.90E-10	10	Rab22a
0.929171464	2.54E-06	1.90E-10	10	Tbc1d13
1.13265595	2.57E-06	1.92E-10	10	Ubn1
0.832313431	2.62E-06	1.96E-10	10	Bckdk
1.212182513	2.67E-06	2.00E-10	10	Pik3r1
0.593820799	2.68E-06	2.01E-10	10	Tcp11l1
1.097705181	2.72E-06	2.04E-10	10	Tmem259
0.976706708	2.77E-06	2.07E-10	10	Ercc6
0.792741841	2.79E-06	2.09E-10	10	Nf2
1.265849582	2.81E-06	2.10E-10	10	Eif2s3y
1.042834203	2.95E-06	2.21E-10	10	Mrpl55
1.293477221	2.99E-06	2.24E-10	10	Gtpbp4
0.313083011	3.12E-06	2.34E-10	10	Cdc42ep3
1.147642101	3.16E-06	2.37E-10	10	Sae1
0.800694321	3.17E-06	2.37E-10	10	Zc3h12d
1.451209193	3.18E-06	2.38E-10	10	Tgfbrap1
0.993069711	3.32E-06	2.49E-10	10	Polr1c
0.372535563	3.33E-06	2.50E-10	10	Rnf167
0.778862593	3.35E-06	2.51E-10	10	Cdc27
0.65442472	3.35E-06	2.51E-10	10	Lsm14b
0.753531447	3.42E-06	2.56E-10	10	Fbxl20
0.943865508	3.42E-06	2.56E-10	10	Pom121
1.114649167	3.57E-06	2.68E-10	10	Hck
1.039462841	3.59E-06	2.69E-10	10	Trim27
0.691416007	3.65E-06	2.74E-10	10	Med17
0.977625681	3.79E-06	2.84E-10	10	Zfp281
0.375123881	3.84E-06	2.88E-10	10	Arhgap19
0.862454676	3.88E-06	2.91E-10	10	Itgb1bp1
0.384316466	4.03E-06	3.02E-10	10	Top3a
0.683695731	4.13E-06	3.09E-10	10	Fbxw2
0.569702546	4.21E-06	3.15E-10	10	Sp2
0.535551891	4.22E-06	3.16E-10	10	Lzts2
1.651404327	4.29E-06	3.21E-10	10	Ogfr
0.843633038	4.52E-06	3.39E-10	10	Xpnpep1

0.368330377	4.59E-06	3.44E-10	10	Nde1
0.815301545	4.70E-06	3.52E-10	10	Esrra
0.758013538	4.77E-06	3.57E-10	10	Ampd2
0.683071529	4.80E-06	3.60E-10	10	Nckipsd
1.170488121	5.04E-06	3.77E-10	10	Cyc1
0.795798035	5.22E-06	3.91E-10	10	Srp68
1.287246107	5.25E-06	3.93E-10	10	Surf2
0.885448225	5.39E-06	4.04E-10	10	Specc1l
1.154070572	5.52E-06	4.14E-10	10	Odc1
1.113740945	5.74E-06	4.30E-10	10	Ubr3
0.569087587	5.86E-06	4.39E-10	10	Tusc1
0.529114677	5.89E-06	4.41E-10	10	Eya3
1.17498025	5.95E-06	4.45E-10	10	Ccng1
0.436382362	5.97E-06	4.47E-10	10	Cks2
1.044367841	6.02E-06	4.51E-10	10	Kdm5a
0.350296242	6.05E-06	4.53E-10	10	Stat5a
0.649068412	6.10E-06	4.57E-10	10	Extl2
1.308085538	6.12E-06	4.58E-10	10	Il1r2
0.861781059	6.18E-06	4.63E-10	10	Tbc1d1
1.155990911	6.20E-06	4.64E-10	10	Stx3
0.701174468	6.23E-06	4.67E-10	10	Fbxo28
0.761877708	6.30E-06	4.72E-10	10	Sdccag8
0.378566519	6.33E-06	4.74E-10	10	Rrp9
0.766936956	6.45E-06	4.83E-10	10	Zcchc17
0.403980197	6.53E-06	4.89E-10	10	Cntln
0.405224752	6.64E-06	4.97E-10	10	Kin
0.472666033	6.68E-06	5.00E-10	10	Ltn1
0.664279431	6.73E-06	5.04E-10	10	Pik3ap1
0.766971963	6.80E-06	5.09E-10	10	Ptgr1
0.935196244	6.89E-06	5.16E-10	10	Med1
1.07380876	6.95E-06	5.20E-10	10	Actn4
0.361561019	7.21E-06	5.40E-10	10	Chil3
1.149195689	7.24E-06	5.42E-10	10	Eif3l
1.006230192	7.35E-06	5.50E-10	10	Ptrf
0.643230573	7.36E-06	5.51E-10	10	Pigyl
1.138485092	7.41E-06	5.55E-10	10	Lxn
0.345590606	7.65E-06	5.73E-10	10	Zfp738
0.742391294	7.72E-06	5.78E-10	10	5730409E04Rik
0.72029983	8.44E-06	6.32E-10	10	Ing1
0.599134559	8.59E-06	6.43E-10	10	Nelfe
0.578721543	8.74E-06	6.55E-10	10	Bcl2l13
0.732802771	8.87E-06	6.64E-10	10	Il1a
1.187226463	9.38E-06	7.03E-10	10	Traf3
0.962679935	9.78E-06	7.32E-10	10	Snx4
1.153115859	9.80E-06	7.34E-10	10	Copb2
0.253621345	1.01E-05	7.60E-10	10	Pla2g12a
0.351878772	1.05E-05	7.88E-10	10	Mib1
1.213473454	1.06E-05	7.91E-10	10	Exoc5
0.571780829	1.13E-05	8.50E-10	10	Smtn
0.702927981	1.16E-05	8.71E-10	10	Snx24
0.460446083	1.18E-05	8.80E-10	10	Ylpm1
0.468363949	1.18E-05	8.82E-10	10	Mta1
0.407077634	1.19E-05	8.89E-10	10	Mir6516
0.575538985	1.26E-05	9.44E-10	10	Med25
1.085858879	1.26E-05	9.46E-10	10	Rbm18
1.033446425	1.26E-05	9.47E-10	10	Zfp655
1.132264401	1.30E-05	9.71E-10	10	Timm17a
0.736375915	1.32E-05	9.86E-10	10	Cd276
0.534337043	1.35E-05	1.01E-09	10	Fam65a
0.900437536	1.35E-05	1.01E-09	10	Tnpo3
0.921076236	1.35E-05	1.01E-09	10	Arid3a

0.761475263	1.38E-05	1.03E-09	10	Skiv2l2
1.016366928	1.39E-05	1.04E-09	10	Mea1
0.476845161	1.40E-05	1.05E-09	10	Ogn
0.665487118	1.43E-05	1.07E-09	10	Rab10os
0.472506759	1.45E-05	1.08E-09	10	Wdyhv1
0.992273155	1.51E-05	1.13E-09	10	Piezo1
0.961022096	1.52E-05	1.14E-09	10	Trim26
0.693105375	1.64E-05	1.23E-09	10	Fert2
0.474195636	1.70E-05	1.27E-09	10	Arg2
0.709694161	1.72E-05	1.29E-09	10	Ss18
0.517291578	1.75E-05	1.31E-09	10	Gm5801
0.661631862	1.75E-05	1.31E-09	10	Dtymk
0.734138691	1.76E-05	1.31E-09	10	Bzw2
0.626207698	1.81E-05	1.36E-09	10	Frs2
0.857071663	1.83E-05	1.37E-09	10	Prkch
1.238833354	1.83E-05	1.37E-09	10	Hsd17b10
1.121538992	1.85E-05	1.38E-09	10	Tns1
0.85789918	1.89E-05	1.42E-09	10	Kifap3
0.4802617	1.90E-05	1.43E-09	10	Saysd1
0.467524188	1.91E-05	1.43E-09	10	Clcn5
0.663620386	1.91E-05	1.43E-09	10	Ero1lb
0.53536865	1.94E-05	1.45E-09	10	Slc25a19
1.090400099	1.99E-05	1.49E-09	10	Tnip3
1.056760837	2.06E-05	1.54E-09	10	Cd8b1
1.105244812	2.06E-05	1.55E-09	10	Med15
0.40611467	2.07E-05	1.55E-09	10	Prkag2
1.622254254	2.07E-05	1.55E-09	10	Herc2
0.293528188	2.08E-05	1.56E-09	10	Met
0.895624283	2.15E-05	1.61E-09	10	Atf2
1.185269513	2.18E-05	1.63E-09	10	Lnpep
0.421213465	2.19E-05	1.64E-09	10	Stxbp1
0.691253768	2.24E-05	1.67E-09	10	Prr14
0.890490287	2.27E-05	1.70E-09	10	Cetn2
0.69562277	2.27E-05	1.70E-09	10	Erlec1
0.844384337	2.30E-05	1.72E-09	10	Slc25a46
0.793245432	2.34E-05	1.75E-09	10	Naf1
0.640664925	2.36E-05	1.77E-09	10	Ccdc174
0.845036091	2.39E-05	1.79E-09	10	Dnajc21
0.375618759	2.39E-05	1.79E-09	10	Ppdpf
1.014588116	2.42E-05	1.81E-09	10	N4bp2l2
-0.760096118	2.43E-05	1.82E-09	10	Tmsb4x
0.9604117	2.44E-05	1.82E-09	10	Plxnc1
0.475937397	2.44E-05	1.83E-09	10	Lrpprc
0.528757703	2.49E-05	1.86E-09	10	Rlf
0.964060543	2.59E-05	1.94E-09	10	Rps6ka3
0.412468483	2.74E-05	2.05E-09	10	Enoph1
0.364950853	2.79E-05	2.09E-09	10	B3gat3
0.352316632	2.79E-05	2.09E-09	10	Fto
0.717753741	2.82E-05	2.11E-09	10	Pagr1a
0.885367257	2.83E-05	2.12E-09	10	Cdk12
1.119759954	2.87E-05	2.15E-09	10	St3gal1
0.544329377	2.91E-05	2.18E-09	10	Katna1
0.724683211	2.91E-05	2.18E-09	10	Hbegf
0.883888701	2.97E-05	2.22E-09	10	Cgrrf1
0.737323007	3.02E-05	2.26E-09	10	Lage3
0.957891377	3.02E-05	2.27E-09	10	Oxct1
0.762319862	3.06E-05	2.29E-09	10	Exosc8
0.883077347	3.09E-05	2.32E-09	10	Batf
1.012835546	3.13E-05	2.34E-09	10	Map2k3
0.796003711	3.20E-05	2.40E-09	10	Ankle2
0.597952162	3.21E-05	2.41E-09	10	Nfatc3

0.816562976	3.31E-05	2.48E-09	10	Pyurf
0.624993822	3.36E-05	2.52E-09	10	Ccndbp1
0.719122667	3.43E-05	2.57E-09	10	Exo5
1.224402258	3.44E-05	2.57E-09	10	2310035C23Rik
0.716625787	3.48E-05	2.60E-09	10	Mrps33
0.825746607	3.57E-05	2.67E-09	10	Cyth3
1.00809497	3.69E-05	2.76E-09	10	Rangap1
0.980753	3.70E-05	2.77E-09	10	Sppl3
0.728415714	3.75E-05	2.81E-09	10	Ccdc127
0.493017404	3.82E-05	2.86E-09	10	Hnrnpul1
0.987936255	3.91E-05	2.93E-09	10	Kmt2b
1.120182009	3.97E-05	2.98E-09	10	Itga6
1.031961296	4.04E-05	3.02E-09	10	Hadha
1.045866261	4.07E-05	3.05E-09	10	Rexo4
1.058585482	4.11E-05	3.08E-09	10	Plcb2
1.089254187	4.21E-05	3.15E-09	10	Commd1
1.031149311	4.33E-05	3.24E-09	10	Sntb2
0.759272041	4.52E-05	3.38E-09	10	Luc7l
0.926889713	4.55E-05	3.41E-09	10	Mmp19
0.972212637	4.76E-05	3.57E-09	10	Magt1
0.967675028	4.90E-05	3.67E-09	10	Pqbp1
0.817324956	4.97E-05	3.72E-09	10	Ift46
0.625052926	4.97E-05	3.72E-09	10	Lrmp
0.5453118	5.24E-05	3.92E-09	10	Ipo8
0.79551865	5.30E-05	3.97E-09	10	Rps6ka4
0.262768758	5.39E-05	4.03E-09	10	Dus1l
0.925579299	5.78E-05	4.33E-09	10	Commd9
0.656898212	5.83E-05	4.37E-09	10	Timm50
0.857567047	6.13E-05	4.59E-09	10	Zc3h12a
0.498717236	6.26E-05	4.69E-09	10	Mnat1
0.763858561	6.29E-05	4.71E-09	10	Abhd5
0.900304097	6.48E-05	4.85E-09	10	Stk38
0.983454491	6.49E-05	4.86E-09	10	Dnajc13
0.309025447	6.95E-05	5.20E-09	10	2410004B18Rik
0.446361137	6.95E-05	5.21E-09	10	Rcan3
0.938353531	7.03E-05	5.26E-09	10	Mknk2
0.903684494	7.05E-05	5.28E-09	10	Sptlc2
0.906470025	7.13E-05	5.34E-09	10	Ubr4
0.657598796	7.26E-05	5.44E-09	10	Nek6
0.605484462	7.93E-05	5.94E-09	10	Vcl
0.745547895	8.06E-05	6.04E-09	10	Sf3b4
0.896456682	8.16E-05	6.11E-09	10	Bcl2a1d
0.755828778	8.20E-05	6.14E-09	10	2310009A05Rik
0.755171383	8.21E-05	6.15E-09	10	Lactb
0.696921957	8.42E-05	6.31E-09	10	Sacm1l
0.977859689	8.64E-05	6.47E-09	10	Ndrg1
0.439058825	8.76E-05	6.56E-09	10	Vrk1
0.553954189	8.78E-05	6.57E-09	10	Lmnb1
1.143669277	8.78E-05	6.58E-09	10	Anp32e
0.976934058	9.01E-05	6.75E-09	10	Srsf11
0.910973726	9.15E-05	6.85E-09	10	Zmiz2
0.495306265	9.56E-05	7.16E-09	10	Tmc6
0.853083797	9.68E-05	7.25E-09	10	Rab32
0.989528562	9.72E-05	7.28E-09	10	Arpc5l
0.814120396	9.74E-05	7.30E-09	10	Rpl10
1.065915097	9.94E-05	7.44E-09	10	Trappc2l
0.684483682	0.00010022	7.51E-09	10	Hltf
0.851750309	0.000102033	7.64E-09	10	Sgsm2
1.034352203	0.000102798	7.70E-09	10	Slc38a1
0.795942174	0.000103334	7.74E-09	10	Filip1l
1.017323123	0.000104469	7.82E-09	10	Cib1

0.870298803	0.000104857	7.85E-09	10	Ccr2
0.328771197	0.000105325	7.89E-09	10	Hcfc2
0.750061189	0.000108525	8.13E-09	10	Gpaa1
1.015212911	0.000110226	8.26E-09	10	Tnfsf9
0.583907162	0.000110492	8.28E-09	10	Sart3
0.806529379	0.000110942	8.31E-09	10	Ino80c
0.365838296	0.000112593	8.43E-09	10	Orc3
0.329943301	0.000114128	8.55E-09	10	Fam173b
0.474136088	0.000114332	8.56E-09	10	Grasp
0.518503626	0.000116353	8.71E-09	10	9130023H24Rik
0.506552054	0.000117329	8.79E-09	10	Sec22b
0.8201482	0.000118492	8.87E-09	10	Rnasel
0.40056199	0.000122369	9.16E-09	10	Gm14005
0.988179896	0.000125368	9.39E-09	10	Ano6
0.529269064	0.000126801	9.50E-09	10	Nifk
0.839536151	0.000127288	9.53E-09	10	Nxf1
0.431684765	0.000135281	1.01E-08	10	Ccdc84
0.547535603	0.000136047	1.02E-08	10	Kdm3b
0.595508711	0.000136102	1.02E-08	10	Gpn3
0.971280474	0.000137499	1.03E-08	10	Mllt6
0.98419005	0.000139816	1.05E-08	10	Plekha1
0.777016645	0.000140231	1.05E-08	10	Knop1
0.425419904	0.000140501	1.05E-08	10	Abcd4
0.681398489	0.000141192	1.06E-08	10	Scamp1
0.897595707	0.000141999	1.06E-08	10	Pak1
0.604740469	0.000142223	1.07E-08	10	Cish
0.90477982	0.000144041	1.08E-08	10	Pcf11
0.458034727	0.000144475	1.08E-08	10	Nvl
0.458907836	0.000145652	1.09E-08	10	Zbtb1
1.02281622	0.00014723	1.10E-08	10	Yif1b
1.013847022	0.000152031	1.14E-08	10	Trex1
0.879291419	0.000153969	1.15E-08	10	Utp3
0.431050046	0.000155912	1.17E-08	10	Cep164
0.727614503	0.000156612	1.17E-08	10	Epb4.1
0.661670493	0.000156641	1.17E-08	10	Trim33
0.373761483	0.000158432	1.19E-08	10	Sik3
0.59119094	0.00016114	1.21E-08	10	Arhgef7
0.92948505	0.000161489	1.21E-08	10	Ufc1
0.641004888	0.000164627	1.23E-08	10	Eif2a
1.750579214	0.000166713	1.25E-08	10	Igfbp7
0.711894964	0.000169256	1.27E-08	10	Camk2d
0.487310911	0.000170548	1.28E-08	10	Wdr75
0.931565479	0.000173954	1.30E-08	10	Thoc2
0.579988012	0.000174929	1.31E-08	10	Smim20
0.895550327	0.00017666	1.32E-08	10	Il2rg
0.661443555	0.000179347	1.34E-08	10	Gemin2
0.919036073	0.000180569	1.35E-08	10	Zak
0.591327032	0.00018178	1.36E-08	10	Patl1
0.516181823	0.000182192	1.36E-08	10	Zdhhc18
0.556109457	0.000184711	1.38E-08	10	Sin3a
1.099937361	0.000193311	1.45E-08	10	Snx27
0.630162686	0.000194544	1.46E-08	10	Rnf146
0.406915052	0.00019634	1.47E-08	10	Drosha
0.839773617	0.000197223	1.48E-08	10	Crk
0.396210477	0.000200901	1.50E-08	10	Zfp945
0.756955123	0.000207434	1.55E-08	10	Ube4b
0.909966675	0.000207508	1.55E-08	10	Scd2
0.433004316	0.000209979	1.57E-08	10	9430008C03Rik
0.515967023	0.000212866	1.59E-08	10	Scyl2
0.662570497	0.000213696	1.60E-08	10	Stmn1
0.468023038	0.000222599	1.67E-08	10	Mrps30

0.588626178	0.00022385	1.68E-08	10	Klhl24
0.591245818	0.000225835	1.69E-08	10	Gimap5
0.856527096	0.000230499	1.73E-08	10	Tfg
0.98028867	0.000231975	1.74E-08	10	Aldh3b1
0.864169307	0.000237224	1.78E-08	10	Cd93
0.961483789	0.00024447	1.83E-08	10	Mrps15
0.78076683	0.000245881	1.84E-08	10	Vegfb
0.975315257	0.000246291	1.84E-08	10	Casp4
0.948859166	0.00024824	1.86E-08	10	Cnot1
0.963093306	0.000254038	1.90E-08	10	Ppp6r3
0.554228512	0.00026078	1.95E-08	10	Rnaseh2a
0.886078455	0.000265134	1.99E-08	10	Tcirg1
0.417517699	0.000267098	2.00E-08	10	Brd9
0.818473359	0.000273103	2.05E-08	10	Trim28
0.75934388	0.000276629	2.07E-08	10	Fam175b
0.623904134	0.000277261	2.08E-08	10	Ppa1
0.693808048	0.000284735	2.13E-08	10	Parp14
0.513232364	0.000287972	2.16E-08	10	2310009B15Rik
0.741566534	0.000288635	2.16E-08	10	Ahsa2
0.597549355	0.000289176	2.17E-08	10	Trim16
0.567215882	0.000293108	2.20E-08	10	Snhg12
1.082787811	0.000305924	2.29E-08	10	Pqlc1
0.783994577	0.000307005	2.30E-08	10	Tmem51
0.387186141	0.000308763	2.31E-08	10	Ino80e
0.393873893	0.000313553	2.35E-08	10	Bloc1s6
0.69932004	0.000316306	2.37E-08	10	Fam110a
0.802040439	0.000320124	2.40E-08	10	Brpf1
0.334710968	0.000322511	2.42E-08	10	Crls1
1.190869976	0.000324568	2.43E-08	10	Msra
0.340730138	0.000324877	2.43E-08	10	Lyrm2
0.980600696	0.000331304	2.48E-08	10	Dars
0.962156114	0.000349476	2.62E-08	10	Scarb2
0.973868057	0.000349604	2.62E-08	10	Il1rn
0.969665192	0.000349689	2.62E-08	10	Pnp
1.094142301	0.000352995	2.64E-08	10	Prkx
0.397341146	0.000355828	2.66E-08	10	Htt
0.315353912	0.000357187	2.68E-08	10	Zfp513
0.909403446	0.000365971	2.74E-08	10	Got1
0.722764749	0.000372886	2.79E-08	10	Snx8
0.969384752	0.000379776	2.84E-08	10	Entpd1
0.947051957	0.000381902	2.86E-08	10	Kdm1a
0.892327492	0.000382443	2.86E-08	10	Cebpz
0.726009069	0.00038757	2.90E-08	10	Fech
0.717165125	0.000390919	2.93E-08	10	Nkiras2
0.955632848	0.000403084	3.02E-08	10	Copa
0.639920802	0.000404812	3.03E-08	10	AW112010
0.960843431	0.000408208	3.06E-08	10	H2afv
0.293352293	0.000410264	3.07E-08	10	Zbtb16
1.010024692	0.000414031	3.10E-08	10	Rbbp6
0.508957711	0.000414381	3.10E-08	10	Terf2
0.632560493	0.00041538	3.11E-08	10	Faf1
0.572877167	0.000418767	3.14E-08	10	Rpl31
0.975456047	0.000432611	3.24E-08	10	Cog7
0.520837787	0.000441478	3.31E-08	10	Dhx36
-1.773393843	0.000445121	3.33E-08	10	Rpl37a
0.628864305	0.00044589	3.34E-08	10	Mak16
0.762907176	0.000447823	3.35E-08	10	Cdk4
0.94775498	0.000456791	3.42E-08	10	Mknk1
0.720577213	0.000457262	3.42E-08	10	Dpf2
0.452970041	0.000458092	3.43E-08	10	Dtx3l
0.90501131	0.000459359	3.44E-08	10	Eif1a

0.342066559	0.000463139	3.47E-08	10	Nedd9
0.414961274	0.00046814	3.51E-08	10	Gpr89
0.543913976	0.000470382	3.52E-08	10	Mogs
0.841384993	0.000471344	3.53E-08	10	Chsy1
0.815776341	0.000477176	3.57E-08	10	Ict1
0.709831548	0.000487748	3.65E-08	10	2310045N01Rik
0.535773678	0.00048793	3.65E-08	10	Gtf2f2
1.028709575	0.000492825	3.69E-08	10	Agap1
0.611449738	0.000495526	3.71E-08	10	Ppp3cb
0.801497638	0.000495871	3.71E-08	10	Ap2a2
0.717266283	0.000500383	3.75E-08	10	Stat1
0.496353517	0.000525171	3.93E-08	10	Xpo6
0.442358024	0.000532486	3.99E-08	10	Arhgap35
0.775899183	0.000536841	4.02E-08	10	Ranbp2
0.35483473	0.000538213	4.03E-08	10	E230008N13Rik
0.956236007	0.000539258	4.04E-08	10	Riok1
0.853782516	0.00053986	4.04E-08	10	Xbp1
0.455843071	0.000546596	4.09E-08	10	Map2k7
0.389476395	0.000563971	4.22E-08	10	Map3k3
0.659282667	0.00056654	4.24E-08	10	Ppfibp1
0.685884916	0.000566755	4.24E-08	10	Pigf
0.793259342	0.000574604	4.30E-08	10	Rbm45
0.325151938	0.000575575	4.31E-08	10	Cstf3
0.586718363	0.000575695	4.31E-08	10	Timm22
0.511364034	0.000579281	4.34E-08	10	Ms4a4c
0.496201363	0.000592747	4.44E-08	10	Tmem222
0.670332503	0.000594595	4.45E-08	10	Heatr3
1.041685004	0.000599789	4.49E-08	10	Acp5
0.871568071	0.000600362	4.50E-08	10	Edem1
0.71775996	0.000603248	4.52E-08	10	Odf2
0.942266218	0.0006054	4.53E-08	10	Dnajc15
0.585560638	0.000608124	4.55E-08	10	Sharpin
0.6138417	0.000608666	4.56E-08	10	Ckap4
0.914948794	0.000616542	4.62E-08	10	Rc3h1
0.855959927	0.000617831	4.63E-08	10	Phf23
0.582949171	0.00062089	4.65E-08	10	Tet2
0.47071278	0.00062395	4.67E-08	10	9130401M01Rik
0.929450694	0.000639388	4.79E-08	10	Uqcrfs1
0.688414362	0.000645187	4.83E-08	10	Srrt
0.417657345	0.000652688	4.89E-08	10	Pno1
0.832375883	0.000656974	4.92E-08	10	Azi2
0.603077251	0.0006574	4.92E-08	10	B230219D22Rik
0.453516909	0.000660107	4.94E-08	10	Wdr7
0.604494208	0.00068584	5.14E-08	10	Mrpl46
0.639777318	0.000689605	5.16E-08	10	Zdhhc5
1.087553048	0.000691641	5.18E-08	10	Atp13a2
0.544861027	0.000710136	5.32E-08	10	Gtf3c2
0.746063664	0.000716155	5.36E-08	10	Ppfia1
0.431385286	0.000726782	5.44E-08	10	Acbd6
0.777613936	0.000739201	5.54E-08	10	2210016F16Rik
0.296497939	0.000740483	5.55E-08	10	Hirip3
0.309455872	0.00077901	5.83E-08	10	Usp33
0.38759333	0.000792213	5.93E-08	10	Cxxc1
0.716833622	0.000816388	6.11E-08	10	Atf1
0.327819873	0.000826315	6.19E-08	10	Ppp1r8
0.971959024	0.00083272	6.24E-08	10	Cnpy3
0.562971512	0.000836372	6.26E-08	10	Agps
0.491961224	0.000855198	6.41E-08	10	Ddx18
0.765328069	0.000865984	6.49E-08	10	BC003331
1.155515696	0.000871116	6.52E-08	10	Rnf8
0.379952007	0.000896476	6.71E-08	10	Rpl36

0.396133781	0.000921484	6.90E-08	10	Rnf41
1.378554124	0.000939828	7.04E-08	10	Chd3
0.688230606	0.000951757	7.13E-08	10	Mvb12a
0.686854802	0.000957835	7.17E-08	10	Tmem33
0.735836457	0.000969678	7.26E-08	10	Ddx39
0.699513557	0.000996342	7.46E-08	10	Rtp4
0.689546148	0.001001691	7.50E-08	10	Gpbp1l1
-0.443100923	0.001012132	7.58E-08	10	Actb
0.527335184	0.001057003	7.92E-08	10	Eif4e3
0.781634258	0.001061362	7.95E-08	10	Pdia6
0.840329526	0.001066647	7.99E-08	10	Mdfic
0.49491718	0.001092726	8.18E-08	10	Ccnk
0.663438217	0.001134878	8.50E-08	10	Atp5a1
0.520251913	0.001138473	8.53E-08	10	Cyld
0.914523997	0.001144437	8.57E-08	10	Hilpda
0.666306446	0.001147694	8.60E-08	10	Nr4a2
0.483604387	0.001167191	8.74E-08	10	Atf7ip
0.566681522	0.001187654	8.89E-08	10	Ammecr1l
0.858656125	0.001191616	8.92E-08	10	Zfand2a
0.790513883	0.001236474	9.26E-08	10	Slc29a3
0.905241886	0.001237072	9.27E-08	10	Nhp2
0.528314496	0.001244897	9.32E-08	10	Mefv
0.743200549	0.001253227	9.39E-08	10	Rab11fip2
0.355217289	0.00125959	9.43E-08	10	Gid8
0.78873062	0.001263818	9.47E-08	10	Nlrp3
0.613593896	0.00127106	9.52E-08	10	Sf3b3
0.827243562	0.001273877	9.54E-08	10	Smim3
0.453358739	0.001274973	9.55E-08	10	Polr3k
1.013266012	0.001286198	9.63E-08	10	Clec4n
0.686543979	0.001289032	9.65E-08	10	Agpat6
0.574492381	0.001301776	9.75E-08	10	Tnrc6c
0.970035556	0.001304563	9.77E-08	10	Lrrc59
0.812387321	0.001314035	9.84E-08	10	Krcc1
0.825848514	0.001342392	1.01E-07	10	Bhlhe40
0.409722334	0.001356772	1.02E-07	10	Sfmbt1
0.689170345	0.001385806	1.04E-07	10	Anapc7
0.797003774	0.00139065	1.04E-07	10	Rbm5
0.843628131	0.001400564	1.05E-07	10	Mkl1
0.390538603	0.001408762	1.06E-07	10	Arfgap1
0.378612833	0.00142297	1.07E-07	10	Lin37
0.67576692	0.001429122	1.07E-07	10	Cyp4f18
0.385382333	0.001454448	1.09E-07	10	Ctnnbl1
1.097864705	0.00147903	1.11E-07	10	Mrvi1
0.271077111	0.001518826	1.14E-07	10	Zfp518a
0.32659807	0.001556052	1.17E-07	10	Tagap1
0.431548359	0.001567548	1.17E-07	10	Hgf
0.265202796	0.00158897	1.19E-07	10	Zfp637
0.460870975	0.001644576	1.23E-07	10	Fas
0.424933558	0.001677356	1.26E-07	10	Ak6
0.682648822	0.001684538	1.26E-07	10	Smchd1
0.855457699	0.001708557	1.28E-07	10	Vimp
0.779853732	0.001712394	1.28E-07	10	Ppp2ca
0.52918824	0.001764938	1.32E-07	10	Adipor2
0.863674045	0.001787562	1.34E-07	10	Maea
0.760419859	0.001796771	1.35E-07	10	Rwdd1
0.487701779	0.001902005	1.42E-07	10	Kdsr
0.939660147	0.001905062	1.43E-07	10	Ech1
0.444391747	0.001952332	1.46E-07	10	Naa40
0.792574514	0.001969399	1.47E-07	10	Exoc3
0.525199368	0.002015485	1.51E-07	10	Trmt1
0.887935874	0.00202385	1.52E-07	10	Otulin

0.367521549	0.002044362	1.53E-07	10	Hspa4l
0.593176321	0.002094781	1.57E-07	10	Gorasp2
0.805614698	0.002102739	1.57E-07	10	Wbp5
0.587028117	0.002119466	1.59E-07	10	Rpl15
0.806783606	0.002159429	1.62E-07	10	Fam46c
0.76582127	0.002179873	1.63E-07	10	Lat
0.821633366	0.002194064	1.64E-07	10	Syt11
0.886230355	0.002229511	1.67E-07	10	Mar.05
0.450943062	0.002233619	1.67E-07	10	Nqo2
0.714996576	0.002271922	1.70E-07	10	Abcd1
0.783211289	0.00228962	1.71E-07	10	Tnfaip2
0.593269155	0.00228968	1.71E-07	10	Mrps7
0.764556316	0.002299769	1.72E-07	10	Arl8b
0.823592314	0.002301558	1.72E-07	10	Arcn1
0.770209746	0.002311261	1.73E-07	10	Nucb2
0.733321837	0.002317921	1.74E-07	10	Clip1
0.778121008	0.002328899	1.74E-07	10	Mrpl12
1.289502302	0.002335687	1.75E-07	10	Ubqln1
0.728679054	0.002355705	1.76E-07	10	Tuba1b
0.437839448	0.00240697	1.80E-07	10	Clp1
0.871538011	0.00242418	1.82E-07	10	Hspa4
0.644090798	0.002460361	1.84E-07	10	Rps19bp1
0.599577516	0.002485072	1.86E-07	10	Max
0.479806256	0.002485913	1.86E-07	10	Tiprl
0.427954033	0.002500831	1.87E-07	10	Setd5
0.607164645	0.002595765	1.94E-07	10	Slc4a2
0.91149456	0.002595825	1.94E-07	10	Cadm1
0.858666102	0.002607641	1.95E-07	10	Map4
0.780598399	0.002667949	2.00E-07	10	Dyrk1a
0.814237999	0.002673506	2.00E-07	10	Zranb2
0.574723325	0.002725862	2.04E-07	10	Abce1
0.328123388	0.002775108	2.08E-07	10	Hspbap1
0.773306066	0.002775504	2.08E-07	10	Commd7
0.816916582	0.002832955	2.12E-07	10	Clec7a
0.499498816	0.002867894	2.15E-07	10	Tmem184c
0.367615548	0.002890469	2.16E-07	10	Nudt14
0.890160008	0.002935128	2.20E-07	10	Usp14
0.716639956	0.00300306	2.25E-07	10	Pkib
0.831803675	0.003007562	2.25E-07	10	Ssr3
0.513741572	0.003042307	2.28E-07	10	1810011H11Rik
0.753748115	0.00312996	2.34E-07	10	Hdac2
0.881372294	0.003164985	2.37E-07	10	Tcf25
0.536031702	0.003208702	2.40E-07	10	Mdp1
0.672770537	0.003217654	2.41E-07	10	Ncs1
0.430012094	0.003228824	2.42E-07	10	Zbtb2
0.563281687	0.00331961	2.49E-07	10	Stxbp3a
0.438626546	0.003359207	2.52E-07	10	Zyg11b
0.774156947	0.00340455	2.55E-07	10	Pja2
0.409801419	0.003429223	2.57E-07	10	Psat1
0.53290247	0.003433164	2.57E-07	10	Mctp1
0.712866971	0.003442341	2.58E-07	10	Smu1
0.308918805	0.003442574	2.58E-07	10	Stk3
0.557322746	0.003524732	2.64E-07	10	Vps72
0.666391104	0.00352573	2.64E-07	10	Cct5
0.667400725	0.003615786	2.71E-07	10	Top1
0.800259723	0.003653112	2.74E-07	10	Dctn5
0.823253926	0.003654945	2.74E-07	10	Ctbp2
0.360875683	0.003672335	2.75E-07	10	Dennd2c
0.821922362	0.003674279	2.75E-07	10	Thbs1
0.750211182	0.003718417	2.78E-07	10	Cdt1
0.514530013	0.003749358	2.81E-07	10	Cep83

0.516392239	0.00384087	2.88E-07	10	Nop9
0.866038853	0.003869951	2.90E-07	10	Eif2s1
0.638369036	0.003913437	2.93E-07	10	Tmem251
0.621778299	0.003918479	2.93E-07	10	Hnrnpll
0.859522175	0.003933292	2.95E-07	10	Aurkaip1
0.735299152	0.00398588	2.99E-07	10	Ube2f
0.475789566	0.004016087	3.01E-07	10	Pdgfc
0.732247211	0.004052664	3.04E-07	10	Rabgef1
0.267450092	0.00408895	3.06E-07	10	Taf1
0.421062647	0.004091883	3.06E-07	10	Ldlrad4
0.673334006	0.004106917	3.08E-07	10	Snap29
0.326405132	0.004166457	3.12E-07	10	Lancl1
0.450294544	0.00417583	3.13E-07	10	Egr2
0.577830276	0.004224198	3.16E-07	10	Khdrbs1
0.426622674	0.004237683	3.17E-07	10	Uhrf1
0.735383188	0.004379404	3.28E-07	10	Acbd3
-0.686681227	0.004459147	3.34E-07	10	Cst3
0.402593864	0.00457628	3.43E-07	10	Galnt2
0.901639197	0.004577673	3.43E-07	10	Asap1
0.849848344	0.004601163	3.45E-07	10	Pabpn1
0.818536347	0.004632894	3.47E-07	10	Etfb
0.535517688	0.004690953	3.51E-07	10	Mdn1
0.412730849	0.004788138	3.59E-07	10	Mob4
0.415136728	0.004832517	3.62E-07	10	Cry1
0.702886148	0.00487298	3.65E-07	10	Fhl3
0.639367027	0.004877883	3.65E-07	10	Dot1l
0.655055701	0.004884057	3.66E-07	10	Pptc7
0.553542023	0.004897074	3.67E-07	10	Nol8
0.73316672	0.0049147	3.68E-07	10	Polr2a
0.324385631	0.004934981	3.70E-07	10	Crebrf
0.739776485	0.004972364	3.72E-07	10	Ddx46
1.058098034	0.004975771	3.73E-07	10	Snx17
0.447858819	0.005223598	3.91E-07	10	Ccdc115
0.791457292	0.005287991	3.96E-07	10	Psmd1
0.565654403	0.005353553	4.01E-07	10	Snx12
1.01542164	0.005368221	4.02E-07	10	Camta2
0.820313614	0.005394584	4.04E-07	10	Vps4b
0.251353676	0.005423038	4.06E-07	10	Parp16
0.680000564	0.005440686	4.07E-07	10	Tapbp
0.695304639	0.005462087	4.09E-07	10	Tax1bp1
0.872202492	0.005571066	4.17E-07	10	Map2k4
0.727622544	0.005607699	4.20E-07	10	Gnaq
0.450947811	0.005620598	4.21E-07	10	Rnpc3
-2.584422509	0.005728384	4.29E-07	10	Syngr1
0.8399782	0.005776813	4.33E-07	10	Jak2
0.261928966	0.005908489	4.43E-07	10	Lsm2
0.712024507	0.00594304	4.45E-07	10	Il1b
0.525702217	0.005994224	4.49E-07	10	Nme3
0.254445699	0.006332944	4.74E-07	10	Mettl16
0.585238076	0.006395984	4.79E-07	10	Gpr137
0.508222435	0.006411166	4.80E-07	10	Mrps35
0.271152771	0.006510656	4.88E-07	10	Mbip
0.595084724	0.006572668	4.92E-07	10	Gon4l
0.656420257	0.006582903	4.93E-07	10	Nop14
0.485316737	0.00661694	4.96E-07	10	Slc1a5
0.725735392	0.006799798	5.09E-07	10	Ogdh
0.460887796	0.006854171	5.13E-07	10	Taf6
0.490866159	0.006895755	5.16E-07	10	Cspp1
0.765825646	0.006997842	5.24E-07	10	Clec4e
0.307427009	0.007075652	5.30E-07	10	Clcn3
0.413784878	0.007095732	5.31E-07	10	Mtmr4

0.775643622	0.007144486	5.35E-07	10	Uba1
0.568549808	0.007282573	5.45E-07	10	Naip5
0.270485387	0.00733563	5.49E-07	10	Clec2d
0.531449295	0.007350154	5.50E-07	10	Ado
0.408562415	0.007352331	5.51E-07	10	Flot1
0.783060768	0.007352796	5.51E-07	10	Sdf2
0.271926886	0.00748104	5.60E-07	10	Zfp524
0.409628334	0.007817792	5.86E-07	10	Cept1
0.703243741	0.008185561	6.13E-07	10	Usp34
0.716257335	0.00821395	6.15E-07	10	Nceh1
0.680845002	0.008266787	6.19E-07	10	Ubxn4
0.62648159	0.008359844	6.26E-07	10	Smarcc2
0.742400352	0.008434549	6.32E-07	10	Ube2b
0.535638223	0.008561108	6.41E-07	10	R3hdm1
0.90715356	0.008718626	6.53E-07	10	Dpp7
0.785949519	0.008728558	6.54E-07	10	Pa2g4
0.577758689	0.008738606	6.54E-07	10	Arl6ip1
0.730320364	0.008748451	6.55E-07	10	Itsn2
0.461577689	0.008951523	6.70E-07	10	Myo10
0.790701296	0.008984167	6.73E-07	10	Rbm42
0.565707552	0.009091503	6.81E-07	10	Gnptg
0.751034024	0.009144086	6.85E-07	10	Gla
0.578655158	0.009164912	6.86E-07	10	Papd5
0.735789719	0.009178966	6.87E-07	10	Fam134a
0.27838942	0.009315976	6.98E-07	10	Tfpt
0.278817054	0.009329252	6.99E-07	10	Polr1a
0.76540979	0.009391891	7.03E-07	10	Psmd7
0.573124097	0.009402624	7.04E-07	10	Crebbp
0.769655678	0.009416426	7.05E-07	10	Ebp
0.482661595	0.009429164	7.06E-07	10	Asnsd1
0.274291169	0.009523473	7.13E-07	10	Glb1
0.46350633	0.009571703	7.17E-07	10	Hspa14
0.627675458	0.009728911	7.29E-07	10	Cyp4v3
0.670038601	0.009748346	7.30E-07	10	Prdx4
0.543199085	0.009847132	7.38E-07	10	Zc3h18
0.458183919	0.010366813	7.76E-07	10	Nsmaf
0.476176488	0.010386325	7.78E-07	10	Ralgapb
0.319927172	0.01045105	7.83E-07	10	Cdc42ep2
0.589419094	0.010529983	7.89E-07	10	Fnbp1l
0.301235039	0.010637699	7.97E-07	10	Lrrc75a
0.626930669	0.010662757	7.99E-07	10	Ehmt2
0.588231475	0.010879534	8.15E-07	10	Cped1
0.411076442	0.010918813	8.18E-07	10	Aqr
0.263245334	0.010937804	8.19E-07	10	Zfp276
0.416768355	0.011011576	8.25E-07	10	Tctex1d4
0.311711402	0.011021717	8.25E-07	10	Trnt1
0.743597376	0.011137887	8.34E-07	10	Dnajc3
0.451510946	0.011289731	8.46E-07	10	N4bp2l1
0.510899026	0.011321396	8.48E-07	10	Kidins220
0.600105825	0.011386984	8.53E-07	10	Atg13
0.728715232	0.011408095	8.54E-07	10	Hnrnpa0
0.659354026	0.011496002	8.61E-07	10	Pak1ip1
1.248552484	0.011500875	8.61E-07	10	Lfng
0.483432457	0.011636537	8.72E-07	10	AI504432
0.347990238	0.011745753	8.80E-07	10	Rab11fip1
0.729037124	0.011877888	8.90E-07	10	Ndufs7
0.784440713	0.011883486	8.90E-07	10	Uggt1
0.406239981	0.01238068	9.27E-07	10	Gna15
0.373080979	0.012675166	9.49E-07	10	Pde6d
0.643144446	0.012681357	9.50E-07	10	Ghitm
0.536180442	0.012790355	9.58E-07	10	Acat1

0.429718947	0.013377588	1.00E-06	10	Mettl9
0.671556343	0.013397389	1.00E-06	10	Trps1
0.662091294	0.01346421	1.01E-06	10	Ngdn
0.786337372	0.014531341	1.09E-06	10	Fam195b
0.57656878	0.014553983	1.09E-06	10	Slc3a2
0.645488409	0.014634575	1.10E-06	10	Ep400
0.311706339	0.014638017	1.10E-06	10	Kctd5
0.65348113	0.014782549	1.11E-06	10	Mcts1
0.458774126	0.01497427	1.12E-06	10	Fam49a
0.596357161	0.01506704	1.13E-06	10	Emb
0.717396515	0.0153529	1.15E-06	10	Smc6
0.261013825	0.015396375	1.15E-06	10	Hras
0.47114714	0.015783776	1.18E-06	10	Plxnd1
0.712912089	0.015810396	1.18E-06	10	Ndufs4
0.726610307	0.015946436	1.19E-06	10	Psmc4
0.638549596	0.016004942	1.20E-06	10	Ripk1
0.426764047	0.016067666	1.20E-06	10	Phf11b
0.474925706	0.016313685	1.22E-06	10	Ring1
-1.266557495	0.016401199	1.23E-06	10	Rps19
0.376500549	0.016507429	1.24E-06	10	Kremen1
0.616148537	0.01655955	1.24E-06	10	Mesdc2
0.360986527	0.016955904	1.27E-06	10	Slc15a2
0.489232693	0.016982948	1.27E-06	10	P2rx7
0.699033602	0.017269183	1.29E-06	10	Plekho2
0.385844301	0.017371319	1.30E-06	10	Manbal
0.603385748	0.017380904	1.30E-06	10	Fam117b
0.752099754	0.017590923	1.32E-06	10	Bmpr2
0.63832475	0.017617885	1.32E-06	10	Cyth2
0.64195435	0.018013083	1.35E-06	10	Vps11
0.89116901	0.018252577	1.37E-06	10	Rreb1
0.810646235	0.018319662	1.37E-06	10	Lpp
0.608372854	0.018847699	1.41E-06	10	Anapc2
0.281443939	0.019621901	1.47E-06	10	Skap1
0.841118865	0.019790085	1.48E-06	10	Slc25a11
0.573549038	0.01982935	1.49E-06	10	Spast
0.448253348	0.019982889	1.50E-06	10	Atg101
0.781871745	0.0207525	1.55E-06	10	Rnpep
0.442007593	0.020830121	1.56E-06	10	Timp3
0.279058971	0.020897937	1.57E-06	10	Fyttd1
0.463037756	0.02124332	1.59E-06	10	Ulk2
0.475853334	0.021288431	1.59E-06	10	Wdr11
0.494580095	0.021647884	1.62E-06	10	Trappc10
0.493482235	0.022483087	1.68E-06	10	Ifrd1
0.682529319	0.02289234	1.71E-06	10	Atp6v0d1
0.466959833	0.023002923	1.72E-06	10	Cd3e
0.792449694	0.023113164	1.73E-06	10	Itgb3
0.572267816	0.023444455	1.76E-06	10	Macf1
0.3428886	0.023914415	1.79E-06	10	Htra2
0.592441168	0.023952867	1.79E-06	10	Riok3
0.762687463	0.023993439	1.80E-06	10	Npepl1
0.472357868	0.024532113	1.84E-06	10	2310022A10Rik
0.34100171	0.024736854	1.85E-06	10	Srp54a
1.050341348	0.024894198	1.86E-06	10	Slc15a3
0.668936206	0.024925354	1.87E-06	10	Slamf7
0.842717136	0.024970235	1.87E-06	10	Slc20a1
0.388620597	0.025063157	1.88E-06	10	Rbm14
0.304686293	0.025478601	1.91E-06	10	Clock
0.583019937	0.025822829	1.93E-06	10	Psmd6
0.679576062	0.026011862	1.95E-06	10	Ten1
0.501928984	0.026253131	1.97E-06	10	Cbx5
0.546882622	0.026369968	1.97E-06	10	Mrps23

0.69380714	0.02637473	1.98E-06	10	Igsf8
0.589153175	0.026445076	1.98E-06	10	Rnf149
0.314009734	0.026452004	1.98E-06	10	Tango2
0.667613589	0.026793464	2.01E-06	10	Rnf111
-2.24478026	0.027096943	2.03E-06	10	Eps15
0.514755696	0.027824416	2.08E-06	10	Cops8
0.643534824	0.028404692	2.13E-06	10	Scand1
0.817939513	0.02902519	2.17E-06	10	Eif3d
0.5453118	0.029580456	2.22E-06	10	Twistnb
0.448524957	0.029750982	2.23E-06	10	Irs2
0.547986313	0.02976021	2.23E-06	10	Mrpl3
0.622038493	0.030051117	2.25E-06	10	Arl5a
0.71834863	0.030101238	2.25E-06	10	Rapgef6
0.73808928	0.03041735	2.28E-06	10	Gde1
0.44613131	0.030948659	2.32E-06	10	Camkk2
0.729079479	0.031393768	2.35E-06	10	Pdgfa
0.872057272	0.031776228	2.38E-06	10	Atp2a2
0.554819616	0.031786058	2.38E-06	10	Ptpn2
0.640585174	0.031832534	2.38E-06	10	Vav1
0.317524085	0.032019679	2.40E-06	10	Serpinb8
0.706901455	0.032230827	2.41E-06	10	Ptpro
0.668565197	0.032666022	2.45E-06	10	Psmc5
0.633984789	0.033358495	2.50E-06	10	Bag1
0.533667948	0.033475706	2.51E-06	10	Cacybp
0.593449033	0.033574274	2.51E-06	10	Lbr
0.57205609	0.033884279	2.54E-06	10	Dhx15
0.377434465	0.034061579	2.55E-06	10	Hps1
0.307722823	0.034224397	2.56E-06	10	AB041803
0.53680111	0.034985919	2.62E-06	10	Gpr183
0.475702218	0.035112565	2.63E-06	10	Lhpp
0.500909282	0.035324106	2.65E-06	10	Rps6kb1
0.332080186	0.035329561	2.65E-06	10	Tollip
0.492676305	0.035790291	2.68E-06	10	Snx13
0.309923044	0.036028633	2.70E-06	10	Rbm27
0.802558625	0.036545223	2.74E-06	10	Plod1
0.443001925	0.037101882	2.78E-06	10	Braf
0.665761812	0.03710385	2.78E-06	10	Ppp2r5c
-2.349096367	0.038852738	2.91E-06	10	Rin2
0.751471695	0.039963087	2.99E-06	10	Slc11a2
0.666195663	0.040184527	3.01E-06	10	Emc6
-2.237472981	0.040214749	3.01E-06	10	Dpysl2
0.275427155	0.04022515	3.01E-06	10	Ccne1
0.324330754	0.040420112	3.03E-06	10	Mtm1
0.442188941	0.040572789	3.04E-06	10	Zfp597
1.166348054	0.041617924	3.12E-06	10	Polr2i
0.295578727	0.041722694	3.12E-06	10	Nup35
-0.566621828	0.041823141	3.13E-06	10	B2m
0.30881854	0.042132026	3.16E-06	10	Ciao1
0.56664442	0.042879095	3.21E-06	10	Atpif1
0.439707847	0.043455114	3.25E-06	10	Wdr74
0.534057624	0.04373448	3.28E-06	10	Fmnl3
0.297292205	0.044149271	3.31E-06	10	Nup62
-2.313892011	0.044412442	3.33E-06	10	Cdk2ap2
0.268676159	0.044490558	3.33E-06	10	Ap1m1
0.363755396	0.044775788	3.35E-06	10	Col27a1
0.346809766	0.045301293	3.39E-06	10	Tmcc1
0.273646019	0.045425675	3.40E-06	10	Nr1d2
0.600540864	0.045615808	3.42E-06	10	Taf1d
0.422253276	0.045783137	3.43E-06	10	Uaca
0.659719657	0.045798675	3.43E-06	10	Cops2
0.315833838	0.046257224	3.46E-06	10	Cbr1

0.40565562	0.046269629	3.47E-06	10	Polr2m
0.30765372	0.046403223	3.48E-06	10	Mrps28
0.612930713	0.046575157	3.49E-06	10	Rab3gap2
0.331537984	0.046763615	3.50E-06	10	Spryd7
0.561878793	0.047016304	3.52E-06	10	Plcg2
0.768867516	0.048113433	3.60E-06	10	Flna
0.560927977	0.048163834	3.61E-06	10	Mrpl48
0.568012319	0.049023985	3.67E-06	10	Rbp1
0.790388528	0.049123665	3.68E-06	10	Mmp14
0.411389588	0.049167061	3.68E-06	10	Nfil3
0.620670575	0.049207478	3.69E-06	10	Ccdc86
0.74009579	0.04955136	3.71E-06	10	Hdgfrp3
0.657303905	0.049799303	3.73E-06	10	Cds1
0.555915863	0.050447148	3.78E-06	10	Cntrl
0.2920968	0.051102302	3.83E-06	10	Flot2
0.63155341	0.052033715	3.90E-06	10	Ifi204
0.657874354	0.052424438	3.93E-06	10	Ndufb8
0.626742067	0.053005287	3.97E-06	10	Plaur
0.266569523	0.053654602	4.02E-06	10	Mtr
0.463876996	0.054139947	4.05E-06	10	Gas2l3
0.262225385	0.054383424	4.07E-06	10	Cul2
0.737122899	0.054406611	4.07E-06	10	Tmem126a
0.726235732	0.054838432	4.11E-06	10	Itgax
0.618067919	0.054925892	4.11E-06	10	Ormdl2
0.711918946	0.055764524	4.18E-06	10	Ap2m1
0.591726328	0.056871382	4.26E-06	10	Tnfaip3
0.869622611	0.057882041	4.34E-06	10	Zmat5
0.490644533	0.059387646	4.45E-06	10	Gnai3
0.596723768	0.059416915	4.45E-06	10	Mfsd1
0.592867947	0.059586145	4.46E-06	10	BC005537
0.340934615	0.059993148	4.49E-06	10	Stim2
0.829645536	0.06108175	4.57E-06	10	Tgm2
0.436289661	0.062088723	4.65E-06	10	Cisd2
0.331264485	0.062589561	4.69E-06	10	Irf2bp1
0.319694262	0.062598667	4.69E-06	10	Ccdc130
0.633504066	0.063954379	4.79E-06	10	Uvrag
0.531606293	0.064084011	4.80E-06	10	Rsrp1
0.670555278	0.064197041	4.81E-06	10	Elovl1
0.478705848	0.065204675	4.88E-06	10	Stau1
0.628671921	0.065259409	4.89E-06	10	Tra2b
0.356217173	0.066773749	5.00E-06	10	Ubxn6
0.467238226	0.067190407	5.03E-06	10	Slc25a44
0.262828658	0.067534189	5.06E-06	10	Hnrnpr
0.476176488	0.067939032	5.09E-06	10	1300002K09Rik
0.344429218	0.068904326	5.16E-06	10	Ncln
0.575364145	0.070099976	5.25E-06	10	Birc6
0.642702357	0.07022365	5.26E-06	10	Fam13b
0.587212252	0.071362464	5.34E-06	10	Nfkbib
0.642506989	0.072907655	5.46E-06	10	Rbm33
0.554716315	0.073491903	5.50E-06	10	Tpm2
0.574589516	0.073815443	5.53E-06	10	Pin1
0.272527919	0.0739777	5.54E-06	10	Ugdh
0.511200762	0.074179688	5.56E-06	10	Tmem65
0.510061207	0.074536267	5.58E-06	10	Mia3
0.514750696	0.074981128	5.62E-06	10	Hk2
0.525028574	0.075469111	5.65E-06	10	Smc4
0.476698727	0.076043745	5.70E-06	10	Smek1
0.57001716	0.076617972	5.74E-06	10	Lsm14a
0.353116396	0.078443055	5.88E-06	10	Il18
0.567829583	0.079797244	5.98E-06	10	Atp5d
0.621935224	0.07997555	5.99E-06	10	Wapal

0.390383334	0.080018967	5.99E-06	10	Cpsf2
0.688474702	0.080155467	6.00E-06	10	Smad1
0.440617186	0.080279105	6.01E-06	10	Tars2
0.407645084	0.080392588	6.02E-06	10	Rpain
0.421765329	0.080424127	6.02E-06	10	Mrpl28
0.282964612	0.080429877	6.02E-06	10	Cpsf3l
0.749829045	0.082077549	6.15E-06	10	Esyt1
0.255579898	0.082320596	6.17E-06	10	Ddhd2
0.65734153	0.082500924	6.18E-06	10	Utp11l
0.325316166	0.083020603	6.22E-06	10	Gga3
0.595613759	0.083609957	6.26E-06	10	Sft2d2
0.65123264	0.083943358	6.29E-06	10	Nipbl
0.717287617	0.084507982	6.33E-06	10	Pde4b
0.584810329	0.084997044	6.37E-06	10	NP-904339.1
0.622048074	0.08683345	6.50E-06	10	Rel
0.572415378	0.087066568	6.52E-06	10	Eif4g1
-2.094573723	0.089082998	6.67E-06	10	Trim25
0.330618772	0.089605497	6.71E-06	10	Mar.06
-1.853107811	0.091131149	6.83E-06	10	Ski
0.544990234	0.091578598	6.86E-06	10	Csnk1a1
0.263439843	0.091891168	6.88E-06	10	Nmt2
0.447529145	0.091939912	6.89E-06	10	Cerk
0.324196773	0.092751365	6.95E-06	10	Il2rb
0.5168591	0.093337618	6.99E-06	10	Safb2
0.456728261	0.095022681	7.12E-06	10	Dpm3
0.680820699	0.097389103	7.29E-06	10	Zfpl1
0.298112348	0.097883945	7.33E-06	10	Qtrt1
0.419466204	0.098389845	7.37E-06	10	Anks3
0.369031851	0.099230218	7.43E-06	10	Pign
0.643814825	0.10110937	7.57E-06	10	Gsdmd
0.279125575	0.101406509	7.59E-06	10	Prkrip1
-0.476266528	0.101470195	7.60E-06	10	Gpx1
1.028936257	0.10257407	7.68E-06	10	Lyst
0.253741906	0.103240896	7.73E-06	10	Mrps12
0.757919419	0.10338855	7.74E-06	10	Kdm5c
0.364546077	0.10509272	7.87E-06	10	Mrpl41
0.331953688	0.105767205	7.92E-06	10	Bbip1
0.340733145	0.107160024	8.03E-06	10	Vbp1
0.630913109	0.107743284	8.07E-06	10	Polr2e
0.652378699	0.107883089	8.08E-06	10	Bnip3l
0.421157113	0.107903015	8.08E-06	10	Fnbp4
0.727346042	0.108039881	8.09E-06	10	Dtnbp1
0.371671954	0.108512462	8.13E-06	10	Ilf3
0.457937758	0.109724682	8.22E-06	10	Klhdc3
-2.039982705	0.110710846	8.29E-06	10	Fndc3b
0.643543347	0.111634846	8.36E-06	10	Psmd12
0.386484301	0.111966625	8.39E-06	10	Dcun1d2
-0.959729516	0.112828844	8.45E-06	10	Rps26
0.497904044	0.113382186	8.49E-06	10	Anp32a
0.669218884	0.113823897	8.52E-06	10	Fam129b
0.372702217	0.114469275	8.57E-06	10	Rab35
0.307091326	0.115158783	8.62E-06	10	Mxd1
0.365697679	0.115210444	8.63E-06	10	Dync1li2
0.326484572	0.122526465	9.18E-06	10	Mgat2
0.614456837	0.122549442	9.18E-06	10	Ndufa5
0.857304774	0.124801553	9.35E-06	10	Gdf15
0.625945442	0.126218843	9.45E-06	10	Cops5
0.342645096	0.126436729	9.47E-06	10	Cenpw
0.634922931	0.126955318	9.51E-06	10	Usp7
0.437710208	0.131481257	9.85E-06	10	Smarca5
0.30524708	0.131923549	9.88E-06	10	Rnd3

0.42038834	0.132707571	9.94E-06	10	Cmas
0.409186835	0.133166212	9.97E-06	10	Cox16
-2.133938832	0.133458944	1.00E-05	10	Atraid
0.347798603	0.13362943	1.00E-05	10	Nmd3
0.254087048	0.134088528	1.00E-05	10	Fbxl5
0.561101749	0.136832383	1.02E-05	10	Hdlbp
0.494621342	0.137282612	1.03E-05	10	Tnf
0.495666664	0.13914145	1.04E-05	10	Rufy1
0.652632926	0.140090316	1.05E-05	10	Sars
0.907921851	0.141684679	1.06E-05	10	Tfeb
0.769399731	0.142744752	1.07E-05	10	2010012O05Rik
0.516272129	0.143724249	1.08E-05	10	Rnf7
0.63156977	0.144167683	1.08E-05	10	Irf1
0.556534946	0.144410316	1.08E-05	10	Igsf6
0.597335858	0.145582718	1.09E-05	10	Ptpre
0.340894293	0.145646061	1.09E-05	10	Arhgef2
0.528781208	0.146870785	1.10E-05	10	Mrpl18
0.382101131	0.148426515	1.11E-05	10	Lrwd1
0.291028221	0.149044286	1.12E-05	10	Tsnax
0.667138447	0.149984117	1.12E-05	10	Hpgds
0.594098926	0.152194768	1.14E-05	10	Eprs
0.435680339	0.152898349	1.15E-05	10	Dph3
0.494715973	0.153682239	1.15E-05	10	Trappc1
0.516502758	0.153965087	1.15E-05	10	Grb2
0.787317499	0.154333497	1.16E-05	10	Wdfy3
0.471548798	0.15505262	1.16E-05	10	Zc3hav1
0.44860438	0.155560282	1.17E-05	10	Mex3c
0.499030978	0.155690883	1.17E-05	10	Baiap2
0.441660551	0.158579846	1.19E-05	10	Elmo1
0.539994981	0.159084554	1.19E-05	10	Thy1
0.314780638	0.159700764	1.20E-05	10	Celf1
0.278670738	0.162343427	1.22E-05	10	Tiam1
0.455638218	0.163739578	1.23E-05	10	Ltbp3
0.372227533	0.164010633	1.23E-05	10	Parp1
0.360399821	0.165494767	1.24E-05	10	Pla2g15
0.3621846	0.169688827	1.27E-05	10	Rap2c
0.483234586	0.172669132	1.29E-05	10	Tgif2
0.479829238	0.175231299	1.31E-05	10	Cyb561d2
0.518101209	0.176307116	1.32E-05	10	Trafd1
0.293854932	0.176439839	1.32E-05	10	Chka
0.603797796	0.177013644	1.33E-05	10	Ssr2
0.355042775	0.177417347	1.33E-05	10	Ovca2
0.503349494	0.181226524	1.36E-05	10	Hint1
1.29316755	0.184852067	1.38E-05	10	Creb3l1
0.337633805	0.187604192	1.41E-05	10	Setd8
0.641874491	0.191351383	1.43E-05	10	Mrps14
0.419628762	0.192491246	1.44E-05	10	Dusp7
-2.081506715	0.197540603	1.48E-05	10	Gas7
0.502248729	0.197863459	1.48E-05	10	Hace1
0.343887698	0.199170759	1.49E-05	10	Mvp
0.457910203	0.199610735	1.49E-05	10	Smox
0.265950232	0.200050758	1.50E-05	10	Enc1
0.341887348	0.202115716	1.51E-05	10	Srrd
-1.529375124	0.206574572	1.55E-05	10	Asah1
0.638133971	0.207924029	1.56E-05	10	Lamtor4
0.38036367	0.210519544	1.58E-05	10	Hars
0.641104899	0.215032428	1.61E-05	10	Chmp2b
0.595948783	0.217483406	1.63E-05	10	Galt
0.408273535	0.218821939	1.64E-05	10	Lclat1
0.586867892	0.222733794	1.67E-05	10	Txnl1
0.490687021	0.223057735	1.67E-05	10	Smg5

0.415149598	0.223881317	1.68E-05	10	Map1lc3a
0.582025285	0.224178515	1.68E-05	10	Thoc7
0.254122855	0.22424703	1.68E-05	10	Sord
0.276341704	0.224283963	1.68E-05	10	Eed
0.560119045	0.226806672	1.70E-05	10	Fbxw11
-2.074743021	0.232292405	1.74E-05	10	Ugp2
0.429261019	0.234825819	1.76E-05	10	Sucla2
-0.457817319	0.241890552	1.81E-05	10	Ftl1
0.534836981	0.241990294	1.81E-05	10	Ccni
0.547624251	0.245158573	1.84E-05	10	Vps26a
0.423503427	0.245852872	1.84E-05	10	Stk40
0.306989698	0.246968661	1.85E-05	10	Rbm38
0.630706028	0.249324686	1.87E-05	10	Acaa2
0.342254058	0.251706901	1.89E-05	10	Ptdss2
0.572452728	0.252372725	1.89E-05	10	Eif4a3
0.468428266	0.253725674	1.90E-05	10	Shisa5
0.509064355	0.25543276	1.91E-05	10	Phpt1
-1.2759464	0.256290197	1.92E-05	10	Rps21
0.287224302	0.256409079	1.92E-05	10	Pttg1
0.460581926	0.256694767	1.92E-05	10	Basp1
0.496187781	0.256852732	1.92E-05	10	Spag9
0.438549134	0.260225352	1.95E-05	10	Ptpn7
1.064459378	0.262137091	1.96E-05	10	Mtch1
0.515619551	0.263503794	1.97E-05	10	Pcif1
0.533072207	0.26900697	2.01E-05	10	Psmd8
-1.407193941	0.279078707	2.09E-05	10	Lyz1
-2.063873664	0.280850187	2.10E-05	10	Cmtm3
-0.853947601	0.291925152	2.19E-05	10	Mir6236
0.504175883	0.29362645	2.20E-05	10	Smcr8
0.52881606	0.300752547	2.25E-05	10	Cct3
0.416244148	0.3018043	2.26E-05	10	Elp5
0.501837829	0.302716215	2.27E-05	10	Wdr37
0.268090085	0.303338106	2.27E-05	10	Morf4l2
0.478801351	0.304439877	2.28E-05	10	Litaf
0.6266086	0.310403157	2.32E-05	10	Amdhd2
0.281079983	0.310555635	2.33E-05	10	Sash3
0.441752627	0.321976838	2.41E-05	10	Nek7
0.397471574	0.322114439	2.41E-05	10	Shkbp1
0.397626885	0.323837296	2.43E-05	10	Casp8
0.559367115	0.335290834	2.51E-05	10	Rilpl2
0.637398681	0.335684957	2.51E-05	10	Atxn7l3b
0.583437999	0.336548652	2.52E-05	10	Eif6
0.415445731	0.336724852	2.52E-05	10	Dmxl1
0.447758644	0.341495526	2.56E-05	10	Ash1l
0.560738212	0.341940923	2.56E-05	10	Vps37b
0.545702551	0.344599661	2.58E-05	10	Fam168a
0.278162226	0.349281384	2.62E-05	10	Ccdc53
0.514196302	0.350153615	2.62E-05	10	Dock2
0.325031522	0.358600226	2.69E-05	10	Col1a1
0.695847489	0.362086426	2.71E-05	10	Bloc1s1
0.495189953	0.363138903	2.72E-05	10	Acot13
0.612826334	0.367907835	2.76E-05	10	Fcrls
0.260760613	0.36947562	2.77E-05	10	Arfgef2
0.358504238	0.373049216	2.79E-05	10	Esf1
0.39170374	0.38476619	2.88E-05	10	Picalm
0.618507435	0.38782499	2.90E-05	10	Poldip3
0.314484347	0.389917101	2.92E-05	10	Rbm26
0.333951378	0.389982208	2.92E-05	10	Cpsf1
0.430149915	0.411959486	3.09E-05	10	Nemf
0.495929856	0.413217867	3.09E-05	10	Trim44
0.718478545	0.415407365	3.11E-05	10	Rbbp7

0.51115742	0.415450056	3.11E-05	10	Minos1
0.555133144	0.418524631	3.13E-05	10	Gnptab
0.335403934	0.420635464	3.15E-05	10	Iws1
0.481275712	0.423982694	3.18E-05	10	Psd3
0.355018492	0.434242503	3.25E-05	10	Slc23a2
0.311711402	0.436899236	3.27E-05	10	Zc3hc1
0.338975367	0.438546578	3.28E-05	10	Apmap
0.708697401	0.440331058	3.30E-05	10	Gch1
0.531083941	0.443010119	3.32E-05	10	Gns
0.276174466	0.447910085	3.35E-05	10	Klhl6
0.27216912	0.448670685	3.36E-05	10	Rnf139
0.487267175	0.450448682	3.37E-05	10	Reps1
0.353393742	0.460704367	3.45E-05	10	Dhrs1
0.644819687	0.463089128	3.47E-05	10	Nkg7
0.392438437	0.465188591	3.48E-05	10	Nfam1
0.527635652	0.465288794	3.48E-05	10	Mtmr10
0.344639179	0.467765925	3.50E-05	10	Rad21
0.480809051	0.470184746	3.52E-05	10	Copz1
0.361174025	0.475990634	3.56E-05	10	Slc12a6
0.51018671	0.476898879	3.57E-05	10	Ufm1
-1.745793838	0.478260624	3.58E-05	10	Hpcal1
0.466693068	0.479350935	3.59E-05	10	Ccl12
-0.649837923	0.481044408	3.60E-05	10	Hsp90ab1
0.54811487	0.482049265	3.61E-05	10	Tmem43
0.493425237	0.487006607	3.65E-05	10	Hn1
0.633205981	0.491327364	3.68E-05	10	Lhfpl2
0.821392725	0.507138699	3.80E-05	10	Lars
0.34338066	0.507165513	3.80E-05	10	Tlr8
0.343032168	0.509494205	3.82E-05	10	Aldh9a1
0.266418329	0.510241524	3.82E-05	10	Nup50
0.516806204	0.510311735	3.82E-05	10	Bud31
0.66331493	0.514640628	3.85E-05	10	Spns1
0.479350893	0.517275332	3.87E-05	10	Nrbp1
-2.051408018	0.517717841	3.88E-05	10	Cnpy2
0.314532067	0.518846044	3.89E-05	10	Arpc1a
0.296816474	0.520992297	3.90E-05	10	Tomm70a
0.325073829	0.525649742	3.94E-05	10	Sec31a
0.550001695	0.527145052	3.95E-05	10	Eif1ax
0.357905814	0.534833886	4.01E-05	10	Dcxr
0.618973073	0.536251065	4.02E-05	10	Kdm7a
0.434138195	0.536293903	4.02E-05	10	Gpr35
0.584171828	0.537432309	4.03E-05	10	Ilk
0.337654537	0.537777153	4.03E-05	10	Ppp1r7
0.477449158	0.538495998	4.03E-05	10	Stra13
1.046872188	0.559549209	4.19E-05	10	Ubp1
0.385034222	0.561475337	4.21E-05	10	Myd88
0.355251456	0.58380809	4.37E-05	10	Tmem134
0.485330722	0.592164142	4.44E-05	10	Txnl4a
0.308168067	0.601412667	4.50E-05	10	Endod1
0.467533392	0.60316641	4.52E-05	10	Chd7
0.331659536	0.604144828	4.52E-05	10	Nap1l4
0.588885012	0.60441754	4.53E-05	10	Anxa1
0.425540481	0.614015794	4.60E-05	10	Trmt10b
0.453283016	0.627762473	4.70E-05	10	Rnf187
0.58645452	0.644107406	4.82E-05	10	Osbpl8
0.539264772	0.647875567	4.85E-05	10	H13
0.482963147	0.648229582	4.85E-05	10	Hsd17b12
-1.158324738	0.661542007	4.95E-05	10	Comt
-0.410576251	0.668492631	5.01E-05	10	Calm1
0.472842725	0.674722096	5.05E-05	10	Ctbp1
0.549150731	0.682811814	5.11E-05	10	Gpr137b-ps

0.305358756	0.686345245	5.14E-05	10	Wdr45b
0.331312494	0.689354196	5.16E-05	10	Dgkz
0.477838643	0.692269948	5.18E-05	10	Slfn2
0.387046726	0.692934975	5.19E-05	10	Dnajb11
0.446532353	0.697087824	5.22E-05	10	Rab11b
0.500931116	0.735899509	5.51E-05	10	Wwc2
0.28262243	0.73865066	5.53E-05	10	Taf7
0.539387096	0.74254736	5.56E-05	10	Ubap2
0.288964946	0.755886461	5.66E-05	10	Arf2
-0.942918728	0.756035746	5.66E-05	10	Gm1821
0.338190745	0.756702079	5.67E-05	10	Sertad2
-2.030272469	0.768841325	5.76E-05	10	Fam89b
0.359645801	0.770945216	5.77E-05	10	Coa5
0.29551179	0.77642605	5.82E-05	10	Ube2j1
0.349225859	0.780666437	5.85E-05	10	Zc3h13
0.334494619	0.782943511	5.86E-05	10	Chordc1
0.423251292	0.784783987	5.88E-05	10	Cflar
0.272704425	0.791448545	5.93E-05	10	Ptbp2
0.44534733	0.793344304	5.94E-05	10	Rpap3
0.308044204	0.81725222	6.12E-05	10	Sdhc
0.298011371	0.820587754	6.15E-05	10	Lrch3
0.35415181	0.820771421	6.15E-05	10	C1qbp
0.267983035	0.82429489	6.17E-05	10	Spag7
-0.715961858	0.826554525	6.19E-05	10	Rpl14
0.290284208	0.830181887	6.22E-05	10	Tab3
0.519281949	0.834851357	6.25E-05	10	Trip12
0.491947843	0.843095151	6.31E-05	10	Rpp21
0.517563993	0.850859625	6.37E-05	10	Rnh1
0.499199844	0.856313234	6.41E-05	10	Polr2f
1.310902442	0.868363752	6.50E-05	10	Mgp
0.321500736	0.882612653	6.61E-05	10	Peo1
0.980316564	0.897418877	6.72E-05	10	Gm4832
0.323384851	0.909993269	6.82E-05	10	Sltm
0.31110059	0.917839583	6.87E-05	10	Rbm8a
0.535260357	0.930534119	6.97E-05	10	Sdf4
-1.851478704	0.936684584	7.02E-05	10	Nipa2
-2.098694992	0.940874751	7.05E-05	10	Tmbim4
0.549816478	0.96263062	7.21E-05	10	Plbd1
0.26749811	0.96819569	7.25E-05	10	Raph1
-0.458057132	0.979388287	7.34E-05	10	Malat1
0.532728259	0.979396352	7.34E-05	10	Dnttip2
-1.950610117	1	7.50E-05	10	Blvra
0.441737996	1	7.50E-05	10	Usp16
0.264068736	1	7.55E-05	10	Col4a3bp
-1.925277507	1	7.59E-05	10	Ppp1r11
-0.875146431	1	7.63E-05	10	Junb
0.450027443	1	7.69E-05	10	Gspt1
0.466102045	1	7.83E-05	10	Ifi35
0.585239008	1	7.87E-05	10	Rbx1
0.466918321	1	7.90E-05	10	Pbrm1
0.368389252	1	7.92E-05	10	Med13
0.558993715	1	7.93E-05	10	Bag6
0.416947252	1	7.94E-05	10	Arhgap22
0.518305274	1	8.06E-05	10	H2-DMa
0.388360773	1	8.13E-05	10	Rpn1
0.335834167	1	8.15E-05	10	Sgk3
0.372223613	1	8.22E-05	10	Zfr
0.460316582	1	8.22E-05	10	Cnppd1
0.369747026	1	8.23E-05	10	Fbxw5
0.524446178	1	8.24E-05	10	H2-DMb1
0.381757972	1	8.29E-05	10	Pld2

-2.088557375	1	8.36E-05	10	Irf2
-0.928533854	1	8.47E-05	10	Rpl18a
0.461160895	1	8.48E-05	10	Plin2
0.457984644	1	8.53E-05	10	Gsto1
0.406041561	1	8.54E-05	10	Sh3pxd2b
0.44847872	1	8.58E-05	10	Slc25a39
-0.436095718	1	8.66E-05	10	NP-904340.1
0.461687322	1	8.72E-05	10	Csf2ra
-0.340801256	1	8.78E-05	10	Fth1
0.371993136	1	8.85E-05	10	Spty2d1
0.638993947	1	9.05E-05	10	Fundc2
0.409941495	1	9.06E-05	10	Aph1c
0.598815743	1	9.16E-05	10	Npc1
0.280787893	1	9.18E-05	10	Hscb
0.37356912	1	9.22E-05	10	Micu3
0.56076009	1	9.23E-05	10	Eva1b
0.27194707	1	9.26E-05	10	Hnrnph2
-1.984311634	1	9.49E-05	10	F11r
0.388899149	1	9.76E-05	10	Tnfaip1
0.517161061	1	9.85E-05	10	Il7r
0.474819291	1	0.0001012	10	Nckap1l
0.489724209	1	0.0001015	10	Lonrf3
-0.762311045	1	0.0001015	10	H2-K1
0.310387801	1	0.0001024	10	Gmfb
0.461717052	1	0.0001035	10	Otud5
-0.570288434	1	0.000104	10	Rps4x
0.3553659	1	0.0001055	10	Ube3a
0.541721573	1	0.0001064	10	Mgrn1
0.306185544	1	0.000107	10	Rnf138
0.35935606	1	0.0001076	10	Smc3
0.336016785	1	0.0001095	10	Spg21
0.448177501	1	0.0001102	10	Brd4
0.318476845	1	0.0001105	10	Sp4
0.328828882	1	0.0001108	10	Dock5
0.442059274	1	0.0001112	10	Slc39a7
0.893746653	1	0.0001121	10	Zeb1
0.280731059	1	0.0001122	10	Slc35f6
0.392928634	1	0.0001169	10	Inpp5d
0.363031835	1	0.0001177	10	Fkbp8
-1.983385892	1	0.0001182	10	Slc25a12
1.350138066	1	0.0001201	10	8430419L09Rik
0.284792478	1	0.0001205	10	Bcl2l1
0.360751827	1	0.0001219	10	Ddx17
0.317799188	1	0.0001219	10	Gabarapl1
0.537732064	1	0.0001224	10	Zcchc6
0.435581453	1	0.0001241	10	Zmiz1
0.39729198	1	0.0001277	10	Adrm1
0.519172852	1	0.0001296	10	Tnfrsf12a
-1.902918789	1	0.0001306	10	Map3k8
0.401745362	1	0.0001314	10	Galnt6
0.394540567	1	0.0001318	10	Rexo2
0.408878985	1	0.0001328	10	Glrx3
0.497658847	1	0.000133	10	Anapc13
0.353635315	1	0.0001389	10	Poc1b
0.355889089	1	0.0001401	10	Bmp2k
0.296599576	1	0.0001419	10	Nasp
0.398831424	1	0.0001421	10	Uso1
0.424618679	1	0.0001473	10	Ccdc88a
0.401396473	1	0.0001509	10	2310022B05Rik
0.483044579	1	0.0001531	10	Axl
0.282368294	1	0.0001543	10	Havcr2

-1.772014533	1	0.0001567	10	Cmtm6
0.418297553	1	0.0001585	10	Fnip1
-1.795949397	1	0.0001591	10	Lpcat2
-1.829036727	1	0.0001628	10	Galnt7
-1.769068058	1	0.0001629	10	Yme1l1
0.332272367	1	0.0001667	10	Eif3a
-1.823463094	1	0.0001686	10	Dlgap4
0.443875544	1	0.0001694	10	Bcap31
0.375318661	1	0.0001696	10	B4galt5
-1.796429371	1	0.0001698	10	Stxbp2
0.292667283	1	0.0001725	10	Nol7
0.524289776	1	0.0001727	10	Csf2rb2
0.568289697	1	0.000175	10	Vav3
0.493578154	1	0.000176	10	Prpsap1
0.357284833	1	0.0001806	10	Skil
0.408119475	1	0.0001824	10	E030024N20Rik
-1.828631886	1	0.000184	10	Sec61a1
0.491783385	1	0.0001846	10	Fnip2
-1.661090979	1	0.0001849	10	Mcfd2
0.351439694	1	0.0001864	10	Tbc1d9
0.306664523	1	0.0001888	10	Farsa
0.326466517	1	0.0001897	10	Ubap2l
0.351729507	1	0.0001904	10	Idh3b
0.2746254	1	0.0001913	10	Nrd1
0.361109686	1	0.0001948	10	Tpp2
0.385470799	1	0.0001962	10	Zbtb7a
0.360099938	1	0.0001993	10	Cdc16
-0.377077785	1	0.0002052	10	Eef1a1
0.468344686	1	0.0002084	10	Lgals3bp
0.486562236	1	0.0002088	10	Ankrd11
0.457921378	1	0.0002089	10	Golga3
0.443503258	1	0.0002103	10	Txnrd1
0.437388647	1	0.0002109	10	Sel1l
0.296391744	1	0.000211	10	Acadsb
-1.117139515	1	0.0002125	10	Rps15a-ps6
0.337535896	1	0.0002222	10	Dag1
-2.043582818	1	0.0002227	10	Cfh
0.425175729	1	0.0002265	10	Azin1
0.251141556	1	0.0002298	10	Tial1
0.281845731	1	0.0002314	10	Bcl2l11
0.327424661	1	0.0002315	10	Arhgdia
0.468039747	1	0.0002321	10	Zc3h12c
0.44149093	1	0.0002333	10	D19Bwg1357e
-1.593412757	1	0.0002361	10	Trim8
-0.342285346	1	0.0002365	10	Fcer1g
0.385170149	1	0.0002366	10	Hipk1
0.265625258	1	0.000241	10	Rtf1
0.488794952	1	0.0002433	10	Tes
0.405102814	1	0.0002436	10	Sdha
0.406399611	1	0.000247	10	Ddx10
0.305249299	1	0.00025	10	Mkln1
0.453898339	1	0.000254	10	Gtf2h3
-0.378232422	1	0.0002542	10	H2-D1
0.54576978	1	0.0002561	10	Bri3
0.265144288	1	0.0002567	10	Mapk1
-1.382217666	1	0.0002612	10	Ndufa11
0.363094991	1	0.0002628	10	Bicd2
-1.74278444	1	0.0002638	10	Irak1
-0.410587066	1	0.0002653	10	Itm2b
0.452951291	1	0.0002656	10	Pik3r5
-1.904687433	1	0.0002668	10	Yipf1

-1.839998704	1	0.0002696	10	Rit1
0.592370961	1	0.0002704	10	Abca2
0.358162799	1	0.0002737	10	Scamp3
0.489883446	1	0.0002802	10	Gpd2
0.461022801	1	0.0002824	10	Lmna
-1.460407524	1	0.0002852	10	Atp5k
0.506991915	1	0.0002863	10	Strbp
-1.545582918	1	0.0002878	10	Ndufa6
0.336776284	1	0.0002894	10	Larp4b
0.402266107	1	0.0002909	10	Psmc2
0.401976609	1	0.0002917	10	Cops6
-0.437693912	1	0.000292	10	Mir682
0.444639014	1	0.0002924	10	0610010K14Rik
0.290685562	1	0.0002944	10	Snx10
0.268803703	1	0.0003016	10	Man1a
-0.300538797	1	0.0003026	10	Hcar2
0.674658192	1	0.0003044	10	Aup1
-1.971272599	1	0.0003045	10	Idnk
0.435239227	1	0.0003077	10	Psma2
-0.380902557	1	0.0003122	10	Tyrobp
-1.849060323	1	0.000313	10	Pkn1
0.275160846	1	0.0003169	10	Ptafr
-1.795518421	1	0.000318	10	Mpp1
-2.035901992	1	0.0003184	10	Tap1
0.398379732	1	0.000321	10	Synj1
-0.701984762	1	0.0003316	10	Ssu72
-0.410315503	1	0.000332	10	Ctss
-1.889896154	1	0.0003348	10	Usp47
0.377211716	1	0.0003392	10	Necap2
0.605515003	1	0.0003406	10	Tomm40
0.582865109	1	0.0003446	10	Tbxas1
-1.898513245	1	0.0003487	10	Elof1
0.307572822	1	0.0003499	10	Atp7a
-0.589438395	1	0.000351	10	Cfl1
0.590677148	1	0.0003568	10	Adh5
-2.009954901	1	0.0003615	10	Atp6v0d2
-1.27933489	1	0.0003628	10	Slamf9
0.437849773	1	0.0003663	10	Huwe1
-1.946459449	1	0.0003667	10	Mbp
0.345716208	1	0.0003713	10	Cpne3
0.31153216	1	0.000374	10	Rars
0.380663577	1	0.0003743	10	Fam32a
-1.839349716	1	0.0003814	10	Emg1
0.409446348	1	0.0003852	10	Abcf1
0.434621879	1	0.0003928	10	Sep.06
0.358124888	1	0.0003944	10	Cct6a
-1.525789174	1	0.0003953	10	Cul1
0.34946342	1	0.0003975	10	Mitf
0.443752522	1	0.0003981	10	Lasp1
-1.81199145	1	0.0003997	10	Anxa6
0.375964714	1	0.0004045	10	Napa
-1.815813458	1	0.0004079	10	Impact
0.415294344	1	0.0004139	10	Nars
0.454334159	1	0.0004221	10	Atp1a1
0.649531016	1	0.0004263	10	Nampt
-1.967752537	1	0.000427	10	Il21r
0.336369678	1	0.0004362	10	Cd164
-0.425545828	1	0.0004364	10	C1qb
-1.440421677	1	0.0004375	10	Grcc10
0.369747026	1	0.0004429	10	Sec11a
0.869461178	1	0.0004441	10	Sparc

-1.677208884	1	0.0004452	10	Daglb
0.462783276	1	0.000447	10	Myo1e
0.318865219	1	0.00045	10	Cbr2
0.433367215	1	0.0004509	10	Dync1i2
0.318552903	1	0.0004539	10	Tubb6
0.277281798	1	0.0004546	10	Cyb5b
0.551897364	1	0.0004594	10	Qsox1
0.318195398	1	0.000464	10	Coro2a
-1.993391241	1	0.000472	10	Ly6a
0.397807421	1	0.0004723	10	Actn1
0.554468338	1	0.0004732	10	Psap
0.295879313	1	0.0004755	10	Slc48a1
-2.024708271	1	0.0004774	10	Abca9
0.366190011	1	0.0004784	10	Xylt2
0.279466194	1	0.0004836	10	Ppp2r4
-1.514755228	1	0.0004909	10	Hsph1
0.360159718	1	0.0005008	10	Eif4h
0.315617309	1	0.0005123	10	Rp9
-1.629800607	1	0.0005231	10	Ccr5
0.276747205	1	0.0005293	10	Sec14l1
-1.868279822	1	0.0005314	10	Ubash3b
-1.73698791	1	0.0005375	10	Fkbp15
0.394272326	1	0.0005376	10	Plcl1
-1.716220493	1	0.0005378	10	Atg12
-1.720763877	1	0.0005471	10	Pon3
-1.056389169	1	0.0005475	10	Sat1
-1.755035636	1	0.0005476	10	Wls
-1.852685705	1	0.0005481	10	Mrps34
0.39802184	1	0.0005559	10	Cox7b
0.323403169	1	0.0005584	10	Wdr26
0.271330815	1	0.0005585	10	Dhx8
-1.565684361	1	0.0005587	10	Bdp1
0.364493789	1	0.0005601	10	Cox8a
-0.899274987	1	0.0005682	10	Rpl18
0.435265971	1	0.000572	10	Mdh2
0.28126384	1	0.0005756	10	Grpel1
0.366523181	1	0.0005789	10	Qk
0.41939352	1	0.0005818	10	Mrps16
0.359071849	1	0.0005847	10	Tln1
0.304778179	1	0.0005863	10	Tuba4a
0.567456912	1	0.0005888	10	Prdm15
0.426222491	1	0.0005977	10	Psmc3
-0.367983905	1	0.0006051	10	NP-904328.1
-1.690232662	1	0.0006092	10	Spcs3
0.381122003	1	0.0006149	10	Mbnl2
0.255804942	1	0.0006308	10	Pold1
0.37106055	1	0.0006312	10	Uqcr10
-0.25019634	1	0.000634	10	Ubac2
-1.805289321	1	0.000636	10	H2-Ke6
0.307318047	1	0.0006493	10	Vps51
-1.381605129	1	0.0006673	10	Rhob
0.271410493	1	0.0006751	10	Ythdc1
0.353821245	1	0.000676	10	Ube3c
-1.8857576	1	0.0006778	10	Dcaf11
-1.760861171	1	0.0006794	10	Tbc1d22a
0.359515134	1	0.0006883	10	Clec12a
1.430816167	1	0.000704	10	Rassf1
0.253127059	1	0.0007062	10	Trp53inp2
0.37112981	1	0.0007067	10	Snrpb
0.278692096	1	0.000707	10	Ankrd40
-1.777556519	1	0.0007119	10	Smim11

0.426180245	1	0.0007126	10	Paf1
-1.791118238	1	0.0007131	10	Coq10b
0.380372006	1	0.0007269	10	Rab1
0.351916635	1	0.0007282	10	Skap2
0.36688455	1	0.0007325	10	Tlr2
-1.163722529	1	0.0007442	10	Gm6402
0.2647133	1	0.0007451	10	Ccl7
0.432629255	1	0.0007454	10	Cuta
0.410097409	1	0.0007533	10	Dynlrb1
0.38447341	1	0.0007583	10	Ppib
0.258234026	1	0.0007702	10	Ptplad2
-1.692742749	1	0.0007747	10	Samhd1
-1.717159821	1	0.0007762	10	Ppp1r9b
-1.751938576	1	0.0007809	10	Lin7c
0.298900723	1	0.0007904	10	Park7
-1.620319427	1	0.000802	10	Rgl2
0.336205376	1	0.0008024	10	Coa3
0.299366229	1	0.000817	10	Yipf3
-0.749943865	1	0.0008175	10	Alox5ap
-1.915507584	1	0.0008177	10	Hes1
-1.266359228	1	0.0008199	10	Tgif1
-1.297369999	1	0.000821	10	Rpl38
-1.864825232	1	0.0008229	10	Dok2
-1.859388163	1	0.0008427	10	Ptger4
-1.585781164	1	0.0008462	10	Gltscr2
0.438108905	1	0.0008468	10	Aip
-1.775837444	1	0.0008552	10	BC004004
-1.809391568	1	0.0008585	10	Pop5
-1.846913057	1	0.0008669	10	Tcerg1
-1.77511531	1	0.0008807	10	Cd38
-1.298784312	1	0.0008907	10	Egr1
-1.806449715	1	0.0008917	10	Fcgr4
0.360238626	1	0.0008951	10	Rps17
0.276168214	1	0.0008984	10	Derl1
-1.821385115	1	0.000903	10	Tnfaip8l2
0.370691354	1	0.0009081	10	Prpf40a
-1.783875911	1	0.0009181	10	Ncoa1
-1.760118928	1	0.0009378	10	Dpep2
0.443665753	1	0.0009405	10	Eea1
0.304964434	1	0.0009451	10	Cd86
-1.65762248	1	0.0009509	10	Adam10
-1.777719055	1	0.0009781	10	1500011K16Rik
0.520939206	1	0.0009869	10	Tnpo2
0.356938637	1	0.0009941	10	Cct4
0.595551508	1	0.0010146	10	Pmepa1
-0.481160303	1	0.001021	10	Rps15
0.37464683	1	0.0010439	10	Fam162a
-1.761160214	1	0.0010497	10	Naa60
-1.654090324	1	0.0010595	10	Apex1
-0.577268955	1	0.0010663	10	Pfn1
0.258000415	1	0.0010706	10	Ggnbp2
-1.054257003	1	0.0010754	10	Rhog
-1.665042413	1	0.0010843	10	Eapp
0.364950853	1	0.0010956	10	Clec4a3
-1.61406091	1	0.0010977	10	Hsd17b4
0.265043343	1	0.0011328	10	Tgoln1
0.30832387	1	0.0011447	10	Nfkbid
0.392848357	1	0.0011485	10	Mrpl54
0.388091028	1	0.0011539	10	Vps28
-1.721563952	1	0.0011541	10	Triap1
-1.772253396	1	0.0011564	10	Kdm2a

0.320402947	1	0.0011579	10	Polr2j
0.279609083	1	0.0011614	10	Herpud1
-2.016737235	1	0.0011747	10	C4b
-1.666654017	1	0.0011877	10	Hook3
-1.694685531	1	0.0012188	10	Pou2f2
-1.684538039	1	0.0012369	10	Prcp
0.35516952	1	0.0012376	10	Tgfbr1
0.271122746	1	0.0012429	10	Ctdsp2
-0.459408252	1	0.0012701	10	1110004F10Rik
-1.635648179	1	0.0012783	10	Sptssa
0.291604427	1	0.0012861	10	Mmp12
0.294579236	1	0.0012876	10	Clta
0.331628531	1	0.0012955	10	Gpr108
-1.772831459	1	0.001302	10	Fam126a
-1.695013965	1	0.0013213	10	Sep.09
0.330754095	1	0.0013259	10	Tpr
-1.506211869	1	0.0013325	10	Pqlc3
0.552416089	1	0.0013348	10	Fam53c
0.286911698	1	0.001336	10	Baz1a
-1.596726343	1	0.0013364	10	Il13ra1
-1.675704663	1	0.0013468	10	Hk3
0.287299919	1	0.0013597	10	Dhx40
0.306385925	1	0.0013779	10	Leprotl1
0.437391543	1	0.0013902	10	Ncaph2
0.404051273	1	0.0013926	10	Aimp1
0.286572902	1	0.0014325	10	Ptp4a2
0.308927384	1	0.0014336	10	Cpd
-1.429888503	1	0.0014351	10	Cebpb
0.336249788	1	0.001441	10	Ube2k
-1.50102555	1	0.0014555	10	Rnf128
-1.711667157	1	0.0014598	10	Rnaseh2c
0.345864218	1	0.0014609	10	Ezr
-1.049004483	1	0.0014624	10	Ywhah
0.593527092	1	0.0014743	10	Plekhm1
-0.922018871	1	0.0014951	10	Mir703
-1.553274479	1	0.0015005	10	Nolc1
-1.595884946	1	0.0015049	10	Vcpip1
0.254451377	1	0.0015049	10	Ppm1g
-1.677362521	1	0.0015064	10	Gars
0.335171978	1	0.0015313	10	Pet100
0.378584034	1	0.001552	10	Syk
0.259174205	1	0.0015528	10	Frmd4b
0.268718069	1	0.0015666	10	Lsm4
-1.740850716	1	0.0015806	10	Kat6a
0.367021848	1	0.001601	10	Lipa
1.063574771	1	0.0016056	10	2310047M10Rik
0.387812724	1	0.001614	10	Chd8
0.317143007	1	0.0016195	10	Glt25d1
-1.872490575	1	0.0016222	10	Stard8
-1.573885463	1	0.0016247	10	Ccpg1
-1.833526072	1	0.0016281	10	St8sia4
-1.684081455	1	0.0016337	10	Itpk1
0.564914385	1	0.0016352	10	Pyroxd2
-1.580761413	1	0.0016355	10	Mtf2
-1.033796969	1	0.0016484	10	Eno1b
-1.792362157	1	0.0016515	10	Snrnp200
-1.71518297	1	0.0016649	10	Lbh
-1.652902566	1	0.0016931	10	Myo7a
-1.537694008	1	0.0016978	10	Rab12
-1.873212671	1	0.0016983	10	Samsn1
0.358047372	1	0.0017159	10	Nme1

-1.680226094	1	0.0017162	10	Lima1
0.320505369	1	0.0017212	10	Eif4g2
-1.651218422	1	0.0017374	10	Arl8a
-1.905688936	1	0.0017461	10	Ccr7
-1.720581127	1	0.001761	10	Atxn10
-1.394434219	1	0.0017638	10	Fau
0.265944787	1	0.0017754	10	Hspa9
0.251332571	1	0.0018171	10	Arfgef1
-1.642741605	1	0.0018187	10	Tnfrsf11a
-1.128423193	1	0.0018188	10	Agpat5
0.255501639	1	0.0018494	10	Fn1
0.325610431	1	0.0018575	10	Gm2a
0.310800199	1	0.001869	10	Rpn2
0.32729175	1	0.0018727	10	Cd2
0.303766874	1	0.0018844	10	Ifi30
-1.645094943	1	0.0018897	10	Abcc1
-1.654558348	1	0.001915	10	Ubtd1
0.295988493	1	0.0019362	10	Cyb5r4
0.333727911	1	0.0019364	10	Txndc17
-1.599761794	1	0.0019391	10	Ypel5
-1.596274734	1	0.0019482	10	Csnk1g3
-1.520461184	1	0.0019502	10	Arid1b
-0.405771639	1	0.001986	10	Rpph1
-1.683701152	1	0.002012	10	Slc15a4
-0.596080103	1	0.0020137	10	Msn
-1.542136272	1	0.0020442	10	Ndufa9
-1.623412612	1	0.0020574	10	Cat
-1.619070853	1	0.00206	10	Pdpn
0.30187886	1	0.0020945	10	2010111I01Rik
-1.594773217	1	0.002115	10	Usp48
-1.685228991	1	0.0021207	10	Ppp4c
0.367646636	1	0.0021675	10	Chchd1
-1.715874772	1	0.0022028	10	Ephx1
-0.308872054	1	0.0022083	10	Oxr1
-0.471624375	1	0.0022311	10	Lyz2
-1.537885843	1	0.0022313	10	Ppm1a
0.345590606	1	0.0022515	10	Pim1
-1.475826597	1	0.0022551	10	Tnip2
-1.751621669	1	0.0022573	10	Bcl10
-1.324869268	1	0.0022724	10	Cast
0.292702194	1	0.0022818	10	Rspry1
-1.708855322	1	0.0023076	10	Mrpl35
0.517365605	1	0.0023228	10	Gm12338
-1.511316091	1	0.0023289	10	Sphk2
-1.58569677	1	0.0023324	10	Rabggtb
0.353188947	1	0.0023412	10	Mtss1
-1.434538448	1	0.0023525	10	Iqsec1
-1.619070853	1	0.0023563	10	Rpl27a
0.267883988	1	0.002362	10	Jkamp
0.332732626	1	0.0023664	10	Sdcbp
-0.253683415	1	0.0023896	10	Rassf2
0.365127248	1	0.0023924	10	Glipr1
-0.627325786	1	0.0024017	10	Dusp1
-1.585499823	1	0.0024154	10	4833420G17Rik
-1.598804656	1	0.0024183	10	Zzef1
0.350733515	1	0.0024197	10	Pfdn4
-1.619959291	1	0.002422	10	Med11
-1.493947919	1	0.0024306	10	Pcyox1
-1.562165826	1	0.0024488	10	Tec
0.389771051	1	0.0024735	10	Ran
-1.435678156	1	0.0025054	10	Pik3cb

0.312894491	1	0.0025152	10	Tpm4
-0.781281324	1	0.0025199	10	Rps2
-1.605584343	1	0.002522	10	Hsd3b7
0.313467282	1	0.0025253	10	Cstb
-1.682683444	1	0.0025427	10	Usp15
-1.076715014	1	0.002571	10	2900060B14Rik
-1.41939431	1	0.0025865	10	Tspan31
-1.638128152	1	0.0025897	10	Sbf2
0.328668654	1	0.0026052	10	Mrc1
-1.196159093	1	0.0026166	10	Akap13
-1.403944627	1	0.0026324	10	Slc16a6
0.302632585	1	0.0026761	10	Fuca1
-1.24366801	1	0.0026886	10	Asph
-2.007933275	1	0.0027044	10	Mgl2
0.681030927	1	0.0027047	10	Pold4
-1.991658178	1	0.0027479	10	Ltc4s
0.293101068	1	0.0028225	10	Ctc1
-1.522706217	1	0.0028299	10	Nipsnap3b
0.307266848	1	0.0028449	10	Cndp2
-1.032695241	1	0.0028456	10	Rps16
-1.518923905	1	0.0028462	10	2510009E07Rik
-1.517659949	1	0.0028775	10	N6amt2
0.27688112	1	0.0029109	10	Ywhab
-1.468184474	1	0.00294	10	Cds2
0.273538361	1	0.0029754	10	Srsf3
-1.674705906	1	0.0029826	10	Ddx47
-0.300248957	1	0.0029957	10	Atp11b
0.28454016	1	0.0030051	10	Ppt1
-0.995194876	1	0.0030261	10	Adprh
-1.691054824	1	0.0030448	10	Acvrl1
-1.646066512	1	0.0030459	10	1600002K03Rik
-0.605097666	1	0.0030581	10	Lamp2
-1.61076487	1	0.0030809	10	Sumf1
0.299307885	1	0.0030877	10	Stom
-1.606934782	1	0.0030897	10	Ccdc9
-1.602877982	1	0.003109	10	Vps13c
-1.420788686	1	0.0031183	10	Chmp1b
-1.650453295	1	0.0031226	10	Nsun2
0.654635043	1	0.0031258	10	Ext1
-1.29798132	1	0.0031512	10	Hnrnpl
-1.396168528	1	0.0031715	10	Kcnab2
-2.043889092	1	0.0031948	10	Cd163
-1.190155253	1	0.0032034	10	Sumo1
-1.482385651	1	0.0032299	10	Commd6
-1.548993563	1	0.0032357	10	Fam114a2
-1.531887137	1	0.0032513	10	Rassf3
-0.315839595	1	0.0032537	10	Ccng2
0.268997976	1	0.0032654	10	Ctnna1
0.26708469	1	0.0032843	10	Cox17
-1.533968067	1	0.0032987	10	Gyg
-1.661745344	1	0.0033119	10	Toporsos
-1.333273055	1	0.0033412	10	Igf1
-1.692957241	1	0.0033484	10	Bcl2
-1.58879972	1	0.0033506	10	Slc35b2
-1.156893029	1	0.0033569	10	Ap1s1
-1.579176941	1	0.0033578	10	Jade2
-1.587049734	1	0.0034	10	Acox3
0.330774016	1	0.0034469	10	AF251705
-1.612126602	1	0.0034881	10	Tpgs1
-1.383603367	1	0.0035263	10	Ube2q1
-1.634036712	1	0.003551	10	Orai1

-1.497646506	1	0.0035569	10	Vps18
-1.538107145	1	0.0035674	10	Gstp1
-1.745963855	1	0.0035821	10	Fkbp4
0.339853553	1	0.0035831	10	Sgpl1
-1.597118752	1	0.0035873	10	Mndal
0.392971366	1	0.0035928	10	Plekhg2
-1.569617019	1	0.0036232	10	Snrpa1
-0.97729686	1	0.0036814	10	Marcks
-1.404634758	1	0.0036881	10	Cep250
-1.482914871	1	0.0036957	10	Ahcyl2
0.313289303	1	0.0036966	10	Ehd4
-1.253305571	1	0.0037395	10	AI462493
-1.553187296	1	0.0037531	10	Tmem135
-1.377483221	1	0.00379	10	Taok3
0.287366864	1	0.0038228	10	Timm13
-1.473706299	1	0.0038257	10	C1d
-1.583528213	1	0.0038377	10	Alox5
-1.560305743	1	0.0038387	10	Nr4a1
-1.603839337	1	0.0038665	10	Tlr13
-1.700192276	1	0.0039075	10	Pip5k1a
0.304559289	1	0.0039694	10	Fmnl1
-1.421949183	1	0.0039751	10	Eif2b5
-1.540273323	1	0.0040118	10	Emc1
-1.468848705	1	0.0040561	10	Ppm1b
-0.74124852	1	0.0040706	10	Rhoa
-1.539964156	1	0.0041251	10	Klhdc4
-0.825943377	1	0.004171	10	Jund
0.26988482	1	0.0041993	10	Sep.07
-1.461517782	1	0.004201	10	Rc3h2
-1.742151663	1	0.0042086	10	Sdccag3
-1.304182457	1	0.00421	10	Ptges3
-1.659326374	1	0.0042143	10	Trnau1ap
0.80413246	1	0.0042369	10	Puf60
-1.568060329	1	0.004326	10	Bst1
0.391017008	1	0.0043424	10	Tubb5
-1.671072582	1	0.0043566	10	Ubtf
-1.441573223	1	0.0043944	10	Cant1
-0.320135379	1	0.0044239	10	Dpagt1
-1.583640982	1	0.0044518	10	Itch
-1.47569517	1	0.0044557	10	Gcc2
-1.203808856	1	0.0044624	10	Coro1a
-1.491511597	1	0.0045103	10	Vps36
-1.491823132	1	0.0045228	10	Dnlz
-1.555834259	1	0.0045748	10	Fbxl3
-1.655783088	1	0.0046025	10	Adap2
0.276152063	1	0.0046108	10	Ndufb10
-1.43072522	1	0.0046207	10	Trak2
0.265130034	1	0.0046363	10	Rgs1
-1.686927765	1	0.0046694	10	Tppp3
-1.548584899	1	0.004694	10	Ipo9
-1.470175845	1	0.004708	10	Dld
-1.433709955	1	0.0047795	10	Traf6
-1.584337043	1	0.0047838	10	Gga1
0.259964923	1	0.0048261	10	H2-Ab1
-0.612111917	1	0.0048935	10	Rps3a1
0.40463784	1	0.0049156	10	Stam2
-1.391090533	1	0.0049395	10	Ppp6c
-1.663438242	1	0.0049663	10	Batf3
-1.422413005	1	0.0049691	10	Kcnk13
-1.485094946	1	0.0049715	10	Wdr48
-1.209807398	1	0.0050037	10	Uap1l1

-1.539286599	1	0.0050039	10	Dlst
-1.675772241	1	0.0050045	10	Mocs2
0.383652267	1	0.0050279	10	Ssr1
-1.487921957	1	0.00506	10	Uba7
-1.543667864	1	0.0050635	10	1700123O20Rik
-1.491823132	1	0.0050638	10	Tmem19
-1.552082323	1	0.0050884	10	Osbpl9
-0.71637568	1	0.0051348	10	Mbnl1
-1.457943957	1	0.00515	10	Pank2
-1.407023532	1	0.0051678	10	Casp1
-0.63722403	1	0.0051799	10	Rps18
-1.307850078	1	0.0051958	10	Bin2
-0.483956881	1	0.0052217	10	Mir692-1
-1.671113479	1	0.005295	10	Man2c1
0.627073326	1	0.005323	10	Sik1
-1.333481694	1	0.0053549	10	Bola3
-0.262679473	1	0.0054217	10	Fbxo30
-1.673134733	1	0.005424	10	Plxdc2
-0.379818515	1	0.0054274	10	Brpf3
-2.142279576	1	0.0054289	10	Lyve1
-1.500867121	1	0.0054473	10	Bmyc
-1.557537146	1	0.0054492	10	Pdcd2l
0.255218172	1	0.0054641	10	Mdm2
-1.441142527	1	0.0054839	10	Mgat4b
-1.516394393	1	0.0055448	10	Gxylt1
-1.369717504	1	0.0055541	10	Qrich1
-1.514426	1	0.005612	10	Skp1a
-1.340300764	1	0.0056396	10	Zfp644
-0.603632673	1	0.0056417	10	Gm6654
-1.697378345	1	0.0056444	10	Clec4b1
-1.533155487	1	0.0056487	10	Stard3nl
0.658105965	1	0.005654	10	Kxd1
-1.351314642	1	0.0056573	10	Olfml3
-1.307781225	1	0.0057397	10	Arhgef1
-1.533281873	1	0.0057754	10	Clcn4-2
-1.513878942	1	0.0058493	10	Ube2a
0.258234605	1	0.0058532	10	Rpl23
-1.602038812	1	0.0058553	10	Fibp
-1.468184474	1	0.0058554	10	Phyh
-1.463967399	1	0.0058632	10	Ccz1
-1.3404458	1	0.0058902	10	Ctage5
-0.766361897	1	0.0059222	10	Rps8
-1.532226338	1	0.0059885	10	Mxi1
-1.561025081	1	0.0060163	10	Milr1
0.369897277	1	0.0061217	10	Mcm3
-1.513576299	1	0.0061383	10	Rbck1
0.27169336	1	0.0061517	10	Zyx
-1.478978719	1	0.0061847	10	Nmi
0.253811679	1	0.0062059	10	Cd63
-1.090567193	1	0.0062551	10	Nt5dc2
-1.509823801	1	0.006284	10	Rassf5
-1.611088647	1	0.0062853	10	Ift20
-1.485965647	1	0.0062991	10	Sh3bp5
-1.601337426	1	0.0063082	10	Rora
-1.558510851	1	0.0063118	10	Eif2b2
0.769404327	1	0.0063196	10	Pygm
-1.633028686	1	0.0064581	10	Rab24
-1.497464521	1	0.0064688	10	Otub1
0.274924702	1	0.0064854	10	Cript
-1.517238274	1	0.0065281	10	Vrk2
-1.364176556	1	0.0065327	10	Dpy19l1

-1.469070017	1	0.0066461	10	Rab5a
-1.318931342	1	0.0066467	10	Angptl2
-1.371278337	1	0.0066933	10	Ppp3ca
-1.299235915	1	0.0066994	10	Hmox1
0.293927479	1	0.0067045	10	Rgs10
-1.406671004	1	0.0067244	10	Tra2a
-1.417997986	1	0.0067486	10	Prune2
-1.678255943	1	0.0067787	10	Itgb7
-1.458049252	1	0.0068035	10	Myo1c
0.782497624	1	0.0068638	10	Il4ra
-1.33018178	1	0.0068747	10	Chchd4
-1.441246818	1	0.0070018	10	Eps15l1
-1.346073148	1	0.0070102	10	Snhg6
-0.274129106	1	0.0070218	10	Akip1
-1.288724268	1	0.0070883	10	Smim7
-1.499794735	1	0.0071267	10	Psmg4
-1.523227133	1	0.0071299	10	C5ar1
-1.361525533	1	0.0071819	10	Txndc15
0.277477902	1	0.0072065	10	Senp5
-1.448831528	1	0.0072852	10	Extl3
-1.349819874	1	0.007299	10	3110043O21Rik
-1.471721953	1	0.00739	10	Atl2
-1.547650183	1	0.0073981	10	Usp8
-1.271271935	1	0.0074023	10	Dnajc19
-1.409905915	1	0.0074768	10	Pik3cg
-1.37590403	1	0.0075237	10	Hcfc1
0.252835783	1	0.0075329	10	Med28
0.267345538	1	0.0075926	10	Atp5b
-1.246740158	1	0.0076351	10	Cox7c
-1.502213634	1	0.0077933	10	Cmc1
-1.443398112	1	0.0078091	10	Swap70
-1.252687638	1	0.0078147	10	Ifngr1
-1.333228241	1	0.0080771	10	Tpra1
-1.453851908	1	0.0081444	10	Rufy3
-0.531701377	1	0.0081736	10	Oasl1
-1.454740876	1	0.0082154	10	BC028528
-1.487052958	1	0.0082166	10	Zc3h4
-1.429805046	1	0.0082254	10	Cdr2
-1.395224669	1	0.0082337	10	Tmem160
-1.625255378	1	0.0082467	10	Ctsk
-1.489574312	1	0.0082508	10	Csnk1e
0.313926	1	0.0082731	10	Aprt
-1.416650407	1	0.0082903	10	Usp19
-1.322012217	1	0.0083465	10	Ttc33
-1.525354795	1	0.0083649	10	Hip1
-1.347323617	1	0.0083664	10	Gtf2a1
-1.428269537	1	0.0083795	10	Plekhm3
-0.458762803	1	0.0084373	10	Rpl39
-1.951818875	1	0.0084718	10	Ifitm1
-0.342350075	1	0.0086472	10	Sun2
-1.518923905	1	0.0086579	10	St3gal6
-1.411690291	1	0.0086586	10	Pde1b
0.321511853	1	0.0087075	10	Prrc2c
0.636773303	1	0.0087078	10	Arf4
-0.279886533	1	0.0088359	10	Fam133b
-1.466632883	1	0.0089726	10	Ate1
-1.38533236	1	0.0089752	10	Pitrm1
-0.301993955	1	0.0090546	10	Chd9
-1.400851354	1	0.0091022	10	Inpp5f
-1.318159638	1	0.0091307	10	Rab28
-1.368345783	1	0.0091386	10	Parl

-0.821625262	1	0.0091853	10	Map3k1
-0.369827711	1	0.0092342	10	Sowahc
-1.896103844	1	0.009269	10	Spn
-1.461071752	1	0.0092815	10	Nkap
-1.460955752	1	0.0093198	10	Ccr1
-1.380395244	1	0.0093408	10	Ppp2r5a
-0.288207776	1	0.0094015	10	Arhgap26
-0.29341654	1	0.0094465	10	Snx30
-0.2561379	1	0.0094574	10	Was
-1.348322867	1	0.0094625	10	Nabp2
-1.407193941	1	0.0094747	10	Ap2b1
-1.287323644	1	0.0095556	10	Cnih1
-1.4534961	1	0.0095703	10	Rabl6
-1.324060874	1	0.0096074	10	Dguok
-1.373958977	1	0.0096111	10	Zcchc9
-1.362208536	1	0.0096167	10	Fam53b
-1.50897989	1	0.0096236	10	Tagap
-1.186300253	1	0.0096869	10	Wbp4
-0.279222323	1	0.0096933	10	Zcchc7
-1.514533328	1	0.0097008	10	Zdhhc9
0.27355731	1	0.0097325	10	Stx7
-0.255487152	1	0.0097453	10	Phax
-1.545924507	1	0.0097869	10	Mtmr12
-1.472403889	1	0.0098148	10	Ufsp2
-1.459819866	1	0.0099029	10	Eepd1
-1.536165711	1	0.0099186	10	Pnpla8
2.270730856	4.10E-163	3.07E-167	11	Defb47
1.403993938	4.55E-145	3.41E-149	11	Myom2
2.490354925	1.79E-144	1.34E-148	11	Pla1a
1.674488415	7.92E-130	5.93E-134	11	Gpr113
1.536659905	1.47E-113	1.10E-117	11	Btnl9
1.723072865	1.35E-107	1.01E-111	11	Amer2
2.69399807	3.79E-100	2.84E-104	11	Slc4a11
2.201891692	3.76E-83	2.82E-87	11	Ccdc173
1.579346826	2.70E-77	2.02E-81	11	Fsip1
1.372153332	1.02E-74	7.61E-79	11	Acsbg1
1.284466149	1.71E-62	1.28E-66	11	Edaradd
2.081004081	9.10E-61	6.81E-65	11	Hspa1l
2.06652943	1.74E-60	1.30E-64	11	Zkscan8
2.951377206	1.10E-59	8.21E-64	11	Tm7sf2
1.994731662	2.57E-55	1.92E-59	11	Kcnab3
1.563194201	2.60E-54	1.94E-58	11	Shcbp1l
1.467284934	5.21E-52	3.90E-56	11	Kcnh4
1.906983214	1.03E-51	7.68E-56	11	Scel
2.209097009	4.11E-51	3.08E-55	11	Asb1
1.302797926	1.53E-50	1.15E-54	11	Xkrx
0.494563045	1.76E-49	1.32E-53	11	Vmn1r24
0.750249306	3.93E-48	2.94E-52	11	Lrrc23
1.314779108	1.97E-47	1.47E-51	11	Snora23
1.58833774	2.41E-47	1.81E-51	11	Tmem220
1.271539681	7.61E-47	5.70E-51	11	Nefh
1.784043157	1.59E-46	1.19E-50	11	Myo6
1.034815669	1.23E-45	9.21E-50	11	Pde1c
3.365899645	3.68E-45	2.75E-49	11	Htr7
1.627421621	7.32E-45	5.49E-49	11	Serpinb1c
2.242992996	9.51E-44	7.12E-48	11	Nudt5
0.947827677	1.47E-43	1.10E-47	11	4930529C04Rik
2.600141102	3.72E-43	2.78E-47	11	F9
1.983461495	2.68E-42	2.01E-46	11	Shroom3
0.968388964	5.45E-41	4.08E-45	11	4932416H05Rik
1.269430021	1.04E-40	7.76E-45	11	Lmbr1

1.079716975	8.29E-40	6.21E-44	11	Ceacam16
1.278124759	1.59E-38	1.19E-42	11	1700012D14Rik
1.698906202	7.12E-38	5.34E-42	11	Clcn5
2.178419768	1.97E-36	1.47E-40	11	Zbtb25
2.500868607	2.77E-36	2.08E-40	11	Zfp618
2.480768493	2.69E-35	2.02E-39	11	Gpr56
1.086753064	4.57E-34	3.42E-38	11	Zfp773
1.958320707	5.95E-34	4.46E-38	11	Acn9
1.286467017	6.85E-34	5.13E-38	11	Adam11
0.683679058	7.43E-34	5.57E-38	11	Plscr5
2.237913673	2.28E-33	1.71E-37	11	Rrp1b
1.79532273	2.41E-31	1.81E-35	11	Frat1
2.826890878	3.03E-31	2.27E-35	11	Nupl1
1.33581551	3.89E-31	2.91E-35	11	Gm2447
1.420030937	5.48E-31	4.10E-35	11	Zfp933
2.058331844	1.02E-30	7.62E-35	11	Zbed5
2.047786461	1.87E-30	1.40E-34	11	Cnnm3
1.7836584	7.38E-30	5.53E-34	11	Prkcq
2.16945684	9.12E-30	6.83E-34	11	Oaf
1.087899239	1.35E-29	1.01E-33	11	Mei1
1.885635272	1.57E-29	1.18E-33	11	Mief2
1.069254804	1.81E-29	1.36E-33	11	Mall
1.646461762	1.14E-28	8.53E-33	11	Tnfsf8
2.803682414	1.13E-27	8.49E-32	11	Pgf
1.959824196	1.55E-27	1.16E-31	11	Clec16a
1.250709434	4.63E-27	3.46E-31	11	Aldh1b1
1.624683501	1.11E-26	8.30E-31	11	Tnk2
1.094528903	1.26E-25	9.47E-30	11	Ift81
1.254678678	1.54E-25	1.15E-29	11	Dak
1.20580566	1.43E-24	1.07E-28	11	Rasd1
1.976653813	1.43E-24	1.07E-28	11	Ifi205
1.271539681	1.62E-24	1.22E-28	11	Dnah8
1.457948932	1.77E-24	1.33E-28	11	Tjp3
1.324038647	5.92E-24	4.43E-28	11	Impdh2
1.678276451	6.69E-24	5.01E-28	11	Phf11c
1.018562889	4.92E-23	3.68E-27	11	Klhdc9
1.418185067	8.09E-23	6.06E-27	11	D17H6S53E
1.677511074	2.70E-22	2.02E-26	11	Fktn
2.1499836	3.16E-22	2.37E-26	11	Vdr
1.499521533	4.75E-22	3.56E-26	11	Suox
2.264702656	6.81E-22	5.10E-26	11	Cst7
1.560262847	9.27E-22	6.95E-26	11	Prepl
1.183635009	3.54E-21	2.65E-25	11	Cdhr3
1.646810255	4.33E-21	3.24E-25	11	Cxx1b
1.051874811	1.26E-20	9.43E-25	11	Snord16a
0.87872763	1.48E-20	1.11E-24	11	Irg1
1.840595121	2.12E-20	1.59E-24	11	St3gal3
0.948466239	3.47E-20	2.60E-24	11	Ccr9
1.424871523	4.12E-20	3.09E-24	11	Rgag4
1.553712307	7.00E-20	5.24E-24	11	Stmn4
1.817579294	1.68E-19	1.26E-23	11	Hdac10
2.187171146	2.54E-19	1.90E-23	11	Ccdc102a
1.777797764	2.59E-19	1.94E-23	11	Lcor
2.389221565	3.46E-19	2.59E-23	11	Mpp7
0.922690754	5.00E-19	3.74E-23	11	Mir324
1.722063128	6.08E-19	4.55E-23	11	Samd4
2.34164133	8.17E-19	6.12E-23	11	Ifitm6
1.569694313	1.07E-18	7.99E-23	11	Ppp1r1a
1.035570102	1.19E-18	8.89E-23	11	Slc25a32
1.611353622	3.32E-18	2.49E-22	11	Gm3414
1.454778353	3.56E-18	2.66E-22	11	Cep57l1

1.726455691	9.00E-18	6.74E-22	11	Alpk2
1.163027595	1.18E-17	8.84E-22	11	Cby1
1.141325676	1.18E-17	8.85E-22	11	Fam71f2
1.22010621	1.69E-17	1.27E-21	11	Gprc5a
1.553044012	1.79E-17	1.34E-21	11	Zfp628
1.330712754	5.97E-17	4.47E-21	11	Zfp958
2.08129453	1.02E-16	7.61E-21	11	Utp23
0.810712754	1.38E-16	1.03E-20	11	Cacna1b
0.827739526	1.41E-16	1.06E-20	11	Lsamp
1.62283356	1.99E-16	1.49E-20	11	Abcb8
1.982626823	2.61E-16	1.96E-20	11	Irf4
2.408693047	2.87E-16	2.15E-20	11	Mllt1
2.13881221	5.96E-16	4.47E-20	11	Zfp809
1.705612566	5.97E-16	4.47E-20	11	Ap5z1
1.830169461	6.12E-16	4.58E-20	11	Supv3l1
2.54208396	7.54E-16	5.65E-20	11	Mcemp1
1.523474479	9.76E-16	7.31E-20	11	Abca2
1.941621836	1.05E-15	7.88E-20	11	Alkbh7
1.370302199	1.13E-15	8.44E-20	11	Lipt2
1.307733431	1.18E-15	8.84E-20	11	Cdk5rap2
1.428106782	1.87E-15	1.40E-19	11	Gprasp1
1.73860311	2.63E-15	1.97E-19	11	Itprip
1.787574599	5.02E-15	3.76E-19	11	Ankrd13b
1.189720782	1.61E-14	1.20E-18	11	Tbl2
1.493322046	1.76E-14	1.32E-18	11	Tgif2
1.756780348	1.88E-14	1.41E-18	11	Ankrd33b
1.837154047	2.26E-14	1.69E-18	11	Rrp9
1.277049851	2.64E-14	1.98E-18	11	Usp30
1.790582393	2.77E-14	2.07E-18	11	Timmdc1
2.829904748	3.37E-14	2.52E-18	11	Ramp3
2.149433913	4.44E-14	3.32E-18	11	Acot2
2.544298455	5.59E-14	4.19E-18	11	Timp1
2.007117043	6.22E-14	4.66E-18	11	Rhoj
1.195167262	7.19E-14	5.38E-18	11	Gm16894
1.250148882	1.00E-13	7.50E-18	11	Palm
1.81865899	1.82E-13	1.36E-17	11	Smpdl3b
1.113154799	2.12E-13	1.59E-17	11	Mtrr
0.949055372	2.29E-13	1.72E-17	11	Cul9
1.448475217	2.41E-13	1.80E-17	11	Ndufaf5
1.442179375	2.44E-13	1.83E-17	11	Dexi
2.448488452	3.08E-13	2.30E-17	11	Arap2
1.447279133	4.13E-13	3.09E-17	11	Tmem18
1.534651262	6.55E-13	4.91E-17	11	Pspc1
1.98844465	7.43E-13	5.56E-17	11	Cyp4f16
2.014800129	7.44E-13	5.57E-17	11	Csnk1g1
2.168288443	7.46E-13	5.59E-17	11	Dcstamp
1.519072703	7.74E-13	5.79E-17	11	Cdc42ep2
2.208294619	7.82E-13	5.86E-17	11	AU040320
1.967942826	8.58E-13	6.42E-17	11	Kdm5b
1.277626429	1.37E-12	1.02E-16	11	Zfp282
1.497793631	1.49E-12	1.12E-16	11	Cd209d
1.219063457	1.50E-12	1.13E-16	11	Pik3r2
1.399985071	1.83E-12	1.37E-16	11	Coq6
1.597493439	1.86E-12	1.39E-16	11	Agl
1.866388637	2.08E-12	1.56E-16	11	Cstf1
1.31479198	2.68E-12	2.01E-16	11	Ogfod3
1.596261223	3.19E-12	2.39E-16	11	Smyd5
1.389619631	3.52E-12	2.64E-16	11	Iffo1
2.057836156	3.74E-12	2.80E-16	11	Cmtm4
1.217375407	4.68E-12	3.50E-16	11	Cep85
1.711089442	4.89E-12	3.66E-16	11	Siglech

2.059250539	5.23E-12	3.92E-16	11	Clmp
1.909710994	5.68E-12	4.25E-16	11	Ubxn8
1.430089049	5.88E-12	4.40E-16	11	Zfp938
2.368100302	5.92E-12	4.44E-16	11	H2-Oa
1.145942349	8.35E-12	6.26E-16	11	Rps6ka2
1.737301026	8.69E-12	6.51E-16	11	St18
1.631693306	9.56E-12	7.16E-16	11	Abhd13
2.023955448	1.23E-11	9.24E-16	11	Avpi1
1.465341962	1.32E-11	9.85E-16	11	Aig1
1.160875271	1.47E-11	1.10E-15	11	Bag5
1.498608708	1.71E-11	1.28E-15	11	Ddx19b
1.065924576	1.74E-11	1.31E-15	11	Gapt
2.333121817	1.85E-11	1.39E-15	11	Pwwp2b
1.957108489	2.18E-11	1.63E-15	11	Fem1a
0.943172654	2.92E-11	2.18E-15	11	B3galnt2
1.789542647	3.79E-11	2.84E-15	11	Ms4a4c
1.232892113	4.60E-11	3.44E-15	11	Cfl2
1.140906521	5.61E-11	4.20E-15	11	Ssh3
1.155414788	6.25E-11	4.68E-15	11	Srr
0.66956206	8.03E-11	6.01E-15	11	Ggact
1.593853718	9.47E-11	7.09E-15	11	Armc6
1.446589815	9.91E-11	7.42E-15	11	Urgcp
1.315344559	2.21E-10	1.65E-14	11	Fam126b
1.767467775	2.78E-10	2.08E-14	11	Pla2g4a
1.605834909	3.09E-10	2.31E-14	11	2610034B18Rik
1.536519906	3.89E-10	2.91E-14	11	9030617O03Rik
1.581497574	4.45E-10	3.33E-14	11	Cdh1
1.501460287	4.50E-10	3.37E-14	11	Kifap3
2.163403613	5.33E-10	3.99E-14	11	Atl2
1.217993653	6.29E-10	4.71E-14	11	Ints3
1.907453549	7.39E-10	5.53E-14	11	Srf
1.218686413	7.97E-10	5.97E-14	11	Pop1
1.463667638	7.99E-10	5.99E-14	11	Ddx28
1.399168236	9.21E-10	6.90E-14	11	9430008C03Rik
1.577907185	1.05E-09	7.88E-14	11	Mtf1
1.774089578	1.09E-09	8.14E-14	11	Ttc39b
1.34527767	1.43E-09	1.07E-13	11	Il1rl1
1.114449964	1.50E-09	1.12E-13	11	Polr3a
1.200751116	1.65E-09	1.23E-13	11	Slx4
0.584442979	1.65E-09	1.23E-13	11	Jazf1
2.207820096	1.72E-09	1.29E-13	11	Cd209a
0.689040791	1.82E-09	1.36E-13	11	L3mbtl1
1.583830547	1.96E-09	1.47E-13	11	Arl13b
1.09173596	2.34E-09	1.75E-13	11	Dhx29
1.961838331	2.44E-09	1.83E-13	11	Dnajc4
0.734645501	2.46E-09	1.85E-13	11	Ccdc15
1.340291328	2.52E-09	1.89E-13	11	Lrrk2
1.747765477	2.53E-09	1.89E-13	11	1110012L19Rik
2.194088326	2.77E-09	2.08E-13	11	Itgb1bp1
1.348956866	2.78E-09	2.08E-13	11	Cep70
1.222430244	2.90E-09	2.17E-13	11	Hsf2
1.0531401	3.04E-09	2.28E-13	11	Fam151b
0.855398423	3.19E-09	2.39E-13	11	1810014B01Rik
0.923179393	3.20E-09	2.40E-13	11	Armcx6
1.834213061	3.86E-09	2.89E-13	11	Rnf185
1.611008826	4.32E-09	3.24E-13	11	Prmt7
1.175304298	4.54E-09	3.40E-13	11	Prkca
1.20713214	5.37E-09	4.02E-13	11	Kti12
2.38540532	5.43E-09	4.06E-13	11	Ifitm1
2.071627805	5.64E-09	4.22E-13	11	Arl6ip5
0.754011065	6.12E-09	4.58E-13	11	Ppp1r16b

0.948805395	6.14E-09	4.60E-13	11	Zbtb5
0.871189408	6.38E-09	4.78E-13	11	Dnajb2
1.779985662	6.42E-09	4.80E-13	11	Mrps6
1.459963966	6.94E-09	5.19E-13	11	Mad2l1bp
1.505110416	7.79E-09	5.84E-13	11	Diap3
1.514245698	8.20E-09	6.14E-13	11	Mroh1
1.837445266	8.22E-09	6.15E-13	11	Nudt3
1.727181076	8.70E-09	6.52E-13	11	Ripk3
1.140017204	1.06E-08	7.94E-13	11	Rundc1
1.843075657	1.11E-08	8.31E-13	11	Slc2a6
1.425408033	1.14E-08	8.53E-13	11	Lonp1
1.36925099	1.19E-08	8.92E-13	11	Fbxl15
1.280168588	1.19E-08	8.94E-13	11	Fam102a
1.227370403	1.22E-08	9.17E-13	11	Hmgn2
1.613363779	1.26E-08	9.44E-13	11	Rtca
1.324196016	1.26E-08	9.46E-13	11	Exosc10
1.151468123	1.32E-08	9.87E-13	11	Kit
0.522677231	1.43E-08	1.07E-12	11	Intu
1.76238038	1.49E-08	1.11E-12	11	Rhoh
1.39133284	1.60E-08	1.20E-12	11	Fance
1.543959983	1.65E-08	1.23E-12	11	Insig2
0.952705012	1.65E-08	1.24E-12	11	Nudcd1
0.743509739	2.50E-08	1.87E-12	11	2010320M18Rik
0.974072128	2.62E-08	1.96E-12	11	Galnt12
1.58138871	2.63E-08	1.97E-12	11	Chid1
0.730658954	2.69E-08	2.01E-12	11	Snn
1.081709481	2.91E-08	2.18E-12	11	Aldh18a1
1.195138923	3.12E-08	2.33E-12	11	BC033916
1.171028649	3.28E-08	2.45E-12	11	Tbc1d8
1.231361039	3.33E-08	2.50E-12	11	Nrp
1.343687332	3.76E-08	2.81E-12	11	4930453N24Rik
1.122149671	3.95E-08	2.96E-12	11	Slc35b4
1.417620811	4.43E-08	3.32E-12	11	Eva1b
1.109380848	5.51E-08	4.13E-12	11	5730455P16Rik
1.937212955	5.62E-08	4.21E-12	11	Mtap
1.614568589	5.64E-08	4.22E-12	11	Fxr2
1.713047545	6.21E-08	4.65E-12	11	Tmem223
1.940352864	6.69E-08	5.01E-12	11	Siae
1.446127982	6.75E-08	5.06E-12	11	Il1rap
0.945419005	6.76E-08	5.06E-12	11	Lrrc42
1.286293928	6.89E-08	5.16E-12	11	Parp10
1.144080226	7.05E-08	5.28E-12	11	Cdca3
1.322442987	7.07E-08	5.29E-12	11	Cyb561d2
1.189659124	7.50E-08	5.62E-12	11	Mybpc3
1.318233343	7.56E-08	5.66E-12	11	Nat10
1.154846358	7.93E-08	5.94E-12	11	Mrps10
1.299124944	8.13E-08	6.09E-12	11	Trem1
1.984440256	8.24E-08	6.17E-12	11	4931406C07Rik
2.247531474	8.42E-08	6.30E-12	11	Tnip3
1.284721699	8.74E-08	6.55E-12	11	Dcps
1.253352883	9.44E-08	7.07E-12	11	Gas8
1.610818499	9.83E-08	7.36E-12	11	Nhlrc3
1.036594467	9.98E-08	7.47E-12	11	Mrps11
1.442039327	1.13E-07	8.44E-12	11	Ccr7
2.071785823	1.19E-07	8.90E-12	11	Olfml3
1.63786678	1.24E-07	9.25E-12	11	Araf
1.172882217	1.32E-07	9.88E-12	11	Alkbh2
1.407481638	1.44E-07	1.08E-11	11	Ttc27
0.783946411	1.70E-07	1.27E-11	11	Sell
1.469371972	1.76E-07	1.31E-11	11	Tm9sf4
1.314727042	1.80E-07	1.35E-11	11	Akr7a5

1.027850647	2.12E-07	1.59E-11	11	Adcy9
1.336933348	2.21E-07	1.65E-11	11	Smcr8
1.277227803	2.42E-07	1.81E-11	11	Rgs12
1.269643776	2.61E-07	1.96E-11	11	B9d2
1.404144212	3.02E-07	2.26E-11	11	Procr
1.112078415	3.60E-07	2.69E-11	11	Ciita
1.033545021	3.90E-07	2.92E-11	11	Tcf7l2
0.592620361	4.28E-07	3.20E-11	11	Zkscan14
1.726232295	4.45E-07	3.33E-11	11	Hsd3b7
1.757399885	4.65E-07	3.48E-11	11	Scnm1
1.013027367	4.71E-07	3.53E-11	11	Btbd9
1.30697186	4.98E-07	3.73E-11	11	Tcf20
0.893498948	5.43E-07	4.07E-11	11	Cd1d1
1.291039948	5.84E-07	4.37E-11	11	1110032A03Rik
0.959545196	5.85E-07	4.38E-11	11	Myo1b
0.935726722	6.18E-07	4.63E-11	11	Nup210
0.869806965	6.75E-07	5.05E-11	11	Fastk
0.78635431	7.03E-07	5.26E-11	11	Pdcd1
1.550627232	7.66E-07	5.74E-11	11	B230219D22Rik
0.913480162	8.78E-07	6.57E-11	11	Cspg4
1.430125217	8.81E-07	6.60E-11	11	Stat5a
1.351473016	1.19E-06	8.90E-11	11	Nup62
1.423501155	1.22E-06	9.12E-11	11	Drosha
1.093214205	1.23E-06	9.18E-11	11	Trpm4
1.041527715	1.28E-06	9.62E-11	11	Kif16b
1.125444349	1.30E-06	9.75E-11	11	Prune2
0.812988732	1.46E-06	1.09E-10	11	Mtmr7
1.051612784	1.55E-06	1.16E-10	11	Slc25a20
1.357742921	1.60E-06	1.20E-10	11	Heatr1
1.477439634	1.68E-06	1.26E-10	11	Usp32
0.638333918	1.77E-06	1.32E-10	11	Oplah
0.839999038	2.00E-06	1.50E-10	11	Jak3
1.75799128	2.01E-06	1.50E-10	11	2310045N01Rik
1.524197027	2.02E-06	1.51E-10	11	Esco1
1.157650523	2.45E-06	1.84E-10	11	Haus2
0.864457529	2.62E-06	1.96E-10	11	Kpna6
1.06317755	2.72E-06	2.04E-10	11	Wdr82
0.804757917	2.81E-06	2.11E-10	11	Sh3bgrl2
1.112253128	2.89E-06	2.16E-10	11	Atat1
1.638800789	2.94E-06	2.20E-10	11	Mamdc2
0.83228479	3.24E-06	2.43E-10	11	Tacc2
2.752361247	3.38E-06	2.53E-10	11	Fem1b
1.302355757	3.58E-06	2.68E-10	11	Arhgap12
1.001349706	3.93E-06	2.94E-10	11	Rrnad1
1.858502419	4.09E-06	3.06E-10	11	Hilpda
1.050325903	4.99E-06	3.74E-10	11	Gbf1
1.360649508	5.01E-06	3.75E-10	11	Atg14
1.148487208	5.14E-06	3.85E-10	11	Eid3
1.274772309	5.19E-06	3.89E-10	11	Gtl3
1.328067394	5.42E-06	4.06E-10	11	Ipo13
1.544259007	5.82E-06	4.36E-10	11	Rmnd5b
0.868697456	6.11E-06	4.58E-10	11	AI846148
1.819487576	6.22E-06	4.66E-10	11	Timm50
1.652170357	6.44E-06	4.82E-10	11	Atad1
1.303678596	7.47E-06	5.59E-10	11	Tmc6
1.559389412	7.65E-06	5.73E-10	11	Gpr35
0.700035842	7.67E-06	5.74E-10	11	Gba2
1.980269939	7.86E-06	5.89E-10	11	Il1r2
1.662369238	7.87E-06	5.90E-10	11	Reep3
1.52209348	7.89E-06	5.91E-10	11	Mthfr
0.959308117	7.91E-06	5.93E-10	11	Ly9

1.500759722	7.94E-06	5.95E-10	11	Nprl2
1.027830981	9.07E-06	6.79E-10	11	Urod
0.706311602	9.51E-06	7.12E-10	11	Atr
1.152829166	1.00E-05	7.51E-10	11	Ogfod2
1.420757034	1.07E-05	8.00E-10	11	Tmem175
0.988786234	1.29E-05	9.65E-10	11	Snord65
1.032932075	1.31E-05	9.78E-10	11	Ccdc43
1.399220659	1.31E-05	9.78E-10	11	Tmem38b
1.138583677	1.33E-05	9.96E-10	11	Acadsb
1.290052533	1.33E-05	9.99E-10	11	Slc30a9
1.400635946	1.39E-05	1.04E-09	11	Pmpca
1.240508968	1.42E-05	1.06E-09	11	C1galt1
0.422170861	1.44E-05	1.08E-09	11	Slc4a8
1.476625098	1.95E-05	1.46E-09	11	Mark3
1.573293754	2.00E-05	1.50E-09	11	Mapk1ip1l
1.134354679	2.03E-05	1.52E-09	11	Gclc
1.210631753	2.06E-05	1.54E-09	11	Gtf3a
1.735440591	2.13E-05	1.59E-09	11	Wdr33
1.154658686	2.54E-05	1.90E-09	11	Hmgcr
1.456535665	2.67E-05	2.00E-09	11	Hsd17b10
0.704075476	2.68E-05	2.01E-09	11	Cox10
0.755113674	2.71E-05	2.03E-09	11	Slc39a14
1.522302995	2.79E-05	2.09E-09	11	Aco2
0.554842509	2.89E-05	2.17E-09	11	Zfp748
1.334715659	2.93E-05	2.19E-09	11	Asxl1
1.454482213	3.04E-05	2.27E-09	11	Def6
1.493805953	3.13E-05	2.35E-09	11	Mllt6
1.26912654	3.22E-05	2.41E-09	11	Igf2bp2
1.253391458	3.34E-05	2.50E-09	11	Srprb
0.983064604	3.43E-05	2.57E-09	11	Polr3d
1.23495671	3.46E-05	2.59E-09	11	Spdl1
1.044580571	4.18E-05	3.13E-09	11	Lphn2
1.190707159	4.19E-05	3.14E-09	11	Mnat1
0.901181444	4.37E-05	3.28E-09	11	Zfp787
0.785838447	4.53E-05	3.39E-09	11	Slc30a4
1.406126505	4.61E-05	3.45E-09	11	Apoa1bp
1.390651729	5.19E-05	3.89E-09	11	Atxn2l
1.20887582	5.36E-05	4.02E-09	11	Zfyve19
1.28243018	5.41E-05	4.05E-09	11	Wwp2
0.813878984	5.46E-05	4.09E-09	11	6330416G13Rik
0.64262788	5.48E-05	4.10E-09	11	Zfp952
1.400339579	5.93E-05	4.44E-09	11	Samsn1
0.863015931	6.38E-05	4.78E-09	11	Htra4
1.368959616	6.41E-05	4.80E-09	11	Gdap2
1.504196064	6.68E-05	5.00E-09	11	Dennd5a
1.497282816	6.75E-05	5.06E-09	11	Cul1
1.378128152	6.96E-05	5.21E-09	11	Lcmt2
1.017086064	7.12E-05	5.33E-09	11	Tbc1d14
1.357162639	7.53E-05	5.64E-09	11	Phtf1
1.392263163	7.62E-05	5.71E-09	11	Il1rn
1.01090456	7.76E-05	5.81E-09	11	Pstpip2
0.953318674	7.94E-05	5.95E-09	11	Wrn
0.764112161	7.99E-05	5.99E-09	11	Elk1
1.437329364	8.44E-05	6.32E-09	11	Ndrg1
0.930524101	8.50E-05	6.37E-09	11	Itpr1
2.03909356	8.81E-05	6.60E-09	11	Gfpt1
0.840534227	8.92E-05	6.68E-09	11	Zfp580
0.888121781	0.000108312	8.11E-09	11	Gnpda2
0.844303942	0.000108487	8.13E-09	11	Pias3
1.259571654	0.000110422	8.27E-09	11	Qdpr
1.167560652	0.000113415	8.49E-09	11	Fbxl3

1.284989551	0.00011763	8.81E-09	11	2310009A05Rik
1.429584238	0.000118312	8.86E-09	11	Cops5
1.312530544	0.000121907	9.13E-09	11	Kcnn4
1.53901857	0.000122384	9.17E-09	11	Cc2d1b
0.75505285	0.00012339	9.24E-09	11	Gm2027
1.427663071	0.000123847	9.28E-09	11	Babam1
1.188728226	0.000123991	9.29E-09	11	Tnfsf13b
1.285141955	0.00013323	9.98E-09	11	Gramd1a
1.04940116	0.000133465	1.00E-08	11	Exosc1
0.804298201	0.000142506	1.07E-08	11	Zbtb37
1.475641659	0.000146267	1.10E-08	11	Vps37a
1.085209683	0.000148028	1.11E-08	11	Tnni2
1.15411423	0.000151219	1.13E-08	11	Phldb1
1.457113277	0.00016054	1.20E-08	11	Atp6v0a2
1.126104975	0.000161918	1.21E-08	11	Msl1
1.152707184	0.000164372	1.23E-08	11	Ttyh2
1.090934142	0.000164533	1.23E-08	11	Atp10a
0.853248545	0.000172976	1.30E-08	11	Cep44
0.824611902	0.000178847	1.34E-08	11	Usp37
0.931400621	0.000194664	1.46E-08	11	Lrrc8a
0.846314259	0.000194776	1.46E-08	11	Fkbpl
0.764192738	0.000198842	1.49E-08	11	Prtg
1.29253886	0.000203406	1.52E-08	11	Fau
1.241179598	0.000204489	1.53E-08	11	Ppp1r13l
0.781133664	0.000213632	1.60E-08	11	Wdfy1
1.391459605	0.00021516	1.61E-08	11	Trim41
1.095521096	0.000219396	1.64E-08	11	Vps45
0.524580423	0.000219596	1.64E-08	11	Fyco1
0.863912545	0.000229645	1.72E-08	11	Clstn1
1.178120574	0.000237361	1.78E-08	11	Pex13
1.306488927	0.00023784	1.78E-08	11	Napsa
1.183073723	0.000238331	1.78E-08	11	Thoc5
1.013516621	0.000238855	1.79E-08	11	Blcap
1.402821605	0.000240356	1.80E-08	11	Clcn7
1.147065033	0.000246872	1.85E-08	11	Pitrm1
1.326476303	0.000254719	1.91E-08	11	Smarcal1
1.248618818	0.000269716	2.02E-08	11	Tbc1d19
0.855816411	0.000283564	2.12E-08	11	Trappc13
1.132144793	0.000307732	2.30E-08	11	Fmn1
1.133059723	0.000308441	2.31E-08	11	Mrps27
1.361856354	0.000321669	2.41E-08	11	Ifih1
1.074669094	0.000325853	2.44E-08	11	Ado
0.923068504	0.000331397	2.48E-08	11	Nol12
1.264697112	0.000333894	2.50E-08	11	Rtfdc1
1.091453797	0.000338271	2.53E-08	11	Cul2
0.723364976	0.000367466	2.75E-08	11	Pde12
1.021704412	0.000381067	2.85E-08	11	Abcf2
1.462008333	0.000383747	2.87E-08	11	Ptpn22
1.077561947	0.000396618	2.97E-08	11	Nrm
0.571388905	0.000416195	3.12E-08	11	Caap1
0.857293135	0.000432348	3.24E-08	11	Tmem62
1.396899775	0.000439396	3.29E-08	11	Tnpo3
1.250066901	0.000444532	3.33E-08	11	2310061I04Rik
1.114648084	0.00045247	3.39E-08	11	Bloc1s4
1.118726298	0.000456943	3.42E-08	11	Mrps34
0.604733279	0.000457395	3.43E-08	11	Gmeb2
1.121834617	0.000461189	3.45E-08	11	Hivep1
0.954077068	0.000469813	3.52E-08	11	Eif2b4
1.312501598	0.000477803	3.58E-08	11	Dhrs7b
0.728107165	0.000478442	3.58E-08	11	Pigh
0.913763563	0.000509505	3.82E-08	11	Xpot

1.235096397	0.000512027	3.83E-08	11	Emilin2
1.057581262	0.000559881	4.19E-08	11	Rtn1
1.215657957	0.000561479	4.21E-08	11	Acot7
0.652060167	0.000575525	4.31E-08	11	Sh2d3c
0.71507647	0.000627856	4.70E-08	11	Cyb561a3
1.071933798	0.000645636	4.84E-08	11	Lyar
0.839040724	0.000661073	4.95E-08	11	Brd3
0.749358722	0.000665414	4.98E-08	11	Mki67
1.223201434	0.000690092	5.17E-08	11	Decr1
1.092639729	0.000696334	5.22E-08	11	Pdlim1
1.21722051	0.000708523	5.31E-08	11	Dync1li1
1.14131186	0.000718415	5.38E-08	11	Tuba4a
1.383153109	0.000740475	5.55E-08	11	Pdf
1.253784223	0.00074297	5.56E-08	11	Bmyc
0.836203859	0.000743719	5.57E-08	11	Vps33b
0.674117041	0.000757066	5.67E-08	11	C2cd5
1.161599056	0.000758269	5.68E-08	11	Cd97
0.593929898	0.000782954	5.86E-08	11	Il18rap
0.939827713	0.000786407	5.89E-08	11	Rfwd2
0.630721783	0.000795325	5.96E-08	11	Ccdc66
0.992780417	0.000886208	6.64E-08	11	A530054K11Rik
1.197193117	0.000909484	6.81E-08	11	Snhg1
0.70322041	0.000937122	7.02E-08	11	Sfxn5
0.546082194	0.000977876	7.32E-08	11	1810030O07Rik
0.741947418	0.000978012	7.32E-08	11	Cxcl14
1.004066018	0.001013625	7.59E-08	11	Rnmt
1.285627459	0.001055149	7.90E-08	11	Leng1
1.299851521	0.001090789	8.17E-08	11	Fam134a
1.181475008	0.00115573	8.66E-08	11	Wdfy3
1.291352302	0.001249629	9.36E-08	11	Scmh1
0.901173582	0.001255011	9.40E-08	11	3110040N11Rik
0.757877108	0.001269188	9.51E-08	11	Zranb3
0.852874993	0.001281185	9.60E-08	11	Crot
1.590096218	0.001313346	9.84E-08	11	Cers6
1.036991278	0.001338425	1.00E-07	11	Coq10a
0.992929562	0.001395584	1.05E-07	11	Rnpepl1
1.105545684	0.001397868	1.05E-07	11	Derl1
1.198746114	0.001404124	1.05E-07	11	Tulp4
1.14330999	0.001426354	1.07E-07	11	Clec4e
1.356238673	0.001448135	1.08E-07	11	Sos2
1.214558289	0.001480411	1.11E-07	11	Nufip1
0.787360346	0.001491408	1.12E-07	11	Swt1
1.293460645	0.001521906	1.14E-07	11	Slc16a6
0.599563378	0.001532766	1.15E-07	11	Ubr7
0.888015317	0.001545599	1.16E-07	11	Comt
1.106355174	0.001595059	1.19E-07	11	Hook3
1.291691908	0.00162853	1.22E-07	11	Il1b
0.873934742	0.001634013	1.22E-07	11	Gpd2
1.16328622	0.001793785	1.34E-07	11	Tgm2
1.226858218	0.001825785	1.37E-07	11	Agpat5
0.751404516	0.001880304	1.41E-07	11	Dus3l
1.131783941	0.001895928	1.42E-07	11	Brpf1
0.930552698	0.00192333	1.44E-07	11	Mettl5
1.162538669	0.001951547	1.46E-07	11	Gch1
1.120209886	0.00201481	1.51E-07	11	Ndufa11
0.972618182	0.002049505	1.53E-07	11	Tap1
1.023227196	0.002110753	1.58E-07	11	Atp13a1
0.931806823	0.002119021	1.59E-07	11	Tlr2
0.738498289	0.002120511	1.59E-07	11	Coasy
1.089144166	0.002127508	1.59E-07	11	Utp15
1.088861812	0.002178495	1.63E-07	11	Nop58

0.750249306	0.002195633	1.64E-07	11	Wdr7
1.132431349	0.002198811	1.65E-07	11	Fam198b
0.942076756	0.002231206	1.67E-07	11	Ercc3
0.941018118	0.002242796	1.68E-07	11	Mcph1
1.108756929	0.002271741	1.70E-07	11	Tnip1
0.741679098	0.002297211	1.72E-07	11	Flrt3
1.307307296	0.002416941	1.81E-07	11	Angel2
0.846611352	0.002427373	1.82E-07	11	Setd5
1.175282947	0.002441869	1.83E-07	11	Ifi35
0.880097092	0.002478868	1.86E-07	11	Trmt1
0.933708022	0.002520498	1.89E-07	11	Cpsf7
0.994479938	0.002543954	1.91E-07	11	Nudt14
1.961964285	0.002650472	1.99E-07	11	Gapvd1
1.046198932	0.00266502	2.00E-07	11	Col14a1
0.804907718	0.002682313	2.01E-07	11	Ccdc58
1.170886209	0.002730635	2.05E-07	11	Cask
1.022540875	0.002737801	2.05E-07	11	Ncoa1
1.028763823	0.002777015	2.08E-07	11	Plrg1
1.019687749	0.002832235	2.12E-07	11	Cox7a1
1.075699446	0.002911677	2.18E-07	11	Qtrt1
0.69481435	0.003012963	2.26E-07	11	Cmtr1
1.224464899	0.003144096	2.35E-07	11	Zyg11b
1.018414362	0.003151008	2.36E-07	11	Drg1
0.955926204	0.003242766	2.43E-07	11	Slamf9
1.220584625	0.003247158	2.43E-07	11	Mgl2
0.956616521	0.003268884	2.45E-07	11	Klhl7
0.750084384	0.003291039	2.46E-07	11	Taf1a
1.001297637	0.003383676	2.53E-07	11	Vma21
1.129466188	0.003409851	2.55E-07	11	Ttc14
1.322983437	0.003459889	2.59E-07	11	Milr1
0.582623218	0.003495927	2.62E-07	11	B630005N14Rik
0.5447312	0.003517628	2.63E-07	11	Tspan33
1.005847721	0.003633881	2.72E-07	11	Nsmce1
1.220954936	0.003638509	2.73E-07	11	Pcp4l1
0.78401452	0.003775299	2.83E-07	11	Tpra1
1.010792272	0.003945058	2.95E-07	11	Chchd1
1.201671715	0.004242722	3.18E-07	11	Ccdc101
0.652363464	0.004342991	3.25E-07	11	Unc119
0.982765117	0.004421846	3.31E-07	11	Mbnl2
1.221812683	0.004456863	3.34E-07	11	Ptpmt1
1.027280472	0.004585755	3.43E-07	11	Mrps7
0.983245153	0.004620587	3.46E-07	11	Tmem167
1.457713058	0.004667199	3.50E-07	11	Svil
0.953516215	0.00473246	3.54E-07	11	Strip1
0.917088498	0.004740384	3.55E-07	11	Rnf126
1.074988673	0.004779982	3.58E-07	11	Tmem135
0.953488813	0.004807736	3.60E-07	11	Ptafr
1.144384813	0.004817977	3.61E-07	11	Pqlc1
1.222805847	0.004914576	3.68E-07	11	Elf4
0.91079932	0.004953513	3.71E-07	11	Slc35c2
0.885921906	0.005024842	3.76E-07	11	Zfp335
1.156671635	0.005172269	3.87E-07	11	Etv3
0.798922892	0.005336843	4.00E-07	11	Sec24d
0.903584401	0.005467375	4.09E-07	11	Ripk2
0.814708247	0.005549406	4.16E-07	11	Zfp451
0.768761497	0.00559539	4.19E-07	11	Lyrm4
0.94784178	0.00562859	4.22E-07	11	Ndufaf1
0.900706537	0.005662893	4.24E-07	11	Tnpo2
1.242654155	0.005684437	4.26E-07	11	Pogk
0.790466722	0.005701991	4.27E-07	11	Dyrk3
0.996397088	0.005852217	4.38E-07	11	Pdrg1

0.87384438	0.006128157	4.59E-07	11	Cbfa2t3
0.884326401	0.00615964	4.61E-07	11	Wdr43
0.397364039	0.006304667	4.72E-07	11	Recql
0.819778706	0.006542538	4.90E-07	11	Slc7a2
1.342300369	0.006545984	4.90E-07	11	Acvr1
0.752554623	0.006729341	5.04E-07	11	2810008D09Rik
1.074692622	0.006936589	5.20E-07	11	Cnot1
0.829279355	0.006967059	5.22E-07	11	Vcpkmt
0.602917482	0.007052117	5.28E-07	11	Tfrc
1.011575729	0.007230987	5.42E-07	11	Fubp1
0.868564398	0.007263142	5.44E-07	11	Foxn3
0.673518801	0.007540645	5.65E-07	11	Kat2b
0.900254261	0.00763203	5.72E-07	11	0610010F05Rik
1.086541756	0.00767047	5.74E-07	11	Prpf3
0.909326872	0.007728202	5.79E-07	11	Iqcb1
1.06372783	0.007809488	5.85E-07	11	Mrps21
1.219954988	0.00785778	5.89E-07	11	Desi2
0.97404929	0.00797962	5.98E-07	11	Apbb2
0.965939887	0.008207688	6.15E-07	11	Atp2b4
1.112382769	0.008266895	6.19E-07	11	Clec4n
0.674454139	0.008317933	6.23E-07	11	Slc38a7
0.93497894	0.008559991	6.41E-07	11	Mgrn1
2.397462778	0.008721241	6.53E-07	11	Fgfr1op
0.666562233	0.008914777	6.68E-07	11	Pard6a
1.161166602	0.008967753	6.72E-07	11	Arhgap1
1.117369507	0.009174214	6.87E-07	11	Ormdl2
0.952058903	0.009488043	7.11E-07	11	N4bp1
0.9343602	0.009663247	7.24E-07	11	Dstn
0.936616131	0.009724575	7.28E-07	11	Cd109
0.938792373	0.009932672	7.44E-07	11	Sec63
0.97688319	0.010012606	7.50E-07	11	Swap70
0.852630116	0.010045043	7.52E-07	11	Hmces
0.798357246	0.010089241	7.56E-07	11	Guca1a
0.98945093	0.010322669	7.73E-07	11	Atp8b4
0.971832103	0.010494832	7.86E-07	11	St6gal1
1.009497861	0.010627123	7.96E-07	11	Abhd12
0.865107237	0.010751053	8.05E-07	11	Srxn1
0.483344203	0.010807094	8.09E-07	11	Rad50
0.849226966	0.011092808	8.31E-07	11	Ccnk
0.951061451	0.011107091	8.32E-07	11	Ccr2
0.764535263	0.011109531	8.32E-07	11	Zfp53
0.86987304	0.011211225	8.40E-07	11	Osbpl8
0.886451971	0.011256255	8.43E-07	11	Chst12
0.846541965	0.011391447	8.53E-07	11	Tmed9
0.688373902	0.01149375	8.61E-07	11	Gphn
0.8948649	0.011709973	8.77E-07	11	Atg13
0.402121422	0.011842544	8.87E-07	11	Dag1
0.909314	0.012015689	9.00E-07	11	Pxdc1
0.778813609	0.012308986	9.22E-07	11	Traf7
0.889090417	0.012329924	9.23E-07	11	6330407A03Rik
0.89412571	0.012332803	9.24E-07	11	1810011H11Rik
0.712246338	0.012517721	9.38E-07	11	Nom1
1.051077487	0.013815383	1.03E-06	11	Ifngr2
1.079859921	0.013975497	1.05E-06	11	Ralgds
0.651535087	0.014359593	1.08E-06	11	Ell
1.29336431	0.014414107	1.08E-06	11	Nes
0.922142809	0.014485139	1.08E-06	11	Fgd3
0.785699109	0.014498852	1.09E-06	11	AW112010
0.475782281	0.01450817	1.09E-06	11	Mthfd1
1.007284358	0.014782866	1.11E-06	11	Tcn2
1.023760308	0.015235517	1.14E-06	11	Acot8

1.085011096	0.015972926	1.20E-06	11	Slc31a2
1.069990077	0.016832128	1.26E-06	11	Anapc16
1.142540535	0.017012413	1.27E-06	11	Mvp
0.981367835	0.017174522	1.29E-06	11	Vimp
0.71960485	0.017539749	1.31E-06	11	Glce
0.962710502	0.017601168	1.32E-06	11	1110038B12Rik
1.08487034	0.01873212	1.40E-06	11	Neurl2
1.013710238	0.019009531	1.42E-06	11	Plbd1
0.818191682	0.019083868	1.43E-06	11	Mex3c
0.976807988	0.019177742	1.44E-06	11	Actr1a
0.926809941	0.019297235	1.45E-06	11	Serpine1
0.975888624	0.019520374	1.46E-06	11	Elof1
0.937981393	0.019748717	1.48E-06	11	Prpf8
0.963488346	0.020199647	1.51E-06	11	Ifi27
1.185637291	0.020236985	1.52E-06	11	Chchd4
0.862066132	0.02056572	1.54E-06	11	Mtx1
0.772359558	0.02100236	1.57E-06	11	Dgka
0.506891271	0.022815218	1.71E-06	11	Bcr
0.643161559	0.023186581	1.74E-06	11	Orc6
1.050584722	0.023445216	1.76E-06	11	Cflar
0.60343422	0.023760747	1.78E-06	11	Nsmce4a
0.949761331	0.024742106	1.85E-06	11	Ramp1
0.765317552	0.024850485	1.86E-06	11	Tsc22d2
1.020364966	0.025286259	1.89E-06	11	Clec5a
0.504310943	0.025631845	1.92E-06	11	C920009B18Rik
0.846270745	0.025861298	1.94E-06	11	Ehd1
0.72855011	0.026052638	1.95E-06	11	Chtop
0.359785628	0.026211043	1.96E-06	11	Insr
0.983787225	0.026462785	1.98E-06	11	Cirh1a
0.625449586	0.026562056	1.99E-06	11	Kmo
0.49908541	0.027145077	2.03E-06	11	Nifk
0.727259787	0.027746622	2.08E-06	11	Fam120aos
1.277985487	0.027987095	2.10E-06	11	Ubr3
0.917866681	0.02813367	2.11E-06	11	Mettl17
1.097482001	0.028571416	2.14E-06	11	Pilrb1
0.884551005	0.028654825	2.15E-06	11	Itfg1
0.873695214	0.029821242	2.23E-06	11	Tbc1d9b
0.916458303	0.030194917	2.26E-06	11	Mcts1
0.908729023	0.030399387	2.28E-06	11	Arfrp1
0.429863207	0.030562928	2.29E-06	11	Dync2h1
0.504546887	0.030844648	2.31E-06	11	Serpinb6b
1.143354074	0.031077316	2.33E-06	11	Rchy1
0.930513954	0.031188238	2.34E-06	11	Odc1
1.090585051	0.03237885	2.43E-06	11	Bmi1
0.874286225	0.033630741	2.52E-06	11	Ndufb7
0.950309534	0.034458242	2.58E-06	11	Csf2rb2
1.12551181	0.035600768	2.67E-06	11	Yipf3
0.84989986	0.036694181	2.75E-06	11	Tubgcp3
0.816817588	0.036841903	2.76E-06	11	Adnp
0.434377887	0.037784876	2.83E-06	11	Zfp74
2.394046514	0.039642057	2.97E-06	11	Rapgef2
0.770438855	0.040953311	3.07E-06	11	Lrrc28
1.003944006	0.041106877	3.08E-06	11	Aim1
0.861735717	0.041122698	3.08E-06	11	Stap1
1.223520679	0.041684029	3.12E-06	11	Znrd1
0.76606248	0.042041684	3.15E-06	11	Pttg1
0.804967169	0.042394044	3.18E-06	11	Mrpl34
0.851711956	0.042572327	3.19E-06	11	Sde2
0.488519866	0.042893848	3.21E-06	11	Klhl25
0.821905294	0.042951082	3.22E-06	11	D10Jhu81e
0.742070821	0.043620624	3.27E-06	11	Arhgap11a

0.642388789	0.044260094	3.31E-06	11	Zswim8
0.903570391	0.044543587	3.34E-06	11	Nfkb1
0.272366271	0.045067626	3.38E-06	11	4831440E17Rik
0.699966258	0.045386591	3.40E-06	11	Rnf146
1.125168053	0.045431858	3.40E-06	11	Arhgef10l
0.894893396	0.046579127	3.49E-06	11	Myo7a
0.674650321	0.048287537	3.62E-06	11	Dis3l
0.632454508	0.049064936	3.67E-06	11	Timm17b
1.076049468	0.049505253	3.71E-06	11	Tmem263
0.794754305	0.049745408	3.73E-06	11	Prpf38a
0.768861153	0.049929102	3.74E-06	11	Adam15
0.788979733	0.050734519	3.80E-06	11	Mdm2
0.697601531	0.050796062	3.80E-06	11	Csnk1g3
1.288002767	0.05082152	3.81E-06	11	Mrpl43
0.813137769	0.050958051	3.82E-06	11	Farsa
0.761904767	0.051848555	3.88E-06	11	Fam117b
0.47545042	0.052700191	3.95E-06	11	Vbp1
0.783225171	0.053015954	3.97E-06	11	Gpr171
1.077664788	0.053087494	3.98E-06	11	Timm8b
0.517124426	0.053333757	3.99E-06	11	Capn7
1.198433535	0.053408721	4.00E-06	11	Kidins220
0.880235324	0.053489862	4.01E-06	11	Denr
0.921086904	0.054259511	4.06E-06	11	Surf1
0.496180597	0.054334559	4.07E-06	11	2410004N09Rik
0.50817099	0.055164579	4.13E-06	11	Cdca4
1.163163112	0.056629078	4.24E-06	11	Fcrls
1.13150507	0.056681589	4.25E-06	11	Gtf2h2
0.698526273	0.056998158	4.27E-06	11	Upf2
0.860235548	0.057205355	4.28E-06	11	Bcap31
1.030437499	0.058042593	4.35E-06	11	Sumf1
1.413244048	0.059172538	4.43E-06	11	Pdcd6
0.713881661	0.059341684	4.44E-06	11	Coq3
0.952867013	0.059491195	4.46E-06	11	Pon3
0.853571126	0.060039922	4.50E-06	11	Rela
0.915812377	0.060557915	4.54E-06	11	Dnmt3a
1.261114113	0.061077426	4.57E-06	11	Slk
0.924383032	0.06124006	4.59E-06	11	Elp5
0.684127611	0.061419677	4.60E-06	11	Rusc2
1.082444713	0.061494576	4.61E-06	11	Sntb2
0.83390685	0.063053248	4.72E-06	11	Uty
0.788568928	0.063523975	4.76E-06	11	Psmb4
0.598555224	0.064001682	4.79E-06	11	Bin3
0.763187251	0.064002917	4.79E-06	11	Sh2b3
0.620443083	0.064479229	4.83E-06	11	Nt5c2
0.290012152	0.065667189	4.92E-06	11	B3gntl1
0.751447407	0.065836261	4.93E-06	11	Arl6ip6
0.552554626	0.065849438	4.93E-06	11	Fkbp4
0.569617795	0.066059438	4.95E-06	11	Daam1
1.169420908	0.067673711	5.07E-06	11	Stk16
0.588025928	0.06838339	5.12E-06	11	Bdp1
-1.258862928	0.069302714	5.19E-06	11	Rps26
0.89235616	0.069481726	5.20E-06	11	Nab1
0.829541132	0.07012915	5.25E-06	11	Tnfaip2
0.956222948	0.07157595	5.36E-06	11	Plod1
1.026594793	0.071812462	5.38E-06	11	Brms1
0.820510135	0.072084629	5.40E-06	11	Edem1
0.932285694	0.072298898	5.41E-06	11	Rftn1
1.368215844	0.07246767	5.43E-06	11	Tnfsf9
1.223093846	0.073641028	5.52E-06	11	Rpgrip1
1.689344944	0.074539914	5.58E-06	11	Hipk1
1.022615566	0.075258974	5.64E-06	11	Dhx57

1.016877969	0.076857215	5.76E-06	11	Tmem120a
0.751070845	0.076995083	5.77E-06	11	Zbtb2
0.637680787	0.077163294	5.78E-06	11	Dhrs9
0.530321071	0.077665449	5.82E-06	11	Smg6
0.735320565	0.077804402	5.83E-06	11	Ktn1
0.936265623	0.077818961	5.83E-06	11	Osbpl9
0.90744615	0.077837614	5.83E-06	11	Ndufs8
0.251608914	0.079680244	5.97E-06	11	Agmo
0.988547462	0.079744853	5.97E-06	11	Cav2
-1.520328922	0.079938298	5.99E-06	11	Jund
0.8525089	0.080296555	6.01E-06	11	Hnrnpf
0.999739276	0.081080745	6.07E-06	11	Ccl2
1.104964499	0.081563535	6.11E-06	11	Creb5
0.934855735	0.082778765	6.20E-06	11	Secisbp2l
0.752541538	0.082884513	6.21E-06	11	Stx3
0.973112017	0.082952513	6.21E-06	11	Xpr1
1.07941835	0.084351918	6.32E-06	11	Kif3a
0.927876282	0.085447036	6.40E-06	11	Nploc4
0.469582665	0.086189617	6.46E-06	11	Cish
0.469296577	0.086837331	6.50E-06	11	Rqcd1
1.129170599	0.087310763	6.54E-06	11	Ell2
0.954822687	0.088388009	6.62E-06	11	Usp16
0.929565523	0.088637984	6.64E-06	11	Ccdc97
0.876893489	0.089575403	6.71E-06	11	Dnajc14
0.784362278	0.090310704	6.76E-06	11	Atp1b3
0.668345922	0.092031643	6.89E-06	11	Mphosph10
0.757022598	0.092121821	6.90E-06	11	Mrpl28
1.079981975	0.092194348	6.90E-06	11	Cdk18
0.755364456	0.092537435	6.93E-06	11	Gusb
0.593544838	0.093782095	7.02E-06	11	Swi5
0.625597496	0.094435323	7.07E-06	11	Zfp90
0.718934186	0.097468601	7.30E-06	11	Chd7
0.935736457	0.098264693	7.36E-06	11	Eif1ax
0.761043248	0.098402105	7.37E-06	11	Vps16
0.875678266	0.098913905	7.41E-06	11	Bre
0.688808779	0.099639151	7.46E-06	11	Hes1
0.574804745	0.100391054	7.52E-06	11	Fam120b
0.836925471	0.100790321	7.55E-06	11	Cyc1
1.150446887	0.101900837	7.63E-06	11	Ift52
0.417190829	0.103467996	7.75E-06	11	Iqce
0.96778377	0.104569807	7.83E-06	11	Pigyl
0.764535263	0.104895897	7.86E-06	11	Parvb
0.960829493	0.105146084	7.87E-06	11	Surf2
1.065619508	0.105347607	7.89E-06	11	Polr1d
1.67240177	0.10623687	7.96E-06	11	Pdgfa
0.549834987	0.106935272	8.01E-06	11	Aldh16a1
0.842382936	0.108167503	8.10E-06	11	Pdpn
0.738375326	0.109679977	8.21E-06	11	Nubp2
0.707451612	0.109694876	8.22E-06	11	Siah2
1.88469176	0.110782754	8.30E-06	11	Nktr
0.913237144	0.110989328	8.31E-06	11	Anapc2
0.973691806	0.111427392	8.35E-06	11	Gm11974
0.475134306	0.111827922	8.38E-06	11	Clec2d
0.667435583	0.113417779	8.49E-06	11	Tspo
0.610641553	0.113531775	8.50E-06	11	Chchd7
0.385256021	0.11568735	8.66E-06	11	Uimc1
0.77798606	0.116008896	8.69E-06	11	Tomm40
0.690598597	0.11910715	8.92E-06	11	Ubr4
0.680544838	0.119663503	8.96E-06	11	Slfn2
0.853248545	0.119940933	8.98E-06	11	Bcl2l1
0.802140623	0.124136726	9.30E-06	11	Ten1

0.665429278	0.126863125	9.50E-06	11	Tmem134
0.68043232	0.131245101	9.83E-06	11	Trpc4ap
0.651294743	0.132578438	9.93E-06	11	Myeov2
0.955137452	0.132745356	9.94E-06	11	Tmem106a
0.879649714	0.132882727	9.95E-06	11	Edc3
0.679348091	0.133807717	1.00E-05	11	Tbc1d4
0.85074334	0.134624726	1.01E-05	11	Ddx21
0.758081314	0.134808448	1.01E-05	11	Ddit4
0.787351816	0.135776676	1.02E-05	11	Ddx1
1.014047511	0.138488105	1.04E-05	11	Smad4
0.72697614	0.138809359	1.04E-05	11	Gpr126
0.616544947	0.143186929	1.07E-05	11	Zfyve1
0.696809381	0.143499625	1.07E-05	11	Ube3a
0.657700249	0.143737642	1.08E-05	11	Ppp2r2a
0.533081017	0.145859672	1.09E-05	11	Ube2v1
0.818003143	0.147115697	1.10E-05	11	Cep350
0.598403943	0.147514482	1.10E-05	11	Xrcc5
-1.047823555	0.147828148	1.11E-05	11	Calr
0.892286091	0.151384298	1.13E-05	11	Cpsf3l
0.806871478	0.153149071	1.15E-05	11	BC029214
0.678260202	0.15352767	1.15E-05	11	1700020I14Rik
0.646522301	0.154251744	1.16E-05	11	Brap
0.788106545	0.155272562	1.16E-05	11	Atraid
0.690041959	0.156529878	1.17E-05	11	Stx12
0.638180202	0.157577339	1.18E-05	11	Mak16
0.984828721	0.158460227	1.19E-05	11	Tspan4
0.937672559	0.161575174	1.21E-05	11	Emb
0.824846636	0.165925288	1.24E-05	11	Capn2
0.624423789	0.167615968	1.26E-05	11	Phlpp1
0.670021642	0.170184189	1.27E-05	11	Basp1
1.005091652	0.172362134	1.29E-05	11	Pbxip1
0.849151429	0.175476578	1.31E-05	11	Bscl2
0.709043572	0.177920862	1.33E-05	11	Gpbp1
1.036446212	0.178071368	1.33E-05	11	Slc31a1
0.82526234	0.178409281	1.34E-05	11	Dnajb1
0.840304825	0.178850154	1.34E-05	11	Stag1
1.053185165	0.18033396	1.35E-05	11	Baiap2
0.822383774	0.181400719	1.36E-05	11	Cux1
1.000399738	0.182148257	1.36E-05	11	Golph3l
0.328978025	0.182661287	1.37E-05	11	Gmcl1
0.717477387	0.183263402	1.37E-05	11	S100a4
0.665104285	0.187714315	1.41E-05	11	Nedd8
0.811260849	0.193733237	1.45E-05	11	Dtd2
0.594206228	0.194862256	1.46E-05	11	Rpl27a
0.858707896	0.195062071	1.46E-05	11	Jak2
0.829350674	0.197406369	1.48E-05	11	Tnfrsf12a
0.989785008	0.200704627	1.50E-05	11	Sh3kbp1
1.198274028	0.203712457	1.53E-05	11	Rap2a
0.964489142	0.212849152	1.59E-05	11	Snx20
0.626761736	0.216615501	1.62E-05	11	Arsk
0.809189701	0.221187963	1.66E-05	11	Smarcb1
0.7137217	0.223832558	1.68E-05	11	Dnajc5
0.495668731	0.224565081	1.68E-05	11	Man2c1
0.608477725	0.224656781	1.68E-05	11	Pet112
0.7887209	0.227200206	1.70E-05	11	Pmp22
0.714698321	0.231414787	1.73E-05	11	Chd3
1.963969503	0.231917607	1.74E-05	11	Rnf6
0.542477748	0.23196303	1.74E-05	11	Lbr
0.588137604	0.236497508	1.77E-05	11	Metap1d
1.262257662	0.2453487	1.84E-05	11	Tppp3
0.642883576	0.252014367	1.89E-05	11	Csf2ra

1.049184369	0.258011668	1.93E-05	11	Etfdh
0.708095724	0.260513466	1.95E-05	11	Rxrb
0.603838487	0.263135872	1.97E-05	11	Nlrp3
0.419040968	0.263281218	1.97E-05	11	Ino80b
0.934766285	0.265518255	1.99E-05	11	Mfsd5
1.183451169	0.267475531	2.00E-05	11	Slc38a1
1.030193745	0.276517263	2.07E-05	11	Pank3
0.629239176	0.27698329	2.07E-05	11	Mgst3
0.373076662	0.281095694	2.11E-05	11	Inpp1
0.919436769	0.282139564	2.11E-05	11	Pak1ip1
0.637204748	0.28806042	2.16E-05	11	Flna
0.536051107	0.295649109	2.21E-05	11	Ptgr1
0.423045075	0.299364491	2.24E-05	11	F11r
0.833334519	0.302171926	2.26E-05	11	Snrnp200
0.656833559	0.304017073	2.28E-05	11	Smim12
0.518596824	0.304045492	2.28E-05	11	Ccny
1.192139162	0.307962989	2.31E-05	11	Nop9
0.73218912	0.308975849	2.31E-05	11	Emc2
0.689635959	0.314155141	2.35E-05	11	Atf6
0.817668728	0.320104036	2.40E-05	11	C1qbp
0.770128436	0.320205495	2.40E-05	11	Ostm1
0.498989225	0.322041921	2.41E-05	11	P2ry10
0.596360491	0.342212181	2.56E-05	11	Jmjd1c
0.719647695	0.344705416	2.58E-05	11	0610012G03Rik
0.944278382	0.346363208	2.59E-05	11	Cct3
0.733074081	0.350974716	2.63E-05	11	Rel
0.79037307	0.353199492	2.65E-05	11	Specc1
0.811849736	0.365986619	2.74E-05	11	Clec4d
0.636782917	0.371588102	2.78E-05	11	Arhgef1
0.749119304	0.373502385	2.80E-05	11	Il4ra
0.637997694	0.373533525	2.80E-05	11	Rab32
0.729933632	0.376168562	2.82E-05	11	Hccs
0.504726524	0.378707689	2.84E-05	11	1500011K16Rik
0.592469721	0.382926302	2.87E-05	11	Rab24
0.530671585	0.387825718	2.90E-05	11	Pgap2
0.619697036	0.388623591	2.91E-05	11	Plbd2
0.749591483	0.393469149	2.95E-05	11	Ptpn2
0.519402715	0.394502601	2.95E-05	11	Gpn1
1.102662873	0.395479145	2.96E-05	11	Mcmbp
0.778764476	0.395990737	2.97E-05	11	Gpr137b-ps
0.529224746	0.396447303	2.97E-05	11	Slc4a1ap
0.623641601	0.397318575	2.98E-05	11	Hmgn5
0.779765165	0.397581214	2.98E-05	11	Cytip
0.384667068	0.400876977	3.00E-05	11	Pias4
0.501958173	0.402425162	3.01E-05	11	2810013P06Rik
0.462301311	0.404281525	3.03E-05	11	Trip4
0.587995992	0.407525028	3.05E-05	11	Acly
0.555133814	0.408155374	3.06E-05	11	Dpp9
0.968136347	0.409009433	3.06E-05	11	Scyl2
0.831374197	0.419915223	3.14E-05	11	Acsl1
0.813746642	0.423336419	3.17E-05	11	Pgk1
0.737627942	0.42501506	3.18E-05	11	Rilpl2
-1.377232537	0.425293612	3.19E-05	11	Gm1821
1.061855964	0.434437533	3.25E-05	11	Fdx1
0.592131755	0.436916184	3.27E-05	11	Hmbs
0.726276186	0.4382952	3.28E-05	11	Fam110a
0.457091518	0.438741559	3.29E-05	11	Pank2
0.896061195	0.442859302	3.32E-05	11	Pqlc2
0.724931498	0.450205092	3.37E-05	11	Map3k11
0.780919005	0.455082507	3.41E-05	11	Arid4b
0.717154488	0.458484178	3.43E-05	11	Synj1

0.614833467	0.472939296	3.54E-05	11	Zfp568
0.616034561	0.473882375	3.55E-05	11	Jarid2
-1.983450771	0.475964469	3.56E-05	11	Pirb
0.796443784	0.482450645	3.61E-05	11	Pmepa1
0.51956036	0.490323229	3.67E-05	11	Dcun1d2
0.426687177	0.492258704	3.69E-05	11	Kctd10
0.350579788	0.497744378	3.73E-05	11	Csgalnact2
0.753513204	0.5014079	3.76E-05	11	Papss1
0.694652072	0.510995357	3.83E-05	11	Rsbn1l
0.834487444	0.521409182	3.91E-05	11	Ckb
0.761215708	0.524418945	3.93E-05	11	Cldn25
0.790301164	0.527561909	3.95E-05	11	Pias2
0.640951838	0.528499137	3.96E-05	11	Nol7
0.638811835	0.531578759	3.98E-05	11	Srsf10
0.481787723	0.541660183	4.06E-05	11	Cd59a
0.759854337	0.542591216	4.06E-05	11	2900026A02Rik
1.145890068	0.546379757	4.09E-05	11	Etf1
-1.441328473	0.557328096	4.17E-05	11	Sepp1
0.78811178	0.558602246	4.18E-05	11	Map4k4
-1.822480439	0.561742191	4.21E-05	11	Gnb1
0.35807322	0.577168622	4.32E-05	11	Nsun2
0.34077162	0.577759766	4.33E-05	11	Wbp1l
0.532957614	0.578261952	4.33E-05	11	Smarca2
1.887449158	0.578282819	4.33E-05	11	Rest
0.52875953	0.578311933	4.33E-05	11	Aak1
0.349140504	0.580590296	4.35E-05	11	Smpd1
0.611661462	0.580969622	4.35E-05	11	Chchd3
0.618763519	0.581765959	4.36E-05	11	Zfp800
0.55627546	0.583551137	4.37E-05	11	Raph1
0.723192902	0.589639245	4.42E-05	11	Tcirg1
0.700160261	0.590219021	4.42E-05	11	Yaf2
0.910042846	0.592071591	4.43E-05	11	Psmc4
0.978169317	0.592523834	4.44E-05	11	Tmem147
0.747022813	0.594150525	4.45E-05	11	Mmp12
0.760037002	0.597176352	4.47E-05	11	Pex11b
0.82401103	0.622784635	4.66E-05	11	Cct7
0.653613215	0.624726429	4.68E-05	11	Scaf8
0.750594391	0.628157572	4.70E-05	11	Mdp1
0.631622716	0.629465779	4.71E-05	11	Nars
0.408433056	0.634270279	4.75E-05	11	Lrrc58
-1.037986694	0.636140713	4.76E-05	11	Lars2
0.268550291	0.640634041	4.80E-05	11	Gdap10
0.545681475	0.642244897	4.81E-05	11	Celf2
0.6024001	0.650530824	4.87E-05	11	Appl1
0.815258056	0.651566379	4.88E-05	11	Cpd
0.767745767	0.661465421	4.95E-05	11	Cfh
0.421311117	0.671782727	5.03E-05	11	Cxcr3
0.768254653	0.674551337	5.05E-05	11	Dap
0.88486433	0.690594402	5.17E-05	11	Aamdc
0.629327334	0.727995504	5.45E-05	11	1110007C09Rik
0.600323638	0.731984833	5.48E-05	11	Sgta
0.702397703	0.738827734	5.53E-05	11	Rab10
0.702608062	0.742202378	5.56E-05	11	Cdc34
0.719496499	0.750888822	5.62E-05	11	Tmem140
0.458320942	0.755882966	5.66E-05	11	1600012H06Rik
0.601016332	0.759249681	5.69E-05	11	Inpp5d
0.718079056	0.759652948	5.69E-05	11	Atg16l1
0.434853662	0.768949578	5.76E-05	11	Zfp236
0.70617061	0.771023259	5.77E-05	11	Nufip2
0.694859535	0.777229926	5.82E-05	11	Zfp414
0.973931521	0.780671198	5.85E-05	11	Pde4b

0.783607346	0.783406437	5.87E-05	11	Grk6
0.705054759	0.796330751	5.96E-05	11	Tank
0.592890577	0.808527282	6.06E-05	11	1810043H04Rik
0.443868745	0.810471532	6.07E-05	11	Srek1
0.466184341	0.813392043	6.09E-05	11	Tnks2
0.508524534	0.819498938	6.14E-05	11	Pafah1b2
0.543093617	0.825679233	6.18E-05	11	A830080D01Rik
0.729401679	0.829864683	6.22E-05	11	Baz1a
1.020974995	0.831417841	6.23E-05	11	Cd83
0.77121218	0.832914779	6.24E-05	11	Kansl3
0.876249314	0.835747796	6.26E-05	11	A630007B06Rik
1.116981947	0.835930435	6.26E-05	11	Fn1
0.576990742	0.845597525	6.33E-05	11	Cd96
0.900379709	0.850404496	6.37E-05	11	Fam134b
0.669738303	0.853794754	6.39E-05	11	Fam195b
0.522416957	0.865646286	6.48E-05	11	Foxp1
0.761836679	0.870699545	6.52E-05	11	Gars
0.597066212	0.896475337	6.71E-05	11	Tra2a
0.620784484	0.903173484	6.76E-05	11	Emc3
0.550952237	0.909302609	6.81E-05	11	Eif4e
0.59181832	0.912223359	6.83E-05	11	D8Ertd738e
0.385303592	0.924721177	6.93E-05	11	Etv6
0.716028596	0.929674215	6.96E-05	11	Olfm1
0.712101463	0.952889285	7.14E-05	11	Cdr2
0.568156556	0.958021483	7.18E-05	11	Ctns
0.802657532	0.9598586	7.19E-05	11	Pdxk
0.666594457	0.960806387	7.20E-05	11	Cebpg
0.3459231	0.962804177	7.21E-05	11	Ppp3cc
0.786049044	0.964969488	7.23E-05	11	Smox
0.475096284	0.965933599	7.23E-05	11	Srgn
0.578280418	0.96701558	7.24E-05	11	Stat6
0.634792629	0.987366477	7.39E-05	11	Mmadhc
0.789159361	0.987913477	7.40E-05	11	Gas7
0.747000521	0.988177664	7.40E-05	11	Srd5a3
0.564387922	0.994360615	7.45E-05	11	Cyr61
0.557187599	0.998579944	7.48E-05	11	Nrbp1
0.369004109	1	7.50E-05	11	Acap1
0.731029077	1	7.64E-05	11	Slc35b1
0.460330096	1	7.70E-05	11	Brd9
0.945199394	1	7.76E-05	11	Rras
0.759423041	1	7.78E-05	11	Ppp3r1
0.633848785	1	7.86E-05	11	Ms4a6d
0.668616351	1	7.95E-05	11	Tep1
0.645727781	1	8.01E-05	11	Map2k3
0.639750836	1	8.07E-05	11	Commd9
0.714280366	1	8.14E-05	11	Ston2
0.535267011	1	8.18E-05	11	Tnf
0.846978932	1	8.23E-05	11	Acadl
0.667568585	1	8.57E-05	11	Ncf4
0.635748162	1	8.63E-05	11	Fam173a
0.787391386	1	8.64E-05	11	Larp4b
0.357534103	1	8.64E-05	11	Endog
0.684832778	1	8.73E-05	11	Ndufv2
0.690481384	1	9.02E-05	11	2810025M15Rik
1.091393024	1	9.03E-05	11	Klf7
0.551151413	1	9.21E-05	11	Nr2c2ap
0.77744273	1	9.29E-05	11	Elmod3
0.638330168	1	9.30E-05	11	Ctnna1
0.518151388	1	9.30E-05	11	Lrrc47
0.855891992	1	9.37E-05	11	Rit1
0.68789209	1	9.39E-05	11	Vopp1

0.531436411	1	9.51E-05	11	Trim11
0.669626016	1	9.57E-05	11	Esd
0.649943561	1	9.71E-05	11	Ndufs6
0.479791178	1	9.71E-05	11	Pitpna
0.651040338	1	9.95E-05	11	Ppp1r12c
-0.810885574	1	9.97E-05	11	Cfl1
0.71798447	1	0.0001015	11	Galnt6
0.777970964	1	0.0001015	11	Pstpip1
0.738925617	1	0.0001029	11	Cast
0.651587126	1	0.0001038	11	Ik
0.757255607	1	0.0001042	11	Nrp2
0.823375264	1	0.0001047	11	Adam8
0.914299096	1	0.0001057	11	Prdx4
0.662436403	1	0.0001063	11	Rps19bp1
0.717425729	1	0.0001065	11	Casp4
0.691465704	1	0.0001068	11	Adrbk1
0.407512422	1	0.0001074	11	Psme4
0.519278107	1	0.0001075	11	AI504432
0.821080577	1	0.0001078	11	Sec24b
0.815646263	1	0.0001096	11	0610009L18Rik
0.961150077	1	0.0001105	11	Fbxo32
0.768993536	1	0.000111	11	Cyhr1
0.826591583	1	0.0001149	11	Rwdd4a
0.536208908	1	0.0001169	11	Prkx
0.513926401	1	0.0001178	11	4930526I15Rik
0.454838419	1	0.0001188	11	Vps37b
0.61697491	1	0.0001194	11	Arhgef2
0.788384768	1	0.0001205	11	Ascc1
0.573231786	1	0.0001205	11	Tlr7
0.639010179	1	0.0001211	11	Ubxn4
0.437765493	1	0.0001213	11	Ccl5
0.698218116	1	0.0001217	11	Ap1g2
0.705601335	1	0.0001218	11	Ints1
0.690833063	1	0.0001233	11	Dgat1
0.493172682	1	0.0001237	11	Frrs1
0.271747211	1	0.0001244	11	Dtx4
0.51100285	1	0.0001254	11	Spast
0.633203171	1	0.0001263	11	Cd52
0.536984273	1	0.0001267	11	Ola1
0.768152291	1	0.0001318	11	Mlf2
0.300463103	1	0.0001324	11	Zranb2
0.427109143	1	0.0001327	11	Adamts10
0.318077862	1	0.0001329	11	Fundc1
0.475807302	1	0.0001337	11	Mettl2
0.669116085	1	0.0001339	11	Cisd3
0.537657684	1	0.0001348	11	Ankrd28
0.663101111	1	0.0001352	11	Hpcal1
0.579818495	1	0.0001361	11	Pim1
0.800362944	1	0.0001365	11	Pusl1
0.660317579	1	0.0001371	11	Tbcd
0.375024083	1	0.000139	11	Tmem87a
0.350171561	1	0.0001395	11	Ap2b1
0.360850961	1	0.0001396	11	Fam168a
0.527044085	1	0.0001432	11	Spns1
0.705318828	1	0.0001434	11	Wbp2
0.476717459	1	0.000145	11	Ccnd2
0.800453235	1	0.0001467	11	Btaf1
0.630353754	1	0.0001497	11	Ppm1g
0.473781752	1	0.0001504	11	Arih2
0.41000334	1	0.0001511	11	Hars2
0.542740595	1	0.0001512	11	Stat4

0.540453772	1	0.0001512	11	Senp3
0.589669636	1	0.0001517	11	Mapkap1
0.528811535	1	0.0001525	11	Lsp1
0.589990048	1	0.0001531	11	Sars
0.406618448	1	0.0001546	11	Lpcat3
0.493782252	1	0.0001557	11	Strap
0.983315376	1	0.0001564	11	Camk2g
0.574814083	1	0.0001579	11	Baz2b
0.377247869	1	0.0001588	11	Dok2
0.453801655	1	0.0001622	11	Uri1
0.634220755	1	0.0001624	11	Lpp
0.403326877	1	0.0001633	11	Anapc15
0.6707523	1	0.0001639	11	Senp1
0.601584772	1	0.0001651	11	Tspan13
0.346267847	1	0.0001653	11	Mycbp
0.965282937	1	0.0001654	11	Ccdc124
0.525547327	1	0.0001658	11	Psmd14
0.495262946	1	0.0001662	11	Zc3hav1
0.540775252	1	0.0001669	11	Sdhc
0.288973191	1	0.0001673	11	Slc7a11
0.619600056	1	0.0001688	11	Cd44
0.629406426	1	0.0001693	11	Smndc1
0.842804586	1	0.0001701	11	Pigq
0.789718968	1	0.0001708	11	Srrm1
-1.20900205	1	0.0001718	11	Chchd2
0.411519468	1	0.0001746	11	Fryl
0.568113704	1	0.0001752	11	Cyth4
0.378002473	1	0.0001775	11	Pgs1
0.364108944	1	0.000183	11	Cln5
0.44227631	1	0.0001893	11	Snhg6
0.560620619	1	0.0001901	11	Ptk2b
0.922165847	1	0.0001917	11	Prkaa1
0.355268672	1	0.0001969	11	Uck1
0.621525754	1	0.0002019	11	Igf1
0.412678274	1	0.0002026	11	Smchd1
0.671512035	1	0.0002055	11	Snx1
0.505303562	1	0.0002057	11	Abhd16a
0.509567287	1	0.000206	11	Rrp15
0.522940758	1	0.0002077	11	Rnf149
0.499563901	1	0.0002084	11	Nmi
0.855384697	1	0.0002103	11	Tpd52l2
0.679213255	1	0.0002105	11	Txnrd1
0.577326239	1	0.0002117	11	Eps8
0.880704567	1	0.0002135	11	Dennd4a
0.622456462	1	0.000216	11	Sec61a1
0.870605575	1	0.0002164	11	Mitf
0.695482441	1	0.0002164	11	Rdh11
-1.767361371	1	0.0002177	11	Aif1
-2.029699968	1	0.0002261	11	Skp1a
-0.485893261	1	0.0002268	11	Tmsb4x
0.494957288	1	0.0002268	11	Por
0.610815667	1	0.000227	11	Vcpip1
0.326069189	1	0.0002273	11	Trappc5
0.690772256	1	0.00023	11	Mgea5
0.391112855	1	0.0002321	11	Tbxas1
0.376786309	1	0.0002346	11	Smurf1
0.384098241	1	0.0002372	11	Selt
0.756741317	1	0.0002391	11	Ebi3
0.5430268	1	0.0002512	11	Itfg3
0.865808196	1	0.0002513	11	Mettl6
0.667746689	1	0.0002517	11	Lats1

0.402142156	1	0.0002547	11	Pitpnm1
0.276264251	1	0.0002553	11	Rab3ip
0.581015383	1	0.0002558	11	Ddx47
0.349019819	1	0.0002558	11	Cab39l
1.067830964	1	0.0002561	11	Mettl23
0.419619839	1	0.0002571	11	2410089E03Rik
0.549737167	1	0.0002584	11	Maea
0.321675289	1	0.0002618	11	Snrpd3
0.427292511	1	0.0002622	11	Rbm43
0.423932793	1	0.0002642	11	Tubgcp2
0.50581035	1	0.0002652	11	Atp6v0e
0.505734935	1	0.0002751	11	Marveld1
0.777763863	1	0.0002757	11	Vamp5
0.528704879	1	0.0002761	11	Ist1
0.351901665	1	0.0002786	11	Mrpl2
0.551847	1	0.0002823	11	Gba
0.499497909	1	0.0002827	11	Ppm1b
2.554647953	1	0.0002834	11	Apoe
0.604763748	1	0.0002853	11	Gadd45gip1
-1.123034757	1	0.0002867	11	Arpc4
0.544611339	1	0.0002931	11	Cep97
0.706655947	1	0.0002957	11	Myo9a
-1.150740251	1	0.0002979	11	Rps18
0.752769064	1	0.000299	11	Srgap3
0.832825185	1	0.000301	11	Zbtb21
0.982343506	1	0.0003019	11	Alg3
0.590486787	1	0.0003055	11	Pcif1
0.398747774	1	0.0003068	11	Rtf1
0.496721018	1	0.000307	11	Phkb
0.65602295	1	0.0003102	11	Tcp1
0.38327532	1	0.0003151	11	Top2b
0.680986292	1	0.0003207	11	Coa3
-1.013292524	1	0.0003225	11	Psap
0.751751702	1	0.0003314	11	Slc37a2
-1.019719746	1	0.0003375	11	Shfm1
0.383709772	1	0.0003423	11	Rapgef6
0.591045558	1	0.0003425	11	Cd40
0.575983039	1	0.0003517	11	1700025G04Rik
0.739877421	1	0.0003543	11	Vezf1
0.460531768	1	0.0003587	11	Cxcl16
0.639327436	1	0.0003644	11	Tmx3
0.64488879	1	0.0003696	11	Mob3b
0.405263668	1	0.0003741	11	Mb21d1
0.416393404	1	0.0003748	11	Lmnb1
1.075691425	1	0.0003774	11	Mob1b
0.47142838	1	0.0003777	11	Glmn
0.576236402	1	0.0003786	11	Col27a1
0.689791573	1	0.0003786	11	Paip2
0.409036408	1	0.0003901	11	Polb
0.676921	1	0.0003941	11	Rhoc
0.718425481	1	0.000396	11	Alad
0.765334943	1	0.0003966	11	Pip5k1c
0.907618813	1	0.0003988	11	Ubap2
0.411805995	1	0.0004004	11	Suclg2
0.586376177	1	0.0004021	11	Grap2
0.324705922	1	0.0004039	11	Cd24a
0.462567233	1	0.0004041	11	Snx12
-0.761235114	1	0.0004056	11	H2-K1
0.884919601	1	0.0004117	11	Polr3k
0.411817319	1	0.000414	11	Nfat5
0.521514473	1	0.0004186	11	Capg

0.622708966	1	0.0004239	11	Spp1
0.694597851	1	0.0004245	11	Zfp281
0.310455418	1	0.0004303	11	Fads1
0.573546999	1	0.000431	11	Hspa9
0.590425403	1	0.0004327	11	Antxr2
0.536738799	1	0.0004365	11	Jak1
0.491346198	1	0.0004374	11	Tma16
0.50464982	1	0.0004404	11	Papola
0.493530341	1	0.000443	11	Bsg
0.40894804	1	0.0004438	11	2510039O18Rik
0.533556885	1	0.0004475	11	Rgs10
0.471037949	1	0.0004499	11	Aldoa
0.405890504	1	0.0004523	11	AA467197
0.394260939	1	0.0004532	11	Aarsd1
0.476039079	1	0.0004533	11	Scd2
0.289477513	1	0.0004551	11	Setd3
0.50681454	1	0.0004593	11	Alg5
-0.347881592	1	0.000461	11	Fth1
0.638326666	1	0.0004655	11	Snapin
-1.736057292	1	0.0004671	11	Zfand6
0.695502051	1	0.0004674	11	Tnfrsf1a
1.066746658	1	0.0004689	11	Uvrag
0.46062127	1	0.0004713	11	Rab4b
0.441513824	1	0.0004794	11	Stt3b
0.679805515	1	0.0004866	11	Mrps16
0.648896811	1	0.0004946	11	Tmem60
0.605416314	1	0.000497	11	Mrps18a
0.795398689	1	0.0004994	11	Tmem159
1.119350877	1	0.0005094	11	Runx3
0.274059178	1	0.000516	11	Sla
0.509128435	1	0.0005235	11	Pitpnb
0.285133489	1	0.0005278	11	Dpp4
-1.441692036	1	0.0005282	11	Ier5
-1.687213791	1	0.0005344	11	Aes
0.750422301	1	0.0005347	11	Polr3b
0.486992905	1	0.0005375	11	Psmb1
0.727586886	1	0.0005428	11	Yif1a
0.466609782	1	0.0005452	11	Ecm1
0.558320774	1	0.000547	11	Mrps18c
0.554029506	1	0.0005472	11	Myo1g
0.301751882	1	0.0005533	11	Cpne8
0.936248234	1	0.0005538	11	Bola3
0.368092901	1	0.0005587	11	Sbds
0.494107223	1	0.0005613	11	S100a6
0.463082122	1	0.000562	11	Pcbd2
0.306622088	1	0.000565	11	Flt3
-1.706902314	1	0.0005679	11	Cox7b
-0.605840118	1	0.0005684	11	Rpl8
0.556761015	1	0.0005745	11	Hck
0.503586253	1	0.0005748	11	Asb2
-1.180899247	1	0.0005918	11	Rps21
0.524642494	1	0.0005936	11	Hiatl1
0.485488366	1	0.000595	11	2510009E07Rik
0.573828425	1	0.000603	11	Slc25a12
0.368281585	1	0.000607	11	Ranbp10
0.535991017	1	0.0006097	11	Katna1
0.890393822	1	0.0006098	11	Gm12942
0.436909297	1	0.0006135	11	Atp6v0b
0.587639455	1	0.0006154	11	Tgfbr1
0.501716631	1	0.0006183	11	Gab2
0.5078755	1	0.0006225	11	Gtf2a2

1.914136268	1	0.0006252	11	Otub1
0.488839077	1	0.000643	11	2410006H16Rik
0.393522022	1	0.0006512	11	Cd14
0.466471349	1	0.0006569	11	Slc3a2
0.612957263	1	0.0006587	11	Il13ra1
0.258625534	1	0.0006611	11	Ago3
-1.292742503	1	0.0006616	11	Rpl37a
0.35250462	1	0.0006644	11	Ebna1bp2
0.358604963	1	0.0006645	11	Ganab
0.314514133	1	0.0006723	11	Rcn2
0.537420446	1	0.0006729	11	Rbm5
0.299013761	1	0.0006739	11	Nmrk1
0.533000912	1	0.0006752	11	Mrpl20
0.566635917	1	0.0006764	11	Tmem173
0.38162873	1	0.0006768	11	Iqgap1
0.505902206	1	0.0006814	11	Rab11a
0.589584932	1	0.000691	11	Ctbp2
0.754805952	1	0.0006928	11	Thumpd1
0.53093016	1	0.0006929	11	Kars
0.382446592	1	0.0006951	11	Slc27a4
0.30473251	1	0.0007096	11	Prkrip1
0.443864001	1	0.0007097	11	Cbl
0.519767768	1	0.0007158	11	Fkbp2
0.328171445	1	0.0007246	11	Arhgef3
0.65099957	1	0.0007288	11	Tmem206
0.512874902	1	0.000732	11	Eed
0.510672053	1	0.0007405	11	Tfg
0.332504314	1	0.0007423	11	Fyn
0.549153329	1	0.0007455	11	Ghitm
0.620270315	1	0.0007471	11	Coro2a
-1.593857301	1	0.0007561	11	Prkcd
0.587123805	1	0.0007581	11	Hnrnpc
0.443181881	1	0.0007626	11	Ndufa2
0.44088588	1	0.0007636	11	Tmx1
0.578944751	1	0.0007707	11	Ube4b
0.688559104	1	0.0007802	11	Atp11b
0.51162339	1	0.0008051	11	Dock11
0.711929099	1	0.0008177	11	Ndufb9
0.483888834	1	0.000821	11	Sdhb
0.469027022	1	0.0008228	11	Dhx15
-2.004650393	1	0.0008244	11	Zfand2a
0.260619376	1	0.0008303	11	Tpgs2
0.540185351	1	0.0008357	11	Acaa2
0.655584079	1	0.0008465	11	Mctp1
0.405930754	1	0.0008466	11	Mrpl15
0.541428606	1	0.0008521	11	Plscr3
0.455861272	1	0.0008538	11	Sbf2
0.566255707	1	0.0008542	11	Mthfd2
0.436424754	1	0.0008571	11	Vrk1
0.261422215	1	0.0008601	11	Vps41
-1.618730643	1	0.0008656	11	Ptpn18
0.555098952	1	0.0008705	11	Med10
0.48613196	1	0.0008713	11	Rnf103
0.441173314	1	0.0008722	11	Cxcl2
0.377286893	1	0.0008752	11	Ssna1
0.41008647	1	0.0008834	11	Rab8b
0.418221632	1	0.000885	11	Homer1
0.563773577	1	0.0008925	11	Cd93
0.519459104	1	0.0008995	11	Sod2
0.63294266	1	0.0009174	11	Zdhhc3
0.526625521	1	0.0009231	11	Purb

0.513244484	1	0.0009274	11	Plec
0.377131565	1	0.0009295	11	Creb1
0.477124708	1	0.0009394	11	Nop16
0.490354302	1	0.0009493	11	Nfkbiz
0.655846374	1	0.0009575	11	1700123O20Rik
0.354676073	1	0.0009585	11	Max
0.574815199	1	0.000964	11	Naa15
0.491711228	1	0.0009687	11	Rragc
0.84843833	1	0.0009817	11	Ssbp4
0.696117065	1	0.0009828	11	Nupr1
0.566676967	1	0.0009874	11	Ube2g1
0.348561048	1	0.000988	11	Clcn4-2
-2.048587643	1	0.0009945	11	Cuta
0.540148734	1	0.0009946	11	Mapre2
0.354973974	1	0.0009998	11	Cxcl1
0.317774844	1	0.001011	11	Ube3c
0.499179466	1	0.0010181	11	Sco2
0.454529192	1	0.0010236	11	Ubfd1
-0.956379673	1	0.0010262	11	Rps19
0.420469457	1	0.0010344	11	Sik2
0.359869835	1	0.0010352	11	Mpp6
0.356674944	1	0.0010387	11	Slc9a6
0.525395308	1	0.001055	11	Rars2
0.44049453	1	0.0010603	11	Atp6ap2
0.530975753	1	0.0010641	11	Poc1b
0.87526788	1	0.0010654	11	Sh2d1b1
-1.883856576	1	0.0011015	11	Tpst2
0.35160409	1	0.0011066	11	Sipa1l1
0.561882574	1	0.0011194	11	Zdhhc20
0.383119852	1	0.0011293	11	Ccdc174
0.429114554	1	0.0011343	11	Dnajb11
0.546051395	1	0.0011408	11	Ppp2ca
-1.983964681	1	0.0011436	11	Tsc22d3
0.294189109	1	0.0011464	11	Zfp326
-1.835982373	1	0.0011502	11	Jun
0.36751691	1	0.0011561	11	Akap11
0.588804772	1	0.0011638	11	Eif6
0.716729128	1	0.0011724	11	Icmt
0.378206797	1	0.0011726	11	Thoc7
-0.934064409	1	0.0011804	11	Dusp1
0.618128803	1	0.0011807	11	Golt1b
0.525103932	1	0.0012142	11	Otud4
0.33329282	1	0.0012163	11	Acsl5
0.642184519	1	0.0012187	11	Slc39a11
-1.961745823	1	0.0012247	11	Leprotl1
0.297785937	1	0.001241	11	Mul1
0.445670682	1	0.0012475	11	Tes
-0.761092756	1	0.001254	11	Sdcbp
0.431737916	1	0.0012557	11	Cnot2
0.503093763	1	0.0012638	11	Erap1
0.614746376	1	0.0012646	11	Gxylt1
0.45320679	1	0.0012673	11	Dusp3
0.4904934	1	0.0012675	11	Dctn2
0.42302265	1	0.0012804	11	Spsb3
0.42899274	1	0.0012818	11	Zc3h7a
0.414671443	1	0.0012874	11	Frmd4a
0.273944265	1	0.0012959	11	Bicd2
0.549637225	1	0.0012982	11	Pik3c2a
0.379256549	1	0.0013245	11	Lrrfip2
0.52729966	1	0.0013328	11	Mrpl54
0.399003115	1	0.0013357	11	Lsm4

0.577352023	1	0.0013408	11	Btbd2
0.45827899	1	0.0013448	11	Rnh1
0.332958417	1	0.0013538	11	Aldh9a1
0.487407469	1	0.0013608	11	Tmem50a
0.667335508	1	0.0013664	11	Sh3pxd2a
0.404856333	1	0.001377	11	Crnkl1
0.528849929	1	0.0013848	11	Gimap4
0.474642023	1	0.0013954	11	Isoc2b
0.483268609	1	0.0013959	11	Tmem70
0.313309093	1	0.0013987	11	Pvr
-2.014775421	1	0.0014045	11	Rgl1
0.363135703	1	0.0014081	11	Slirp
-2.001789822	1	0.0014124	11	Gpnmb
0.395855061	1	0.0014137	11	Smg5
-1.435741032	1	0.001415	11	5430435G22Rik
0.254880035	1	0.0014225	11	Tbc1d9
0.410795051	1	0.0014301	11	Snrpe
-1.928703062	1	0.0014427	11	Samhd1
0.435483655	1	0.0014488	11	Rps17
0.544530564	1	0.0014686	11	Ubr5
0.382595687	1	0.0014707	11	Rell1
-1.933686798	1	0.0014756	11	Egr1
0.454607301	1	0.001481	11	Usp34
-1.99033499	1	0.0014825	11	Slc48a1
0.458465406	1	0.0014894	11	Rbl2
0.630197943	1	0.0014968	11	Hypk
0.372110123	1	0.0015032	11	Ctsa
0.51640384	1	0.0015059	11	Bcl2a1b
0.51100285	1	0.001507	11	Gm561
0.557187599	1	0.0015174	11	Srp68
0.260174029	1	0.0015225	11	Gfm2
-2.093706667	1	0.0015333	11	Tbcb
0.325532822	1	0.0015473	11	Cd2bp2
-1.82289711	1	0.0015537	11	Pla2g7
0.523990046	1	0.0015754	11	Mtmr10
0.774964315	1	0.0015903	11	Tspan3
0.33085023	1	0.0015967	11	Dst
0.502728703	1	0.0016105	11	Dnajc3
0.434758384	1	0.0016123	11	Arl8a
0.327772682	1	0.0016182	11	Pum2
0.352123174	1	0.0016251	11	Gins2
0.318102915	1	0.0016354	11	Scarb2
-1.708141109	1	0.0016578	11	Eif3d
0.441035622	1	0.0016738	11	Krtcap2
0.349904836	1	0.0017085	11	Tmem106b
0.425909089	1	0.0017219	11	Esyt2
0.496443001	1	0.001749	11	Dctn3
0.580160317	1	0.0017495	11	Pts
-0.361815532	1	0.0017512	11	Actb
-1.557622275	1	0.0017718	11	Ptpn1
-1.064105593	1	0.0017801	11	Tceb2
0.421287078	1	0.0017894	11	Pik3r5
0.675128675	1	0.0017894	11	Tmem185b
0.683104789	1	0.0017992	11	Dnajc12
0.388044868	1	0.0018042	11	Cxxc1
0.420256985	1	0.001818	11	Uhrf1bp1l
0.417853172	1	0.0018215	11	Peli1
0.397782679	1	0.0018319	11	Fam107b
0.347515693	1	0.0018623	11	Ndufaf4
0.383903901	1	0.0018694	11	Ubl3
0.453589182	1	0.0018715	11	Ldha

-1.705788261	1	0.0018789	11	Sash1
-1.30917682	1	0.0018792	11	Lyz1
0.473596512	1	0.0019076	11	Set
-1.899253419	1	0.0019096	11	Myo9b
0.250088682	1	0.0019229	11	Mbtps1
0.420745583	1	0.0019642	11	Nagpa
0.693590738	1	0.0019642	11	Cd180
-0.420936569	1	0.001973	11	Malat1
0.488194728	1	0.0019927	11	Arfgef1
0.372457823	1	0.0020387	11	Srgap2
0.267342573	1	0.0020666	11	Hist1h2bc
0.362775748	1	0.0020753	11	Zscan26
0.286969314	1	0.0020813	11	Gpr183
0.442055096	1	0.0020827	11	Trip10
0.335396019	1	0.0020893	11	Gmip
-1.080229174	1	0.0020984	11	Zfp36
0.606342223	1	0.0021183	11	Slc11a1
-1.502066774	1	0.0021286	11	Nipbl
0.340400118	1	0.002133	11	Pttg1ip
-1.966015031	1	0.00217	11	Clec4a2
0.263657543	1	0.0021732	11	Abl2
-1.850599969	1	0.0021834	11	Ubr2
-2.01845333	1	0.0021942	11	Ntpcr
0.40306753	1	0.0022109	11	Atg5
-1.882042359	1	0.002222	11	Mycbp2
-1.622635378	1	0.0022349	11	Csk
0.364631152	1	0.0022742	11	Fam20c
1.411465858	1	0.0022771	11	Ndufs7
0.511161448	1	0.002289	11	Tmbim1
0.499022439	1	0.0023062	11	Psmg2
0.504093481	1	0.0023158	11	Zbtb18
0.346426485	1	0.0023204	11	Hnrnpul2
0.389797542	1	0.002331	11	Ggnbp2
0.358346255	1	0.0023388	11	Arpc2
0.308622643	1	0.0023558	11	Eprs
0.551514719	1	0.0023743	11	Zcrb1
-1.920001051	1	0.0023906	11	1500012F01Rik
0.444316254	1	0.0024034	11	Zwint
0.722091071	1	0.0024166	11	Tns3
0.281947592	1	0.0024257	11	Smim3
0.542318523	1	0.0024448	11	Mrpl17
0.519364345	1	0.0024604	11	Tram1
0.288870303	1	0.0024629	11	Nup88
0.411689584	1	0.0024852	11	Wasl
0.683411112	1	0.0024931	11	Cmc1
0.305847959	1	0.0024955	11	Sun1
-2.09007994	1	0.0025436	11	Ppp2r5a
0.403176666	1	0.002556	11	Fam96b
-1.897508665	1	0.0025936	11	Cadm1
0.408320857	1	0.0025961	11	Msra
0.412297375	1	0.0026146	11	Slc25a4
0.429013661	1	0.002622	11	Zfp207
-1.706073008	1	0.0026269	11	Ptpro
0.668035685	1	0.0026316	11	Hras
0.502676407	1	0.0026696	11	Got2
-1.469637692	1	0.0026811	11	Yif1b
0.644818456	1	0.0026924	11	Vps25
0.464655352	1	0.0027054	11	Kpna4
0.358072018	1	0.0027137	11	Itgb1
0.389832637	1	0.0027198	11	Akap13
-1.989249819	1	0.0027556	11	Ly6a

0.360612968	1	0.0027828	11	Nav1
-1.67588848	1	0.0027832	11	Klf3
0.460264049	1	0.0028093	11	Cul3
0.293605531	1	0.0028279	11	Nup214
0.255355391	1	0.0028307	11	Ring1
0.272098351	1	0.0028727	11	Utrn
0.3847442	1	0.0028801	11	Pfkp
0.449947218	1	0.0028933	11	Bcl2
0.320609162	1	0.0029005	11	Plekhm1
0.408096224	1	0.0029014	11	Mafg
0.447443532	1	0.0029153	11	Chst11
-0.665003644	1	0.0029208	11	Gm4832
0.561288621	1	0.0029266	11	Os9
0.573835295	1	0.002964	11	Cnep1r1
0.344374277	1	0.0029651	11	Hcfc1r1
0.348488693	1	0.0029707	11	Tnfrsf1b
0.51237411	1	0.0029922	11	Tmem179b
-1.736367791	1	0.0029935	11	Ndufb2
-1.069062471	1	0.0030074	11	Hnrnpu
-1.598656498	1	0.0030119	11	Hmox2
0.54957861	1	0.003019	11	Ddx41
0.318395253	1	0.003022	11	Furin
-0.521898652	1	0.0030243	11	Rps20
0.473644913	1	0.0030488	11	Nptn
0.500354324	1	0.0030647	11	Cep63
-0.306938202	1	0.0031116	11	Nkg7
0.402693832	1	0.0032121	11	Tnpo1
0.547933101	1	0.003224	11	Uchl5
0.308356529	1	0.0032243	11	Ccnl1
0.999410276	1	0.0032368	11	Zmat5
0.269113014	1	0.0032433	11	Marcksl1
0.492160385	1	0.0032891	11	Arpc5
-1.769023306	1	0.0033097	11	Vaultrc5
-1.676346828	1	0.003374	11	Nagk
0.559855051	1	0.0033971	11	Zcchc17
0.42206397	1	0.0034037	11	Psma1
0.322159696	1	0.003463	11	Folr2
-1.76449727	1	0.0035239	11	Becn1
0.349576329	1	0.003532	11	Mtss1
0.450013385	1	0.0035329	11	Rgl2
-1.961764661	1	0.0035347	11	Mdfic
-1.654143902	1	0.0035403	11	Cebpa
0.370096709	1	0.003555	11	1600002K03Rik
0.460226466	1	0.0035851	11	Dpep2
0.350828128	1	0.0035891	11	Mfsd6
0.474653292	1	0.0036034	11	Elovl1
-0.599222998	1	0.0036274	11	Pfn1
0.397364039	1	0.003639	11	Zak
0.619857795	1	0.0036617	11	Mpv17
0.298449213	1	0.0036647	11	Rnf111
-1.44579946	1	0.0036806	11	Itm2c
0.59260308	1	0.0036903	11	Cap1
0.492286149	1	0.0037849	11	Ftsj3
0.330014104	1	0.0037975	11	Pdap1
0.604980172	1	0.0038142	11	Pls3
-1.681586594	1	0.0038164	11	Drap1
-1.96146756	1	0.0038309	11	Igsf6
0.40289205	1	0.0038951	11	Esf1
0.329347815	1	0.0039063	11	Fndc3b
-0.417201548	1	0.0039103	11	Hspa8
0.296932595	1	0.0039194	11	Cops2

0.485498414	1	0.0039588	11	Cox6a1
-0.360976075	1	0.00397	11	B2m
-1.72088513	1	0.003988	11	Arl2
0.357363753	1	0.0040128	11	Banf1
0.318795292	1	0.0040197	11	Pxn
-0.947592937	1	0.0040318	11	Myh9
0.328967649	1	0.0040345	11	Nt5e
-1.701349544	1	0.004108	11	Nek9
0.26893284	1	0.0041196	11	Mars
-1.068943871	1	0.0041303	11	Ctsc
0.355257563	1	0.0041636	11	Pgd
0.310279487	1	0.0041847	11	Bud31
-1.504008051	1	0.0042024	11	Snrpd1
0.294480612	1	0.0042153	11	Dgkd
-1.921975964	1	0.0042243	11	Irf7
-1.805696977	1	0.0042559	11	Sf3b3
-1.643677525	1	0.004263	11	Mrpl18
0.414000335	1	0.0042631	11	Ptpra
0.420894537	1	0.0042844	11	Mrps28
0.307587938	1	0.0043009	11	Mrpl40
0.274688703	1	0.0043021	11	Mrpl55
0.428266825	1	0.004308	11	Spag9
0.382772169	1	0.0043169	11	2310036O22Rik
0.437423839	1	0.0043285	11	Rnf215
-1.628848637	1	0.004355	11	Ptges3
0.266511858	1	0.004357	11	Psmb10
-1.641826358	1	0.0043905	11	Tmem126a
0.300750225	1	0.0044235	11	Rnf34
0.485073438	1	0.0044411	11	Crybg3
0.400229751	1	0.004446	11	Nckap1l
-2.100304202	1	0.0045061	11	Hspa1b
0.394375188	1	0.0045109	11	Tapbp
-0.572019456	1	0.0046276	11	Mir682
-1.699077412	1	0.0046764	11	Arhgef7
0.318264275	1	0.0046774	11	Rps6ka3
0.34516399	1	0.0046832	11	Exoc5
-0.649920245	1	0.0047155	11	Capzb
0.497742298	1	0.004756	11	Sar1a
0.391680565	1	0.0047567	11	Ssb
0.443087388	1	0.0048086	11	Rgs19
-1.791599967	1	0.0048127	11	C3
0.38269385	1	0.0048766	11	Mrpl38
-1.687501625	1	0.0048985	11	Tsn
-1.629521952	1	0.004899	11	Cant1
-1.918516238	1	0.0049079	11	Fcgr4
0.526830572	1	0.0049283	11	Sms
0.706941849	1	0.0049764	11	Sfxn2
-1.39785614	1	0.0049953	11	Zc3h13
0.521085142	1	0.0050519	11	Tagln2
0.410742165	1	0.0050545	11	Srp72
0.644690888	1	0.0050913	11	Gng5
-0.590254467	1	0.005105	11	Tpm3
-1.629521952	1	0.0051307	11	Nin
-1.664179799	1	0.0051358	11	Vipas39
0.279457965	1	0.005144	11	Gypc
0.587824943	1	0.0052844	11	Srpr
-1.892628253	1	0.0053124	11	Cpt1a
0.399484505	1	0.0053212	11	Rnf7
0.258801431	1	0.0053777	11	Trappc4
0.912745597	1	0.0054322	11	Uqcrq
0.653650466	1	0.0054331	11	Slc25a13

-1.900781535	1	0.0054401	11	Nenf
-1.949877242	1	0.0054402	11	Ms4a6b
0.815770726	1	0.0054522	11	Stx4a
0.301786063	1	0.0054566	11	Cr1l
0.316566967	1	0.0055198	11	Gaa
-1.553753222	1	0.0055239	11	Pdha1
0.356773148	1	0.0055346	11	Lcp1
0.508716297	1	0.0055411	11	Trappc6a
0.591574518	1	0.0055568	11	Mrpl11
-1.83535274	1	0.0055628	11	Sec31a
0.45795255	1	0.0055875	11	Ppapdc1b
-1.797125165	1	0.0056081	11	Gpx3
0.647517865	1	0.0056081	11	2810002D19Rik
0.374146705	1	0.0056343	11	Vps29
-1.550104323	1	0.0056427	11	Eny2
0.329331392	1	0.0056492	11	Padi2
0.281905713	1	0.0056563	11	Arhgap26
0.333384588	1	0.0056649	11	Trappc2l
-1.764271886	1	0.0057201	11	Preb
-1.307006452	1	0.0057504	11	Rpl38
0.426110195	1	0.005758	11	Trove2
-1.718602817	1	0.0057715	11	Abr
0.333636317	1	0.0057762	11	Csnk2a1
0.425155302	1	0.0057974	11	Sdc1
-1.622746094	1	0.0058235	11	Ergic1
0.791674685	1	0.0058387	11	Ssfa2
-1.798869198	1	0.0058755	11	Dnajc13
0.312566	1	0.0058919	11	Lamp2
1.365815279	1	0.005907	11	Wdr26
0.375166726	1	0.0059524	11	Lamtor5
0.267548443	1	0.0059529	11	Nfkbia
0.322673147	1	0.0059761	11	Rbx1
0.419467445	1	0.006006	11	Sgpl1
0.399579519	1	0.0060087	11	Rtn4
0.519704434	1	0.0060279	11	Slc12a4
-0.909968196	1	0.0060341	11	Mir703
-1.819489437	1	0.0060633	11	Ifi203
-1.730257269	1	0.0060782	11	Acadvl
-1.613639599	1	0.00608	11	Zfp62
0.293407469	1	0.0061108	11	H2-DMb1
0.34117417	1	0.0061187	11	Rasa1
-1.685149644	1	0.006168	11	Btg2
0.412790035	1	0.006188	11	Ndufa9
-1.641641053	1	0.00619	11	Slc16a10
-1.650521691	1	0.0062006	11	H2-K2
-1.785640307	1	0.0062102	11	Gtf3c2
0.271918359	1	0.0062155	11	Vdac1
0.375276804	1	0.0062158	11	Lims1
-1.809485534	1	0.0062663	11	Phactr2
-1.70639962	1	0.0062691	11	Adrbk2
-1.709867631	1	0.0062711	11	Sppl3
-1.534477614	1	0.0062726	11	Commd6
-1.604904863	1	0.0062748	11	Calm3
-1.793721783	1	0.0062838	11	Xpo1
-1.754745782	1	0.0062943	11	Rsrc2
-1.385277097	1	0.006322	11	Irf2bp2
0.656945074	1	0.0063719	11	Tmem43
-1.572977589	1	0.0063805	11	Emc1
0.431111233	1	0.006426	11	Zc3h11a
0.392148984	1	0.006431	11	Sqstm1
0.413416935	1	0.006484	11	P4hb

0.385842006	1	0.0065075	11	Fes
-1.6799611	1	0.0065294	11	Luc7l3
0.328692273	1	0.0065385	11	Ifi30
-1.672300389	1	0.0065861	11	Gas2l1
-1.517910044	1	0.0065872	11	Cdc5l
-1.866216739	1	0.0065912	11	Msrb1
0.615429253	1	0.0066123	11	Ccdc6
0.377403477	1	0.006614	11	Rnf11
-1.098750826	1	0.0066324	11	Ehd4
0.256002914	1	0.0066857	11	Sidt2
0.362048206	1	0.0066916	11	Rac2
-1.64275237	1	0.0067625	11	Mfsd10
0.270137838	1	0.0067625	11	Tmem126b
-1.614067037	1	0.0068656	11	Ccdc88a
-1.759176961	1	0.0068842	11	Ccdc88b
-1.646816665	1	0.0068852	11	Acbd5
-1.521051079	1	0.00689	11	Sh3bp2
-0.653122316	1	0.0069039	11	Gm6654
-1.766237166	1	0.0069774	11	Ict1
0.312275587	1	0.0070016	11	Prdx6
-1.740550113	1	0.0070132	11	Ythdf3
0.310224891	1	0.0070177	11	AI314180
-1.054595403	1	0.0070707	11	Rps25
-1.76607354	1	0.0070845	11	Slmap
0.313680452	1	0.0071054	11	Plaur
-1.808955268	1	0.0071771	11	Rnf216
0.606439493	1	0.0071972	11	Psd4
-1.587964991	1	0.0071977	11	Nkiras2
-1.621044931	1	0.0072125	11	Srsf1
-1.526264312	1	0.0072857	11	Slc1a5
-1.558799151	1	0.0073042	11	Slc35e4
0.256572906	1	0.0073393	11	Ddx24
-1.530001082	1	0.0074215	11	Mid1ip1
-1.441775492	1	0.0074298	11	Cuedc2
-1.391336421	1	0.0074777	11	Myo5a
0.389448605	1	0.0074794	11	Nceh1
-1.451356071	1	0.0075388	11	Ppfia1
-1.232099172	1	0.0075414	11	Atp1a1
0.491483929	1	0.0075573	11	BC005561
-1.924958537	1	0.007579	11	Vkorc1
-1.448418936	1	0.0075943	11	Atp13a3
-1.461161354	1	0.0076064	11	Ascc3
0.456651867	1	0.0076091	11	Gtf2f2
0.257814296	1	0.0076597	11	Trim30a
-1.597694588	1	0.0077511	11	Cwc25
0.304083791	1	0.0077769	11	Mrpl46
0.274250731	1	0.007857	11	Cisd2
0.380399126	1	0.0078743	11	Mllt11
-1.59011364	1	0.0078825	11	Kif1c
-1.772998723	1	0.0079738	11	Tmem261
-1.651035874	1	0.007983	11	Map2k4
0.30603904	1	0.0079906	11	2810004N23Rik
-1.584046491	1	0.0079908	11	Mical1
-1.537638646	1	0.0080095	11	Pdcl3
0.281841319	1	0.0080108	11	Pik3cb
-1.082986878	1	0.008012	11	Sdc3
0.350872594	1	0.0080262	11	Abhd17b
0.306283634	1	0.0080276	11	Mrpl13
0.339480626	1	0.0080351	11	Tpm4
-1.3609116	1	0.0080611	11	Klf13
-1.779241251	1	0.0080709	11	Hmgn1

-1.567818152	1	0.0081112	11	Tmem87b
0.311216823	1	0.0081143	11	Ppt1
0.283798078	1	0.0081412	11	Nip7
0.328134696	1	0.0081464	11	Chfr
0.391236671	1	0.0083203	11	Dph6
0.340167338	1	0.0083256	11	Atp6v0d1
0.401143059	1	0.0083407	11	Stk10
0.35009498	1	0.0083862	11	Zfx
0.38435541	1	0.0084543	11	Derl2
-1.670045272	1	0.0084583	11	Tnfrsf11a
0.451039728	1	0.0084922	11	Psmd1
-0.91933703	1	0.0084932	11	Rps15a
-1.60464174	1	0.0084957	11	Usp19
0.293514783	1	0.0085025	11	Herpud1
0.629448118	1	0.008504	11	Napg
-1.6847841	1	0.0085201	11	Pacsin2
0.34006119	1	0.0085501	11	S100a13
-1.726269228	1	0.0085584	11	Fam213b
-1.522095902	1	0.00861	11	Kdm1a
-1.590698834	1	0.0086121	11	Dusp22
-1.45649862	1	0.0086149	11	Fam175b
0.26775152	1	0.0086206	11	Itpripl2
-0.510928363	1	0.0087005	11	Rpl14-ps1
0.43035833	1	0.0087148	11	Ercc2
-1.721740781	1	0.0087299	11	Ctsk
0.336634193	1	0.0087347	11	Cd151
0.322013245	1	0.0087368	11	Trip12
0.369851562	1	0.0088033	11	Ino80e
-1.199293093	1	0.0088347	11	Tuba1b
0.334468681	1	0.0088357	11	Lrrc59
-1.534136737	1	0.0088379	11	Taf7
0.57441747	1	0.008839	11	Cox7a2
-1.628395826	1	0.0088674	11	Fam63a
-1.520841984	1	0.0088805	11	Wdr91
-1.478118699	1	0.0088829	11	Mrpl4
-1.823661415	1	0.0088872	11	Cyp4f18
-1.716548961	1	0.0088891	11	Tnfaip8l2
-1.74801338	1	0.0089454	11	Pih1d1
-1.584046491	1	0.0090014	11	Ero1lb
0.532886729	1	0.0090139	11	Mrpl53
0.480530476	1	0.0090244	11	Ctu2
-1.552943506	1	0.0091302	11	Pds5a
-1.516012894	1	0.0092551	11	Cln3
-1.650497199	1	0.0092588	11	Parp2
-1.653431904	1	0.0092644	11	Slfn8
-1.427637127	1	0.0092659	11	Ctdsp2
-0.933296579	1	0.0093222	11	Vapa
-1.696507767	1	0.0093579	11	Akap9
0.293909248	1	0.0093828	11	Agtpbp1
-1.530622521	1	0.0093884	11	Mkks
0.467306578	1	0.0094294	11	Rpf1
0.385865575	1	0.0094988	11	Taok3
-1.715081642	1	0.0095825	11	Dip2b
0.353425219	1	0.0096166	11	Polr2e
-1.079946336	1	0.0096221	11	Slc25a5
-1.525223837	1	0.0096731	11	Wipf2
-1.503971065	1	0.0096854	11	Nbeal1
0.395488001	1	0.009694	11	Bach2
0.516937586	1	0.0097572	11	Capza1
-1.664481741	1	0.0097847	11	Utp3
-1.340994044	1	0.0098119	11	Zfp36l2

0.442713373	1	0.0098874	11	Grn
0.270172214	1	0.0098947	11	Sfpq
-1.605719958	1	0.0099076	11	Lst1
0.472564539	1	0.0099581	11	Hbegf
-1.719108442	1	0.0099703	11	Tbrg1
-0.930964658	1	0.0099748	11	Cox6c
0.59975271	1	0.0099866	11	Emc4
